# Evolutionarily conserved bias of amino-acid usage refines the definition of PDZ-binding motif

**DOI:** 10.1186/1471-2164-12-300

**Published:** 2011-06-08

**Authors:** Takahiko Chimura, Thomas Launey, Masao Ito

**Affiliations:** 1 Laboratory for Memory and Learning, RIKEN Brain Science Institute, 2-1 Hirosawa, Wako, Saitama 351-0198, Japan; 2 Launey Research Unit for Molecular Neurocybernetics, RIKEN Brain Science Institute, 2-1 Hirosawa, Wako, Saitama 351-0198, Japan

## Abstract

**Background:**

The interactions between PDZ (PSD-95, Dlg, ZO-1) domains and PDZ-binding motifs play central roles in signal transductions within cells. Proteins with PDZ domains bind to PDZ-binding motifs almost exclusively when the motifs are located at the carboxyl (C-) terminal ends of their binding partners. However, it remains little explored whether PDZ-binding motifs show any preferential location at the C-terminal ends of proteins, at genome-level.

**Results:**

Here, we examined the distribution of the type-I (x-x-S/T-x-I/L/V) or type-II (x-x-V-x-I/V) PDZ-binding motifs in proteins encoded in the genomes of five different species (human, mouse, zebrafish, fruit fly and nematode). We first established that these PDZ-binding motifs are indeed preferentially present at their C-terminal ends. Moreover, we found specific amino acid (AA) bias for the 'x' positions in the motifs at the C-terminal ends. In general, hydrophilic AAs were favored. Our genomics-based findings confirm and largely extend the results of previous interaction-based studies, allowing us to propose refined consensus sequences for all of the examined PDZ-binding motifs. An ontological analysis revealed that the refined motifs are functionally relevant since a large fraction of the proteins bearing the motif appear to be involved in signal transduction. Furthermore, co-precipitation experiments confirmed two new protein interactions predicted by our genomics-based approach. Finally, we show that influenza virus pathogenicity can be correlated with PDZ-binding motif, with high-virulence viral proteins bearing a refined PDZ-binding motif.

**Conclusions:**

Our refined definition of PDZ-binding motifs should provide important clues for identifying functional PDZ-binding motifs and proteins involved in signal transduction.

## Background

The proteins that contain PDZ domain(s), often called PDZ proteins, play pivotal roles in dynamically organizing molecular architectures at specific intracellular regions in differentiating and differentiated cells [1,2]. Membrane proteins such as cell adhesion molecules, receptors, and channels form functional clusters within selective subcellular regions by binding to PDZ domains [2-5]. Furthermore, some PDZ proteins also anchor specific cytosolic proteins such as protein kinases, cytoskeleton-regulating enzymes and second-messenger-producing enzymes [2,6], and hence, contribute to precise signal transduction between extracellular and intracellular spaces at specific sites such as postsynaptic densities in neurons [2,7], immunological synapses in T-lymphocytes [8,9] and tight junctions in endothelial and epithelial cells [1,10].

**Figure 1:**
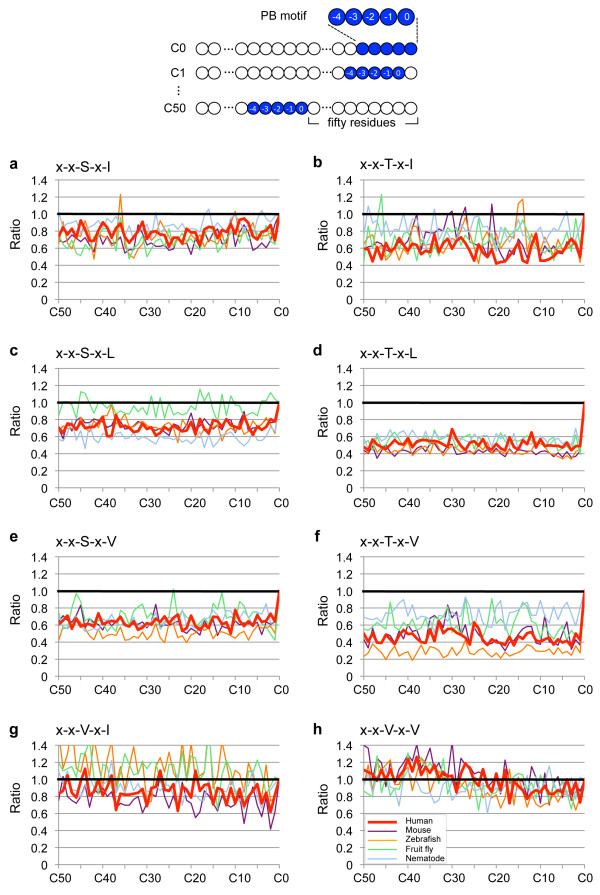
**Preferential localization of two-position-specified PB motifs at C-terminal ends of proteins**. Diagram on the top shows how positions (0, -1, -2, -3, -4) in a five-AA long PB motif are defined. Position 0 is defined as the last AA of the motif toward the C-terminal end. C0: the PB motif is located at the C-terminal end, C1: one AA away, and C50: fifty AAs away from the C-terminal end. (a-h) The "ratio" indicates the number of genes identified at each C0 to C50 position divided by the number at C0. The AAs at positions 0 and -2 are specified at the upper left in each graph. AAs are indicated with single letter code (S, serine; T, threonine; I, isoleucine; L, leucine; V, valine). × denotes any AA. Curves for the five species are shown in different colors.

PDZ domain, an evolutionarily conserved globular structure composed of 80-90 AAs (amino acids) recognizes particular regions of their interactors [6,11,12]. PDZ domains primarily bind to the C-terminal ends of proteins. Interactions between PDZ domains and internal regions of their binding partners have been also reported, though they are less common [2,11,12]. PDZ-binding motifs (hereafter 'PB motifs') have been proposed by sequence similarity in the C-terminal ends of proteins, whose bindings to PDZ domains are mediated by their C-terminal ends. PB motifs are currently categorized into at least three major types on the basis of two AAs located at positions 0 and -2 (Figure 1, upper panel), both being essential for binding to PDZ domains [11,13-15]. The type-I PB motif has the form S/T-x-I/L/V, in which Serine (S) or Threonine (T) is positioned at -2, any AA (x) at -1, and Isoleucine (I), Leucine (L) or Valine (V) at 0. The Type-II PB motif has the form Φ-x-Φ (where Φ denotes any hydrophobic AA). The type-III PB motif has the form D/E-x-Φ [11,12,14,16]. Although most of the reported PDZ-type interactions are mediated via these canonical C-terminal motifs [17], non-canonical C-terminal motifs are also reported [18-20]. As for the recognition of internal region by PDZ domains, studies based on tertiary structures revealed the binding mode of PDZ domains with internal amino acid sequences within protein fold resembling a C-terminal end such as beta-hairpin "finger"-like structure [21] and Aspartate residue whose side chain mimics C-terminal end [22], suggesting that the recognition of internal sequences by PDZ domains requires particular strict conditions. Although some internal sequences are similar to those of C-terminal PB motifs [21,23-25], this is not always the case [22,26,27] or even not identified as "motifs" [28-30]. Overall, these internal PB represents less than 5% of PDZ-PB interactions in mammals [31] and are not included in the present study.

Protein-level interaction analysis in recent decades identified a number of protein bindings to PDZ domains *in vitro *and *in vivo *and revealed that most of them possess PB motifs at their C-terminal ends. These C-terminal motifs play an essential role in the bindings to PDZ domains because deletion of C-terminal regions or mutations in these motifs abrogates the PDZ-type interactions. Furthermore, when one or more amino acid residue(s) is added to the C-terminal side of the original "functional" C-terminal PB motifs, PDZ domain cannot recognize such "hidden" PB motifs [32-35]. These results indicate that PDZ domains have a strong positional preference for PB motifs located at the C-terminal ends of proteins. The short PB motif and multiple possible substitutions lead to loose definition of the motif however, making it a very common feature in proteins. Thus its presence at the C-terminal end of a protein cannot be considered as a strong predictor of this protein being involved in a PDZ interaction. Given the central role played by PDZ interactions in signaling and protein localization, it seemed important to revisit the issue of the PB "consensus" motifs and try to refine its current definition.

In order to solve this problem, in this report, we focused on the best-characterized canonical type-I and type-II PB motifs as model systems, and performed genomics-based characterization of these PB motifs. Based on the knowledge that functional PB motifs show positional preference at the C-terminal ends in terms of protein-protein interactions, we hypothesized that, also at the genome-level, PB motifs should show similar positional preferences, because if the encoded protein is genuinely involved in a PDZ interaction important for cell functions, genomes harboring mutations within functional PB motifs at the C-terminal ends would face selective disadvantage. Thus, selection pressure would prevent mutations within the functional PB motifs at the C-terminal ends. In contrast, random creation and disruption of PB motifs by mutations are likely to occur without affecting the cell functions, thus yielding a background scatter of PB motif in proteins. In order to test this hypothesis, we measured the frequency of occurrence of PB motifs at the C-terminal ends of proteins relative to occurrence in the upstream fifty AAs region. Because PDZ domains are evolutionarily conserved [2,6,11,12,20], it is further expected that these patterns would be conserved to some extent across species. Analysis of the genomes from five species representative of major phylogenetic interval (*Homo sapiens, Mus musculus*, *Danio rerio, Drosophila melanogaster, Caenorhabditis elegans*) shows that PB motifs are indeed preferentially located at the C-terminal ends of proteins, which provides novel perspective on PB motifs.

The second point examined below is the preferential AA usage at the position surrounding 0 and -2. For the few PDZ domains proteins that have been previously studied, it has been established that the AA residues surrounding the canonical positions 0 and -2 also play a role for recognition. For example, screen of peptides or proteins binding to the well-characterized mammalian Dlg protein family members, revealed that those proteins preferentially bind to type-I PB motif containing a glutamate at position -3 [19,20,32,36]. Thus, from these particular examples it can be inferred that for most PDZ domain proteins, recognition of a PB motifs involves surrounding AAs in addition to the canonical position 0 and -2. We present a new genomics-based bioinformatics approach to identify such preferred AAs, for the most common variants of type-I and type-II PB motifs. We demonstrate biased AA usage at positions -4, -3 or -1, specifically for the PB motifs located at the C-terminal ends, relative to PB motifs found intra-proteins. In general, hydrophilic (i.e. charged and polar) AAs were favored. Importantly, our genomics-based approach also identified the AAs previously identified by interaction experiments, such as glutamate at position -3, thereby validating the accuracy of our genomics-based sieve. Moreover, the presence of these AAs at positions -4, -3 or -1 identified by genomics strongly correlates with only a few specific ontological classes, most notably transports across plasma membrane and intracellular signaling, suggesting that the presence of PB motifs containing these preferred AAs ('refined PB motifs') at C-terminal ends can serve as indicators for the function of the proteins bearing the refined motif. Consistently, several novel bindings between PDZ proteins and proteins possessing refined PB motifs were identified. We also provide evidence showing clear correlation between the AA compositions of PB motifs in influenza viral proteins and their pathogenicity. Our findings thus refine the "classical" PB motifs definition, as motifs having a strong positional preference at the C-terminal ends of proteins in terms of genomics, and several significant AA bias among the -4, -3 and -1 positions. Experiment, ontological correlation analysis and co-evolution all show that the presence of a refined sequence at the C-terminus of a protein is predictive of its genuine involvement in a PDZ-type interaction and should accelerate the elucidation of the molecular mechanisms underlying signal transduction.

## Results and discussion

### **Preferential positioning of PB motifs at the C-terminal ends**

We chose six frequently encountered variants of the type-I PB motif (S/T-x-I/L/V). From the relatively small amount of data on the type-II PB motif (Φ-x-Φ) [17], we chose the two best-characterized variants, having the form V-x-V/I [13,15,37,38]. From the protein sequences of *H. sapiens *(58192 proteins, 20787 genes), *M. musculus *(37985 proteins, 22271 genes), *D. rerio *(21235 proteins, 18208 genes), *D. melanogaster *(20698 proteins, 14057 genes) and *C. elegans *(27287 proteins, 20003 genes) (Additional files 1 and 2, also see Materials and Methods) obtained from the Ensembl genome database project [39], we identified all the proteins containing these five-AA motifs, 'x-x-S/T-x-I/L/V' or 'x-x-V-x-V/I' within their fifty five AAs C-terminal domain (Figure 1). In this study, five residues (i.e. position 0 up to -4) were taken into consideration, because point mutation analyses have demonstrated that residues playing critical roles in binding to PDZ domains are mostly located within C-terminal 4 or 5 residues [15,40].

The identified proteins were categorized as "C0, C1...C50" based on the position of the motif, with "C0" denoting a PB motif at the C-terminal end, "C1" denoting a PB motif one AA away from the C-terminal end, and "C50" denoting a PB motif fifty AAs away from the C-terminal end (Figure 1 and Additional file 1). Individual proteins in each category (i.e. C0-C50) consist of the proteins derived from different genes and also of the splice variants derived from a single gene. Since our focus is on motif conservation among proteins derived from different genes, we conserved only one record per gene to eliminate the bias that would be introduced by genes with high number of redundant splice variants. Our pilot survey revealed that 84.6%, 82.7%, 80.7%, 77.4% and 79.7% of genes in the genome of the respective species listed above encodes proteins possessing at least one PB motif at any C0-C50 positions, confirming that the PB motifs are highly common AA sequence.

The rational for our approach is that if functional motifs are preferentially located at the C-terminal end, the number of genes identified at C0 should be significantly larger than those identified at positions other than C0 (i.e. C1-C50). Indeed, we found that this was the case (Figure 1 and Additional file 3). To compare the numbers of genes at C1-C50 to that at C0, the number of genes found at each of the C1-C50 positions was divided by that at C0. We plotted these relative numbers as 'ratio' in Figure 1. The 'ratio' is found to be lower than unity (i.e. 1) in most cases, as shown in Figure 1a-h for six type-I and two type-II PB motifs, in the five species examined. Furthermore, the ratio decreases abruptly between C0 and C1 rather than gradually, as is evident for type-I PB motifs (Figure 1). These results are consistent with previous reports showing that artificially shifting PB motifs at the C1 position by addition of a single residue at the C-terminal end abolishes the interactions between PB motifs and PDZ domains [33-35]. The genes identified by the C0 position search are shown in the Additional file 4.

**Figure 2:**
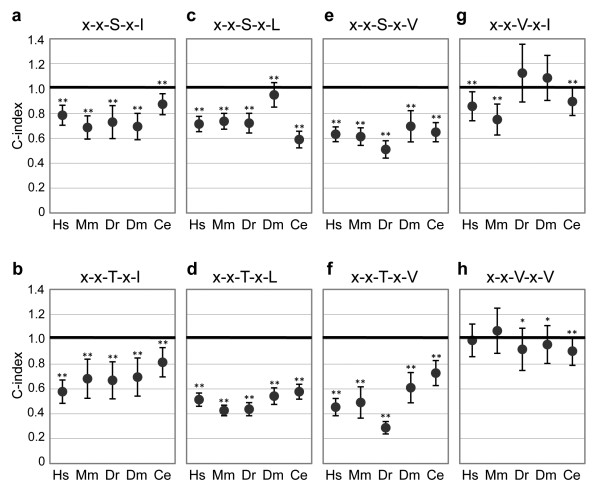
**C-indexes of two-position-specified PB motifs in five species**. The averages of the values in C1 to C50 shown in Figure 1 are plotted as C-indexes. Values significantly lower than 1 are indicated by asterisks (Mann-Whitney test, *P < 0.01, **P < 0.001). Error bars indicate standard deviations. Hs, *Homo sapiens *(human); Mm, *Mus musculus *(mouse); Dr, *Danio rerio *(zebrafish); Dm, *Drosophila melanogaster *(fruit fly); Ce, *Caenorhabditis elegans *(nematode). Actual values for each plot are shown in Additional file 3.

To statistically evaluate the overall extent to which PBs are preferentially located at C0 relative to C1-C50, we defined the 'C-index' as the average of the ratios calculated for C1-C50 positions (Figure 2).

Equation 1: Ci indicates the number of genes found with a PB at position 1 ≤ i ≤ 50.

A C-index lower than 1 implies that the PB motif is more often located at C-terminal end than within C1-C50. For example, the C-index of the x-x-T-x-V motifs in human is approximately 0.45 (Figure 2 and Additional file 3), which indicates that this motif localizes at the C-terminal end with a probability 2.2-fold higher than at C1-C50. As shown in Figure 2a-f, C-indexes are generally significantly lower than unity for type-I motifs. In contrast, for the two type-II PB motifs tested, the C-index is statistically not different from unity in several cases because of the large standard deviations of these C-indexes (Figures 2g and 2h). Therefore, among all the PB motifs considered here, the two type-II PB motifs are less preferentially positioned at the C-terminal ends of proteins than type-I PB motifs.

### **Biased amino-acid usage at positions -4, -3 and -1 of the C-terminal PB motifs**

**Figure 3:**
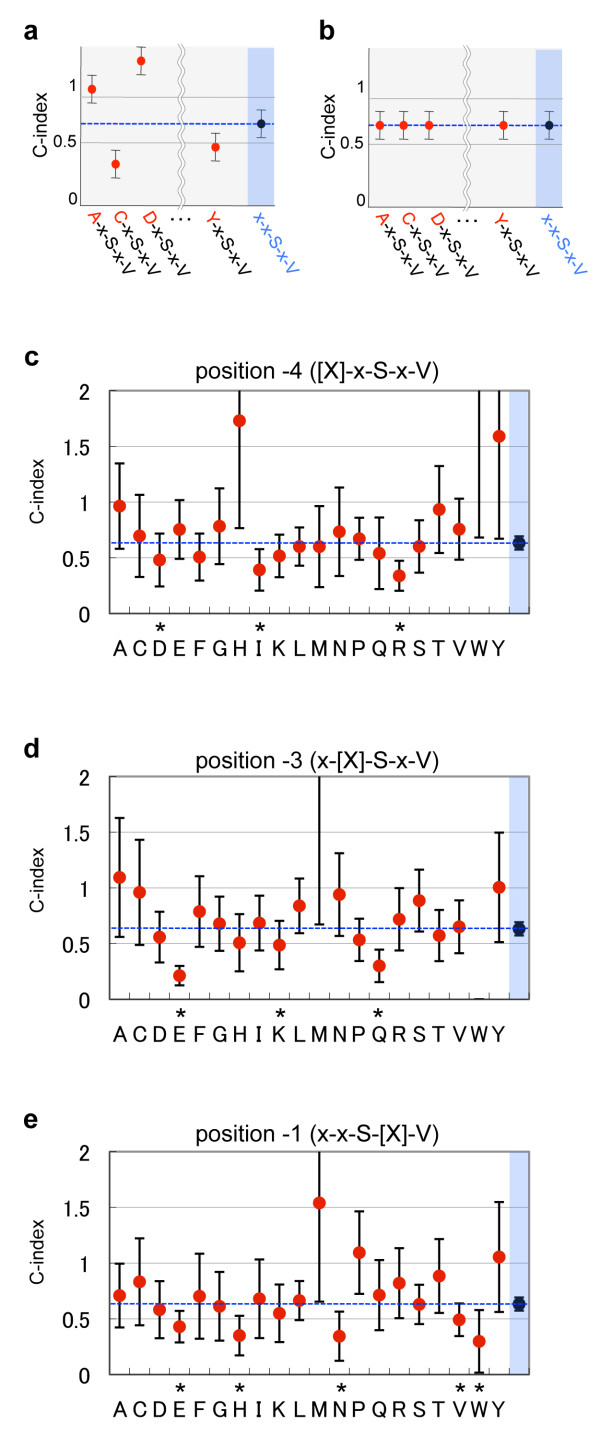
**C-indexes of three-position-specified PB motifs derived from human x-x-S-x-V**. (a and b) Expected patterns of the C-indexes of the three-position specified PB motifs when the AA compositions in the 'x' positions at C0 are different (a) or similar (b) to those at C1-C50, respectively. (c-e) Actual C-indexes of three-position specified PB motifs derived from human x-x-S-x-V. Additionally specified positions (-4 in c, -3 in d, -1 in e) in the three-position-specified PB motifs are indicated at the top of each panel with an [X]. Twenty AAs in single character code are indicated below the panels. The black filled circle in the most right column in each plot indicates the C-index of two-position-specified PB motifs, which is the same value as that shown in Figure 2. Values significantly lower than those of two-position-specified PB motifs are indicated with asterisks (Steel test, *P < 1.00E-4). Error bars indicate standard deviations. Actual values for each plot are shown in Additional file 5.

**Figure 4:**
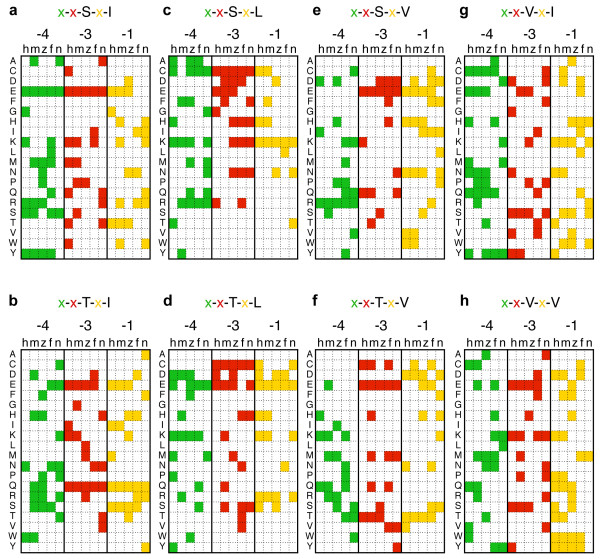
**Biased AA usages in the PB motifs located at the C-terminal ends of proteins**. Each grid indicates the AAs providing significantly lower C-indexes when placed at the positions -4, -3 or -1 in the three-position-specified PB motifs, relative to the two-position-specified PB motifs. The three colors represent positions (-4: green,-3: red, and -1: yellow). (a-h) Six and two variants of type-I (a-f) and type-II (g and h)PB motifs. Each panel contains three major columns that represent three positions (-4,-3, -1). These "position" columns are subdivided into five columns that represent the five species examined (h, human; m, mouse; z, zebrafish; f, fruit fly; n, nematode).Twenty AAs are arranged vertically in single letter code.

Next we examined whether the AAs present at the positions -4, -3 and -1 in the C0 PB motifs show any biased usage frequency compared to similar positions in PB motifs located within C1-C50. For example, if a particular AA [X] is over-represented at position -4 in x-x-S-x-V motif located at the C0 position but not for C1-C50 PB positions, the C-index of [X]-x-S-x-V would be lower than the C-index of x-x-S-x-V (e.g. C and Y in Figure 3a). In contrast, if such C0-specific over-representations is not the case, the C-index of [X]-x-S-x-V would not vary with the AA-substitution in the [X], showing values similar to that of x-x-S-x-V (Figure 3b). This approach has the advantage of determining usage bias in the context of a particular sequence, independently of the overall frequency of individual AA in proteins. Furthermore, the C-index provides an unbiased normalization that only depends on the position of this sequence along the C-terminal domain. Figure 3c-e shows the actual C-indexes and standard deviations of human x-x-S-x-V motif, in which twenty AAs are substituted in each "x" at positions -4, -3 and -1, respectively. The actual numbers of identified genes, C-indexes and their standard deviations are listed in Additional file 5. It is clearly apparent that the C-indexes of the three-position-specified PB motifs vary with the types of AAs at positions -4, -3 and -1. In the case of human x-x-S-x-V shown as an example here (Figure 3), the AA substitution D, I and R at position -4, E, K and Q at position -3, and E, H, N, V and W at position -1 all result in significantly lower C-index than that of the two-position-specified PB motif. This observation was confirmed for all the variants of the PB motif examined here (Additional file 5). These results indicate that there are specific bias for the AAs surrounding PB motifs located at the C-terminal ends. The results are summarized in the Figure 4, showing all the AA substitutions that result in significantly lower C-indexes than those of two-position specified PB motifs. Among these substitutions, note that glutamate (E) at -3 shows a very robust usage bias (Figures 3c and 4, Additional file 5), across variants of the Type-I and Type-II motifs as well as across distant species, strongly suggesting that its presence in C-terminal PB motif results from an evolutionary process. Since the PB motifs containing these AAs ('refined PB motifs') selectively appear at the C-terminal ends (Figure 3), such PB motifs are probably actually recognized by PDZ proteins. If not, random mutations during evolution would have erased this selective localization at the C-terminal ends of the refined PB motifs. Furthermore, most of the identified AAs (Figure 4) are hydrophilic ones including electrically charged AAs D, E, H, K and R, and polar N, Q, S and T. Meanwhile, hydrophobic AAs such as I, L and V, or AAs with simple side chain such as G and A are rarely identified. This is consistent with the previous reports that have demonstrated the important roles of electrostatic interactions in the bindings between PB motifs and PDZ domains [41-43]. This finding also support the notion that the refined PB motifs are likely to be recognized by PDZ domains.

The additional AAs (Figure 4) in the refined PB motif may also contribute to the regulation of PDZ binding. As a case in point, a recent report [44] about the regulation of the NR2B NMDA receptor subunit may shed some light on the physiological role of variations within refined motifs. Sanz-Clemente and collaborators have confirmed that the binding of NR2B to PDZ protein PSD-95 is attenuated by casein kinase 2 (CK2)-mediated phosphorylation of serine at position -2 within the type-I PB motifs of NR2B (-IESDV). A point mutation of E at -3 to Q (i.e. -IQSDV) abolished phosphorylation by CK2 however, while binding to PSD95 was unaffected, which is consistent with our findings that both E and Q at -3 are identified as biased amino acids at position -3 of x-x-S-x-V (Figure 4e, column '-3' and sub-column 'h' and 'm'). Thus, we suggest that selection pressure may have been directed not only toward increasing PDZ binding affinity but also toward creating both constitutive and regulated interactions.

Because amino acids identified in Figure 4 are evolutionarily conserved to some extent, orthologous genes should be commonly identified in the species studied here. As expected, this was the case. For example, our analysis revealed that human and mouse genome encode 1089 genes and 972 genes that contain at least one refined PB motifs at their C-terminal ends, respectively, in which 501 of human 1089 genes and 512 of mouse 972 genes are orthologous to each other (Additional file 6).

### **Over-representations of proteins involved in signal transductions**

To date, several approaches using bioinformatics to predict PDZ-type interactions have been implemented. For example, Giallourakis and colleagues used evolutionary conservation, similarity of coexpression profiles relative to PDZ proteins in different tissues as well as the presence of PB motifs as criteria for their predictions [31]. Several strategies based on genome-wide proteomics have also been formulated to identify binding sequences of particular PDZ domains by screening of random or genome-encoded peptide libraries *in vitro *[19,20,45]. Using such quasi-exhaustive analyses, computational simulations to calculate binding affinity between any PDZ domains and genome-encoded proteins have been performed [46-48]. Although predictions can be made as whether a protein may bind to PDZ domains, *in vitro *screening of peptides is not exempt of artifacts that may negatively affect prediction accuracy [49]. Similarly, prediction algorithms that are trained against a set of known PDZ-PB interactions may be influenced by any error or omission in the training set. Our approach is orthogonal to these studies since it does not depend *a priori *on experimental evidence and may thus provide complementary information and help identify both false positive and false negative. We used three different strategies to evaluate the quality of our predictions: An ontological analysis, a comparison with some published PDZ-type interaction data and an *in vivo *test of interactions predicted by our analysis.

If the refined PB motifs correspond to genuine evolution-selected sequences, it is expected that the proteins possessing these PB motifs would present some similarities of function. Furthermore, considering the literature on proteins with identified PDZ-type interactions [1,2,6,11,12], it is expected that proteins involved in signal transductions and membrane proteins should be over-represented in this subset of C-terminal PB motifs. In order to test *a posteriori *the quality of our predictions, we determined whether any ontological category is over-represented among the human proteins (1089 genes) that possess the refined PB motifs shown in Figure 4 based on the GO (Gene Ontology at http://www.geneontology.org/) [50] molecular function term (Table 1). This analysis showed that some functional categories are clearly over-represented among the proteins with refined PB. These include receptors of growth factors, G-protein-coupled receptors, cell adhesion molecules, ion channels, kinases, proteins involved in trafficking, GTPase-activating proteins (GAPs) and guanine nucleotide exchange factors (GEFs), all of which are important players in signal transductions. In contrast, however, no obvious function over-representation was detected for the human proteins (488 genes in total) that did not possess any of the -4, -3, -1 biases shown in Figure 4, despite the presence of a C-terminal classical PB motifs. Taking that PDZ proteins play central roles in trafficking and organizing molecular architectures containing membrane proteins and signaling proteins, the results shown in Table 1 are consistent with our refined PB motif definitions being better predictor of molecular function than the classical PB motif.

**Table 1:** GO terms over-represented in the proteins possessing PB motifs with the amino acids identified in Figure 4.

**category: Molecular Function**		**WITH the AAs**		**WITHOUT the AAs**	
	
**GO term**	**size**	**hit**	**p-value**	**hit**	**p-value**
	
transmembrane transporter activity	880	86	4.06E-13	23	5.55E-02
	
signal transducer activity	2116	149	2.26E-10	53	1.05E-02
	
substrate-specific transporter activity	936	80	2.08E-09	24	6.09E-02
	
peptide binding	177	16	4.00E-03	7	4.49E-02
	
nucleoside-triphosphatase regulator activity	406	29	5.14E-03	10	2.14E-01

Significantly (p < 0.01) over-represented GO terms (Molecular Function) in the proteins possessing PB motif WITH the amino acids shown in Figure 4 are identified by web-based tool PIPE2. The identified GO terms are indicated in the 'GO term' column. The number of genes categorized in the GO terms among the proteins possessing PB motifs is indicated in the 'hit' column. The extent of statistical significance is indicated in the 'p-value' column. Although significantly over-represented GO terms were not detected in the proteins possessing PB motifs WITHOUT the amino acids shown in Figure 4, the number of genes and their p-values are also shown in the right side ('WITHOUT AAs' column) for comparison. The total number of genes in each category in the PIPE2 database is indicated in the 'size' column. AA; amino acid.

### **Binding activities of PB motifs containing the identified amino acids**

Next, we further asked whether the presence of refined PB motifs serves as a reliable indicator for the identification of PDZ-type interactions. For this purpose, we surveyed published quasi-exhaustive interaction data [19,45], in which interactions between 157 mouse PDZ domains and 217 genome-encoded C-terminal peptides were tested *in vitro*. We found that 68 out of 217 peptides possess refined PB motifs (Additional file 7). According to the aforementioned study, 61 out of 68 are experimentally validated PDZ-binding peptides. Furthermore, 4 (Kir3.3, GluR3, EphA4 and ErbB2) out of 7 negative peptides, have already been shown to be PDZ-binding proteins in other works [33,51-55]. Thus, combined with these results, 95% (65 out of 68) were true positives.

**Figure 5:**
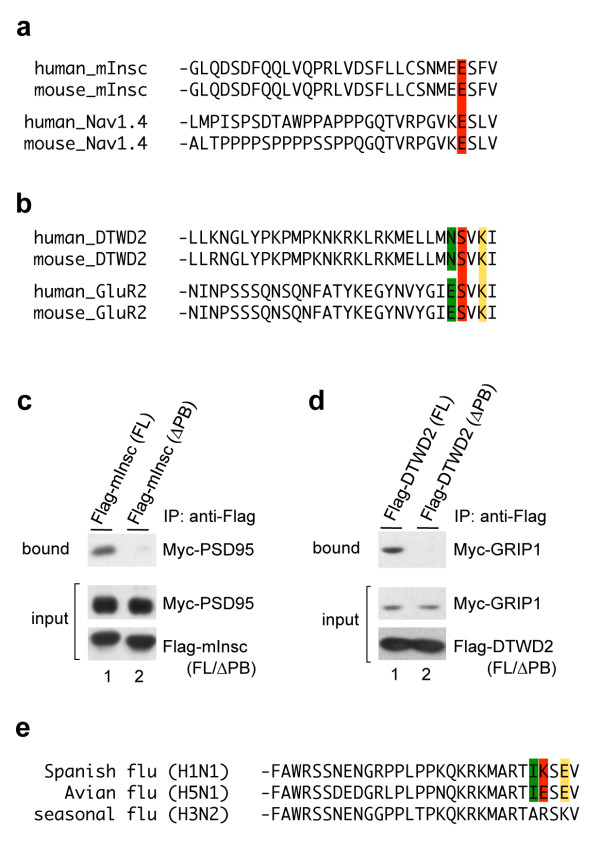
**Interactions between refined PB motifs and PDZ proteins**. (a) Comparison of the C-terminal sequences of mInsc and voltage-dependent sodium channel Nav1.4. (b) Comparison of the C-terminal sequences of DTWD2 and GluR2. AA identified in Figure 4 at each position are indicated with diffent colors (-4: green, -3: red, -1: yellow). (c) Bindings between mInsc and PSD-95 in physiological conditions, which is dependent on the PB motif of mInsc. (d) Bindings between DTWD2 and GRIP1 mediated by PB motif of DTWD2. (e) PB motifs of NS1 proteins of several Influenza strains. C-terminal thirty AA sequences of NS1 proteins are shown. Upper strain (A/Brevig Mission/1/1918(H1N1)) caused Spanish Flu in 1918 and middle (A/Hong Kong/213/2003(H5N1)) caused outbreaks of avian influenza among humans in 2003-2004. The lower (A/New York/1/2003(H3N2)) is shown as a representative example of a strain that causes seasonal flu widely and predominantly spreads among humans. AAs identified in Figure 4 are also indicated with different colors (positions - 4, -3 and -1 are indicated in green, red and yellow, respectively).

These results prompted us to test *in vivo *some of the predictions produced by *in silico *sequence analysis and to examine bindings between PDZ proteins and proteins possessing refined PB motifs shown in Figure 4. The mammalian homologue of *Drosophila *Inscuteable (mInsc) play important roles in defining cell polarity during development [56]. mInsc possess a refined type-I PB motif, x-E-S-x-V (Figure 5a), as identified in Figure 4e. However, it has not been shown whether the PB motif is functional and allows mInsc to bind to PDZ domains. This three-position specified PB motif is also observed at the C-terminal ends of voltage-dependent sodium channel Nav1.4 proteins (Figure 5a). Because Nav1.4 binds to a PDZ protein PSD-95 [52], we predicted that mInsc would also bind to PSD-95. In order to test this possibility, Flag-tagged mInsc and Myc-tagged PSD-95 were coexpressed in COS-7 cells and co-immunoprecipitation analyses were performed (Figure 5c). As expected, PSD-95 was coimmunoprecipitated by mInsc (lane 1 in Figure 5c). Furthermore, deletion of C-terminal four AAs of mInsc disruputed this binding, indicating that the binding between PSD-95 and mInsc is mediated by the PB motif of mInsc. We also tried to identify functional type-II PB motifs. As shown in Figure 5b, DTWD2, an unknown-function protein, possess three refined type-II PB motifs N-x-V-x-I, x-S-V-x-I and x-x-V-K-I, all of which are identified in Figure 4. Interestingly, two of them, x-S-V-x-I and x-x-V-K-I are also found at the C-terminal end of GluR2, a subunit of AMPA-type glutamate receptors. Considering that a PDZ protein GRIP1 binds to the type-II PB motif of the GluR2 [37,52,57], it is expected that DTWD2 also binds to GRIP1. As shown in Figure 5d, the interactions between DTWD2 and GRIP1 was indeed observed, in which the type-II PB motifs of DTWD2 was essential. Thus, we successfully identified functional PB motifs based on the three-position specified PB motifs identified in our study (Figure 4).

Finally, we tested the hypothesis that the refined PB motifs correspond to evolutionary selected sequences by examining the co-evolution of virus pathogens. Several types of virus express viral proteins that possess type-I PB motifs at their C-terminal ends and bind to cellular PDZ proteins [58]. The PB motif sequences of NS1 proteins, viral proteins of influenza, vary with the isolates of influenza strains, whose pahtogenicity can correlate with the binding activity of the PB motif of NS1 with cellular PDZ proteins [59-61]. These results prompted us to test whether NS1 derived from highly pathogenic strain possess the refined PB motifs shown in Figure 4. As shown in Figure 5e, the NS1 proteins of the highly pathogenic influenza viruses H1N1 that caused the "Spanish Flu" in 1918 and H5N1 that caused several outbreaks of Avian flu in Asia in 2003-2004 possess I-K-S-E-V and I-E-S-E-V motifs at their C-terminal ends, respectively [62,63]. These correspond to some of the refined PB motifs identified here, I-x-S-x-V, x-K-S-x-V and x-x-S-E-V in Spanish flu NS1 (Figure 5e, top row) and I-x-S-x-V, x-E-S-x-V and x-x-S-E-V in Avian flu NS1 (Figure 5e, middle row). In contrast, the PB motifs of the NS1 proteins derived from low-pathogenic strains producing seasonal flu (H3N2) correspond to a non-refined A-R-S-K-V (compare to Figure 5e, bottom row). Interestingly, two of the three-positions specified PB motifs, I-x-S-x-V and x-K-S-x-V, found in highly pathogenic strain are specifically found in human (Figure 4e, column 'h'), which may suggest that these strains of highly pathogenic influenza viruses have evolved to efficiently bind to human PDZ proteins. These results suggest that the three-position specified PB motifs should be evaluated as potential indicators of viral pathogenicities.

## Conclusions

We did a genome-level comprehensive study of the PB motif variants present in five phylogenetically distant species. We have shown that PB motifs are preferentially located at the C-terminal ends of proteins, in line with experimental results showing that PDZ interactions preferentially take place with C-terminal PB motifs. Our analysis identified specific AA usage bias for the -4, -3 and -1 positions surrounding the "classical" two-position-specified PB motifs, x-x-S/T-x-I/L/V and x-x-Φ-x-Φ. Ontological analysis of the proteins presenting this refined C-terminal PB motifs revealed very specific bias toward signaling and transport proteins. PDZ-type interactions are known to play key roles in these cellular processes, suggesting that the protein subset with refined PB motif are likely to be engaged in genuine PDZ-type interaction. By correlating motif position with sequence variation, the innovative analysis method presented here allows to detect fine variations in protein motifs, across variants and across species, while not requiring any training set. Being orthogonal with previously described strategies, we have shown that it provides a complementary approach to refine *in silico *predictions. Because these *in silico *analyses are applicable to any species whose protein sequences are comprehensively registered into databases, the methodology shown here has general applicability in discovering and evaluating any protein motif with an identified positional biases.

## Methods

### Bioinformatics

We downloaded protein sequences ('dataset_1' in Additional file 1) assigned by 'protein_coding genes' with gene ID numbers and protein ID numbers from Ensembl project http://www.ensembl.org/index.html[39] by using BioMart (Additional file 1). A version of the dataset was Release 55. Because each dataset contains the information of single species, the following procedures were separately done for the five species. After removing extraneous characters, each text line contains a single gene ID, a single protein ID and a single protein sequence. We further extracted protein sequences that contain asterisks (*) denoting stop codons and more than fifty-four AA long to perform the C0 to C50 searches ('dataset_2' in Additional file 1). Specifically for human datasets, proteins encoded in the haplotypic chromosomal regions, denoted by chromosome name HSCHR6_MHC_APD, HSCHR6_MHC_COX, HSCHR6_MHC_DBB, HSCHR6_MHC_MANN, HSCHR6_MHC_MCF, HSCHR6_MHC_QBL, HSCHR6_MHC_SSTO, HSCHR4_1 and HSCHR17_1, were removed to avoid multiple identifications of the same genes [64]. The numbers of proteins and genes in dataset_1 and _2 are shown in Additional file 2. Fifty-one data subsets ('dataset_C0' to 'dataset_C50' in Additional file 1) were generated for each species, based on the position of the motif within C0-C50, then C0-C50 searches were performed. All the Perl and UNIX scripts corresponding to these steps are available upon request to the author.

### Data analysis and statistics

All the statistical tests were performed using KyPlot 5.0 software (KyenceLab Inc. Japan). Non-parametric Mann-Whitney test or Steel test was used to examine statistical difference. *P*-values are indicated in each figure.

### Detection of over-represented GO molecular function term

The Ensembl gene IDs were converted to Entrez Gene ID using web-based tool Clone/Gene ID Converter, version 2.0 http://idconverter.bioinfo.cnio.es/[65]. The over-represented ontological categories were identified using PIPE2 http://pipe2.systemsbiology.net/PIPE2/[66] with the Entrez Gene IDs.

### Co-immunoprecipitation assay

The cDNAs encoding full-length mInsc, DTWD2, PSD-95 and GRIP1 were amplified from mouse brain cDNA libraries by PCR and subcloned into pCMV-Tag2 or pCMV-Tag3 (Clontech) for the expression of Flag-tagged or Myc-tagged proteins, respectively. As for mInsc and DTWD2, deletion mutants lacking C-terminal four AAs were also constructed. Transfection of these plasmids into COS-7 cells (RIKEN Cell Bank) was performed using Lipofectamine 2000 (Invitrogen) according to the manufacturer's protocols. Transfected cells were lysed in Tris buffer (120 mM NaCl, 1 mM EDTA, 20 mM Tris-Cl pH 7.5, 0.5% (v/v) Triton X-100, protease inhibitors cocktail) and briefly sonicated. Lysates were centrifugated (10 min; 15,000 × g) to remove insoluble matter. Anti-Flag-M2 agarose (Sigma-Aldrich) were added to the supernatant fraction and incubated for 2 hrs at 4°C. After washing, all precipitated complex were denatured in SDS sample buffer and subjected to SDS-PAGE followed by Western blot analysis using anti-Myc antibody (Santa Cruz Biotechnology) or anti-Flag antibody (Sigma-Aldrich), and chemiluminescence-based detection system ECL plus (GE Healthcare).

## Abbreviations

aa: amino acid; PB motif: PDZ (PSD-95, Dlg, ZO-1)-binding motif; GO: gene ontology; AMPA: alpha-amino-3-hydroxy-5-methylisoxazole-4-propionic acid;

## Competing interests

The authors declare that they have no competing interests.

## Authors' contributions

TC and MI designed the project; TC performed the experiments; TC, TL and MI performed the data analysis; TC, TL and MI wrote the manuscript. All authors read and approved the final manuscript.

## Supplementary Material

Additional file 1 **Schema of bioinformatics used in this study**. The blue filled squares indicate datasets that contain gene IDs and protein IDs (black filled circles) and protein sequences (horizontal lines). Asterisk (*) in each line end denotes a stop codon. Details are described in Materials and Methods. Click here for file

Additional file 2 **The number of proteins and genes in the datasets used in the bioinformatics analyses**. Click here for file

Additional file 3 **The number of genes identified by C0 to C50 searches using two-position-specified PB motifs as queries**. Click here for file

Additional file 4 **Gene IDs and five-amino-acid sequences located at the C-terminal ends of proteins isolated by each C0 search**. Click here for file

Additional file 5 **The number of genes that encode proteins possessing three-position-specified PB motifs located at C0 to C50 positions**. Click here for file

Additional file 6 **Orthologous genes between human and mouse that encode proteins possessing refined PB motifs**. Click here for file

Additional file 7 **68 C-terminal peptides of mouse genome-encoded proteins possessing refined PB motifs and their bindings to PDZ domains**. Click here for file
