# Supplementary material for: Evolutionarily conserved bias of amino-acid usage refines the definition of PDZ-binding motif
Source: BMC Genomics. 2011 Jun 8;12:300. doi: 10.1186/1471-2164-12-300 (PMC3138430; doi:10.1186/1471-2164-12-300)
Supplement: Additional file 1 — Schema of bioinformatics used in this study. The blue filled squares indicate datasets that contain gene IDs and protein IDs (black filled circles) and protein sequences (horizontal lines). Asterisk (*) in each line end denotes a stop codon. Details are described in Materials and Methods. [file 1471-2164-12-300-S1.PDF]

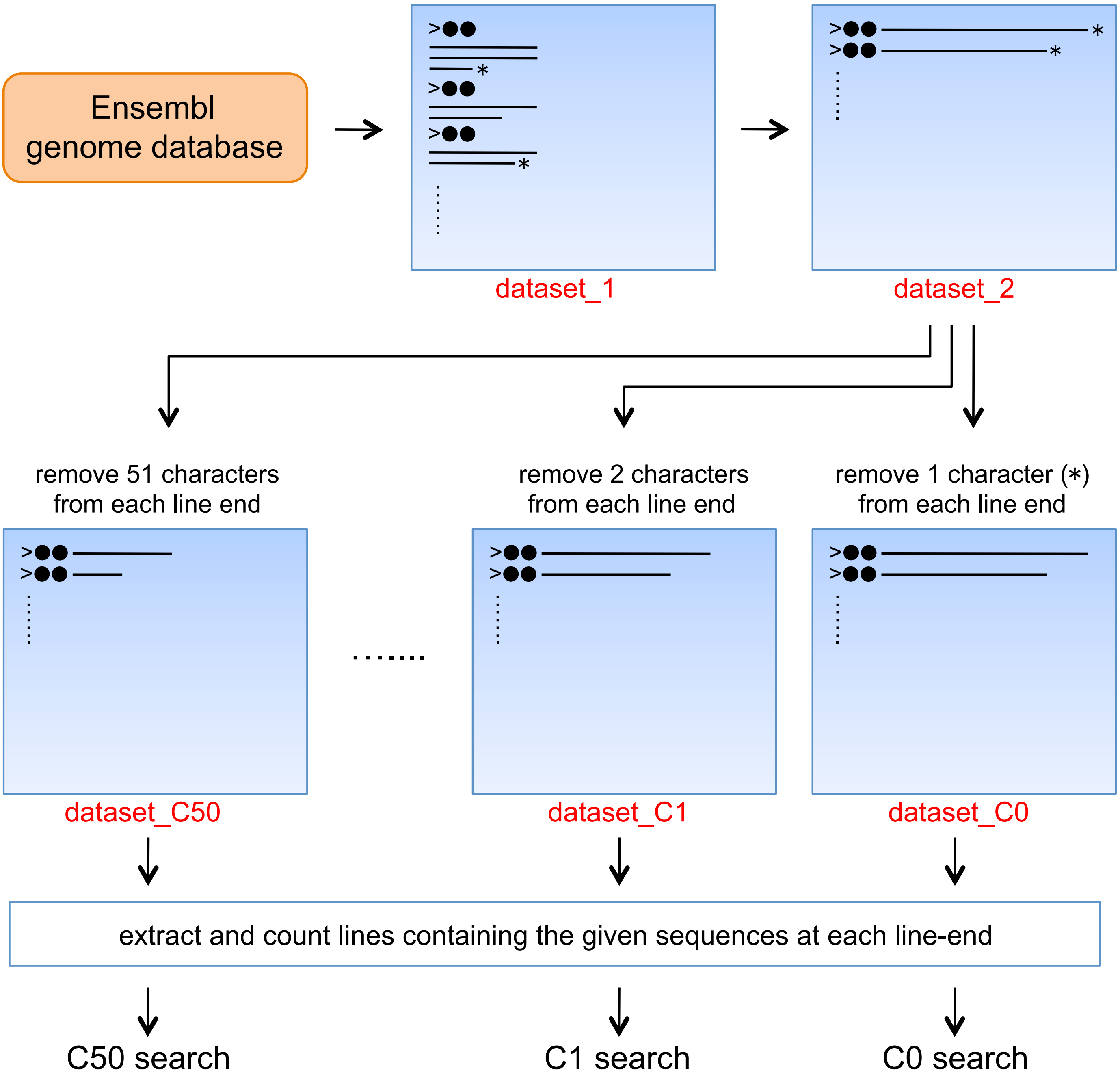

Additional file 1
Schema of bioinformatics used in this study. The blue filled squares indicate datasets that contain gene IDs and protein IDs (black filled circles) and protein sequences (horizontal lines). Asterisk (*) in each line end denotes a stop codon. Details are described in Materials and Methods.
