# Supplementary material for: Evolutionarily conserved bias of amino-acid usage refines the definition of PDZ-binding motif
Source: BMC Genomics. 2011 Jun 8;12:300. doi: 10.1186/1471-2164-12-300 (PMC3138430; doi:10.1186/1471-2164-12-300)
Supplement: Additional file 2 — The number of proteins and genes in the datasets used in the bioinformatics analyses. [file 1471-2164-12-300-S2.PDF]

Additional file 2

The number of proteins and genes in the datasets used in the bioinformatics analyses.

	human		mouse		zebrafish		fruit fly		nematode	
	protein	gene	protein	gene	protein	gene	protein	gene	protein	gene
dataset_1	75593	23279	40341	23117	28630	24147	20815	14141	27533	20158
dataset_2	58192	20787	37985	22271	21235	18208	20698	14057	27287	20003

dataset_1 is the file downloaded from Ensembl genome project. dataset_2 is generated from dataset_1 by keeping only valid amino acid sequences with gene IDs and protein IDs for the C0-C50 searches. Details are described in Materials and Methods.
