# Supplementary material for: Evolutionarily conserved bias of amino-acid usage refines the definition of PDZ-binding motif
Source: BMC Genomics. 2011 Jun 8;12:300. doi: 10.1186/1471-2164-12-300 (PMC3138430; doi:10.1186/1471-2164-12-300)
Supplement: Additional file 3 — The number of genes identified by C0 to C50 searches using two-position-specified PB motifs as queries. [file 1471-2164-12-300-S3.PDF]

Additional file 3

The number of genes identified by C0 to C50 searches using two-position-specified PB motifs as queries.

x-x-S-x-I

	C50	C49	C48	C47	C46	C45	C44	C43	C42	C41	C40	C39	C38	C37	C36	C35	C34	C33	C32	C31	C30	C29	C28	C27	C26	C25	C24	C23	C22	C21	C20	C19	C18	C17	C16	C15	C14	C13	C12	C11	C10	C9	C8	C7	C6	C5	C4	C3	C2	C1	C0	C-index	SD	*P*-value	mark
Hs	99	113	102	111	97	124	110	92	92	108	121	113	97	112	119	98	89	98	107	122	95	106	107	102	101	83	97	97	88	111	105	105	85	110	114	113	96	109	106	118	103	125	127	120	99	94	106	108	117	93	134	0.786	0.080	3.17E-20	**
Mm	88	85	88	96	91	86	92	81	71	100	82	94	83	85	93	69	74	74	111	98	81	84	79	79	81	73	75	92	82	84	67	91	77	101	86	99	105	83	84	119	79	100	111	99	73	77	84	77	88	112	127	0.687	0.093	3.17E-20	**
Dr	59	83	60	54	69	67	84	70	43	71	67	66	57	60	112	57	48	44	54	59	72	63	68	84	75	67	57	67	58	61	69	64	69	56	64	62	59	70	54	81	94	67	71	73	82	65	68	68	71	60	91	0.730	0.132	2.30E-17	**
Dm	58	49	45	53	41	39	49	44	56	56	63	63	58	43	73	54	36	46	49	57	38	45	52	54	47	63	47	53	59	51	54	48	44	57	64	62	42	47	54	59	62	50	63	52	62	59	53	66	52	49	76	0.695	0.106	3.15E-20	**
Ce	121	100	105	106	109	91	99	100	103	109	100	112	106	115	101	88	102	82	97	96	104	85	120	87	101	102	104	97	105	94	98	97	93	116	125	98	106	99	93	113	116	108	96	104	87	111	123	112	107	115	118	0.874	0.084	1.00E-14	**

x-x-S-x-L

	C50	C49	C48	C47	C46	C45	C44	C43	C42	C41	C40	C39	C38	C37	C36	C35	C34	C33	C32	C31	C30	C29	C28	C27	C26	C25	C24	C23	C22	C21	C20	C19	C18	C17	C16	C15	C14	C13	C12	C11	C10	C9	C8	C7	C6	C5	C4	C3	C2	C1	C0	C-index	SD	*P*-value	mark
Hs	275	250	294	289	266	302	273	277	278	297	325	236	237	333	279	284	274	255	259	293	269	276	239	251	278	263	285	271	258	262	301	245	315	269	249	310	304	279	314	286	279	271	330	279	243	280	265	278	314	303	390	0.715	0.062	3.18E-20	**
Mm	214	176	233	204	241	253	233	234	247	242	236	217	223	259	238	211	213	222	238	230	216	212	206	213	200	188	229	223	205	225	268	211	222	248	228	211	225	208	244	244	198	235	274	202	215	215	228	247	233	237	306	0.737	0.064	3.18E-20	**
Dr	136	148	142	142	138	169	143	134	161	144	171	173	199	153	160	177	146	146	155	159	149	144	145	139	151	156	143	108	163	148	154	122	121	142	139	144	151	112	136	143	135	154	128	135	160	147	151	168	150	130	204	0.722	0.079	3.18E-20	**
Dm	87	104	104	100	84	116	114	102	90	103	101	96	102	89	93	90	89	99	92	97	88	111	85	109	84	103	88	91	88	90	99	94	119	105	115	93	80	113	87	111	100	99	85	89	91	87	105	105	115	107	103	0.949	0.098	8.18E-04	**
Ce	127	123	143	176	125	150	103	170	128	118	143	137	127	143	146	129	131	145	126	134	130	122	115	136	130	131	126	133	120	126	116	128	148	105	141	107	120	139	141	147	133	120	110	122	148	125	124	135	160	148	224	0.590	0.067	3.18E-20	**

x-x-S-x-V

	C50	C49	C48	C47	C46	C45	C44	C43	C42	C41	C40	C39	C38	C37	C36	C35	C34	C33	C32	C31	C30	C29	C28	C27	C26	C25	C24	C23	C22	C21	C20	C19	C18	C17	C16	C15	C14	C13	C12	C11	C10	C9	C8	C7	C6	C5	C4	C3	C2	C1	C0	C-index	SD	*P*-value	mark
Hs	139	149	162	127	154	133	139	139	133	168	139	144	148	145	159	137	133	147	124	130	142	138	154	145	160	141	156	165	121	129	162	134	124	138	129	159	158	139	144	114	177	148	134	147	135	163	145	148	167	146	228	0.633	0.060	3.16E-20	**
Mm	130	144	133	129	145	169	114	137	128	123	110	133	120	130	138	120	121	125	124	129	115	131	171	118	122	121	126	131	108	102	114	107	98	133	114	134	97	119	115	112	132	122	127	125	124	128	99	129	124	129	203	0.614	0.071	3.17E-20	**
Dr	73	102	98	106	120	110	98	83	75	97	77	84	86	84	101	74	75	82	78	91	75	85	108	79	69	109	107	79	83	69	85	89	101	94	99	77	90	91	88	83	78	88	92	74	92	103	99	99	103	88	175	0.511	0.070	3.17E-20	**
Dm	73	54	55	78	94	85	63	66	57	64	61	51	57	51	75	71	58	74	79	82	66	72	60	57	62	68	99	66	74	67	65	56	61	66	59	83	76	95	66	68	63	85	80	66	73	73	60	56	48	41	97	0.697	0.126	9.14E-19	**
Ce	108	89	85	91	108	91	80	82	86	81	103	98	111	95	92	96	90	106	87	91	89	99	114	84	94	106	104	91	119	92	91	86	91	101	105	118	110	96	118	117	104	104	117	106	93	115	111	130	95	98	153	0.649	0.077	3.15E-20	**

x-x-T-x-I

	C50	C49	C48	C47	C46	C45	C44	C43	C42	C41	C40	C39	C38	C37	C36	C35	C34	C33	C32	C31	C30	C29	C28	C27	C26	C25	C24	C23	C22	C21	C20	C19	C18	C17	C16	C15	C14	C13	C12	C11	C10	C9	C8	C7	C6	C5	C4	C3	C2	C1	C0	C-index	SD	*P*-value	mark
Hs	72	71	54	74	57	80	68	73	87	77	69	79	77	64	69	67	92	67	80	86	72	83	88	80	69	55	78	66	60	71	51	53	54	62	70	81	55	52	77	93	55	55	57	61	68	67	77	86	78	60	121	0.578	0.094	3.16E-20	**
Mm	51	53	54	58	56	50	48	53	58	54	59	72	86	50	68	67	67	71	69	85	89	61	81	93	58	56	54	53	43	96	56	46	38	50	45	61	54	42	57	64	43	54	48	54	47	46	58	53	59	45	86	0.682	0.157	1.73E-15	**
Dr	48	54	56	43	47	34	38	45	34	44	39	64	63	47	47	48	48	43	40	47	53	51	60	71	57	45	31	52	59	45	57	44	50	55	53	83	87	41	57	47	42	54	46	38	52	43	44	47	51	32	74	0.669	0.149	2.31E-17	**
Dm	25	49	34	51	70	28	31	43	33	44	45	38	26	40	36	28	36	29	40	48	45	48	36	43	39	36	54	36	38	44	33	37	48	43	45	47	53	46	38	34	33	31	35	26	35	37	45	32	54	36	57	0.695	0.153	9.10E-19	**
Ce	63	93	76	79	63	78	63	70	69	86	66	80	80	67	53	63	90	59	69	66	57	60	75	69	67	82	83	72	73	61	48	72	66	57	65	80	64	63	55	67	72	56	65	71	68	79	88	58	60	74	85	0.814	0.118	1.00E-14	**

x-x-T-x-L

	C50	C49	C48	C47	C46	C45	C44	C43	C42	C41	C40	C39	C38	C37	C36	C35	C34	C33	C32	C31	C30	C29	C28	C27	C26	C25	C24	C23	C22	C21	C20	C19	C18	C17	C16	C15	C14	C13	C12	C11	C10	C9	C8	C7	C6	C5	C4	C3	C2	C1	C0	C-index	SD	*P*-value	mark
Hs	149	137	176	158	149	142	157	174	160	158	181	148	169	174	174	170	171	167	158	149	214	177	153	137	164	170	154	178	145	140	160	144	136	184	149	139	173	159	202	147	178	152	155	153	153	147	164	137	152	152	311	0.514	0.053	3.17E-20	**
Mm	136	125	151	107	134	156	129	114	133	137	132	123	143	151	131	160	161	137	128	153	158	121	123	127	134	122	138	128	138	126	143	124	113	141	123	139	135	132	134	120	131	134	127	113	127	111	131	110	114	133	309	0.427	0.042	3.17E-20	**
Dr	83	89	114	88	99	91	85	93	89	100	115	80	91	99	104	84	118	99	80	95	105	98	89	94	89	87	106	94	114	85	92	91	85	94	84	85	83	88	93	114	110	103	86	82	78	72	80	71	90	96	212	0.437	0.052	3.16E-20	**
Dm	63	67	53	73	58	61	73	79	69	59	73	62	63	66	74	60	82	68	70	74	73	56	70	73	76	55	72	66	54	56	63	78	66	63	68	66	69	74	67	61	69	57	68	72	52	56	59	52	75	47	121	0.542	0.067	3.14E-20	**
Ce	92	71	82	100	86	91	107	90	101	105	93	106	94	85	104	86	102	94	91	99	90	91	91	87	84	99	91	96	96	76	100	90	98	75	100	71	92	82	80	93	95	90	78	88	91	81	86	78	109	77	157	0.578	0.060	3.14E-20	**

x-x-T-x-V

	C50	C49	C48	C47	C46	C45	C44	C43	C42	C41	C40	C39	C38	C37	C36	C35	C34	C33	C32	C31	C30	C29	C28	C27	C26	C25	C24	C23	C22	C21	C20	C19	C18	C17	C16	C15	C14	C13	C12	C11	C10	C9	C8	C7	C6	C5	C4	C3	C2	C1	C0	C-index	SD	*P*-value	mark
Hs	105	85	106	83	82	102	91	79	100	114	104	105	93	84	82	116	86	135	106	116	117	107	102	95	78	132	118	87	77	91	84	82	77	97	76	99	92	106	109	88	85	91	95	78	91	83	87	74	105	89	210	0.454	0.069	3.17E-20	**
Mm	81	62	105	76	74	93	89	73	99	82	113	98	101	75	75	127	133	124	107	156	128	141	90	113	68	107	91	82	70	72	80	70	56	74	78	92	104	109	109	73	70	134	75	74	68	94	119	59	80	68	187	0.491	0.126	3.18E-20	**
Dr	50	59	82	70	60	42	62	76	51	58	62	40	59	79	55	75	72	65	47	53	73	53	65	91	58	55	66	71	58	49	59	46	61	51	56	60	67	80	76	64	63	63	69	55	72	58	63	53	77	59	216	0.288	0.050	3.16E-20	**
Dm	42	41	44	48	45	42	66	44	41	48	44	51	41	43	52	37	53	50	53	62	39	45	37	70	46	49	44	35	46	69	47	51	46	34	48	57	66	62	49	47	34	46	38	33	40	45	32	41	36	38	76	0.610	0.121	3.15E-20	**
Ce	68	66	65	67	62	73	72	77	84	88	69	82	65	69	68	65	50	70	69	63	74	69	69	91	80	53	78	61	57	56	80	87	53	73	74	76	82	85	70	70	87	69	61	80	65	78	85	72	58	79	98	0.727	0.100	3.14E-20	**

Additional file 3 continued.

x-x-V-x-I

	C50	C49	C48	C47	C46	C45	C44	C43	C42	C41	C40	C39	C38	C37	C36	C35	C34	C33	C32	C31	C30	C29	C28	C27	C26	C25	C24	C23	C22	C21	C20	C19	C18	C17	C16	C15	C14	C13	C12	C11	C10	C9	C8	C7	C6	C5	C4	C3	C2	C1	C0	C-index	SD	*P*-value	mark
Hs	74	77	93	75	70	87	100	72	76	89	72	81	93	57	74	73	73	74	75	79	61	80	81	98	78	77	84	56	82	78	70	98	77	83	84	63	80	78	62	80	64	79	61	71	77	84	64	75	55	73	89	0.858	0.117	5.27E-13	**
Mm	67	92	71	67	64	71	83	60	79	74	80	71	76	59	64	62	62	68	71	54	64	74	71	92	71	59	72	58	86	67	64	49	55	77	72	64	56	72	63	60	63	72	43	67	81	62	50	70	37	55	89	0.751	0.124	2.30E-17	**
Dr	73	53	61	76	53	51	62	48	70	56	43	58	68	43	54	41	54	65	50	57	34	35	54	59	51	62	51	46	68	47	67	47	49	51	77	42	49	68	56	44	52	50	40	56	49	38	37	55	35	37	47	1.124	0.232	−	−
Dm	48	44	45	46	41	52	52	58	41	33	47	46	36	34	43	52	47	42	43	48	45	48	48	39	35	47	47	39	53	39	51	50	40	43	63	40	26	42	49	44	33	36	26	44	43	49	43	33	37	41	40	1.086	0.181	−	−
Ce	90	78	90	72	71	65	75	85	74	72	77	80	100	69	78	68	84	71	78	68	71	69	77	71	77	76	67	87	57	74	66	67	62	62	82	57	70	71	70	81	72	60	74	76	91	66	54	69	72	78	82	0.895	0.112	9.29E-11	**

x-x-V-x-V

	C50	C49	C48	C47	C46	C45	C44	C43	C42	C41	C40	C39	C38	C37	C36	C35	C34	C33	C32	C31	C30	C29	C28	C27	C26	C25	C24	C23	C22	C21	C20	C19	C18	C17	C16	C15	C14	C13	C12	C11	C10	C9	C8	C7	C6	C5	C4	C3	C2	C1	C0	C-index	SD	*P*-value	mark
Hs	131	128	125	116	131	117	132	115	122	130	146	129	149	124	135	127	138	119	128	122	123	100	126	140	133	116	109	132	110	101	126	103	131	85	115	109	103	91	99	103	100	109	98	98	103	100	118	98	114	86	118	0.990	0.131	8.56E-01	n.s.
Mm	130	127	93	116	111	85	112	88	104	111	120	113	131	105	114	127	103	111	98	95	134	84	116	109	107	99	101	99	84	90	94	77	114	89	91	92	91	74	107	95	67	100	65	89	96	73	93	70	89	80	93	1.067	0.182	−	−
Dr	77	97	72	100	92	97	102	78	78	90	95	72	89	104	99	70	82	91	84	86	85	73	76	82	64	85	83	65	70	65	68	55	65	93	58	85	72	56	69	59	56	59	60	69	61	69	68	67	53	66	83	0.918	0.170	5.50E-03	*
Dm	50	48	46	74	49	60	47	52	51	60	76	63	71	72	65	69	65	55	72	48	57	57	55	51	56	55	37	54	53	48	49	56	60	56	60	62	61	51	56	45	61	53	43	56	47	58	49	38	50	47	58	0.957	0.152	1.65E-03	*
Ce	84	96	75	94	87	102	82	80	62	91	100	90	88	77	75	76	65	68	85	89	68	74	77	77	86	78	79	87	54	76	81	76	84	75	73	81	77	94	70	93	78	87	104	83	77	87	72	92	69	92	90	0.904	0.115	7.99E-08	**

The numbers of genes that encode proteins possessing two-position-specified PB motifs at C0 to C50 positions are indicated in each grid. The amino acid sequence of two-position-specified PB motif is shown at the upper left of each panel. C-indexes and their standard deviations (SD) are shown at the right of each panel. C-index significantly lower than 1 is indicated by asterisk(s) (Mann-Whitney test, *$P < 0.01$, **$P < 0.001$). Hs, *Homo sapiens* (human); Mm, *Mus musculus* (mouse); Dr, *Danio rerio* (zebrafish); Dm, *Drosophila melanogaster* (fruit fly); Ce, *Caenorhabditis elegans* (nematode).
