# Supplementary material for: Evolutionarily conserved bias of amino-acid usage refines the definition of PDZ-binding motif
Source: BMC Genomics. 2011 Jun 8;12:300. doi: 10.1186/1471-2164-12-300 (PMC3138430; doi:10.1186/1471-2164-12-300)
Supplement: Additional file 4 — Gene IDs and five-amino-acid sequences located at the C-terminal ends of proteins isolated by each C0 search. [file 1471-2164-12-300-S4.PDF]

Additional file 4. Gene IDs and five-amino-acid sequences located at the C-terminal ends of proteins isolated by each C0 search

ID numbers of genes which encode proteins possessing the PB motifs at their C-terminal ends (i.e. C0 positions) are listed with the actual five-amino-acid sequences.

human xxSxI

No.	gene ID	5aa	No.	gene ID	5aa	No.	gene ID	5aa
1	>ENSG00000116729	AASGI	51	>ENSG00000205669	KHSKI	101	>ENSG00000175538	RVSMI
2	>ENSG00000136560	AESSI	52	>ENSG00000154814	KISLI	102	>ENSG00000112394	SDSII
3	>ENSG00000164124	AFSKI	53	>ENSG00000179195	KISVI	103	>ENSG00000162769	SESAI
4	>ENSG00000116209	AHSEI	54	>ENSG00000198604	KKSRI	104	>ENSG00000123146	SESGI
5	>ENSG00000184716	AISCI	55	>ENSG00000222017	KQSCI	105	>ENSG00000167130	SFSCI
6	>ENSG00000174374	AKSFI	56	>ENSG00000112333	KSSDI	106	>ENSG00000144339	SISDI
7	>ENSG00000102144	ALSNI	57	>ENSG00000115607	KSSII	107	>ENSG00000197969	SKSLI
8	>ENSG00000101323	AVSKI	58	>ENSG00000091181	KSSVI	108	>ENSG00000011304	SKSTI
9	>ENSG00000102901	AWSQI	59	>ENSG00000179134	KTSTI	109	>ENSG00000117569	SKSTI
10	>ENSG00000126785	CCSII	60	>ENSG00000073803	LGSAI	110	>ENSG00000119314	SKSTI
11	>ENSG00000004468	CTSEI	61	>ENSG00000162654	LGSRI	111	>ENSG00000154269	SMSPI
12	>ENSG00000224130	CWSGI	62	>ENSG00000144741	LISAI	112	>ENSG00000187546	SNSAI
13	>ENSG00000099834	DDSYI	63	>ENSG00000187790	LKSDI	113	>ENSG00000166927	SRSWI
14	>ENSG00000197991	DISNI	64	>ENSG00000146410	LNSRI	114	>ENSG00000205336	SSSRI
15	>ENSG00000093144	EASVI	65	>ENSG00000164100	LPSPI	115	>ENSG00000108506	SVSGI
16	>ENSG00000065325	EESEI	66	>ENSG00000054179	LPSTI	116	>ENSG00000215545	SYSHI
17	>ENSG00000170777	EGSRI	67	>ENSG00000099899	LQSPI	117	>ENSG00000100196	SYSSI
18	>ENSG00000120586	EHSVI	68	>ENSG00000160097	LRSKI	118	>ENSG00000010610	TCSPI
19	>ENSG00000183748	EHSVI	69	>ENSG00000144130	LSSAI	119	>ENSG00000165730	TESGI
20	>ENSG00000198933	ENSKI	70	>ENSG00000126091	LSSGI	120	>ENSG00000134278	TISEI
21	>ENSG00000137269	EPSYI	71	>ENSG00000204540	LSSLI	121	>ENSG00000204084	TKSLI
22	>ENSG00000006042	EQSAI	72	>ENSG00000078403	LVSLI	122	>ENSG00000206530	TVSLI
23	>ENSG00000010626	EQSLI	73	>ENSG00000126773	LWSQI	123	>ENSG00000128849	VASQI
24	>ENSG00000187510	ESSEI	74	>ENSG00000129084	LWSVI	124	>ENSG00000215103	VLSSI
25	>ENSG00000145911	ESSKI	75	>ENSG00000206150	LYSGI	125	>ENSG00000187893	VMSTI
26	>ENSG00000174368	ETSHI	76	>ENSG00000056291	NSSEI	126	>ENSG00000197646	VNSAI
27	>ENSG00000106018	ETSVI	77	>ENSG00000119042	PASWI	127	>ENSG00000197576	VPSSI
28	>ENSG00000002933	EVSGI	78	>ENSG00000165511	PCSVI	128	>ENSG00000167614	WQSSI
29	>ENSG00000103023	FCSSI	79	>ENSG00000230642	PESRI	129	>ENSG00000139865	YASVI
30	>ENSG00000188938	FLSPI	80	>ENSG00000187959	PGSKI	130	>ENSG00000107968	YCSFI
31	>ENSG00000188800	FTSPI	81	>ENSG00000163618	PHSAI	131	>ENSG00000136960	YESEI
32	>ENSG00000148634	GFSLI	82	>ENSG00000178149	PLSHI	132	>ENSG00000012174	YISSI
33	>ENSG00000176842	GMSDI	83	>ENSG00000132613	PSSPI	133	>ENSG00000182899	YPSRI
34	>ENSG00000139144	GNSII	84	>ENSG00000196517	QDSRI	134	>ENSG00000166896	YYSNI
35	>ENSG00000161610	GQSGI	85	>ENSG00000127081	QLSSI			
36	>ENSG00000164871	GRSGI	86	>ENSG00000074803	QTSLI			
37	>ENSG00000178287	GRSGI	87	>ENSG00000165660	QTSQI			
38	>ENSG00000119685	GSSFI	88	>ENSG00000144868	QVSEI			
39	>ENSG00000113522	GTSFI	89	>ENSG00000205835	QVSTI			
40	>ENSG00000152463	GVSSI	90	>ENSG00000130826	RCSSI			
41	>ENSG00000213390	HCSLI	91	>ENSG00000168135	RESAI			
42	>ENSG00000114982	HESTI	92	>ENSG00000123700	RESEI			
43	>ENSG00000178385	HYSQI	93	>ENSG00000184185	RESEI			
44	>ENSG00000102290	ICSEI	94	>ENSG00000142046	RESLI			
45	>ENSG00000110060	IKSII	95	>ENSG00000185973	RGSLI			
46	>ENSG00000136546	IQSQI	96	>ENSG00000160094	RISLI			
47	>ENSG00000135074	ISSKI	97	>ENSG00000173612	RMSSI			
48	>ENSG00000114739	KESSI	98	>ENSG00000080293	RTSII			
49	>ENSG00000164691	KESYI	99	>ENSG00000140350	RTSRI			
50	>ENSG00000082497	KGSKI	100	>ENSG00000020577	RTSTI			

Additional file 4. continued.

human xxSxL

No.	gene ID	5aa	No.	gene ID	5aa	No.	gene ID	5aa	No.	gene ID	5aa	No.	gene ID	5aa
1	>ENSG00000188000	AASCL	51	>ENSG00000139890	DLSVL	101	>ENSG00000124780	GGSHL	151	>ENSG00000151092	KFSDL	201	>ENSG00000102910	LNSKL
2	>ENSG00000166035	AASEL	52	>ENSG00000164949	DLSVL	102	>ENSG00000166762	GGSHL	152	>ENSG00000130313	KHSTL	202	>ENSG00000179934	LPSGL
3	>ENSG00000128340	ACSLL	53	>ENSG00000166592	DLSVL	103	>ENSG00000101298	GGSQL	153	>ENSG00000164761	KISCL	203	>ENSG00000138622	LPSNL
4	>ENSG00000113263	AESGL	54	>ENSG00000175220	DPSGL	104	>ENSG00000077150	GGSRL	154	>ENSG00000102057	KISSL	204	>ENSG00000112761	LPSSL
5	>ENSG00000174876	AESKL	55	>ENSG00000140564	DQSAL	105	>ENSG00000136051	GGSRL	155	>ENSG00000172774	KISSL	205	>ENSG00000161533	LQSKL
6	>ENSG00000187733	AESKL	56	>ENSG00000214717	DSSFL	106	>ENSG00000160908	GGSRL	156	>ENSG00000197887	KISSL	206	>ENSG00000158270	LSSAL
7	>ENSG00000197839	AESKL	57	>ENSG00000165959	DVSRL	107	>ENSG00000198208	GGSRL	157	>ENSG00000011114	KKSAL	207	>ENSG00000129116	LSSFL
8	>ENSG00000237763	AESKL	58	>ENSG00000224373	DVSSL	108	>ENSG00000198556	GGSRL	158	>ENSG00000163093	KLSKL	208	>ENSG00000145365	LSSLL
9	>ENSG00000224383	AESLL	59	>ENSG00000185614	DVSYL	109	>ENSG00000146576	GHSRL	159	>ENSG00000005187	KLSLL	209	>ENSG00000236302	LSSML
10	>ENSG00000138472	AESNL	60	>ENSG00000113721	EDSFL	110	>ENSG00000196826	GLSGL	160	>ENSG00000181544	KLSNL	210	>ENSG00000099822	LSSNL
11	>ENSG00000198663	AFSEL	61	>ENSG00000134853	EDSFL	111	>ENSG00000197857	GLSGL	161	>ENSG00000204634	KLSNL	211	>ENSG00000138771	LTSPL
12	>ENSG00000170803	AHSTL	62	>ENSG00000161835	EESQL	112	>ENSG00000138835	GLSLL	162	>ENSG00000101624	KLSTL	212	>ENSG00000142182	LTSSL
13	>ENSG00000188124	AHSTL	63	>ENSG00000196652	EGSLL	113	>ENSG00000215854	GSSHL	163	>ENSG00000103150	KNSKL	213	>ENSG00000123342	MDSLL
14	>ENSG00000185985	AISQL	64	>ENSG00000186105	EHSAL	114	>ENSG00000188994	GSSWL	164	>ENSG00000205535	KNSSL	214	>ENSG00000064547	MDSTL
15	>ENSG00000144230	AKSEL	65	>ENSG00000132932	EISPL	115	>ENSG00000185888	GWSVL	165	>ENSG00000227226	KNSSL	215	>ENSG00000168229	MESSL
16	>ENSG00000182896	AKSGL	66	>ENSG00000180613	EISPL	116	>ENSG00000196449	HASYL	166	>ENSG00000145604	KPSCL	216	>ENSG00000176463	MESVL
17	>ENSG00000171714	AKSTL	67	>ENSG00000007171	EMSAL	117	>ENSG00000196739	HGSHL	167	>ENSG00000204947	KPSSL	217	>ENSG00000150676	MKSFL
18	>ENSG00000106211	ALSAL	68	>ENSG00000167494	EMSAL	118	>ENSG00000157353	HGSRL	168	>ENSG00000150093	KQSGL	218	>ENSG00000136928	MVSGL
19	>ENSG00000080854	ALSKL	69	>ENSG00000161179	EPSLL	119	>ENSG00000004766	HISLL	169	>ENSG00000140563	KRSAL	219	>ENSG00000169252	NDSLL
20	>ENSG00000095794	ALSLL	70	>ENSG00000009790	EPSPL	120	>ENSG00000162437	HKSCL	170	>ENSG00000181214	KRSFL	220	>ENSG00000144218	NFSPL
21	>ENSG00000124608	ALSQL	71	>ENSG00000143889	ERSPL	121	>ENSG00000141556	HLSGL	171	>ENSG00000129493	KTSFL	221	>ENSG00000134242	NFSWL
22	>ENSG00000131142	ANSGL	72	>ENSG00000139218	ERSYL	122	>ENSG00000158006	HLSSL	172	>ENSG00000078795	KVSAL	222	>ENSG00000205740	NLSPL
23	>ENSG00000162636	APSLL	73	>ENSG00000164588	FASNL	123	>ENSG00000176381	HLSTL	173	>ENSG00000171385	KVSAL	223	>ENSG00000154305	NPSTL
24	>ENSG00000185955	AQSPL	74	>ENSG00000177453	FCSIL	124	>ENSG00000151687	HNSYL	174	>ENSG00000107815	KVSGL	224	>ENSG00000111674	NPSVL
25	>ENSG00000011566	ARSNL	75	>ENSG00000189403	FFSCL	125	>ENSG00000156711	HPSLL	175	>ENSG00000128512	KVSQL	225	>ENSG00000196277	NQSNL
26	>ENSG00000167702	ARSPL	76	>ENSG00000223507	FFSFL	126	>ENSG00000165152	HPSLL	176	>ENSG00000103043	KVSSL	226	>ENSG00000167881	NSSLL
27	>ENSG00000144619	ARSSL	77	>ENSG00000135241	FFSKL	127	>ENSG00000188707	HRSLL	177	>ENSG00000178115	LASCL	227	>ENSG00000005206	NTSLL
28	>ENSG00000152954	ASSEL	78	>ENSG00000130167	FFSSL	128	>ENSG00000152076	HRSVL	178	>ENSG00000188626	LASCL	228	>ENSG00000060140	NYSML
29	>ENSG00000173530	ATSCL	79	>ENSG00000197863	FISLL	129	>ENSG00000163040	HRSVL	179	>ENSG00000206127	LASCL	229	>ENSG00000232405	PCSFL
30	>ENSG00000204410	ATSIL	80	>ENSG00000132205	FLSHL	130	>ENSG00000138050	HSSGL	180	>ENSG00000232653	LASCL	230	>ENSG00000067829	PCSLL
31	>ENSG00000140876	ATSRL	81	>ENSG00000116191	FLSLL	131	>ENSG00000214882	HYSSL	181	>ENSG00000168389	LASIL	231	>ENSG00000072041	PESDL
32	>ENSG00000054654	CASYL	82	>ENSG00000156136	FLSTL	132	>ENSG00000164647	ICSQL	182	>ENSG00000129933	LASLL	232	>ENSG00000197106	PESEL
33	>ENSG00000162909	CFSVL	83	>ENSG00000196502	FRSEL	133	>ENSG00000068831	IDSVL	183	>ENSG00000161217	LCSWL	233	>ENSG00000170385	PESSL
34	>ENSG00000173464	CGSKL	84	>ENSG00000197165	FRSEL	134	>ENSG00000126432	IISQL	184	>ENSG00000169413	LDSIL	234	>ENSG00000135597	PFSHL
35	>ENSG00000159445	CGSRL	85	>ENSG00000213599	FRSEL	135	>ENSG00000148488	INSIL	185	>ENSG00000161634	LDSVL	235	>ENSG00000168597	PFSLL
36	>ENSG00000145708	CLSGL	86	>ENSG00000213648	FRSEL	136	>ENSG00000166006	IPSIL	186	>ENSG00000067798	LESTL	236	>ENSG00000129255	PFSQL
37	>ENSG00000015413	CLSLL	87	>ENSG00000123415	FSSQL	137	>ENSG00000173467	IQSEL	187	>ENSG00000143612	LESTL	237	>ENSG00000162599	PFSTL
38	>ENSG00000180305	CMSIL	88	>ENSG00000215251	FTSAL	138	>ENSG00000163344	IRSRL	188	>ENSG00000166833	LESTL	238	>ENSG00000149634	PGSCL
39	>ENSG00000182931	CMSIL	89	>ENSG00000138375	FTSPL	139	>ENSG00000182247	ISSNL	189	>ENSG00000197635	LFSLL	239	>ENSG00000225688	PGSGL
40	>ENSG00000144410	CMSLL	90	>ENSG00000173867	FVSDL	140	>ENSG00000107371	ISSRL	190	>ENSG00000109062	LFSNL	240	>ENSG00000187258	PGSSL
41	>ENSG00000126233	CNSEL	91	>ENSG00000070950	FYSKL	141	>ENSG00000133640	ISSYL	191	>ENSG00000204130	LFSPL	241	>ENSG00000094914	PHSHL
42	>ENSG00000068724	CRSHL	92	>ENSG00000003402	GCSTL	142	>ENSG00000119535	ITSVL	192	>ENSG00000156869	LGSCL	242	>ENSG00000203797	PKSNL
43	>ENSG00000157617	CSSAL	93	>ENSG00000167703	GDSCL	143	>ENSG00000119655	IVSHL	193	>ENSG00000227130	LGSFL	243	>ENSG00000130540	PLSAL
44	>ENSG00000065882	CSSSL	94	>ENSG00000183530	GDSSL	144	>ENSG00000156931	IVSIL	194	>ENSG00000149016	LHSLL	244	>ENSG00000116199	PLSHL
45	>ENSG00000215454	CVSLL	95	>ENSG00000072110	GESDL	145	>ENSG00000104112	IYSSL	195	>ENSG00000131650	LISAL	245	>ENSG00000158555	PLSPL
46	>ENSG00000237787	DASHL	96	>ENSG00000077522	GESDL	146	>ENSG00000124205	KASRL	196	>ENSG00000227103	LISKL	246	>ENSG00000110887	PPSHL
47	>ENSG00000154783	DASVL	97	>ENSG00000130402	GESDL	147	>ENSG00000110723	KESEL	197	>ENSG00000218940	LKSHL	247	>ENSG00000204568	PQSAL
48	>ENSG00000213096	DFSLL	98	>ENSG00000214694	GFSSL	148	>ENSG00000119865	KESFL	198	>ENSG00000087008	LKSKL	248	>ENSG00000156521	PRSKL
49	>ENSG00000135253	DGSRL	99	>ENSG00000136449	GGSCL	149	>ENSG00000121989	KESSL	199	>ENSG00000102539	LLSVL	249	>ENSG00000113790	PSSKL
50	>ENSG00000114115	DLSSL	100	>ENSG00000196960	GGSCL	150	>ENSG00000115902	KESVL	200	>ENSG00000213214	LLSVL	250	>ENSG00000172500	PSSSL

Additional file 4. continued.

human xxSxL

No.	gene ID	5aa	No.	gene ID	5aa	No.	gene ID	5aa
251	>ENSG00000143971	PTSFL	301	>ENSG00000172243	RVSHL	351	>ENSG00000188343	TVSLL
252	>ENSG00000172724	PVSSL	302	>ENSG00000184304	RVSIL	352	>ENSG00000155893	TWSAL
253	>ENSG00000115306	PWSGL	303	>ENSG00000197683	SCSGL	353	>ENSG00000205726	TWSLL
254	>ENSG00000122574	QASCL	304	>ENSG00000087206	SCSHL	354	>ENSG00000133136	VCSFL
255	>ENSG00000110203	QDSCL	305	>ENSG00000187479	SDSAL	355	>ENSG00000174021	VCSFL
256	>ENSG00000075624	QDSLL	306	>ENSG00000101473	SESKL	356	>ENSG00000183172	VCSPL
257	>ENSG00000166682	QDSLL	307	>ENSG00000198223	SESSL	357	>ENSG00000141469	VESPL
258	>ENSG00000137841	QESRL	308	>ENSG00000099251	SGSCL	358	>ENSG00000133250	VFSQL
259	>ENSG00000174500	QFSHL	309	>ENSG00000132196	SGSCL	359	>ENSG00000230055	VGSPL
260	>ENSG00000116882	QFSRL	310	>ENSG00000135838	SGSHL	360	>ENSG00000120253	VHSML
261	>ENSG00000146457	QGSVL	311	>ENSG00000181274	SGSLL	361	>ENSG00000138399	VKSCL
262	>ENSG00000186409	QKSEL	312	>ENSG00000198898	SGSLL	362	>ENSG00000204252	VLSAL
263	>ENSG00000175575	QLSDL	313	>ENSG00000024048	SGSPL	363	>ENSG00000180011	VNSKL
264	>ENSG00000106415	QLSVL	314	>ENSG00000144028	SGSRL	364	>ENSG00000226757	VPSDL
265	>ENSG00000104142	QLSWL	315	>ENSG00000145536	SKSNL	365	>ENSG00000176040	VPSLL
266	>ENSG00000088888	QPSAL	316	>ENSG00000108474	SLSFL	366	>ENSG00000167523	VRSLL
267	>ENSG00000085721	QPSPL	317	>ENSG00000101311	SLSHL	367	>ENSG00000163464	VSSNL
268	>ENSG00000197119	QPSSL	318	>ENSG00000115947	SLSWL	368	>ENSG00000163464	VSSNL
269	>ENSG00000160055	QQSTL	319	>ENSG00000133612	SPSLL	369	>ENSG00000197604	VSSSL
270	>ENSG00000146576	QSSGL	320	>ENSG00000100147	SQSEL	370	>ENSG00000181323	VTSLL
271	>ENSG00000162009	QTSKL	321	>ENSG00000146477	SRSHL	371	>ENSG00000136881	VTSQL
272	>ENSG00000196498	QTSPL	322	>ENSG00000188186	SSSNL	372	>ENSG00000165084	VYSGL
273	>ENSG00000116171	QTSWL	323	>ENSG00000168959	SSSSL	373	>ENSG00000196588	WDSCL
274	>ENSG00000168418	QVSGL	324	>ENSG00000152822	SSSTL	374	>ENSG00000105483	WISSL
275	>ENSG00000083844	QVSSL	325	>ENSG00000160310	STSAL	375	>ENSG00000179087	WISTL
276	>ENSG00000204524	QVSSL	326	>ENSG00000105792	STSGL	376	>ENSG00000160298	WLSSL
277	>ENSG00000163349	QYSYL	327	>ENSG00000186810	SYSGL	377	>ENSG00000162775	WNSKL
278	>ENSG00000087085	RCSDL	328	>ENSG00000198807	TASAL	378	>ENSG00000140987	WQSPL
279	>ENSG00000166128	RCSLL	329	>ENSG00000214405	TCSLL	379	>ENSG00000168306	WRSKL
280	>ENSG00000132300	RESFL	330	>ENSG00000169220	TDSAL	380	>ENSG00000101331	WSSWL
281	>ENSG00000134313	RESIL	331	>ENSG00000118515	TDSFL	381	>ENSG00000099246	YCSVL
282	>ENSG00000167634	RESKL	332	>ENSG00000104823	TFSKL	382	>ENSG00000171044	YESSL
283	>ENSG00000092140	RESLL	333	>ENSG00000160746	THSWL	383	>ENSG00000174672	YESSL
284	>ENSG00000123610	RFSHL	334	>ENSG00000180708	TISLL	384	>ENSG00000174236	YISIL
285	>ENSG00000135740	RGSRL	335	>ENSG00000102805	TLSGL	385	>ENSG00000077498	YQSHL
286	>ENSG00000105287	RISVL	336	>ENSG00000071553	TLSQL	386	>ENSG00000123447	YQSHL
287	>ENSG00000183473	RISYL	337	>ENSG00000120253	TNSML	387	>ENSG00000118245	YRSHL
288	>ENSG00000164938	RKSCL	338	>ENSG00000102384	TNSPL	388	>ENSG00000101624	YRSVL
289	>ENSG00000213965	RKSHL	339	>ENSG00000157326	TPSRL	389	>ENSG00000163291	YVSHL
290	>ENSG00000198721	RKSKL	340	>ENSG00000187630	TPSRL	390	>ENSG00000151067	YVSSL
291	>ENSG00000175485	RKSPL	341	>ENSG00000225766	TPSRL			
292	>ENSG00000186130	RQSLL	342	>ENSG00000149133	TSSFL			
293	>ENSG00000186642	RQSML	343	>ENSG00000143889	TSSHL			
294	>ENSG00000072840	RRSNL	344	>ENSG00000116679	TSSLL			
295	>ENSG00000148814	RRSRL	345	>ENSG00000196220	TSSRL			
296	>ENSG00000150403	RRSSL	346	>ENSG00000166105	TSSSL			
297	>ENSG00000095370	RSSEL	347	>ENSG00000096070	TSSYL			
298	>ENSG00000196600	RSSVL	348	>ENSG00000164082	TTSSL			
299	>ENSG00000184792	RTSHL	349	>ENSG00000198822	TTSSL			
300	>ENSG00000184408	RVSAL	350	>ENSG00000149488	TVSAL			

Additional file 4. continued.

human xxSxV

No.	gene ID	5aa	No.	gene ID	5aa	No.	gene ID	5aa	No.	gene ID	5aa	No.	gene ID	5aa
1	>ENSG00000135423	ACSPV	51	>ENSG00000165092	GASVV	101	>ENSG00000221926	LFSGV	151	>ENSG00000104881	QRSKV	201	>ENSG00000143653	SSSEV
2	>ENSG00000117394	ADSQV	52	>ENSG00000197183	GCSSV	102	>ENSG00000083223	LGSWV	152	>ENSG00000157992	QRSWV	202	>ENSG00000088387	SSSVV
3	>ENSG00000196923	AFSHV	53	>ENSG00000163947	GESNV	103	>ENSG00000147099	LHSLV	153	>ENSG00000109756	QVSAV	203	>ENSG00000156282	STSYV
4	>ENSG00000144847	AGSLV	54	>ENSG00000157514	GGSAV	104	>ENSG00000155744	LISQV	154	>ENSG00000158987	QVSAV	204	>ENSG00000115415	SVSEV
5	>ENSG00000149564	AGSLV	55	>ENSG00000116030	GHSTV	105	>ENSG00000106261	LKSCV	155	>ENSG00000070526	QVSEV	205	>ENSG00000174748	SVSRV
6	>ENSG00000187553	AGSLV	56	>ENSG00000155622	GKSQV	106	>ENSG00000056345	LKSLV	156	>ENSG00000196642	QVSPV	206	>ENSG00000171680	TASEV
7	>ENSG00000145736	APSGV	57	>ENSG00000185751	GKSQV	107	>ENSG00000066583	LLSKV	157	>ENSG00000135914	QVSYV	207	>ENSG00000124357	THSPV
8	>ENSG00000183474	APSGV	58	>ENSG00000153936	GLSAV	108	>ENSG00000184108	LNSHV	158	>ENSG00000178188	QYSFV	208	>ENSG00000188981	TKSSV
9	>ENSG00000226259	APSGV	59	>ENSG00000166948	GLSPV	109	>ENSG00000068831	LPSGV	159	>ENSG00000152154	RDSTV	209	>ENSG00000135409	TLSPV
10	>ENSG00000064300	ATSPV	60	>ENSG00000183161	GLSSV	110	>ENSG00000114770	LPSTV	160	>ENSG00000185033	RDSVV	210	>ENSG00000152683	TNSIV
11	>ENSG00000189431	CESLV	61	>ENSG00000174547	GRSSV	111	>ENSG00000178217	LPSVV	161	>ENSG00000197245	RESEV	211	>ENSG00000169208	TPSEV
12	>ENSG00000136842	CRSGV	62	>ENSG00000213996	GTSQV	112	>ENSG00000154229	LQSAV	162	>ENSG00000183873	RESIV	212	>ENSG00000174567	TSSMV
13	>ENSG00000176542	CSSAV	63	>ENSG00000144550	GTSVV	113	>ENSG00000114993	LQSPV	163	>ENSG00000105963	RESQV	213	>ENSG00000169894	TSSSV
14	>ENSG00000148057	CSSEV	64	>ENSG00000127507	HFSPV	114	>ENSG00000101187	LQSSV	164	>ENSG00000131263	RESVV	214	>ENSG00000116903	TTSVV
15	>ENSG00000111640	CSSWV	65	>ENSG00000140873	HLSKV	115	>ENSG00000213921	LQSSV	165	>ENSG00000005448	RFSSV	215	>ENSG00000090565	VASFV
16	>ENSG00000143469	DCSVV	66	>ENSG00000188869	ICSDV	116	>ENSG00000197992	LRSSV	166	>ENSG00000163870	RGSAV	216	>ENSG00000165449	VASNV
17	>ENSG00000144406	DESHV	67	>ENSG00000235000	IDSEV	117	>ENSG00000070182	LVSFV	167	>ENSG00000124523	RHSRV	217	>ENSG00000205277	VASTV
18	>ENSG00000095539	DESSV	68	>ENSG00000150086	IESDV	118	>ENSG00000175866	LVSTV	168	>ENSG00000131591	RISNV	218	>ENSG00000099889	VDSWV
19	>ENSG00000130921	DFSQV	69	>ENSG00000183454	IESDV	119	>ENSG00000144724	MESLV	169	>ENSG00000177807	RISNV	219	>ENSG00000170921	VESNV
20	>ENSG00000154639	DGSIV	70	>ENSG00000115183	IESNV	120	>ENSG00000156564	MESTV	170	>ENSG00000147246	RISSV	220	>ENSG00000063438	VFSIV
21	>ENSG00000147606	DHSEV	71	>ENSG00000153339	IISNV	121	>ENSG00000102763	MLSSV	171	>ENSG00000012171	RLSGV	221	>ENSG00000137204	VGSGV
22	>ENSG00000198121	DHSVV	72	>ENSG00000163362	IISQV	122	>ENSG00000091129	MNSFV	172	>ENSG00000131951	RNSPV	222	>ENSG00000083782	VGSLV
23	>ENSG00000111452	DLSAV	73	>ENSG00000213928	ILSLV	123	>ENSG00000182973	MRSGV	173	>ENSG00000160219	RQSKV	223	>ENSG00000180776	VKSGV
24	>ENSG00000122966	DQSSV	74	>ENSG00000119673	IPSKV	124	>ENSG00000157542	NESKV	174	>ENSG00000182489	RQSVV	224	>ENSG00000171885	VLSSV
25	>ENSG00000166024	DQSTV	75	>ENSG00000184227	IPSKV	125	>ENSG00000135973	NQSAV	175	>ENSG00000186654	RQSVV	225	>ENSG00000164438	VTSLV
26	>ENSG00000150893	DSSEV	76	>ENSG00000026103	IQSLV	126	>ENSG00000107165	NQSVV	176	>ENSG00000070087	RRSDV	226	>ENSG00000198815	WDSIV
27	>ENSG00000134874	DTSDV	77	>ENSG00000084754	IQSSV	127	>ENSG00000071127	NRSSV	177	>ENSG00000171303	RRSSV	227	>ENSG00000198948	YESHV
28	>ENSG00000017260	DVSCV	78	>ENSG00000175305	ISSHV	128	>ENSG00000196169	NSSWV	178	>ENSG00000168256	RSSGV	228	>ENSG00000185933	YFSKV
29	>ENSG00000081479	EDSEV	79	>ENSG00000166016	ITSRV	129	>ENSG00000138449	NTSVV	179	>ENSG00000183695	RSSLV			
30	>ENSG00000110841	EDSNV	80	>ENSG00000007314	KESLV	130	>ENSG00000144283	PDSWV	180	>ENSG00000169499	RTSDV			
31	>ENSG00000137491	EDSRV	81	>ENSG00000165029	KESYV	131	>ENSG00000169862	PDSWV	181	>ENSG00000008323	SASEV			
32	>ENSG00000188487	EESFV	82	>ENSG00000164068	KGSLV	132	>ENSG00000109943	PESIV	182	>ENSG00000043591	SESKV			
33	>ENSG00000155380	EESPV	83	>ENSG00000151136	KGSVV	133	>ENSG00000139517	PGSLV	183	>ENSG00000162728	SESKV			
34	>ENSG00000168758	EESSV	84	>ENSG00000162620	KHSEV	134	>ENSG00000204070	PKSNV	184	>ENSG00000134569	SESQV			
35	>ENSG00000173621	EESVV	85	>ENSG00000130702	KISWV	135	>ENSG00000164118	PLSCV	185	>ENSG00000106868	SFSAV			
36	>ENSG00000132139	EESWV	86	>ENSG00000175048	KLSSV	136	>ENSG00000228120	PLSGV	186	>ENSG00000078269	SGSSV			
37	>ENSG00000163449	ETSTV	87	>ENSG00000116001	KMSCV	137	>ENSG00000177283	PLSQV	187	>ENSG00000139998	SHSRV			
38	>ENSG00000114812	EVSLV	88	>ENSG00000174015	KPSRV	138	>ENSG00000163888	PPSGV	188	>ENSG00000085276	SISHV			
39	>ENSG00000133067	FASHV	89	>ENSG00000136522	KSSLV	139	>ENSG00000177098	PPSKV	189	>ENSG00000166436	SISQV			
40	>ENSG00000164690	FDSSV	90	>ENSG00000115107	KTSHV	140	>ENSG00000102780	PQSEV	190	>ENSG00000111339	SKSRV			
41	>ENSG00000184465	FGSSV	91	>ENSG00000102468	KVSCV	141	>ENSG00000198157	PQSIV	191	>ENSG00000165309	SKSYV			
42	>ENSG00000197535	FISRV	92	>ENSG00000169122	KVSHV	142	>ENSG00000164463	PTSKV	192	>ENSG00000163251	SLSHV			
43	>ENSG00000230301	FKSNV	93	>ENSG00000158517	LASAV	143	>ENSG00000107679	PVSDV	193	>ENSG00000040341	SNSAV			
44	>ENSG00000166912	FPSRV	94	>ENSG00000165178	LASAV	144	>ENSG00000135315	PVSRV	194	>ENSG00000146242	SNSDV			
45	>ENSG00000175077	FRSSV	95	>ENSG00000115297	LASVV	145	>ENSG00000177352	PYSAV	195	>ENSG00000132975	SPSDV			
46	>ENSG00000164850	FSSAV	96	>ENSG00000105464	LESEV	146	>ENSG00000162511	PYSEV	196	>ENSG00000181773	SPSDV			
47	>ENSG00000069188	FSSFV	97	>ENSG00000161509	LESEV	147	>ENSG00000162692	QKSKV	197	>ENSG00000146360	SPSEV			
48	>ENSG00000146555	FSSFV	98	>ENSG00000106278	LESLV	148	>ENSG00000186143	QKSSV	198	>ENSG00000178104	SPSEV			
49	>ENSG00000119125	FSSSV	99	>ENSG00000135423	LESMV	149	>ENSG00000186001	QLSAV	199	>ENSG00000175224	SPSTV			
50	>ENSG00000133657	FYSLV	100	>ENSG00000128011	LESTV	150	>ENSG00000157551	QQSNV	200	>ENSG00000120645	SRSLV			

Additional file 4. continued.

human xxTxI

No.	gene ID	5aa	No.	gene ID	5aa	No.	gene ID	5aa
1	>ENSG00000119685	AHTKI	51	>ENSG00000112077	LGTAI	101	>ENSG00000131981	SYTMI
2	>ENSG00000114544	AKTSI	52	>ENSG00000124772	LHTHI	102	>ENSG00000107816	TATEI
3	>ENSG00000187239	AKTYI	53	>ENSG00000156299	LNTEI	103	>ENSG00000106823	TGTEI
4	>ENSG00000141750	ALTEI	54	>ENSG00000090905	LQTFI	104	>ENSG00000166351	TKTNI
5	>ENSG00000115109	AMTEI	55	>ENSG00000131050	LQTLI	105	>ENSG00000183206	TKTNI
6	>ENSG00000233475	ATTGI	56	>ENSG00000139117	LQTQI	106	>ENSG00000237375	TKTNI
7	>ENSG00000137942	AVTYI	57	>ENSG00000180616	LQTSI	107	>ENSG00000230031	TKTSI
8	>ENSG00000187824	CKTVI	58	>ENSG00000016602	LSTTI	108	>ENSG00000233917	TKTSI
9	>ENSG00000106384	CLTFI	59	>ENSG00000112214	MDTDI	109	>ENSG00000134317	TLTEI
10	>ENSG00000212913	CPTRI	60	>ENSG00000151338	MRTVI	110	>ENSG00000173681	VATGI
11	>ENSG00000164509	CPTSI	61	>ENSG00000163162	MWTSI	111	>ENSG00000163825	VKTAI
12	>ENSG00000188343	CQTLI	62	>ENSG00000196648	NITII	112	>ENSG00000162747	VKTNI
13	>ENSG00000135622	DETSI	63	>ENSG00000197978	NITII	113	>ENSG00000172995	VPTAI
14	>ENSG00000055211	DKTCI	64	>ENSG00000205281	NITII	114	>ENSG00000157985	VPTII
15	>ENSG00000197121	DLTGI	65	>ENSG00000215749	NITII	115	>ENSG00000166924	WDTAI
16	>ENSG00000140416	DMTSI	66	>ENSG00000230135	NITII	116	>ENSG00000041515	WDTTI
17	>ENSG00000143549	DMTSI	67	>ENSG00000188910	NLTPI	117	>ENSG00000008277	WETSI
18	>ENSG00000186193	DSTFI	68	>ENSG00000007944	NLTVI	118	>ENSG00000115841	WSTKI
19	>ENSG00000172752	EATDI	69	>ENSG00000134291	NSTAI	119	>ENSG00000174950	YQTLI
20	>ENSG00000136869	EATSI	70	>ENSG00000214433	NTTII	120	>ENSG00000116254	YVTDI
21	>ENSG00000101745	ELTPI	71	>ENSG00000139826	NVTII	121	>ENSG00000157483	YVTKI
22	>ENSG00000088899	ESTEI	72	>ENSG00000133121	PETKI			
23	>ENSG00000206432	ESTGI	73	>ENSG00000088812	PGTCI			
24	>ENSG00000177683	EVTMI	74	>ENSG00000185670	PKTNI			
25	>ENSG00000186921	EVTMI	75	>ENSG00000134595	PLTHI			
26	>ENSG00000154269	FETTI	76	>ENSG00000182968	PLTHI			
27	>ENSG00000109193	FRTEI	77	>ENSG00000008282	PPTGI			
28	>ENSG00000173597	FRTEI	78	>ENSG00000213397	PRTRI			
29	>ENSG00000196228	FRTEI	79	>ENSG00000188523	PTTWI			
30	>ENSG00000181609	GKTSI	80	>ENSG00000128594	QETQI			
31	>ENSG00000228761	GPTQI	81	>ENSG00000131409	QETQI			
32	>ENSG00000196758	HATVI	82	>ENSG00000148948	QETQI			
33	>ENSG00000182771	HGTSI	83	>ENSG00000047849	QETSI			
34	>ENSG00000115661	HTTQI	84	>ENSG00000004866	QLTRI			
35	>ENSG00000061337	IATEI	85	>ENSG00000153802	QQTGI			
36	>ENSG00000034677	IQTEI	86	>ENSG00000171873	RETDI			
37	>ENSG00000165164	IVTYI	87	>ENSG00000118596	RETNI			
38	>ENSG00000017797	KETSI	88	>ENSG00000187123	RGTMI			
39	>ENSG00000107551	KFTRI	89	>ENSG00000152208	RGTSI			
40	>ENSG00000185658	KGTLI	90	>ENSG00000080815	RGTVI			
41	>ENSG00000213974	KITLI	91	>ENSG00000087077	RPTGI			
42	>ENSG00000167110	KITVI	92	>ENSG00000170935	RQTQI			
43	>ENSG00000220356	KITVI	93	>ENSG00000173786	SCTII			
44	>ENSG00000129083	KKTSI	94	>ENSG00000113580	SHTLI			
45	>ENSG00000182261	KNTYI	95	>ENSG00000088053	SITLI			
46	>ENSG00000212739	KQTFI	96	>ENSG00000211451	SITSI			
47	>ENSG00000173451	KSTFI	97	>ENSG00000087128	SKTGI			
48	>ENSG00000169297	LCTKI	98	>ENSG00000187054	SKTGI			
49	>ENSG00000136315	LDTII	99	>ENSG00000148848	SLTLI			
50	>ENSG00000169397	LDTTI	100	>ENSG00000164175	STTWI			

Additional file 4. continued.

human xxTxL

No.	gene ID	5aa	No.	gene ID	5aa	No.	gene ID	5aa	No.	gene ID	5aa	No.	gene ID	5aa
1	>ENSG00000011405	AATYL	51	>ENSG00000154124	EETSL	101	>ENSG00000127588	KCTIL	151	>ENSG00000130487	LQTSL	201	>ENSG00000117676	PSTTL
2	>ENSG00000174059	ADTEL	52	>ENSG00000180263	EGTIL	102	>ENSG00000005483	KETHL	152	>ENSG00000134516	LSTDL	202	>ENSG00000165801	PTTPL
3	>ENSG00000164199	ADTHL	53	>ENSG00000107736	EITEL	103	>ENSG00000111181	KETHL	153	>ENSG00000115109	LTTEL	203	>ENSG00000203908	PVTRL
4	>ENSG00000033867	AETSL	54	>ENSG00000091536	EITLL	104	>ENSG00000138381	KETKL	154	>ENSG00000213199	LVTQL	204	>ENSG00000169891	PVTVL
5	>ENSG00000064687	AETVL	55	>ENSG00000123739	EKTDL	105	>ENSG00000049192	KETLL	155	>ENSG00000072071	LVTSL	205	>ENSG00000085552	QATLL
6	>ENSG00000102100	AGTKL	56	>ENSG00000102858	ELTPL	106	>ENSG00000151458	KETPL	156	>ENSG00000117114	LVTSL	206	>ENSG00000160209	QATVL
7	>ENSG00000112062	AGTSL	57	>ENSG00000149782	ENTQL	107	>ENSG00000188130	KETPL	157	>ENSG00000150471	LVTSL	207	>ENSG00000148110	QCTEL
8	>ENSG00000120798	AITDL	58	>ENSG00000137707	ERTKL	108	>ENSG00000139155	KETQL	158	>ENSG00000178252	MCTRL	208	>ENSG00000001626	QDTRL
9	>ENSG00000042813	AKTCL	59	>ENSG00000107537	ERTNL	109	>ENSG00000117335	KFTSL	159	>ENSG00000181171	MDTAL	209	>ENSG00000105641	QETNL
10	>ENSG00000162775	ALTLL	60	>ENSG00000006740	ESTAL	110	>ENSG00000100083	KGTLL	160	>ENSG00000151849	MDTEL	210	>ENSG00000138074	QETSL
11	>ENSG00000099953	ANTFL	61	>ENSG00000140750	ESTAL	111	>ENSG00000078674	KITVL	161	>ENSG00000100448	METPL	211	>ENSG00000151917	QGTKL
12	>ENSG00000170419	APTVL	62	>ENSG00000168065	ESTSL	112	>ENSG00000101180	KKTCL	162	>ENSG00000108465	MGTSL	212	>ENSG00000124568	QHTRL
13	>ENSG00000080845	AQTRL	63	>ENSG00000236840	EVTCL	113	>ENSG00000189280	KKTIL	163	>ENSG00000163482	MLTSL	213	>ENSG00000198399	QKTLL
14	>ENSG00000116544	AQTRL	64	>ENSG00000138483	EVTRL	114	>ENSG00000163406	KKTKL	164	>ENSG00000172943	MMTLL	214	>ENSG00000146755	QPTKL
15	>ENSG00000170579	AQTRL	65	>ENSG00000176371	EVTSL	115	>ENSG00000105997	KLTHL	165	>ENSG00000165409	MQTVL	215	>ENSG00000064042	QPTTL
16	>ENSG00000198010	AQTRL	66	>ENSG00000147912	FCTIL	116	>ENSG00000120093	KLTHL	166	>ENSG00000135917	MSTKL	216	>ENSG00000181214	QRTLL
17	>ENSG00000215750	AQTRL	67	>ENSG00000168243	FCTIL	117	>ENSG00000128652	KLTHL	167	>ENSG00000105707	MVTQL	217	>ENSG00000133460	QSTEL
18	>ENSG00000227328	AQTRL	68	>ENSG00000040199	FDTAL	118	>ENSG00000183808	KLTLL	168	>ENSG00000131183	NATRL	218	>ENSG00000150275	QSTSL
19	>ENSG00000145248	AQTSL	69	>ENSG00000168036	FDTDL	119	>ENSG00000124564	KLTRL	169	>ENSG00000171444	NETSL	219	>ENSG00000111252	QYTPL
20	>ENSG00000145050	ARTDL	70	>ENSG00000182621	FDTPL	120	>ENSG00000204683	KMTFL	170	>ENSG00000139357	NGTRL	220	>ENSG00000131650	RATGL
21	>ENSG00000070601	ASTAL	71	>ENSG00000125257	FETAL	121	>ENSG00000124440	KPTVL	171	>ENSG00000146910	NHTEL	221	>ENSG00000148444	RATQL
22	>ENSG00000145012	ASTDL	72	>ENSG00000119778	FETFL	122	>ENSG00000111859	KQTSL	172	>ENSG00000188763	NPTHL	222	>ENSG00000119139	RDTEL
23	>ENSG00000147099	AVTSL	73	>ENSG00000018408	FLTWL	123	>ENSG00000197849	KRTFL	173	>ENSG00000140093	NPTLL	223	>ENSG00000144290	RETCL
24	>ENSG00000204561	CGTPL	74	>ENSG00000137693	FLTWL	124	>ENSG00000135838	KRTSL	174	>ENSG00000150403	NRTAL	224	>ENSG00000130037	RETDL
25	>ENSG00000157388	CITTL	75	>ENSG00000126216	FRTRL	125	>ENSG00000108599	KSTKL	175	>ENSG00000005469	NSTHL	225	>ENSG00000160767	RETGL
26	>ENSG00000100380	CTTTL	76	>ENSG00000164961	FRTVL	126	>ENSG00000171462	KTTAL	176	>ENSG00000109572	NSTTL	226	>ENSG00000152402	RETSL
27	>ENSG00000226588	DATAL	77	>ENSG00000174595	FTTCL	127	>ENSG00000164050	KVTDL	177	>ENSG00000228562	NVTFL	227	>ENSG00000135063	RETVL
28	>ENSG00000165240	DDTAL	78	>ENSG00000105668	FYTML	128	>ENSG00000196576	KVTDL	178	>ENSG00000182010	NVTIL	228	>ENSG00000221838	RFTAL
29	>ENSG00000159346	DDTLL	79	>ENSG00000155511	GATGL	129	>ENSG00000198753	KVTDL	179	>ENSG00000105289	PATDL	229	>ENSG00000172831	RHTEL
30	>ENSG00000102606	DETNL	80	>ENSG00000130307	GDTFL	130	>ENSG00000070961	LETSL	180	>ENSG00000072609	PATSL	230	>ENSG00000121644	RHTKL
31	>ENSG00000107147	DETQL	81	>ENSG00000169860	GDTSL	131	>ENSG00000157087	LETSL	181	>ENSG00000148942	PCTSL	231	>ENSG00000204361	RHTWL
32	>ENSG00000160460	DGTCL	82	>ENSG00000181619	GDTSL	132	>ENSG00000206199	LETVL	182	>ENSG00000116039	PDTAL	232	>ENSG00000139874	RITTL
33	>ENSG00000076641	DITRL	83	>ENSG00000182667	GETVL	133	>ENSG00000103111	LFTGL	183	>ENSG00000130052	PETKL	233	>ENSG00000147003	RLTPL
34	>ENSG00000198353	DITRL	84	>ENSG00000179454	GLTAL	134	>ENSG00000132466	LITHL	184	>ENSG00000104973	PETPL	234	>ENSG00000173486	RRTEL
35	>ENSG00000198467	DITSL	85	>ENSG00000163541	GLTCL	135	>ENSG00000106541	LKTEL	185	>ENSG00000072201	PGTFL	235	>ENSG00000160584	RRTTL
36	>ENSG00000077274	DLTEL	86	>ENSG00000163472	GLTHL	136	>ENSG00000116005	LKTEL	186	>ENSG00000214733	PGTGL	236	>ENSG00000073756	RSTEL
37	>ENSG00000182224	DLTEL	87	>ENSG00000182704	GPTIL	137	>ENSG00000163389	LKTEL	187	>ENSG00000189233	PGTSL	237	>ENSG00000104889	SATSL
38	>ENSG00000127191	DLTGL	88	>ENSG00000054690	GPTLL	138	>ENSG00000084453	LKTKL	188	>ENSG00000166783	PITKL	238	>ENSG00000156313	SCTIL
39	>ENSG00000105696	DLTKL	89	>ENSG00000152527	GPTLL	139	>ENSG00000159086	LKTKL	189	>ENSG00000185267	PKTEL	239	>ENSG00000006534	SCTLL
40	>ENSG00000182534	DLTSL	90	>ENSG00000197070	GPTVL	140	>ENSG00000095752	LKTRL	190	>ENSG00000129194	PLTHL	240	>ENSG00000132746	SCTLL
41	>ENSG00000170166	DLTTL	91	>ENSG00000089177	HLTRL	141	>ENSG00000137878	LLTKL	191	>ENSG00000150275	PMTKL	241	>ENSG00000182271	SETAL
42	>ENSG00000074276	DTTDL	92	>ENSG00000224821	HPTQL	142	>ENSG00000157017	LLTSL	192	>ENSG00000081059	PMTVL	242	>ENSG00000109066	SETKL
43	>ENSG00000171357	DVTFL	93	>ENSG00000143476	HSTEL	143	>ENSG00000176057	LLTSL	193	>ENSG00000133424	PNTCL	243	>ENSG00000073910	SGTSL
44	>ENSG00000157765	ECTAL	94	>ENSG00000213402	HVTAL	144	>ENSG00000221917	LLTSL	194	>ENSG00000079277	PPTAL	244	>ENSG00000119685	SHTAL
45	>ENSG00000182040	EDTEL	95	>ENSG00000142279	HVTEL	145	>ENSG00000223839	LLTSL	195	>ENSG00000161981	PQTAL	245	>ENSG00000183337	SKTDL
46	>ENSG00000114631	EDTHL	96	>ENSG00000086102	HWTFL	146	>ENSG00000112081	LLTTL	196	>ENSG00000163106	PQTKL	246	>ENSG00000185873	SKTGL
47	>ENSG00000128567	EDTHL	97	>ENSG00000184895	HWTKL	147	>ENSG00000095203	LMTAL	197	>ENSG00000095303	PSTEL	247	>ENSG00000168026	SLTHL
48	>ENSG00000099992	EDTYL	98	>ENSG00000186451	IHTHL	148	>ENSG00000196843	LNTKL	198	>ENSG00000167700	PSTFL	248	>ENSG00000064932	SLTRL
49	>ENSG00000205359	EETDL	99	>ENSG00000163092	IITSL	149	>ENSG00000129493	LPTKL	199	>ENSG00000150347	PSTKL	249	>ENSG00000162384	SPTSL
50	>ENSG00000162687	EETQL	100	>ENSG00000140274	ITTNL	150	>ENSG00000198055	LPTRL	200	>ENSG00000168795	PSTLL	250	>ENSG00000157873	SQTDL

Additional file 4. continued.

human xxTxL

No.	gene ID	5aa	No.	gene ID	5aa
251	>ENSG00000181090	SQTIL	301	>ENSG00000206579	YETTL
252	>ENSG00000074842	SRTEL	302	>ENSG00000140297	YGTEL
253	>ENSG00000170871	SRTHL	303	>ENSG00000166013	YHTGL
254	>ENSG00000109743	SRTQL	304	>ENSG00000225581	YHTGL
255	>ENSG00000149932	SRTRL	305	>ENSG00000132950	YKTSL
256	>ENSG00000205636	SSTCL	306	>ENSG00000158552	YLTLL
257	>ENSG00000187961	SSTSL	307	>ENSG00000150275	YNTAL
258	>ENSG00000170956	STTCL	308	>ENSG00000152332	YQTLL
259	>ENSG00000055950	STTWL	309	>ENSG00000091136	YSTCL
260	>ENSG00000205015	SVTGL	310	>ENSG00000100867	YSTRL
261	>ENSG00000164099	SVTKL	311	>ENSG00000121057	YYTSL
262	>ENSG00000124207	SVTLL			
263	>ENSG00000225697	SVTRL			
264	>ENSG00000136286	SWTSL			
265	>ENSG00000152056	SWTSL			
266	>ENSG00000146828	TCTDL			
267	>ENSG00000089127	TCTIL			
268	>ENSG00000042781	TDTHL			
269	>ENSG00000122679	TDTLL			
270	>ENSG00000080493	TETTL			
271	>ENSG00000146039	TFTHL			
272	>ENSG00000174502	TLTAL			
273	>ENSG00000112337	TLTRL			
274	>ENSG00000140937	TLTSL			
275	>ENSG00000177189	TSTAL			
276	>ENSG00000072133	TSTGL			
277	>ENSG00000071242	TSTRL			
278	>ENSG00000180871	TSTTL			
279	>ENSG00000126705	TVTSL			
280	>ENSG00000092203	TWTTL			
281	>ENSG00000175311	VDTSL			
282	>ENSG00000067842	VETSL			
283	>ENSG00000178233	VETSL			
284	>ENSG00000142910	VITEL			
285	>ENSG00000145882	VKTEL			
286	>ENSG00000126947	VLTKL			
287	>ENSG00000099977	VMTFL			
288	>ENSG00000177428	VPTWL			
289	>ENSG00000072315	VTTRL			
290	>ENSG00000133107	VTTRL			
291	>ENSG00000137501	VTTRL			
292	>ENSG00000197415	VTTYL			
293	>ENSG00000197415	VTTYL			
294	>ENSG00000197415	VTTYL			
295	>ENSG00000115594	WATAL			
296	>ENSG00000188991	WETAL			
297	>ENSG00000162889	WLTRL			
298	>ENSG00000117859	WTTML			
299	>ENSG00000081913	YDTPL			
300	>ENSG00000162813	YETDL			

Additional file 4. continued.

human xxTxV

No.	gene ID	5aa	No.	gene ID	5aa	No.	gene ID	5aa	No.	gene ID	5aa	No.	gene ID	5aa
1	>ENSG00000186806	AATQV	51	>ENSG00000149970	IETHV	101	>ENSG00000151079	MLTEV	151	>ENSG00000139266	RVTTV	201	>ENSG00000164488	VMTMV
2	>ENSG00000212859	ASTWV	52	>ENSG00000187068	IETTV	102	>ENSG00000149571	MQTHV	152	>ENSG00000144583	RVTTV	202	>ENSG00000197380	VMTTV
3	>ENSG00000215437	AVTDV	53	>ENSG00000177272	IFTDV	103	>ENSG00000183853	MQTHV	153	>ENSG00000162738	SETSV	203	>ENSG00000173933	VPTGV
4	>ENSG00000174807	CRTSV	54	>ENSG00000153896	IHTRV	104	>ENSG00000166573	NCTHV	154	>ENSG00000173218	SETSV	204	>ENSG00000087053	VQTVV
5	>ENSG00000138759	DGTEV	55	>ENSG00000143340	ISTDV	105	>ENSG00000156395	NCTSV	155	>ENSG00000020181	SETTV	205	>ENSG00000176884	VSTVV
6	>ENSG00000138271	DYTDV	56	>ENSG00000196990	ISTDV	106	>ENSG00000197177	NETTV	156	>ENSG00000163235	SETVV	206	>ENSG00000137269	VTTSV
7	>ENSG00000117983	EATAV	57	>ENSG00000215611	ISTDV	107	>ENSG00000151893	NFTAV	157	>ENSG00000174804	SETVV	207	>ENSG00000100221	WRTDV
8	>ENSG00000185149	EATNV	58	>ENSG00000144711	ISTIV	108	>ENSG00000149300	NGTLV	158	>ENSG00000185800	SGTVV	208	>ENSG00000075188	WVTEV
9	>ENSG00000099785	EETPV	59	>ENSG00000124313	ISTVV	109	>ENSG00000163331	NLTGV	159	>ENSG00000167646	SKTGV	209	>ENSG00000101321	YETTV
10	>ENSG00000168269	EGTEV	60	>ENSG00000179796	ISTVV	110	>ENSG00000171860	NSTTV	160	>ENSG00000111432	SPTCV	210	>ENSG00000067208	YSTTV
11	>ENSG00000186766	EGTEV	61	>ENSG00000184650	KDTHV	111	>ENSG00000166342	NTTRV	161	>ENSG00000122786	SPTKV			
12	>ENSG00000135519	EGTGV	62	>ENSG00000173261	KDTLV	112	>ENSG00000171940	NTTVV	162	>ENSG00000146938	STTRV			
13	>ENSG00000125844	EGTSV	63	>ENSG00000173848	KETLV	113	>ENSG00000183067	NTTVV	163	>ENSG00000165246	STTRV			
14	>ENSG00000137478	EITLV	64	>ENSG00000152207	KETRV	114	>ENSG00000101333	PATVV	164	>ENSG00000169760	STTRV			
15	>ENSG00000100012	ELTPV	65	>ENSG00000169933	KETTV	115	>ENSG00000047648	PETLV	165	>ENSG00000169992	STTRV			
16	>ENSG00000135632	EMTDV	66	>ENSG00000173926	KETVV	116	>ENSG00000141526	PETSV	166	>ENSG00000196338	STTRV			
17	>ENSG00000149418	ENTGV	67	>ENSG00000171204	KFTNV	117	>ENSG00000167723	PETSV	167	>ENSG00000081019	STTSV			
18	>ENSG00000162510	ENTVV	68	>ENSG00000173214	KGTNV	118	>ENSG00000114541	PGTLV	168	>ENSG00000173320	SVTSV			
19	>ENSG00000189337	EVTNV	69	>ENSG00000074071	KGTPV	119	>ENSG00000147144	PGTLV	169	>ENSG00000067840	SVTTV			
20	>ENSG00000072135	EWTRV	70	>ENSG00000131558	KITTV	120	>ENSG00000112077	PKTRV	170	>ENSG00000121440	SVTTV			
21	>ENSG00000139971	FATGV	71	>ENSG00000196843	KKTPV	121	>ENSG00000150637	PKTRV	171	>ENSG00000165966	SVTTV			
22	>ENSG00000100360	FETSV	72	>ENSG00000175697	KPTLV	122	>ENSG00000166847	PLTQV	172	>ENSG00000154358	SYTAV			
23	>ENSG00000187650	FGTTV	73	>ENSG00000119878	KQTSV	123	>ENSG00000183150	PNTFV	173	>ENSG00000171587	SYTLV			
24	>ENSG00000121753	FQTEV	74	>ENSG00000223354	KSTPV	124	>ENSG00000206561	PPTFV	174	>ENSG00000177103	SYTLV			
25	>ENSG00000135298	FQTEV	75	>ENSG00000043093	KSTTV	125	>ENSG00000165626	PPTVV	175	>ENSG00000189013	TDTSV			
26	>ENSG00000159842	FSTDV	76	>ENSG00000142408	KTTPV	126	>ENSG00000179023	PQTRV	176	>ENSG00000215764	TDTSV			
27	>ENSG00000186716	FSTEV	77	>ENSG00000116771	KVTTV	127	>ENSG00000011422	QATHV	177	>ENSG00000169435	TETTV			
28	>ENSG00000160145	FSTYV	78	>ENSG00000198931	LATGV	128	>ENSG00000156875	QDTNV	178	>ENSG00000186814	THTRV			
29	>ENSG00000125046	GCTIV	79	>ENSG00000148541	LCTDV	129	>ENSG00000149091	QETAV	179	>ENSG00000101596	TKTDV			
30	>ENSG00000021574	GDTTV	80	>ENSG00000157680	LETAV	130	>ENSG00000214694	QGTAV	180	>ENSG00000087470	TPTSV			
31	>ENSG00000155760	GETAV	81	>ENSG00000168078	LETDV	131	>ENSG00000107518	QGTCV	181	>ENSG00000126934	TRTAV			
32	>ENSG00000065243	GETSV	82	>ENSG00000114790	LETNV	132	>ENSG00000183128	QHTDV	182	>ENSG00000059804	TTTNV			
33	>ENSG00000157240	GETTV	83	>ENSG00000162383	LETNV	133	>ENSG00000083857	QHTEV	183	>ENSG00000173262	TTTNV			
34	>ENSG00000180340	GETTV	84	>ENSG00000058668	LETSV	134	>ENSG00000047617	QHTNV	184	>ENSG00000184838	TTTTV			
35	>ENSG00000232013	GFTMV	85	>ENSG00000125871	LFTPV	135	>ENSG00000171862	QITKV	185	>ENSG00000118518	TVTEV			
36	>ENSG00000140153	GGTVV	86	>ENSG00000205436	LITCV	136	>ENSG00000165186	QITTV	186	>ENSG00000128045	TVTSV			
37	>ENSG00000154639	GITVV	87	>ENSG00000111262	LLTDV	137	>ENSG00000160883	QLTRV	187	>ENSG00000132972	TVTTV			
38	>ENSG00000175898	GNTVV	88	>ENSG00000161217	LLTQV	138	>ENSG00000197283	QQTRV	188	>ENSG00000082196	TWTKV			
39	>ENSG00000228727	GPTRV	89	>ENSG00000165617	LMTTV	139	>ENSG00000120088	QSTAV	189	>ENSG00000080298	TYTAV			
40	>ENSG00000159788	HATFV	90	>ENSG00000070413	LNTVV	140	>ENSG00000142621	RETNV	190	>ENSG00000121904	VCTAV			
41	>ENSG00000184792	HATVV	91	>ENSG00000181790	LQTEV	141	>ENSG00000054523	RETTV	191	>ENSG00000170482	VCTKV			
42	>ENSG00000188133	HETIV	92	>ENSG00000126259	LQTHV	142	>ENSG00000117614	RGTAV	192	>ENSG00000164796	VCTMV			
43	>ENSG00000152990	HETTV	93	>ENSG00000183077	LRTPV	143	>ENSG00000166508	RITFV	193	>ENSG00000183117	VCTVV			
44	>ENSG00000165323	HQTQV	94	>ENSG00000158169	LRTQV	144	>ENSG00000178568	RNTVV	194	>ENSG00000113946	VDTRV			
45	>ENSG00000112218	HRTVV	95	>ENSG00000169550	LRTSV	145	>ENSG00000185101	RSTDV	195	>ENSG00000130762	VETDV			
46	>ENSG00000121797	HSTEV	96	>ENSG00000204913	LSTVV	146	>ENSG00000131188	RTTAV	196	>ENSG00000182255	VETDV			
47	>ENSG00000178729	HVTTV	97	>ENSG00000104848	LVTEV	147	>ENSG00000006116	RTTPV	197	>ENSG00000130368	VETVV			
48	>ENSG00000128917	IATEV	98	>ENSG00000134982	LVTSV	148	>ENSG00000075461	RTTPV	198	>ENSG00000143507	VETVV			
49	>ENSG00000198719	IATEV	99	>ENSG00000119820	LYTGV	149	>ENSG00000166862	RTTPV	199	>ENSG00000065427	VGTSV			
50	>ENSG00000187862	ICTIV	100	>ENSG00000177301	MLTDV	150	>ENSG00000183654	RVTSV	200	>ENSG00000063601	VHTSV			

Additional file 4. continued.

human xxVxI

No.	gene ID	5aa	No.	gene ID	5aa
1	>ENSG00000150961	AFVVI	51	>ENSG00000106415	NYVII
2	>ENSG00000081181	ARVRI	52	>ENSG00000155307	PDVTI
3	>ENSG00000169682	ASVLI	53	>ENSG00000206187	PHVFI
4	>ENSG00000155886	CPVSI	54	>ENSG00000204711	PIVPI
5	>ENSG00000143515	CTVNI	55	>ENSG00000109956	PSVEI
6	>ENSG00000183632	CVVHI	56	>ENSG00000139330	QAVII
7	>ENSG00000205457	CVVHI	57	>ENSG00000107779	QDVKI
8	>ENSG00000197410	DEVQI	58	>ENSG00000171931	QNVFI
9	>ENSG00000160294	DMVDI	59	>ENSG00000181464	QNVFI
10	>ENSG00000135503	EDVKI	60	>ENSG00000107282	QPVYI
11	>ENSG00000120251	ESVKI	61	>ENSG00000161973	QRVRI
12	>ENSG00000125675	ESVKI	62	>ENSG00000188783	QSVVI
13	>ENSG00000072444	EVVTI	63	>ENSG00000014138	QVVRI
14	>ENSG00000188611	EVVTI	64	>ENSG00000110092	RDVDI
15	>ENSG00000204147	EVVTI	65	>ENSG00000165071	RFVKI
16	>ENSG00000104413	EWVCI	66	>ENSG00000008710	RGVTI
17	>ENSG00000061492	FGVYI	67	>ENSG00000181785	RIVNI
18	>ENSG00000188107	FIVRI	68	>ENSG00000169519	RSVGI
19	>ENSG00000134758	FQVNI	69	>ENSG00000145103	RSVVI
20	>ENSG00000075884	FTVVI	70	>ENSG00000171720	SDVEI
21	>ENSG00000198223	FYVFI	71	>ENSG00000236127	SLVFI
22	>ENSG00000185842	GAVYI	72	>ENSG00000114805	SLVQI
23	>ENSG00000196104	HDVYI	73	>ENSG00000138095	SVVLI
24	>ENSG00000138443	HGVKI	74	>ENSG00000171747	SYVQI
25	>ENSG00000181007	HNVKI	75	>ENSG00000120942	TGVLI
26	>ENSG00000048052	IKVII	76	>ENSG00000075073	THVEI
27	>ENSG00000111261	IYVDI	77	>ENSG00000160505	TRVEI
28	>ENSG00000170919	KGVMI	78	>ENSG00000182963	TSVWI
29	>ENSG00000163749	KKVMI	79	>ENSG00000198835	TTVWI
30	>ENSG00000135205	KPVEI	80	>ENSG00000197894	TVVKI
31	>ENSG00000139180	KTVNI	81	>ENSG00000139287	VAVDI
32	>ENSG00000100379	LGVPI	82	>ENSG00000138780	VGVPI
33	>ENSG00000176153	LKVAI	83	>ENSG00000124743	VPVSI
34	>ENSG00000174496	LNVKI	84	>ENSG00000119973	VSVVI
35	>ENSG00000147255	LPVPI	85	>ENSG00000138356	WNVPI
36	>ENSG00000184785	LQVYI	86	>ENSG00000163006	YAVNI
37	>ENSG00000166025	MEVLI	87	>ENSG00000063015	YEVSI
38	>ENSG00000224689	MNVTI	88	>ENSG00000100095	YEVSI
39	>ENSG00000171469	MSVTI	89	>ENSG00000174938	YEVSI
40	>ENSG00000204084	NAVCI			
41	>ENSG00000122591	NLVCI			
42	>ENSG00000105948	NRVSI			
43	>ENSG00000012779	NSVAI			
44	>ENSG00000161905	NSVAI			
45	>ENSG00000169570	NSVKI			
46	>ENSG00000179148	NSVSI			
47	>ENSG00000179593	NSVSI			
48	>ENSG00000108839	NSVTI			
49	>ENSG00000116525	NTVRI			
50	>ENSG00000163659	NTVSI			

Additional file 4. continued.

human xxVxV

No.	gene ID	5aa	No.	gene ID	5aa	No.	gene ID	5aa
1	>ENSG00000136824	AHVEV	51	>ENSG00000141736	LDVPV	101	>ENSG00000108018	TYVNV
2	>ENSG00000169727	AHVQV	52	>ENSG00000131116	LEVDV	102	>ENSG00000111364	VCVCV
3	>ENSG00000090372	AKVFV	53	>ENSG00000231036	LFVTV	103	>ENSG00000160117	VCVCV
4	>ENSG00000115808	AKVFV	54	>ENSG00000173535	LIVFV	104	>ENSG00000179820	VFVKV
5	>ENSG00000196792	AKVFV	55	>ENSG00000083223	LKVPV	105	>ENSG00000181250	VFVKV
6	>ENSG00000204967	AKVSV	56	>ENSG00000189266	LKVQV	106	>ENSG00000166851	VGVAV
7	>ENSG00000222011	ASVEV	57	>ENSG00000215700	LKVQV	107	>ENSG00000182504	VGVTV
8	>ENSG00000183018	ASVKV	58	>ENSG00000031823	LLVRV	108	>ENSG00000099389	VKVFV
9	>ENSG00000203697	CCVLV	59	>ENSG00000069535	LLVRV	109	>ENSG00000006210	VLVPV
10	>ENSG00000168038	CLVYV	60	>ENSG00000182580	LPVQV	110	>ENSG00000164651	VRVVV
11	>ENSG00000214706	CPVKV	61	>ENSG00000177627	LYVVV	111	>ENSG00000137204	VSVYV
12	>ENSG00000074621	CPVSV	62	>ENSG00000110768	MAVTV	112	>ENSG00000138463	VVVSV
13	>ENSG00000135451	CQVRV	63	>ENSG00000236023	MEVDV	113	>ENSG00000079385	WDVLV
14	>ENSG00000164989	CQVRV	64	>ENSG00000221823	MVVDV	114	>ENSG00000183185	WGVYV
15	>ENSG00000128191	CTVDV	65	>ENSG00000187902	NEVTV	115	>ENSG00000158125	WSVRV
16	>ENSG00000133624	DDVAV	66	>ENSG00000109163	NLVLV	116	>ENSG00000164953	WSVVV
17	>ENSG00000128283	DEVKV	67	>ENSG00000178965	NNVQV	117	>ENSG00000204420	YAVVV
18	>ENSG00000131023	DLVYV	68	>ENSG00000172466	NVVKV	118	>ENSG00000105509	YRVQV
19	>ENSG00000163075	DRVGV	69	>ENSG00000049247	NYVWV			
20	>ENSG00000055609	EEVRV	70	>ENSG00000180730	PAVTV			
21	>ENSG00000153094	EEVVV	71	>ENSG00000132703	PLVWV			
22	>ENSG00000177030	EKVTV	72	>ENSG00000188760	PPVRV			
23	>ENSG00000144655	EPVPV	73	>ENSG00000066735	QEVDV			
24	>ENSG00000178662	ETVPV	74	>ENSG00000082293	QEVDV			
25	>ENSG00000145416	EVVSV	75	>ENSG00000211584	QEVKV			
26	>ENSG00000141582	EYVTV	76	>ENSG00000183317	QGVQV			
27	>ENSG00000147036	FSVFV	77	>ENSG00000156313	QIVSV			
28	>ENSG00000172578	GAVSV	78	>ENSG00000136240	QKVSV			
29	>ENSG00000196923	GGVCV	79	>ENSG00000150457	QPVYV			
30	>ENSG00000167874	GKVWV	80	>ENSG00000133216	QSVEV			
31	>ENSG00000145506	GLVAV	81	>ENSG00000167720	QSVSV			
32	>ENSG00000044524	GPVPV	82	>ENSG00000167967	QTVIV			
33	>ENSG00000205106	GQVSV	83	>ENSG00000077713	QWVTV			
34	>ENSG00000106123	GSVEV	84	>ENSG00000130202	RAVYV			
35	>ENSG00000030304	GTVSV	85	>ENSG00000183317	RGVGV			
36	>ENSG00000157540	HDVPV	86	>ENSG00000172367	RGVPV			
37	>ENSG00000075891	HIVPV	87	>ENSG00000116106	RMVPV			
38	>ENSG00000063015	HRVSV	88	>ENSG00000164270	RPVPV			
39	>ENSG00000229361	IEVDV	89	>ENSG00000163898	SAVSV			
40	>ENSG00000234376	IEVDV	90	>ENSG00000100336	SCVCV			
41	>ENSG00000131142	IIVQV	91	>ENSG00000118762	SNVHV			
42	>ENSG00000009765	IMVTV	92	>ENSG00000157873	SPVIV			
43	>ENSG00000213639	KAVLV	93	>ENSG00000196422	SVVKV			
44	>ENSG00000105401	KDVSV	94	>ENSG00000100364	SVVQV			
45	>ENSG00000170456	KGVDV	95	>ENSG00000197852	TAVWV			
46	>ENSG00000182450	KGVPV	96	>ENSG00000188803	TEVTV			
47	>ENSG00000181481	KQVKV	97	>ENSG00000237515	TEVTV			
48	>ENSG00000122257	KSVTV	98	>ENSG00000132589	TGVQV			
49	>ENSG00000102531	KSVYV	99	>ENSG00000188316	TLVKV			
50	>ENSG00000173064	KYVAV	100	>ENSG00000164038	TSVQV			

Additional file 4. continued.

mouse xxSxI

No.	gene ID	5aa	No.	gene ID	5aa	No.	gene ID	5aa
1	>ENSMUSG00000074264	AESKI	51	>ENSMUSG00000019803	KSSDI	101	>ENSMUSG00000006498	SKSTI
2	>ENSMUSG00000074787	AGSEI	52	>ENSMUSG00000073174	KSSTI	102	>ENSMUSG00000028134	SKSTI
3	>ENSMUSG00000028618	AHSEI	53	>ENSMUSG00000002308	KTSLI	103	>ENSMUSG00000028382	SKSTI
4	>ENSMUSG00000061979	AKSFI	54	>ENSMUSG00000037513	KTSTI	104	>ENSMUSG00000040154	SKSTI
5	>ENSMUSG00000027879	ALSGI	55	>ENSMUSG00000038982	LHSRI	105	>ENSMUSG00000027618	SSSGI
6	>ENSMUSG00000055013	APSVI	56	>ENSMUSG00000073455	LISQI	106	>ENSMUSG00000067650	SSSHI
7	>ENSMUSG00000034416	ASSRI	57	>ENSMUSG00000079709	LISQI	107	>ENSMUSG00000073068	SSSHI
8	>ENSMUSG00000027261	AVSKI	58	>ENSMUSG00000004631	LLSGI	108	>ENSMUSG00000078123	SSSHI
9	>ENSMUSG00000066936	AVSLI	59	>ENSMUSG00000074393	LMSEI	109	>ENSMUSG00000074414	SSSQI
10	>ENSMUSG00000031555	CTSFI	60	>ENSMUSG00000078803	LMSEI	110	>ENSMUSG00000031785	SSSRI
11	>ENSMUSG00000055874	DGSDI	61	>ENSMUSG00000078804	LMSEI	111	>ENSMUSG00000029236	STSFI
12	>ENSMUSG00000050505	DISNI	62	>ENSMUSG00000045608	LQSTI	112	>ENSMUSG00000018068	SVSGI
13	>ENSMUSG00000042918	DLSRI	63	>ENSMUSG00000001334	LRSKI	113	>ENSMUSG00000028995	SVSKI
14	>ENSMUSG00000046337	DLSRI	64	>ENSMUSG00000028538	LSSGI	114	>ENSMUSG00000074883	TASWI
15	>ENSMUSG00000049928	EESEI	65	>ENSMUSG00000044639	MESHI	115	>ENSMUSG00000021639	TPSGI
16	>ENSMUSG00000022724	EFSDI	66	>ENSMUSG00000079145	MLSSI	116	>ENSMUSG00000056023	VPSFI
17	>ENSMUSG00000038517	ENSKI	67	>ENSMUSG00000070531	MSSWI	117	>ENSMUSG00000042156	VRSEI
18	>ENSMUSG00000043789	ENSTI	68	>ENSMUSG00000074595	MSSWI	118	>ENSMUSG00000022040	VTSKI
19	>ENSMUSG00000035413	EQSAI	69	>ENSMUSG00000033446	NESAI	119	>ENSMUSG00000030428	WQSSI
20	>ENSMUSG00000001053	ESSKI	70	>ENSMUSG00000053164	NGSHI	120	>ENSMUSG00000022425	YESEI
21	>ENSMUSG00000033219	ESSSI	71	>ENSMUSG00000019039	PLSHI	121	>ENSMUSG00000060636	YPSRI
22	>ENSMUSG00000069720	ESSSI	72	>ENSMUSG00000027925	PPSSI	122	>ENSMUSG00000062279	YPSRI
23	>ENSMUSG00000070933	ESSSI	73	>ENSMUSG00000036537	PVSII	123	>ENSMUSG00000067558	YPSRI
24	>ENSMUSG00000011171	ETSVI	74	>ENSMUSG00000028542	QDSRI	124	>ENSMUSG00000067575	YPSRI
25	>ENSMUSG00000004609	EYSEI	75	>ENSMUSG00000043929	QDSSI	125	>ENSMUSG00000068324	YPSRI
26	>ENSMUSG00000046080	FGSCI	76	>ENSMUSG00000040734	QRSKI	126	>ENSMUSG00000068674	YPSRI
27	>ENSMUSG00000057174	FLSII	77	>ENSMUSG00000030965	QTSQI	127	>ENSMUSG00000070519	YPSRI
28	>ENSMUSG00000029273	FRSEI	78	>ENSMUSG00000042757	QVSEI			
29	>ENSMUSG00000046078	FSSDI	79	>ENSMUSG00000026228	QVSYI			
30	>ENSMUSG00000020064	GFSLI	80	>ENSMUSG00000038047	RDSLI			
31	>ENSMUSG00000012428	GKSDI	81	>ENSMUSG00000043398	RDSSI			
32	>ENSMUSG00000031737	GMSDI	82	>ENSMUSG00000044216	RESAI			
33	>ENSMUSG00000030228	GNSII	83	>ENSMUSG00000041695	RESEI			
34	>ENSMUSG00000049561	GPSEI	84	>ENSMUSG00000042529	RESEI			
35	>ENSMUSG00000054652	GQSEI	85	>ENSMUSG00000033450	RESYI			
36	>ENSMUSG00000050777	GTSLI	86	>ENSMUSG00000052031	RESYI			
37	>ENSMUSG00000073155	GVSVI	87	>ENSMUSG00000079571	RESYI			
38	>ENSMUSG00000050854	IASLI	88	>ENSMUSG00000005947	RFSKI			
39	>ENSMUSG00000032232	IASQI	89	>ENSMUSG00000028799	RISLI			
40	>ENSMUSG00000034755	ICSEI	90	>ENSMUSG00000019905	RSSSI			
41	>ENSMUSG00000022534	IFSMI	91	>ENSMUSG00000006930	RSSTI			
42	>ENSMUSG00000019936	IGSFI	92	>ENSMUSG00000055951	RSSWI			
43	>ENSMUSG00000000942	IPSSI	93	>ENSMUSG00000017679	RTSDI			
44	>ENSMUSG00000011256	ISSKI	94	>ENSMUSG00000021838	RTSTI			
45	>ENSMUSG00000020102	KESNI	95	>ENSMUSG00000035165	RVSMI			
46	>ENSMUSG00000061393	KESSI	96	>ENSMUSG00000019838	SASII			
47	>ENSMUSG00000079215	KISVI	97	>ENSMUSG00000064354	SASMI			
48	>ENSMUSG00000035021	KKSRI	98	>ENSMUSG00000025920	SKSAI			
49	>ENSMUSG00000024810	KLSKI	99	>ENSMUSG00000031698	SKSCI			
50	>ENSMUSG00000004633	KSSAI	100	>ENSMUSG00000046230	SKSLI			

Additional file 4. continued.

mouse xxSxL

No.	gene ID	5aa	No.	gene ID	5aa	No.	gene ID	5aa	No.	gene ID	5aa	No.	gene ID	5aa
1	>ENSMUSG00000033200	AASEL	51	>ENSMUSG00000061517	FCSQL	101	>ENSMUSG00000029735	IQSSL	151	>ENSMUSG00000071158	LSSDL	201	>ENSMUSG00000023577	QSSFL
2	>ENSMUSG00000052926	AASSL	52	>ENSMUSG00000007415	FFSAL	102	>ENSMUSG00000052390	IRSPL	152	>ENSMUSG00000047490	LSSML	202	>ENSMUSG00000050824	QTSRL
3	>ENSMUSG00000055323	ACSKL	53	>ENSMUSG00000036257	FFSKL	103	>ENSMUSG00000043885	ITSGL	153	>ENSMUSG00000020331	LSSNL	203	>ENSMUSG00000026725	QVSDL
4	>ENSMUSG00000046959	ADSAL	54	>ENSMUSG00000066252	FKSAL	104	>ENSMUSG00000032181	IYSSL	154	>ENSMUSG00000039653	LSSQL	204	>ENSMUSG00000008730	QYSYL
5	>ENSMUSG00000060701	ADSEL	55	>ENSMUSG00000024053	FLSHL	105	>ENSMUSG00000034584	KESEL	155	>ENSMUSG00000070577	LSSSL	205	>ENSMUSG00000060808	RASCL
6	>ENSMUSG00000074266	ADSKL	56	>ENSMUSG00000032679	FLSHL	106	>ENSMUSG00000044629	KESFL	156	>ENSMUSG00000049314	LTSDL	206	>ENSMUSG00000023328	RCSDL
7	>ENSMUSG00000074267	ADSKL	57	>ENSMUSG00000024273	FLSSL	107	>ENSMUSG00000052155	KESSL	157	>ENSMUSG00000029381	LTSPL	207	>ENSMUSG00000003037	RCSLL
8	>ENSMUSG00000074268	ADSKL	58	>ENSMUSG00000029366	FLSTL	108	>ENSMUSG00000020142	KESVL	158	>ENSMUSG00000040680	LVSAL	208	>ENSMUSG00000036943	RCSLL
9	>ENSMUSG00000079850	ADSKL	59	>ENSMUSG00000044807	FNSSL	109	>ENSMUSG00000029122	KGSNL	159	>ENSMUSG00000057985	MASFL	209	>ENSMUSG00000068777	RDSCL
10	>ENSMUSG00000026466	AGSCL	60	>ENSMUSG00000028689	FQSDL	110	>ENSMUSG00000031807	KHSTL	160	>ENSMUSG00000015652	MASRL	210	>ENSMUSG00000071591	RDSCL
11	>ENSMUSG00000057914	AHSSL	61	>ENSMUSG00000022209	FSSKL	111	>ENSMUSG00000063727	KISCL	161	>ENSMUSG00000031861	MDSTL	211	>ENSMUSG00000072741	RDSCL
12	>ENSMUSG00000036790	AISQL	62	>ENSMUSG00000079043	FTSAL	112	>ENSMUSG00000009731	KISSL	162	>ENSMUSG00000025790	MESVL	212	>ENSMUSG00000079365	RDSCL
13	>ENSMUSG00000023938	ALSQL	63	>ENSMUSG00000022594	FWSLL	113	>ENSMUSG00000041702	KKSAL	163	>ENSMUSG00000017307	MGSLL	213	>ENSMUSG00000079385	RDSCL
14	>ENSMUSG00000019579	ARSEL	64	>ENSMUSG00000006457	GESDL	114	>ENSMUSG00000069818	KKSLL	164	>ENSMUSG00000028383	MNSRL	214	>ENSMUSG00000079397	RDSCL
15	>ENSMUSG00000052229	ARSEL	65	>ENSMUSG00000015143	GESDL	115	>ENSMUSG00000075307	KLSKL	165	>ENSMUSG00000039809	MVSGL	215	>ENSMUSG00000079400	RDSCL
16	>ENSMUSG00000029723	ARSPL	66	>ENSMUSG00000052374	GESDL	116	>ENSMUSG00000046667	KLSLL	166	>ENSMUSG00000028525	NCSFL	216	>ENSMUSG00000079404	RDSCL
17	>ENSMUSG00000064293	ARSSL	67	>ENSMUSG00000054808	GESDL	117	>ENSMUSG00000052137	KLSLL	167	>ENSMUSG00000045730	NDSPL	217	>ENSMUSG00000079407	RDSCL
18	>ENSMUSG00000073400	AVSLL	68	>ENSMUSG00000043088	GFSCL	118	>ENSMUSG00000068205	KMSKL	168	>ENSMUSG00000051978	NHSLL	218	>ENSMUSG00000036333	RESIL
19	>ENSMUSG00000073859	CASWL	69	>ENSMUSG00000019464	GFSHL	119	>ENSMUSG00000054115	KPSCL	169	>ENSMUSG00000028992	NHSTL	219	>ENSMUSG00000053475	RFSHL
20	>ENSMUSG00000055976	CDSDL	70	>ENSMUSG00000035020	GGSPL	120	>ENSMUSG00000035649	KPSGL	170	>ENSMUSG00000049232	NKSHL	220	>ENSMUSG00000017767	RGSCL
21	>ENSMUSG00000029076	CLSRL	71	>ENSMUSG00000027457	GGSQL	121	>ENSMUSG00000030256	KPSSL	171	>ENSMUSG00000069170	NLSHL	221	>ENSMUSG00000072653	RGSPL
22	>ENSMUSG00000021680	CLSSL	72	>ENSMUSG00000031845	GGSSL	122	>ENSMUSG00000074449	KSSFL	172	>ENSMUSG00000038095	NLSNL	222	>ENSMUSG00000043487	RHSKL
23	>ENSMUSG00000079237	CTSVL	73	>ENSMUSG00000030612	GLSCL	123	>ENSMUSG00000074455	KSSFL	173	>ENSMUSG00000074064	NNSKL	223	>ENSMUSG00000041187	RISIL
24	>ENSMUSG00000034037	DASVL	74	>ENSMUSG00000008482	GQSVL	124	>ENSMUSG00000074456	KSSFL	174	>ENSMUSG00000004267	NPSVL	224	>ENSMUSG00000073142	RISQL
25	>ENSMUSG00000026457	DDSLL	75	>ENSMUSG00000078865	GRSDL	125	>ENSMUSG00000040896	KVSAL	175	>ENSMUSG00000042249	NSSGL	225	>ENSMUSG00000035431	RISTL
26	>ENSMUSG00000024008	DHSAL	76	>ENSMUSG00000078906	GRSDL	126	>ENSMUSG00000035954	KVSQL	176	>ENSMUSG00000079176	NSSNL	226	>ENSMUSG00000028211	RKSCL
27	>ENSMUSG00000025288	DISQL	77	>ENSMUSG00000022598	GSSRL	127	>ENSMUSG00000028655	LASIL	177	>ENSMUSG00000028457	NYSFL	227	>ENSMUSG00000016024	RKSFL
28	>ENSMUSG00000015189	DKSRL	78	>ENSMUSG00000075335	GSSYL	128	>ENSMUSG00000031858	LASLL	178	>ENSMUSG00000046947	PASFL	228	>ENSMUSG00000039904	RLSHL
29	>ENSMUSG00000022176	DLSVL	79	>ENSMUSG00000028399	HCSLL	129	>ENSMUSG00000033863	LASPL	179	>ENSMUSG00000033220	PCSLL	229	>ENSMUSG00000056629	RRSEL
30	>ENSMUSG00000028214	DLSVL	80	>ENSMUSG00000039079	HFSVL	130	>ENSMUSG00000078374	LCSLL	180	>ENSMUSG00000019894	PESDL	230	>ENSMUSG00000020185	RRSGL
31	>ENSMUSG00000031880	DLSVL	81	>ENSMUSG00000027309	HLSLL	131	>ENSMUSG00000078375	LCSLL	181	>ENSMUSG00000027894	PESEL	231	>ENSMUSG00000038497	RRSSL
32	>ENSMUSG00000030530	DQSAL	82	>ENSMUSG00000055945	HLSTL	132	>ENSMUSG00000079620	LDSEL	182	>ENSMUSG00000037434	PESSL	232	>ENSMUSG00000048921	RSSAL
33	>ENSMUSG00000021097	DVSRL	83	>ENSMUSG00000028289	HLSVL	133	>ENSMUSG00000027942	LESTL	183	>ENSMUSG00000019854	PFSHL	233	>ENSMUSG00000059013	RSSEL
34	>ENSMUSG00000042106	DVSYL	84	>ENSMUSG00000034216	HLSWL	134	>ENSMUSG00000078697	LESTL	184	>ENSMUSG00000028565	PFSTL	234	>ENSMUSG00000060882	RVSAL
35	>ENSMUSG00000024620	EDSFL	85	>ENSMUSG00000062791	HPSKL	135	>ENSMUSG00000020733	LFSNL	185	>ENSMUSG00000060814	PGSGL	235	>ENSMUSG00000002688	RVSIL
36	>ENSMUSG00000029231	EDSFL	86	>ENSMUSG00000039611	HPSLL	136	>ENSMUSG00000037544	LFSPL	186	>ENSMUSG00000036678	PHSHL	236	>ENSMUSG00000015966	SCSPL
37	>ENSMUSG00000072946	EDSSL	87	>ENSMUSG00000044056	HRSIL	137	>ENSMUSG00000052957	LGSHL	187	>ENSMUSG00000053746	PLSGL	237	>ENSMUSG00000028289	SCSRL
38	>ENSMUSG00000046658	EESIL	88	>ENSMUSG00000022853	HSSKL	138	>ENSMUSG00000031979	LHSGL	188	>ENSMUSG00000033557	PLSHL	238	>ENSMUSG00000022759	SDSGL
39	>ENSMUSG00000000531	EESQL	89	>ENSMUSG00000021532	IASAL	139	>ENSMUSG00000029098	LKSQL	189	>ENSMUSG00000020897	PPSAL	239	>ENSMUSG00000017307	SESKL
40	>ENSMUSG00000078933	EHSAL	90	>ENSMUSG00000078233	IASFL	140	>ENSMUSG00000021620	LKSVL	190	>ENSMUSG00000042096	PPSHL	240	>ENSMUSG00000050945	SESRL
41	>ENSMUSG00000035946	EISPL	91	>ENSMUSG00000061163	IASVL	141	>ENSMUSG00000031841	LLSGL	191	>ENSMUSG00000041936	PPSLL	241	>ENSMUSG00000066595	SESSL
42	>ENSMUSG00000038791	EQSKL	92	>ENSMUSG00000047866	IDSKL	142	>ENSMUSG00000074054	LLSLL	192	>ENSMUSG00000004892	PPSSL	242	>ENSMUSG00000026509	SFSVL
43	>ENSMUSG00000058005	ESSQL	93	>ENSMUSG00000020181	IESTL	143	>ENSMUSG00000024002	LPSEL	193	>ENSMUSG00000020087	PRSKL	243	>ENSMUSG00000032899	SFSVL
44	>ENSMUSG00000042190	ETSTL	94	>ENSMUSG00000073871	IFSLL	144	>ENSMUSG00000062785	LPSIL	194	>ENSMUSG00000036103	PSSIL	244	>ENSMUSG00000060317	SGSKL
45	>ENSMUSG00000028329	EWSRL	95	>ENSMUSG00000073880	IFSLL	145	>ENSMUSG00000032338	LPSNL	195	>ENSMUSG00000073429	PVSKL	245	>ENSMUSG00000070985	SGSKL
46	>ENSMUSG00000066307	EYSSL	96	>ENSMUSG00000078244	IFSLL	146	>ENSMUSG00000028186	LPSRL	196	>ENSMUSG00000067714	QDSAL	246	>ENSMUSG00000054280	SGSPL
47	>ENSMUSG00000042793	FASHL	97	>ENSMUSG00000078360	IFSLL	147	>ENSMUSG00000020777	LQSKL	197	>ENSMUSG00000030498	QESAL	247	>ENSMUSG00000054931	SHSDL
48	>ENSMUSG00000021730	FASNL	98	>ENSMUSG00000024953	ILSQL	148	>ENSMUSG00000039671	LQSKL	198	>ENSMUSG00000040061	QESRL	248	>ENSMUSG00000057335	SHSTL
49	>ENSMUSG00000079302	FCSIL	99	>ENSMUSG00000040601	ILSSL	149	>ENSMUSG00000020213	LQSLL	199	>ENSMUSG00000027870	QFSRL	249	>ENSMUSG00000049538	SKSNL
50	>ENSMUSG00000079833	FCSIL	100	>ENSMUSG00000036231	IQSEL	150	>ENSMUSG00000026737	LRSRL	200	>ENSMUSG00000021265	QPSSL	250	>ENSMUSG00000031767	SLSKL

Additional file 4. continued.

mouse xxSxL

No.	gene ID	5aa
251	>ENSMUSG00000063428	SLSKL
252	>ENSMUSG00000026761	SLSWL
253	>ENSMUSG00000023353	SPSIL
254	>ENSMUSG00000068114	SQSEL
255	>ENSMUSG00000079611	SRSEL
256	>ENSMUSG00000079462	SRSHL
257	>ENSMUSG00000005763	SSSQL
258	>ENSMUSG00000062393	SSSQL
259	>ENSMUSG00000049583	SSSSL
260	>ENSMUSG00000019828	SSSTL
261	>ENSMUSG00000041607	STSPL
262	>ENSMUSG00000030269	STSTL
263	>ENSMUSG00000001497	TASAL
264	>ENSMUSG00000062868	TASLL
265	>ENSMUSG00000054252	TCSEL
266	>ENSMUSG00000056632	TCSHL
267	>ENSMUSG00000053898	TFSKL
268	>ENSMUSG00000079179	TFSLL
269	>ENSMUSG00000031327	TLSAL
270	>ENSMUSG00000052712	TLSDL
271	>ENSMUSG00000044933	TLSHL
272	>ENSMUSG00000032489	TMSLL
273	>ENSMUSG00000022210	TPSRL
274	>ENSMUSG00000066178	TQSSL
275	>ENSMUSG00000024095	TSSHL
276	>ENSMUSG00000038522	TSSRL
277	>ENSMUSG00000040325	TSSYL
278	>ENSMUSG00000063952	TSSYL
279	>ENSMUSG00000049927	TTSCL
280	>ENSMUSG00000003974	TTSSL
281	>ENSMUSG00000023192	TTSSL
282	>ENSMUSG00000068523	VCSFL
283	>ENSMUSG00000078297	VCSFL
284	>ENSMUSG00000019970	VDSFL
285	>ENSMUSG00000059336	VESPL
286	>ENSMUSG00000071489	VESTL
287	>ENSMUSG00000078695	VGSPL
288	>ENSMUSG00000035614	VLSEL
289	>ENSMUSG00000033177	VPSLL
290	>ENSMUSG00000049090	VSSKL
291	>ENSMUSG00000016346	VTSQL
292	>ENSMUSG00000042292	WDSCL
293	>ENSMUSG00000048118	WISFL
294	>ENSMUSG00000078181	WSSLL
295	>ENSMUSG00000073639	YCSVL
296	>ENSMUSG00000035067	YESSL
297	>ENSMUSG00000053046	YESSL
298	>ENSMUSG00000032068	YISTL
299	>ENSMUSG00000072883	YLSGL
300	>ENSMUSG00000037366	YLSSL
301	>ENSMUSG00000027942	YMSTL
302	>ENSMUSG00000004651	YQSHL
303	>ENSMUSG00000026182	YRSHL
304	>ENSMUSG00000073542	YRSVL
305	>ENSMUSG00000055725	YVSHL
306	>ENSMUSG00000051331	YVSNL

Additional file 4. continued.

mouse xxSxV

No.	gene ID	5aa	No.	gene ID	5aa	No.	gene ID	5aa	No.	gene ID	5aa	No.	gene ID	5aa
1	>ENSMUSG00000008348	ACSWV	51	>ENSMUSG00000045471	GGSGV	101	>ENSMUSG00000021745	MESLV	151	>ENSMUSG00000031557	RTSDV	201	>ENSMUSG00000079258	YFSKV
2	>ENSMUSG00000028645	ADSQV	52	>ENSMUSG00000026021	GHSTV	102	>ENSMUSG00000040490	MESTV	152	>ENSMUSG00000048620	RWSSV	202	>ENSMUSG00000063163	YISSV
3	>ENSMUSG00000021493	AFSHV	53	>ENSMUSG00000047840	GPSCV	103	>ENSMUSG00000026104	MMSTV	153	>ENSMUSG00000020718	SASNV	203	>ENSMUSG00000005089	YQSWV
4	>ENSMUSG00000027956	AFSKV	54	>ENSMUSG00000078531	GSSGV	104	>ENSMUSG00000020598	MNSFV	154	>ENSMUSG00000035283	SESKV			
5	>ENSMUSG00000024970	AGSGV	55	>ENSMUSG00000037159	GTSSV	105	>ENSMUSG00000043301	NESKV	155	>ENSMUSG00000038026	SESKV			
6	>ENSMUSG00000001946	AGSLV	56	>ENSMUSG00000059939	HISNV	106	>ENSMUSG00000046942	NFSQV	156	>ENSMUSG00000027253	SESQV			
7	>ENSMUSG00000022790	AGSLV	57	>ENSMUSG00000035842	HPSLV	107	>ENSMUSG00000020074	NGSGV	157	>ENSMUSG00000038578	SFSAV			
8	>ENSMUSG00000051246	AKSNV	58	>ENSMUSG00000062040	HTSAV	108	>ENSMUSG00000022906	NGSSV	158	>ENSMUSG00000067235	SHSLV			
9	>ENSMUSG00000062070	ALSNV	59	>ENSMUSG00000061614	IASIV	109	>ENSMUSG00000005994	NHSMV	159	>ENSMUSG00000027684	SISHV			
10	>ENSMUSG00000034416	ALSRV	60	>ENSMUSG00000038540	ICSNV	110	>ENSMUSG00000050545	NPSAV	160	>ENSMUSG00000031026	SISQV			
11	>ENSMUSG00000075210	APSEV	61	>ENSMUSG00000030209	IESDV	111	>ENSMUSG00000041907	NQSAV	161	>ENSMUSG00000045005	SLSHV			
12	>ENSMUSG00000000120	ATSPV	62	>ENSMUSG00000059003	IESDV	112	>ENSMUSG00000025993	NTSVV	162	>ENSMUSG00000035274	SNSDV			
13	>ENSMUSG00000020732	CCSFV	63	>ENSMUSG00000035168	IESNV	113	>ENSMUSG00000002617	PASHV	163	>ENSMUSG00000041468	SPSDV			
14	>ENSMUSG00000028328	CRSGV	64	>ENSMUSG00000041605	IISQV	114	>ENSMUSG00000048249	PASKV	164	>ENSMUSG00000049649	SPSDV			
15	>ENSMUSG00000068284	CSSAV	65	>ENSMUSG00000034690	ILSSV	115	>ENSMUSG00000068149	PASSV	165	>ENSMUSG00000046922	SPSEV			
16	>ENSMUSG00000055567	DESHV	66	>ENSMUSG00000041343	ISSQV	116	>ENSMUSG00000073995	PASSV	166	>ENSMUSG00000033821	SPSNV			
17	>ENSMUSG00000025207	DESSV	67	>ENSMUSG00000029199	KASKV	117	>ENSMUSG00000022240	PDSWV	167	>ENSMUSG00000028521	SPSPV			
18	>ENSMUSG00000022865	DGSIV	68	>ENSMUSG00000029199	KASKV	118	>ENSMUSG00000026991	PDSWV	168	>ENSMUSG00000040797	SRSLV			
19	>ENSMUSG00000040569	DHSEV	69	>ENSMUSG00000001027	KESLV	119	>ENSMUSG00000032021	PESIV	169	>ENSMUSG00000038936	SSSEV			
20	>ENSMUSG00000038668	DHSVV	70	>ENSMUSG00000015243	KESYV	120	>ENSMUSG00000016520	PGSLV	170	>ENSMUSG00000041717	SSSMV			
21	>ENSMUSG00000044017	DLSAV	71	>ENSMUSG00000020042	KGSVV	121	>ENSMUSG00000045503	PKSNV	171	>ENSMUSG00000025558	SSSVV			
22	>ENSMUSG00000014782	DLSCV	72	>ENSMUSG00000042742	KISTV	122	>ENSMUSG00000031245	PLSIV	172	>ENSMUSG00000032491	SSSVV			
23	>ENSMUSG00000029516	DQSSV	73	>ENSMUSG00000034265	KLSSV	123	>ENSMUSG00000036904	PLSQV	173	>ENSMUSG00000023828	STSDV			
24	>ENSMUSG00000025184	DQSVV	74	>ENSMUSG00000034913	KPSRV	124	>ENSMUSG00000051146	PPSGV	174	>ENSMUSG00000022969	STSEV			
25	>ENSMUSG00000037016	DSSEV	75	>ENSMUSG00000032776	KRSTV	125	>ENSMUSG00000042845	PQSKV	175	>ENSMUSG00000038167	STSEV			
26	>ENSMUSG00000027070	EDSDV	76	>ENSMUSG00000074881	KSSNV	126	>ENSMUSG00000054206	PQSLV	176	>ENSMUSG00000067356	STSEV			
27	>ENSMUSG00000016487	EDSNV	77	>ENSMUSG00000037531	KSSSV	127	>ENSMUSG00000031980	PQSVV	177	>ENSMUSG00000057789	STSVV			
28	>ENSMUSG00000067878	EESDV	78	>ENSMUSG00000026389	KTSHV	128	>ENSMUSG00000040268	PVSDV	178	>ENSMUSG00000055811	STSYV			
29	>ENSMUSG00000048782	EESFV	79	>ENSMUSG00000034997	KVSCV	129	>ENSMUSG00000028581	PYSEV	179	>ENSMUSG00000036411	TASAV			
30	>ENSMUSG00000032902	EESPV	80	>ENSMUSG00000049119	KVSHV	130	>ENSMUSG00000035967	PYSLV	180	>ENSMUSG00000039713	TASEV			
31	>ENSMUSG00000026121	EESSV	81	>ENSMUSG00000068327	LASVV	131	>ENSMUSG00000031967	QESPV	181	>ENSMUSG00000079020	TESMV			
32	>ENSMUSG00000045045	EESVV	82	>ENSMUSG00000040321	LDSTV	132	>ENSMUSG00000027962	QKSKV	182	>ENSMUSG00000017978	TISLV			
33	>ENSMUSG00000020686	EESWV	83	>ENSMUSG00000067825	LDSTV	133	>ENSMUSG00000022801	QLSAV	183	>ENSMUSG00000060640	TKSEV			
34	>ENSMUSG00000015932	EGSPV	84	>ENSMUSG00000002771	LESEV	134	>ENSMUSG00000062609	QQSNV	184	>ENSMUSG00000068385	TKSEV			
35	>ENSMUSG00000023949	ENSVV	85	>ENSMUSG00000020734	LESEV	135	>ENSMUSG00000028028	QTSKV	185	>ENSMUSG00000031481	TMSSV			
36	>ENSMUSG00000051839	ESSNV	86	>ENSMUSG00000068748	LESLV	136	>ENSMUSG00000037533	QVSAV	186	>ENSMUSG00000061388	TPSQV			
37	>ENSMUSG00000026188	ETSTV	87	>ENSMUSG00000044005	LESMV	137	>ENSMUSG00000062232	QVSAV	187	>ENSMUSG00000031258	TQSIV			
38	>ENSMUSG00000032528	EVSLV	88	>ENSMUSG00000030600	LESTV	138	>ENSMUSG00000045202	RASGV	188	>ENSMUSG00000020872	TTSNV			
39	>ENSMUSG00000020696	FHSYV	89	>ENSMUSG00000059303	LFSDV	139	>ENSMUSG00000042472	RASRV	189	>ENSMUSG00000074030	TTSVV			
40	>ENSMUSG00000032690	FLSFV	90	>ENSMUSG00000048400	LGSGV	140	>ENSMUSG00000024245	RDSTV	190	>ENSMUSG00000037762	VASNV			
41	>ENSMUSG00000053647	FSSAV	91	>ENSMUSG00000038174	LISQV	141	>ENSMUSG00000030539	RDSVV	191	>ENSMUSG00000000325	VDSWV			
42	>ENSMUSG00000039683	FSSFV	92	>ENSMUSG00000029729	LKSCV	142	>ENSMUSG00000050105	RESEV	192	>ENSMUSG00000053580	VESNV			
43	>ENSMUSG00000041592	FSSFV	93	>ENSMUSG00000024601	LLSKV	143	>ENSMUSG00000032511	RESIV	193	>ENSMUSG00000021576	VFSIV			
44	>ENSMUSG00000058624	FSSSV	94	>ENSMUSG00000050635	LPSKV	144	>ENSMUSG00000056537	RESVV	194	>ENSMUSG00000024411	VLSSV			
45	>ENSMUSG00000072749	FVSLV	95	>ENSMUSG00000038963	LQSNV	145	>ENSMUSG00000044708	RISNV	195	>ENSMUSG00000040610	VTSLV			
46	>ENSMUSG00000022490	GASLV	96	>ENSMUSG00000034930	LQSPV	146	>ENSMUSG00000041380	RISSV	196	>ENSMUSG00000054102	VTSLV			
47	>ENSMUSG00000079455	GASRV	97	>ENSMUSG00000053062	LSSSV	147	>ENSMUSG00000070900	RLSGV	197	>ENSMUSG00000032724	VTSRV			
48	>ENSMUSG00000079799	GASRV	98	>ENSMUSG00000015950	LTSAV	148	>ENSMUSG00000036106	RPSVV	198	>ENSMUSG00000032998	WDSIV			
49	>ENSMUSG00000031431	GGSAV	99	>ENSMUSG00000070632	LVSIV	149	>ENSMUSG00000032750	RQSKV	199	>ENSMUSG00000073377	YASCV			
50	>ENSMUSG00000036099	GGSGV	100	>ENSMUSG00000025372	LVSTV	150	>ENSMUSG00000049265	RRSSV	200	>ENSMUSG00000031647	YESHV			

Additional file 4. continued.

mouse xxTxI

No.	gene ID	5aa	No.	gene ID	5aa
1	>ENSMUSG00000032352	AATAI	51	>ENSMUSG00000049939	QETQI
2	>ENSMUSG00000002028	AATLI	52	>ENSMUSG00000050587	QETQI
3	>ENSMUSG00000075415	AKTYI	53	>ENSMUSG00000032479	QETSI
4	>ENSMUSG00000026383	AMTEI	54	>ENSMUSG00000029534	QLTRI
5	>ENSMUSG00000039735	AVTYI	55	>ENSMUSG00000061259	QQTGI
6	>ENSMUSG00000050270	CKTVI	56	>ENSMUSG00000027335	RETDI
7	>ENSMUSG00000044538	CYTHI	57	>ENSMUSG00000022126	RFTNI
8	>ENSMUSG00000004668	CYTRI	58	>ENSMUSG00000071424	RGTSI
9	>ENSMUSG00000000627	DETSI	59	>ENSMUSG00000032135	RNTSI
10	>ENSMUSG00000040006	DKTDI	60	>ENSMUSG00000029554	RQTSI
11	>ENSMUSG00000027940	DMTSI	61	>ENSMUSG00000006301	RSTQI
12	>ENSMUSG00000032366	DMTSI	62	>ENSMUSG00000035148	SETEI
13	>ENSMUSG00000025497	DNTYI	63	>ENSMUSG00000045802	SETII
14	>ENSMUSG00000026955	DSTFI	64	>ENSMUSG00000067649	SHTNI
15	>ENSMUSG00000049008	EETII	65	>ENSMUSG00000027887	SITGI
16	>ENSMUSG00000041193	EGTLI	66	>ENSMUSG00000035861	SKTGI
17	>ENSMUSG00000034647	ELTPI	67	>ENSMUSG00000079451	SKTSI
18	>ENSMUSG00000037703	ESTEI	68	>ENSMUSG00000042367	SLTPI
19	>ENSMUSG00000019989	FETII	69	>ENSMUSG00000054537	SNTGI
20	>ENSMUSG00000023943	FRTEI	70	>ENSMUSG00000043760	SRTSI
21	>ENSMUSG00000079135	FRTEI	71	>ENSMUSG00000035342	TATEI
22	>ENSMUSG00000041078	HGTSI	72	>ENSMUSG00000053199	TGTDI
23	>ENSMUSG00000030641	HKTNI	73	>ENSMUSG00000020656	TLTEI
24	>ENSMUSG00000026201	HTTQI	74	>ENSMUSG00000002031	TLTFI
25	>ENSMUSG00000036306	IATEI	75	>ENSMUSG00000035722	VETMI
26	>ENSMUSG00000024026	IATII	76	>ENSMUSG00000024008	VHTHI
27	>ENSMUSG00000006782	ICTII	77	>ENSMUSG00000032503	VPTAI
28	>ENSMUSG00000005089	IETCI	78	>ENSMUSG00000022280	VQTEI
29	>ENSMUSG00000038071	IPTGI	79	>ENSMUSG00000018750	VRTQI
30	>ENSMUSG00000034810	IQTQI	80	>ENSMUSG00000045348	WDTAI
31	>ENSMUSG00000029992	IWTWI	81	>ENSMUSG00000054976	WDTAI
32	>ENSMUSG00000024096	KETPI	82	>ENSMUSG00000040537	WETSI
33	>ENSMUSG00000022914	KGTLI	83	>ENSMUSG00000035351	WVTEI
34	>ENSMUSG00000059631	KLTVI	84	>ENSMUSG00000028865	YQTLI
35	>ENSMUSG00000061096	KSTFI	85	>ENSMUSG00000005045	YVTDI
36	>ENSMUSG00000066196	KTTPI	86	>ENSMUSG00000032220	YVTKI
37	>ENSMUSG00000018387	LATLI			
38	>ENSMUSG00000058621	LGTKI			
39	>ENSMUSG00000021280	LITCI			
40	>ENSMUSG00000002489	LNTEI			
41	>ENSMUSG00000052560	LQTQI			
42	>ENSMUSG00000030617	MKTFI			
43	>ENSMUSG00000067049	MKTKI			
44	>ENSMUSG00000038175	NLTVI			
45	>ENSMUSG00000040396	NVTII			
46	>ENSMUSG00000016128	PETKI			
47	>ENSMUSG00000027312	PGTCI			
48	>ENSMUSG00000045179	PLTHI			
49	>ENSMUSG00000079994	PLTHI			
50	>ENSMUSG00000047085	QETQI			

Additional file 4. continued.

mouse xxTxL

No.	gene ID	5aa	No.	gene ID	5aa	No.	gene ID	5aa	No.	gene ID	5aa	No.	gene ID	5aa
1	>ENSMUSG00000030660	AATYL	51	>ENSMUSG00000039194	ENTAL	101	>ENSMUSG00000020826	KATRL	151	>ENSMUSG00000028434	LMTAL	201	>ENSMUSG00000037995	QATLL
2	>ENSMUSG00000016494	ADTEL	52	>ENSMUSG00000024960	ENTQL	102	>ENSMUSG00000025739	KCTIL	152	>ENSMUSG00000028434	LMTEL	202	>ENSMUSG00000002791	QATVL
3	>ENSMUSG00000069170	ADTHL	53	>ENSMUSG00000030766	ESTAL	103	>ENSMUSG00000028270	KCTIL	153	>ENSMUSG00000037447	LNTKL	203	>ENSMUSG00000032788	QATVL
4	>ENSMUSG00000000792	AETNL	54	>ENSMUSG00000033389	ESTAL	104	>ENSMUSG00000022610	KETAL	154	>ENSMUSG00000001056	LPTPL	204	>ENSMUSG00000038212	QCTEL
5	>ENSMUSG00000021733	AETSL	55	>ENSMUSG00000050870	ESTTL	105	>ENSMUSG00000030109	KETHL	155	>ENSMUSG00000041406	LPTWL	205	>ENSMUSG00000041301	QETRL
6	>ENSMUSG00000046916	APTCL	56	>ENSMUSG00000073598	EVTYL	106	>ENSMUSG00000078761	KETPL	156	>ENSMUSG00000022582	LQTFL	206	>ENSMUSG00000006641	QETSL
7	>ENSMUSG00000007035	APTIL	57	>ENSMUSG00000021303	FCTIL	107	>ENSMUSG00000045980	KETQL	157	>ENSMUSG00000022288	LQTLL	207	>ENSMUSG00000020640	QKTLL
8	>ENSMUSG00000034911	AQTFL	58	>ENSMUSG00000048232	FCTIL	108	>ENSMUSG00000030235	KETRL	158	>ENSMUSG00000022583	LQTLL	208	>ENSMUSG00000030741	QMTGL
9	>ENSMUSG00000075510	AQTLL	59	>ENSMUSG00000063594	FCTLL	109	>ENSMUSG00000050097	KHTEL	159	>ENSMUSG00000022584	LQTLL	209	>ENSMUSG00000037736	QPTTL
10	>ENSMUSG00000003279	AQTRL	60	>ENSMUSG00000031732	FDTAL	110	>ENSMUSG00000078439	KKTAL	160	>ENSMUSG00000075601	LQTLL	210	>ENSMUSG00000061298	QRTHL
11	>ENSMUSG00000042388	AQTRL	61	>ENSMUSG00000006932	FDTDL	111	>ENSMUSG00000042357	KKTIL	161	>ENSMUSG00000075602	LQTLL	211	>ENSMUSG00000052613	QSTSL
12	>ENSMUSG00000047495	AQTRL	62	>ENSMUSG00000051177	FDTPL	112	>ENSMUSG00000030754	KKTSL	162	>ENSMUSG00000079018	LQTLL	212	>ENSMUSG00000051154	RATQL
13	>ENSMUSG00000061689	AQTRL	63	>ENSMUSG00000032849	FETAL	113	>ENSMUSG00000048763	KLTHL	163	>ENSMUSG00000026383	LTTEL	213	>ENSMUSG00000024812	RDTEL
14	>ENSMUSG00000032575	ARTDL	64	>ENSMUSG00000020631	FETFL	114	>ENSMUSG00000079277	KLTHL	164	>ENSMUSG00000038276	LVTRL	214	>ENSMUSG00000026904	RETCL
15	>ENSMUSG00000035615	ASTAL	65	>ENSMUSG00000027803	FLTWL	115	>ENSMUSG00000079560	KLTHL	165	>ENSMUSG00000013033	LVTSL	215	>ENSMUSG00000045534	RETDL
16	>ENSMUSG00000033306	ASTDL	66	>ENSMUSG00000053110	FLTWL	116	>ENSMUSG00000036083	KLTRL	166	>ENSMUSG00000028184	LVTSL	216	>ENSMUSG00000032657	RETGL
17	>ENSMUSG00000049526	ATTRL	67	>ENSMUSG00000022350	FRTVL	117	>ENSMUSG00000022899	KNTRL	167	>ENSMUSG00000037605	LVTSL	217	>ENSMUSG00000041624	RETSL
18	>ENSMUSG00000015968	CITTL	68	>ENSMUSG00000048939	FWTRL	118	>ENSMUSG00000028992	KPTGL	168	>ENSMUSG00000031376	METSL	218	>ENSMUSG00000071604	RETVL
19	>ENSMUSG00000066772	CYTHL	69	>ENSMUSG00000006313	FYTTL	119	>ENSMUSG00000020963	KQTAL	169	>ENSMUSG00000018669	MGTSL	219	>ENSMUSG00000026502	RHTKL
20	>ENSMUSG00000074369	CYTHL	70	>ENSMUSG00000020524	GATGL	120	>ENSMUSG00000040383	KQTVL	170	>ENSMUSG00000074875	MHTKL	220	>ENSMUSG00000044162	RKTDL
21	>ENSMUSG00000033792	DDTTL	71	>ENSMUSG00000027603	GATIL	121	>ENSMUSG00000047804	KSTKL	171	>ENSMUSG00000061742	MSTRL	221	>ENSMUSG00000030237	RKTKL
22	>ENSMUSG00000031511	DETNL	72	>ENSMUSG00000074088	GATLL	122	>ENSMUSG00000074910	KSTTL	172	>ENSMUSG00000079042	MTTSL	222	>ENSMUSG00000060549	RKTLL
23	>ENSMUSG00000058740	DETQL	73	>ENSMUSG00000041479	GATQL	123	>ENSMUSG00000047428	KTTAL	173	>ENSMUSG00000021490	NATRL	223	>ENSMUSG00000060725	RKTLL
24	>ENSMUSG00000024727	DHTRL	74	>ENSMUSG00000064043	GDTEL	124	>ENSMUSG00000021335	KTTRL	174	>ENSMUSG00000071856	NETSL	224	>ENSMUSG00000063610	RKTLL
25	>ENSMUSG00000075394	DITRL	75	>ENSMUSG00000027765	GDTSL	125	>ENSMUSG00000031385	KVTDL	175	>ENSMUSG00000020062	NGTRL	225	>ENSMUSG00000015401	RLTPL
26	>ENSMUSG00000028464	DITSL	76	>ENSMUSG00000068323	GETTL	126	>ENSMUSG00000036606	KVTDL	176	>ENSMUSG00000025194	NHTEL	226	>ENSMUSG00000034401	RMTHL
27	>ENSMUSG00000032889	DLTEL	77	>ENSMUSG00000024903	GHTAL	127	>ENSMUSG00000053646	KVTDL	177	>ENSMUSG00000049551	NPTHL	227	>ENSMUSG00000043913	RPTLL
28	>ENSMUSG00000044795	DLTEL	78	>ENSMUSG00000074156	GHTEL	128	>ENSMUSG00000024757	KVTPL	178	>ENSMUSG00000061947	NPTVL	228	>ENSMUSG00000060256	RPTQL
29	>ENSMUSG00000067276	DLTEL	79	>ENSMUSG00000020948	GLTAL	129	>ENSMUSG00000075044	KVTRL	179	>ENSMUSG00000004319	NSTIL	229	>ENSMUSG00000074432	RPTQL
30	>ENSMUSG00000026942	DLTGL	80	>ENSMUSG00000040852	GPTLL	130	>ENSMUSG00000009894	LATVL	180	>ENSMUSG00000028396	NTTVL	230	>ENSMUSG00000032487	RSTEL
31	>ENSMUSG00000020814	DLTSL	81	>ENSMUSG00000048174	GRTRL	131	>ENSMUSG00000029245	LCTDL	181	>ENSMUSG00000034917	PATDL	231	>ENSMUSG00000047250	RSTEL
32	>ENSMUSG00000035964	DLTTL	82	>ENSMUSG00000073779	GSTIL	132	>ENSMUSG00000019943	LETSL	182	>ENSMUSG00000035364	PATIL	232	>ENSMUSG00000042102	RTTKL
33	>ENSMUSG00000042464	DLTTL	83	>ENSMUSG00000055397	GSTKL	133	>ENSMUSG00000030302	LETSL	183	>ENSMUSG00000054115	PDTLL	233	>ENSMUSG00000045826	RVTAL
34	>ENSMUSG00000022540	DPTHL	84	>ENSMUSG00000066990	HFTTL	134	>ENSMUSG00000078908	LFTGL	184	>ENSMUSG00000031216	PETKL	234	>ENSMUSG00000027792	SCTAL
35	>ENSMUSG00000034918	DTTDL	85	>ENSMUSG00000061356	HGTKL	135	>ENSMUSG00000035649	LFTLL	185	>ENSMUSG00000029228	PGTFL	235	>ENSMUSG00000020310	SCTHL
36	>ENSMUSG00000028701	DVTFL	86	>ENSMUSG00000040372	HRTVL	136	>ENSMUSG00000030207	LHTVL	186	>ENSMUSG00000052613	PMTKL	236	>ENSMUSG00000028269	SCTIL
37	>ENSMUSG00000027508	DVTRL	87	>ENSMUSG00000037474	HSTEL	137	>ENSMUSG00000020581	LKTEL	187	>ENSMUSG00000000782	PMTVL	237	>ENSMUSG00000031174	SCTIL
38	>ENSMUSG00000045288	EDTEL	88	>ENSMUSG00000071770	HSTFL	138	>ENSMUSG00000029998	LKTEL	188	>ENSMUSG00000024112	PPTGL	238	>ENSMUSG00000024885	SCTLL
39	>ENSMUSG00000025608	EDTHL	89	>ENSMUSG00000071771	HSTFL	139	>ENSMUSG00000034064	LKTEL	189	>ENSMUSG00000039496	PQTEL	239	>ENSMUSG00000037263	SCTLL
40	>ENSMUSG00000033152	EDTHL	90	>ENSMUSG00000079628	HSTFL	140	>ENSMUSG00000041698	LKTKL	190	>ENSMUSG00000045750	PQTFL	240	>ENSMUSG00000075296	SCTLL
41	>ENSMUSG00000050810	EDTTL	91	>ENSMUSG00000079630	HSTFL	141	>ENSMUSG00000063975	LKTKL	191	>ENSMUSG00000029919	PQTKL	241	>ENSMUSG00000006269	SDTAL
42	>ENSMUSG00000034412	EDTYL	92	>ENSMUSG00000079633	HSTFL	142	>ENSMUSG00000079262	LKTKL	192	>ENSMUSG00000042642	PRTLL	242	>ENSMUSG00000020839	SETAL
43	>ENSMUSG00000052726	EETQL	93	>ENSMUSG00000079635	HSTFL	143	>ENSMUSG00000079263	LKTKL	193	>ENSMUSG00000019947	PSTKL	243	>ENSMUSG00000030236	SETPL
44	>ENSMUSG00000020021	EGTVL	94	>ENSMUSG00000079639	HSTFL	144	>ENSMUSG00000004371	LKTRL	194	>ENSMUSG00000047517	PSTKL	244	>ENSMUSG00000022701	SFTLL
45	>ENSMUSG00000061959	EHTEL	95	>ENSMUSG00000070526	HSTNL	145	>ENSMUSG00000028229	LLTGL	195	>ENSMUSG00000049657	PSTLL	245	>ENSMUSG00000056602	SGTSL
46	>ENSMUSG00000012819	EITEL	96	>ENSMUSG00000036459	HVTEL	146	>ENSMUSG00000020315	LLTLL	196	>ENSMUSG00000003644	PSTTL	246	>ENSMUSG00000048764	SKTGL
47	>ENSMUSG00000042678	EITLL	97	>ENSMUSG00000040785	INTVL	147	>ENSMUSG00000080025	LLTML	197	>ENSMUSG00000004562	PTTPL	247	>ENSMUSG00000061184	SKTGL
48	>ENSMUSG00000050685	EITRL	98	>ENSMUSG00000027225	ITTLL	148	>ENSMUSG00000072843	LLTSL	198	>ENSMUSG00000041287	PVTHL	248	>ENSMUSG00000072845	SKTGL
49	>ENSMUSG00000027999	EKTDL	99	>ENSMUSG00000050884	ITTSL	149	>ENSMUSG00000071172	LLTTL	199	>ENSMUSG00000060657	PVTKL	249	>ENSMUSG00000043510	SKTPL
50	>ENSMUSG00000034833	ELTNL	100	>ENSMUSG00000066910	ITTSL	150	>ENSMUSG00000059119	LLTVL	200	>ENSMUSG00000040855	PVTVL	250	>ENSMUSG00000073482	SKTPL

Additional file 4. continued.

mouse xxTxL

No.	gene ID	5aa
251	>ENSMUSG00000079973	SLTFL
252	>ENSMUSG00000050106	SRTEL
253	>ENSMUSG00000029190	SRTHL
254	>ENSMUSG00000055124	SRTRL
255	>ENSMUSG00000060538	SRTRL
256	>ENSMUSG00000025573	SSTGL
257	>ENSMUSG00000050147	SSTLL
258	>ENSMUSG00000045362	SSTML
259	>ENSMUSG00000007783	SSTNL
260	>ENSMUSG00000078484	SSTSL
261	>ENSMUSG00000029452	SSTVL
262	>ENSMUSG00000046321	SVTHL
263	>ENSMUSG00000002718	SVTLL
264	>ENSMUSG00000027978	SVTSL
265	>ENSMUSG00000075184	SWTLL
266	>ENSMUSG00000021120	TATSL
267	>ENSMUSG00000037344	TCTDL
268	>ENSMUSG00000026609	TDTHL
269	>ENSMUSG00000022125	TFTDL
270	>ENSMUSG00000021336	TFTHL
271	>ENSMUSG00000033628	TKTDL
272	>ENSMUSG00000026648	TKTLL
273	>ENSMUSG00000042268	TLTAL
274	>ENSMUSG00000036110	TLTRL
275	>ENSMUSG00000038836	TLTSL
276	>ENSMUSG00000029219	TQTAL
277	>ENSMUSG00000031309	TSTAL
278	>ENSMUSG00000025665	TSTGL
279	>ENSMUSG00000023809	TSTRL
280	>ENSMUSG00000026180	TSTTL
281	>ENSMUSG00000039153	TTTKL
282	>ENSMUSG00000050808	TTTQL
283	>ENSMUSG00000037692	TVTSL
284	>ENSMUSG00000037375	TYTVL
285	>ENSMUSG00000030909	VDTSL
286	>ENSMUSG00000032198	VETQL
287	>ENSMUSG00000050405	VETSL
288	>ENSMUSG00000039133	VETTL
289	>ENSMUSG00000021950	VGTDL
290	>ENSMUSG00000034677	VGTEL
291	>ENSMUSG00000024579	VKTEL
292	>ENSMUSG00000033460	VLTKL
293	>ENSMUSG00000001666	VMTFL
294	>ENSMUSG00000037390	VMTSL
295	>ENSMUSG00000001504	VQTYL
296	>ENSMUSG00000079227	VSTGL
297	>ENSMUSG00000027748	VTTRL
298	>ENSMUSG00000041710	VTTRL
299	>ENSMUSG00000027831	VTTYL
300	>ENSMUSG00000078490	VVTGL
301	>ENSMUSG00000044378	WETAL
302	>ENSMUSG00000025995	WLTAL
303	>ENSMUSG00000044340	YDTPL
304	>ENSMUSG00000051951	YETTL
305	>ENSMUSG00000032226	YGTEL
306	>ENSMUSG00000052613	YNTAL
307	>ENSMUSG00000026667	YQTLL
308	>ENSMUSG00000002900	YSTCL
309	>ENSMUSG00000001472	YTTEL

Additional file 4. continued.

mouse xxTxV

No.	gene ID	5aa	No.	gene ID	5aa	No.	gene ID	5aa	No.	gene ID	5aa
1	>ENSMUSG00000070604	AATQV	51	>ENSMUSG00000014773	IATEV	101	>ENSMUSG00000050321	NTTRV	151	>ENSMUSG00000029761	SPTKV
2	>ENSMUSG00000025962	ADTTV	52	>ENSMUSG00000027314	IATEV	102	>ENSMUSG00000000159	NVTLV	152	>ENSMUSG00000031302	STTRV
3	>ENSMUSG00000055489	AKTTV	53	>ENSMUSG00000025658	IETHV	103	>ENSMUSG00000039943	PATVV	153	>ENSMUSG00000051790	STTRV
4	>ENSMUSG00000034773	AMTPV	54	>ENSMUSG00000068037	IETVV	104	>ENSMUSG00000031355	PETLV	154	>ENSMUSG00000063887	STTRV
5	>ENSMUSG00000009214	CCTCV	55	>ENSMUSG00000047959	IFTDV	105	>ENSMUSG00000025161	PETSV	155	>ENSMUSG00000038143	SVTSV
6	>ENSMUSG00000049295	CCTTV	56	>ENSMUSG00000024172	IGTCV	106	>ENSMUSG00000043029	PETSV	156	>ENSMUSG00000002006	SVTTV
7	>ENSMUSG00000056481	CRTSV	57	>ENSMUSG00000015484	ISTDV	107	>ENSMUSG00000024038	PGTLV	157	>ENSMUSG00000035357	SVTTV
8	>ENSMUSG00000034687	DGTEV	58	>ENSMUSG00000041115	ISTVV	108	>ENSMUSG00000026657	PGTLV	158	>ENSMUSG00000032087	SYTLV
9	>ENSMUSG00000048377	DGTEV	59	>ENSMUSG00000045201	ISTVV	109	>ENSMUSG00000030064	PGTLV	159	>ENSMUSG00000050272	SYTLV
10	>ENSMUSG00000051431	DYTDV	60	>ENSMUSG00000032656	KETIV	110	>ENSMUSG00000030868	PLTQV	160	>ENSMUSG00000029370	TETTV
11	>ENSMUSG00000028004	EATNV	61	>ENSMUSG00000021215	KETLV	111	>ENSMUSG00000032641	PNTFV	161	>ENSMUSG00000035027	TRTAV
12	>ENSMUSG00000046034	EETSV	62	>ENSMUSG00000049176	KETTV	112	>ENSMUSG00000046480	PPTKV	162	>ENSMUSG00000073565	TTTTV
13	>ENSMUSG00000025066	EFTGV	63	>ENSMUSG00000038880	KGTPV	113	>ENSMUSG00000078234	PQTPV	163	>ENSMUSG00000038876	TVTEV
14	>ENSMUSG00000073057	EGTCV	64	>ENSMUSG00000029763	KITTV	114	>ENSMUSG00000044098	PSTPV	164	>ENSMUSG00000049907	TVTSV
15	>ENSMUSG00000047861	EGTEV	65	>ENSMUSG00000059455	KNTLV	115	>ENSMUSG00000079346	PVTAV	165	>ENSMUSG00000040929	TYTAV
16	>ENSMUSG00000037579	EGTGV	66	>ENSMUSG00000002266	KPTDV	116	>ENSMUSG00000027958	QDTNV	166	>ENSMUSG00000028804	VCTAV
17	>ENSMUSG00000027422	EGTSV	67	>ENSMUSG00000046961	KPTLV	117	>ENSMUSG00000040479	QETAV	167	>ENSMUSG00000024354	VCTKV
18	>ENSMUSG00000031995	EHTGV	68	>ENSMUSG00000024146	KQTSV	118	>ENSMUSG00000054843	QGTCV	168	>ENSMUSG00000022311	VCTMV
19	>ENSMUSG00000027589	EHTYV	69	>ENSMUSG00000027708	KSTTV	119	>ENSMUSG00000070047	QHTEV	169	>ENSMUSG00000060924	VCTVV
20	>ENSMUSG00000030691	EITLV	70	>ENSMUSG00000046605	KTTTV	120	>ENSMUSG00000038115	QHTNV	170	>ENSMUSG00000038148	VDTRV
21	>ENSMUSG00000054986	ELTPV	71	>ENSMUSG00000040706	KVTTV	121	>ENSMUSG00000013663	QITKV	171	>ENSMUSG00000029032	VETDV
22	>ENSMUSG00000038000	ELTQV	72	>ENSMUSG00000038665	LETAV	122	>ENSMUSG00000041552	QITTV	172	>ENSMUSG00000042604	VETDV
23	>ENSMUSG00000033706	EMTDV	73	>ENSMUSG00000008932	LETNV	123	>ENSMUSG00000029149	QKTWV	173	>ENSMUSG00000015214	VHTSV
24	>ENSMUSG00000040606	EVTNV	74	>ENSMUSG00000036885	LETNV	124	>ENSMUSG00000074505	QQTQV	174	>ENSMUSG00000048826	VMTMV
25	>ENSMUSG00000026126	EWTRV	75	>ENSMUSG00000026463	LETPV	125	>ENSMUSG00000018634	QSTAV	175	>ENSMUSG00000078794	VMTTV
26	>ENSMUSG00000054723	FGTTV	76	>ENSMUSG00000031434	LITRV	126	>ENSMUSG00000035125	QSTGV	176	>ENSMUSG00000031918	VQTVV
27	>ENSMUSG00000016637	FHTLV	77	>ENSMUSG00000047976	LLTDV	127	>ENSMUSG00000063894	QSTGV	177	>ENSMUSG00000009216	VSTDV
28	>ENSMUSG00000018543	FKTDV	78	>ENSMUSG00000044548	LMTTV	128	>ENSMUSG00000064165	RATKV	178	>ENSMUSG00000026959	VSTVV
29	>ENSMUSG00000066842	FPTHV	79	>ENSMUSG00000003166	LNTVV	129	>ENSMUSG00000064165	RATKV	179	>ENSMUSG00000032352	VTTSV
30	>ENSMUSG00000028782	FQTEV	80	>ENSMUSG00000034730	LQTEV	130	>ENSMUSG00000063077	RETTV	180	>ENSMUSG00000016940	WKTLV
31	>ENSMUSG00000033569	FQTEV	81	>ENSMUSG00000029885	LQTGV	131	>ENSMUSG00000028821	RGTAV	181	>ENSMUSG00000042631	YETTV
32	>ENSMUSG00000033383	FRTSV	82	>ENSMUSG00000036915	LQTHV	132	>ENSMUSG00000029730	RITFV	182	>ENSMUSG00000045775	YMTNV
33	>ENSMUSG00000017631	FSTDV	83	>ENSMUSG00000005871	LVTSV	133	>ENSMUSG00000073405	RKTQV	183	>ENSMUSG00000040943	YNTFV
34	>ENSMUSG00000009681	FSTEV	84	>ENSMUSG00000024072	LYTGV	134	>ENSMUSG00000045275	RKTVV	184	>ENSMUSG00000026365	YPTCV
35	>ENSMUSG00000061751	FSTYV	85	>ENSMUSG00000043391	METTV	135	>ENSMUSG00000062209	RNTVV	185	>ENSMUSG00000033898	YPTCV
36	>ENSMUSG00000024068	GDTTV	86	>ENSMUSG00000039384	METVV	136	>ENSMUSG00000034686	RTTAV	186	>ENSMUSG00000027168	YPTVV
37	>ENSMUSG00000041075	GETAV	87	>ENSMUSG00000040724	MLTDV	137	>ENSMUSG00000075558	RTTLV	187	>ENSMUSG00000011831	YSTTV
38	>ENSMUSG00000044674	GETTV	88	>ENSMUSG00000038077	MLTEV	138	>ENSMUSG00000019146	RTTPV			
39	>ENSMUSG00000050288	GETTV	89	>ENSMUSG00000032036	MQTHV	139	>ENSMUSG00000020723	RTTPV			
40	>ENSMUSG00000037957	GGTVV	90	>ENSMUSG00000041734	MQTHV	140	>ENSMUSG00000066189	RTTPV			
41	>ENSMUSG00000022185	GITGV	91	>ENSMUSG00000033389	MSTAV	141	>ENSMUSG00000056867	RVTSV			
42	>ENSMUSG00000022865	GITVV	92	>ENSMUSG00000040552	MSTDV	142	>ENSMUSG00000039372	RVTTV			
43	>ENSMUSG00000043895	GNTVV	93	>ENSMUSG00000038201	MVTEV	143	>ENSMUSG00000040502	RVTTV			
44	>ENSMUSG00000036185	GPTLV	94	>ENSMUSG00000024553	NCTHV	144	>ENSMUSG00000040370	SDTKV			
45	>ENSMUSG00000026548	GPTPV	95	>ENSMUSG00000063434	NCTSV	145	>ENSMUSG00000026556	SETSV			
46	>ENSMUSG00000029101	HATFV	96	>ENSMUSG00000025475	NETTV	146	>ENSMUSG00000027860	SETSV			
47	>ENSMUSG00000046593	HETIV	97	>ENSMUSG00000049791	NETVV	147	>ENSMUSG00000031486	SETTV			
48	>ENSMUSG00000029090	HETTV	98	>ENSMUSG00000032062	NGTLV	148	>ENSMUSG00000029999	SETVV			
49	>ENSMUSG00000026450	HPTPV	99	>ENSMUSG00000078124	NHTCV	149	>ENSMUSG00000030410	SGTVV			
50	>ENSMUSG00000042256	HVTTV	100	>ENSMUSG00000004814	NSTEV	150	>ENSMUSG00000035547	SLTAV			

Additional file 4. continued.

mouse xxVxI

No.	gene ID	5aa	No.	gene ID	5aa
1	>ENSMUSG00000038085	AGVLI	51	>ENSMUSG00000021796	QDVKI
2	>ENSMUSG00000028012	APVRI	52	>ENSMUSG00000050772	QFVKI
3	>ENSMUSG00000043448	ATVWI	53	>ENSMUSG00000047821	QNVFI
4	>ENSMUSG00000075188	CKVCI	54	>ENSMUSG00000036872	QNVKI
5	>ENSMUSG00000037996	CPVSI	55	>ENSMUSG00000024897	QPVYI
6	>ENSMUSG00000073854	CSVHI	56	>ENSMUSG00000041577	QSVVI
7	>ENSMUSG00000021125	ECVRI	57	>ENSMUSG00000024833	QVVRI
8	>ENSMUSG00000000532	EDVKI	58	>ENSMUSG00000070348	RDVDI
9	>ENSMUSG00000061367	ENVQI	59	>ENSMUSG00000057697	RIVNI
10	>ENSMUSG00000036676	ENVSI	60	>ENSMUSG00000059546	RIVNI
11	>ENSMUSG00000001986	ESVKI	61	>ENSMUSG00000060151	RIVNI
12	>ENSMUSG00000033981	ESVKI	62	>ENSMUSG00000061653	RIVNI
13	>ENSMUSG00000040728	EWVCI	63	>ENSMUSG00000067262	RIVNI
14	>ENSMUSG00000048668	FLVKI	64	>ENSMUSG00000068234	RIVNI
15	>ENSMUSG00000026691	FLVLI	65	>ENSMUSG00000075325	RLVDI
16	>ENSMUSG00000079718	FVVKI	66	>ENSMUSG00000022900	RSVVI
17	>ENSMUSG00000079723	FVVKI	67	>ENSMUSG00000024454	SDVEI
18	>ENSMUSG00000020522	HRVSI	68	>ENSMUSG00000060407	SFVPI
19	>ENSMUSG00000004698	IKVII	69	>ENSMUSG00000029620	SKVDI
20	>ENSMUSG00000020385	IPVTI	70	>ENSMUSG00000012076	SQVHI
21	>ENSMUSG00000032718	IYVDI	71	>ENSMUSG00000055691	SSVCI
22	>ENSMUSG00000048796	KKVEI	72	>ENSMUSG00000030741	SSVLI
23	>ENSMUSG00000052310	LFVQI	73	>ENSMUSG00000053964	SYVQI
24	>ENSMUSG00000032035	LGVPI	74	>ENSMUSG00000032066	TFVPI
25	>ENSMUSG00000042808	LKVAI	75	>ENSMUSG00000049491	TRVHI
26	>ENSMUSG00000044976	LSVLI	76	>ENSMUSG00000055961	TSVAI
27	>ENSMUSG00000031111	LTVPI	77	>ENSMUSG00000034520	TSVWI
28	>ENSMUSG00000013076	MEVLI	78	>ENSMUSG00000053693	TYVHI
29	>ENSMUSG00000033653	MSVSI	79	>ENSMUSG00000064010	VPVAI
30	>ENSMUSG00000056832	NRVPI	80	>ENSMUSG00000044938	VPVSI
31	>ENSMUSG00000018924	NSVAI	81	>ENSMUSG00000045052	VSVVI
32	>ENSMUSG00000025701	NSVAI	82	>ENSMUSG00000054920	VTVKI
33	>ENSMUSG00000024505	NSVKI	83	>ENSMUSG00000026866	VVVCI
34	>ENSMUSG00000020891	NSVSI	84	>ENSMUSG00000069601	VYVHI
35	>ENSMUSG00000020892	NSVSI	85	>ENSMUSG00000024066	WSVRI
36	>ENSMUSG00000018907	NSVTI	86	>ENSMUSG00000021922	WTVEI
37	>ENSMUSG00000041000	NTVRI	87	>ENSMUSG00000000632	YEVSI
38	>ENSMUSG00000034640	NTVSI	88	>ENSMUSG00000030683	YEVSI
39	>ENSMUSG00000073076	NYVDI	89	>ENSMUSG00000058153	YEVSI
40	>ENSMUSG00000029638	NYVMI			
41	>ENSMUSG00000041505	PEVDI			
42	>ENSMUSG00000079089	PEVDI			
43	>ENSMUSG00000079090	PEVDI			
44	>ENSMUSG00000079091	PEVDI			
45	>ENSMUSG00000079093	PEVDI			
46	>ENSMUSG00000079094	PEVDI			
47	>ENSMUSG00000052757	PFVSI			
48	>ENSMUSG00000061707	PKVDI			
49	>ENSMUSG00000045994	PSVEI			
50	>ENSMUSG00000046268	PTVYI			

mouse xxVxV

No.	gene ID	5aa	No.	gene ID	5aa
1	>ENSMUSG00000028015	AAVFV	51	>ENSMUSG00000033953	MVVDV
2	>ENSMUSG00000020954	AKVFV	52	>ENSMUSG00000053550	NEVTV
3	>ENSMUSG00000024077	AKVFV	53	>ENSMUSG00000051469	NVVKV
4	>ENSMUSG00000030374	AKVFV	54	>ENSMUSG00000044461	PAVTV
5	>ENSMUSG00000022555	APVGV	55	>ENSMUSG00000023216	PEVSV
6	>ENSMUSG00000026504	APVKV	56	>ENSMUSG00000030924	PGVRV
7	>ENSMUSG00000040447	ASVKV	57	>ENSMUSG00000051703	PPVRV
8	>ENSMUSG00000034452	CPVSV	58	>ENSMUSG00000037486	PVVYV
9	>ENSMUSG00000022718	CTVDV	59	>ENSMUSG00000008686	PYVCV
10	>ENSMUSG00000046318	CVVCV	60	>ENSMUSG00000021294	QEVDV
11	>ENSMUSG00000024846	DCVQV	61	>ENSMUSG00000028876	QGVQV
12	>ENSMUSG00000049521	DEVKV	62	>ENSMUSG00000053442	QIVTV
13	>ENSMUSG00000040021	DLVYV	63	>ENSMUSG00000057722	QKVTV
14	>ENSMUSG00000041921	DSVWV	64	>ENSMUSG00000021959	QPVYV
15	>ENSMUSG00000058886	EKVAV	65	>ENSMUSG00000028664	QSVEV
16	>ENSMUSG00000043319	EKVPV	66	>ENSMUSG00000024137	QTVIV
17	>ENSMUSG00000031220	ELVIV	67	>ENSMUSG00000001323	QTVSV
18	>ENSMUSG00000032515	EPVPV	68	>ENSMUSG00000062300	RAVYV
19	>ENSMUSG00000012889	EQVLV	69	>ENSMUSG00000056856	REVKV
20	>ENSMUSG00000030733	ERVTV	70	>ENSMUSG00000058595	RIVNV
21	>ENSMUSG00000044647	ETVPV	71	>ENSMUSG00000026235	RMVPV
22	>ENSMUSG00000039989	EYVTV	72	>ENSMUSG00000026322	RPVPV
23	>ENSMUSG00000027713	FPVLV	73	>ENSMUSG00000020527	RPVQV
24	>ENSMUSG00000028982	FSVDV	74	>ENSMUSG00000030779	RSVTV
25	>ENSMUSG00000047344	FSVFV	75	>ENSMUSG00000041241	SEVVV
26	>ENSMUSG00000021241	FSVKV	76	>ENSMUSG00000026729	SMVLV
27	>ENSMUSG00000043008	GAVSV	77	>ENSMUSG00000068551	SSVSV
28	>ENSMUSG00000045377	GKVWV	78	>ENSMUSG00000048458	TAVWV
29	>ENSMUSG00000029245	GMVPV	79	>ENSMUSG00000053930	TEVTV
30	>ENSMUSG00000052504	GPVPV	80	>ENSMUSG00000022683	TFVQV
31	>ENSMUSG00000029869	GSVEV	81	>ENSMUSG00000000248	TPVPV
32	>ENSMUSG00000057280	GTVGV	82	>ENSMUSG00000024295	TTVDV
33	>ENSMUSG00000063157	HSVSV	83	>ENSMUSG00000054855	VCVCV
34	>ENSMUSG00000058447	HTVKV	84	>ENSMUSG00000068566	VFVKV
35	>ENSMUSG00000019762	IMVTV	85	>ENSMUSG00000031786	VKVFV
36	>ENSMUSG00000024957	KAVPV	86	>ENSMUSG00000032502	VLVDV
37	>ENSMUSG00000053091	KCVNV	87	>ENSMUSG00000031778	VLVPV
38	>ENSMUSG00000075240	KDVAV	88	>ENSMUSG00000013928	VSVGV
39	>ENSMUSG00000025193	KDVLV	89	>ENSMUSG00000022848	VVVSV
40	>ENSMUSG00000030313	KGVDV	90	>ENSMUSG00000022906	WVVQV
41	>ENSMUSG00000062312	LDVPV	91	>ENSMUSG00000020333	YLVSV
42	>ENSMUSG00000013653	LKVLV	92	>ENSMUSG00000003665	YRVQV
43	>ENSMUSG00000028675	LKVQV	93	>ENSMUSG00000024403	YRVTV
44	>ENSMUSG00000070461	LLVLV			
45	>ENSMUSG00000010830	LPVPV			
46	>ENSMUSG00000005958	LPVQV			
47	>ENSMUSG00000021639	LSVKV			
48	>ENSMUSG00000068173	MAVSV			
49	>ENSMUSG00000038216	MEVQV			
50	>ENSMUSG00000030701	MQVLV			

Additional file 4. continued.

zebrafish xxSxI

No.	gene ID	5aa	No.	gene ID	5aa
1	>ENSDARG00000037409	AFSKI	51	>ENSDARG00000028067	PASSI
2	>ENSDARG00000014498	APSGI	52	>ENSDARG00000031956	PDSDI
3	>ENSDARG00000077850	APSMI	53	>ENSDARG00000068784	PESNI
4	>ENSDARG00000036247	AYSKI	54	>ENSDARG00000020984	PESVI
5	>ENSDARG00000008779	DASFI	55	>ENSDARG00000043175	PFSHI
6	>ENSDARG00000024775	DESEI	56	>ENSDARG00000029177	PGSLI
7	>ENSDARG00000052997	DESEI	57	>ENSDARG00000003584	PHSSI
8	>ENSDARG00000002210	EASAI	58	>ENSDARG00000003573	PLSQI
9	>ENSDARG00000042989	ECSQI	59	>ENSDARG00000061466	PTSTI
10	>ENSDARG00000060649	EDSDI	60	>ENSDARG00000022832	QCSTI
11	>ENSDARG00000075187	EESGI	61	>ENSDARG00000013397	QFSFI
12	>ENSDARG00000075053	EFSVI	62	>ENSDARG00000029100	QGSLI
13	>ENSDARG00000075766	EFSVI	63	>ENSDARG00000019418	RESEI
14	>ENSDARG00000036424	EISNI	64	>ENSDARG00000059822	RESEI
15	>ENSDARG00000061473	ENSKI	65	>ENSDARG00000079670	RESTI
16	>ENSDARG00000060007	ESSEI	66	>ENSDARG00000077734	RESVI
17	>ENSDARG00000062855	ESSQI	67	>ENSDARG00000075327	RHSAI
18	>ENSDARG00000043322	ETSPI	68	>ENSDARG00000060900	RISLI
19	>ENSDARG00000075089	GESTI	69	>ENSDARG00000062231	RISLI
20	>ENSDARG00000004405	GHSVI	70	>ENSDARG00000043466	RRSSI
21	>ENSDARG00000034043	GMSNI	71	>ENSDARG00000029133	RSSNI
22	>ENSDARG00000028746	GVSDI	72	>ENSDARG00000068363	RSSTI
23	>ENSDARG00000041947	HFSII	73	>ENSDARG00000022983	RTSSI
24	>ENSDARG00000036612	ILSLI	74	>ENSDARG00000015230	SKSTI
25	>ENSDARG00000031693	ILSVI	75	>ENSDARG00000015901	SKSTI
26	>ENSDARG00000062618	KESEI	76	>ENSDARG00000019362	SKSTI
27	>ENSDARG00000007086	KMSSI	77	>ENSDARG00000031907	SKSTI
28	>ENSDARG00000036457	KNSDI	78	>ENSDARG00000043757	SKSTI
29	>ENSDARG00000017107	KSSDI	79	>ENSDARG00000069374	SPSRI
30	>ENSDARG00000040435	KSSTI	80	>ENSDARG00000035379	SRSLI
31	>ENSDARG00000015537	LASNI	81	>ENSDARG00000007441	SSSGI
32	>ENSDARG00000070116	LDSKI	82	>ENSDARG00000067758	SVSDI
33	>ENSDARG00000017720	LESRI	83	>ENSDARG00000078703	TLSPI
34	>ENSDARG00000024160	LISRI	84	>ENSDARG00000045973	TPSKI
35	>ENSDARG00000079556	LLSGI	85	>ENSDARG00000069974	TPSPI
36	>ENSDARG00000015902	LLSSI	86	>ENSDARG00000045401	TSSTI
37	>ENSDARG00000076040	LPSRI	87	>ENSDARG00000059583	VKSLI
38	>ENSDARG00000069368	LYSWI	88	>ENSDARG00000011257	YESEI
39	>ENSDARG00000068296	MESAI	89	>ENSDARG00000075384	YESEI
40	>ENSDARG00000070371	MPSLI	90	>ENSDARG00000079507	YESEI
41	>ENSDARG00000070375	MPSLI	91	>ENSDARG00000071235	YESVI
42	>ENSDARG00000015905	MSSDI			
43	>ENSDARG00000074197	NASAI			
44	>ENSDARG00000068010	NESAI			
45	>ENSDARG00000068825	NESAI			
46	>ENSDARG00000009939	NGSPI			
47	>ENSDARG00000077019	NISLI			
48	>ENSDARG00000077827	NMSLI			
49	>ENSDARG00000019033	NQSSI			
50	>ENSDARG00000062702	NYSFI			

Additional file 4. continued.

zebrafish xxSxL

No.	gene ID	5aa	No.	gene ID	5aa	No.	gene ID	5aa	No.	gene ID	5aa	No.	gene ID	5aa
1	>ENSDARG00000010936	AASTL	51	>ENSDARG00000003380	FSSKL	101	>ENSDARG00000061042	MVSGL	151	>ENSDARG00000008068	SDSDL	201	>ENSDARG00000043410	YESSL
2	>ENSDARG00000009443	ADSKL	52	>ENSDARG00000078824	FTSIL	102	>ENSDARG00000017199	NLSAL	152	>ENSDARG00000053570	SDSRL	202	>ENSDARG00000074757	YLSML
3	>ENSDARG00000013856	ADSKL	53	>ENSDARG00000063719	FYSSL	103	>ENSDARG00000077537	NNSKL	153	>ENSDARG00000076528	SFSSL	203	>ENSDARG00000058319	YRSVL
4	>ENSDARG00000030357	ADSKL	54	>ENSDARG00000069597	GASKL	104	>ENSDARG00000014287	NPSAL	154	>ENSDARG00000061461	SHSFL	204	>ENSDARG00000037904	YVSVL
5	>ENSDARG00000001437	ADSQL	55	>ENSDARG00000021309	GCSLL	105	>ENSDARG00000069048	NPSDL	155	>ENSDARG00000069055	SHSVL			
6	>ENSDARG00000007412	ADSQL	56	>ENSDARG00000001431	GESDL	106	>ENSDARG00000058562	NQSDL	156	>ENSDARG00000069059	SHSVL			
7	>ENSDARG00000028583	AESDL	57	>ENSDARG00000007219	GESDL	107	>ENSDARG00000004415	NTSSL	157	>ENSDARG00000038876	SKSKL			
8	>ENSDARG00000070973	AESNL	58	>ENSDARG00000013755	GESDL	108	>ENSDARG00000034211	NVSML	158	>ENSDARG00000055591	SKSSL			
9	>ENSDARG00000062346	AGSAL	59	>ENSDARG00000071090	GESDL	109	>ENSDARG00000075867	NWSSL	159	>ENSDARG00000074666	SLSCL			
10	>ENSDARG00000042642	AGSVL	60	>ENSDARG00000071086	GESFL	110	>ENSDARG00000057056	PDSCL	160	>ENSDARG00000057223	SLSSL			
11	>ENSDARG00000034211	AISRL	61	>ENSDARG00000045461	GKSKL	111	>ENSDARG00000062821	PESDL	161	>ENSDARG00000041952	SPSYL			
12	>ENSDARG00000035601	AKSRL	62	>ENSDARG00000043638	GRSQL	112	>ENSDARG00000045051	PESSL	162	>ENSDARG00000014138	SQSKL			
13	>ENSDARG00000035602	AKSRL	63	>ENSDARG00000071168	HCSQL	113	>ENSDARG00000002877	PFSHL	163	>ENSDARG00000061917	SQSKL			
14	>ENSDARG00000035603	AKSRL	64	>ENSDARG00000034877	HSSKL	114	>ENSDARG00000062418	PFSKL	164	>ENSDARG00000053463	SRSEL			
15	>ENSDARG00000006251	AQSWL	65	>ENSDARG00000009123	IDSLL	115	>ENSDARG00000043323	PGSLL	165	>ENSDARG00000078101	SRSVL			
16	>ENSDARG00000015717	ATSNL	66	>ENSDARG00000012915	IFSKL	116	>ENSDARG00000059438	PGSTL	166	>ENSDARG00000018958	SSSAL			
17	>ENSDARG00000043006	AVSLL	67	>ENSDARG00000032959	IPSIL	117	>ENSDARG00000036499	PHSEL	167	>ENSDARG00000052343	SSSRL			
18	>ENSDARG00000058673	AYSKL	68	>ENSDARG00000074895	IQSKL	118	>ENSDARG00000029609	PKSKL	168	>ENSDARG00000004445	SSSSL			
19	>ENSDARG00000054418	CCSIL	69	>ENSDARG00000019260	KASIL	119	>ENSDARG00000017606	PKSNL	169	>ENSDARG00000041162	SSSTL			
20	>ENSDARG00000054827	CCSIL	70	>ENSDARG00000077311	KCSLL	120	>ENSDARG00000079571	PRSKL	170	>ENSDARG00000023797	SSSVL			
21	>ENSDARG00000037009	CDSFL	71	>ENSDARG00000078559	KCSLL	121	>ENSDARG00000042510	PRSML	171	>ENSDARG00000023759	STSTL			
22	>ENSDARG00000042270	CNSAL	72	>ENSDARG00000039100	KDSDL	122	>ENSDARG00000062686	PSSLL	172	>ENSDARG00000057029	SVSCL			
23	>ENSDARG00000071496	CNSDL	73	>ENSDARG00000017389	KDSEL	123	>ENSDARG00000074980	PVSNL	173	>ENSDARG00000058585	TASKL			
24	>ENSDARG00000034643	CNSTL	74	>ENSDARG00000011188	KESSL	124	>ENSDARG00000031981	QASVL	174	>ENSDARG00000037646	TASRL			
25	>ENSDARG00000057724	CSSNL	75	>ENSDARG00000079944	KESSL	125	>ENSDARG00000030129	QDSSL	175	>ENSDARG00000054746	THSEL			
26	>ENSDARG00000073844	CVSDL	76	>ENSDARG00000056101	KISAL	126	>ENSDARG00000030656	QESNL	176	>ENSDARG00000008057	THSTL			
27	>ENSDARG00000031983	DGSDL	77	>ENSDARG00000074746	KISSL	127	>ENSDARG00000027992	QFSRL	177	>ENSDARG00000055976	TKSKL			
28	>ENSDARG00000062573	DGSGL	78	>ENSDARG00000007354	KKSEL	128	>ENSDARG00000003757	QHSEL	178	>ENSDARG00000045663	TNSGL			
29	>ENSDARG00000052011	DLSVL	79	>ENSDARG00000073820	KTSKL	129	>ENSDARG00000060522	QHSLL	179	>ENSDARG00000062909	TQSAL			
30	>ENSDARG00000039497	DWSLL	80	>ENSDARG00000076213	KVSQL	130	>ENSDARG00000059052	QHSSL	180	>ENSDARG00000040352	TQSQL			
31	>ENSDARG00000062460	EDSFL	81	>ENSDARG00000079157	KVSQL	131	>ENSDARG00000059741	QNSAL	181	>ENSDARG00000070971	TQSVL			
32	>ENSDARG00000070494	EDSFL	82	>ENSDARG00000029975	KYSSL	132	>ENSDARG00000057352	QPSSL	182	>ENSDARG00000020529	TRSNL			
33	>ENSDARG00000000588	EESKL	83	>ENSDARG00000005476	LESAL	133	>ENSDARG00000079452	QSSFL	183	>ENSDARG00000042747	TRSSL			
34	>ENSDARG00000014477	EISYL	84	>ENSDARG00000074673	LESSL	134	>ENSDARG00000027131	QTSNL	184	>ENSDARG00000004150	TTSSL			
35	>ENSDARG00000008100	EKSEL	85	>ENSDARG00000055613	LESTL	135	>ENSDARG00000032340	QTSRL	185	>ENSDARG00000031712	TTSSL			
36	>ENSDARG00000052375	EKSQL	86	>ENSDARG00000073672	LESTL	136	>ENSDARG00000017338	RESIL	186	>ENSDARG00000053829	VASAL			
37	>ENSDARG00000009021	ELSML	87	>ENSDARG00000000068	LFSNL	137	>ENSDARG00000058569	RESIL	187	>ENSDARG00000039830	VCSFL			
38	>ENSDARG00000007795	ENSCL	88	>ENSDARG00000074042	LFSNL	138	>ENSDARG00000078833	RESLL	188	>ENSDARG00000005463	VESSL			
39	>ENSDARG00000069192	ENSCL	89	>ENSDARG00000012453	LKSSL	139	>ENSDARG00000028099	RHSEL	189	>ENSDARG00000000551	VESVL			
40	>ENSDARG00000045561	EYSGL	90	>ENSDARG00000041853	LLSFL	140	>ENSDARG00000006899	RHSRL	190	>ENSDARG00000031749	VHSVL			
41	>ENSDARG00000061936	FCSIL	91	>ENSDARG00000014403	LQSWL	141	>ENSDARG00000003069	RKSAL	191	>ENSDARG00000004896	VLSKL			
42	>ENSDARG00000013371	FFSSL	92	>ENSDARG00000014727	LRSKL	142	>ENSDARG00000070387	RLSKL	192	>ENSDARG00000044751	VMSFL			
43	>ENSDARG00000071303	FLSLL	93	>ENSDARG00000061665	LSSNL	143	>ENSDARG00000068779	RNSKL	193	>ENSDARG00000054766	VNSLL			
44	>ENSDARG00000051983	FLSQL	94	>ENSDARG00000025522	MDSYL	144	>ENSDARG00000023724	RNSLL	194	>ENSDARG00000079981	VPSKL			
45	>ENSDARG00000075395	FLSQL	95	>ENSDARG00000018726	MESVL	145	>ENSDARG00000044540	RRSDL	195	>ENSDARG00000026762	VTSDL			
46	>ENSDARG00000070413	FLSSL	96	>ENSDARG00000070029	MHSHL	146	>ENSDARG00000004539	RSSEL	196	>ENSDARG00000076229	WDSCL			
47	>ENSDARG00000077407	FLSSL	97	>ENSDARG00000042562	MISKL	147	>ENSDARG00000010276	RTSEL	197	>ENSDARG00000051746	WESLL			
48	>ENSDARG00000044807	FLSTL	98	>ENSDARG00000002523	MKSKL	148	>ENSDARG00000032799	RVSAL	198	>ENSDARG00000055291	YCSLL			
49	>ENSDARG00000068166	FNSAL	99	>ENSDARG00000021135	MNSRL	149	>ENSDARG00000040141	RVSVL	199	>ENSDARG00000078530	YDSDL			
50	>ENSDARG00000078745	FRSKL	100	>ENSDARG00000070433	MSSWL	150	>ENSDARG00000056723	SCSAL	200	>ENSDARG00000020443	YESSL			

Additional file 4. continued.

zebrafish xxSxV

No.	gene ID	5aa	No.	gene ID	5aa	No.	gene ID	5aa	No.	gene ID	5aa
1	>ENSDARG00000077799	AFSLV	51	>ENSDARG00000070543	IESDV	101	>ENSDARG00000062415	PDSWV	151	>ENSDARG00000078931	TCSSV
2	>ENSDARG00000076639	AGSLV	52	>ENSDARG00000075341	IISDV	102	>ENSDARG00000071478	PGSLV	152	>ENSDARG00000057630	TFSVV
3	>ENSDARG00000077039	AGSLV	53	>ENSDARG00000059324	IISQV	103	>ENSDARG00000045444	PLSQV	153	>ENSDARG00000075666	TGSAV
4	>ENSDARG00000028012	AKSGV	54	>ENSDARG00000079040	IPSPV	104	>ENSDARG00000038569	PLSRV	154	>ENSDARG00000079898	TKSHV
5	>ENSDARG00000055974	AKSGV	55	>ENSDARG00000079660	IRSHV	105	>ENSDARG00000059154	PRSLV	155	>ENSDARG00000060400	TPSDV
6	>ENSDARG00000054191	ALSNV	56	>ENSDARG00000007578	ISSEV	106	>ENSDARG00000069829	PRSTV	156	>ENSDARG00000062934	TPSDV
7	>ENSDARG00000059883	ASSSV	57	>ENSDARG00000061494	IVSQV	107	>ENSDARG00000076442	PSSDV	157	>ENSDARG00000070023	TPSDV
8	>ENSDARG00000042552	ATSAV	58	>ENSDARG00000079713	KASAV	108	>ENSDARG00000004452	PSSHV	158	>ENSDARG00000063357	TQSSV
9	>ENSDARG00000032984	ATSSV	59	>ENSDARG00000074414	KDSIV	109	>ENSDARG00000025420	PSSHV	159	>ENSDARG00000029204	TQSVV
10	>ENSDARG00000042344	CDSSV	60	>ENSDARG00000076381	KDSPV	110	>ENSDARG00000030064	PVSEV	160	>ENSDARG00000058957	TRSLV
11	>ENSDARG00000001686	CSSEV	61	>ENSDARG00000080000	KDSPV	111	>ENSDARG00000002240	QFSTV	161	>ENSDARG00000036560	TSSDV
12	>ENSDARG00000020236	DDSNV	62	>ENSDARG00000027461	KDSVV	112	>ENSDARG00000061459	QISAV	162	>ENSDARG00000079069	TSSVV
13	>ENSDARG00000076753	DDSNV	63	>ENSDARG00000008797	KESIV	113	>ENSDARG00000075206	QNSSV	163	>ENSDARG00000070571	VASQV
14	>ENSDARG00000061535	DESHV	64	>ENSDARG00000062517	KESTV	114	>ENSDARG00000003989	QSSAV	164	>ENSDARG00000052723	VCSNV
15	>ENSDARG00000076595	DESSV	65	>ENSDARG00000043475	KESYV	115	>ENSDARG00000063037	QSSVV	165	>ENSDARG00000061688	VDSWV
16	>ENSDARG00000028878	DFSAV	66	>ENSDARG00000063040	KGSIV	116	>ENSDARG00000078762	QTSSV	166	>ENSDARG00000079004	VESNV
17	>ENSDARG00000019659	DGSEV	67	>ENSDARG00000063255	KGSIV	117	>ENSDARG00000035352	RASLV	167	>ENSDARG00000062531	VLSLV
18	>ENSDARG00000043658	DGSIV	68	>ENSDARG00000037069	KLSSV	118	>ENSDARG00000034588	RESIV	168	>ENSDARG00000010565	VLSSV
19	>ENSDARG00000074636	DGSMV	69	>ENSDARG00000021241	KRSFV	119	>ENSDARG00000020758	RESSV	169	>ENSDARG00000010717	VTSLV
20	>ENSDARG00000061676	DHSPV	70	>ENSDARG00000061981	LASDV	120	>ENSDARG00000079306	RESVV	170	>ENSDARG00000077422	VTSRV
21	>ENSDARG00000008637	DHSQV	71	>ENSDARG00000079800	LCSIV	121	>ENSDARG00000002353	RESYV	171	>ENSDARG00000077559	VWSFV
22	>ENSDARG00000044213	DHSVV	72	>ENSDARG00000034493	LESDV	122	>ENSDARG00000070927	RFSSV	172	>ENSDARG00000075774	WDSIV
23	>ENSDARG00000053344	DLSAV	73	>ENSDARG00000045006	LESLV	123	>ENSDARG00000014697	RISNV	173	>ENSDARG00000061370	WSSMV
24	>ENSDARG00000017422	DLSKV	74	>ENSDARG00000051814	LESLV	124	>ENSDARG00000024189	RQSEV	174	>ENSDARG00000035084	YCSLV
25	>ENSDARG00000005213	DPSRV	75	>ENSDARG00000056690	LHSSV	125	>ENSDARG00000011955	RRSQV	175	>ENSDARG00000077021	YYSKV
26	>ENSDARG00000075325	DPSRV	76	>ENSDARG00000016145	LISQV	126	>ENSDARG00000052898	RRSSV			
27	>ENSDARG00000074369	DSSEV	77	>ENSDARG00000060587	LISTV	127	>ENSDARG00000079645	RTSPV			
28	>ENSDARG00000074677	DSSEV	78	>ENSDARG00000046087	LLSDV	128	>ENSDARG00000016311	SASTV			
29	>ENSDARG00000076856	DSSEV	79	>ENSDARG00000060069	LLSKV	129	>ENSDARG00000074661	SESEV			
30	>ENSDARG00000013117	EDSSV	80	>ENSDARG00000059130	LPSTV	130	>ENSDARG00000011863	SESKV			
31	>ENSDARG00000010023	EDSTV	81	>ENSDARG00000031345	LQSQV	131	>ENSDARG00000058985	SESKV			
32	>ENSDARG00000079611	EESSV	82	>ENSDARG00000035895	LRSKV	132	>ENSDARG00000041533	SGSEV			
33	>ENSDARG00000055926	EGSEV	83	>ENSDARG00000076321	LSSTV	133	>ENSDARG00000011555	SGSSV			
34	>ENSDARG00000063704	ENSTV	84	>ENSDARG00000063525	LTSEV	134	>ENSDARG00000077856	SGSSV			
35	>ENSDARG00000055722	FNSSV	85	>ENSDARG00000013244	MESLV	135	>ENSDARG00000010728	SLSVV			
36	>ENSDARG00000062854	FSSFV	86	>ENSDARG00000060459	MESTV	136	>ENSDARG00000056151	SQSVV			
37	>ENSDARG00000078116	FSSFV	87	>ENSDARG00000077489	MESTV	137	>ENSDARG00000052289	SSSAV			
38	>ENSDARG00000078866	FSSFV	88	>ENSDARG00000075619	MGSIV	138	>ENSDARG00000070148	SSSAV			
39	>ENSDARG00000075887	GFSLV	89	>ENSDARG00000002576	MLSSV	139	>ENSDARG00000063484	SSSIV			
40	>ENSDARG00000052244	GSSNV	90	>ENSDARG00000006396	MNSFV	140	>ENSDARG00000074105	SSSSV			
41	>ENSDARG00000077431	GTSAV	91	>ENSDARG00000012460	NASYV	141	>ENSDARG00000074295	SSSSV			
42	>ENSDARG00000057143	GVSVV	92	>ENSDARG00000029832	NDSSV	142	>ENSDARG00000076304	SSSSV			
43	>ENSDARG00000075687	HDSGV	93	>ENSDARG00000062552	NESHV	143	>ENSDARG00000076384	SSSSV			
44	>ENSDARG00000052099	HKSRV	94	>ENSDARG00000039673	NGSIV	144	>ENSDARG00000076397	SSSSV			
45	>ENSDARG00000007080	HRSHV	95	>ENSDARG00000019920	NISVV	145	>ENSDARG00000077127	SSSSV			
46	>ENSDARG00000074540	HRSVV	96	>ENSDARG00000040257	NTSIV	146	>ENSDARG00000077280	SSSSV			
47	>ENSDARG00000035820	HSSSV	97	>ENSDARG00000009121	PCSTV	147	>ENSDARG00000078946	SSSSV			
48	>ENSDARG00000018228	HTSSV	98	>ENSDARG00000074013	PCSTV	148	>ENSDARG00000079607	SSSSV			
49	>ENSDARG00000070940	HVSHV	99	>ENSDARG00000003779	PDSWV	149	>ENSDARG00000056847	TASEV			
50	>ENSDARG00000030376	IESDV	100	>ENSDARG00000045331	PDSWV	150	>ENSDARG00000077326	TASSV			

Additional file 4. continued.

zebrafish xxTxI

No.	gene ID	5aa	No.	gene ID	5aa
1	>ENSDARG00000077207	AATEI	51	>ENSDARG00000006368	QYTRI
2	>ENSDARG00000021439	AATQI	52	>ENSDARG00000000837	QYTTI
3	>ENSDARG00000009626	AITTI	53	>ENSDARG00000057184	RATPI
4	>ENSDARG00000038219	AITYI	54	>ENSDARG00000036117	RCTVI
5	>ENSDARG00000036156	AMTYI	55	>ENSDARG00000055302	RGTSI
6	>ENSDARG00000020131	AVTYI	56	>ENSDARG00000039052	RLTKI
7	>ENSDARG00000052419	DLTPI	57	>ENSDARG00000032896	RSTAI
8	>ENSDARG00000005162	DMTSI	58	>ENSDARG00000069415	RSTVI
9	>ENSDARG00000033683	DMTSI	59	>ENSDARG00000052792	SDTHI
10	>ENSDARG00000023587	DNTDI	60	>ENSDARG00000012369	SKTGI
11	>ENSDARG00000060430	DRTGI	61	>ENSDARG00000020132	SLTEI
12	>ENSDARG00000044655	EITGI	62	>ENSDARG00000036442	SNTVI
13	>ENSDARG00000068892	ELTLI	63	>ENSDARG00000077897	SQTTI
14	>ENSDARG00000056029	ESTHI	64	>ENSDARG00000054033	SSTAI
15	>ENSDARG00000013842	FATSI	65	>ENSDARG00000059209	SVTII
16	>ENSDARG00000013782	FLTRI	66	>ENSDARG00000074597	TATEI
17	>ENSDARG00000003181	FRTEI	67	>ENSDARG00000038646	TDTVI
18	>ENSDARG00000028275	FRTEI	68	>ENSDARG00000061974	TLTEI
19	>ENSDARG00000041540	FRTEI	69	>ENSDARG00000069748	TLTNI
20	>ENSDARG00000079079	FRTKI	70	>ENSDARG00000019478	TLTPI
21	>ENSDARG00000035607	GDTPI	71	>ENSDARG00000054510	TVTSI
22	>ENSDARG00000031587	HETTI	72	>ENSDARG00000015033	VCTKI
23	>ENSDARG00000074583	HGTSI	73	>ENSDARG00000069717	VLTNI
24	>ENSDARG00000070919	HHTQI	74	>ENSDARG00000036179	YVTKI
25	>ENSDARG00000044161	HSTSI			
26	>ENSDARG00000077725	HYTSI			
27	>ENSDARG00000003977	ILTLI			
28	>ENSDARG00000012040	IQTEI			
29	>ENSDARG00000044528	KQTKI			
30	>ENSDARG00000077192	KQTKI			
31	>ENSDARG00000023362	KRTCI			
32	>ENSDARG00000025189	LQTQI			
33	>ENSDARG00000037926	LQTQI			
34	>ENSDARG00000059090	LQTTI			
35	>ENSDARG00000057227	LSTLI			
36	>ENSDARG00000055592	NLTMI			
37	>ENSDARG00000076868	PETKI			
38	>ENSDARG00000079542	PETKI			
39	>ENSDARG00000008131	PLTHI			
40	>ENSDARG00000010770	PLTHI			
41	>ENSDARG00000040266	PLTHI			
42	>ENSDARG00000053569	PLTHI			
43	>ENSDARG00000069866	PLTHI			
44	>ENSDARG00000074433	PMTFI			
45	>ENSDARG00000003020	QETQI			
46	>ENSDARG00000014792	QETQI			
47	>ENSDARG00000037493	QETQI			
48	>ENSDARG00000069402	QETQI			
49	>ENSDARG00000078370	QETQI			
50	>ENSDARG00000060181	QETTI			

Additional file 4. continued.

zebrafish xxTxL

No.	gene ID	5aa	No.	gene ID	5aa	No.	gene ID	5aa	No.	gene ID	5aa	No.	gene ID	5aa
1	>ENSDARG00000021137	ADTHL	51	>ENSDARG00000032157	EPTRL	101	>ENSDARG00000054718	KMTSL	151	>ENSDARG00000051957	QHTDL	201	>ENSDARG00000074146	VHTGL
2	>ENSDARG00000040851	APTLL	52	>ENSDARG00000052380	FCTIL	102	>ENSDARG00000021859	KNTEL	152	>ENSDARG00000079060	QPTTL	202	>ENSDARG00000010434	VITKL
3	>ENSDARG00000012823	AQTRL	53	>ENSDARG00000078664	FCTIL	103	>ENSDARG00000078560	KPTQL	153	>ENSDARG00000074829	QRTRL	203	>ENSDARG00000045947	VNTFL
4	>ENSDARG00000014280	AQTRL	54	>ENSDARG00000042970	FCTVL	104	>ENSDARG00000069497	KRTEL	154	>ENSDARG00000008127	QSTVL	204	>ENSDARG00000029866	VSTRL
5	>ENSDARG00000055459	AQTRL	55	>ENSDARG00000014571	FDTDL	105	>ENSDARG00000003811	KVTDL	155	>ENSDARG00000031796	RCTDL	205	>ENSDARG00000070504	VTTQL
6	>ENSDARG00000059340	AQTRL	56	>ENSDARG00000023472	FDTDL	106	>ENSDARG00000017211	KVTDL	156	>ENSDARG00000013161	RCTLL	206	>ENSDARG00000035606	WCTIL
7	>ENSDARG00000060849	AQTRL	57	>ENSDARG00000058953	FETAL	107	>ENSDARG00000032990	KVTDL	157	>ENSDARG00000041425	RCTLL	207	>ENSDARG00000043154	WHTPL
8	>ENSDARG00000076070	AQTRL	58	>ENSDARG00000058000	FKTTL	108	>ENSDARG00000074417	KVTDL	158	>ENSDARG00000007278	RETLL	208	>ENSDARG00000014973	WITVL
9	>ENSDARG00000063177	ARTDL	59	>ENSDARG00000067719	FLTWL	109	>ENSDARG00000052376	KYTPL	159	>ENSDARG00000007077	RETPL	209	>ENSDARG00000078694	YDTPL
10	>ENSDARG00000073940	ARTDL	60	>ENSDARG00000068401	FLTWL	110	>ENSDARG00000043474	LETCL	160	>ENSDARG00000041644	RFTKL	210	>ENSDARG00000019080	YETTL
11	>ENSDARG00000019405	ARTSL	61	>ENSDARG00000076614	FRTAL	111	>ENSDARG00000070672	LETKL	161	>ENSDARG00000040736	RITDL	211	>ENSDARG00000077497	YMTIL
12	>ENSDARG00000023578	ATTDL	62	>ENSDARG00000017190	FRTVL	112	>ENSDARG00000018418	LETML	162	>ENSDARG00000015930	RKTAL	212	>ENSDARG00000030098	YVTGL
13	>ENSDARG00000031095	ATTYL	63	>ENSDARG00000031051	FTTGL	113	>ENSDARG00000007788	LETSL	163	>ENSDARG00000075754	RLTAL			
14	>ENSDARG00000024004	CITTL	64	>ENSDARG00000071288	GETKL	114	>ENSDARG00000012684	LETSL	164	>ENSDARG00000045172	RNTEL			
15	>ENSDARG00000061850	CLTDL	65	>ENSDARG00000004470	GHTEL	115	>ENSDARG00000063433	LETSL	165	>ENSDARG00000058208	RRTLL			
16	>ENSDARG00000053580	CSTVL	66	>ENSDARG00000041379	GHTVL	116	>ENSDARG00000074366	LITSL	166	>ENSDARG00000059166	RSTKL			
17	>ENSDARG00000008398	CVTSL	67	>ENSDARG00000075113	GITLL	117	>ENSDARG00000070480	LKTEL	167	>ENSDARG00000003049	RTTTL			
18	>ENSDARG00000042717	DDTLL	68	>ENSDARG00000063447	GLTAL	118	>ENSDARG00000074207	LKTEL	168	>ENSDARG00000067925	RTTTL			
19	>ENSDARG00000075683	DETHL	69	>ENSDARG00000053835	GPTLL	119	>ENSDARG00000060792	LLTGL	169	>ENSDARG00000046013	SATAL			
20	>ENSDARG00000079096	DETHL	70	>ENSDARG00000036237	GRTRL	120	>ENSDARG00000059360	LLTTL	170	>ENSDARG00000055534	SCTLL			
21	>ENSDARG00000006299	DETNL	71	>ENSDARG00000031533	GYTVL	121	>ENSDARG00000039616	LPTDL	171	>ENSDARG00000078758	SETFL			
22	>ENSDARG00000069715	DGTEL	72	>ENSDARG00000054389	HCTVL	122	>ENSDARG00000069806	LQTSL	172	>ENSDARG00000054127	SGTRL			
23	>ENSDARG00000070338	DITRL	73	>ENSDARG00000036722	HHTRL	123	>ENSDARG00000040001	LSTGL	173	>ENSDARG00000003655	SKTSL			
24	>ENSDARG00000036433	DKTEL	74	>ENSDARG00000040644	HQTVL	124	>ENSDARG00000032324	LTTEL	174	>ENSDARG00000012051	SNTEL			
25	>ENSDARG00000056007	DLTEL	75	>ENSDARG00000053961	HSTAL	125	>ENSDARG00000039187	LTTEL	175	>ENSDARG00000038040	SQTFL			
26	>ENSDARG00000017812	DLTGL	76	>ENSDARG00000063666	HVTEL	126	>ENSDARG00000079886	LTTPL	176	>ENSDARG00000041554	SQTQL			
27	>ENSDARG00000030173	DLTGL	77	>ENSDARG00000059545	IETLL	127	>ENSDARG00000018436	LVTSL	177	>ENSDARG00000077875	SSTFL			
28	>ENSDARG00000020057	DMTCL	78	>ENSDARG00000053884	IMTML	128	>ENSDARG00000061121	LVTSL	178	>ENSDARG00000037324	SSTSL			
29	>ENSDARG00000023963	DMTSL	79	>ENSDARG00000071579	IQTKL	129	>ENSDARG00000069356	LVTSL	179	>ENSDARG00000079383	STTTL			
30	>ENSDARG00000068385	DMTTL	80	>ENSDARG00000046091	IQTNL	130	>ENSDARG00000033450	MNTLL	180	>ENSDARG00000058219	SYTHL			
31	>ENSDARG00000003925	DNTAL	81	>ENSDARG00000078415	ISTVL	131	>ENSDARG00000042057	MWTGL	181	>ENSDARG00000036864	TATHL			
32	>ENSDARG00000077844	DRTFL	82	>ENSDARG00000077104	ITTDL	132	>ENSDARG00000063309	NDTEL	182	>ENSDARG00000060366	TCTEL			
33	>ENSDARG00000044406	DVTFL	83	>ENSDARG00000012903	KATSL	133	>ENSDARG00000035889	NGTLL	183	>ENSDARG00000020602	TCTLL			
34	>ENSDARG00000036546	EATVL	84	>ENSDARG00000074713	KATSL	134	>ENSDARG00000028541	NLTTL	184	>ENSDARG00000068932	TCTVL			
35	>ENSDARG00000070653	EATVL	85	>ENSDARG00000070822	KCTIL	135	>ENSDARG00000036212	NPTDL	185	>ENSDARG00000029482	TDTHL			
36	>ENSDARG00000012504	EDTAL	86	>ENSDARG00000062873	KCTKL	136	>ENSDARG00000074923	NTTAL	186	>ENSDARG00000041375	TDTNL			
37	>ENSDARG00000031228	EDTHL	87	>ENSDARG00000036846	KDTFL	137	>ENSDARG00000002909	PATEL	187	>ENSDARG00000061103	TETAL			
38	>ENSDARG00000075004	EDTYL	88	>ENSDARG00000015240	KETAL	138	>ENSDARG00000003375	PCTIL	188	>ENSDARG00000068613	TETSL			
39	>ENSDARG00000077871	EDTYL	89	>ENSDARG00000042021	KETAL	139	>ENSDARG00000006553	PCTLL	189	>ENSDARG00000073952	TETSL			
40	>ENSDARG00000062757	EITAL	90	>ENSDARG00000071868	KETLL	140	>ENSDARG00000014003	PETKL	190	>ENSDARG00000079074	TLTAL			
41	>ENSDARG00000054778	EITDL	91	>ENSDARG00000045666	KETPL	141	>ENSDARG00000070512	PKTEL	191	>ENSDARG00000005355	TQTCL			
42	>ENSDARG00000007561	EITEL	92	>ENSDARG00000006409	KETSL	142	>ENSDARG00000075998	PMTKL	192	>ENSDARG00000078579	TQTKL			
43	>ENSDARG00000020979	EITIL	93	>ENSDARG00000014916	KGTKL	143	>ENSDARG00000033437	PSTSL	193	>ENSDARG00000035556	TSTAL			
44	>ENSDARG00000078474	EITML	94	>ENSDARG00000045071	KGTRL	144	>ENSDARG00000062357	PYTSL	194	>ENSDARG00000028469	TSTRL			
45	>ENSDARG00000060754	EITSL	95	>ENSDARG00000016981	KHTEL	145	>ENSDARG00000023443	QDTEL	195	>ENSDARG00000068878	TSTYL			
46	>ENSDARG00000059276	EITTL	96	>ENSDARG00000003697	KITKL	146	>ENSDARG00000041107	QDTRL	196	>ENSDARG00000079190	TTTDL			
47	>ENSDARG00000001214	ELTIL	97	>ENSDARG00000029263	KLTHL	147	>ENSDARG00000052866	QDTSL	197	>ENSDARG00000079229	TYTQL			
48	>ENSDARG00000076409	ELTIL	98	>ENSDARG00000036231	KLTHL	148	>ENSDARG00000060096	QDTYL	198	>ENSDARG00000060034	VETSL			
49	>ENSDARG00000004695	EMTDL	99	>ENSDARG00000059280	KLTHL	149	>ENSDARG00000042859	QETAL	199	>ENSDARG00000079878	VETSL			
50	>ENSDARG00000005448	ENTSL	100	>ENSDARG00000003684	KLTVL	150	>ENSDARG00000076388	QETDL	200	>ENSDARG00000012764	VHTCL			

Additional file 4. continued.

zebrafish xxTxV

No.	gene ID	5aa	No.	gene ID	5aa	No.	gene ID	5aa	No.	gene ID	5aa	No.	gene ID	5aa
1	>ENSDARG00000044415	ADTAV	51	>ENSDARG00000061664	IETTV	101	>ENSDARG00000058071	METSV	151	>ENSDARG00000075755	RITPV	201	>ENSDARG00000052322	TNTAV
2	>ENSDARG00000058042	ADTEV	52	>ENSDARG00000036043	IETVV	102	>ENSDARG00000063282	MFTDV	152	>ENSDARG00000052045	RKTSV	202	>ENSDARG00000002830	TNTDV
3	>ENSDARG00000041332	APTGV	53	>ENSDARG00000079491	IFTDV	103	>ENSDARG00000002241	MLTDV	153	>ENSDARG00000054734	RKTSV	203	>ENSDARG00000075358	TSTTV
4	>ENSDARG00000014673	CPTHV	54	>ENSDARG00000040860	IITPV	104	>ENSDARG00000013477	MPTLV	154	>ENSDARG00000017108	RLTDV	204	>ENSDARG00000001996	TVTKV
5	>ENSDARG00000052294	CPTVV	55	>ENSDARG00000076429	IITPV	105	>ENSDARG00000001634	MQTHV	155	>ENSDARG00000020169	RNTVV	205	>ENSDARG00000015611	TVTSV
6	>ENSDARG00000036548	CTTCV	56	>ENSDARG00000058995	IRTIV	106	>ENSDARG00000019473	MQTHV	156	>ENSDARG00000063207	RNTVV	206	>ENSDARG00000001559	VCTAV
7	>ENSDARG00000037781	CYTAV	57	>ENSDARG00000073914	ISTDV	107	>ENSDARG00000056998	MQTHV	157	>ENSDARG00000078874	RNTVV	207	>ENSDARG00000014391	VCTMV
8	>ENSDARG00000061738	DETIV	58	>ENSDARG00000077470	ISTDV	108	>ENSDARG00000060939	MQTHV	158	>ENSDARG00000060597	RRTAV	208	>ENSDARG00000033473	VCTMV
9	>ENSDARG00000054619	DGTEV	59	>ENSDARG00000070222	ISTLV	109	>ENSDARG00000075806	MQTHV	159	>ENSDARG00000078221	RTTDV	209	>ENSDARG00000058353	VCTVV
10	>ENSDARG00000043313	DQTEV	60	>ENSDARG00000062335	ISTNV	110	>ENSDARG00000022378	MQTTV	160	>ENSDARG00000009621	RTTPV	210	>ENSDARG00000014358	VGTPV
11	>ENSDARG00000024933	DTTGV	61	>ENSDARG00000068814	ISTPV	111	>ENSDARG00000073916	MSTDV	161	>ENSDARG00000020450	RTTPV	211	>ENSDARG00000075832	VITSV
12	>ENSDARG00000030530	DVTAV	62	>ENSDARG00000063215	ISTVV	112	>ENSDARG00000038094	MSTGV	162	>ENSDARG00000032565	RTTPV	212	>ENSDARG00000056986	VMTLV
13	>ENSDARG00000036272	EETNV	63	>ENSDARG00000077709	ISTVV	113	>ENSDARG00000057718	NATDV	163	>ENSDARG00000058460	RTTPV	213	>ENSDARG00000004616	VQTFV
14	>ENSDARG00000077987	EETTV	64	>ENSDARG00000058868	IVTSV	114	>ENSDARG00000036774	NATFV	164	>ENSDARG00000074669	RTTPV	214	>ENSDARG00000025728	VSTVV
15	>ENSDARG00000009550	EGTEV	65	>ENSDARG00000073830	IYTLV	115	>ENSDARG00000077349	NCTNV	165	>ENSDARG00000076401	RTTPV	215	>ENSDARG00000026333	YETTV
16	>ENSDARG00000057910	EGTPV	66	>ENSDARG00000079152	IYTLV	116	>ENSDARG00000075865	NCTSV	166	>ENSDARG00000075212	RVTSV	216	>ENSDARG00000036952	YETTV
17	>ENSDARG00000041703	EGTSV	67	>ENSDARG00000009610	KATFV	117	>ENSDARG00000005522	NCTTV	167	>ENSDARG00000055740	RVTTV			
18	>ENSDARG00000044220	EGTSV	68	>ENSDARG00000005673	KETIV	118	>ENSDARG00000075102	NETTV	168	>ENSDARG00000055839	RVTTV			
19	>ENSDARG00000076108	EKTEV	69	>ENSDARG00000032765	KETMV	119	>ENSDARG00000076577	NETTV	169	>ENSDARG00000056439	RVTTV			
20	>ENSDARG00000061173	EKTGV	70	>ENSDARG00000062959	KETVV	120	>ENSDARG00000073795	NHTPV	170	>ENSDARG00000075375	RVTWV			
21	>ENSDARG00000076923	ELTNV	71	>ENSDARG00000074635	KETVV	121	>ENSDARG00000045638	NSTGV	171	>ENSDARG00000070892	RYTTV			
22	>ENSDARG00000071669	EMTDV	72	>ENSDARG00000004336	KITTV	122	>ENSDARG00000030472	NTTCV	172	>ENSDARG00000061187	SATAV			
23	>ENSDARG00000077217	EMTYV	73	>ENSDARG00000046014	KLTDV	123	>ENSDARG00000077691	NTTDV	173	>ENSDARG00000063553	SETKV			
24	>ENSDARG00000011724	ERTVV	74	>ENSDARG00000076745	KMTMV	124	>ENSDARG00000071596	NTTRV	174	>ENSDARG00000004305	SETSV			
25	>ENSDARG00000012353	ETTVV	75	>ENSDARG00000031013	KNTQV	125	>ENSDARG00000068213	PATCV	175	>ENSDARG00000027397	SETSV			
26	>ENSDARG00000002336	FATEV	76	>ENSDARG00000045093	KQTSV	126	>ENSDARG00000075062	PETWV	176	>ENSDARG00000079220	SGTVV			
27	>ENSDARG00000004282	FGTEV	77	>ENSDARG00000037371	KSTAV	127	>ENSDARG00000074599	PGTIV	177	>ENSDARG00000070162	SITSV			
28	>ENSDARG00000006916	FGTTV	78	>ENSDARG00000073684	KSTEV	128	>ENSDARG00000076598	PGTIV	178	>ENSDARG00000075481	SKTTV			
29	>ENSDARG00000025218	FLTRV	79	>ENSDARG00000070626	KTTPV	129	>ENSDARG00000012449	PGTLV	179	>ENSDARG00000035185	SPTHV			
30	>ENSDARG00000061635	FLTRV	80	>ENSDARG00000076478	KTTTV	130	>ENSDARG00000032856	PGTLV	180	>ENSDARG00000009131	SPTPV			
31	>ENSDARG00000074622	FLTRV	81	>ENSDARG00000002172	LATKV	131	>ENSDARG00000004561	PLTAV	181	>ENSDARG00000078325	SRTRV			
32	>ENSDARG00000018361	FRTEV	82	>ENSDARG00000036670	LATKV	132	>ENSDARG00000041125	PNTFV	182	>ENSDARG00000062376	STTRV			
33	>ENSDARG00000074553	FSTDV	83	>ENSDARG00000078356	LATKV	133	>ENSDARG00000075964	PSTIV	183	>ENSDARG00000062581	STTRV			
34	>ENSDARG00000029096	FSTEV	84	>ENSDARG00000062820	LETEV	134	>ENSDARG00000035505	PTTPV	184	>ENSDARG00000077329	STTRV			
35	>ENSDARG00000042329	FSTEV	85	>ENSDARG00000070772	LETEV	135	>ENSDARG00000043055	QDTNV	185	>ENSDARG00000077710	STTRV			
36	>ENSDARG00000027589	GETTV	86	>ENSDARG00000070774	LETEV	136	>ENSDARG00000077312	QETAV	186	>ENSDARG00000077761	STTRV			
37	>ENSDARG00000054438	GETTV	87	>ENSDARG00000052466	LETLV	137	>ENSDARG00000077188	QGTCV	187	>ENSDARG00000079251	STTRV			
38	>ENSDARG00000038496	GKTSV	88	>ENSDARG00000034940	LETNV	138	>ENSDARG00000013763	QGTSV	188	>ENSDARG00000038293	STTTV			
39	>ENSDARG00000076025	HETAV	89	>ENSDARG00000078317	LETQV	139	>ENSDARG00000017591	QHTEV	189	>ENSDARG00000042861	SVTPV			
40	>ENSDARG00000073673	HETTV	90	>ENSDARG00000014599	LETSV	140	>ENSDARG00000056623	QITKV	190	>ENSDARG00000074049	SVTPV			
41	>ENSDARG00000057468	HGTGV	91	>ENSDARG00000009482	LITEV	141	>ENSDARG00000071018	QITKV	191	>ENSDARG00000045687	SVTTV			
42	>ENSDARG00000037341	HITAV	92	>ENSDARG00000062942	LLTDV	142	>ENSDARG00000018066	QITTV	192	>ENSDARG00000060182	SVTTV			
43	>ENSDARG00000068946	HITAV	93	>ENSDARG00000039675	LMTTV	143	>ENSDARG00000021113	QKTDV	193	>ENSDARG00000073757	SVTTV			
44	>ENSDARG00000035761	HITFV	94	>ENSDARG00000078650	LQTDV	144	>ENSDARG00000032603	QLTNV	194	>ENSDARG00000073869	SVTTV			
45	>ENSDARG00000008249	HVTNV	95	>ENSDARG00000076130	LQTEV	145	>ENSDARG00000052383	QTTAV	195	>ENSDARG00000024865	SYTLV			
46	>ENSDARG00000010791	IATEV	96	>ENSDARG00000076460	LQTEV	146	>ENSDARG00000042550	RDTFV	196	>ENSDARG00000028118	SYTLV			
47	>ENSDARG00000020219	IATEV	97	>ENSDARG00000042962	LSTVV	147	>ENSDARG00000037020	RETAV	197	>ENSDARG00000057052	SYTLV			
48	>ENSDARG00000070425	IATEV	98	>ENSDARG00000074147	LSTVV	148	>ENSDARG00000004706	RGTAV	198	>ENSDARG00000013552	TETDV			
49	>ENSDARG00000074480	IETHV	99	>ENSDARG00000075627	LTTMV	149	>ENSDARG00000053164	RHTRV	199	>ENSDARG00000000804	TETTV			
50	>ENSDARG00000053228	IETTV	100	>ENSDARG00000030957	LYTGV	150	>ENSDARG00000039238	RITPV	200	>ENSDARG00000075164	TLTVV			

Additional file 4. continued.

zebrafish xxVxI

No.	gene ID	5aa
1	>ENSDARG00000039066	APVSI
2	>ENSDARG00000041431	CPVSI
3	>ENSDARG00000079953	CVVDI
4	>ENSDARG00000037496	DAVKI
5	>ENSDARG00000078610	EDVQI
6	>ENSDARG00000032737	ESVKI
7	>ENSDARG00000037498	ESVKI
8	>ENSDARG00000052765	ESVKI
9	>ENSDARG00000070173	ESVKI
10	>ENSDARG00000070775	ETVRI
11	>ENSDARG00000038668	GKVKI
12	>ENSDARG00000006981	GLVPI
13	>ENSDARG00000068955	LCVII
14	>ENSDARG00000010078	LKVQI
15	>ENSDARG00000073862	LSVVI
16	>ENSDARG00000078260	MKVHI
17	>ENSDARG00000019874	NHVNI
18	>ENSDARG00000058724	NKVGI
19	>ENSDARG00000043089	NSVAI
20	>ENSDARG00000069966	NSVAI
21	>ENSDARG00000016789	NSVTI
22	>ENSDARG00000042827	PEVTI
23	>ENSDARG00000040547	PNVEI
24	>ENSDARG00000060977	PSVFI
25	>ENSDARG00000023609	PSVHI
26	>ENSDARG00000077563	PSVWI
27	>ENSDARG00000034138	PTVTI
28	>ENSDARG00000069411	QEVHI
29	>ENSDARG00000054152	QGVCI
30	>ENSDARG00000030752	QPVFI
31	>ENSDARG00000014105	QPVYI
32	>ENSDARG00000074328	QPVYI
33	>ENSDARG00000037568	QQVKI
34	>ENSDARG00000015070	QVVKI
35	>ENSDARG00000056938	RAVVI
36	>ENSDARG00000030250	RGVVI
37	>ENSDARG00000037514	SDVEI
38	>ENSDARG00000021193	SKVAI
39	>ENSDARG00000011925	SSVLI
40	>ENSDARG00000061841	STVAI
41	>ENSDARG00000017927	TEVWI
42	>ENSDARG00000024575	TFVGI
43	>ENSDARG00000040910	TSVVI
44	>ENSDARG00000073896	TSVWI
45	>ENSDARG00000041005	VNVQI
46	>ENSDARG00000076028	YEVSI
47	>ENSDARG00000076052	YEVSI

zebrafish xxVxV

No.	gene ID	5aa	No.	gene ID	5aa
1	>ENSDARG00000052855	AAVSV	51	>ENSDARG00000001939	PDVEV
2	>ENSDARG00000021239	ACVCV	52	>ENSDARG00000030780	PEVYV
3	>ENSDARG00000078218	AHVFV	53	>ENSDARG00000079553	PEVYV
4	>ENSDARG00000079906	AHVFV	54	>ENSDARG00000034685	PHVRV
5	>ENSDARG00000024877	AIVKV	55	>ENSDARG00000004898	QPVPV
6	>ENSDARG00000001729	AKVFV	56	>ENSDARG00000021539	QSVEV
7	>ENSDARG00000060595	AKVFV	57	>ENSDARG00000037373	QSVEV
8	>ENSDARG00000041823	ALVSV	58	>ENSDARG00000042090	RAVIV
9	>ENSDARG00000069445	ALVSV	59	>ENSDARG00000015016	REVEV
10	>ENSDARG00000035564	CTVDV	60	>ENSDARG00000043852	RGVQV
11	>ENSDARG00000006501	DLVFV	61	>ENSDARG00000054454	RMVPV
12	>ENSDARG00000068283	DLVFV	62	>ENSDARG00000043884	RQVPV
13	>ENSDARG00000003751	DLVYV	63	>ENSDARG00000060001	SAVSV
14	>ENSDARG00000079917	EGVYV	64	>ENSDARG00000073900	SAVWV
15	>ENSDARG00000040928	EKVFV	65	>ENSDARG00000062756	STVPV
16	>ENSDARG00000045542	EPVIV	66	>ENSDARG00000062831	TAVYV
17	>ENSDARG00000078864	EPVYV	67	>ENSDARG00000074731	TCVVV
18	>ENSDARG00000043767	ESVVV	68	>ENSDARG00000045145	TEVTV
19	>ENSDARG00000043474	FCVRV	69	>ENSDARG00000052642	TEVTV
20	>ENSDARG00000069058	FYVHV	70	>ENSDARG00000004635	TGVQV
21	>ENSDARG00000057729	GHVEV	71	>ENSDARG00000011600	THVQV
22	>ENSDARG00000011973	GKVAV	72	>ENSDARG00000054575	TNVAV
23	>ENSDARG00000039373	GPVPV	73	>ENSDARG00000057138	TQVPV
24	>ENSDARG00000055279	GRVEV	74	>ENSDARG00000020080	TQVQV
25	>ENSDARG00000056920	GSVWV	75	>ENSDARG00000038243	TVVVV
26	>ENSDARG00000026862	HKVSV	76	>ENSDARG00000009783	VFVSV
27	>ENSDARG00000026400	IAVLV	77	>ENSDARG00000073823	VFVTV
28	>ENSDARG00000076056	IMVLV	78	>ENSDARG00000079940	VFVTV
29	>ENSDARG00000078715	IMVLV	79	>ENSDARG00000044815	VSVSV
30	>ENSDARG00000058874	IQVVV	80	>ENSDARG00000070355	VTVDV
31	>ENSDARG00000036171	KGVIV	81	>ENSDARG00000055240	WAVQV
32	>ENSDARG00000059606	KRVHV	82	>ENSDARG00000005679	YEVWV
33	>ENSDARG00000042595	KTVKV	83	>ENSDARG00000044093	YGVLV
34	>ENSDARG00000033589	LPVQV			
35	>ENSDARG00000035474	LSVRV			
36	>ENSDARG00000038459	LSVRV			
37	>ENSDARG00000042991	LSVRV			
38	>ENSDARG00000077448	LSVRV			
39	>ENSDARG00000078996	LSVRV			
40	>ENSDARG00000059824	LWVSV			
41	>ENSDARG00000045092	MGVHV			
42	>ENSDARG00000013013	MVVDV			
43	>ENSDARG00000069360	MVVDV			
44	>ENSDARG00000044514	NDVPV			
45	>ENSDARG00000062462	NEVTV			
46	>ENSDARG00000063144	NEVTV			
47	>ENSDARG00000057303	NIVAV			
48	>ENSDARG00000057273	NSVAV			
49	>ENSDARG00000073781	NSVSV			
50	>ENSDARG00000035458	NYVDV			

Additional file 4. continued.

fruit fly xxSxI

No.	gene ID	5aa	No.	gene ID	5aa
1	>FBgn0026394	AKSVI	51	>FBgn0035765	RESDI
2	>FBgn0039172	ATSSI	52	>FBgn0001624	RKSSI
3	>FBgn0040297	DESII	53	>FBgn0000630	RLSLI
4	>FBgn0051961	DISII	54	>FBgn0053688	RVSNI
5	>FBgn0014031	DLSKI	55	>FBgn0035567	SESNI
6	>FBgn0053208	EESII	56	>FBgn0053054	SFSLI
7	>FBgn0032264	ENSII	57	>FBgn0035914	SHSHI
8	>FBgn0031150	EQSAI	58	>FBgn0085403	SISPI
9	>FBgn0015400	ETSDI	59	>FBgn0011224	SKSNI
10	>FBgn0040060	ETSGI	60	>FBgn0061492	SKSRI
11	>FBgn0036741	FCSRI	61	>FBgn0034075	SLSPI
12	>FBgn0050377	FGSMI	62	>FBgn0026136	SPSHI
13	>FBgn0021872	FPSLI	63	>FBgn0015608	SRSPI
14	>FBgn0027330	GKSLI	64	>FBgn0015316	SVSGI
15	>FBgn0038672	GRSWI	65	>FBgn0037236	SVSYI
16	>FBgn0039718	GVSLI	66	>FBgn0030027	TISAI
17	>FBgn0036193	HISRI	67	>FBgn0053178	TLSFI
18	>FBgn0035800	IASNI	68	>FBgn0035954	TSSSI
19	>FBgn0037356	IASYI	69	>FBgn0030379	VKSLI
20	>FBgn0031718	ILSRI	70	>FBgn0034162	VRSSI
21	>FBgn0033112	KASYI	71	>FBgn0000055	WDSGI
22	>FBgn0038612	KISAI	72	>FBgn0039358	YFSQI
23	>FBgn0031484	KKSAI	73	>FBgn0036689	YISSI
24	>FBgn0011692	KKSKI	74	>FBgn0023441	YNSGI
25	>FBgn0031289	KLSII	75	>FBgn0037328	YPSRI
26	>FBgn0033818	KSSII	76	>FBgn0051910	YVSRI
27	>FBgn0030524	LASSI			
28	>FBgn0085521	LDSNI			
29	>FBgn0085693	LDSNI			
30	>FBgn0039876	LESRI			
31	>FBgn0001977	LKSDI			
32	>FBgn0005630	LKSII			
33	>FBgn0004652	LNSSI			
34	>FBgn0036711	LNSSI			
35	>FBgn0043070	LSSFI			
36	>FBgn0040011	LSSRI			
37	>FBgn0034271	LSSVI			
38	>FBgn0028891	LTSGI			
39	>FBgn0034774	MHSPI			
40	>FBgn0034628	MKSNI			
41	>FBgn0029154	MLSAI			
42	>FBgn0050429	NISQI			
43	>FBgn0034049	NTSVI			
44	>FBgn0039734	PRSII			
45	>FBgn0038156	QESCI			
46	>FBgn0038390	QLSVI			
47	>FBgn0028852	QMSGI			
48	>FBgn0023181	QSSLI			
49	>FBgn0011582	QVSAI			
50	>FBgn0038874	RASKI			

Additional file 4. continued.

fruit fly xxSxL

No.	gene ID	5aa	No.	gene ID	5aa	No.	gene ID	5aa
1	>FBgn0037312	AHSEL	51	>FBgn0031930	LKSAL	101	>FBgn0039490	VSSLL
2	>FBgn0029803	AISQL	52	>FBgn0039537	LKSKL	102	>FBgn0005633	YASVL
3	>FBgn0035309	AKSKL	53	>FBgn0052791	LLSLL	103	>FBgn0035632	YQSSL
4	>FBgn0042710	AKSKL	54	>FBgn0026178	LLSWL			
5	>FBgn0039723	ANSQL	55	>FBgn0046302	LRSAL			
6	>FBgn0032775	AQSKL	56	>FBgn0015808	LRSKL			
7	>FBgn0001247	ARSKL	57	>FBgn0034789	LTSIL			
8	>FBgn0027793	ARSSL	58	>FBgn0038636	LVSAL			
9	>FBgn0015954	ATSKL	59	>FBgn0030595	MNSGL			
10	>FBgn0037828	ATSSL	60	>FBgn0085303	MRSLL			
11	>FBgn0032079	CCSFL	61	>FBgn0026076	NCSIL			
12	>FBgn0029801	CDSFL	62	>FBgn0031860	NGSKL			
13	>FBgn0033458	CDSYL	63	>FBgn0015239	NTSGL			
14	>FBgn0033697	CRSSL	64	>FBgn0032237	NVSNL			
15	>FBgn0035500	DISLL	65	>FBgn0085292	NVSRL			
16	>FBgn0085595	DLSTL	66	>FBgn0027507	NVSTL			
17	>FBgn0033551	DNSPL	67	>FBgn0023529	PHSEL			
18	>FBgn0037310	DSSAL	68	>FBgn0030056	PKSKL			
19	>FBgn0050463	DSSKL	69	>FBgn0031987	PTSAL			
20	>FBgn0019948	EASTL	70	>FBgn0032271	PTSEL			
21	>FBgn0053194	EDSTL	71	>FBgn0037585	QGSAL			
22	>FBgn0038564	EQSLL	72	>FBgn0039156	QKSKL			
23	>FBgn0029898	ESSRL	73	>FBgn0085440	QMSYL			
24	>FBgn0051281	FISDL	74	>FBgn0028569	RASAL			
25	>FBgn0036367	FISFL	75	>FBgn0015796	RCSLL			
26	>FBgn0029848	FISVL	76	>FBgn0035929	RDSPL			
27	>FBgn0033543	GNSKL	77	>FBgn0004573	RESFL			
28	>FBgn0035015	GNSSL	78	>FBgn0026593	RKSNL			
29	>FBgn0032497	GQSDL	79	>FBgn0085548	RLSVL			
30	>FBgn0038652	GRSAL	80	>FBgn0033827	RNSLL			
31	>FBgn0030680	GRSSL	81	>FBgn0035312	RSSKL			
32	>FBgn0028479	GSSKL	82	>FBgn0036749	RTSQL			
33	>FBgn0003366	GVSRL	83	>FBgn0004377	SCSIL			
34	>FBgn0038294	HRSIL	84	>FBgn0085614	SDSKL			
35	>FBgn0035092	HRSRL	85	>FBgn0035173	SESKL			
36	>FBgn0033115	HVSQL	86	>FBgn0025383	SGSWL			
37	>FBgn0003961	INSHL	87	>FBgn0085336	SISVL			
38	>FBgn0037456	IRSDL	88	>FBgn0027095	SRSEL			
39	>FBgn0035065	IRSKL	89	>FBgn0036994	SRSSL			
40	>FBgn0037070	KESKL	90	>FBgn0050085	SSSNL			
41	>FBgn0039543	KESKL	91	>FBgn0086532	SSSVL			
42	>FBgn0002781	KHSHL	92	>FBgn0034488	TESKL			
43	>FBgn0034486	KQSFL	93	>FBgn0037440	TKSKL			
44	>FBgn0034988	KQSSL	94	>FBgn0033584	TKSQL			
45	>FBgn0026570	KRSKL	95	>FBgn0040234	TSSLL			
46	>FBgn0031837	KVSLL	96	>FBgn0033785	VDSRL			
47	>FBgn0034426	LASKL	97	>FBgn0058249	VHSTL			
48	>FBgn0039560	LDSIL	98	>FBgn0026415	VKSKL			
49	>FBgn0013679	LFSLL	99	>FBgn0031887	VNSLL			
50	>FBgn0085539	LHSLL	100	>FBgn0000363	VRSYL			

Additional file 4. continued.

fruit fly xxSxV

No.	gene ID	5aa	No.	gene ID	5aa
1	>FBgn0016797	AASHV	51	>FBgn0086901	PESKV
2	>FBgn0036170	AHSDV	52	>FBgn0064225	PHSAV
3	>FBgn0086446	ARSVV	53	>FBgn0030347	PKSRV
4	>FBgn0030349	ATSFV	54	>FBgn0037057	PLSFV
5	>FBgn0033391	DASDV	55	>FBgn0037783	PRSAV
6	>FBgn0085650	DGSSV	56	>FBgn0040001	PVSVV
7	>FBgn0085724	DGSSV	57	>FBgn0031842	PYSEV
8	>FBgn0037724	DSSAV	58	>FBgn0037553	QDSEV
9	>FBgn0050432	DSSCV	59	>FBgn0083950	QESCV
10	>FBgn0028539	ECSYV	60	>FBgn0011674	QESFV
11	>FBgn0003016	EDSEV	61	>FBgn0041245	QFSSV
12	>FBgn0050002	EFSDV	62	>FBgn0037603	QKSDV
13	>FBgn0051954	EHSGV	63	>FBgn0037026	QLSRV
14	>FBgn0085663	EVSKV	64	>FBgn0030648	QTSNV
15	>FBgn0026616	FRSEV	65	>FBgn0037736	RDSTV
16	>FBgn0027525	GASIV	66	>FBgn0013974	RESIV
17	>FBgn0035207	GFSLV	67	>FBgn0030476	RNSIV
18	>FBgn0037242	GGSVV	68	>FBgn0004858	RSSLV
19	>FBgn0037199	GISFV	69	>FBgn0011554	RTSYV
20	>FBgn0028741	GLSKV	70	>FBgn0029789	RVSRV
21	>FBgn0085383	GMSEV	71	>FBgn0036278	RVSSV
22	>FBgn0000163	HASKV	72	>FBgn0031910	SDSDV
23	>FBgn0040295	HISAV	73	>FBgn0029531	SDSPV
24	>FBgn0036467	HPSLV	74	>FBgn0000543	SESAV
25	>FBgn0037021	INSAV	75	>FBgn0035049	SGSFV
26	>FBgn0037565	KESDV	76	>FBgn0069946	SHSAV
27	>FBgn0034345	KESTV	77	>FBgn0085716	SHSAV
28	>FBgn0051534	KFSCV	78	>FBgn0013725	SISLV
29	>FBgn0011834	KHSDV	79	>FBgn0036513	SKSGV
30	>FBgn0000635	KNSAV	80	>FBgn0033183	SLSTV
31	>FBgn0003717	KQSDV	81	>FBgn0046114	SRSSV
32	>FBgn0030582	KSSIV	82	>FBgn0020278	SSSFV
33	>FBgn0052683	KSSNV	83	>FBgn0051164	SVSIV
34	>FBgn0029949	LASVV	84	>FBgn0036533	TDSNV
35	>FBgn0030989	LESPV	85	>FBgn0003410	TISLV
36	>FBgn0038032	LGSFV	86	>FBgn0085593	TNSNV
37	>FBgn0058275	LPSFV	87	>FBgn0023513	VLSRV
38	>FBgn0085448	LSSLV	88	>FBgn0002571	VLSTV
39	>FBgn0030506	MHSFV	89	>FBgn0086898	VSSLV
40	>FBgn0085200	MLSIV	90	>FBgn0039911	VTSSV
41	>FBgn0013751	MPSEV	91	>FBgn0033452	WQSCV
42	>FBgn0033662	MSSIV	92	>FBgn0037166	YCSKV
43	>FBgn0020617	MVSIV	93	>FBgn0000455	YDSPV
44	>FBgn0036610	NLSPV	94	>FBgn0030303	YESSV
45	>FBgn0013591	NNSGV	95	>FBgn0032189	YFSSV
46	>FBgn0015954	NNSKV	96	>FBgn0011239	YQSEV
47	>FBgn0027343	NQSRV	97	>FBgn0085431	YQSYV
48	>FBgn0034172	NSSNV			
49	>FBgn0032555	PESCV			
50	>FBgn0083949	PESCV			

fruit fly xxTxI

No.	gene ID	5aa	No.	gene ID	5aa
1	>FBgn0039782	CLTSI	51	>FBgn0050185	TGTHI
2	>FBgn0033916	DETDI	52	>FBgn0035664	TNTGI
3	>FBgn0029990	EATTI	53	>FBgn0052432	VETKI
4	>FBgn0037005	EATTI	54	>FBgn0011737	VITNI
5	>FBgn0039536	EDTLI	55	>FBgn0027565	VKTAI
6	>FBgn0037100	EETCI	56	>FBgn0015008	YDTNI
7	>FBgn0036884	EETII	57	>FBgn0025624	YSTTI
8	>FBgn0023197	EHTGI			
9	>FBgn0032779	ELTPI			
10	>FBgn0015129	EQTYI			
11	>FBgn0036834	EVTRI			
12	>FBgn0004784	FITII			
13	>FBgn0015933	FLTKI			
14	>FBgn0039451	GGTVI			
15	>FBgn0038568	GVTDI			
16	>FBgn0031520	IATHI			
17	>FBgn0045500	IFTLI			
18	>FBgn0040344	INTDI			
19	>FBgn0033032	IQTDI			
20	>FBgn0051146	ITTNI			
21	>FBgn0050190	KETQI			
22	>FBgn0034716	KNTEI			
23	>FBgn0085373	KQTRI			
24	>FBgn0016059	KRTYI			
25	>FBgn0025936	LDTII			
26	>FBgn0029692	MPTPI			
27	>FBgn0025704	NETRI			
28	>FBgn0054036	NETRI			
29	>FBgn0032296	NFTRI			
30	>FBgn0053981	NITSI			
31	>FBgn0038770	NQTKI			
32	>FBgn0039821	NSTSI			
33	>FBgn0038442	PDTQI			
34	>FBgn0040343	PETII			
35	>FBgn0084026	PKTQI			
36	>FBgn0033549	PNTLI			
37	>FBgn0000636	QETEI			
38	>FBgn0030844	QSTSI			
39	>FBgn0000588	QTTSI			
40	>FBgn0083976	RETDI			
41	>FBgn0033556	RGTAI			
42	>FBgn0034853	RRTEI			
43	>FBgn0003475	RRTSI			
44	>FBgn0034121	SETWI			
45	>FBgn0069969	SITRI			
46	>FBgn0052642	SNTEI			
47	>FBgn0039214	SPTQI			
48	>FBgn0052095	SSTEI			
49	>FBgn0050286	STTHI			
50	>FBgn0034398	SYTKI			

Additional file 4. continued.

fruit fly xxTxL

No.	gene ID	5aa	No.	gene ID	5aa	No.	gene ID	5aa
1	>FBgn0033422	AKTIL	51	>FBgn0039296	KHTEL	101	>FBgn0045843	SSTHL
2	>FBgn0039761	ALTNL	52	>FBgn0030847	KHTKL	102	>FBgn0035217	STTFL
3	>FBgn0034264	AMTSL	53	>FBgn0036331	KITKL	103	>FBgn0039595	STTGL
4	>FBgn0037897	APTPL	54	>FBgn0086448	KITTL	104	>FBgn0026875	SVTEL
5	>FBgn0058133	APTVL	55	>FBgn0033663	KKTEL	105	>FBgn0034179	SYTQL
6	>FBgn0019643	AQTKL	56	>FBgn0036349	KKTTL	106	>FBgn0035451	TETNL
7	>FBgn0032749	ATTEL	57	>FBgn0031168	KNTHL	107	>FBgn0052220	TKTEL
8	>FBgn0034553	ATTHL	58	>FBgn0039674	KSTGL	108	>FBgn0022724	TKTNL
9	>FBgn0034540	ATTSL	59	>FBgn0042103	KSTWL	109	>FBgn0032689	TSTTL
10	>FBgn0015558	AVTEL	60	>FBgn0036789	LHTDL	110	>FBgn0039709	TTTEL
11	>FBgn0050479	CCTIL	61	>FBgn0030603	LKTDL	111	>FBgn0035131	TVTSL
12	>FBgn0025806	CCTLL	62	>FBgn0026396	LKTFL	112	>FBgn0031902	VNTCL
13	>FBgn0052711	CLTFL	63	>FBgn0038431	LLTKL	113	>FBgn0034490	VNTKL
14	>FBgn0000439	DLTAL	64	>FBgn0050361	LQTNL	114	>FBgn0032663	VSTDL
15	>FBgn0032790	DPTKL	65	>FBgn0034942	LRTDL	115	>FBgn0026239	VSTFL
16	>FBgn0032514	DRTEL	66	>FBgn0036249	LTTNL	116	>FBgn0027556	VVTRL
17	>FBgn0030669	EATGL	67	>FBgn0031671	LTTYL	117	>FBgn0029962	VVTSL
18	>FBgn0035676	EATRL	68	>FBgn0039117	LYTEL	118	>FBgn0037950	WTTFL
19	>FBgn0039597	EDTIL	69	>FBgn0028668	LYTKL	119	>FBgn0085420	YLTLL
20	>FBgn0041096	EITDL	70	>FBgn0053513	METVL	120	>FBgn0027660	YSTEL
21	>FBgn0051321	EITSL	71	>FBgn0020377	MVTDL	121	>FBgn0036588	YTTVL
22	>FBgn0086904	ELTML	72	>FBgn0027342	NETLL			
23	>FBgn0020620	ENTRL	73	>FBgn0029768	NETVL			
24	>FBgn0035572	ESTEL	74	>FBgn0038752	NNTRL			
25	>FBgn0026379	ESTYL	75	>FBgn0040299	NQTRL			
26	>FBgn0030747	ESTYL	76	>FBgn0050033	NSTAL			
27	>FBgn0038247	ETTEL	77	>FBgn0035672	NSTKL			
28	>FBgn0026239	FETAL	78	>FBgn0051100	NTTHL			
29	>FBgn0069969	FRTCL	79	>FBgn0085433	PETTL			
30	>FBgn0036229	FVTEL	80	>FBgn0024989	PGTIL			
31	>FBgn0003129	GATGL	81	>FBgn0037234	PITDL			
32	>FBgn0034249	GETAL	82	>FBgn0030993	PKTNL			
33	>FBgn0000667	GETDL	83	>FBgn0034907	PQTGL			
34	>FBgn0028379	GKTRL	84	>FBgn0023095	PVTEL			
35	>FBgn0000659	GTTSL	85	>FBgn0033432	QFTTL			
36	>FBgn0033938	HPTIL	86	>FBgn0031288	QQTDL			
37	>FBgn0014033	HRTDL	87	>FBgn0036790	QQTDL			
38	>FBgn0031106	ILTKL	88	>FBgn0036565	QTTLL			
39	>FBgn0029931	ISTDL	89	>FBgn0034396	QTTNL			
40	>FBgn0004359	ITTEL	90	>FBgn0038569	QTTQL			
41	>FBgn0004049	ITTQL	91	>FBgn0035331	QVTNL			
42	>FBgn0054046	IVTKL	92	>FBgn0003189	RNTAL			
43	>FBgn0016081	IVTNL	93	>FBgn0022382	RNTNL			
44	>FBgn0004009	IYTCL	94	>FBgn0004921	SCTVL			
45	>FBgn0015286	KCTLL	95	>FBgn0037751	SDTYL			
46	>FBgn0034117	KFTAL	96	>FBgn0032621	SFTHL			
47	>FBgn0039645	KGTTL	97	>FBgn0085665	SGTSL			
48	>FBgn0042102	KGTWL	98	>FBgn0031395	SHTGL			
49	>FBgn0030593	KHTEL	99	>FBgn0052387	SITYL			
50	>FBgn0037756	KHTEL	100	>FBgn0038067	SPTFL			

Additional file 4. continued.

fruit fly xxTxV

No.	gene ID	5aa	No.	gene ID	5aa
1	>FBgn0032095	AETKV	51	>FBgn0051076	QTTSV
2	>FBgn0003048	AGTTV	52	>FBgn0004919	QVTDV
3	>FBgn0030052	APTNV	53	>FBgn0035329	QVTNV
4	>FBgn0032341	AYTNV	54	>FBgn0025704	RETEV
5	>FBgn0027598	CVTQV	55	>FBgn0029646	RITAV
6	>FBgn0039740	DCTPV	56	>FBgn0039897	RNTNV
7	>FBgn0020224	DDTEV	57	>FBgn0026179	RSTEV
8	>FBgn0024836	DETTV	58	>FBgn0032048	SETDV
9	>FBgn0031016	DGTEV	59	>FBgn0015838	SETSV
10	>FBgn0037245	DTTTV	60	>FBgn0041582	SSTCV
11	>FBgn0030360	FYTSV	61	>FBgn0038327	SVTSV
12	>FBgn0028481	GVTNV	62	>FBgn0025704	TETAV
13	>FBgn0036773	HSTEV	63	>FBgn0036943	TETDV
14	>FBgn0010416	HYTTV	64	>FBgn0003731	TETRV
15	>FBgn0003380	IETDV	65	>FBgn0038150	TPTNV
16	>FBgn0031568	ITTSV	66	>FBgn0052982	TPTRV
17	>FBgn0034005	KCTHV	67	>FBgn0037915	TTTAV
18	>FBgn0051082	KETTV	68	>FBgn0033579	TTTCV
19	>FBgn0063497	KFTIV	69	>FBgn0052046	TVTIV
20	>FBgn0033753	KMTRV	70	>FBgn0002968	VATYV
21	>FBgn0030598	KNTIV	71	>FBgn0028862	VDTQV
22	>FBgn0051004	KSTEV	72	>FBgn0016762	VFTVV
23	>FBgn0033980	KVTRV	73	>FBgn0039393	VKTEV
24	>FBgn0028369	LATHV	74	>FBgn0031044	VQTTV
25	>FBgn0035104	LGTLV	75	>FBgn0086604	YATRV
26	>FBgn0038932	LITAV	76	>FBgn0037336	YGTTV
27	>FBgn0039419	LPTEV			
28	>FBgn0031053	LSTLV			
29	>FBgn0001323	LTTKV			
30	>FBgn0086349	MVTCV			
31	>FBgn0031159	NGTAV			
32	>FBgn0039525	NGTYV			
33	>FBgn0051906	NITPV			
34	>FBgn0041238	NKTKV			
35	>FBgn0039053	NLTEV			
36	>FBgn0052576	NRTTV			
37	>FBgn0052252	NVTCV			
38	>FBgn0031406	PATNV			
39	>FBgn0036732	PDTTV			
40	>FBgn0037720	PGTAV			
41	>FBgn0035367	PKTPV			
42	>FBgn0037228	PMTCV			
43	>FBgn0000636	PNTSV			
44	>FBgn0003285	PPTAV			
45	>FBgn0001987	PQTSV			
46	>FBgn0034031	PTTAV			
47	>FBgn0010226	PVTEV			
48	>FBgn0024188	QFTFV			
49	>FBgn0085392	QGTIV			
50	>FBgn0010414	QGTSV			

fruit fly xxVxI

No.	gene ID	5aa
1	>FBgn0085645	ALVDI
2	>FBgn0085694	ALVDI
3	>FBgn0086655	ALVPI
4	>FBgn0052212	AQVII
5	>FBgn0030878	AQVLI
6	>FBgn0030242	AVVAI
7	>FBgn0034638	CCVLI
8	>FBgn0052458	CEVHI
9	>FBgn0085696	DEVEI
10	>FBgn0051438	EIVNI
11	>FBgn0054011	ERVAI
12	>FBgn0032901	GFVGI
13	>FBgn0000356	GVVAI
14	>FBgn0044011	HIVDI
15	>FBgn0005630	HSVLI
16	>FBgn0034647	INVSI
17	>FBgn0050488	IQVDI
18	>FBgn0026207	KIVGI
19	>FBgn0019947	KQVFI
20	>FBgn0033067	KRVYI
21	>FBgn0020660	LKVLI
22	>FBgn0036515	LVVQI
23	>FBgn0035264	MTVEI
24	>FBgn0032013	NGVDI
25	>FBgn0003423	NYVPI
26	>FBgn0051021	PGVSI
27	>FBgn0037405	QNVLI
28	>FBgn0017566	QSVAI
29	>FBgn0015295	QTVHI
30	>FBgn0002777	RIVRI
31	>FBgn0051681	SIVKI
32	>FBgn0037517	SPVQI
33	>FBgn0050385	STVQI
34	>FBgn0036763	TKVEI
35	>FBgn0034083	TTVDI
36	>FBgn0052633	VLVRI
37	>FBgn0002938	YAVDI
38	>FBgn0035621	YAVII
39	>FBgn0019990	YRVII
40	>FBgn0024943	YVVEI

Additional file 4. continued.

fruit fly xxVxV

No.	gene ID	5aa	No.	gene ID	5aa
1	>FBgn0025680	ADVVV	51	>FBgn0004197	TTVMV
2	>FBgn0026375	AFVMV	52	>FBgn0037515	VAVAV
3	>FBgn0044323	AKVFV	53	>FBgn0002570	VLVAV
4	>FBgn0036594	APVVV	54	>FBgn0035409	VSVVV
5	>FBgn0011739	APVYV	55	>FBgn0036643	VVVFV
6	>FBgn0034548	ATVDV	56	>FBgn0053967	YGVEV
7	>FBgn0003317	DEVCV	57	>FBgn0051534	YIVWV
8	>FBgn0053224	DEVQV	58	>FBgn0020300	YKVRV
9	>FBgn0032242	DIVNV			
10	>FBgn0038762	EEVDV			
11	>FBgn0036361	EFVGV			
12	>FBgn0033232	ELVQV			
13	>FBgn0034136	EPVDV			
14	>FBgn0032471	ETVLV			
15	>FBgn0032485	GAVVV			
16	>FBgn0035696	GEVYV			
17	>FBgn0039352	HGVWV			
18	>FBgn0039685	HSVKV			
19	>FBgn0085397	HVVYV			
20	>FBgn0001320	IFVCV			
21	>FBgn0086371	IGVKV			
22	>FBgn0050166	ILVPV			
23	>FBgn0085560	KDVEV			
24	>FBgn0052227	KEVAV			
25	>FBgn0002905	KEVSV			
26	>FBgn0085401	KEVSV			
27	>FBgn0086685	KGVVV			
28	>FBgn0026319	KIVAV			
29	>FBgn0032877	LSVYV			
30	>FBgn0028663	MAVTV			
31	>FBgn0028936	MCVPV			
32	>FBgn0031420	MRVFV			
33	>FBgn0010014	MVVDV			
34	>FBgn0015614	MVVDV			
35	>FBgn0030514	NAVNV			
36	>FBgn0036368	NHVEV			
37	>FBgn0037912	NKVWV			
38	>FBgn0031872	NSVGV			
39	>FBgn0041233	NWVYV			
40	>FBgn0037185	PDVQV			
41	>FBgn0037956	PEVHV			
42	>FBgn0014023	PKVGV			
43	>FBgn0050156	QDVPV			
44	>FBgn0058211	QQVRV			
45	>FBgn0043854	RAVEV			
46	>FBgn0050071	RFVTV			
47	>FBgn0053111	RGVDV			
48	>FBgn0004143	RLVKV			
49	>FBgn0031033	SNVLV			
50	>FBgn0030375	SPVLV			

Additional file 4. continued.

nematode xxSxI

No.	gene ID	5aa	No.	gene ID	5aa	No.	gene ID	5aa
1	>ZK863.9	AASQI	51	>Y52E8A.4	KQSEI	101	>F09F3.7	TNSKI
2	>Y39G8B.4	AASWI	52	>SSSD1.1	KQSQI	102	>M199.1	TQSRI
3	>F57G9.4	ADSWI	53	>T07G12.12	KRSGI	103	>F52D2.8	TRSRI
4	>Y51A2A.12	AESAI	54	>Y47D9A.5	LDSTI	104	>R04D3.8	TRSRI
5	>F57G9.1	AESWI	55	>Y94H6A.6	LFSSI	105	>C08G5.5	TSSQI
6	>T21B4.9	AFSQI	56	>ZK1307.1	LFSVI	106	>M04F3.1	TVSAI
7	>ZC518.1	AGSII	57	>F15G9.5	LISFI	107	>C49G7.10	TWSII
8	>F14D2.13	AKSII	58	>C44B12.8	LISKI	108	>Y54E2A.8	TYSKI
9	>F16F9.4	AKSVI	59	>F27E11.3	LISQI	109	>F07G11.5	VASVI
10	>W05H5.6	ANSWI	60	>T06C12.11	LPSII	110	>W10C8.4	VDSFI
11	>Y57A10C.6	AQSKI	61	>T19E7.3	LRSPI	111	>F36H9.4	VFSVI
12	>Y54G11A.5	ARSHI	62	>W06D11.2	LSSPI	112	>W06G6.13	VISVI
13	>W05H5.8	ARSWI	63	>R11A5.6	LVSII	113	>T01C8.5	VKSNI
14	>ZK112.2	ASSQI	64	>F09G8.2	LVSKI	114	>Y45F10A.4	VPSHI
15	>C32C4.5	ATSLI	65	>ZK945.4	NESII	115	>R08H2.4	VQSTI
16	>F14E5.3	CDSRI	66	>W05H5.7	NESWI	116	>W02H5.6	VRSRI
17	>R02C2.1	CHSSI	67	>R53.1	NRSNI	117	>C07G1.8	YASKI
18	>T26C11.8	DNSSI	68	>F15A4.4	NSSII	118	>F10E7.7	YPSNI
19	>H32C10.2	DVSTI	69	>C06B3.10	NTSKI			
20	>Y46G5A.1	DVSVI	70	>T23G7.4	NTSNI			
21	>T04F8.9	EESII	71	>T07H8.5	PASRI			
22	>F21H7.10	EFSMI	72	>F49H6.13	PCSII			
23	>Y45F3A.3	EKSKI	73	>Y87G2A.14	PKSKI			
24	>T22F3.2	EKSMI	74	>ZK384.3	PNSVI			
25	>F23C8.8	EMSEI	75	>T27C5.8	PSSII			
26	>R13D7.4	EMSNI	76	>T22D2.1	PTSTI			
27	>F42G8.5	ENSQI	77	>Y34F4.1	QMSDI			
28	>K03H1.5	ETSAI	78	>Y87G2A.2	QPSKI			
29	>Y39A1A.15	ETSVI	79	>F13G3.8	QRSFI			
30	>Y57G11A.1	EVSDI	80	>Y102E9.6	QTSTI			
31	>C27H5.2	EYSLI	81	>F54H5.4	RHSTI			
32	>Y44E3A.1	FFSEI	82	>K10B3.1	RLSHI			
33	>F37D6.3	FFSSI	83	>Y75B8A.21	RRSSI			
34	>F16F9.3	FLSYI	84	>R02E4.1	RSSSI			
35	>T27C5.12	FRSTI	85	>Y48A6B.9	SASHI			
36	>F13E9.10	FRSVI	86	>ZC518.1	SGSII			
37	>F27C1.10	GESYI	87	>F49C5.1	SISLI			
38	>F25B5.5	GQSSI	88	>F54D11.2	SISLI			
39	>T08G2.3	GTSRI	89	>K09E4.4	SISNI			
40	>C26D10.5	HASCI	90	>T08B1.4	SISSI			
41	>F28G4.4	HESLI	91	>D2089.4	SKSGI			
42	>F13H10.2	HNSRI	92	>Y25C1A.7	SPSPI			
43	>Y53C12A.7	IASLI	93	>Y41E3.12	SRSRI			
44	>C44B9.4	IFSRI	94	>T20F10.1	SSSHI			
45	>C53C7.1	IPSSI	95	>F55A11.5	SSSSI			
46	>C07G3.6	ISSNI	96	>Y17G7B.15	STSDI			
47	>Y59A8B.3	ISSRI	97	>F36D1.12	STSEI			
48	>Y34F4.2	KMSDI	98	>C09H5.4	STSFI			
49	>B0272.4	KPSKI	99	>ZK256.1	SVSGI			
50	>C15B12.1	KPSKI	100	>T11G6.4	TNSFI			

Additional file 4. continued.

nematode xxSxL

No.	gene ID	5aa	No.	gene ID	5aa	No.	gene ID	5aa	No.	gene ID	5aa	No.	gene ID	5aa
1	>C08E8.6	AFSKL	51	>ZK816.4	FSSLL	101	>F16H11.3	KPSIL	151	>T04A8.1	PNSEL	201	>R08C7.6	TASRL
2	>T21E12.4	ANSTL	52	>ZK1320.11	FSSSL	102	>C03B1.14	KRSKL	152	>ZK858.4	PPSGL	202	>F56A11.4	TESVL
3	>C24A3.4	ARSKL	53	>ZC64.3	GCSDL	103	>Y69A2AR.23	KRSPL	153	>F53H8.2	PPSLL	203	>F58G6.9	TGSSL
4	>F59F4.1	ARSKL	54	>C39B10.6	GISKL	104	>Y39A3CL.5	KSSLL	154	>B0495.10	QDSSL	204	>F54C9.2	TISEL
5	>C39E9.10	ASSRL	55	>F26C11.3	GKSEL	105	>E04F6.3	LASKL	155	>C09B8.3	QFSSL	205	>T16H5.1	TKSFL
6	>ZK484.2	ASSYL	56	>Y32B12B.6	GLSLL	106	>F53B2.6	LISNL	156	>F58F9.7	QGSKL	206	>C05D11.13	TLSGL
7	>F38B2.4	ATSKL	57	>Y54G2A.50	GLSRL	107	>T05E7.3	LISNL	157	>R05D3.9	QHSEL	207	>ZK185.1	TRSEL
8	>C30A5.5	AYSFL	58	>T10B11.5	GNSKL	108	>F30A10.8	LISYL	158	>Y37E11B.10	QISCL	208	>Y76A2B.6	TSSSL
9	>ZK795.4	AYSFL	59	>Y32H12A.3	GNSRL	109	>H06I04.1	LNSKL	159	>C06A6.5	QKSEL	209	>F09C6.7	VDSTL
10	>C37E2.4	AYSPL	60	>T26C11.6	GPSHL	110	>K09F5.5	LNSRL	160	>ZK1320.1	QKSKL	210	>F44G3.9	VISFL
11	>C56E6.2	CCSML	61	>F31B9.3	GPSTL	111	>C06B3.1	LRSSL	161	>Y73E7A.3	QLSHL	211	>C10G11.2	VISSL
12	>T08G5.12	CCSVL	62	>M01E11.7	GRSYL	112	>K05F6.6	LTSLL	162	>R09E10.7	QLSPL	212	>C40C9.4	VISVL
13	>D1081.5	CDSQL	63	>C45B11.3	GSSKL	113	>F41B5.8	MASTL	163	>Y39H10B.2	QRSSL	213	>T27E7.1	VKSTL
14	>C10F3.3	CHSTL	64	>Y46E12BL.4	GTSIL	114	>R10D12.17	MESSL	164	>T24C2.3	QSSTL	214	>D2024.4	VNSNL
15	>C56E6.6	CLSYL	65	>F31F6.6	GTSKL	115	>F07G11.7	MFSFL	165	>T05A1.1	QSSYL	215	>T01D3.2	VSSIL
16	>C17C3.20	CPSKL	66	>D2005.6	GVSML	116	>F56C3.3	MFSGL	166	>F28H7.11	QTSCL	216	>C13B7.4	VSSQL
17	>T14G8.3	DASEL	67	>F41E6.5	HCSKL	117	>ZC513.3	MISFL	167	>C17C3.3	QTSKL	217	>Y45G12C.9	VSSQL
18	>T28A11.16	DASKL	68	>F46E10.1	HFSEL	118	>K09H11.1	MKSRL	168	>Y6E2A.6	QTSVL	218	>B0432.4	VTSAL
19	>T02D1.4	DDSFL	69	>F37B12.1	HFSLL	119	>B0491.3	MLSKL	169	>VH15N14R.1	RDSTL	219	>Y39D8B.3	WLSTL
20	>Y6D1A.1	DESAL	70	>C54D2.5	HKSAL	120	>F36H12.11	MLSKL	170	>F58A4.7	RDSWL	220	>F28H1.1	WTSNL
21	>ZC513.10	DESFL	71	>ZC250.2	HKSEL	121	>R13H9.1	MLSKL	171	>C06B8.1	RESKL	221	>M116.1	WTSSL
22	>C36C5.4	DFSGL	72	>F08B4.2	HKSIL	122	>T23B3.3	MLSKL	172	>T05A8.6	RESSL	222	>C11E4.7	YFSIL
23	>C17B7.4	DFSKL	73	>C47B2.5	IESML	123	>F22F7.2	MPSKL	173	>K07C11.4	RHSEL	223	>C50E3.12	YLSKL
24	>C18F10.7	DFSNL	74	>F58G4.4	IFSDL	124	>Y105E8A.7	MRSTL	174	>Y37A1A.3	RKSLL	224	>C28D4.2	YLSNL
25	>D1022.1	DGSEL	75	>T21C12.1	IFSRL	125	>F01G10.3	MSSKL	175	>C14A4.3	RKSNL			
26	>C26F1.3	DHSFL	76	>F13D12.8	ILSEL	126	>C48B4.1	MTSKL	176	>F26G1.1	RLSLL			
27	>F42G9.9	DISSL	77	>H31G24.3	ILSTL	127	>F54E4.1	MTSLL	177	>F54B8.12	RLSNL			
28	>M02B7.1	DKSQL	78	>Y43E12A.3	ILSTL	128	>T21B4.7	NASFL	178	>C02A12.5	RNSSL			
29	>C47E8.4	DLSDL	79	>Y17G9A.1	INSTL	129	>F25E2.4	NDSLL	179	>B0454.10	RPSGL			
30	>C07B5.2	DQSHL	80	>F09E5.2	IQSTL	130	>T11F8.3	NDSLL	180	>B0454.2	RPSNL			
31	>W02A2.5	DTSTL	81	>Y48G1C.4	IRSFL	131	>ZC8.4	NDSNL	181	>F08A8.1	RQSKL			
32	>T05E11.3	EHSEL	82	>K01H12.3	ISSIL	132	>F55D12.2	NESYL	182	>C44E4.8	RRSNL			
33	>F07E5.4	EKSTL	83	>Y22D7AR.1	ISSIL	133	>C24B9.1	NFSLL	183	>ZK550.6	RRSNL			
34	>Y37D8A.2	ELSSL	84	>T06F4.2	KASFL	134	>F59A1.11	NHSTL	184	>C53D6.2	SCSCL			
35	>Y45F3A.1	ELSSL	85	>T14E8.2	KASYL	135	>T02H6.5	NISAL	185	>K02A4.2	SCSVL			
36	>B0395.3	EMSKL	86	>F08B6.2	KCSML	136	>H11E01.3	NISSL	186	>T15B7.7	SFSDL			
37	>D2021.4	ENSKL	87	>K10F12.3	KDSVL	137	>F58A6.10	NKSKL	187	>R04B5.7	SFSSL			
38	>C17C3.1	EPSKL	88	>T03E6.3	KESKL	138	>R05H5.4	NKSRL	188	>C24H10.2	SGSGL			
39	>F11C3.1	EQSLL	89	>F47H4.11	KFSRL	139	>F59D12.1	NNSFL	189	>F08C6.4	SGSRL			
40	>K09C8.8	ERSVL	90	>E02H9.1	KGSAL	140	>W08A12.4	NNSQL	190	>F25E5.12	SISRL			
41	>Y53F4B.39	ESSNL	91	>W09C5.7	KISLL	141	>C31H5.6	NRSRL	191	>K06B4.7	SNSLL			
42	>K07A12.5	ETSGL	92	>F43H9.1	KKSKL	142	>C47A10.5	NSSKL	192	>ZK867.1	SRSPL			
43	>Y111B2A.15	EVSKL	93	>F53C11.3	KKSKL	143	>T07D10.1	NSSKL	193	>T12B3.3	SSSDL			
44	>C16A3.10	FCSAL	94	>M03A8.1	KKSKL	144	>C09H5.3	NSSNL	194	>C07A9.9	SSSSL			
45	>T07H8.4	FGSFL	95	>W03D8.8	KKSKL	145	>W03A5.3	NSSNL	195	>Y39B6A.38	SSSTL			
46	>K12C11.3	FGSRL	96	>Y69A2AR.5	KKSKL	146	>F20D1.6	NSSYL	196	>C06A8.7	SSSWL			
47	>Y38F2AL.6	FHSSL	97	>Y71F9AL.9	KLSIL	147	>F20E11.1	NYSTL	197	>T08A9.4	STSKL			
48	>C09B8.4	FISKL	98	>F55D10.1	KMSKL	148	>F58A6.1	PFSKL	198	>F17A2.10	SVSRL			
49	>K07C5.5	FKSHL	99	>C02A12.6	KNSRL	149	>T02G6.5	PHSSL	199	>F23C8.13	SYSLL			
50	>C05E4.6	FSSAL	100	>F46E10.3	KNSYL	150	>F35G2.2	PLSKL	200	>C28F5.1	TASLL			

Additional file 4. continued.

nematode xxSxV

No.	gene ID	5aa	No.	gene ID	5aa	No.	gene ID	5aa	No.	gene ID	5aa
1	>W03B1.4	AKSLV	51	>K08F4.7	KDSIV	101	>F29B9.4	QNSLV	151	>H12C20.6	YDSDV
2	>K10C9.6	AVSTV	52	>F18H3.4	KDSPV	102	>K08F8.7	QTSSV	152	>Y110A7A.17	YGSPV
3	>F07B10.4	CKSEV	53	>C52B9.6	KESDV	103	>T14G10.2	QVSRV	153	>C08H9.1	YTSPV
4	>K09C8.5	CKSQV	54	>F01F1.3	KESIV	104	>Y44A6D.6	RDSLV			
5	>F28B12.3	CSSEV	55	>K03D7.9	KESSV	105	>C34H4.4	RFSHV			
6	>F56F3.3	CSSTV	56	>K11B4.1	KESTV	106	>C16D9.2	RISQV			
7	>C07A9.3	CVSNV	57	>Y81G3A.5	KFSVV	107	>T02C5.3	RMSIV			
8	>Y105C5B.21	DDSWV	58	>F55G1.5	KGSRV	108	>Y47G6A.24	RMSPV			
9	>C04E12.2	DFSNV	59	>T28D9.7	KHSYV	109	>C48D1.2	RNSAV			
10	>C05B5.7	DISMV	60	>C02H7.2	KISAV	110	>K04F10.6	RNSFV			
11	>Y54G2A.1	DRSRV	61	>ZC132.8	KLSKV	111	>F32H2.6	RNSIV			
12	>T27E9.3	DTSDV	62	>ZC132.3	KLSNV	112	>F28C1.3	RNSPV			
13	>F13A2.8	DVSGV	63	>Y113G7B.9	KNSEV	113	>F13H6.5	RPSNV			
14	>C09B7.1	EASDV	64	>F13D12.10	KPSYV	114	>R13A5.1	RPSNV			
15	>M02F4.1	EGSFV	65	>T26C12.3	KQSLV	115	>F52H3.2	RSSAV			
16	>F54D12.10	EISNV	66	>F49E8.3	KQSNV	116	>C29E4.8	RVSFV			
17	>F53A2.6	EKSAV	67	>T10H9.6	LCSPV	117	>T08G3.12	RVSIV			
18	>T21H8.1	ESSNV	68	>C27H6.3	LESSV	118	>F43E2.3	SESAV			
19	>T08H4.1	ETSDV	69	>ZC317.2	LISTV	119	>R107.6	SHSHV			
20	>F29D11.1	EVSDV	70	>Y57A10A.30	LKSIV	120	>C48E7.11	SSSAV			
21	>C24D10.9	EVSLV	71	>B0198.2	LLSSV	121	>Y54G2A.16	SSSVV			
22	>F28G4.3	FFSRV	72	>K09H11.6	LPSFV	122	>F59H6.4	STSQV			
23	>C02H7.1	FISNV	73	>R03H10.2	LQSEV	123	>F01E11.5	STSRV			
24	>Y105C5B.11	FPSLV	74	>F49F1.14	LRSDV	124	>F47C12.11	SVSGV			
25	>C35D10.15	FQSSV	75	>T11B7.3	LRSNV	125	>F26G5.10	THSLV			
26	>Y42H9B.2	FSSFV	76	>Y60A3A.19	LYSGV	126	>W02D7.8	TISLV			
27	>M02H5.12	FVSPV	77	>ZK1055.4	MKSFV	127	>Y94A7B.6	TKSFV			
28	>F56A6.2	FYSPV	78	>Y53C12B.1	MRSSV	128	>ZC196.1	TKSIV			
29	>Y50D4C.5	GASFV	79	>Y37E11AL.5	MVSIV	129	>C27D8.2	TRSGV			
30	>F32H2.4	GHSTV	80	>K02E10.5	NDSSV	130	>Y65B4BL.2	TSSRV			
31	>W06B11.3	GPSMV	81	>T11B7.2	NDSTV	131	>ZK673.4	TTSEV			
32	>F59B2.13	GTSFV	82	>T07C12.6	NDSWV	132	>B0250.8	TTSSV			
33	>ZK849.5	HDSKV	83	>B0410.2	NESAV	133	>R07B1.3	TVSVV			
34	>R07D5.2	HNSVV	84	>R07E4.1	NESLV	134	>W02B3.2	VDSGV			
35	>Y79H2A.6	HPSLV	85	>F42G2.5	NFSTV	135	>F54H12.2	VDSIV			
36	>R193.1	HPSSV	86	>Y82E9BL.13	NISRV	136	>K09F6.5	VDSIV			
37	>H23L24.4	HWSFV	87	>R160.4	NNSRV	137	>ZK892.1	VGSFV			
38	>W04A8.2	IDSIV	88	>Y87G2A.9	PASEV	138	>F15B9.4	VGSPV			
39	>T05H4.2	IDSQV	89	>F37B1.1	PDSVV	139	>F57G8.7	VISSV			
40	>F26E4.1	IFSDV	90	>F25F8.2	PESLV	140	>C14E2.2	VKSNV			
41	>F26E4.5	IGSAV	91	>K06B4.12	PESQV	141	>K08H2.8	VLSGV			
42	>C17F4.10	IHSTV	92	>T01C1.2	PISLV	142	>F09F3.2	VNSPV			
43	>T12A7.1	INSYV	93	>T26C5.4	PLSVV	143	>ZK262.6	VNSTV			
44	>Y39A1C.1	IQSQV	94	>ZK377.1	PQSFV	144	>H17B01.3	VQSLV			
45	>K12D9.10	IVSPV	95	>F25B3.6	PSSAV	145	>F56C9.8	VQSSV			
46	>C27D9.2	IWSNV	96	>B0348.4	PTSVV	146	>Y10G11A.3	VRSIV			
47	>C34C6.6	KASLV	97	>Y38F1A.4	QESEV	147	>F53H2.1	VSSDV			
48	>C28H8.9	KASRV	98	>F10D2.1	QESQV	148	>Y39C12A.1	VSSHV			
49	>T01H8.5	KCSDV	99	>F27D9.1	QKSLV	149	>ZK1128.7	VTSIV			
50	>C56G3.1	KDSFV	100	>F28F5.3	QLSKV	150	>C14B1.4	WRSDV			

Additional file 4. continued.

nematode xxTxI

No.	gene ID	5aa	No.	gene ID	5aa
1	>C33D9.4	AQTQI	51	>C24G6.6	PITSI
2	>Y55D5A.4	ASTFI	52	>M01G5.6	PPTAI
3	>M01D7.9	CKTRI	53	>F37B1.2	PVTEI
4	>T24D5.4	CLTMI	54	>F37B1.3	PVTEI
5	>Y38C1AA.9	CQTPI	55	>ZK1010.6	QNTYI
6	>C25E10.13	CSTQI	56	>F57C9.6	QPTDI
7	>C06G8.2	DHTRI	57	>Y32B12C.2	QPTII
8	>F58G4.6	DKTKI	58	>F52E1.13	RCTKI
9	>B0280.4	DVTTI	59	>M01G5.5	RETAI
10	>F14F9.5	DYTFI	60	>K04G7.11	RGTAI
11	>C14A6.3	ENTKI	61	>E02H9.9	RGTHI
12	>H02I12.3	EPTAI	62	>F09E5.9	RSTGI
13	>H09F14.1	ESTAI	63	>C24H12.1	RTTLI
14	>C27A7.5	ETTLI	64	>T19E10.1	RVTDI
15	>T03F7.3	ETTYI	65	>F20B4.6	SQTSI
16	>T03F7.4	ETTYI	66	>C09G9.8	SVTVI
17	>C17H11.1	EVTQI	67	>K04A8.6	TGTEI
18	>C27H2.2	EVTRI	68	>C24H12.8	TKTRI
19	>F53F8.1	FMTFI	69	>B0207.12	TNTGI
20	>C17E7.2	GITVI	70	>Y54G9A.10	TNTWI
21	>T22D1.1	GNTYI	71	>F28A10.2	TQTHI
22	>F44F1.7	GTTGI	72	>Y50D4C.4	TQTLI
23	>C09H10.6	GTTSI	73	>F16B3.1	TRTRI
24	>F21D9.8	HITLI	74	>C06E7.7	TVTRI
25	>F28G4.2	HYTLI	75	>F32H2.11	VATYI
26	>F59E12.3	ICTGI	76	>F35H12.5	VDTKI
27	>F12B6.2	IFTKI	77	>R119.6	VDTTI
28	>F14F8.10	IQTQI	78	>W04D2.3	VITNI
29	>T04F8.2	IRTQI	79	>C27H5.6	VNTAI
30	>C08B6.12	ITTVI	80	>F45E6.1	VPTRI
31	>C37H5.3	IVTPI	81	>K12F2.2	VTTFI
32	>Y48G1C.9	KETEI	82	>T19H5.4	WTTSI
33	>C25D7.6	KITLI	83	>F53C3.4	YFTRI
34	>ZC196.7	KKTTI	84	>C50B8.5	YSTLI
35	>F52H2.3	KQTEI	85	>F42F12.11	YSTTI
36	>ZK783.2	KQTGI			
37	>C06B3.3	KVTKI			
38	>D2013.9	KVTPI			
39	>Y55B1AR.4	KVTQI			
40	>F27C1.7	LATAI			
41	>F56C4.2	LETSI			
42	>K01C8.3	LQTPI			
43	>H12D21.4	NATGI			
44	>C05D11.9	NATKI			
45	>B0303.9	NITNI			
46	>C18H9.6	NLTFI			
47	>Y116F11B.11	NQTAI			
48	>C45H4.3	NVTVI			
49	>E03H4.4	NYTII			
50	>R186.5	PHTLI			

Additional file 4. continued.

nematode xxTxL

No.	gene ID	5aa	No.	gene ID	5aa	No.	gene ID	5aa	No.	gene ID	5aa
1	>F54G8.3	ADTGL	51	>F43G6.1	IPTQL	101	>ZK180.1	PSTKL	151	>F18A1.3	VMTTL
2	>F57C9.2	AITVL	52	>T04B8.5	ISTAL	102	>F45H11.4	QATPL	152	>R05H5.1	VQTQL
3	>W03H9.1	AQTEL	53	>Y27F2A.3	ISTVL	103	>K06A1.6	QETHL	153	>C03G6.7	VSTVL
4	>C45E5.6	AQTTL	54	>F57A10.1	ITTKL	104	>F18F11.3	QSTEL	154	>ZK185.4	VTTEL
5	>Y2H9A.2	ATTVL	55	>T23D5.11	ITTKL	105	>F07C3.1	QSTKL	155	>K05C4.6	YDTDL
6	>Y32F6B.3	CCTIL	56	>ZC155.7	IVTKL	106	>T24F1.3	QSTNL	156	>Y41D4B.24	YETLL
7	>Y76B12C.4	CCTIL	57	>C37H5.2	IVTPL	107	>F30F8.3	QVTRL	157	>F31D5.3	YVTVL
8	>C05C10.4	CKTPL	58	>C09G12.8	KCTVL	108	>F52D10.2	QVTSL			
9	>Y54E5B.1	CRTSL	59	>ZK1067.1	KETCL	109	>R07B5.5	RATLL			
10	>F40A3.1	DCTNL	60	>F59C12.2	KETFL	110	>K08H10.2	RCTIL			
11	>ZC84.4	DETLL	61	>Y59E1B.2	KFTDL	111	>Y66D12A.8	RGTLL			
12	>C09H6.3	DETVL	62	>H06O01.1	KKTEL	112	>T11F1.7	RGTNL			
13	>F18A12.3	DGTSL	63	>C32A9.1	KLTFL	113	>M03A8.3	RHTDL			
14	>B0403.4	DKTEL	64	>R07E3.3	KSTML	114	>F55D10.3	RITNL			
15	>C30H7.2	DKTEL	65	>F07B10.2	KSTQL	115	>F45E4.7	RRTCL			
16	>F35G12.3	DPTDL	66	>F26G5.4	KVTRL	116	>T04B2.7	RSTLL			
17	>C04C3.6	DRTKL	67	>C26C6.8	LATDL	117	>T23B12.1	RTTKL			
18	>Y41D4B.19	ECTRL	68	>C14C6.8	LFTKL	118	>F10A3.7	SATML			
19	>F56B6.5	EGTKL	69	>K06H6.4	LFTKL	119	>C49G7.12	SETLL			
20	>B0238.13	EGTRL	70	>F07C3.4	LGTQL	120	>T08D10.1	SFTNL			
21	>F14B8.7	EHTPL	71	>C28A5.2	LHTYL	121	>Y102A5C.40	SGTEL			
22	>Y49E10.4	EKTEL	72	>F59A6.3	LPTRL	122	>T03G11.6	SHTDL			
23	>F28F9.4	EKTRL	73	>F35G8.1	LRTDL	123	>F48E3.3	SHTEL			
24	>Y37E3.13	ELTHL	74	>C07E3.4	LSTEL	124	>C56A3.3	SITAL			
25	>T02D1.6	EQTHL	75	>F57H12.5	LSTEL	125	>C49A9.3	SITKL			
26	>F18H3.1	EQTIL	76	>F31B9.1	LSTLL	126	>R03G5.7	SNTFL			
27	>F41H10.4	ERTGL	77	>C55F2.1	LVTNL	127	>T04B2.5	SNTVL			
28	>F31C3.5	ESTPL	78	>C48A7.1	LVTTL	128	>M195.2	SPTSL			
29	>ZK669.1	ESTRL	79	>Y95B8A.6	LVTYL	129	>F58E2.9	SQTQL			
30	>C02E7.9	ETTRL	80	>F42A10.3	MATKL	130	>F26G5.9	SQTRL			
31	>R01E6.2	FCTEL	81	>Y45F10B.10	MCTLL	131	>M05D6.7	SQTSL			
32	>F25E2.1	FETRL	82	>T20B3.5	MITNL	132	>Y9C9A.10	SSTQL			
33	>K11D12.12	FGTFL	83	>T06H11.5	MKTCL	133	>C11G6.4	STTQL			
34	>F21E9.4	FVTLL	84	>C07G3.4	MNTGL	134	>C30D11.1	TDTIL			
35	>C07A12.4	GHTEL	85	>R01H10.3	MRTTL	135	>C07D10.2	TETNL			
36	>T23E7.6	GITDL	86	>K03D3.10	NCTVL	136	>M01G12.14	TETTL			
37	>K05D4.6	GITPL	87	>F57G8.3	NETKL	137	>K08C7.6	TFTSL			
38	>F59E11.13	GRTTL	88	>ZK1127.5	NKTIL	138	>T14F9.3	TKTEL			
39	>F46C3.3	GSTEL	89	>C06G4.5	NPTFL	139	>F55A3.2	TLTCL			
40	>F14H8.6	GVTVL	90	>C25B8.4	NPTRL	140	>F48E8.3	TRTEL			
41	>Y53G8AR.3	HCTIL	91	>Y4C6A.2	NSTFL	141	>T05E11.2	TSTSL			
42	>ZC506.4	HDTFL	92	>C18B10.5	NSTHL	142	>F56A4.1	TTTKL			
43	>C07B5.4	HVTRL	93	>B0563.6	NTTFL	143	>Y19D10A.6	TTTKL			
44	>ZC247.1	ICTML	94	>C40H5.5	NYTHL	144	>B0507.11	TTTTL			
45	>T20F7.5	IETIL	95	>Y53F4B.37	PDTVL	145	>C05C10.8	TWTFL			
46	>F45C12.13	IITFL	96	>F47A4.5	PFTDL	146	>T01C3.10	VETNL			
47	>T24H7.2	IKTEL	97	>F53B2.3	PGTPL	147	>H25K10.5	VFTYL			
48	>F49E7.1	IKTIL	98	>Y71F9B.8	PITRL	148	>K03H6.2	VITIL			
49	>C07G3.5	INTGL	99	>K05D4.2	PMTCL	149	>H06H21.10	VITRL			
50	>C56G7.3	INTLL	100	>Y53H1C.1	PMTRL	150	>F25H2.9	VITSL			

Additional file 4. continued.

nematode xxTxV

No.	gene ID	5aa	No.	gene ID	5aa
1	>C05B10.1	AETAV	51	>F21H7.1	PVTLV
2	>Y48E1B.12	AGTSV	52	>T22F7.1	QETKV
3	>F58H1.5	AMTYV	53	>F39C12.2	QETYV
4	>Y70C5A.2	ATTTV	54	>K03H6.5	QSTQV
5	>C08G5.3	ATTWV	55	>T28B4.2	QTTMV
6	>Y67A10A.9	DETTV	56	>E03H12.6	QTTRV
7	>F46H5.4	DPTVV	57	>F54B3.3	RETAV
8	>K11C4.4	DSTHV	58	>ZK697.1	RETTV
9	>Y53H1B.2	EETDV	59	>F16A11.1	RHTQV
10	>F09B9.1	ENTKV	60	>F38E9.5	RITKV
11	>T01B11.1	EYTTV	61	>C51E3.1	RMTLV
12	>C56C10.11	FLTDV	62	>C51E3.2	RMTVV
13	>Y42A5A.3	FLTPV	63	>Y38F2AL.1	SETCV
14	>K04D7.4	GGTRV	64	>F09G8.4	SETSV
15	>F26F4.10	GKTCV	65	>F13H10.1	SETSV
16	>R09A1.1	GMTFV	66	>T14E8.1	SETSV
17	>ZK563.2	GQTQV	67	>C34B4.3	SGTPV
18	>C48B4.4	HSTHV	68	>W08F4.12	SITFV
19	>T16H12.9	HTTPV	69	>F09F7.7	SKTSV
20	>C26C6.4	HYTLV	70	>K12H4.8	SLTTV
21	>F43G6.4	IDTSV	71	>Y24D9A.1	SSTNV
22	>R10D12.2	IRTFV	72	>F13E9.14	STTTV
23	>C32D5.7	ITTFV	73	>C50F7.6	SVTTV
24	>F20D1.9	KATHV	74	>F46F11.9	SYTVV
25	>F33G12.6	KATNV	75	>F07C6.4	TATIV
26	>C25B8.8	KETIV	76	>C47E12.13	TETAV
27	>F58A3.2	KETIV	77	>C10A4.8	THTNV
28	>C30E1.7	KITFV	78	>T28H10.2	TKTSV
29	>F45F2.11	KVTDV	79	>Y54E10BR.7	TLTPV
30	>T10D4.6	LETDV	80	>F44A2.5	TRTEV
31	>B0205.9	LFTVV	81	>C18F3.2	TSTFV
32	>F54D1.6	LKTSV	82	>K02E10.8	TSTHV
33	>R09E10.5	LNTSV	83	>D2092.6	TSTNV
34	>C48B6.9	LSTLV	84	>Y46G5A.10	TTTAV
35	>T22C1.4	LSTVV	85	>C18H9.7	TVTDV
36	>K04G2.8	LVTIV	86	>Y48A6B.11	TVTDV
37	>Y73B6BR.1	LYTSV	87	>ZK675.4	TVTKV
38	>F01G4.3	LYTTV	88	>C09H6.1	TVTTV
39	>F37B1.4	MDTEV	89	>F59A7.11	VETTV
40	>C14A4.9	MGTAV	90	>W02D7.7	VRTMV
41	>W08D2.3	MPTRV	91	>B0240.2	VTTVV
42	>C09F5.1	NATLV	92	>C08F8.9	WATVV
43	>C07B5.6	NDTKV	93	>F55B11.1	WITSV
44	>C39E6.6	NDTLV	94	>C16E9.4	YFTFV
45	>F35D2.3	NGTDV	95	>Y37H2A.7	YITKV
46	>Y40H7A.4	NLTVV	96	>C06E1.4	YNTAV
47	>F23F12.10	NVTLV	97	>R10D12.1	YQTKV
48	>F45H7.2	PETNV	98	>F44E7.9	YSTQV
49	>M110.8	PPTAV			
50	>ZK563.4	PVTEV			

nematode xxVxI

No.	gene ID	5aa	No.	gene ID	5aa
1	>C27A12.4	ADVSI	51	>F46G10.6	MAVKI
2	>C47C12.6	AFVSI	52	>C36C5.9	MDVKI
3	>F17A2.9	ASVMI	53	>C33F10.13	MFVWI
4	>C41G6.10	ATVII	54	>T26H2.10	MNVVI
5	>F16F9.3	DDVKI	55	>F09C3.3	MYVRI
6	>ZK616.6	DEVFI	56	>C43F9.9	NCVQI
7	>Y113G7A.12	DFVAI	57	>Y48E1B.9	NDVKI
8	>F43H9.3	DKVII	58	>C31B8.11	NIVFI
9	>T11G6.5	DPVTI	59	>T12A2.1	NVVDI
10	>T20B12.1	DSVAI	60	>T14C1.1	PEVLI
11	>W03F9.2	DTVGI	61	>F23H11.4	PLVYI
12	>Y4C6B.4	DVVKI	62	>F38E1.8	QLVLI
13	>F07A11.4	EEVKI	63	>H19N07.4	QTVGI
14	>T25D10.4	ESVVI	64	>F38C2.1	RFVQI
15	>B0205.1	FAVSI	65	>C24G7.1	RSVLI
16	>F42G8.10	FDVLI	66	>W09G3.1	SAVVI
17	>Y82E9BR.7	FFVVI	67	>T14G10.2	SDVLI
18	>Y46H3D.5	FTVPI	68	>Y57G11C.42	SGVRI
19	>F33E2.2	GAVRI	69	>F21A3.2	SHVEI
20	>F49H6.3	HDVNI	70	>K02C4.3	SLVKI
21	>B0212.2	HSVVI	71	>F59B1.1	SRVDI
22	>Y53F4B.16	IGVSI	72	>T23B3.4	SSVHI
23	>B0546.5	ILVII	73	>C43H8.1	VIVDI
24	>D2024.5	INVEI	74	>ZC239.6	VPVRI
25	>R10F2.1	IQVEI	75	>F47C10.4	VSVII
26	>C39H7.9	IVVKI	76	>Y41C4A.11	VTVEI
27	>F16D3.2	KDVFI	77	>C54H2.1	VYVHI
28	>C03G6.15	KFVKI	78	>Y41C4A.17	YDVHI
29	>C49G7.8	KFVKI	79	>R13.2	YPVSI
30	>K07C6.5	KFVKI	80	>W04A8.1	YSVII
31	>K09D9.2	KFVKI	81	>Y20F4.5	YSVQI
32	>F01F1.13	KFVMI	82	>R02E12.6	YSVTI
33	>K06B4.10	KFVSI			
34	>T23B12.5	KGVRI			
35	>C25H3.11	KKVSI			
36	>Y69A2AR.9	KKVWI			
37	>K04G2.6	KNVKI			
38	>C04E12.11	KPVKI			
39	>F25H2.6	KPVSI			
40	>C16C8.18	KSVKI			
41	>F45D3.1	KSVKI			
42	>F56D2.3	KTVEI			
43	>C01H6.4	KVVDI			
44	>F55B12.5	KYVII			
45	>T19H5.7	LCVNI			
46	>Y48B6A.9	LIVVI			
47	>Y102A5C.27	LNVHI			
48	>R02C2.6	LNVSI			
49	>ZK1290.5	LPVCI			
50	>F14F9.7	LSVII			

Additional file 4. continued.

nematode xxVxV

No.	gene ID	5aa	No.	gene ID	5aa
1	>F53G12.6	AAVAV	51	>ZK970.5	MRVEV
2	>C48C5.3	AAVYV	52	>F09C3.5	MYVGV
3	>F40H6.4	AFVEV	53	>W10G6.2	NRVLV
4	>D1069.4	APVGV	54	>F39B2.8	NSVRV
5	>F49A5.10	ARVEV	55	>Y54E2A.1	PAVVV
6	>F47D2.9	AVVTV	56	>C38H2.2	PEVAV
7	>T16D1.1	CFVFV	57	>F54D11.4	PPVMV
8	>K09H9.5	CPVSV	58	>C36B1.12	PPVNV
9	>Y67H2A.7	DAVTV	59	>F28A10.5	PPVTV
10	>R09H3.1	DIVIV	60	>C35D10.6	PRVVV
11	>F15A8.4	DKVDV	61	>T28A8.1	QEVHV
12	>F25H5.7	DKVKV	62	>ZK1193.5	QKVFV
13	>T19D2.3	DLVKV	63	>C44B12.6	QLVLV
14	>F54E7.5	DPVFV	64	>F22F7.3	QYVMV
15	>R06A4.10	DTVRV	65	>T22A3.3	RNVPV
16	>F10D11.4	EKVEV	66	>Y39C12A.6	RVVLV
17	>E03H4.5	ENVFV	67	>T01C3.8	SAVPV
18	>M02F4.2	ENVFV	68	>F53A10.2	SDVSV
19	>Y82E9BL.5	EVVEV	69	>F17C11.1	SDVVV
20	>K09E4.6	FAVSV	70	>C25D7.10	SRVPV
21	>F37B4.3	FNVVV	71	>ZK673.10	SRVSV
22	>Y73C8C.8	FPVLV	72	>K01B6.2	SSVHV
23	>F36F12.7	FSVIV	73	>K04H4.2	SSVSV
24	>W03F11.4	FSVMV	74	>K04F1.5	STVVV
25	>H19M22.2	GAVSV	75	>Y51A2D.21	SVVDV
26	>T23F6.1	GDVMV	76	>C08F11.13	SVVTV
27	>M01F1.8	GEVDV	77	>F11F1.8	TPVNV
28	>K07A1.13	GFVQV	78	>T25E12.4	TPVYV
29	>R01H10.6	IHVDV	79	>R166.5	TQVNV
30	>T13F3.1	IHVTV	80	>Y44A6B.2	TTVLV
31	>Y39H10A.7	INVDV	81	>F22E5.13	VFVPV
32	>F52E1.14	KIVGV	82	>F15H9.1	VIVFV
33	>T06E4.14	KKVSV	83	>C52B9.7	VSVKV
34	>C55B7.3	KKVVV	84	>H22D14.1	VSVSV
35	>C23H3.2	KLVEV	85	>ZC132.7	VVVTV
36	>F56H9.4	KMVGV	86	>H36L18.2	YAVLV
37	>Y116A8C.29	KPVFV	87	>C54D2.2	YFVIV
38	>M176.10	KRVKV	88	>F54E2.2	YNVCV
39	>Y77E11A.2	KTVIV	89	>B0462.4	YRVLV
40	>F54B11.3	KVVQV	90	>B0336.6	YVVPV
41	>Y56A3A.17	LEVAV			
42	>T27E9.1	LFVVV			
43	>D2096.2	LNVKV			
44	>Y32H12A.7	LNVLV			
45	>T21D12.9	LPVQV			
46	>F44G4.6	LPVTV			
47	>ZK20.1	LRVLV			
48	>T21C9.11	LSVVV			
49	>Y38C1AA.8	LVVQV			
50	>T21B4.14	LVVRV			
