# Supplementary material for: Evolutionarily conserved bias of amino-acid usage refines the definition of PDZ-binding motif
Source: BMC Genomics. 2011 Jun 8;12:300. doi: 10.1186/1471-2164-12-300 (PMC3138430; doi:10.1186/1471-2164-12-300)
Supplement: Additional file 5 — The number of genes that encode proteins possessing three-position-specified PB motifs located at C0 to C50 positions. [file 1471-2164-12-300-S5.PDF]

Additional file 5.

The number of genes that encode proteins possessing three-position-specified PB motifs located at C0 to C50 positions.

HUMAN x-x-S-x-I

five-amino-acid-long motif, positions 0 and -2 are already specified

Example

species

position -4

	C50	C49	C48	C47	C46	C45	C44	C43	C42	C41	C40	C39	C38	C37	C36	C35	C34	C33	C32	C31	C30	C29	C28	C27	C26	C25	C24	C23	C22	C21	C20	C19	C18	C17	C16	C15	C14	C13	C12	C11	C10	C9	C8	C7	C6	C5	C4	C3	C2	C1	C0	C-index	SD	*P*-value	mark	
A	5	7	9	1	8	[illegible]	[illegible]	[illegible]	[illegible]	[illegible]	8	7	6	9	3	4	3	7	15	10	6	6	9	3	2	5	3	6	8	9	6	3	5	7	7	10	4	7	7	8	5	5	8	2	2	3	1	2	5	5	9	0.618	0.317	8.27E-03	n.s.	A
C	3	1	2	0	2	[illegible]	[illegible]	[illegible]	[illegible]	[illegible]	1	7	4	9	1	1	4	3	1	5	1	1	1	1	0	1	0	3	3	2	2	2	2	3	0	3	0	3	4	3	0	4	5	1	3	1	5	4	4	5	3	0.807	0.628	–	–	C
D	5	5	1	9	2	8	[illegible]	[illegible]	[illegible]	[illegible]	1	6	2	6	4	6	3	4	0	6	6	4	4	6	7	6	5	7	3	7	2	4	4	8	0	5	7	6	4	4	2	3	2	5	4	4	3	6	5	2	2	2.170	1.038	–	–	D
E	2	10	11	13	4	9	10	11	5	8	7	12	5	5	4	4	3	11	3	6	3	8	3	8	7	5	5	3	5	4	5	3	9	8	2	8	7	5	5	7	11	9	3	2	9	7	7	10	7	7	14	0.464	0.209	5.55E-08	*	E
F	3	6	2	3	1	7	6	3	5	6	12	3	9	3	5	8	9	1	9	7	3	11	3	7	7	6	2	9	1	8	2	5	3	6	4	0	1	4	6	5	10	9	10	6	3	4	1	6	6	4	3	1.733	0.985	–	–	F
G	2	3	8	7	1	5	8	2	5	8	5	2	3	5	10	3	4	4	7	7	6	9	6	7	4	6	5	4	2	1	3	6	4	2	4	10	3	7	4	3	4	4	8	6	7	5	6	8	8	6	9	0.571	0.256	5.63E-05	*	G
H	2	3	3	3	2	2	3	1	3	3	3	5	3	5	2	1	2	2	3	4	0	2	4	2	2	6	3	1	3	1	10	2	2	1	6	3	4	1	3	3	1	10	1	2	1	3	2	2	2	5	3	0.953	0.656	–	–	H
I	3	4	3	4	2	6	6	8	7	6	5	2	6	7	5	6	4	4	7	8	6	9	5	3	2	7	4	2	4	6	5	6	4	7	6	6	9	7	4	7	2	8	9	8	8	6	7	5	4	5	4	1.370	0.493	–	–	I
K	10	6	3	6	1	9	5	0	6	6	7	3	2	6	26	2	6	7	2	11	4	10	5	9	2	3	4	6	4	13	3	12	6	7	8	5	5	12	9	12	7	3	6	5	8	3	13	6	6	5	12	0.542	0.354	2.12E-06	*	K
L	13	14	8	8	13	13	17	6	6	7	9	13	7	7	12	11	13	11	11	11	8	11	17	14	15	7	17	17	12	10	26	8	10	19	16	16	15	9	14	13	5	13	22	14	11	15	14	9	8	7	16	0.753	0.268	9.81E-01	n.s.	L
M	4	2	0	6	3	6	2	1	1	3	5	2	4	2	2	2	2	1	2	1	1	2	5	1	7	1	1	2	1	0	4	3	1	1	3	3	4	2	1	2	4	4	3	4	0	2	1	3	4	0	0	–	–	–	–	M
N	4	3	2	4	13	6	2	4	5	6	12	3	5	6	3	7	7	8	6	4	4	3	2	3	4	0	4	1	1	2	1	4	4	4	5	4	4	3	2	8	5	4	2	3	7	2	2	2	3	5	1	4.160	2.518	–	–	N
P	7	3	9	7	4	3	7	5	5	8	7	6	8	7	5	7	8	5	1	5	9	8	4	10	4	6	4	9	9	10	2	6	4	2	6	9	11	5	6	11	7	5	4	6	4	8	4	8	5	8	7	0.889	0.338	–	–	P
Q	8	2	4	6	4	5	4	5	1	3	6	3	4	6	4	4	3	3	4	4	8	4	7	1	2	3	6	2	5	12	1	6	2	2	10	5	1	10	6	4	3	6	9	11	4	5	3	6	5	2	6	0.780	0.434	8.88E-01	n.s.	Q
R	6	9	6	2	5	3	5	5	0	5	2	7	4	3	3	5	3	5	8	5	9	7	8	5	7	5	6	5	5	7	7	7	6	6	9	5	3	8	5	6	6	6	8	10	7	2	5	7	8	6	12	0.470	0.173	5.55E-08	*	R
S	7	15	10	9	14	13	12	11	12	16	11	12	8	13	12	7	5	10	9	9	9	[illegible]	[illegible]	[illegible]	[illegible]	[illegible]	[illegible]	[illegible]	[illegible]	[illegible]	[illegible]	[illegible]	[illegible]	[illegible]	[illegible]	[illegible]	[illegible]	[illegible]	[illegible]	[illegible]	[illegible]	[illegible]	[illegible]	[illegible]	[illegible]	10	12	12	14	11	16	0.620	0.214	1.67E-05	*	S
T	9	10	8	9	10	8	4	12	10	6	8	9	7	6	8	5	3	4	7	8	[illegible]	[illegible]	[illegible]	[illegible]	[illegible]	[illegible]	[illegible]	[illegible]	[illegible]	[illegible]	[illegible]	[illegible]	[illegible]	[illegible]	[illegible]	[illegible]	[illegible]	[illegible]	[illegible]	[illegible]	[illegible]	[illegible]	[illegible]	[illegible]	[illegible]	3	5	6	8	5	5	1.312	0.500	–	–	T
V	3	9	9	11	3	13	4	8	8	4	8	8	4	4	3	8	5	5	8	11	7	[illegible]	[illegible]	[illegible]	[illegible]	[illegible]	[illegible]	[illegible]	[illegible]	[illegible]	[illegible]	[illegible]	[illegible]	[illegible]	[illegible]	[illegible]	[illegible]	[illegible]	[illegible]	[illegible]	[illegible]	[illegible]	[illegible]	[illegible]	[illegible]	8	8	6	5	0	5	1.224	0.558	–	–	V
W	2	1	0	2	0	0	0	1	2	3	1	0	1	1	1	4	0	3	3	0	0	1	1	0	4	2	2	1	0	0	0	0	1	3	0	0	1	1	0	0	2	2	1	1	2	1	1	0	7	3	1	1.240	1.408	–	–	W
Y	1	0	4	1	5	1	3	1	6	1	3	3	5	2	6	3	2	0	1	0	2	1	6	3	1	1	1	3	2	1	2	1	1	1	1	2	2	1	2	4	1	1	5	6	2	2	6	0	3	2	6	0.380	0.294	7.18E-08	*	Y
total	99	113	102	111	97	124	110	92	92	108	121	113	97	112	119	98	89	98	107	122	95	106	107	102	101	83	97	97	88	111	105	105	85	110	114	113	96	109	106	118	103	125	127	120	99	94	106	108	117	93	134	0.786	0.080			total

the number of genes that encode proteins possessing R-x-S-x-I motif at C35 position

position -3

	C50	C49	C48	C47	C46	C45	C44	C43	C42	C41	C40	C39	C38	C37	C36	C35	C34	C33	C32	C31	C30	C29	C28	C27	C26	C25	C24	C23	C22	C21	C20	C19	C18	C17	C16	C15	C14	C13	C12	C11	C10	C9	C8	C7	C6	C5	C4	C3	C2	C1	C0	C-index	SD	*P*-value	mark	
A	8	13	6	8	6	6	5	10	2	5	6	7	4	10	7	10	3	9	8	8	10	6	4	4	3	4	5	4	7	5	5	5	5	3	2	8	8	7	5	13	8	13	4	5	12	5	2	5	6	3	5	1.268	0.572	–	–	A
C	6	0	3	1	4	2	3	0	0	3	3	1	1	3	2	2	1	1	3	3	0	0	4	3	2	2	4	1	1	1	3	3	3	0	8	0	6	6	6	2	2	3	4	3	4	3	7	2	3	2	8	0.325	0.238	5.55E-08	*	C
D	3	3	4	5	6	3	8	4	5	2	8	7	4	4	7	4	3	5	2	5	6	7	7	7	9	4	9	2	3	8	6	7	4	7	5	5	3	5	3	5	4	5	6	9	6	6	5	5	5	4	3	1.727	0.623	–	–	D
E	3	4	4	8	5	10	7	7	5	7	6	7	7	6	2	4	4	6	8	6	3	6	4	2	2	2	1	10	2	4	5	4	6	6	3	5	4	7	6	1	7	10	4	9	7	2	4	7	4	2	14	0.364	0.168	5.55E-08	*	E
F	5	4	5	5	[illegible]	[illegible]	[illegible]	[illegible]	[illegible]	[illegible]	[illegible]	[illegible]	7	3	3	6	3	5	7	6	8	4	2	3	2	7	11	5	2	4	2	4	3	3	1	6	7	7	9	4	4	10	12	5	1	7	5	7	7	6	3	1.633	0.839	–	–	F
G	6	8	9	8	[illegible]	[illegible]	[illegible]	[illegible]	[illegible]	[illegible]	[illegible]	[illegible]	7	5	6	4	10	5	7	9	7	8	6	7	8	4	6	4	5	9	8	7	3	8	8	5	5	9	3	6	5	5	8	10	4	5	3	4	5	4	6	1.110	0.423	–	–	G
H	7	3	3	3	[illegible]	[illegible]	[illegible]	[illegible]	[illegible]	[illegible]	[illegible]	[illegible]	3	2	2	4	3	3	2	7	4	4	3	6	2	3	6	3	4	2	10	6	3	6	3	0	2	4	3	2	6	7	1	4	3	3	3	5	8	2	5	0.740	0.407	7.74E-02	n.s.	H
I	7	8	6	5	[illegible]	[illegible]	[illegible]	[illegible]	[illegible]	[illegible]	[illegible]	[illegible]	5	7	6	6	5	4	5	5	5	[illegible]	[illegible]	[illegible]	[illegible]	[illegible]	[illegible]	[illegible]	[illegible]	[illegible]	[illegible]	[illegible]	[illegible]	[illegible]	[illegible]	[illegible]	[illegible]	[illegible]	[illegible]	[illegible]	[illegible]	[illegible]	[illegible]	[illegible]	[illegible]	4	5	7	6	8	9	0.624	0.222	2.70E-04	n.s.	I
K	5	6	10	7	[illegible]	[illegible]	[illegible]	[illegible]	[illegible]	[illegible]	[illegible]	[illegible]	5	9	6	4	5	4	8	6	3	[illegible]	[illegible]	[illegible]	[illegible]	[illegible]	[illegible]	[illegible]	[illegible]	[illegible]	[illegible]	[illegible]	[illegible]	[illegible]	[illegible]	[illegible]	[illegible]	[illegible]	[illegible]	[illegible]	[illegible]	[illegible]	[illegible]	[illegible]	[illegible]	4	5	8	8	4	9	0.571	0.241	2.63E-06	*	K
L	3	7	3	12	[illegible]	[illegible]	[illegible]	[illegible]	[illegible]	[illegible]	[illegible]	[illegible]	13	7	10	10	10	7	6	12	6	13	15	12	9	11	3	9	8	13	10	18	9	17	11	15	10	18	16	11	5	11	8	7	7	13	8	7	9	6	5	2.040	0.747	–	–	L
M	0	0	4	1	2	1	0	0	4	1	3	1	0	2	2	3	1	2	0	1	1	1	4	5	2	1	3	3	1	1	0	0	2	5	1	2	1	1	2	1	4	1	0	4	1	3	2	1	2	3	4	0.430	0.346	8.70E-07	*	M
N	6	5	2	4	6	2	6	4	6	3	7	5	6	7	2	5	3	7	1	9	4	6	5	4	9	6	8	5	3	6	2	5	8	7	11	6	3	0	2	7	1	6	7	5	0	2	3	4	2	5	5	0.952	0.484	–	–	N
P	5	8	5	6	6	14	6	5	5	9	12	3	3	8	10	8	4	3	12	7	5	11	9	3	3	4	5	7	4	7	9	7	4	6	12	5	6	7	6	8	9	3	5	3	5	3	5	10	9	5	5	1.296	0.559	–	–	P
Q	3	6	4	2	4	7	5	2	2	2	1	2	3	5	4	5	6	2	1	2	7	3	5	1	2	4	4	1	4	0	1	2	1	2	4	3	3	4	6	4	1	5	7	5	6	1	6	4	7	4	7	0.500	0.276	9.64E-07	*	Q
R	5	8	4	3	8	2	7	2	7	17	3	7	4	5	24	0	2	3	7	4	4	5	3	6	9	2	2	6	5	1	3	0	3	4	10	7	5	7	2	4	6	6	4	5	3	1	13	5	6	4	4	1.315	1.026	–	–	R
S	10	12	8	22	9	9	10	9	6	10	12	17	10	14	9	9	7	7	8	12	10	4	7	12	10	4	9	9	9	22	18	9	11	12	8	16	7	1	10	19	16	12	15	13	13	9	17	9	8	19	13	0.843	0.339	–	–	S
T	8	5	10	2	7	10	5	4	4	5	6	4	3	6	6	6	6	8	7	7	5	5	4	12	5	2	4	4	10	8	1	5	5	8	10	7	7	6	3	7	6	9	12	5	4	5	3	8	7	7	11	0.551	0.223	1.46E-07	*	T
V	6	6	6	5	5	9	7	7	0	9	7	6	8	4	4	4	4	11	8	7	2	3	8	3	4	9	6	4	6	8	7	6	4	4	5	5	8	7	6	12	3	5	9	14	7	10	3	5	9	3	9	0.684	0.296	8.28E-02	n.s.	V
W	0	2	2	2	1	0	0	0	1	1	2	2	0	1	3	1	1	2	3	1	1	1	3	1	1	0	0	0	0	1	2	3	2	1	1	2	0	0	1	1	1	0	2	2	3	3	1	0	1	0	4	0.295	0.246	5.55E-08	*	W
Y	3	5	4	2	0	5	2	3	3	3	1	1	4	4	4	3	8	4	4	5	4	6	4	1	5	1	1	5	2	7	7	3	2	3	3	5	2	3	4	4	4	4	10	1	6	5	6	5	5	2	5	0.752	0.395	1.00E+00	n.s.	Y
total	99	113	102	111	97	124	110	92	92	108	121	113	97	112	119	98	89	98	107	122	95	106	107	102	101	83	97	97	88	111	105	105	85	110	114	113	96	109	106	118	103	125	127	120	99	94	106	108	117	93	134	0.786	0.080			total

Additionary specified positions in three-position-specified PB motifs

the number of genes that encode proteins possessing x-E-S-x-I motif at C0 position

position -1

	C50	C49	C48	C47	C46	C45	C44	C43	C42	C41	C40	C39	C38	C37	C36	C35	C34	C33	C32	C31	C30	C29	C28	C27	C26	C25	C24	C23	C22	C21	C20	C19	C18	C17	C16	C15	C14	C13	C12	C11	C10	C9	C8	C7	C6	C5	C4	C3	C2	C1	C0	C-index	SD	*P*-value	mark	
A	7	3	7	4	8	4	8	3	3	4	4	9	3	7	7	6	7	7	5	5	7	6	6	9	6	3	4	10	10	1	9	6	6	6	10	7	1	7	2	9	9	11	7	3	5	4	2	4	5	3	9	0.642	0.280	2.06E-02	n.s.	A
C	6	6	2	1	4	5	3	4	2	5	3	2	2	9	4	2	2	4	0	4	3	0	2	1	0	3	2	1	5	3	4	4	1	2	6	3	6	2	3	0	2	1	1	5	4	4	1	4	4	0	3	0.980	0.644	–	–	C
D	5	7	5	7	4	5	9	5	6	3	3	6	2	1	2	4	5	8	5	5	6	8	7	4	3	1	4	0	7	5	5	3	2	6	13	5	4	8	6	4	3	7	4	12	1	2	6	4	7	4	4	1.240	0.643	–	–	D
E	7	5	9	5	6	8	6	10	2	18	6	6	5	10	5	7	4	6	9	2	5	6	3	4	8	2	11	2	4	4	4	8	5	9	6	11	3	7	6	7	7	3	7	4	6	8	10	9	5	1	11	0.565	0.274	2.49E-06	*	E
F	5	7	5	4	6	6	5	5	3	4	2	8	5	5	5	4	3	3	5	13	7	5	6	3	6	4	2	1	2	8	6	3	4	6	8	8	4	6	4	9	6	6	10	4	4	8	6	5	10	4	4	1.340	0.575	–	–	F
G	15	9	5	8	2	5	12	5	1	8	6	12	12	3	6	11	5	7	6	6	5	14	3	5	9	7	6	5	7	7	2	6	8	4	3	6	5	5	6	6	3	11	10	3	4	4	8	2	6	11	11	0.591	0.293	5.91E-05	*	G
H	1	4	3	1	1	2	2	1	1	5	1	2	1	4	5	1	6	4	2	4	0	5	4	4	0	0	1	6	3	4	3	3	2	2	1	3	2	1	6	4	4	5	3	2	1	2	3	2	6	4	3	0.913	0.571	–	–	H
I	5	7	7	8	6	4	7	4	3	8	3	7	5	6	7	3	7	6	2	8	9	2	4	4	7	2	5	6	5	3	5	2	4	5	3	5	6	3	4	4	3	8	7	4	5	6	4	2	5	5	6	0.833	0.312	–	–	I
K	4	8	[illegible]	[illegible]	[illegible]	[illegible]	[illegible]	[illegible]	[illegible]	[illegible]	10	6	2	4	9	9	5	9	10	10	3	5	2	3	6	0	5	6	3	0	2	5	8	6	6	8	8	5	2	4	5	7	8	8	9	3	4	5	12	4	9	0.616	0.317	2.06E-02	n.s.	K
L	7	[illegible]	[illegible]	[illegible]	[illegible]	[illegible]	[illegible]	[illegible]	[illegible]	[illegible]	20	16	11	11	8	8	5	8	14	9	4	[illegible]	[illegible]	[illegible]	[illegible]	[illegible]	[illegible]	[illegible]	[illegible]	[illegible]	[illegible]	[illegible]	[illegible]	[illegible]	[illegible]	[illegible]	[illegible]	[illegible]	[illegible]	[illegible]	[illegible]	[illegible]	[illegible]	[illegible]	[illegible]	10	10	10	10	7	13	0.791	0.313	–	–	L
M	2	[illegible]	[illegible]	[illegible]	[illegible]	[illegible]	[illegible]	[illegible]	[illegible]	[illegible]	3	2	9	1	2	0	2	0	3	3	0	[illegible]	[illegible]	[illegible]	[illegible]	[illegible]	[illegible]	[illegible]	[illegible]	[illegible]	[illegible]	[illegible]	[illegible]	[illegible]	[illegible]	[illegible]	[illegible]	[illegible]	[illegible]	[illegible]	[illegible]	[illegible]	[illegible]	[illegible]	[illegible]	3	1	3	2	3	1	2.000	1.702	–	–	M
N	3	[illegible]	[illegible]	[illegible]	[illegible]	[illegible]	[illegible]	[illegible]	[illegible]	[illegible]	5	3	7	1	6	6	3	4	6	2	5	6	1	2	4	3	1	4	6	0	4	2	3	2	4	3	5	6	4	10	6	1	2	4	5	5	9	3	7	12	3	1.367	0.823	–	–	N
P	5	[illegible]	[illegible]	[illegible]	[illegible]	[illegible]	[illegible]	[illegible]	[illegible]	[illegible]	4	10	3	8	2	7	8	5	5	8	6	5	9	13	13	11	3	4	1	7	14	5	4	3	6	7	5	2	2	3	3	6	7	9	12	4	6	7	4	4	7	0.846	0.442	–	–	P
Q	6	[illegible]	[illegible]	[illegible]	[illegible]	[illegible]	[illegible]	[illegible]	[illegible]	[illegible]	8	3	4	3	6	2	2	3	5	7	1	3	6	4	5	2	8	8	5	8	2	7	1	6	1	8	5	6	4	4	8	7	5	5	2	5	6	7	6	6	6	0.837	0.399	–	–	Q
R	3	10	[illegible]	[illegible]	[illegible]	[illegible]	[illegible]	[illegible]	[illegible]	[illegible]	8	4	4	3	8	4	4	2	6	9	5	5	7	0	4	5	6	7	1	7	4	8	5	4	5	2	5	8	6	9	11	8	9	12	6	3	11	13	3	4	9	0.651	0.317	2.78E-02	n.s.	R
S	9	9	7	8	8	10	12	6	10	9	16	6	8	12	6	4	8	5	8	14	7	4	7	16	8	6	6	7	7	11	15	8	13	15	13	9	12	15	13	9	5	12	11	12	14	7	11	12	11	6	12	0.795	0.274	–	–	S
T	0	4	4	19	3	11	7	3	4	4	8	8	5	1	20	6	4	3	6	3	8	17	7	5	3	6	4	3	3	5	4	3	2	12	6	11	6	5	4	2	6	7	6	4	2	5	3	5	6	5	9	0.640	0.454	1.31E-05	*	T
V	6	9	11	5	6	12	9	7	8	3	8	1	5	12	5	6	5	12	4	5	10	4	11	5	4	8	7	4	5	8	2	12	4	5	10	3	6	6	12	11	11	6	9	10	5	7	1	4	3	3	9	0.744	0.349	6.87E-01	n.s.	V
W	1	1	1	3	0	4	1	3	3	3	0	0	0	4	1	2	2	0	1	2	3	2	3	2	3	3	2	2	3	1	3	3	0	0	2	1	1	2	4	2	2	2	5	5	3	2	1	4	2	5	2	1.050	0.687	–	–	W
Y	2	4	4	2	1	4	0	3	4	2	3	2	4	7	5	6	2	2	5	3	1	3	5	4	1	7	3	3	5	2	2	5	0	5	1	5	2	0	1	5	0	2	4	3	1	2	3	3	3	2	3	0.987	0.587	–	–	Y
total	99	113	102	111	97	124	110	92	92	108	121	113	97	112	119	98	89	98	107	122	95	106	107	102	101	83	97	97	88	111	105	105	85	110	114	113	96	109	106	118	103	125	127	120	99	94	106	108	117	93	134	0.786	0.080			total

Values shown in 'total' are the same as those in two-position-specified PB motifs in Additional file 3.

the number of genes that encode proteins possessing x-x-S-D-I motif at C2 position

The three panels are examples to explain how to read the information in the following actual results. Three panels vertically arranged in each motif represent three positions (upper, -4; middle. -3; lower, -1), one of which is additionally specified in three-position-specified PB motifs. Twenty amino acids that are substituted at each position are vertically arranged at the most left and right columns in each panel. C-index and standard deviation (SD) of each three-position-specified motifs are shown at the right of each panel. *P*-value and asterisk are also shown when C-index is significantly (Steel test, *P <1.00E-04) lower than that of ‘total’ (i.e. two-position-specified motif). 'n.s.', not significant.

Additional file 5. Continued.

HUMAN x-x-S-x-I

position -4

	C50	C49	C48	C47	C46	C45	C44	C43	C42	C41	C40	C39	C38	C37	C36	C35	C34	C33	C32	C31	C30	C29	C28	C27	C26	C25	C24	C23	C22	C21	C20	C19	C18	C17	C16	C15	C14	C13	C12	C11	C10	C9	C8	C7	C6	C5	C4	C3	C2	C1	C0	C-index	SD	*P-value*	mark	
A	5	7	9	1	8	4	6	2	1	4	8	7	6	9	3	4	3	7	15	10	6	6	9	3	2	5	3	6	8	9	6	3	5	7	7	10	4	7	7	8	5	5	8	2	2	3	1	2	5	5	9	0.618	0.317	8.27E-03	n.s.	A
C	3	1	2	0	2	3	3	1	1	2	1	7	4	9	1	1	4	3	1	5	1	1	1	1	0	1	0	3	3	2	2	2	2	3	0	3	0	3	4	3	0	4	5	1	3	1	5	4	4	5	3	0.807	0.628	−	−	C
D	5	5	1	9	2	8	3	5	3	3	1	6	2	6	4	6	3	4	0	6	6	4	4	6	7	6	5	7	3	7	2	4	4	8	0	5	7	6	4	4	2	3	2	5	4	4	3	6	5	2	2	2.170	1.038	−	−	D
E	2	10	11	13	4	9	10	11	5	8	7	12	5	5	4	4	3	11	3	6	3	8	3	8	7	5	5	3	5	4	5	3	9	8	2	8	7	5	5	7	11	9	3	2	9	7	7	10	7	7	14	0.464	0.209	5.55E-08	✻	E
F	3	6	2	3	1	7	6	3	5	6	12	3	9	3	5	8	9	1	9	7	3	11	3	7	7	6	2	9	1	8	2	5	3	6	4	0	1	4	6	5	10	9	10	6	3	4	1	6	6	4	3	1.733	0.985	−	−	F
G	2	3	8	7	1	5	8	2	5	8	5	2	3	5	10	3	4	4	7	7	6	9	6	7	4	6	5	4	2	1	3	6	4	2	4	10	3	7	4	3	4	4	8	6	7	5	6	8	8	6	9	0.571	0.256	5.63E-05	✻	G
H	2	3	3	3	2	2	3	1	3	3	3	5	3	5	2	1	2	2	3	4	0	2	4	2	2	6	3	1	3	1	10	2	2	1	6	3	4	1	3	3	1	10	1	2	1	3	2	2	2	5	3	0.953	0.656	−	−	H
I	3	4	3	4	2	6	6	8	7	6	5	2	6	7	5	6	4	4	7	8	6	9	5	3	2	7	4	2	4	6	5	6	4	7	6	6	9	7	4	7	2	8	9	8	8	6	7	5	4	5	4	1.370	0.493	−	−	I
K	10	6	3	6	1	9	5	0	6	6	7	3	2	6	26	2	6	7	2	11	4	10	5	9	2	3	4	6	4	13	3	12	6	7	8	5	5	12	9	12	7	3	6	5	8	3	13	6	6	5	12	0.542	0.354	2.12E-06	✻	K
L	13	14	8	8	13	13	17	6	6	7	9	13	7	7	12	11	13	11	11	11	8	11	17	14	15	7	17	17	12	10	26	8	10	19	16	16	15	9	14	13	5	13	22	14	11	15	14	9	8	7	16	0.753	0.268	9.81E-01	n.s.	L
M	4	2	0	6	3	6	2	1	1	3	5	2	4	2	2	2	2	1	2	1	1	2	5	1	7	1	1	2	1	0	4	3	1	1	3	3	4	2	1	2	4	4	3	4	0	2	1	3	4	0	0	−	−	−	−	M
N	4	3	2	4	13	6	2	4	5	6	12	3	5	6	3	7	7	8	6	4	4	3	2	3	4	0	4	1	1	2	1	4	4	4	5	4	4	3	2	8	5	4	2	3	7	2	2	2	3	5	1	4.160	2.518	−	−	N
P	7	3	9	7	4	3	7	5	5	8	7	6	8	7	5	7	8	5	1	5	9	8	4	10	4	6	4	9	9	10	2	6	4	2	6	9	11	5	6	11	7	5	4	6	4	8	4	8	5	8	7	0.889	0.338	−	−	P
Q	8	2	4	6	4	5	4	5	1	3	6	3	4	6	4	4	3	3	4	4	8	4	7	1	2	3	6	2	5	12	1	6	2	2	10	5	1	10	6	4	3	6	9	11	4	5	3	6	5	2	6	0.780	0.434	8.88E-01	n.s.	Q
R	6	9	6	2	5	3	5	5	0	5	2	7	4	3	3	5	3	5	8	5	9	7	8	5	7	5	6	5	5	7	7	7	6	6	9	5	3	8	5	6	6	6	8	10	7	2	5	7	8	6	12	0.470	0.173	5.55E-08	✻	R
S	7	15	10	9	14	13	12	11	12	16	11	12	8	13	12	7	5	10	9	9	9	5	10	10	12	3	11	4	11	6	8	11	5	10	19	7	4	7	10	6	17	6	8	11	12	10	12	12	14	11	16	0.620	0.214	1.67E-05	✻	S
T	9	10	8	9	10	8	4	12	10	6	8	9	7	6	8	5	3	4	7	8	3	3	4	3	4	5	5	4	6	7	11	10	8	6	4	4	7	6	9	5	6	10	6	11	3	3	5	6	8	5	5	1.312	0.500	−	−	T
V	3	9	9	11	3	13	4	8	8	4	8	8	4	4	3	8	5	5	8	11	7	1	3	6	8	5	9	8	3	5	5	6	4	7	4	8	4	5	5	7	5	13	7	6	2	8	8	6	5	0	5	1.224	0.558	−	−	V
W	2	1	0	2	0	0	0	1	2	3	1	0	1	1	1	4	0	3	3	0	0	1	1	0	4	2	2	1	0	0	0	0	1	3	0	0	1	1	0	0	2	2	1	1	2	1	1	0	7	3	1	1.240	1.408	−	−	W
Y	1	0	4	1	5	1	3	1	6	1	3	3	5	2	6	3	2	0	1	0	2	1	6	3	1	1	1	3	2	1	2	1	1	1	1	2	2	1	2	4	1	1	5	6	2	2	6	0	3	2	6	0.380	0.294	7.18E-08	✻	Y
total	99	113	102	111	97	124	110	92	92	108	121	113	97	112	119	98	89	98	107	122	95	106	107	102	101	83	97	97	88	111	105	105	85	110	114	113	96	109	106	118	103	125	127	120	99	94	106	108	117	93	134	0.786	0.080			total

position -3

	C50	C49	C48	C47	C46	C45	C44	C43	C42	C41	C40	C39	C38	C37	C36	C35	C34	C33	C32	C31	C30	C29	C28	C27	C26	C25	C24	C23	C22	C21	C20	C19	C18	C17	C16	C15	C14	C13	C12	C11	C10	C9	C8	C7	C6	C5	C4	C3	C2	C1	C0	C-index	SD	*P-value*	mark	
A	8	13	6	8	6	6	5	10	2	5	6	7	4	10	7	10	3	9	8	8	10	6	4	4	3	4	5	4	7	5	5	5	5	3	2	8	8	7	5	13	8	13	4	5	12	5	2	5	6	3	5	1.268	0.572	−	−	A
C	6	0	3	1	4	2	3	0	0	3	3	1	1	3	2	2	1	1	3	3	0	0	4	3	2	2	4	1	1	1	3	3	3	0	8	0	6	6	6	2	2	3	4	3	4	3	7	2	3	2	8	0.325	0.238	5.55E-08	✻	C
D	3	3	4	5	6	3	8	4	5	2	8	7	4	4	7	4	3	5	2	5	6	7	7	7	9	4	9	2	3	8	6	7	4	7	5	5	3	5	3	5	4	5	6	9	6	6	5	5	5	4	3	1.727	0.623	−	−	D
E	3	4	4	8	5	10	7	7	5	7	6	7	7	6	2	4	4	6	8	6	3	6	4	2	2	2	1	10	2	4	5	4	6	6	3	5	4	7	6	1	7	10	4	9	7	2	4	7	4	2	14	0.364	0.168	5.55E-08	✻	E
F	5	4	5	5	4	6	6	3	4	1	2	2	7	3	3	6	3	5	7	6	8	4	2	3	2	7	11	5	2	4	2	4	3	3	1	6	7	7	9	4	4	10	12	5	1	7	5	7	7	6	3	1.633	0.839	−	−	F
G	6	8	9	8	5	8	2	8	11	9	14	13	7	5	6	4	10	5	7	9	7	8	6	7	8	4	6	4	5	9	8	7	3	8	8	5	5	9	3	6	5	5	8	10	4	5	3	4	5	4	6	1.110	0.423	−	−	G
H	7	3	3	3	4	5	1	2	2	2	2	7	3	2	2	4	3	3	2	7	4	4	3	6	2	3	6	3	4	2	10	6	3	6	3	0	2	4	3	2	6	7	1	4	3	3	3	5	8	2	5	0.740	0.407	7.74E-02	n.s.	H
I	7	8	6	5	4	10	7	3	4	2	8	5	5	7	6	6	5	4	5	5	5	3	9	6	9	8	5	10	7	4	5	7	3	4	2	7	5	7	5	4	4	4	3	9	4	4	5	7	6	8	9	0.624	0.222	2.70E-04	n.s.	I
K	5	6	10	7	3	4	6	8	7	7	5	5	5	9	6	4	5	4	8	6	3	10	1	4	5	5	5	5	5	0	1	4	4	4	6	4	4	3	8	3	7	6	6	2	3	4	5	8	8	4	9	0.571	0.241	2.63E-06	✻	K
L	3	7	3	12	8	11	17	11	14	10	13	11	13	7	10	10	10	7	6	12	6	13	15	12	9	11	3	9	8	13	10	18	9	17	11	15	10	18	16	11	5	11	8	7	7	13	8	7	9	6	5	2.040	0.747	−	−	L
M	0	0	4	1	2	1	0	0	4	1	3	1	0	2	2	3	1	2	0	1	1	1	4	5	2	1	3	3	1	1	0	0	2	5	1	2	1	1	2	1	4	1	0	4	1	3	2	1	2	3	4	0.430	0.346	8.70E-07	✻	M
N	6	5	2	4	6	2	6	4	6	3	7	5	6	7	2	5	3	7	1	9	4	6	5	4	9	6	8	5	3	6	2	5	8	7	11	6	3	0	2	7	1	6	7	5	0	2	3	4	2	5	5	0.952	0.484	−	−	N
P	5	8	5	6	6	14	6	5	5	9	12	3	3	8	10	8	4	3	12	7	5	11	9	3	3	4	5	7	4	7	9	7	4	6	12	5	6	7	6	8	9	3	5	3	5	3	5	10	9	5	5	1.296	0.559	−	−	P
Q	3	6	4	2	4	7	5	2	2	2	1	2	3	5	4	5	6	2	1	2	7	3	5	1	2	4	4	1	4	0	1	2	1	2	4	3	3	4	6	4	1	5	7	5	6	1	6	4	7	4	7	0.500	0.276	9.64E-07	✻	Q
R	5	8	4	3	8	2	7	2	7	17	3	7	4	5	24	0	2	3	7	4	4	5	3	6	9	2	2	6	5	1	3	0	3	4	10	7	5	7	2	4	6	6	4	5	3	1	13	5	6	4	4	1.315	1.026	−	−	R
S	10	12	8	22	9	9	10	9	6	10	12	17	10	14	9	9	7	7	8	12	10	4	7	12	10	4	9	9	9	22	18	9	11	12	8	16	7	1	10	19	16	12	15	13	13	9	17	9	8	19	13	0.843	0.339	−	−	S
T	8	5	10	2	7	10	5	4	4	5	6	4	3	6	6	6	6	8	7	7	5	5	4	12	5	2	4	4	10	8	1	5	5	8	10	7	7	6	3	7	6	9	12	5	4	5	3	8	7	7	11	0.551	0.223	1.46E-07	✻	T
V	6	6	6	5	5	9	7	7	0	9	7	6	8	4	4	4	4	11	8	7	2	3	8	3	4	9	6	4	6	8	7	6	4	4	5	5	8	7	6	12	3	5	9	14	7	10	3	5	9	3	9	0.684	0.296	8.28E-02	n.s.	V
W	0	2	2	2	1	0	0	0	1	1	2	2	0	1	3	1	1	2	3	1	1	1	3	1	1	0	0	0	0	1	2	3	2	1	1	2	0	0	1	1	1	0	2	2	3	3	1	0	1	0	4	0.295	0.246	5.55E-08	✻	W
Y	3	5	4	2	0	5	2	3	3	3	1	1	4	4	4	3	8	4	4	5	4	6	4	1	5	1	1	5	2	7	7	3	2	3	3	5	2	3	4	4	4	4	10	1	6	5	6	5	5	2	5	0.752	0.395	1.00E+00	n.s.	Y
total	99	113	102	111	97	124	110	92	92	108	121	113	97	112	119	98	89	98	107	122	95	106	107	102	101	83	97	97	88	111	105	105	85	110	114	113	96	109	106	118	103	125	127	120	99	94	106	108	117	93	134	0.786	0.080			total

position -1

	C50	C49	C48	C47	C46	C45	C44	C43	C42	C41	C40	C39	C38	C37	C36	C35	C34	C33	C32	C31	C30	C29	C28	C27	C26	C25	C24	C23	C22	C21	C20	C19	C18	C17	C16	C15	C14	C13	C12	C11	C10	C9	C8	C7	C6	C5	C4	C3	C2	C1	C0	C-index	SD	*P-value*	mark	
A	7	3	7	4	8	4	8	3	3	4	4	9	3	7	7	6	7	7	5	5	7	6	6	9	6	3	4	10	10	1	9	6	6	6	10	7	1	7	2	9	9	11	7	3	5	4	2	4	5	3	9	0.642	0.280	2.06E-02	n.s.	A
C	6	6	2	1	4	5	3	4	2	5	3	2	2	9	4	2	2	4	0	4	3	0	2	1	0	3	2	1	5	3	4	4	1	2	6	3	6	2	3	0	2	1	1	5	4	4	1	4	4	0	3	0.980	0.644	−	−	C
D	5	7	5	7	4	5	9	5	6	3	3	6	2	1	2	4	5	8	5	5	6	8	7	4	3	1	4	0	7	5	5	3	2	6	13	5	4	8	6	4	3	7	4	12	1	2	6	4	7	4	4	1.240	0.643	−	−	D
E	7	5	9	5	6	8	6	10	2	18	6	6	5	10	5	7	4	6	9	2	5	6	3	4	8	2	11	2	4	4	4	8	5	9	6	11	3	7	6	7	7	3	7	4	6	8	10	9	5	1	11	0.565	0.274	2.49E-06	✻	E
F	5	7	5	4	6	6	5	5	3	4	2	8	5	5	5	4	3	3	5	13	7	5	6	3	6	4	2	1	2	8	6	3	4	6	8	8	4	6	4	9	6	6	10	4	4	8	6	5	10	4	4	1.340	0.575	−	−	F
G	15	9	5	8	2	5	12	5	1	8	6	12	12	3	6	11	5	7	6	6	5	14	3	5	9	7	6	5	7	7	2	6	8	4	3	6	5	5	6	6	3	11	10	3	4	4	8	2	6	11	11	0.591	0.293	5.91E-05	✻	G
H	1	4	3	1	1	2	2	1	1	5	1	2	1	4	5	1	6	4	2	4	0	5	4	4	0	0	1	6	3	4	3	3	2	2	1	3	2	1	6	4	4	5	3	2	1	2	3	2	6	4	3	0.913	0.571	−	−	H
I	5	7	7	8	6	4	7	4	3	8	3	7	5	6	7	3	7	6	2	8	9	2	4	4	7	2	5	6	5	3	5	2	4	5	3	5	6	3	4	4	3	8	7	4	5	6	4	2	5	5	6	0.833	0.312	−	−	I
K	4	8	1	4	1	6	9	6	7	5	10	6	2	4	9	9	5	9	10	10	3	5	2	3	6	0	5	6	3	0	2	5	8	6	6	8	8	5	2	4	5	7	8	8	9	3	4	5	12	4	9	0.616	0.317	2.06E-02	n.s.	K
L	7	9	9	12	16	8	5	4	13	9	20	16	11	11	8	8	5	8	14	9	4	6	13	12	9	9	16	12	6	24	9	9	11	10	10	5	8	15	16	15	6	15	9	9	7	10	10	10	10	7	13	0.791	0.313	−	−	L
M	2	2	3	3	1	2	1	1	1	0	3	2	9	1	2	0	2	0	3	3	0	0	1	2	2	1	1	6	0	3	6	3	2	2	0	3	2	0	1	1	3	0	3	2	3	3	1	3	2	3	1	2.000	1.702	−	−	M
N	3	3	2	4	5	4	1	8	2	1	5	3	7	1	6	6	3	4	6	2	5	6	1	2	4	3	1	4	6	0	4	2	3	2	4	3	5	6	4	10	6	1	2	4	5	5	9	3	7	12	3	1.367	0.823	−	−	N
P	5	3	6	7	4	9	2	4	7	4	4	10	3	8	2	7	8	5	5	8	6	5	9	13	13	11	3	4	1	7	14	5	4	3	6	7	5	2	2	3	3	6	7	9	12	4	6	7	4	4	7	0.846	0.442	−	−	P
Q	6	3	2	2	10	9	4	5	10	6	8	3	4	3	6	2	2	3	5	7	1	3	6	4	5	2	8	8	5	8	2	7	1	6	1	8	5	6	4	4	8	7	5	5	2	5	6	7	6	6	6	0.837	0.399	−	−	Q
R	3	10	9	4	5	6	7	5	2	7	8	4	4	3	8	4	4	2	6	9	5	5	7	0	4	5	6	7	1	7	4	8	5	4	5	2	5	8	6	9	11	8	9	12	6	3	11	13	3	4	9	0.651	0.317	2.78E-02	n.s.	R
S	9	9	7	8	8	10	12	6	10	9	16	6	8	12	6	4	8	5	8	14	7	4	7	16	8	6	6	7	7	11	15	8	13	15	13	9	12	15	13	9	5	12	11	12	14	7	11	12	11	6	12	0.795	0.274	−	−	S
T	0	4	4	19	3	11	7	3	4	4	8	8	5	1	20	6	4	3	6	3	8	17	7	5	3	6	4	3	3	5	4	3	2	12	6	11	6	5	4	2	6	7	6	4	2	5	3	5	6	5	9	0.640	0.454	1.31E-05	✻	T
V	6	9	11	5	6	12	9	7	8	3	8	1	5	12	5	6	5	12	4	5	10	4	11	5	4	8	7	4	5	8	2	12	4	5	10	3	6	6	12	11	11	6	9	10	5	7	1	4	3	3	9	0.744	0.349	6.87E-01	n.s.	V
W	1	1	1	3	0	4	1	3	3	3	0	0	0	4	1	2	2	0	1	2	3	2	3	2	3	3	2	2	3	1	3	3	0	0	2	1	1	2	4	2	2	2	5	5	3	2	1	4	2	5	2	1.050	0.687	−	−	W
Y	2	4	4	2	1	4	0	3	4	2	3	2	4	7	5	6	2	2	5	3	1	3	5	4	1	7	3	3	5	2	2	5	0	5	1	5	2	0	1	5	0	2	4	3	1	2	3	3	3	2	3	0.987	0.587	−	−	Y
total	99	113	102	111	97	124	110	92	92	108	121	113	97	112	119	98	89	98	107	122	95	106	107	102	101	83	97	97	88	111	105	105	85	110	114	113	96	109	106	118	103	125	127	120	99	94	106	108	117	93	134	0.786	0.080			total

Additional file 5. Continued.

HUMAN x-x-S-x-L

position -4

	C50	C49	C48	C47	C46	C45	C44	C43	C42	C41	C40	C39	C38	C37	C36	C35	C34	C33	C32	C31	C30	C29	C28	C27	C26	C25	C24	C23	C22	C21	C20	C19	C18	C17	C16	C15	C14	C13	C12	C11	C10	C9	C8	C7	C6	C5	C4	C3	C2	C1	C0	C-index	SD	*P-value*	mark	
A	17	20	20	20	20	22	16	18	21	20	16	16	16	22	13	16	26	21	24	24	18	17	16	20	20	12	28	24	16	15	23	19	37	14	11	22	16	17	13	15	24	17	17	19	20	18	12	25	14	18	31	0.610	0.150	3.15E-05	✻	A
C	5	6	7	9	5	9	7	6	6	11	12	3	8	9	4	9	5	8	9	4	6	7	7	7	6	7	3	7	8	13	5	6	12	6	11	8	8	9	10	7	9	8	8	8	10	8	10	12	10	6	14	0.549	0.167	5.47E-07	✻	C
D	14	12	13	11	14	13	8	10	23	10	14	8	19	13	8	7	18	10	11	21	8	5	6	12	7	12	8	10	15	10	18	10	9	20	11	15	14	13	5	7	10	8	9	12	8	16	8	9	21	8	14	0.830	0.310	−	−	D
E	13	8	22	19	15	35	22	16	10	15	25	14	9	18	18	13	22	13	16	17	10	15	13	11	17	15	15	13	10	11	22	10	12	18	16	12	14	14	16	14	12	6	5	9	15	10	7	19	18	16	13	1.131	0.403	−	−	E
F	11	6	7	8	11	18	8	13	11	9	8	8	9	13	14	11	9	6	9	14	12	10	6	12	17	12	12	7	12	10	12	9	14	10	10	14	15	16	14	12	12	21	17	19	12	13	5	14	9	11	19	0.602	0.184	4.89E-04	n.s.	F
G	14	19	25	16	17	18	16	13	13	22	23	17	9	21	19	14	16	18	20	18	21	35	16	12	16	25	22	21	14	16	25	15	12	15	18	21	20	16	18	16	12	8	19	17	13	22	20	11	27	30	24	0.751	0.215	−	−	G
H	9	4	9	5	12	7	6	3	4	10	15	14	6	10	6	9	6	8	11	5	6	4	10	10	6	6	11	4	9	1	6	2	5	5	2	12	8	5	9	7	9	6	12	5	5	7	7	5	9	4	16	0.445	0.194	5.56E-08	✻	H
I	8	18	13	12	7	18	14	7	13	16	17	6	11	21	13	11	13	5	10	15	14	12	6	17	12	9	22	17	6	11	13	14	8	16	9	11	11	13	14	14	20	13	13	14	8	12	12	13	5	9	14	0.880	0.289	−	−	I
K	11	9	11	15	8	12	14	15	8	8	12	14	7	8	13	13	11	9	6	11	7	11	15	14	15	11	8	15	14	15	21	5	12	8	10	12	8	17	16	13	14	20	9	12	7	19	12	17	10	19	31	0.388	0.122	5.55E-08	✻	K
L	37	27	44	37	27	34	27	33	35	31	30	30	21	35	32	34	34	41	36	36	43	38	19	22	28	30	32	37	32	23	38	33	43	33	28	34	51	30	39	28	27	31	34	32	29	36	23	21	39	33	36	0.904	0.177	−	−	L
M	3	10	8	8	9	4	11	4	4	10	4	3	5	8	3	7	5	2	5	5	9	3	5	3	4	7	5	3	6	7	1	7	5	5	4	5	11	2	9	7	9	6	7	8	6	5	4	7	7	5	6	0.967	0.405	−	−	M
N	10	6	11	5	7	11	9	11	11	10	10	9	7	12	10	10	3	9	8	7	8	8	9	7	6	7	3	12	13	7	9	12	9	11	9	11	16	4	6	8	8	7	23	10	6	9	15	10	11	9	10	0.918	0.332	−	−	N
P	18	15	16	23	21	23	19	16	17	26	24	10	15	11	21	19	8	11	17	19	17	11	16	16	24	26	35	15	7	22	17	17	40	24	14	31	16	25	23	11	20	18	23	19	16	17	20	17	17	17	25	0.752	0.249	−	−	P
Q	14	14	13	20	12	9	20	19	14	18	21	10	17	31	24	17	12	4	13	15	9	10	13	11	12	11	11	6	13	13	17	17	12	9	9	18	13	19	24	8	19	17	21	16	10	16	11	16	5	18	25	0.577	0.207	3.76E-04	n.s.	Q
R	13	21	13	20	14	16	18	11	18	20	17	11	19	27	22	22	9	16	13	23	16	11	12	10	13	11	12	9	15	16	13	12	21	15	18	17	17	18	25	17	14	23	24	18	12	12	13	16	24	19	25	0.653	0.180	1.89E-01	n.s.	R
S	24	23	17	25	30	19	23	36	16	29	36	20	26	41	28	28	27	25	21	20	21	21	28	24	45	23	25	28	23	25	28	16	28	20	22	22	25	27	28	36	20	21	25	27	22	22	28	23	40	50	25	1.046	0.281	−	−	S
T	20	10	17	12	14	12	9	13	21	12	13	15	15	5	10	16	16	18	8	11	17	24	21	15	16	14	10	18	14	24	15	10	13	15	11	15	13	14	13	31	14	20	25	12	13	18	16	21	23	12	26	0.588	0.188	2.45E-05	✻	T
V	21	14	21	15	14	12	19	19	15	11	17	15	12	15	13	17	24	19	11	15	14	20	17	16	9	15	11	16	21	17	14	21	17	14	21	20	15	14	17	22	17	10	20	7	19	16	20	15	15	14	18	0.892	0.202	−	−	V
W	2	2	5	7	2	1	2	3	8	4	5	1	3	7	3	1	4	7	4	3	2	8	0	5	3	5	5	2	3	2	0	4	2	3	5	3	8	2	7	7	4	7	8	6	3	2	10	1	3	0	8	0.485	0.308	1.88E-04	n.s.	W
Y	11	6	2	2	7	9	5	11	10	5	6	12	3	6	5	10	6	5	7	10	11	6	4	7	2	5	7	7	7	4	4	6	4	8	10	7	5	4	8	6	5	4	11	9	9	2	12	6	7	5	10	0.660	0.273	1.57E-01	n.s.	Y
total	275	250	294	289	266	302	273	277	278	297	325	236	237	333	279	284	274	255	259	293	269	276	239	251	278	263	285	271	258	262	301	245	315	269	249	310	304	279	314	286	279	271	330	279	243	280	265	278	314	303	390	0.715	0.062			total

position -3

	C50	C49	C48	C47	C46	C45	C44	C43	C42	C41	C40	C39	C38	C37	C36	C35	C34	C33	C32	C31	C30	C29	C28	C27	C26	C25	C24	C23	C22	C21	C20	C19	C18	C17	C16	C15	C14	C13	C12	C11	C10	C9	C8	C7	C6	C5	C4	C3	C2	C1	C0	C-index	SD	*P-value*	mark	
A	15	18	17	22	19	40	18	9	18	23	23	17	22	20	18	17	15	17	16	20	24	12	15	18	17	22	11	16	10	11	18	14	17	21	13	15	24	18	18	26	12	15	13	16	15	13	19	20	23	21	16	1.114	0.316	−	−	A
C	5	7	4	10	8	5	5	10	3	4	4	10	10	11	4	10	10	7	11	7	11	8	5	6	5	6	7	12	6	5	10	6	8	6	8	6	9	7	13	5	4	8	11	5	4	6	6	5	10	9	16	0.453	0.160	5.55E-08	✻	C
D	10	11	25	19	11	11	11	13	17	10	12	9	9	11	17	12	10	8	5	14	8	14	10	13	12	15	9	7	7	7	10	13	8	4	7	16	13	14	15	17	14	10	7	8	6	8	18	8	19	30	18	0.658	0.273	1.34E-01	n.s.	D
E	9	6	15	5	9	9	16	10	9	15	9	10	15	20	10	18	10	16	13	11	14	12	4	10	11	19	16	11	12	12	16	15	11	19	11	14	12	9	10	9	20	5	10	17	11	14	9	10	10	11	33	0.363	0.116	5.55E-08	✻	E
F	17	12	10	12	17	17	10	15	10	17	21	20	7	14	14	16	20	10	13	12	11	7	11	9	15	9	17	15	16	14	17	10	17	15	13	11	9	11	15	19	12	12	16	21	13	17	17	12	14	8	23	0.597	0.157	1.04E-03	n.s.	F
G	22	13	19	13	19	19	12	17	20	21	26	18	14	25	25	13	25	21	17	26	24	41	20	16	12	13	18	12	12	16	12	13	13	23	15	16	20	15	10	17	17	18	17	23	12	14	18	16	15	14	31	0.572	0.176	2.02E-06	✻	G
H	13	7	8	9	6	10	4	10	11	8	5	7	3	9	4	9	6	5	6	3	5	7	5	10	6	10	11	8	5	7	10	9	11	10	11	6	9	10	10	10	13	9	11	14	8	10	11	10	11	8	9	0.929	0.294	−	−	H
I	18	12	25	13	10	11	13	9	9	10	12	11	13	12	13	9	10	17	5	22	16	10	15	12	13	8	21	9	12	11	12	8	15	14	11	14	13	9	12	5	12	18	12	12	17	19	17	23	12	17	18	0.726	0.235	−	−	I
K	15	15	16	16	8	12	9	12	15	20	20	9	6	14	21	6	3	7	13	10	12	17	10	10	17	10	14	14	9	12	13	7	15	8	5	21	18	16	21	19	15	12	20	12	14	12	10	20	13	15	16	0.823	0.285	−	−	K
L	24	33	25	36	32	41	17	29	32	24	29	21	34	43	24	38	29	27	26	33	31	28	23	24	36	25	29	47	32	27	41	15	39	26	32	45	23	30	24	38	21	29	42	25	25	21	24	25	30	26	40	0.740	0.180	−	−	L
M	5	5	4	5	5	4	7	2	1	4	4	8	1	5	3	6	1	0	4	6	5	5	3	4	1	4	7	5	3	4	5	3	4	3	4	4	1	4	9	4	3	6	6	5	3	7	2	4	5	3	5	0.824	0.374	−	−	M
N	8	9	11	8	16	11	16	15	15	15	15	10	8	11	10	14	12	11	10	5	8	10	10	14	11	10	6	11	10	11	8	11	9	15	11	15	7	16	15	9	9	15	17	7	10	7	9	6	11	13	12	0.918	0.258	−	−	N
P	24	19	21	18	15	11	19	12	15	28	25	15	20	16	14	25	24	15	33	24	18	20	24	16	22	14	17	18	14	20	17	28	44	24	12	13	15	16	24	23	16	16	38	24	20	23	16	26	26	13	24	0.842	0.274	−	−	P
Q	5	13	16	11	2	8	20	11	7	10	14	10	8	11	12	6	12	10	10	6	5	10	12	8	12	12	8	15	21	15	18	11	8	13	6	8	14	13	12	13	6	12	15	7	12	9	12	12	17	14	14	0.789	0.278	−	−	Q
R	11	10	11	15	15	23	13	16	11	17	12	10	8	18	13	15	19	15	14	19	15	16	11	15	8	15	17	11	16	10	17	15	31	15	22	24	13	12	16	17	27	15	14	15	12	11	16	16	14	18	25	0.607	0.178	2.31E-06	✻	R
S	30	28	33	35	43	26	32	45	36	40	40	23	29	47	33	26	31	31	30	26	17	22	20	23	32	28	32	25	30	34	32	32	24	24	28	34	45	38	41	19	26	37	51	21	27	34	30	20	30	40	34	0.918	0.225	−	−	S
T	22	10	8	13	7	17	19	12	14	11	24	12	10	14	19	21	14	11	13	21	18	16	19	21	13	9	26	13	12	18	11	10	15	13	20	10	18	14	18	15	13	11	9	21	12	22	9	21	15	14	21	0.712	0.220	9.74E-01	n.s.	T
V	10	18	17	15	14	17	25	17	21	17	19	10	15	21	12	14	17	13	9	12	12	16	11	16	16	21	12	13	20	25	22	11	12	11	13	22	24	19	21	14	26	18	10	12	15	20	12	18	24	12	24	0.676	0.191	5.93E-01	n.s.	V
W	2	1	0	3	5	4	0	6	1	0	2	1	0	4	1	4	1	6	4	4	4	2	6	4	8	6	2	5	1	1	1	3	7	2	0	6	5	3	2	5	6	2	3	2	1	3	4	1	3	11	4	0.790	0.592	−	−	W
Y	10	3	9	11	5	6	7	7	13	3	9	5	5	7	12	5	5	8	7	12	11	3	5	2	11	7	5	4	10	2	11	11	7	3	7	10	12	5	8	2	7	3	8	12	6	10	6	5	12	6	7	1.029	0.452	−	−	Y
total	275	250	294	289	266	302	273	277	278	297	325	236	237	333	279	284	274	255	259	293	269	276	239	251	278	263	285	271	258	262	301	245	315	269	249	310	304	279	314	286	279	271	330	279	243	280	265	278	314	303	390	0.715	0.062			total

position -1

	C50	C49	C48	C47	C46	C45	C44	C43	C42	C41	C40	C39	C38	C37	C36	C35	C34	C33	C32	C31	C30	C29	C28	C27	C26	C25	C24	C23	C22	C21	C20	C19	C18	C17	C16	C15	C14	C13	C12	C11	C10	C9	C8	C7	C6	C5	C4	C3	C2	C1	C0	C-index	SD	*P-value*	mark	
A	14	8	16	15	17	16	25	15	13	19	22	19	15	28	18	18	18	19	20	21	15	23	17	17	13	13	21	19	19	20	22	14	26	16	17	20	9	16	21	21	17	18	14	14	21	17	15	10	10	17	25	0.694	0.164	9.94E-01	n.s.	A
C	4	9	11	13	4	8	10	5	10	14	18	9	6	15	9	5	10	9	10	14	9	8	4	9	7	9	6	7	20	21	10	5	13	9	7	8	9	17	16	4	6	3	10	10	3	6	12	8	12	16	22	0.434	0.196	5.58E-08	✻	C
D	11	8	18	12	15	12	11	10	9	8	11	12	7	9	12	5	17	8	10	12	10	9	9	12	11	6	9	11	10	9	14	9	15	6	5	7	12	11	16	10	9	12	11	14	5	11	13	10	16	33	9	1.227	0.486	−	−	D
E	12	9	16	10	10	10	14	14	10	13	15	10	14	17	8	17	11	11	12	13	23	9	12	12	11	8	11	12	13	18	21	15	11	11	14	17	13	12	11	14	21	8	11	7	10	10	6	10	12	9	15	0.824	0.237	−	−	E
F	14	7	13	14	7	14	8	11	17	17	9	10	14	15	7	14	10	8	12	11	12	12	12	8	21	8	18	20	11	13	19	11	12	14	15	8	15	20	17	19	13	14	11	15	20	14	16	12	15	14	18	0.734	0.208	−	−	F
G	18	23	20	20	16	21	12	23	22	13	13	12	14	30	15	18	15	12	14	26	11	17	11	14	16	19	13	20	13	16	18	9	14	18	10	17	15	12	14	11	17	18	19	14	13	17	20	13	10	19	22	0.732	0.196	−	−	G
H	10	12	7	13	13	11	7	8	10	10	7	3	4	15	16	17	10	10	9	8	10	10	13	6	9	10	9	8	14	11	5	8	10	16	9	14	8	12	17	9	14	19	12	6	9	13	8	18	8	15	27	0.393	0.135	5.55E-08	✻	H
I	17	9	22	16	8	12	7	9	13	13	10	14	14	9	17	12	11	13	8	9	11	31	11	8	14	9	14	7	11	11	7	10	15	14	10	8	7	16	19	23	9	12	20	10	3	8	15	21	19	7	12	1.038	0.429	−	−	I
K	18	5	14	14	13	13	15	13	8	11	11	7	8	15	16	11	12	7	9	8	11	7	5	13	12	14	15	15	8	7	15	9	4	8	5	12	10	10	8	11	10	6	16	10	10	12	6	11	23	12	24	0.453	0.158	5.55E-08	✻	K
L	33	25	33	42	21	35	31	48	28	30	40	19	17	40	20	25	26	34	25	40	24	40	24	19	25	41	26	32	20	46	27	37	38	30	28	39	35	21	30	31	31	32	41	38	23	34	24	32	33	38	44	0.705	0.173	1.00E+00	n.s.	L
M	1	3	7	1	5	5	2	4	2	4	3	7	6	3	3	7	10	4	5	3	4	4	3	1	5	6	11	4	6	1	5	5	1	5	1	5	6	2	2	3	8	3	4	4	3	2	3	3	6	9	5	0.840	0.464	−	−	M
N	13	12	10	12	10	11	6	10	9	16	18	11	6	11	9	6	14	14	9	12	9	8	7	10	12	5	11	10	8	6	6	5	12	17	6	13	19	13	12	12	13	9	8	7	5	9	7	7	10	5	15	0.667	0.229	6.65E-01	n.s.	N
P	14	19	10	11	10	18	19	11	15	25	21	17	16	17	18	19	11	13	23	20	22	11	21	14	23	10	20	21	9	13	15	19	20	22	16	19	17	13	16	17	16	26	26	23	14	16	16	29	18	23	21	0.830	0.225	−	−	P
Q	5	11	15	16	18	10	20	9	8	7	17	4	17	15	10	14	11	9	8	5	13	11	9	10	12	8	5	4	12	9	13	7	18	12	5	13	25	18	17	15	8	10	12	10	11	5	7	12	15	9	12	0.940	0.382	−	−	Q
R	16	19	11	18	24	31	15	13	16	14	20	15	14	16	19	12	17	9	16	20	9	9	19	16	16	13	15	14	18	8	18	8	21	15	13	18	21	11	15	23	11	17	13	14	15	20	20	13	20	12	23	0.687	0.192	8.71E-01	n.s.	R
S	28	30	38	32	40	27	28	29	37	36	41	31	31	30	41	35	29	30	21	28	35	34	19	33	37	42	27	30	30	24	42	35	28	18	36	46	26	34	35	29	33	32	46	39	34	38	28	20	37	36	40	0.813	0.160	−	−	S
T	17	15	5	8	13	12	15	14	15	17	17	14	10	15	13	16	17	17	21	10	16	11	14	18	12	15	17	16	15	13	17	13	9	19	13	16	23	22	17	9	18	16	27	12	17	13	11	19	18	5	17	0.873	0.245	−	−	T
V	18	13	17	15	14	17	23	22	25	18	18	14	15	18	19	19	13	15	19	18	18	9	18	26	16	17	20	15	15	8	11	16	32	12	24	17	19	12	19	17	18	10	14	21	11	19	21	9	12	13	22	0.763	0.211	−	−	V
W	3	5	4	2	4	9	3	1	3	6	6	1	5	7	3	2	5	5	2	8	5	5	6	4	0	1	7	2	1	3	5	4	10	3	6	5	5	5	2	3	2	2	4	3	3	8	10	10	13	7	8	0.570	0.345	1.66E-03	n.s.	W
Y	9	8	7	5	4	10	2	8	8	6	8	7	4	8	6	12	7	8	6	7	2	8	5	1	6	9	10	4	5	5	11	6	6	4	9	8	10	2	10	5	5	4	11	8	13	8	7	11	7	4	9	0.764	0.302	−	−	Y
total	275	250	294	289	266	302	273	277	278	297	325	236	237	333	279	284	274	255	259	293	269	276	239	251	278	263	285	271	258	262	301	245	315	269	249	310	304	279	314	286	279	271	330	279	243	280	265	278	314	303	390	0.715	0.062			total

Additional file 5. Continued.

HUMAN x-x-S-x-V

position -4

	C50	C49	C48	C47	C46	C45	C44	C43	C42	C41	C40	C39	C38	C37	C36	C35	C34	C33	C32	C31	C30	C29	C28	C27	C26	C25	C24	C23	C22	C21	C20	C19	C18	C17	C16	C15	C14	C13	C12	C11	C10	C9	C8	C7	C6	C5	C4	C3	C2	C1	C0	C-index	SD	*P-value*	mark	
A	7	14	7	10	14	8	8	15	7	10	9	17	9	9	17	9	3	7	5	9	11	17	9	11	11	13	6	5	7	8	14	10	13	5	9	11	10	10	7	5	4	11	10	8	11	14	7	3	21	7	10	0.964	0.383	–	–	A
C	2	2	4	4	1	6	6	3	4	4	3	4	5	6	5	4	0	5	5	5	2	2	1	4	2	3	0	4	7	3	7	5	1	4	7	4	1	1	3	5	5	1	5	4	2	2	1	4	2	4	5	0.696	0.369	–	–	C
D	7	6	7	7	3	7	3	13	12	10	6	5	10	8	5	3	2	8	2	4	5	2	5	4	11	7	8	7	4	7	9	8	6	3	9	4	7	16	3	2	7	4	4	9	5	9	2	7	3	7	13	0.480	0.237	4.23E-05	*	D
E	3	5	7	5	5	5	10	8	10	14	5	11	8	6	11	6	8	9	8	6	11	8	12	9	7	2	6	5	6	11	11	6	8	10	5	9	6	5	9	3	9	9	10	9	8	4	7	11	7	4	10	0.754	0.264	–	–	E
F	9	5	9	7	5	3	11	7	3	10	4	9	7	10	4	6	7	7	4	5	7	4	13	3	5	3	4	7	3	9	4	4	6	6	6	5	6	7	3	4	6	9	10	7	2	9	8	3	6	3	12	0.507	0.211	1.08E-03	n.s.	F
G	8	13	9	7	8	5	8	12	8	12	10	7	8	12	9	9	14	7	6	11	10	12	9	8	5	9	18	27	13	7	13	7	10	8	8	9	10	15	10	9	4	6	7	9	8	22	10	19	6	18	13	0.783	0.340	–	–	G
H	8	4	3	4	2	0	2	3	6	6	4	0	3	5	2	3	2	5	5	3	4	5	7	6	2	0	5	2	3	1	1	1	4	6	3	4	1	3	6	2	4	4	1	3	5	4	6	2	6	2	2	1.730	0.965	–	–	H
I	6	7	5	7	4	4	4	6	8	4	1	3	4	4	6	5	6	2	9	6	2	9	11	8	8	2	4	9	3	4	8	6	11	7	1	4	9	3	8	9	7	6	4	2	9	3	5	6	3	2	14	0.391	0.186	5.81E-08	*	I
K	6	6	10	5	8	10	1	3	6	8	9	6	8	6	4	9	6	8	6	8	6	5	6	5	10	9	6	2	8	4	5	2	5	8	5	6	10	8	7	3	7	10	5	6	9	10	6	8	14	8	13	0.517	0.191	4.71E-03	n.s.	K
L	16	15	22	9	18	17	9	15	12	15	26	11	14	12	21	16	21	15	13	11	13	14	13	15	31	13	17	27	9	16	17	15	14	16	16	20	16	14	12	11	15	19	17	18	10	12	18	11	16	18	26	0.601	0.172	2.19E-01	n.s.	L
M	0	3	1	5	1	2	4	2	2	2	4	2	1	6	6	1	5	7	4	2	2	3	4	3	2	0	6	2	5	4	4	1	2	2	0	4	6	1	5	2	6	4	2	1	3	4	3	3	5	1	5	0.600	0.364	9.64E-01	n.s.	M
N	5	4	0	4	3	2	4	1	4	6	8	5	6	5	4	7	5	4	6	2	3	6	4	6	8	4	3	2	0	2	7	6	4	1	5	8	5	5	7	4	1	3	10	4	10	5	1	3	7	1	6	0.733	0.397	–	–	N
P	13	10	14	7	13	11	19	14	11	12	6	12	12	11	11	7	9	9	9	12	12	9	18	12	11	16	13	12	10	9	13	10	7	15	7	14	11	6	11	9	14	8	12	9	9	19	13	14	7	18	17	0.671	0.189	–	–	P
Q	3	9	14	3	24	7	4	5	7	6	5	5	11	5	9	6	5	10	4	3	1	6	2	8	8	7	11	7	8	3	7	4	2	7	11	2	10	5	6	5	6	5	2	11	5	4	11	3	6	6	12	0.540	0.321	3.93E-03	n.s.	Q
R	7	11	9	9	11	3	10	9	8	7	12	7	8	6	4	13	7	12	7	5	13	2	7	3	5	8	4	7	9	7	5	9	2	6	5	5	8	13	6	9	9	12	4	8	8	2	6	10	5	10	22	0.338	0.134	5.55E-08	*	R
S	15	16	13	9	14	17	14	5	7	14	10	16	12	15	18	14	7	10	14	12	18	11	13	15	6	20	24	14	13	18	20	20	13	20	12	31	21	14	13	11	40	14	12	14	9	14	17	16	20	17	25	0.602	0.235	1.38E-01	n.s.	S
T	12	10	11	7	10	15	12	7	4	11	7	6	13	5	4	11	7	6	5	8	9	17	7	10	14	7	7	5	4	5	4	9	10	2	13	3	10	5	10	4	10	9	2	9	7	13	10	11	13	10	9	0.933	0.390	–	–	T
V	7	4	9	12	6	6	7	6	7	12	8	14	7	9	13	7	11	11	7	14	7	5	10	9	8	11	8	14	5	7	6	7	4	8	3	10	4	2	10	13	9	9	13	6	11	8	8	7	12	5	11	0.756	0.274	–	–	V
W	1	1	4	1	1	3	1	3	3	1	0	2	2	1	1	0	4	1	2	1	3	1	1	1	1	1	5	2	2	4	5	1	2	1	0	4	4	4	4	1	5	1	3	2	2	1	4	4	3	1	1	2.120	1.438	–	–	W
Y	4	4	4	5	3	2	2	2	4	4	2	2	0	4	5	1	4	4	3	3	3	0	2	5	5	6	1	5	2	0	2	3	0	3	4	2	3	2	4	3	9	4	1	8	2	4	2	3	5	4	2	1.590	0.919	–	–	Y
total	139	149	162	127	154	133	139	139	133	168	139	144	148	145	159	137	133	147	124	130	142	138	154	145	160	141	156	165	121	129	162	134	124	138	129	159	158	139	144	114	177	148	134	147	135	163	145	148	167	146	228	0.633	0.060			total

position -3

	C50	C49	C48	C47	C46	C45	C44	C43	C42	C41	C40	C39	C38	C37	C36	C35	C34	C33	C32	C31	C30	C29	C28	C27	C26	C25	C24	C23	C22	C21	C20	C19	C18	C17	C16	C15	C14	C13	C12	C11	C10	C9	C8	C7	C6	C5	C4	C3	C2	C1	C0	C-index	SD	*P-value*	mark	
A	11	6	16	10	12	6	14	14	6	13	7	19	14	12	7	14	13	10	12	4	8	6	10	11	36	10	12	9	11	4	7	12	6	13	13	15	13	10	13	9	23	10	13	6	5	14	9	10	4	5	10	1.094	0.534	–	–	A
C	4	4	5	4	4	4	4	4	2	4	4	6	6	5	8	5	3	3	0	6	2	3	5	4	2	3	6	4	4	3	8	4	0	1	2	7	5	3	1	5	5	4	1	2	3	8	3	1	4	4	4	0.960	0.472	–	–	C
D	8	10	11	5	7	8	6	8	9	11	11	4	8	11	7	5	9	9	9	6	11	2	4	6	8	10	10	7	5	6	8	1	7	5	1	4	7	7	3	4	9	10	5	8	5	4	6	3	4	3	12	0.558	0.228	6.21E-01	n.s.	D
E	7	5	7	6	10	3	5	6	2	7	5	7	6	3	7	6	9	7	11	7	6	9	8	10	9	8	8	4	6	6	10	3	9	6	8	6	6	8	6	3	15	2	3	7	14	3	6	5	11	9	32	0.213	0.087	5.55E-08	*	E
F	8	10	1	6	4	3	9	7	5	8	7	5	6	6	12	5	3	11	5	4	3	5	7	9	2	9	7	7	5	5	2	7	4	8	4	9	5	6	3	8	7	9	12	6	8	8	8	5	8	4	8	0.788	0.317	–	–	F
G	8	15	15	12	8	15	7	8	7	9	8	7	5	12	12	13	11	8	1	6	6	16	6	10	11	9	12	15	11	11	14	5	5	10	5	11	14	6	13	5	9	9	8	9	8	10	10	11	14	5	14	0.679	0.243	–	–	G
H	2	4	8	6	2	4	1	1	4	2	3	1	7	4	3	3	2	2	11	4	5	3	6	3	6	1	5	4	5	6	3	5	4	5	7	5	4	5	4	4	6	5	0	6	2	2	4	5	6	3	8	0.508	0.257	1.21E-02	n.s.	H
I	3	14	10	6	6	9	8	5	9	7	9	8	2	7	9	5	3	8	5	4	5	7	8	5	3	8	6	7	9	5	6	7	9	2	7	7	9	5	7	6	7	6	9	11	7	8	5	10	3	11	10	0.684	0.246	–	–	I
K	6	11	5	7	1	4	9	3	3	8	7	5	8	2	7	6	6	2	5	3	7	4	11	4	4	6	6	6	5	4	8	7	1	3	7	12	6	10	4	4	8	10	4	5	6	3	5	10	6	8	12	0.487	0.217	9.58E-05	*	K
L	14	12	15	10	13	10	10	13	18	24	7	10	20	16	10	11	20	11	10	17	21	13	18	17	11	11	12	11	10	16	19	14	11	18	11	18	11	16	24	12	21	19	9	11	10	19	17	16	13	13	17	0.839	0.245	–	–	L
M	1	1	5	2	2	2	1	2	2	5	3	3	4	2	2	2	1	8	1	1	1	5	2	2	0	1	3	3	3	2	0	3	3	2	0	3	0	2	3	0	2	0	2	3	6	2	2	3	2	3	1	2.260	1.588	–	–	M
N	5	5	2	4	8	6	4	6	9	7	5	5	4	7	3	5	5	7	2	4	7	4	6	11	4	5	9	3	7	2	4	5	5	9	6	10	8	3	4	5	3	8	6	11	7	7	5	3	7	5	6	0.940	0.372	–	–	N
P	9	17	13	7	10	11	14	11	10	10	8	16	7	7	5	9	8	5	12	9	7	8	6	10	8	11	16	5	3	8	8	8	13	11	7	11	11	9	10	6	11	11	10	11	6	21	7	9	14	6	18	0.533	0.190	6.50E-04	n.s.	P
Q	5	6	6	3	4	7	9	7	6	11	9	3	2	6	1	6	5	4	4	6	1	5	6	3	1	3	4	8	0	4	6	4	3	5	6	5	4	2	3	3	8	3	2	5	3	5	9	7	7	5	16	0.300	0.146	5.55E-08	*	Q
R	5	5	4	5	4	6	8	6	5	9	9	11	10	7	19	6	3	12	8	10	7	8	8	5	10	10	6	8	8	10	16	10	3	6	10	9	8	8	10	3	6	11	8	9	10	7	11	4	7	7	11	0.718	0.280	–	–	R
S	21	10	17	12	30	13	14	12	16	8	18	13	15	14	10	13	9	16	9	14	15	9	20	16	20	12	12	24	14	13	14	21	20	12	13	10	17	22	18	12	11	13	10	21	11	17	14	16	27	15	17	0.886	0.277	–	–	S
T	8	7	8	5	8	11	7	14	8	5	3	3	6	4	15	7	9	7	6	9	14	2	6	8	6	3	6	4	6	10	10	8	7	3	6	6	13	9	6	9	13	6	9	5	5	10	8	6	10	8	13	0.572	0.230	1.15E-01	n.s.	T
V	7	4	8	11	14	5	5	8	8	12	10	11	10	13	15	12	11	10	6	5	9	24	9	7	13	13	9	10	7	10	11	5	9	13	10	8	9	3	10	6	9	11	12	6	15	11	7	12	13	12	15	0.651	0.237	–	–	V
W	2	1	3	0	3	1	1	2	1	2	3	2	4	1	2	2	1	2	3	1	5	2	4	2	1	1	5	20	2	1	3	0	1	2	2	2	3	0	0	4	3	1	5	2	0	1	2	5	2	15	0	–	–	–	–	W
Y	5	2	3	6	4	5	3	2	3	6	3	5	4	6	5	2	2	5	4	10	2	3	4	2	5	7	2	6	0	3	5	5	4	4	4	1	5	5	2	6	1	0	6	3	4	3	7	7	5	5	4	1.005	0.491	–	–	Y
total	139	149	162	127	154	133	139	139	133	168	139	144	148	145	159	137	133	147	124	130	142	138	154	145	160	141	156	165	121	129	162	134	124	138	129	159	158	139	144	114	177	148	134	147	135	163	145	148	167	146	228	0.633	0.060			total

	C50	C49	C48	C47	C46	C45	C44	C43	C42	C41	C40	C39	C38	C37	C36	C35	C34	C33	C32	C31	C30	C29	C28	C27	C26	C25	C24	C23	C22	C21	C20	C19	C18	C17	C16	C15	C14	C13	C12	C11	C10	C9	C8	C7	C6	C5	C4	C3	C2	C1	C0	C-index	SD	*P-value*	mark	
A	9	8	10	12	27	11	7	10	17	15	9	11	11	10	14	18	10	8	6	9	14	6	15	10	24	11	15	20	6	10	9	9	13	11	7	14	12	10	7	9	15	10	13	3	7	7	9	10	10	19	16	0.709	0.286	–	–	A
C	4	5	7	2	3	4	3	5	3	4	2	6	6	6	2	2	5	4	2	2	1	3	3	5	3	5	8	4	3	2	3	4	3	5	5	6	6	4	2	0	6	5	3	4	9	5	5	4	10	5	5	0.832	0.390	–	–	C
D	7	7	11	8	1	3	8	9	8	8	11	9	2	5	4	8	2	7	7	3	5	6	11	4	7	10	6	6	4	4	6	7	3	5	2	8	3	5	6	1	7	8	5	6	5	4	7	6	3	3	10	0.582	0.256	9.69E-01	n.s.	D
E	7	7	14	9	9	10	12	6	9	9	11	9	10	9	9	4	5	9	8	5	10	11	6	9	12	10	6	4	9	12	9	8	4	8	9	8	8	10	5	2	4	5	7	10	6	14	4	8	11	8	19	0.429	0.141	5.55E-08	*	E
F	4	6	10	2	4	2	5	4	5	6	6	0	6	10	7	4	4	2	4	12	5	14	7	2	2	6	3	5	3	7	6	4	2	2	4	3	3	6	4	7	6	3	2	2	5	8	5	6	6	5	7	0.703	0.382	–	–	F
G	9	2	7	5	3	7	10	4	7	6	9	8	7	6	14	5	14	8	9	3	4	5	12	15	9	10	2	14	5	11	11	2	7	7	8	16	5	20	8	5	6	15	8	7	5	2	6	9	10	11	13	0.612	0.308	6.88E-01	n.s.	G
H	7	6	5	2	2	4	7	7	2	0	4	2	6	2	3	4	5	4	5	2	1	2	5	3	1	4	2	2	2	4	3	1	3	2	4	4	5	5	3	4	5	3	1	5	5	4	2	2	7	2	10	0.350	0.178	5.55E-08	*	H
I	5	5	8	8	3	4	5	4	3	8	4	7	2	4	6	5	2	9	5	6	4	4	6	5	4	3	7	4	6	5	4	7	2	5	6	8	2	5	3	1	1	7	1	3	2	15	3	4	4	4	7	0.680	0.353	–	–	I
K	3	7	6	7	6	15	6	13	9	10	7	5	3	11	14	3	9	8	11	8	6	4	9	3	6	2	5	10	4	4	8	8	6	0	3	11	4	4	7	6	9	7	15	10	8	4	9	10	5	9	13	0.549	0.258	1.73E-01	n.s.	K
L	11	10	19	19	13	15	8	15	8	18	9	11	12	15	8	10	12	15	9	16	16	10	14	15	17	14	18	17	15	9	15	14	9	14	13	15	17	9	11	9	13	11	10	20	21	12	17	16	10	10	20	0.664	0.175	–	–	L
M	4	3	4	2	4	1	2	0	4	4	4	4	2	3	2	2	4	3	4	6	2	4	3	5	2	3	2	6	1	1	2	3	2	2	1	5	7	4	6	2	4	5	3	2	4	1	8	0	0	2	2	1.540	0.885	–	–	M
N	5	4	4	0	2	0	5	4	1	2	6	6	6	2	3	1	3	5	3	3	3	2	4	5	5	4	7	3	0	4	4	9	1	4	9	7	4	2	11	3	1	3	2	1	3	3	2	5	9	4	11	0.344	0.221	5.57E-08	*	N
P	14	13	16	7	7	10	8	4	7	16	8	10	11	11	10	13	9	9	11	8	12	18	20	11	13	9	16	13	13	9	12	10	13	14	5	8	12	2	8	8	11	10	7	11	13	9	14	11	21	12	10	1.094	0.371	–	–	P
Q	7	9	3	3	6	5	11	8	10	5	5	9	9	8	17	10	5	8	6	4	5	5	7	5	6	14	8	4	9	8	16	7	9	13	6	4	15	11	8	9	13	11	8	12	4	7	6	8	4	2	11	0.713	0.315	–	–	Q
R	10	8	6	9	10	5	8	8	8	6	6	7	7	9	7	10	7	6	5	10	11	9	5	8	10	9	15	10	6	4	9	8	3	9	5	8	9	7	13	7	19	9	7	12	5	16	6	8	10	1	10	0.820	0.314	–	–	R
S	11	23	16	15	24	15	11	18	8	17	11	15	19	14	16	16	12	15	10	17	13	14	15	19	19	9	12	14	14	12	16	8	10	14	18	12	16	17	23	14	14	16	26	20	12	18	22	26	23	18	25	0.630	0.176	9.98E-01	n.s.	S
T	6	12	5	5	11	4	9	8	9	18	11	11	11	5	6	10	8	10	6	5	11	5	3	6	9	9	8	12	9	9	13	6	18	11	10	7	13	5	9	7	16	7	10	8	10	10	6	7	6	12	10	0.884	0.332	–	–	T
V	8	11	4	7	11	13	9	4	8	12	10	9	10	10	8	5	11	8	3	8	7	10	5	6	7	4	8	11	9	8	7	9	7	6	6	9	9	6	5	9	8	8	4	8	7	12	9	5	9	6	16	0.491	0.146	6.99E-07	*	V
W	1	0	3	2	4	1	3	5	4	2	0	2	4	0	4	3	1	4	3	1	3	4	1	4	2	0	1	1	0	3	5	3	5	4	6	3	3	1	3	2	15	2	0	0	1	2	0	1	4	8	9	0.298	0.281	5.55E-08	*	W
Y	7	3	4	3	4	4	2	3	3	2	6	3	4	5	5	4	5	5	7	2	9	2	3	5	2	5	7	5	3	3	4	7	4	2	2	3	5	6	2	9	4	3	2	3	3	10	5	2	5	5	4	1.055	0.493	–	–	Y
total	139	149	162	127	154	133	139	139	133	168	139	144	148	145	159	137	133	147	124	130	142	138	154	145	160	141	156	165	121	129	162	134	124	138	129	159	158	139	144	114	177	148	134	147	135	163	145	148	167	146	228	0.633	0.060			total

Additional file 5. Continued.

HUMAN x-x-T-x-I

position -4

	C50	C49	C48	C47	C46	C45	C44	C43	C42	C41	C40	C39	C38	C37	C36	C35	C34	C33	C32	C31	C30	C29	C28	C27	C26	C25	C24	C23	C22	C21	C20	C19	C18	C17	C16	C15	C14	C13	C12	C11	C10	C9	C8	C7	C6	C5	C4	C3	C2	C1	C0	C-index	SD	*P-value*	mark	
A	4	3	2	5	0	10	6	6	7	4	2	2	5	5	6	4	6	1	4	2	3	3	8	5	3	7	4	1	5	2	2	3	6	2	3	3	2	3	17	4	1	3	4	4	2	1	3	3	2	6	7	0.569	0.393	5.48E-01	n.s.	A
C	0	1	1	2	3	3	3	2	2	4	2	3	2	3	3	0	4	2	0	0	0	2	1	1	0	1	5	3	3	3	0	5	5	0	2	1	1	2	2	3	1	4	1	0	1	3	1	5	0	3	5	0.396	0.301	3.26E-03	n.s.	C
D	9	4	1	4	3	2	5	0	2	4	5	2	4	2	6	3	6	0	4	2	1	3	2	5	2	4	3	1	3	1	2	2	1	5	4	3	2	5	3	4	2	2	0	1	0	2	3	2	3	3	6	0.473	0.296	9.78E-02	n.s.	D
E	4	1	5	6	4	5	5	2	4	2	3	4	6	4	2	3	5	2	5	5	5	4	3	1	3	0	4	3	0	5	5	2	1	3	0	2	1	1	3	4	7	2	2	2	6	6	3	3	2	5	7	0.471	0.252	2.27E-01	n.s.	E
F	0	4	4	4	1	2	4	2	10	1	3	7	11	1	6	5	7	14	7	8	2	9	5	4	3	3	2	2	3	3	5	2	1	3	2	3	3	1	2	2	2	2	2	7	3	0	2	3	10	0	4	0.960	0.765	−	−	F
G	3	8	1	5	3	3	4	6	3	6	4	6	2	9	3	1	0	3	8	6	4	2	8	2	4	1	4	1	10	2	2	1	4	5	8	8	1	3	6	6	7	2	2	7	3	3	7	4	3	6	2	2.100	1.245	−	−	G
H	2	1	2	0	1	1	1	0	0	2	0	1	3	2	1	0	2	9	1	3	0	2	1	2	3	3	0	1	0	3	0	1	1	2	0	2	2	0	3	1	0	0	1	3	4	2	0	1	4	2	3	0.507	0.527	6.16E-01	n.s.	H
I	5	2	1	4	3	4	5	9	7	5	6	3	0	4	2	4	6	4	3	4	5	3	5	7	4	2	3	4	2	7	5	2	8	1	6	4	3	5	2	3	3	3	4	3	2	4	7	3	2	1	3	1.293	0.641	−	−	I
K	7	8	6	3	4	0	2	4	11	4	5	0	7	2	7	5	1	2	6	5	10	8	8	8	1	2	9	3	2	3	2	2	1	1	6	4	1	3	7	8	3	2	3	8	12	4	5	5	4	6	10	0.460	0.294	9.06E-02	n.s.	K
L	5	9	6	7	9	11	9	5	11	5	8	11	5	8	7	10	11	7	4	8	8	9	9	5	12	7	10	7	5	8	4	4	3	7	10	5	6	7	5	9	4	3	9	7	7	9	9	9	5	9	11	0.667	0.212	−	−	L
M	1	2	0	2	1	1	0	4	2	1	1	2	2	1	0	1	1	0	5	6	1	3	1	2	3	3	5	2	1	2	0	2	1	0	0	0	3	0	2	0	1	2	1	0	3	1	2	1	2	1	3	0.520	0.462	5.71E-01	n.s.	M
N	3	5	0	2	1	5	5	10	1	2	4	6	3	2	3	3	4	1	2	13	4	3	6	4	3	3	3	3	2	2	0	3	1	2	2	5	3	3	1	1	0	2	6	0	2	5	3	2	5	2	10	0.312	0.237	5.57E-08	*	N
P	6	4	3	2	1	7	3	3	3	5	5	7	3	4	4	4	5	3	5	5	4	7	5	5	2	4	1	9	5	4	4	8	2	7	11	6	2	4	6	8	1	5	2	5	5	8	3	7	3	2	8	0.568	0.273	9.95E-01	n.s.	P
Q	2	3	3	4	2	5	0	3	4	6	1	3	4	2	4	0	6	3	2	4	3	0	5	6	4	2	0	1	0	6	4	2	4	0	6	4	3	3	3	2	4	4	3	1	2	1	5	7	2	1	6	0.497	0.308	8.22E-01	n.s.	Q
R	4	2	3	5	2	3	3	3	6	6	3	3	2	2	2	7	5	2	1	3	2	4	2	7	2	2	1	6	3	1	1	1	3	4	2	2	3	2	4	5	1	7	4	2	2	2	2	5	8	0	7	0.449	0.266	1.06E-03	n.s.	R
S	8	5	5	9	9	6	4	3	2	6	2	8	5	6	4	8	6	2	6	4	6	9	4	8	4	1	5	7	4	3	7	4	5	6	1	11	8	4	1	9	4	5	5	3	5	7	7	13	5	5	9	0.609	0.282	−	−	S
T	4	5	6	2	5	4	3	3	4	5	5	3	4	3	2	5	6	3	5	3	5	4	5	2	4	3	4	2	6	5	2	2	2	7	2	8	6	3	3	6	5	1	5	3	3	5	1	2	10	2	8	0.495	0.227	9.62E-02	n.s.	T
V	3	0	3	5	3	2	3	4	5	6	7	3	5	1	7	4	6	4	6	2	6	5	6	1	4	4	13	7	4	9	5	6	4	2	2	7	5	2	3	8	3	5	3	3	3	2	10	7	4	5	5	0.908	0.485	−	−	V
W	0	0	2	0	1	1	1	0	1	3	1	0	1	0	0	0	1	1	3	0	0	1	0	1	1	0	1	0	0	0	0	0	0	2	1	1	0	0	1	2	1	0	0	1	0	1	1	2	0	0	4	0.165	0.200	5.55E-08	*	W
Y	2	4	0	3	1	5	2	4	2	0	2	5	3	3	0	0	4	4	3	3	3	2	4	4	7	3	1	3	2	2	1	1	1	3	2	2	0	1	3	8	5	1	0	1	3	1	3	2	4	1	3	0.827	0.584	−	−	Y
total	72	71	54	74	57	80	68	73	87	77	69	79	77	64	69	67	92	67	80	86	72	83	88	80	69	55	78	66	60	71	51	53	54	62	70	81	55	52	77	93	55	55	57	61	68	67	77	86	78	60	121	0.578	0.094			total

position -3

	C50	C49	C48	C47	C46	C45	C44	C43	C42	C41	C40	C39	C38	C37	C36	C35	C34	C33	C32	C31	C30	C29	C28	C27	C26	C25	C24	C23	C22	C21	C20	C19	C18	C17	C16	C15	C14	C13	C12	C11	C10	C9	C8	C7	C6	C5	C4	C3	C2	C1	C0	C-index	SD	*P-value*	mark	
A	4	6	6	3	5	5	9	2	5	6	7	6	6	2	5	2	3	0	9	5	6	4	11	3	9	7	12	9	0	6	4	4	0	2	5	3	3	2	1	6	3	2	5	6	6	4	5	7	5	3	6	0.797	0.443	−	−	A
C	2	2	1	0	2	2	1	2	2	2	2	0	1	4	2	2	2	5	1	6	3	4	0	2	2	1	1	0	2	1	2	2	2	2	2	0	0	0	3	2	0	1	0	3	3	1	3	2	2	1	2	0.880	0.643	−	−	C
D	1	6	5	1	0	3	1	4	5	2	4	2	2	5	2	3	2	2	1	5	3	4	1	5	3	3	3	2	3	4	6	3	5	3	2	9	1	8	5	3	1	0	3	0	2	2	4	8	4	3	5	0.636	0.405	−	−	D
E	5	4	2	8	5	3	0	4	4	4	5	4	3	2	5	3	2	2	6	3	9	7	4	3	1	5	3	3	0	3	5	4	4	2	1	5	3	2	10	4	0	6	5	3	3	8	6	5	6	4	11	0.360	0.196	6.82E-08	*	E
F	3	2	3	3	1	5	1	4	6	1	3	1	3	2	1	3	4	7	2	2	2	0	5	5	5	1	0	3	2	4	0	0	3	2	1	3	4	2	0	5	4	1	2	4	2	3	3	4	1	3	1	2.620	1.665	−	−	F
G	10	4	1	3	2	9	4	5	3	5	7	9	5	2	1	3	5	3	5	5	3	7	2	6	6	4	3	8	4	5	2	3	3	4	4	9	5	2	6	2	5	4	2	4	4	5	7	3	8	1	8	0.555	0.280	7.67E-01	n.s.	G
H	3	0	1	2	1	1	1	1	4	2	1	3	5	3	1	2	2	7	0	4	4	2	3	1	1	2	4	2	4	2	1	0	1	2	5	1	1	1	1	4	1	0	2	1	1	1	4	2	3	0	3	0.673	0.511	−	−	H
I	0	2	2	3	7	6	6	1	4	3	4	3	2	2	2	1	9	2	5	5	1	5	12	5	3	2	10	2	3	2	5	1	3	4	2	3	1	3	3	2	4	1	3	2	4	6	0	3	1	2	10	0.334	0.242	6.41E-08	*	I
K	5	4	4	4	4	7	4	5	5	3	3	3	4	4	7	3	11	2	5	5	3	6	1	4	4	1	8	2	6	3	3	4	4	3	5	10	5	3	5	9	6	1	1	1	1	4	10	4	8	3	16	0.275	0.149	5.55E-08	*	K
L	2	5	9	9	7	5	7	10	6	3	3	9	15	8	6	11	7	6	5	11	10	8	9	8	6	6	8	5	8	6	4	5	3	4	9	2	3	9	3	13	6	8	7	8	7	7	3	7	12	4	11	0.622	0.261	−	−	L
M	2	1	2	1	1	2	3	1	2	3	2	1	0	1	1	5	0	0	3	2	0	2	0	0	0	0	0	2	0	1	0	1	1	1	0	1	0	0	2	1	0	2	0	1	2	0	3	1	1	0	3	0.367	0.370	3.28E-03	n.s.	M
N	3	4	1	5	2	1	1	2	5	3	1	0	1	5	6	4	5	4	1	4	3	5	7	1	0	1	3	5	2	1	4	1	1	2	1	1	7	5	3	2	1	3	2	1	7	4	2	7	2	2	2	1.440	0.993	−	−	N
P	2	7	3	6	3	5	1	1	2	9	4	5	4	3	4	2	2	3	6	2	1	4	2	6	2	3	3	5	3	4	1	3	2	3	7	4	0	3	3	6	3	4	2	2	5	1	3	3	2	4	7	0.480	0.259	8.46E-03	n.s.	P
Q	3	3	0	1	1	0	3	3	1	3	4	3	0	2	2	2	3	1	3	3	2	1	3	2	2	2	1	0	6	6	1	1	2	2	6	2	3	0	3	5	1	2	2	1	1	3	3	6	2	6	10	0.236	0.164	5.55E-08	*	Q
R	9	2	0	4	3	4	3	6	6	8	0	5	4	1	4	3	1	1	3	6	4	1	5	7	3	2	4	2	0	1	2	7	4	7	3	4	2	2	7	5	3	5	6	2	3	2	4	2	3	8	5	0.732	0.447	−	−	R
S	6	5	2	6	3	6	5	3	7	5	3	4	5	6	8	4	6	7	8	2	5	5	10	5	6	3	6	7	8	9	1	2	4	4	4	6	6	2	9	10	4	5	2	8	6	6	7	8	4	5	7	0.766	0.311	−	−	S
T	4	3	4	6	3	5	4	7	7	5	5	4	4	3	4	2	14	4	5	5	5	7	3	9	5	2	2	3	3	5	3	5	4	8	3	7	4	2	6	4	6	1	6	1	3	3	2	4	1	3	5	0.872	0.455	−	−	T
V	5	10	6	7	4	7	10	8	8	6	4	7	7	6	3	5	5	5	5	3	4	4	5	4	5	6	5	4	6	6	3	5	2	4	6	6	2	1	6	9	3	6	3	9	5	6	5	5	7	6	7	0.769	0.278	−	−	V
W	1	0	1	0	1	0	1	1	0	0	1	4	1	0	2	0	0	0	0	1	1	1	0	1	1	3	1	0	0	1	1	1	0	2	1	1	0	0	0	0	2	2	0	0	0	1	1	0	1	1	1	0.740	0.853	−	−	W
Y	2	1	1	2	2	4	3	3	5	4	6	6	5	3	3	7	9	6	7	7	3	6	5	3	5	1	1	2	0	1	3	1	6	1	3	4	5	5	1	1	2	1	4	4	3	0	2	5	5	1	1	3.400	2.148	−	−	Y
total	72	71	54	74	57	80	68	73	87	77	69	79	77	64	69	67	92	67	80	86	72	83	88	80	69	55	78	66	60	71	51	53	54	62	70	81	55	52	77	93	55	55	57	61	68	67	77	86	78	60	121	0.578	0.094			total

position -1

	C50	C49	C48	C47	C46	C45	C44	C43	C42	C41	C40	C39	C38	C37	C36	C35	C34	C33	C32	C31	C30	C29	C28	C27	C26	C25	C24	C23	C22	C21	C20	C19	C18	C17	C16	C15	C14	C13	C12	C11	C10	C9	C8	C7	C6	C5	C4	C3	C2	C1	C0	C-index	SD	*P-value*	mark	
A	5	5	2	2	4	6	5	6	9	7	3	4	3	7	3	1	6	8	5	7	4	2	3	14	6	3	10	8	0	3	3	2	3	11	5	4	8	3	5	2	3	2	3	1	4	3	6	6	5	2	5	0.928	0.549	−	−	A
C	1	2	0	1	1	2	4	0	2	1	0	3	2	2	2	0	2	1	3	0	1	2	1	1	1	2	1	2	0	5	1	1	2	2	1	0	1	2	2	2	5	3	2	3	6	1	1	1	7	2	2	0.900	0.749	−	−	C
D	2	2	5	0	3	4	3	1	6	1	5	3	4	2	4	2	2	2	2	4	4	4	3	4	2	1	2	4	0	2	1	2	3	3	4	3	2	6	11	5	2	2	3	1	3	1	1	2	7	9	4	0.770	0.525	−	−	D
E	3	3	4	5	4	5	7	2	1	8	7	2	5	0	5	3	6	4	5	5	4	11	0	3	3	3	6	4	6	5	1	3	5	4	5	9	3	1	2	4	3	1	3	7	3	3	9	7	4	3	12	0.348	0.193	5.93E-08	*	E
F	1	2	1	1	5	6	3	3	6	1	2	3	2	4	3	0	4	7	7	2	0	2	10	3	4	1	0	2	2	2	4	3	4	5	4	1	6	3	1	3	2	2	0	2	1	1	0	5	3	1	5	0.560	0.424	7.08E-01	n.s.	F
G	7	6	3	9	6	6	6	5	3	6	7	6	5	4	8	10	7	5	8	7	2	6	8	4	4	2	2	2	1	2	8	5	1	5	3	7	2	5	0	5	3	4	0	4	1	4	1	1	4	4	9	0.498	0.274	6.43E-01	n.s.	G
H	2	1	0	1	1	1	4	2	0	2	1	3	5	5	0	2	1	2	2	2	2	1	3	2	1	4	5	2	3	8	1	3	5	2	4	3	1	2	0	3	3	1	3	2	3	2	2	3	3	1	3	0.767	0.518	−	−	H
I	7	2	1	5	2	4	3	5	3	2	5	3	5	5	5	6	10	2	3	4	5	3	4	0	3	3	2	3	6	7	1	1	3	0	4	1	2	0	2	6	2	4	1	1	2	4	5	3	1	2	10	0.326	0.206	5.95E-08	*	I
K	1	7	1	3	1	5	0	1	5	2	7	1	2	3	3	3	1	2	3	2	4	4	4	3	5	2	2	6	1	1	1	3	1	3	4	1	0	4	6	7	5	4	3	3	8	2	6	7	3	3	5	0.636	0.409	−	−	K
L	9	3	3	16	6	6	8	7	10	7	5	10	5	2	7	8	12	5	5	11	9	6	9	6	7	6	7	6	7	8	7	3	5	2	6	7	2	5	10	11	5	6	8	2	7	6	10	6	12	5	8	0.853	0.359	−	−	L
M	2	1	1	0	0	1	1	3	1	2	0	1	1	1	2	3	5	2	0	1	2	0	2	4	0	1	1	2	0	1	0	0	1	2	0	3	1	0	1	0	0	0	1	1	1	2	0	2	0	0	4	0.280	0.284	7.81E-08	*	M
N	3	3	3	2	2	2	2	4	3	4	6	5	1	2	2	4	1	3	1	3	1	2	4	4	5	3	2	3	5	4	1	2	1	1	2	4	3	2	3	3	2	2	2	3	3	2	1	3	5	1	6	0.450	0.211	4.84E-03	n.s.	N
P	2	2	3	4	3	2	1	2	5	5	3	4	2	1	0	5	3	1	3	7	3	5	3	8	8	5	9	3	7	4	5	4	2	3	6	8	2	1	1	5	2	2	7	3	3	7	6	11	3	2	2	1.960	1.207	−	−	P
Q	1	7	3	3	1	5	2	4	3	5	2	3	2	3	1	3	2	7	1	3	5	5	3	2	3	3	2	4	0	0	1	2	3	1	2	7	1	1	1	3	6	4	1	4	1	1	4	2	2	3	7	0.394	0.251	9.49E-06	*	Q
R	5	7	6	2	2	4	0	5	9	6	0	1	7	1	5	3	6	2	5	6	3	6	4	3	4	0	4	4	4	5	0	6	2	4	1	1	3	2	4	7	5	3	1	6	6	5	6	7	1	4	4	0.965	0.560	−	−	R
S	4	7	5	5	3	10	6	3	9	4	5	6	8	6	6	3	5	2	6	6	6	6	9	3	4	5	8	3	4	5	3	4	6	10	5	9	6	4	7	8	1	2	8	3	4	5	6	5	9	7	18	0.304	0.120	5.55E-08	*	S
T	3	4	4	6	6	1	2	7	5	5	2	5	9	3	5	1	8	3	3	2	6	6	2	8	3	0	3	1	3	1	3	5	1	1	3	4	3	1	9	4	3	3	6	3	3	3	4	4	3	5	4	0.940	0.536	−	−	T
V	7	4	4	7	6	6	5	8	2	8	6	12	7	9	6	7	5	8	15	12	9	9	9	5	2	3	9	5	7	4	7	3	1	2	6	7	2	5	7	10	1	8	4	4	6	11	5	8	2	5	7	0.886	0.428	−	−	V
W	4	2	0	0	0	1	1	2	1	0	1	1	0	1	1	0	3	1	0	2	0	1	0	0	2	0	2	0	2	2	0	0	1	1	3	2	3	1	1	2	1	1	0	4	0	2	3	1	2	0	2	0.580	0.557	−	−	W
Y	3	1	5	2	1	3	5	3	4	1	2	3	2	3	1	3	3	0	3	0	2	2	7	3	2	8	1	2	2	2	3	1	4	0	2	0	4	4	4	3	1	1	1	4	3	2	1	2	2	1	4	0.610	0.411	−	−	Y
total	72	71	54	74	57	80	68	73	87	77	69	79	77	64	69	67	92	67	80	86	72	83	88	80	69	55	78	66	60	71	51	53	54	62	70	81	55	52	77	93	55	55	57	61	68	67	77	86	78	60	121	0.578	0.094			total

Additional file 5. Continued.

HUMAN x-x-T-x-L

position -4

	C50	C49	C48	C47	C46	C45	C44	C43	C42	C41	C40	C39	C38	C37	C36	C35	C34	C33	C32	C31	C30	C29	C28	C27	C26	C25	C24	C23	C22	C21	C20	C19	C18	C17	C16	C15	C14	C13	C12	C11	C10	C9	C8	C7	C6	C5	C4	C3	C2	C1	C0	C-index	SD	*P-value*	mark	
A	18	15	14	13	14	7	18	20	9	11	19	13	10	10	11	9	9	21	9	21	23	18	16	8	5	11	10	13	9	6	9	11	8	8	12	14	9	8	17	6	11	12	6	11	6	12	12	10	8	12	23	0.515	0.192	–	–	A
C	5	3	2	3	3	1	6	3	6	3	4	5	5	2	5	2	5	9	2	2	1	3	3	5	2	2	5	4	4	7	2	4	1	5	4	2	5	7	4	3	7	3	2	3	3	3	5	3	7	1	3	1.240	0.610	–	–	C
D	2	12	8	9	3	9	3	2	4	6	6	5	6	6	11	8	4	2	5	4	3	6	6	8	3	2	5	9	4	5	2	5	7	6	3	9	5	4	6	3	5	5	6	2	4	7	5	3	6	5	17	0.311	0.140	5.56E-08	*	D
E	5	16	10	13	5	9	3	13	12	5	13	11	14	10	12	9	10	8	9	7	10	4	8	7	24	9	4	11	5	6	6	8	9	10	9	10	9	9	13	6	4	2	7	9	6	11	6	7	9	8	22	0.400	0.170	7.46E-06	*	E
F	6	7	8	8	8	9	7	3	9	8	13	3	8	11	9	5	5	22	13	10	12	9	3	4	5	7	9	2	4	5	7	7	7	9	6	6	6	4	7	4	18	9	10	11	9	4	6	4	3	4	13	0.574	0.289	–	–	F
G	9	7	9	13	14	10	10	10	8	14	11	4	14	8	14	9	11	9	5	6	28	13	11	4	5	19	14	15	10	4	8	8	9	13	5	8	15	5	14	9	7	13	15	9	13	4	5	6	4	5	13	0.758	0.353	–	–	G
H	7	2	4	4	5	5	5	8	4	3	4	3	5	4	1	1	1	4	1	2	6	5	4	7	5	3	4	12	10	3	3	3	3	3	5	2	3	6	3	2	5	4	5	3	3	8	8	5	5	4	7	0.614	0.319	–	–	H
I	7	4	6	9	7	9	8	5	8	15	10	15	5	6	7	11	10	2	6	12	15	10	11	11	16	4	10	7	7	4	7	4	12	8	4	7	10	9	8	16	9	9	3	8	8	3	8	5	7	4	3	2.707	1.154	–	–	I
K	5	5	2	5	3	9	9	6	6	7	11	7	8	7	8	11	5	5	6	6	6	7	4	3	9	3	6	10	1	6	8	7	5	5	6	13	10	6	9	7	9	8	8	9	8	9	6	2	7	6	29	0.230	0.085	5.55E-08	*	K
L	16	13	17	20	19	17	16	26	15	18	18	10	11	25	11	20	28	15	19	15	20	18	24	13	17	14	20	12	15	11	18	19	16	21	17	9	14	18	23	24	23	16	21	18	19	14	11	21	26	19	29	0.607	0.153	–	–	L
M	3	2	5	3	0	3	3	5	7	3	4	3	4	6	5	6	4	5	1	3	5	4	1	3	6	3	2	4	3	6	2	2	1	5	3	0	2	4	4	3	3	3	3	9	4	6	5	2	2	2	10	0.354	0.179	5.75E-06	*	M
N	8	2	7	4	3	3	3	7	5	4	6	7	10	1	4	7	8	6	7	6	8	9	4	6	4	1	2	8	7	6	8	4	5	3	9	3	7	4	7	8	5	9	10	4	5	6	10	3	7	8	11	0.524	0.218	–	–	N
P	6	10	18	9	14	10	13	7	12	12	9	7	9	13	11	11	11	13	14	6	8	14	18	15	9	10	7	15	9	9	17	10	10	13	12	10	13	7	10	13	11	10	8	10	6	9	16	9	9	9	26	0.416	0.116	3.23E-05	*	P
Q	6	3	6	5	7	6	6	9	15	2	7	4	10	12	8	11	3	7	5	6	5	7	6	4	4	14	10	5	5	13	3	6	7	7	6	4	12	4	7	5	8	5	8	6	7	9	10	2	10	5	15	0.456	0.201	1.23E-02	n.s.	Q
R	5	3	13	4	5	8	15	6	7	8	6	8	8	9	9	7	9	3	10	5	15	5	9	6	10	14	2	11	9	15	14	9	3	12	8	4	11	3	7	5	9	3	15	8	10	2	13	7	10	11	17	0.480	0.218	9.22E-01	n.s.	R
S	11	11	11	13	23	10	10	13	11	14	12	16	13	13	7	16	22	13	14	18	16	10	8	12	15	20	16	11	11	12	17	13	11	20	14	17	16	24	21	14	14	10	9	16	12	18	14	16	15	19	29	0.491	0.133	5.60E-01	n.s.	S
T	13	8	8	10	2	5	7	13	8	11	11	13	11	11	15	8	9	8	12	5	10	7	4	4	11	10	6	8	10	11	8	9	12	11	7	7	8	9	23	4	10	6	7	7	9	2	10	9	3	15	15	0.593	0.244	–	–	T
V	8	8	16	9	5	9	7	12	9	13	12	9	15	14	18	10	11	12	14	11	15	19	8	12	9	13	14	16	13	8	11	13	8	19	16	12	13	14	12	9	11	13	8	6	14	11	8	13	7	11	12	0.963	0.274	–	–	V
W	4	3	2	1	5	1	2	1	1	1	1	1	0	3	2	2	0	1	1	0	3	2	0	2	3	3	4	5	3	0	6	2	1	5	1	2	2	5	3	2	3	4	2	1	1	3	5	2	2	2	4	0.555	0.379	–	–	W
Y	5	3	10	3	4	2	6	5	4	0	4	4	3	3	6	7	6	2	5	4	5	7	5	3	2	8	4	0	6	3	4	0	1	1	2	0	3	9	4	4	6	8	2	3	6	6	1	8	5	2	13	0.314	0.185	7.08E-08	*	Y
total	149	137	176	158	149	142	157	174	160	158	181	148	169	174	174	170	171	167	158	149	214	177	153	137	164	170	154	178	145	140	160	144	136	184	149	139	173	159	202	147	178	152	155	153	153	147	164	137	152	152	311	0.514	0.053			total

position -3

	C50	C49	C48	C47	C46	C45	C44	C43	C42	C41	C40	C39	C38	C37	C36	C35	C34	C33	C32	C31	C30	C29	C28	C27	C26	C25	C24	C23	C22	C21	C20	C19	C18	C17	C16	C15	C14	C13	C12	C11	C10	C9	C8	C7	C6	C5	C4	C3	C2	C1	C0	C-index	SD	*P-value*	mark	
A	12	7	15	9	12	2	10	12	5	19	12	11	8	14	10	8	10	8	8	8	13	13	15	6	8	10	9	13	4	6	8	8	5	10	11	11	11	9	10	11	15	7	10	4	8	5	8	7	4	11	13	0.723	0.258	–	–	A
C	2	6	6	2	2	2	5	7	4	6	4	3	3	5	6	2	5	7	4	1	11	3	7	0	3	5	2	3	3	3	2	3	0	14	2	3	3	3	3	4	0	4	1	1	4	2	2	4	3	2	12	0.303	0.213	6.05E-08	*	C
D	6	3	7	6	7	8	0	9	10	6	7	9	4	18	9	8	3	4	9	8	9	11	8	6	24	8	7	5	5	7	4	11	5	8	10	5	5	7	7	10	16	9	6	7	6	7	4	6	9	4	23	0.328	0.167	5.55E-08	*	D
E	4	12	9	5	7	4	7	11	6	4	5	10	9	4	5	15	5	11	14	13	5	8	2	10	8	11	9	9	6	9	5	5	8	9	9	5	19	5	4	7	7	9	11	7	13	2	8	8	11	7	39	0.203	0.088	5.55E-08	*	E
F	2	5	7	8	7	9	7	8	6	8	12	8	10	9	4	11	8	7	3	4	8	7	7	10	4	9	5	3	6	0	6	2	6	11	5	8	5	6	8	9	7	7	6	7	6	7	7	2	2	7	4	1.630	0.637	–	–	F
G	13	4	7	14	5	15	15	6	7	10	14	13	8	12	14	12	15	13	3	10	11	14	16	11	8	10	8	10	12	7	17	11	9	12	13	11	13	8	19	8	13	12	14	11	8	8	7	7	6	12	14	0.766	0.249	–	–	G
H	2	4	3	3	4	5	8	0	2	4	5	6	5	3	3	3	1	3	6	5	5	1	8	6	5	2	3	8	9	9	7	6	7	4	6	4	4	3	3	7	1	3	7	1	5	7	7	3	4	2	9	0.493	0.250	9.72E-01	n.s.	H
I	6	10	4	10	4	4	10	8	8	10	10	9	13	5	15	9	6	6	7	12	6	14	9	12	7	5	3	9	10	6	9	8	4	13	11	3	5	7	14	4	9	4	6	7	4	5	6	6	5	17	13	0.606	0.261	–	–	I
K	9	12	10	7	9	12	10	14	16	5	11	6	13	7	7	4	9	16	9	7	9	10	4	8	4	7	11	16	9	8	9	11	6	5	7	12	8	10	12	9	12	15	9	11	8	7	11	7	7	15	17	0.553	0.185	–	–	K
L	14	10	28	14	20	20	26	24	21	15	18	12	15	17	16	19	34	18	14	18	27	23	14	17	8	16	18	21	13	17	17	14	22	17	16	12	20	22	22	16	18	17	10	18	18	20	21	8	16	8	36	0.488	0.143	6.49E-01	n.s.	L
M	3	4	5	7	4	4	1	2	5	3	2	1	1	4	5	5	4	1	4	1	1	2	6	1	2	5	3	3	7	1	3	1	2	6	2	1	4	6	1	1	3	4	1	5	2	5	4	2	5	4	7	0.454	0.258	9.74E-01	n.s.	M
N	5	4	9	7	6	7	8	6	4	5	8	5	7	10	3	10	4	6	5	3	2	4	5	4	12	13	3	7	6	5	5	3	5	4	3	7	1	6	8	4	2	3	8	7	3	4	8	6	6	5	5	1.124	0.500	–	–	N
P	8	6	6	8	9	7	5	4	7	12	9	7	8	7	19	6	6	9	11	8	23	4	7	5	5	7	9	6	4	6	10	2	12	9	12	12	18	8	13	13	5	7	15	3	12	16	11	11	11	11	16	0.561	0.264	–	–	P
Q	12	5	7	10	5	8	4	14	14	7	8	5	7	9	4	8	13	4	9	5	10	8	5	7	11	8	8	6	8	1	8	6	8	7	7	4	4	9	12	5	12	7	4	7	12	9	9	10	9	5	15	0.512	0.192	1.00E+00	n.s.	Q
R	11	16	12	7	11	6	12	12	9	7	9	6	14	9	7	5	11	4	9	18	16	13	11	7	9	11	8	12	13	10	15	9	6	10	8	7	9	8	14	5	9	10	9	17	15	7	11	9	14	7	15	0.672	0.222	–	–	R
S	17	5	18	12	17	11	11	9	6	16	14	9	18	13	12	17	13	10	7	9	10	14	7	4	15	17	14	12	7	16	13	18	11	14	7	11	11	11	15	8	9	19	20	16	13	12	15	14	22	10	27	0.466	0.152	5.01E-01	n.s.	S
T	11	7	8	10	8	8	7	8	9	8	18	8	8	9	6	9	3	19	2	6	27	9	10	6	10	11	13	16	6	8	12	12	9	9	8	11	9	7	12	6	17	3	5	9	4	10	9	6	9	6	14	0.659	0.310	–	–	T
V	9	6	12	13	9	8	5	11	13	8	10	13	12	15	17	11	15	7	19	7	12	12	5	15	16	7	14	16	8	15	3	7	4	15	6	5	13	12	19	12	15	7	4	9	5	9	9	11	5	16	24	0.438	0.176	2.78E-01	n.s.	V
W	2	0	0	0	1	1	1	1	1	2	0	1	3	0	4	6	3	4	5	1	1	2	1	0	2	3	2	0	3	0	4	2	2	4	3	5	2	0	4	0	5	2	3	5	3	3	2	4	3	3	5	0.436	0.330	7.61E-01	n.s.	W
Y	1	11	3	6	2	1	5	8	7	3	5	6	3	4	8	2	3	10	10	5	8	5	6	2	3	5	5	3	6	6	3	5	5	3	3	2	9	12	2	8	3	3	6	1	4	2	5	6	1	0	3	1.567	0.931	–	–	Y
total	149	137	176	158	149	142	157	174	160	158	181	148	169	174	174	170	171	167	158	149	214	177	153	137	164	170	154	178	145	140	160	144	136	184	149	139	173	159	202	147	178	152	155	153	153	147	164	137	152	152	311	0.514	0.053			total

position -1

	C50	C49	C48	C47	C46	C45	C44	C43	C42	C41	C40	C39	C38	C37	C36	C35	C34	C33	C32	C31	C30	C29	C28	C27	C26	C25	C24	C23	C22	C21	C20	C19	C18	C17	C16	C15	C14	C13	C12	C11	C10	C9	C8	C7	C6	C5	C4	C3	C2	C1	C0	C-index	SD	*P-value*	mark	
A	10	10	16	13	13	11	13	22	10	9	11	12	9	19	8	8	13	9	9	13	18	8	15	11	13	20	11	13	11	9	14	12	12	16	14	14	12	15	14	7	12	11	8	11	12	7	13	8	10	4	25	0.474	0.138	2.03E-01	n.s.	A
C	9	3	4	10	2	2	3	2	6	3	3	7	6	6	5	3	5	5	5	2	6	5	5	3	3	5	3	2	5	3	4	2	2	8	4	1	5	5	3	5	3	5	4	5	7	7	6	5	5	3	11	0.400	0.174	3.43E-05	*	C
D	5	6	8	3	6	7	7	5	9	6	7	10	13	5	8	12	6	5	10	3	7	4	3	5	3	9	11	6	10	8	2	8	5	11	4	6	6	3	8	4	4	10	7	3	7	1	4	8	6	9	17	0.380	0.161	1.69E-05	*	D
E	7	7	12	9	2	10	6	9	15	12	6	8	7	8	11	12	11	17	10	7	17	11	6	10	7	6	8	12	8	8	6	15	6	12	10	10	6	11	7	7	10	4	6	5	11	8	15	9	7	3	26	0.344	0.128	5.57E-08	*	E
F	5	4	5	4	6	5	6	8	2	7	6	6	5	0	7	13	11	4	7	2	7	5	8	7	7	5	7	9	7	6	11	6	5	12	7	2	10	8	14	12	16	5	13	7	10	4	7	2	12	7	11	0.638	0.306	–	–	F
G	8	10	11	9	9	8	12	13	12	6	12	10	13	12	13	11	12	12	7	10	5	16	12	14	3	14	11	7	11	12	18	6	12	8	9	11	11	6	16	3	11	10	7	9	8	10	5	12	8	9	11	0.916	0.287	–	–	G
H	4	11	7	3	6	6	1	3	6	4	5	5	1	8	6	2	4	6	5	4	25	6	4	5	8	3	3	8	2	3	5	5	5	5	7	4	7	8	8	4	5	6	4	6	3	6	3	4	7	5	18	0.301	0.192	5.55E-08	*	H
I	6	6	7	11	14	9	7	6	5	7	16	7	15	14	5	4	7	7	12	13	12	16	10	7	8	12	10	7	4	6	10	5	5	8	8	7	6	10	12	10	11	9	9	4	12	4	0	4	9	7	11	0.764	0.318	–	–	I
K	5	4	6	2	6	5	5	10	5	2	11	5	4	5	5	7	8	7	6	7	12	13	7	5	7	6	0	6	8	13	5	8	9	3	7	3	6	3	7	4	8	8	4	6	8	5	4	6	5	8	24	0.258	0.110	5.55E-08	*	K
L	11	15	24	20	10	16	19	20	18	14	15	18	22	21	17	19	13	23	19	12	14	12	13	18	20	8	19	28	11	18	17	17	14	14	12	12	13	20	24	15	20	19	22	21	13	21	21	20	20	17	20	0.859	0.211	–	–	L
M	4	4	2	1	2	1	4	6	2	5	3	2	2	3	5	3	1	2	5	3	5	4	2	5	9	4	1	5	3	1	2	3	4	1	2	4	5	3	2	2	8	1	2	5	0	2	1	2	0	3	2	1.510	0.939	–	–	M
N	5	4	8	7	5	6	3	4	9	7	4	4	3	1	8	5	4	3	0	6	8	5	3	6	22	6	2	5	3	6	3	1	3	8	2	4	13	6	2	3	3	6	14	1	6	4	8	3	4	9	4	1.325	0.925	–	–	N
P	9	7	13	7	13	8	13	5	11	8	14	10	12	5	11	10	15	9	13	12	25	16	10	10	14	16	9	15	13	9	9	11	10	8	12	10	16	11	10	5	8	9	6	11	4	7	9	4	18	9	11	0.962	0.353	–	–	P
Q	7	4	4	11	7	8	11	7	6	8	9	5	10	10	10	5	5	6	4	7	7	9	10	4	1	6	12	9	7	9	2	7	6	10	6	3	9	7	10	9	8	6	6	5	10	6	7	3	5	5	9	0.773	0.279	–	–	Q
R	11	8	7	9	10	3	5	5	8	12	13	10	11	9	10	7	6	6	9	8	3	8	6	2	7	12	9	10	6	2	9	6	1	6	4	7	6	8	7	11	14	5	6	12	13	14	16	7	9	10	30	0.269	0.111	5.55E-08	*	R
S	12	7	13	13	8	14	21	13	8	14	9	10	11	15	15	12	18	13	15	10	12	10	15	5	13	13	14	14	14	11	18	10	18	16	10	14	9	9	31	24	13	13	17	18	11	13	17	14	8	21	47	0.288	0.096	5.55E-08	*	S
T	8	11	8	4	15	5	4	12	8	15	11	9	3	11	7	16	10	14	9	12	8	6	7	8	7	7	8	7	7	6	5	5	9	17	14	8	9	8	4	6	10	6	4	7	4	10	8	7	6	6	13	0.640	0.257	–	–	T
V	13	9	14	15	9	12	9	21	10	10	14	7	13	13	14	11	13	11	9	9	14	14	14	9	4	16	8	7	8	7	11	9	7	12	12	10	17	8	19	11	9	16	9	10	7	11	6	8	10	9	13	0.843	0.263	–	–	V
W	2	2	2	5	1	3	2	1	2	3	2	1	5	2	2	3	2	4	3	2	1	4	1	1	2	1	0	3	2	0	3	2	1	2	2	4	3	6	2	0	2	1	4	0	4	3	5	8	2	3	5	0.484	0.318	2.70E-01	n.s.	W
Y	8	5	5	2	5	3	6	2	8	6	10	2	4	7	7	7	7	4	1	7	8	5	2	2	6	1	8	5	5	3	6	6	2	7	3	5	4	4	2	5	3	2	3	7	3	4	9	3	1	5	3	1.567	0.766	–	–	Y
total	149	137	176	158	149	142	157	174	160	158	181	148	169	174	174	170	171	167	158	149	214	177	153	137	164	170	154	178	145	140	160	144	136	184	149	139	173	159	202	147	178	152	155	153	153	147	164	137	152	152	311	0.514	0.053			total

Additional file 5. Continued.

HUMAN x-x-T-x-V

position -4

	C50	C49	C48	C47	C46	C45	C44	C43	C42	C41	C40	C39	C38	C37	C36	C35	C34	C33	C32	C31	C30	C29	C28	C27	C26	C25	C24	C23	C22	C21
A	3	4	4	5	2	11	6	8	5	13	6	11	8	6	8	8	2	10	6	9	7	7	3	5	6	13	8	7	2	9
C	3	3	3	1	2	2	1	0	1	2	1	1	1	0	1	0	2	2	0	1	1	2	1	1	1	0	2	1	1	0
D	1	3	9	6	5	3	2	3	9	7	5	6	1	3	6	2	5	2	5	1	3	8	8	4	4	7	7	2	5	3
E	15	5	8	4	6	5	7	6	6	3	11	5	11	1	6	6	10	5	3	6	10	2	5	2	3	4	13	0	6	6
F	9	2	3	6	4	3	3	8	5	6	3	7	6	2	1	11	5	15	6	16	14	17	5	4	4	6	7	3	3	2
G	2	9	5	7	7	8	9	3	6	11	4	9	5	9	4	9	7	11	14	11	8	8	9	12	4	7	9	10	8	7
H	2	0	5	2	3	4	1	2	3	3	3	0	2	3	2	5	1	2	3	3	1	3	1	0	3	3	1	3	3	3
I	10	1	5	5	4	9	7	2	4	6	6	6	2	6	4	3	5	7	8	5	5	6	8	20	6	6	7	5	1	4
K	4	5	3	1	3	3	6	6	6	8	2	7	5	5	6	5	5	2	5	0	5	1	4	4	4	7	4	4	5	2
L	8	13	11	8	4	11	6	11	5	8	13	10	4	12	7	13	7	17	6	16	8	11	9	8	10	11	18	10	7	8
M	2	2	2	1	2	2	2	1	2	6	3	3	3	5	0	1	2	4	5	5	6	3	6	2	2	4	1	0	1	0
N	4	2	2	3	3	4	4	2	6	4	4	2	3	5	5	5	5	2	4	3	5	3	7	4	1	4	3	1	1	5
P	7	5	9	6	7	4	1	2	8	1	4	4	5	4	6	7	5	10	5	8	7	6	9	4	5	9	3	7	3	8
Q	7	3	3	3	6	4	4	3	1	5	6	7	2	6	2	2	4	2	1	1	7	2	3	4	5	8	3	2	4	1
R	5	3	5	10	3	7	6	4	5	6	7	5	6	5	2	5	4	9	7	2	8	1	5	6	1	5	2	6	3	6
S	11	9	11	5	6	8	11	8	14	10	10	6	8	3	10	15	3	12	6	8	7	11	10	5	8	15	9	8	10	7
T	4	6	4	2	5	7	5	3	6	6	8	5	12	2	8	7	2	7	8	11	7	7	6	3	4	10	9	3	4	4
V	4	9	6	5	2	1	5	1	2	7	4	8	5	4	1	7	5	9	11	8	4	6	3	5	4	9	11	10	4	8
W	2	1	1	1	2	4	1	3	2	1	1	1	1	1	1	1	3	5	1	1	3	1	0	1	1	3	0	2	3	5
Y	2	0	7	2	6	2	4	3	4	1	3	2	3	2	2	4	4	2	2	1	1	2	0	1	2	1	1	3	3	3
total	105	85	106	83	82	102	91	79	100	114	104	105	93	84	82	116	86	135	106	116	117	107	102	95	78	132	118	87	77	91

	C20	C19	C18	C17	C16	C15	C14	C13	C12	C11	C10	C9	C8	C7	C6	C5	C4	C3	C2	C1	C0	C-index	SD	*P-value*	mark	
A	5	5	1	9	7	7	3	8	12	3	5	8	8	3	5	6	3	3	4	7	3	2.093	0.971	–	–	A
C	0	2	3	2	3	0	4	1	3	5	1	1	4	2	1	2	3	2	3	1	1	1.600	1.178	–	–	C
D	2	5	6	6	2	1	2	3	4	2	4	3	7	1	4	1	3	0	6	5	2	2.020	1.152	–	–	D
E	1	9	10	11	3	2	5	2	10	3	5	8	5	5	4	5	9	1	2	5	14	0.407	0.239	8.96E-02	n.s.	E
F	4	2	3	1	3	7	1	2	1	4	4	5	2	2	7	2	4	4	0	2	8	0.615	0.485	–	–	F
G	7	5	5	7	9	3	5	3	11	3	3	5	7	7	7	12	7	4	12	4	11	0.651	0.262	–	–	G
H	1	4	1	0	0	3	2	0	1	3	2	3	3	1	1	0	1	0	4	5	8	0.263	0.177	6.63E-08	*	H
I	6	6	6	4	4	3	9	8	3	6	2	0	3	4	4	8	3	6	10	2	13	0.415	0.241	3.95E-01	n.s.	I
K	6	0	7	7	3	6	2	6	6	4	5	2	12	5	6	8	2	4	7	7	17	0.273	0.134	5.55E-08	*	K
L	10	9	2	9	11	14	10	9	13	14	11	11	7	8	6	3	7	8	11	11	22	0.431	0.153	9.41E-01	n.s.	L
M	0	0	2	2	1	1	2	4	3	1	0	3	1	1	3	2	2	1	1	1	4	0.545	0.406	–	–	M
N	5	4	2	5	3	6	2	2	6	3	3	4	2	3	6	2	2	1	3	4	10	0.348	0.150	1.32E-03	n.s.	N
P	4	7	4	5	1	4	5	10	5	5	7	4	5	3	6	5	9	6	2	8	13	0.422	0.177	6.81E-01	n.s.	P
Q	3	1	0	0	4	2	4	1	11	2	6	8	2	5	1	2	5	6	10	3	13	0.288	0.193	4.13E-06	*	Q
R	3	2	4	1	3	10	1	8	6	6	5	2	7	4	7	1	3	0	6	5	13	0.358	0.186	2.34E-02	n.s.	R
S	6	1	10	6	5	8	5	10	4	10	8	13	6	10	5	11	11	15	11	10	22	0.390	0.145	1.34E-01	n.s.	S
T	6	8	4	5	4	2	6	7	2	2	9	5	5	7	4	8	7	2	8	5	15	0.375	0.164	4.35E-02	n.s.	T
V	13	4	4	11	7	14	22	18	4	9	3	6	3	5	4	5	4	7	3	2	17	0.372	0.247	5.69E-03	n.s.	V
W	0	3	1	3	2	2	1	3	1	1	0	0	1	1	2	0	1	3	0	1	2	0.790	0.615	–	–	W
Y	2	5	2	3	1	4	1	1	3	2	2	0	5	1	8	0	1	1	2	1	2	1.180	0.862	–	–	Y
total	84	82	77	97	76	99	92	106	109	88	85	91	95	78	91	83	87	74	105	89	210	0.454	0.069			total

position -3

	C50	C49	C48	C47	C46	C45	C44	C43	C42	C41	C40	C39	C38	C37	C36	C35	C34	C33	C32	C31	C30	C29	C28	C27	C26	C25	C24	C23	C22	C21
A	10	7	6	4	6	8	11	9	5	12	8	4	10	7	4	7	3	9	10	7	7	2	11	1	1	12	7	6	3	4
C	5	1	4	0	1	2	2	0	3	6	1	8	0	1	3	1	3	1	2	1	0	1	2	3	3	4	3	2	0	1
D	5	3	5	1	3	3	5	1	4	5	6	3	3	5	3	4	3	6	3	4	5	3	6	1	1	6	4	1	8	7
E	11	3	7	6	8	4	8	6	9	7	10	4	8	7	4	4	4	5	4	5	7	5	3	5	6	6	12	10	4	4
F	1	1	4	1	4	3	1	4	6	4	3	2	5	5	4	2	2	4	2	5	1	5	3	2	1	2	3	1	3	5
G	7	8	3	14	7	5	6	4	11	7	9	8	6	5	6	6	8	5	9	3	7	6	10	5	5	9	8	6	6	11
H	1	1	2	5	1	5	3	4	1	0	1	1	0	0	4	5	5	4	6	7	3	1	6	4	0	2	2	0	1	1
I	4	5	6	5	10	6	6	4	6	6	8	4	4	10	2	10	3	7	2	7	6	2	4	7	5	19	9	5	6	3
K	1	3	4	6	5	7	9	8	7	10	6	9	3	3	7	9	9	3	2	2	8	9	7	8	3	9	8	4	4	3
L	13	10	12	5	5	5	8	5	9	6	10	14	9	4	10	7	8	10	14	8	8	14	12	9	9	11	2	9	9	7
M	0	4	2	1	3	3	0	1	5	5	1	1	0	2	3	2	2	4	1	2	1	1	3	0	1	0	3	1	2	0
N	8	7	2	0	5	4	2	2	8	5	5	3	6	2	2	3	5	6	3	3	6	4	1	3	4	2	4	2	5	5
P	6	4	12	5	4	6	1	4	3	10	10	5	3	5	6	8	3	8	7	9	3	7	4	4	5	9	5	6	3	8
Q	3	7	7	7	2	3	5	3	2	3	4	3	2	4	3	9	1	10	3	4	8	2	2	21	3	4	6	1	2	4
R	5	7	7	5	2	7	5	4	5	6	3	2	8	2	6	2	6	6	13	6	6	7	6	3	6	5	6	7	7	7
S	6	4	13	9	6	11	8	3	1	6	6	9	9	5	5	8	6	11	9	11	9	7	3	5	6	4	5	8	8	9
T	6	1	2	3	2	3	2	2	1	3	4	4	2	4	4	6	3	8	3	2	5	6	4	4	5	9	9	8	1	1
V	11	6	5	3	5	12	6	9	9	7	6	8	6	10	5	8	4	9	3	7	9	9	6	3	6	13	11	7	3	6
W	0	1	0	2	0	0	2	2	0	2	0	3	2	2	1	3	1	0	1	4	2	1	0	2	2	1	2	0	0	1
Y	2	2	3	1	3	5	1	4	5	4	3	10	7	1	0	12	7	19	9	19	16	15	9	5	6	5	9	3	2	4
total	105	85	106	83	82	102	91	79	100	114	104	105	93	84	82	116	86	135	106	116	117	107	102	95	78	132	118	87	77	91

	C20	C19	C18	C17	C16	C15	C14	C13	C12	C11	C10	C9	C8	C7	C6	C5	C4	C3	C2	C1	C0	C-index	SD	*P-value*	mark	
A	6	3	5	4	2	9	5	7	9	1	5	4	6	3	6	6	4	8	7	2	11	0.551	0.265	–	–	A
C	1	1	0	1	1	2	5	4	4	2	0	1	4	1	1	2	4	1	1	3	9	0.229	0.192	6.56E-08	*	C
D	5	1	4	5	4	2	2	1	3	4	7	4	2	1	4	7	1	5	2	1	7	0.520	0.272	–	–	D
E	3	7	1	5	4	3	4	6	2	4	3	5	7	3	2	3	4	4	7	6	37	0.145	0.065	5.55E-08	*	E
F	5	7	1	2	1	4	9	3	8	7	6	1	7	8	2	3	2	3	1	6	5	0.700	0.436	–	–	F
G	3	8	8	6	7	5	8	6	14	5	6	7	5	6	8	5	8	7	5	5	17	0.402	0.138	1.67E-02	n.s.	G
H	1	2	1	2	1	4	2	4	0	2	1	2	2	2	2	2	4	1	3	2	6	0.387	0.298	3.33E-02	n.s.	H
I	0	4	3	7	5	1	5	6	9	9	6	4	5	2	6	4	3	2	4	4	7	0.771	0.437	–	–	I
K	7	10	2	4	1	4	3	11	2	2	4	6	4	4	4	5	6	3	12	3	5	1.092	0.573	–	–	K
L	10	4	8	12	7	6	8	7	9	7	6	15	12	8	5	4	10	16	9	8	8	1.083	0.389	–	–	L
M	1	1	1	1	0	3	3	0	2	2	2	1	1	0	0	1	2	2	4	5	4	0.430	0.354	8.62E-01	n.s.	M
N	3	1	7	9	2	5	2	1	2	5	2	2	4	1	1	6	2	2	5	6	6	0.617	0.355	–	–	N
P	10	2	4	1	3	10	4	3	5	6	6	13	7	6	9	12	3	2	8	5	8	0.730	0.365	–	–	P
Q	6	4	6	7	4	3	4	2	3	10	3	2	2	8	5	1	2	0	7	4	11	0.402	0.308	9.63E-03	n.s.	Q
R	4	5	2	3	7	5	0	5	3	2	3	5	7	3	7	8	6	4	5	5	7	0.731	0.314	–	–	R
S	6	11	5	5	9	10	5	8	5	6	9	5	7	8	9	1	11	5	6	8	19	0.367	0.138	1.14E-02	n.s.	S
T	4	2	11	6	5	3	4	8	5	2	8	3	5	8	7	3	4	3	8	6	18	0.247	0.137	5.61E-08	*	T
V	1	3	3	10	9	5	8	8	15	6	3	4	4	1	6	9	6	3	5	7	17	0.386	0.179	6.77E-02	n.s.	V
W	4	2	1	3	1	3	0	0	2	4	2	2	0	0	3	1	2	1	3	0	2	0.710	0.607	–	–	W
Y	4	4	4	4	3	12	11	16	7	2	3	5	4	5	4	0	3	2	3	3	6	0.967	0.793	–	–	Y
total	84	82	77	97	76	99	92	106	109	88	85	91	95	78	91	83	87	74	105	89	210	0.454	0.069			total

position -1

	C50	C49	C48	C47	C46	C45	C44	C43	C42	C41	C40	C39	C38	C37	C36	C35	C34	C33	C32	C31	C30	C29	C28	C27	C26	C25	C24	C23	C22	C21
A	6	4	14	11	4	10	7	6	4	13	9	7	4	7	11	7	5	9	13	10	11	2	4	23	5	6	9	5	2	5
C	1	0	7	2	0	1	2	1	1	3	1	3	3	5	2	2	3	1	2	1	3	1	2	3	3	2	1	3	4	2
D	3	8	5	4	8	2	4	4	4	6	2	4	5	2	1	5	5	3	4	8	7	3	5	3	1	5	4	6	1	1
E	4	1	6	6	3	9	5	4	10	6	7	5	6	4	7	5	3	15	2	5	9	4	6	2	3	9	10	5	4	4
F	0	3	3	5	3	5	2	2	4	6	2	6	4	9	1	3	2	6	1	1	3	4	5	3	2	5	2	2	2	3
G	6	3	6	7	9	5	4	4	5	3	11	13	6	7	7	8	4	5	8	13	9	8	7	5	5	10	7	12	6	4
H	6	5	3	3	1	1	4	2	0	1	3	3	5	0	2	4	0	3	6	4	3	2	2	2	5	8	3	2	4	2
I	2	3	7	4	3	1	4	6	4	5	7	4	5	2	4	8	2	4	6	9	5	4	1	3	4	2	4	3	2	4
K	10	9	1	1	6	1	7	3	8	10	3	10	3	4	8	2	7	4	6	2	3	8	10	3	3	4	12	7	7	5
L	11	12	8	10	7	11	6	3	4	11	8	6	4	9	3	15	11	11	10	7	4	13	7	5	8	6	11	4	13	9
M	1	1	3	2	0	5	0	1	1	3	4	2	1	3	1	1	1	3	0	1	4	2	4	3	0	3	1	2	0	2
N	0	4	1	2	4	2	2	2	0	2	5	3	1	0	1	2	5	4	5	8	6	5	4	3	3	5	1	2	4	3
P	15	5	4	1	5	13	5	7	13	6	7	7	6	3	4	8	5	7	10	7	8	10	4	4	6	13	7	4	6	4
Q	3	5	2	4	6	7	6	3	7	4	4	4	4	3	5	3	3	5	10	5	2	5	0	5	4	9	6	5	4	6
R	5	4	3	2	4	5	7	7	2	5	5	5	9	3	6	7	5	9	2	6	4	5	7	7	4	10	10	4	2	6
S	7	2	13	8	7	9	9	7	9	12	5	5	6	4	6	9	6	18	9	7	15	11	12	7	3	12	9	6	5	12
T	10	6	9	5	4	4	7	8	9	7	4	6	10	7	6	9	10	8	4	7	3	6	6	5	6	8	6	2	5	6
V	11	5	5	4	4	6	7	6	9	8	13	11	7	4	5	11	4	16	3	15	17	9	12	6	5	10	11	8	4	6
W	2	3	2	1	1	0	1	2	2	0	2	0	0	5	0	3	5	1	2	0	0	0	1	1	5	0	2	3	0	3
Y	2	2	4	1	3	5	2	1	4	3	2	1	4	3	2	4	0	3	3	0	1	5	3	2	3	5	2	2	2	4
total	105	85	106	83	82	102	91	79	100	114	104	105	93	84	82	116	86	135	106	116	117	107	102	95	78	132	118	87	77	91

	C20	C19	C18	C17	C16	C15	C14	C13	C12	C11	C10	C9	C8	C7	C6	C5	C4	C3	C2	C1	C0	C-index	SD	*P-value*	mark	
A	8	7	4	11	3	9	5	9	5	5	10	9	8	3	4	8	7	6	4	6	13	0.560	0.286	–	–	A
C	2	2	0	1	2	7	0	3	4	2	1	7	3	4	3	3	1	1	2	3	3	0.773	0.549	–	–	C
D	1	2	5	4	3	5	4	4	10	5	1	3	4	1	4	3	4	3	3	5	18	0.219	0.112	5.55E-08	*	D
E	3	6	7	7	4	4	8	1	5	7	8	8	9	5	3	3	4	5	5	5	15	0.368	0.175	2.48E-03	n.s.	E
F	2	2	0	3	2	3	3	6	6	1	2	3	3	1	1	1	3	2	6	3	4	0.760	0.463	–	–	F
G	6	8	2	4	5	5	6	5	13	12	3	7	2	5	6	7	3	4	8	1	8	0.798	0.368	–	–	G
H	2	2	6	2	3	4	1	2	2	2	2	3	3	1	2	3	1	1	5	3	7	0.397	0.242	1.50E-01	n.s.	H
I	7	2	6	5	2	1	0	6	2	6	1	3	3	4	2	3	2	2	5	5	4	0.945	0.498	–	–	I
K	6	0	1	7	3	10	7	7	5	4	6	2	8	3	4	3	9	8	7	3	4	1.350	0.749	–	–	K
L	7	7	12	9	9	10	7	3	7	9	7	9	6	4	11	5	5	5	10	7	10	0.792	0.297	–	–	L
M	5	2	0	0	1	1	1	2	3	1	4	3	2	1	2	0	1	0	1	1	3	0.573	0.457	–	–	M
N	1	1	1	2	0	1	9	2	4	3	3	2	2	2	4	2	1	1	2	3	11	0.245	0.175	5.65E-08	*	N
P	5	4	12	13	5	7	4	7	6	5	4	3	8	5	6	12	13	8	6	4	11	0.620	0.294	–	–	P
Q	4	6	2	5	6	5	2	6	4	4	4	2	2	5	3	1	4	9	1	4	5	0.872	0.404	–	–	Q
R	5	10	3	8	8	10	9	13	5	7	5	6	3	7	9	3	7	4	10	3	17	0.347	0.153	8.94E-04	n.s.	R
S	5	6	6	5	4	4	10	8	7	5	6	5	12	11	9	7	14	7	8	16	23	0.352	0.151	2.58E-04	n.s.	S
T	6	5	6	1	9	4	4	10	6	5	12	4	10	8	8	7	3	5	10	9	28	0.232	0.086	5.55E-08	*	T
V	7	6	1	3	5	7	8	3	6	2	3	8	5	5	5	11	1	2	9	5	24	0.287	0.156	1.82E-07	*	V
W	1	0	0	3	1	1	2	3	1	0	1	2	0	1	2	0	2	1	1	1	1	1.400	1.355	–	–	W
Y	1	4	3	4	1	1	2	6	8	3	2	2	2	2	3	1	2	0	2	2	1	2.580	1.566	–	–	Y
total	84	82	77	97	76	99	92	106	109	88	85	91	95	78	91	83	87	74	105	89	210	0.454	0.069			total

Additional file 5. Continued.

HUMAN x-x-V-x-I

position -4

	C50	C49	C48	C47	C46	C45	C44	C43	C42	C41	C40	C39	C38	C37	C36	C35	C34	C33	C32	C31	C30	C29	C28	C27	C26	C25	C24	C23	C22	C21	C20	C19	C18	C17	C16	C15	C14	C13	C12	C11	C10	C9	C8	C7	C6	C5	C4	C3	C2	C1	C0	C-index	SD	*P-value*	mark	
A	4	5	5	6	3	8	7	5	3	1	2	5	6	3	3	1	4	4	5	8	7	5	8	3	6	5	6	3	3	5	6	3	6	1	5	5	5	5	2	7	2	3	0	1	1	4	1	2	0	5	3	1.353	0.711	−	−	A
C	3	2	1	3	1	1	2	1	1	5	0	3	2	0	2	4	2	3	1	3	1	1	2	2	1	1	1	4	3	2	2	4	2	2	2	1	3	0	3	3	2	2	4	0	6	5	3	3	2	0	4	0.535	0.343	2.94E-07	*	C
D	3	2	5	2	4	2	8	4	3	4	3	3	4	4	1	2	5	6	3	2	2	6	6	7	8	6	4	1	1	1	2	2	3	5	0	4	3	3	3	4	3	3	3	4	1	3	1	5	4	2	2	1.700	0.909	−	−	D
E	8	4	7	4	6	5	5	5	6	3	4	4	3	5	1	1	3	6	3	6	4	3	10	4	5	5	4	2	6	1	4	1	3	6	2	3	5	9	2	6	5	4	3	3	2	7	1	5	5	8	7	0.620	0.298	1.17E-05	*	E
F	3	3	2	5	2	9	2	2	6	6	2	0	3	1	4	4	5	3	3	4	4	2	3	6	2	0	3	0	3	3	3	5	5	2	2	4	3	1	1	7	2	2	3	2	4	3	2	7	2	6	5	0.644	0.380	1.33E-04	n.s.	F
G	4	5	7	5	5	4	2	4	4	7	9	4	8	2	2	3	7	5	3	5	3	6	2	19	9	10	11	5	8	9	6	10	5	5	11	2	5	17	5	11	3	5	3	1	10	2	3	9	4	2	1	5.920	3.741	−	−	G
H	1	3	2	1	1	1	0	0	1	1	1	3	2	1	0	1	1	3	3	1	1	0	1	1	1	5	1	0	1	2	3	6	1	1	3	3	6	2	0	0	1	1	2	1	3	2	4	2	2	0	3	0.553	0.479	6.42E-05	*	H
I	3	3	2	1	3	6	4	1	3	3	4	3	2	3	3	1	4	7	1	2	6	5	4	5	3	4	4	2	6	6	4	7	6	3	4	3	4	4	4	6	4	4	7	5	2	9	8	7	4	5	2	2.040	0.936	−	−	I
K	3	5	5	5	5	8	5	1	3	3	4	4	6	7	7	3	4	4	4	6	5	8	4	6	4	4	9	2	4	6	5	3	2	4	21	5	4	1	6	8	2	3	5	6	5	2	5	2	3	6	4	1.210	0.739	−	−	K
L	4	7	10	13	5	5	9	10	4	9	6	8	5	6	10	9	4	4	11	12	6	9	6	4	8	7	6	5	7	12	4	12	8	12	9	6	9	10	7	6	6	14	4	9	5	13	10	10	5	6	5	1.544	0.566	−	−	L
M	0	2	4	3	4	1	2	1	2	3	0	1	2	0	3	1	2	3	5	1	0	0	0	3	0	1	2	1	1	2	2	1	3	1	0	0	0	0	0	0	1	0	2	2	3	0	2	2	1	0	3	0.467	0.431	3.75E-06	*	M
N	3	5	1	0	0	3	6	4	4	2	5	2	6	4	0	2	4	1	4	1	3	2	2	5	2	3	1	2	1	3	3	5	3	1	2	1	4	0	3	2	1	2	1	1	6	4	2	0	3	3	12	0.213	0.139	5.55E-08	*	N
P	6	4	3	5	2	6	11	4	4	7	8	5	6	2	8	3	2	1	1	5	3	1	4	5	5	6	3	6	5	4	5	8	3	10	6	8	2	1	4	3	3	3	1	5	6	2	2	5	1	2	4	1.075	0.598	−	−	P
Q	9	3	7	1	1	2	3	4	4	4	3	9	3	1	2	5	2	4	1	3	4	4	5	2	2	3	1	3	6	2	4	2	7	7	3	3	5	7	5	2	5	3	6	6	5	2	7	1	1	4	8	0.470	0.264	5.57E-08	*	Q
R	2	3	6	5	5	2	6	5	4	6	2	6	7	5	7	11	2	4	3	2	3	3	8	6	5	0	7	4	3	5	7	6	7	3	4	0	4	0	3	1	2	6	3	8	4	2	1	3	3	7	6	0.703	0.392	1.71E-01	n.s.	R
S	3	10	9	4	5	7	8	5	6	8	5	11	5	2	6	7	7	2	8	5	1	5	5	7	6	9	5	4	9	2	3	8	3	6	2	7	6	5	6	1	11	8	10	9	5	5	4	5	4	6	5	1.160	0.503	−	−	S
T	4	0	6	4	9	6	7	7	4	4	4	4	12	1	3	4	3	5	3	4	2	5	2	3	4	1	3	6	5	1	1	4	4	6	2	3	0	3	4	6	7	5	1	0	5	7	3	0	4	2	6	0.643	0.400	8.73E-04	n.s.	T
V	9	9	7	6	7	7	6	9	7	11	8	3	9	7	7	4	2	5	5	5	3	7	3	6	6	5	10	6	8	8	2	8	4	5	5	3	5	6	4	5	2	7	1	6	2	8	3	4	4	6	4	1.425	0.579	−	−	V
W	0	1	1	0	1	2	0	0	1	0	0	0	0	1	2	1	1	1	2	0	0	0	1	2	0	2	0	0	0	1	2	3	0	1	1	1	4	0	0	1	0	1	0	0	1	3	0	2	1	2	1	0.860	0.969	−	−	W
Y	2	1	3	2	1	2	7	0	6	2	2	3	2	2	3	6	9	3	6	4	3	8	5	2	1	0	3	0	2	3	2	0	2	2	0	1	3	4	0	1	2	3	2	2	1	1	2	1	2	1	4	0.625	0.511	8.66E-06	*	Y
total	74	77	93	75	70	87	100	72	76	89	72	81	93	57	74	73	73	74	75	79	61	80	81	98	78	77	84	56	82	78	70	98	77	83	84	63	80	78	62	80	64	79	61	71	77	84	64	75	55	73	89	0.858	0.117			total

position -3

	C50	C49	C48	C47	C46	C45	C44	C43	C42	C41	C40	C39	C38	C37	C36	C35	C34	C33	C32	C31	C30	C29	C28	C27	C26	C25	C24	C23	C22	C21	C20	C19	C18	C17	C16	C15	C14	C13	C12	C11	C10	C9	C8	C7	C6	C5	C4	C3	C2	C1	C0	C-index	SD	*P-value*	mark	
A	3	5	5	7	7	6	10	5	3	5	6	6	16	2	6	4	7	5	2	4	2	4	3	6	5	4	10	7	6	6	8	5	2	10	7	4	4	3	3	6	8	6	3	5	5	3	5	3	1	3	5	1.044	0.523	−	−	A
C	0	3	6	5	3	5	1	1	3	3	0	3	1	1	2	7	3	3	6	4	1	3	1	1	2	3	2	1	1	1	0	1	1	1	1	3	1	3	2	1	5	5	2	3	7	4	0	1	2	1	0	−	−	−	−	C
D	2	3	3	1	2	8	5	2	8	3	3	5	3	3	6	3	1	5	2	4	3	7	3	5	3	1	3	4	6	5	4	3	4	3	0	2	1	4	2	3	8	3	3	2	4	9	2	5	5	0	6	0.597	0.340	3.64E-06	*	D
E	5	6	5	2	2	6	6	5	3	4	5	6	3	6	4	1	2	1	6	7	6	1	6	5	6	7	3	2	7	6	5	4	5	5	5	2	2	5	5	9	5	3	3	3	2	3	0	7	3	6	5	0.864	0.395	−	−	E
F	1	1	0	5	2	4	5	1	1	3	5	2	1	0	1	4	4	3	4	2	3	3	2	1	1	0	3	0	1	3	0	4	6	2	2	5	3	1	3	5	1	2	3	2	2	5	2	2	7	1	2	1.240	0.859	−	−	F
G	5	4	11	5	2	3	6	6	5	6	5	3	5	1	3	2	3	9	4	3	2	5	3	11	6	4	3	4	4	0	4	8	8	3	5	2	3	4	3	5	6	2	4	4	5	5	7	4	4	4	7	0.637	0.315	2.34E-06	*	G
H	1	4	3	3	5	0	3	2	2	2	1	0	3	0	2	3	0	4	3	2	1	2	1	3	0	4	1	0	1	0	2	2	0	0	0	1	3	3	4	1	3	2	2	3	2	0	1	1	0	0	2	0.860	0.693	−	−	H
I	5	2	3	2	2	8	5	2	1	4	2	3	7	2	3	2	4	4	10	3	4	3	2	2	4	8	6	5	4	4	2	4	2	3	6	2	4	6	3	10	2	5	0	7	4	6	3	1	2	6	3	1.293	0.743	−	−	I
K	8	5	10	5	3	2	3	1	6	5	5	9	0	4	6	4	2	4	4	4	3	4	6	10	5	6	3	3	1	6	7	10	5	3	2	4	4	2	4	3	4	2	1	8	7	1	7	5	1	5	3	1.480	0.822	−	−	K
L	3	11	9	4	4	6	12	9	5	14	5	10	5	8	2	6	9	8	6	6	9	5	10	8	5	14	7	7	5	9	6	12	12	11	7	9	9	5	4	6	4	12	11	3	5	12	10	3	5	6	3	2.487	1.033	−	−	L
M	1	0	3	2	4	1	3	1	2	3	1	1	3	0	1	1	0	1	1	3	0	1	3	2	4	3	2	2	4	2	1	1	2	2	2	1	3	1	2	1	0	2	2	1	1	2	3	5	2	5	1	1.880	1.239	−	−	M
N	1	2	4	7	2	2	1	2	3	4	1	2	2	1	3	3	7	3	3	2	1	4	1	3	1	3	4	2	3	3	2	1	2	0	2	5	4	6	3	2	2	2	0	3	1	3	1	3	3	5	6	0.433	0.259	5.55E-08	*	N
P	3	7	4	4	5	6	6	7	9	2	6	4	4	2	10	7	2	2	4	2	0	3	4	7	2	4	1	1	4	3	5	5	2	4	6	3	4	4	4	4	5	4	3	3	7	5	2	6	4	5	5	0.840	0.406	8.33E-01	n.s.	P
Q	2	4	2	6	4	8	3	4	1	3	2	1	6	3	2	5	0	2	2	3	0	5	6	4	7	1	2	5	6	7	5	11	7	5	2	3	2	2	3	5	2	4	2	5	5	2	1	12	2	4	2	1.900	1.254	−	−	Q
R	7	5	5	1	3	3	2	3	4	5	3	4	5	1	8	2	5	5	3	6	2	3	5	10	10	3	3	3	1	1	5	5	1	5	21	1	12	4	2	2	2	5	7	6	3	3	4	1	3	3	4	1.080	0.855	−	−	R
S	11	4	4	5	6	8	8	10	7	8	8	5	7	9	6	5	13	4	3	7	5	7	6	5	6	2	9	3	9	7	4	10	7	8	7	6	12	10	3	3	5	7	4	2	6	2	7	6	3	9	16	0.398	0.164	5.55E-08	*	S
T	2	3	9	4	3	5	5	7	4	8	3	3	6	3	2	3	5	5	7	9	11	11	12	4	3	3	7	0	6	5	5	2	3	9	5	4	5	5	3	7	1	4	5	5	2	6	3	5	5	4	6	0.820	0.428	3.86E-01	n.s.	T
V	9	2	5	4	7	3	11	3	6	4	7	7	7	8	3	5	4	4	3	6	5	4	4	7	3	5	9	4	7	5	4	8	5	5	3	2	2	7	4	5	0	7	3	1	6	10	4	2	1	4	8	0.610	0.297	2.31E-05	*	V
W	4	1	0	1	2	1	2	1	1	0	0	3	0	0	2	3	1	1	1	0	0	1	0	2	4	0	2	0	4	0	1	0	2	2	1	0	0	3	2	0	0	0	0	1	1	1	1	0	2	0	1	1.080	1.175	−	−	W
Y	1	5	2	2	2	2	3	0	2	3	4	4	9	3	2	3	1	1	1	2	3	4	3	2	1	2	4	3	2	5	0	2	1	2	0	4	2	0	3	2	1	2	3	4	2	2	1	3	0	2	4	0.585	0.396	5.07E-06	*	Y
total	74	77	93	75	70	87	100	72	76	89	72	81	93	57	74	73	73	74	75	79	61	80	81	98	78	77	84	56	82	78	70	98	77	83	84	63	80	78	62	80	64	79	61	71	77	84	64	75	55	73	89	0.858	0.117			total

position -1

	C50	C49	C48	C47	C46	C45	C44	C43	C42	C41	C40	C39	C38	C37	C36	C35	C34	C33	C32	C31	C30	C29	C28	C27	C26	C25	C24	C23	C22	C21	C20	C19	C18	C17	C16	C15	C14	C13	C12	C11	C10	C9	C8	C7	C6	C5	C4	C3	C2	C1	C0	C-index	SD	*P-value*	mark	
A	6	4	10	6	7	1	8	11	6	6	3	8	9	2	5	3	7	6	3	6	11	8	3	5	6	5	6	2	5	3	5	6	6	8	5	8	4	4	6	7	2	5	2	3	6	4	5	1	5	0	3	1.753	0.824	−	−	A
C	4	2	1	5	5	6	3	3	0	2	0	0	1	1	1	0	3	1	2	1	2	3	1	3	2	1	0	0	1	2	1	0	1	4	2	0	1	2	1	0	3	3	2	2	2	0	2	1	2	0	3	0.567	0.482	1.33E-04	n.s.	C
D	3	3	7	1	0	6	8	0	1	7	4	4	4	3	4	3	2	3	0	0	1	4	7	3	2	2	5	4	4	3	7	1	4	5	2	5	5	2	1	6	7	5	4	1	5	5	2	1	2	3	4	0.855	0.532	1.00E+00	n.s.	D
E	6	3	5	5	4	0	3	8	2	4	3	5	5	0	2	5	2	4	3	4	3	2	4	6	4	2	5	3	2	3	5	1	4	7	2	2	3	5	3	3	2	4	1	4	3	4	2	9	3	2	5	0.704	0.362	4.92E-03	n.s.	E
F	2	3	3	1	3	3	3	5	5	6	0	0	4	4	2	3	3	3	5	6	2	5	2	2	6	3	6	1	7	1	8	4	1	1	8	1	5	1	3	3	0	3	4	2	2	9	3	3	3	3	5	0.664	0.423	1.11E-03	n.s.	F
G	8	3	10	5	3	2	5	4	9	7	5	10	5	2	4	3	6	15	13	11	4	3	6	5	7	8	6	7	6	8	4	8	6	5	5	7	4	6	0	8	7	10	4	5	3	4	0	4	4	5	1	5.780	2.957	−	−	G
H	3	1	4	3	4	3	6	1	2	6	6	4	6	3	0	3	2	0	2	1	1	4	2	4	1	2	0	3	2	3	2	1	2	2	2	1	1	2	4	1	1	4	3	1	0	2	4	2	1	3	2	1.210	0.796	−	−	H
I	3	4	8	3	3	5	7	5	2	3	5	2	2	3	3	3	4	4	6	6	4	4	5	8	4	1	6	2	2	13	2	3	7	2	2	4	4	5	1	6	6	4	4	2	6	3	8	3	5	2	3	1.393	0.743	−	−	I
K	4	4	4	5	2	3	5	5	6	1	2	5	3	7	6	7	2	1	3	3	4	5	7	3	12	5	4	1	6	8	1	14	6	10	9	5	7	3	6	3	5	2	2	4	7	6	3	8	4	7	10	0.490	0.273	5.55E-08	*	K
L	5	9	11	7	7	7	4	6	10	10	5	6	8	4	16	10	8	8	8	4	6	4	8	9	7	7	7	8	7	6	8	16	6	6	5	8	7	6	4	3	6	9	8	9	4	10	3	12	4	8	4	1.820	0.693	−	−	L
M	0	1	2	1	0	0	5	1	2	1	0	2	0	0	2	1	2	0	1	0	1	2	0	4	2	0	2	0	1	1	1	0	2	1	0	1	0	2	1	0	2	0	2	0	1	2	0	1	2	2	2	0.540	0.542	1.28E-02	n.s.	M
N	6	1	3	0	2	5	7	3	1	4	3	2	4	1	2	5	5	4	5	3	1	1	2	3	5	2	1	2	3	6	1	5	3	6	1	0	2	3	2	2	4	4	2	6	4	1	1	1	3	3	5	0.584	0.354	9.46E-05	*	N
P	2	3	4	3	4	8	3	2	3	6	6	3	4	3	7	5	2	2	3	6	3	3	6	3	5	5	4	1	9	4	3	6	6	0	3	3	1	3	8	3	2	4	1	5	9	2	2	4	3	2	5	0.768	0.413	3.51E-02	n.s.	P
Q	3	5	5	0	5	3	4	2	3	0	3	4	3	5	4	4	2	7	2	5	3	1	5	2	0	4	5	0	3	3	1	3	4	2	2	0	5	1	1	7	0	3	1	1	3	4	4	4	1	4	3	0.973	0.605	−	−	Q
R	2	3	3	2	8	3	3	2	4	3	5	0	9	2	2	4	2	3	6	3	5	5	3	5	1	0	8	5	7	1	3	5	1	3	2	3	6	3	3	2	3	1	5	4	7	5	3	6	5	6	5	0.740	0.415	1.38E-01	n.s.	R
S	4	7	1	5	2	8	10	2	6	4	8	5	5	8	5	2	8	4	2	3	4	5	12	4	4	7	4	7	6	4	3	10	2	11	3	7	8	16	1	11	1	5	4	7	7	5	9	1	2	11	9	0.622	0.367	2.39E-04	n.s.	S
T	3	7	5	3	4	5	6	4	4	9	6	3	6	3	3	2	0	4	3	10	3	4	4	20	5	15	8	3	5	4	4	2	5	6	20	3	5	3	6	6	6	5	2	11	2	4	3	3	2	4	8	0.658	0.496	5.26E-07	*	T
V	5	6	3	8	6	8	6	4	8	6	4	12	11	3	5	4	2	3	5	3	3	14	3	5	2	5	5	6	5	3	7	8	5	3	2	2	8	5	7	5	5	4	3	0	5	9	4	5	2	4	5	1.024	0.535	−	−	V
W	1	1	1	5	0	5	1	1	2	1	2	5	1	0	0	4	9	2	1	1	0	1	1	0	1	2	1	0	1	0	0	3	1	0	2	0	2	2	1	1	1	1	4	0	0	1	2	0	0	2	2	0.730	0.864	8.62E-03	n.s.	W
Y	4	7	3	7	1	6	3	3	0	3	2	1	3	3	1	2	2	0	2	3	0	2	0	4	2	1	1	1	0	2	4	2	5	1	7	3	2	4	3	3	1	3	3	4	1	4	4	6	2	2	5	0.532	0.365	8.73E-08	*	Y
total	74	77	93	75	70	87	100	72	76	89	72	81	93	57	74	73	73	74	75	79	61	80	81	98	78	77	84	56	82	78	70	98	77	83	84	63	80	78	62	80	64	79	61	71	77	84	64	75	55	73	89	0.858	0.117			total

Additional file 5. Continued.

HUMAN x-x-V-x-V

position -4

	C50	C49	C48	C47	C46	C45	C44	C43	C42	C41	C40	C39	C38	C37	C36	C35	C34	C33	C32	C31	C30	C29	C28	C27	C26	C25	C24	C23	C22	C21	C20	C19	C18	C17	C16	C15	C14	C13	C12	C11	C10	C9	C8	C7	C6	C5	C4	C3	C2	C1	C0	C-index	SD	*P-value*	mark	
A	9	5	8	9	14	11	13	7	6	6	14	4	9	4	4	10	12	8	9	9	9	4	11	14	13	6	6	9	13	7	8	6	9	4	7	8	5	7	1	7	8	14	8	10	6	8	12	11	6	4	8	1.030	0.394	–	–	A
C	5	6	4	2	3	9	2	5	3	3	3	3	6	2	3	3	6	3	5	1	1	4	4	5	5	2	5	3	2	3	5	2	3	2	0	2	1	2	2	4	1	2	2	1	7	4	6	2	2	0	7	0.460	0.267	5.55E-08	*	C
D	7	14	6	4	5	12	8	3	10	14	3	7	2	6	5	8	6	7	5	8	14	2	9	8	9	6	7	8	3	2	11	2	7	4	7	6	3	1	7	7	4	5	2	4	2	3	2	5	5	4	4	1.495	0.817	–	–	D
E	11	9	11	7	6	5	9	5	7	10	5	10	13	8	10	6	7	8	4	8	7	9	5	5	5	14	4	12	9	6	7	10	8	5	6	5	7	7	5	3	9	6	10	3	5	5	8	4	12	3	7	1.037	0.386	–	–	E
F	2	7	3	5	3	5	6	7	8	3	6	7	2	6	6	6	7	7	8	8	1	3	3	3	3	4	6	5	5	0	7	6	6	7	5	6	1	1	7	2	4	1	6	2	2	3	2	6	4	8	1	4.620	2.258	–	–	F
G	7	5	6	12	7	6	3	21	11	21	7	8	8	12	6	12	9	7	11	7	4	6	7	7	9	6	9	6	9	10	2	6	12	6	6	10	8	6	8	7	5	7	5	10	10	8	7	6	3	8	8	0.998	0.446	–	–	G
H	2	5	7	5	1	5	2	7	4	3	4	3	5	2	3	3	1	3	4	1	1	2	1	5	3	4	2	1	7	6	2	3	2	1	3	2	4	1	4	7	4	1	1	2	4	5	3	2	9	1	3	1.087	0.659	–	–	H
I	5	7	6	2	13	4	6	3	3	9	9	5	7	2	5	5	6	8	5	8	8	3	7	7	6	9	4	4	8	2	10	7	7	2	6	5	4	5	3	5	8	7	7	5	6	7	2	5	5	4	4	1.430	0.580	–	–	I
K	10	7	4	4	9	7	8	4	6	6	14	8	28	6	15	4	7	5	7	5	5	4	7	8	5	10	5	6	5	3	6	3	7	8	7	6	9	4	7	9	6	11	3	9	8	9	6	4	8	4	8	0.890	0.494	–	–	K
L	18	16	12	11	16	8	15	8	13	11	19	14	15	9	13	14	18	10	16	16	14	13	9	6	15	6	11	12	12	12	8	14	16	10	13	9	8	5	9	8	14	7	10	10	15	4	12	9	12	16	11	1.075	0.326	–	–	L
M	4	0	2	5	1	3	3	0	2	2	3	2	1	3	1	4	2	3	1	0	4	1	0	5	0	3	1	1	2	0	1	1	2	1	3	8	3	6	3	7	3	1	1	1	4	2	2	2	6	0	3	0.773	0.626	1.00E-02	n.s.	M
N	4	4	2	2	5	6	4	2	3	4	8	3	7	6	1	4	2	6	2	3	2	5	3	5	4	3	6	5	2	0	6	4	8	5	4	7	3	4	5	3	0	2	7	3	6	2	8	6	4	0	5	0.800	0.414	4.09E-02	n.s.	N
P	10	9	6	10	10	4	8	9	3	9	11	6	4	8	10	8	5	3	7	5	8	6	11	8	12	6	8	25	8	4	11	3	7	2	6	5	1	5	5	5	9	23	4	4	5	8	8	8	6	3	3	2.460	1.438	–	–	P
Q	4	6	5	3	2	6	7	3	2	2	8	9	8	4	12	7	4	3	11	4	4	5	5	6	5	4	6	3	4	4	1	4	3	5	4	3	5	5	4	4	4	5	3	7	4	6	7	3	4	2	11	0.435	0.200	5.55E-08	*	Q
R	5	2	10	4	6	5	11	6	11	2	6	6	6	9	1	6	6	8	2	9	4	3	8	2	8	2	7	7	3	4	8	3	8	3	7	3	15	3	9	3	2	3	2	2	6	2	7	5	6	7	5	1.092	0.600	–	–	R
S	13	13	10	6	14	3	5	10	12	6	6	11	5	6	12	8	15	8	8	12	11	6	11	20	7	13	4	7	7	16	7	9	10	1	14	6	6	12	5	12	5	3	13	8	7	13	8	6	9	7	6	1.487	0.640	–	–	S
T	5	6	9	7	6	5	3	3	5	6	7	6	3	13	7	5	7	8	5	5	11	5	11	8	10	5	3	10	5	5	5	6	2	7	3	5	3	5	5	5	3	5	2	6	5	4	7	3	4	5	7	0.811	0.341	1.74E-04	n.s.	T
V	7	5	12	12	8	10	16	9	10	10	6	12	14	12	11	10	9	9	11	7	10	9	7	9	7	7	10	6	4	11	13	7	9	9	10	5	13	7	7	3	10	5	7	9	1	6	9	7	8	5	11	0.782	0.264	4.74E-05	*	V
W	0	0	0	1	1	1	1	0	1	1	2	0	2	2	3	1	3	0	2	2	0	4	4	4	0	1	0	0	1	1	1	5	1	2	0	2	0	1	0	0	1	0	1	0	0	0	0	1	1	1	4	0.275	0.312	5.55E-08	*	W
Y	3	2	2	5	1	2	2	3	2	2	5	5	4	4	7	3	6	5	5	4	5	6	3	5	7	5	5	2	1	5	7	2	4	1	4	6	4	4	3	2	0	1	4	2	0	1	2	3	0	4	2	1.700	0.942	–	–	Y
total	131	128	125	116	131	117	132	115	122	130	146	129	149	124	135	127	138	119	128	122	123	100	126	140	133	116	109	132	110	101	126	103	131	85	115	109	103	91	99	103	100	109	98	98	103	100	118	98	114	86	118	0.990	0.131			total

position -3

	C50	C49	C48	C47	C46	C45	C44	C43	C42	C41	C40	C39	C38	C37	C36	C35	C34	C33	C32	C31	C30	C29	C28	C27	C26	C25	C24	C23	C22	C21	C20	C19	C18	C17	C16	C15	C14	C13	C12	C11	C10	C9	C8	C7	C6	C5	C4	C3	C2	C1	C0	C-index	SD	*P-value*	mark	
A	16	10	13	14	9	7	16	15	7	10	15	13	15	9	11	4	10	9	5	8	7	6	15	12	16	13	8	11	8	6	10	9	7	4	12	7	4	9	7	12	5	6	8	14	7	11	9	5	6	4	8	1.185	0.451	–	–	A
C	4	0	4	3	8	6	4	1	1	4	2	1	6	2	4	8	5	5	4	3	1	3	1	1	5	4	6	18	1	3	2	3	3	2	5	2	5	1	6	2	4	2	3	3	11	3	2	4	2	3	4	0.930	0.739	1.57E-01	n.s.	C
D	6	5	5	7	8	4	4	5	8	7	6	4	4	8	5	7	4	7	5	3	3	8	6	4	3	4	7	5	2	3	7	4	6	5	9	8	3	3	7	6	4	6	4	2	3	2	4	5	7	1	5	1.012	0.384	–	–	D
E	12	12	6	7	12	4	7	5	12	6	11	6	6	6	11	10	5	2	4	8	6	6	8	8	7	3	3	3	13	3	11	2	7	8	5	8	6	13	6	3	6	2	3	3	6	7	12	5	6	6	13	0.518	0.243	5.55E-08	*	E
F	6	5	5	3	5	5	4	1	1	3	6	2	2	3	10	3	1	5	6	4	4	1	5	17	5	5	5	3	2	4	6	4	9	3	5	6	3	1	3	2	1	3	9	3	3	4	8	3	5	6	3	1.453	0.930	–	–	F
G	9	10	8	9	8	8	14	9	5	15	11	12	17	10	22	6	9	7	16	6	4	9	9	6	15	13	7	5	7	18	8	6	9	3	14	7	8	7	9	3	3	10	7	14	2	11	4	4	10	5	10	0.896	0.427	9.07E-02	n.s.	G
H	2	0	2	0	2	1	2	5	1	2	7	3	5	6	9	3	3	4	3	4	2	0	1	4	3	5	6	5	2	0	4	2	4	4	0	2	0	0	4	7	4	1	1	2	1	3	4	3	6	4	2	1.480	1.054	–	–	H
I	6	4	2	4	4	7	7	5	6	5	5	7	4	5	2	3	8	7	7	5	13	4	4	8	3	1	4	8	6	4	2	4	5	4	10	2	3	2	2	7	2	2	5	6	3	2	3	3	5	0	4	1.150	0.610	–	–	I
K	6	4	5	5	6	2	8	6	6	3	2	9	8	8	3	11	17	2	2	8	7	6	6	5	9	15	3	4	2	4	2	5	8	3	7	6	10	3	4	7	5	1	4	9	8	8	4	2	7	6	11	0.529	0.294	5.55E-08	*	K
L	9	23	16	11	12	10	12	8	10	12	14	16	15	13	7	10	14	16	11	15	8	5	7	12	10	6	5	9	11	11	15	9	14	5	16	9	9	12	7	5	4	8	8	5	10	6	9	7	9	9	9	1.142	0.425	–	–	L
M	7	4	2	2	2	4	1	2	1	4	5	2	3	1	5	4	4	3	2	1	2	0	1	1	3	2	3	3	0	2	2	2	3	1	1	3	2	1	2	1	2	2	4	2	3	2	0	2	1	2	2	1.140	0.693	–	–	M
N	2	5	4	2	5	7	5	0	5	2	6	2	9	3	4	7	4	5	4	8	5	6	3	4	1	2	8	2	4	3	10	7	6	4	2	4	8	7	0	0	1	0	0	2	3	1	7	2	2	3	2	1.960	1.285	–	–	N
P	4	8	7	7	7	9	12	10	14	10	4	9	8	8	5	9	3	10	8	6	13	7	9	13	8	6	10	12	10	5	6	11	12	5	4	7	5	3	7	4	11	6	4	6	7	4	5	8	6	7	9	0.842	0.314	4.37E-02	n.s.	P
Q	11	8	3	7	8	3	11	6	5	3	5	5	7	6	8	7	6	5	6	11	7	2	11	8	6	4	2	3	5	4	7	3	7	5	9	6	6	0	4	5	5	4	5	2	4	4	7	4	5	2	4	1.385	0.623	–	–	Q
R	5	5	10	4	12	2	4	6	6	14	12	4	5	6	3	4	9	10	14	6	5	7	6	1	5	8	6	9	11	6	5	8	3	2	5	4	6	4	8	7	7	30	8	3	4	7	9	11	7	6	4	1.745	1.116	–	–	R
S	12	8	8	5	8	13	4	12	16	6	11	12	4	4	4	8	10	8	13	4	10	10	10	6	13	11	9	12	7	7	14	6	12	13	1	8	7	3	5	10	10	8	5	11	7	8	8	5	8	6	12	0.700	0.275	6.23E-07	*	S
T	5	8	6	12	6	8	7	5	11	8	5	10	5	7	4	6	6	4	4	6	5	8	7	16	7	6	5	7	6	5	5	9	6	6	4	9	5	9	5	7	10	7	7	5	7	6	9	9	5	5	4	1.700	0.576	–	–	T
V	3	7	10	11	6	9	8	8	5	10	12	8	22	13	16	9	17	5	7	9	10	7	6	4	8	4	7	11	10	11	7	5	8	5	4	5	7	5	7	11	10	8	8	4	7	6	7	11	11	7	6	1.387	0.592	–	–	V
W	4	1	3	3	0	4	2	2	0	4	5	0	1	3	1	4	2	2	1	2	3	1	2	3	1	2	1	1	2	0	0	1	1	1	1	3	0	3	3	4	2	0	5	0	2	3	6	4	3	1	1	2.060	1.517	–	–	W
Y	2	1	6	0	3	4	0	4	2	2	2	4	3	3	1	4	1	3	6	5	8	4	9	7	5	2	4	1	1	2	3	3	1	2	1	3	6	5	3	0	4	3	0	2	5	2	1	1	3	3	5	0.600	0.412	2.23E-07	*	Y
total	131	128	125	116	131	117	132	115	122	130	146	129	149	124	135	127	138	119	128	122	123	100	126	140	133	116	109	132	110	101	126	103	131	85	115	109	103	91	99	103	100	109	98	98	103	100	118	98	114	86	118	0.990	0.131			total

position -1

	C50	C49	C48	C47	C46	C45	C44	C43	C42	C41	C40	C39	C38	C37	C36	C35	C34	C33	C32	C31	C30	C29	C28	C27	C26	C25	C24	C23	C22	C21	C20	C19	C18	C17	C16	C15	C14	C13	C12	C11	C10	C9	C8	C7	C6	C5	C4	C3	C2	C1	C0	C-index	SD	*P-value*	mark	
A	10	9	12	8	5	12	11	6	7	8	18	12	18	6	10	12	13	7	12	8	8	13	9	6	12	12	8	12	13	10	4	3	13	8	11	10	4	5	7	16	6	7	10	9	5	13	9	5	11	6	4	2.345	0.867	–	–	A
C	8	4	7	3	8	1	2	1	5	3	6	2	5	5	3	3	2	5	3	6	2	1	6	4	4	4	1	2	4	2	2	3	8	1	3	1	3	1	6	4	3	7	4	1	3	5	5	7	9	2	4	0.950	0.546	8.13E-01	n.s.	C
D	3	4	10	8	9	2	2	4	2	0	7	5	7	9	14	9	4	2	6	12	3	4	4	16	9	3	5	4	4	1	4	3	7	3	8	6	6	3	1	2	4	5	2	2	8	4	2	0	3	8	9	0.562	0.383	7.14E-08	*	D
E	5	5	6	5	8	5	5	7	2	12	3	8	6	9	6	9	8	8	8	3	11	4	3	7	6	6	3	8	10	8	11	4	5	3	3	8	8	6	10	2	4	3	11	6	7	9	8	7	4	3	4	1.580	0.654	–	–	E
F	6	7	6	7	2	8	5	6	11	2	0	4	7	2	5	5	5	8	8	4	7	3	4	6	5	2	3	4	3	1	12	5	5	2	3	2	4	4	2	1	11	2	0	2	1	1	11	3	4	3	6	0.747	0.486	1.34E-03	n.s.	F
G	9	9	3	8	6	4	6	4	7	16	11	5	9	7	10	10	10	4	12	5	1	7	12	12	6	12	3	5	5	6	3	4	7	4	6	9	8	4	2	7	5	3	7	4	2	5	4	5	3	2	2	3.180	1.625	–	–	G
H	5	4	4	1	4	3	3	8	2	3	3	1	3	6	2	2	5	9	3	2	2	2	5	5	0	4	1	3	6	1	4	4	3	3	2	5	2	1	2	1	3	0	1	2	3	3	3	6	1	3	1	3.080	1.883	–	–	H
I	6	2	4	5	4	9	4	5	1	6	11	5	7	8	5	3	7	2	9	6	5	2	7	12	4	5	2	1	6	6	4	4	9	2	4	2	3	0	5	7	1	3	8	0	4	5	10	1	2	3	2	2.360	1.418	–	–	I
K	7	10	7	1	10	7	14	7	9	6	5	8	8	11	6	6	7	6	4	6	6	5	9	9	9	6	10	4	5	11	5	10	7	7	6	5	8	4	8	6	4	9	4	7	11	6	6	9	13	8	10	0.724	0.250	2.45E-07	*	K
L	11	13	13	10	14	14	11	8	10	17	11	22	9	7	11	13	13	10	11	14	15	16	12	10	10	12	16	15	12	11	10	13	9	8	11	7	8	12	9	5	7	7	3	5	11	10	8	11	10	13	4	2.740	0.838	–	–	L
M	2	2	2	3	5	3	4	3	4	2	2	4	3	0	6	5	6	3	1	1	8	1	4	2	0	3	2	1	2	3	8	1	1	2	3	3	1	2	4	1	10	1	4	1	1	3	3	1	1	2	0	–	–	–	–	M
N	5	0	4	1	1	5	7	5	4	5	5	3	4	2	5	4	1	2	5	8	2	4	5	5	7	2	3	4	4	1	4	6	2	5	7	5	7	4	2	3	2	3	1	3	2	5	2	3	5	0	1	3.680	1.932	–	–	N
P	9	10	8	12	11	12	15	7	11	7	13	12	12	12	7	8	7	7	7	9	12	9	5	11	12	7	8	6	4	10	11	8	10	5	7	4	4	8	3	9	12	6	4	14	23	6	13	6	8	4	12	0.742	0.303	5.12E-06	*	P
Q	5	7	3	6	6	5	10	5	3	7	8	8	3	4	4	6	7	5	4	4	4	0	8	4	6	11	3	5	1	1	9	3	9	1	4	5	8	6	2	9	2	2	7	5	0	4	2	6	5	1	11	0.442	0.241	5.55E-08	*	Q
R	5	11	8	4	4	4	5	9	6	3	7	0	5	7	6	4	9	5	5	9	5	3	6	3	9	3	11	25	6	8	4	5	6	13	4	5	6	1	4	6	5	6	6	4	3	3	4	7	5	7	7	0.854	0.528	1.91E-03	n.s.	R
S	11	10	8	8	9	8	10	8	6	9	5	10	16	11	9	10	17	10	7	10	13	11	9	13	11	5	9	9	8	6	11	12	9	9	8	8	7	11	11	9	9	31	7	9	6	6	10	7	7	4	13	0.734	0.307	5.58E-08	*	S
T	4	7	11	12	8	7	8	8	16	10	15	8	12	4	7	7	8	15	7	5	11	4	3	6	9	6	7	7	6	6	5	6	12	5	10	12	10	10	9	3	5	6	4	12	3	4	8	3	12	14	12	0.662	0.286	1.55E-07	*	T
V	10	12	5	8	11	3	4	8	11	6	10	10	13	11	10	8	3	6	13	5	5	7	7	8	5	11	8	11	7	7	9	5	6	2	7	8	3	7	9	6	4	4	7	10	6	7	7	6	1	1	5	1.432	0.594	–	–	V
W	0	2	2	5	4	1	2	1	5	2	1	1	0	1	1	1	5	4	2	0	2	2	6	0	3	1	1	0	1	0	2	1	3	0	1	2	2	1	1	4	1	1	3	2	0	0	0	3	3	2	4	0.440	0.380	5.58E-08	*	W
Y	10	0	2	1	2	4	4	5	0	6	5	1	2	2	8	2	1	1	1	5	1	2	2	1	6	1	5	6	3	2	4	3	0	2	7	2	1	1	2	2	2	3	5	0	4	1	3	2	7	0	7	0.406	0.328	5.55E-08	*	Y
total	131	128	125	116	131	117	132	115	122	130	146	129	149	124	135	127	138	119	128	122	123	100	126	140	133	116	109	132	110	101	126	103	131	85	115	109	103	91	99	103	100	109	98	98	103	100	118	98	114	86	118	0.990	0.131			total

Additional file 5. Continued.

MOUSE x-x-S-x-I

position -4

	C50	C49	C48	C47	C46	C45	C44	C43	C42	C41	C40	C39	C38	C37	C36	C35	C34	C33	C32	C31	C30	C29	C28	C27	C26
A	3	6	7	8	6	3	5	7	4	4	4	6	5	5	7	1	0	4	5	4	4	3	7	2	1
C	2	1	2	0	2	1	4	2	2	1	3	3	4	0	0	1	0	2	2	4	2	4	1	2	1
D	5	7	5	4	4	3	5	2	3	2	2	4	4	7	4	7	4	5	1	3	4	2	3	3	4
E	3	8	7	5	6	2	5	4	6	5	5	10	4	2	2	3	2	7	8	5	1	5	4	7	4
F	3	2	3	3	2	5	4	3	5	1	1	2	5	3	3	4	10	1	3	6	1	5	3	10	7
G	4	6	7	9	3	6	1	3	4	6	6	4	2	4	7	2	3	5	5	7	6	4	2	1	6
H	0	3	3	4	3	2	3	3	1	3	6	2	2	6	6	1	1	2	3	4	0	3	3	2	2
I	6	5	6	3	6	5	6	3	5	2	1	2	2	2	2	1	1	2	4	8	8	6	2	2	3
K	8	3	5	3	2	9	8	4	1	7	8	3	5	10	20	10	5	3	5	5	5	1	6	7	5
L	6	9	7	18	10	11	13	5	8	11	5	12	8	5	7	8	9	10	33	10	11	13	9	8	9
M	1	2	2	0	3	3	0	1	0	1	1	2	1	2	3	0	2	2	3	4	1	2	4	0	4
N	6	3	5	1	6	4	3	3	3	4	5	2	2	3	5	8	6	3	2	1	1	3	2	3	3
P	8	3	7	5	6	5	7	5	6	8	3	6	11	5	1	4	8	2	3	2	7	4	4	4	2
Q	5	6	1	5	2	2	7	3	2	3	3	2	3	3	1	4	1	2	4	3	3	3	5	1	5
R	4	3	2	0	2	3	2	10	2	7	2	6	5	5	4	3	5	1	4	5	8	7	3	8	7
S	10	8	10	7	7	11	7	8	8	9	10	9	4	11	6	2	5	8	8	8	9	8	9	8	8
T	8	3	3	8	13	4	7	8	4	9	8	5	5	5	6	2	4	6	6	5	0	3	5	3	4
V	5	5	2	8	4	6	2	5	4	10	7	11	6	4	4	7	4	4	8	13	6	2	4	6	2
W	0	1	0	3	0	0	0	0	0	3	0	1	2	1	1	1	1	3	1	1	1	0	1	0	1
Y	1	1	4	2	4	1	3	2	3	4	2	2	3	2	4	0	3	2	3	0	3	6	2	2	3
total	88	85	88	96	91	86	92	81	71	100	82	94	83	85	93	69	74	74	111	98	81	84	79	79	81

	C25	C24	C23	C22	C21	C20	C19	C18	C17	C16	C15	C14	C13	C12	C11	C10	C9	C8	C7	C6	C5	C4	C3	C2	C1	C0	C-index	SD	*P-value*	mark	
A	2	3	4	8	3	3	3	4	3	5	9	7	3	4	5	3	7	5	7	4	3	7	2	3	2	9	0.489	0.226	3.12E-05	*	A
C	1	2	4	4	0	0	2	2	1	1	4	0	2	4	5	1	0	2	1	0	3	2	1	4	2	1	1.880	1.394	–	–	C
D	6	9	7	8	9	7	7	4	7	5	8	11	3	5	6	1	7	2	8	2	1	3	4	8	4	4	1.195	0.594	–	–	D
E	5	2	5	5	5	5	3	9	7	4	6	4	3	7	3	3	9	3	3	4	8	2	5	3	3	11	0.429	0.193	5.80E-08	*	E
F	5	3	3	7	4	1	3	4	1	3	1	4	3	1	4	10	5	20	6	2	1	2	3	3	4	4	0.990	0.808	–	–	F
G	3	7	6	3	2	3	7	2	4	3	4	5	7	7	15	3	2	5	2	3	4	5	7	6	1	8	0.573	0.310	8.25E-02	n.s.	G
H	2	1	0	2	0	0	1	0	3	5	0	2	1	2	2	0	3	2	4	2	3	3	0	1	3	0	–	–	–	–	H
I	6	2	11	4	1	6	4	7	5	1	4	4	8	6	13	5	3	6	5	8	4	5	5	2	5	7	0.637	0.369	9.23E-01	n.s.	I
K	3	3	6	3	3	2	11	9	9	6	4	12	5	3	3	3	4	5	2	5	3	6	2	3	4	10	0.534	0.340	3.09E-04	n.s.	K
L	6	11	7	7	15	9	7	4	29	10	11	15	7	9	12	10	15	20	9	16	11	10	6	9	56	10	1.152	0.837	–	–	L
M	2	2	1	3	1	3	2	1	2	3	5	5	1	2	0	2	2	1	1	0	3	1	1	1	0	4	0.445	0.325	2.70E-05	*	M
N	2	1	1	1	3	1	0	3	2	3	1	3	3	4	3	4	4	2	2	4	1	0	2	2	3	2	1.420	0.823	–	–	N
P	4	1	11	4	5	4	4	5	3	7	8	4	6	4	9	4	5	6	5	5	5	6	4	2	5	3	1.680	0.741	–	–	P
Q	1	2	5	2	2	2	6	1	3	5	6	4	5	4	7	5	3	5	5	1	2	3	6	3	4	6	0.570	0.284	1.30E-01	n.s.	Q
R	4	3	6	5	7	6	9	2	4	8	1	5	11	3	4	4	10	6	10	5	5	6	9	4	5	16	0.313	0.163	5.55E-08	*	R
S	7	5	3	6	13	8	10	5	10	10	11	11	6	7	10	7	5	7	8	6	6	7	6	11	4	18	0.430	0.128	5.55E-08	*	S
T	5	4	2	4	5	2	6	4	5	4	7	5	5	2	8	5	4	4	8	0	5	6	3	8	3	2	2.480	1.186	–	–	T
V	8	8	7	5	5	4	5	8	2	2	7	2	3	4	4	6	12	9	5	3	6	6	9	5	3	3	1.847	0.884	–	–	V
W	0	1	1	0	0	0	1	1	0	0	1	0	0	1	0	2	0	0	1	2	0	0	1	3	0	1	0.740	0.899	–	–	W
Y	1	5	2	1	1	1	0	2	1	1	1	2	1	5	6	1	0	1	7	1	3	4	1	7	1	8	0.295	0.221	5.55E-08	*	Y
total	73	75	92	82	84	67	91	77	101	86	99	105	83	84	119	79	100	111	99	73	77	84	77	88	112	127	0.687	0.093			total

position -3

	C50	C49	C48	C47	C46	C45	C44	C43	C42	C41	C40	C39	C38	C37	C36	C35	C34	C33	C32	C31	C30	C29	C28	C27	C26
A	6	8	5	6	6	8	6	6	1	5	7	8	2	6	2	5	4	6	8	8	7	4	4	3	2
C	5	2	4	1	1	2	3	0	1	2	2	2	2	0	3	1	1	3	1	5	1	0	3	2	2
D	1	0	4	3	6	3	4	4	3	6	2	5	3	2	3	2	3	10	5	0	4	7	3	5	6
E	11	5	4	4	4	6	3	2	1	2	5	6	6	2	3	3	6	6	4	5	2	5	3	4	3
F	3	4	3	6	5	1	5	3	7	2	4	6	5	5	6	1	2	4	3	1	8	2	1	3	5
G	5	6	8	5	1	5	3	7	8	8	3	5	6	6	4	6	8	0	3	4	4	6	7	3	5
H	2	3	1	2	4	2	1	0	3	4	2	5	4	2	2	2	1	2	3	9	3	4	3	3	7
I	5	5	3	7	7	5	5	4	4	5	5	3	7	10	4	8	5	2	4	6	3	4	5	4	4
K	5	6	3	6	2	4	3	6	5	2	3	5	5	3	2	5	3	2	6	4	1	4	3	6	3
L	6	5	6	12	12	11	10	11	7	6	12	7	12	8	5	8	12	6	5	20	6	10	8	12	9
M	2	1	2	1	2	1	0	1	0	0	3	1	0	1	1	3	4	1	1	1	1	3	2	3	1
N	1	5	3	2	3	6	4	3	5	8	4	1	6	6	5	2	0	4	5	7	8	8	2	3	5
P	8	5	4	1	14	5	7	8	5	14	4	8	3	6	7	6	6	4	6	5	3	8	8	4	8
Q	7	2	5	4	4	6	3	3	2	3	1	1	2	1	2	2	1	4	5	1	1	0	2	0	1
R	0	4	2	6	3	3	10	2	3	11	4	5	3	1	17	2	1	1	3	1	6	4	5	1	8
S	5	8	14	6	7	4	6	5	4	9	9	11	7	7	8	5	7	4	27	9	11	4	4	7	4
T	6	4	6	13	2	7	8	7	5	6	1	6	2	7	6	1	0	4	10	2	6	2	4	5	2
V	8	8	7	7	7	7	8	7	3	4	8	9	4	8	7	5	5	5	7	5	2	4	8	5	4
W	1	2	1	2	0	0	1	1	0	1	1	0	0	0	2	0	2	0	2	1	1	0	2	1	0
Y	1	2	3	2	1	0	2	1	4	2	2	0	4	4	4	2	3	6	3	4	3	5	2	5	2
total	88	85	88	96	91	86	92	81	71	100	82	94	83	85	93	69	74	74	111	98	81	84	79	79	81

	C25	C24	C23	C22	C21	C20	C19	C18	C17	C16	C15	C14	C13	C12	C11	C10	C9	C8	C7	C6	C5	C4	C3	C2	C1	C0	C-index	SD	*P-value*	mark	
A	3	5	4	4	6	5	7	3	4	3	10	8	5	5	5	9	8	5	5	6	6	2	10	4	5	5	1.080	0.422	–	–	A
C	1	1	4	1	0	3	2	0	2	4	0	2	4	3	3	2	2	2	4	0	3	7	0	0	0	1	1.980	1.571	–	–	C
D	4	4	4	2	4	9	5	2	6	4	7	2	10	5	14	4	1	6	5	5	10	6	0	5	6	4	1.120	0.689	–	–	D
E	3	2	6	5	4	3	6	5	5	2	4	7	2	6	6	5	6	3	4	4	1	2	6	7	3	13	0.326	0.145	5.55E-08	*	E
F	4	1	2	2	2	3	2	2	2	1	5	6	3	7	5	5	8	8	1	2	7	6	3	4	2	4	0.940	0.524	–	–	F
G	0	3	14	4	3	2	4	2	8	4	6	5	8	4	6	4	4	4	9	6	5	1	1	2	1	5	0.944	0.525	–	–	G
H	4	2	2	4	1	2	0	3	4	2	1	1	6	2	2	0	4	1	3	4	2	3	2	5	2	2	1.360	0.863	–	–	H
I	6	11	8	1	3	1	6	3	0	8	9	4	3	1	5	5	5	3	6	2	1	8	6	1	2	5	0.928	0.488	–	–	I
K	6	4	7	0	3	5	7	4	2	4	4	5	4	6	4	5	7	7	3	4	3	1	7	8	5	10	0.424	0.180	5.56E-08	*	K
L	9	7	4	16	14	8	14	11	13	11	14	11	9	11	9	4	5	5	17	13	10	10	12	9	6	8	1.195	0.443	–	–	L
M	0	2	4	5	4	1	0	4	4	0	1	1	1	1	0	1	4	0	1	0	0	1	1	1	1	4	0.370	0.340	3.88E-06	*	M
N	3	3	6	5	3	1	4	4	14	9	9	6	2	4	2	3	4	11	4	1	3	3	4	3	2	3	1.460	0.906	–	–	N
P	5	8	3	6	3	2	5	6	2	7	3	7	1	7	11	6	7	8	9	7	1	2	4	6	4	13	0.442	0.219	5.57E-08	*	P
Q	1	2	1	1	1	1	2	3	4	0	3	5	2	4	5	7	4	7	2	1	4	4	5	3	3	4	0.690	0.467	–	–	Q
R	2	4	3	6	2	1	2	0	2	4	6	4	2	0	4	6	3	4	0	2	3	7	2	3	3	4	0.905	0.767	–	–	R
S	3	6	8	8	10	9	7	10	9	6	9	12	6	5	15	6	7	13	8	5	7	10	6	12	7	22	0.360	0.177	5.55E-08	*	S
T	9	4	1	9	5	3	9	5	4	6	3	6	4	5	7	2	10	13	6	2	3	4	3	7	4	10	0.512	0.294	2.77E-04	n.s.	T
V	8	6	5	2	7	4	4	7	5	5	2	10	7	4	4	4	8	6	9	4	5	4	5	4	54	9	0.744	0.789	–	–	V
W	0	0	0	0	1	1	3	1	2	0	1	1	1	2	2	0	0	0	0	2	1	0	0	2	1	0	–	–	–	–	W
Y	2	0	6	1	8	3	2	2	9	6	2	2	3	2	10	1	3	5	3	3	2	3	0	2	1	1	2.960	2.166	–	–	Y
total	73	75	92	82	84	67	91	77	101	86	99	105	83	84	119	79	100	111	99	73	77	84	77	88	112	127	0.687	0.093			total

position -1

	C50	C49	C48	C47	C46	C45	C44	C43	C42	C41	C40	C39	C38	C37	C36	C35	C34	C33	C32	C31	C30	C29	C28	C27	C26
A	8	5	7	7	9	9	5	6	2	6	4	10	4	3	5	5	5	6	6	3	4	3	6	7	6
C	5	5	2	0	3	0	4	4	2	4	1	5	2	2	2	0	4	1	2	6	1	1	1	1	1
D	4	3	4	4	2	4	7	4	1	5	4	4	5	2	2	1	3	5	25	0	3	6	4	5	4
E	5	6	3	2	5	9	3	5	4	11	3	5	3	2	5	8	3	7	2	3	5	6	7	3	6
F	7	6	3	6	5	3	3	4	5	4	1	5	2	5	1	1	3	4	9	8	4	5	4	3	2
G	4	3	3	6	4	1	7	6	1	5	6	7	7	4	3	1	5	6	9	5	7	7	2	2	8
H	4	5	2	2	0	2	2	1	1	1	1	2	2	2	4	1	5	4	3	2	1	5	3	2	3
I	2	4	8	5	2	5	6	1	3	4	4	3	5	4	3	1	3	4	2	6	4	6	2	2	6
K	2	7	6	0	5	6	4	2	8	7	4	5	2	6	5	5	6	3	4	8	6	3	5	2	4
L	14	6	9	8	13	6	6	10	11	7	13	9	11	9	10	5	4	7	15	5	8	7	6	10	5
M	4	1	1	2	1	1	0	1	0	0	1	2	3	2	3	0	0	1	2	2	2	1	0	2	0
N	3	2	4	15	6	4	3	0	3	2	5	7	5	5	2	4	2	1	0	4	6	1	2	1	5
P	4	2	6	3	3	8	3	4	3	3	2	4	4	3	2	2	2	3	5	3	1	2	4	10	5
Q	3	2	4	4	0	6	4	1	3	4	4	6	5	3	7	1	1	1	1	2	4	5	2	3	1
R	6	5	5	6	6	4	5	5	2	4	4	5	3	6	3	3	7	0	5	5	4	5	6	3	7
S	4	7	8	8	14	7	10	7	4	11	15	6	6	11	1	6	6	8	5	15	6	6	10	6	11
T	3	5	4	8	7	5	10	5	7	5	4	4	7	2	21	16	4	7	8	6	6	7	5	4	4
V	2	8	8	6	6	3	7	11	6	5	1	2	3	7	10	7	8	4	4	11	6	5	4	6	2
W	2	1	0	2	0	0	2	3	1	3	0	2	2	1	1	1	1	0	1	0	2	2	3	4	1
Y	2	2	1	2	0	3	1	1	4	9	5	1	2	6	3	1	2	2	3	4	1	1	3	3	0
total	88	85	88	96	91	86	92	81	71	100	82	94	83	85	93	69	74	74	111	98	81	84	79	79	81

	C25	C24	C23	C22	C21	C20	C19	C18	C17	C16	C15	C14	C13	C12	C11	C10	C9	C8	C7	C6	C5	C4	C3	C2	C1	C0	C-index	SD	*P-value*	mark	
A	7	8	6	5	7	6	8	5	12	7	11	4	6	4	7	3	7	3	7	3	4	4	5	6	4	5	1.160	0.428	–	–	A
C	3	0	1	3	4	2	1	1	4	6	4	4	1	6	0	4	2	0	0	5	0	0	2	3	1	2	1.160	0.923	–	–	C
D	2	3	1	5	3	3	6	2	6	6	8	2	6	3	13	4	8	4	8	3	4	5	2	4	4	7	0.646	0.528	1.19E-02	n.s.	D
E	3	2	14	11	6	5	4	2	6	8	8	7	6	5	14	3	3	2	3	2	7	1	6	4	3	16	0.320	0.185	5.55E-08	*	E
F	5	4	5	3	4	3	2	6	7	4	7	5	1	5	7	5	5	3	7	5	2	3	5	10	4	5	0.880	0.404	–	–	F
G	3	6	3	3	5	4	7	6	2	3	7	9	3	4	3	3	8	8	3	2	4	1	1	3	6	6	0.753	0.378	–	–	G
H	0	2	4	3	2	3	3	4	0	3	1	6	0	5	1	6	5	7	3	2	2	6	0	3	3	6	0.447	0.294	2.90E-05	*	H
I	5	4	7	3	12	4	8	5	5	5	4	5	3	3	2	4	3	1	0	1	6	4	1	5	3	4	0.990	0.539	–	–	I
K	1	2	2	2	1	1	7	4	7	4	5	6	1	9	10	4	9	9	5	8	5	6	8	10	1	11	0.440	0.239	6.06E-07	*	K
L	9	12	7	5	6	9	8	7	8	10	6	7	13	7	7	8	10	16	13	7	4	12	6	1	5	8	1.043	0.392	–	–	L
M	1	1	4	0	4	1	1	1	6	0	1	0	1	1	1	2	0	4	1	3	0	1	3	2	2	3	0.487	0.448	2.31E-03	n.s.	M
N	2	2	3	4	1	1	7	3	0	5	0	6	4	2	6	1	3	7	4	2	1	8	1	6	57	2	2.280	4.015	–	–	N
P	4	1	4	4	2	5	3	1	3	4	3	6	3	1	9	2	9	4	6	3	2	1	2	3	1	0	–	–	–	–	P
Q	3	3	10	3	2	1	3	4	1	3	6	4	7	3	1	4	4	9	2	5	4	5	3	3	3	5	0.692	0.414	–	–	Q
R	3	5	6	1	4	4	5	6	4	3	5	5	3	3	11	6	5	4	7	2	5	7	9	3	0	14	0.329	0.143	5.55E-08	*	R
S	6	6	5	13	9	8	7	12	15	7	6	11	10	10	7	4	8	8	12	9	10	8	11	10	4	11	0.753	0.286	–	–	S
T	3	5	5	4	2	2	2	1	6	2	3	8	4	2	3	6	7	2	1	5	8	3	3	3	4	10	0.516	0.349	2.86E-05	*	T
V	6	4	3	5	5	4	7	5	6	2	9	3	3	5	12	5	2	5	5	1	7	3	2	3	4	4	1.290	0.653	–	–	V
W	1	3	1	3	2	1	1	0	1	0	0	2	4	0	3	3	0	13	7	3	1	2	1	4	0	4	0.455	0.539	2.72E-05	*	W
Y	6	2	1	2	3	0	1	2	2	4	5	5	4	6	2	2	2	2	5	2	1	4	6	2	3	4	0.680	0.468	4.06E-01	n.s.	Y
total	73	75	92	82	84	67	91	77	101	86	99	105	83	84	119	79	100	111	99	73	77	84	77	88	112	127	0.687	0.093			total

Additional file 5. Continued.

MOUSE x-x-S-x-L

position -4

	C50	C49	C48	C47	C46	C45	C44	C43	C42	C41	C40	C39	C38	C37	C36	C35	C34	C33	C32	C31	C30	C29	C28	C27	C26	C25	C24	C23	C22	C21	C20	C19	C18	C17	C16	C15	C14	C13	C12	C11	C10	C9	C8	C7	C6	C5	C4	C3	C2	C1	C0	C-index	SD	*P-value*	mark	
A	9	8	6	18	17	14	13	18	20	15	16	10	11	22	9	9	10	13	14	17	12	20	13	19	16	11	27	19	12	17	20	14	13	18	14	17	9	16	15	14	14	16	16	8	20	14	12	23	15	14	18	0.819	0.237	–	–	A
C	4	2	8	5	5	4	6	3	6	3	5	4	5	3	4	3	5	1	9	12	5	5	8	3	6	2	3	4	1	4	2	4	8	8	10	5	4	1	4	7	7	3	4	3	2	3	21	10	5	8	5	1.028	0.674	–	–	C
D	11	9	17	8	8	9	8	12	11	12	17	7	9	16	8	16	14	11	14	12	6	4	3	9	12	10	11	7	8	7	15	7	6	13	7	7	8	10	3	7	11	6	8	6	9	9	9	8	12	8	11	0.864	0.305	–	–	D
E	20	11	21	12	16	30	10	9	9	14	10	33	7	9	14	8	15	11	14	7	7	10	11	10	6	9	5	12	12	7	17	20	15	14	15	10	15	9	12	9	11	12	10	6	13	9	9	10	11	16	12	1.020	0.449	–	–	E
F	6	4	9	6	12	8	4	13	10	5	9	9	11	15	13	12	6	9	10	11	10	9	8	13	14	9	8	4	9	16	11	4	13	7	7	8	9	10	10	5	4	12	10	10	14	10	10	15	9	12	17	0.555	0.184	1.21E-06	*	F
G	9	10	18	9	17	22	22	8	17	14	15	13	12	16	18	10	15	17	13	11	12	24	13	10	10	17	8	11	7	14	15	12	14	15	14	17	18	9	14	3	7	18	11	17	15	15	12	16	16	9	15	0.905	0.279	–	–	G
H	7	4	6	2	9	4	5	5	9	9	13	5	12	10	7	6	8	8	9	5	7	5	7	10	2	6	10	8	7	4	0	4	7	4	2	4	6	2	13	8	4	6	8	4	4	3	8	5	5	3	10	0.618	0.288	8.74E-02	n.s.	H
I	10	8	7	14	10	9	7	5	7	12	7	13	10	17	29	10	8	7	16	12	8	9	10	14	5	9	9	21	9	7	12	8	13	11	6	7	9	8	11	19	13	12	8	3	7	13	10	13	4	10	16	0.645	0.281	4.79E-03	n.s.	I
K	9	5	18	13	11	10	8	14	5	8	15	8	7	7	13	11	12	7	7	13	4	11	14	14	10	9	6	8	12	11	10	6	10	12	5	7	7	10	10	9	12	12	11	10	9	14	16	12	9	14	22	0.459	0.142	5.55E-08	*	K
L	29	18	28	24	23	39	33	29	37	28	36	15	28	31	21	23	29	28	36	24	33	33	28	13	27	15	16	26	21	21	33	26	33	27	23	26	28	26	31	24	24	24	34	30	31	38	19	26	41	37	32	0.858	0.203	–	–	L
M	6	3	6	5	6	5	8	4	5	5	4	7	10	10	6	1	6	5	5	7	3	3	5	6	3	2	13	4	4	4	3	3	4	4	2	6	11	1	9	3	6	5	4	4	0	4	2	2	5	3	7	0.691	0.368	4.09E-01	n.s.	M
N	12	10	8	6	10	8	7	5	9	10	4	7	10	6	9	5	6	7	4	10	7	3	5	8	5	7	3	7	8	7	11	10	4	8	10	10	8	5	6	9	2	4	9	6	5	7	7	9	4	10	12	0.595	0.198	3.32E-03	n.s.	N
P	11	10	13	12	13	17	14	23	12	21	14	15	12	13	15	15	7	11	10	14	17	10	11	12	18	14	25	13	12	21	28	13	18	14	12	17	16	14	18	12	7	14	23	11	14	13	11	34	19	14	18	0.830	0.282	–	–	P
Q	13	8	8	12	10	12	9	9	18	6	6	7	10	24	7	10	6	2	6	6	10	7	6	13	10	9	8	8	8	4	12	14	6	12	29	12	13	16	16	12	16	12	17	11	9	15	9	8	10	10	9	1.180	0.532	–	–	Q
R	6	11	9	11	13	8	16	5	15	11	12	11	20	5	12	15	8	11	10	16	13	10	9	13	7	7	12	9	18	12	9	6	11	10	17	12	14	20	16	12	8	14	17	16	8	9	10	14	12	12	31	0.375	0.118	5.55E-08	*	R
S	15	26	18	16	21	15	31	30	15	32	21	16	23	21	24	22	27	23	23	18	17	17	21	16	27	19	18	31	15	24	22	23	17	26	24	12	15	14	16	29	17	28	29	19	20	18	20	17	20	25	27	0.780	0.191	–	–	S
T	16	7	14	10	10	18	10	12	16	15	10	15	8	10	14	15	13	21	10	13	17	14	16	13	12	15	15	15	15	20	17	11	12	18	13	8	8	13	11	31	11	11	25	14	8	6	8	10	10	13	19	0.702	0.240	5.27E-01	n.s.	T
V	13	17	15	9	21	10	11	20	13	17	11	14	8	20	13	13	12	21	22	17	20	11	13	6	5	9	19	12	17	23	26	11	12	17	13	19	20	14	16	22	16	17	19	15	15	8	20	7	17	15	10	1.502	0.477	–	–	V
W	2	0	2	4	4	3	3	4	1	1	6	1	2	2	1	4	1	3	2	2	2	4	1	3	4	3	4	1	1	1	2	5	1	3	2	1	4	3	3	4	3	4	3	4	3	4	8	0	2	0	3	0.873	0.530	–	–	W
Y	6	5	2	8	5	8	8	6	12	4	5	7	8	2	1	3	5	6	4	3	6	3	4	8	1	6	9	3	9	1	3	10	5	7	3	6	3	7	10	5	5	5	8	5	9	3	7	8	7	4	12	0.463	0.214	5.58E-08	*	Y
total	214	176	233	204	241	253	233	234	247	242	236	217	223	259	238	211	213	222	238	230	216	212	206	213	200	188	229	223	205	225	268	211	222	248	228	211	225	208	244	244	198	235	274	202	215	215	228	247	233	237	306	0.737	0.064			total

position -3

	C50	C49	C48	C47	C46	C45	C44	C43	C42	C41	C40	C39	C38	C37	C36	C35	C34	C33	C32	C31	C30	C29	C28	C27	C26	C25	C24	C23	C22	C21	C20	C19	C18	C17	C16	C15	C14	C13	C12	C11	C10	C9	C8	C7	C6	C5	C4	C3	C2	C1	C0	C-index	SD	*P-value*	mark	
A	13	10	19	14	9	33	15	11	20	16	7	17	15	19	9	16	22	15	19	11	20	14	13	12	17	21	7	15	13	22	18	17	11	16	18	15	15	17	12	17	5	9	17	11	15	11	14	11	22	13	18	0.831	0.268	–	–	A
C	4	3	4	8	6	15	9	4	7	4	2	10	3	13	6	8	7	4	7	9	4	9	3	5	8	2	5	8	2	4	8	13	5	10	4	4	8	8	8	11	4	5	8	5	4	3	5	28	3	18	19	0.363	0.242	5.55E-08	*	C
D	9	21	12	9	7	11	7	9	17	8	11	17	14	11	15	13	11	8	3	7	8	14	10	11	13	11	11	8	6	12	14	6	7	7	6	6	10	10	11	11	17	7	10	9	7	9	9	8	15	9	28	0.366	0.125	5.55E-08	*	D
E	12	5	9	12	8	6	9	13	10	18	13	6	14	13	7	7	12	11	10	13	7	16	5	7	8	10	10	8	7	9	12	9	11	10	15	9	8	4	10	7	16	10	14	7	13	16	5	12	11	9	27	0.373	0.121	5.55E-08	*	E
F	13	7	11	9	11	18	6	15	6	11	5	9	6	11	18	8	12	6	7	14	12	6	11	7	8	6	13	5	10	10	9	8	11	10	8	10	3	6	14	14	2	9	12	7	12	14	10	16	8	13	19	0.513	0.189	5.97E-08	*	F
G	17	9	13	13	14	17	15	17	22	25	19	35	13	16	15	16	16	15	10	13	17	24	11	12	11	7	15	14	9	13	9	15	9	8	11	11	12	17	4	8	7	20	15	15	12	12	15	12	14	13	14	1.003	0.368	–	–	G
H	7	5	11	4	5	8	9	12	8	9	8	4	4	10	17	4	2	3	3	4	4	5	4	10	5	4	11	6	5	6	11	6	6	8	9	8	7	6	6	5	8	12	12	7	5	4	3	9	13	15	11	0.649	0.305	1.10E-01	n.s.	H
I	12	7	19	18	9	12	7	8	7	12	7	6	5	18	8	10	9	15	16	8	11	8	10	3	16	10	23	14	13	6	13	13	13	18	10	5	11	9	10	10	9	10	9	6	8	11	15	11	9	13	10	1.080	0.409	–	–	I
K	9	7	12	6	6	10	13	9	13	10	10	7	11	9	15	10	7	8	15	10	11	6	17	13	8	6	8	9	10	9	6	5	12	11	5	16	24	13	22	14	11	15	17	10	8	5	12	8	10	9	10	1.054	0.407	–	–	K
L	18	9	24	22	32	30	22	27	27	20	31	22	45	27	20	24	27	24	26	28	23	12	17	23	27	24	27	32	32	30	43	22	29	22	29	28	23	23	26	31	18	23	32	33	19	17	18	29	21	24	36	0.701	0.180	5.91E-01	n.s.	L
M	1	3	3	4	5	1	6	2	3	2	3	5	5	8	3	6	2	3	8	3	4	4	2	5	3	1	7	5	5	2	6	2	1	6	3	7	3	4	5	3	4	6	6	1	3	7	5	7	6	3	3	1.347	0.649	–	–	M
N	3	10	9	7	10	3	9	11	10	14	12	4	6	7	9	4	9	11	9	9	3	7	5	11	3	6	8	13	6	11	12	6	8	11	3	5	8	10	6	3	7	6	19	11	6	13	9	8	9	15	3	2.760	1.161	–	–	N
P	18	11	21	11	21	12	21	16	16	19	23	6	13	15	14	18	14	13	28	18	17	12	15	20	15	13	15	12	9	16	13	19	18	26	14	11	13	5	19	16	9	20	16	10	12	11	16	11	13	14	18	0.842	0.254	–	–	P
Q	8	10	3	8	6	6	6	10	9	7	9	10	11	6	11	11	14	6	8	4	8	5	11	13	7	10	8	13	10	12	16	9	10	17	11	13	6	7	16	19	7	7	11	7	17	12	10	10	15	5	12	0.808	0.300	–	–	Q
R	8	10	10	13	13	10	15	14	17	13	11	9	9	10	10	9	4	11	14	14	12	16	11	6	7	12	10	7	10	8	15	11	23	8	15	13	16	11	11	18	14	19	11	12	9	11	32	11	9	12	17	0.711	0.264	2.27E-01	n.s.	R
S	23	19	28	23	40	33	30	25	18	28	33	21	15	29	31	15	19	25	24	29	19	17	26	22	21	20	21	17	21	27	21	20	20	30	39	29	25	25	27	27	25	20	30	23	35	26	30	19	21	25	29	0.852	0.197	–	–	S
T	13	16	7	8	12	10	14	8	15	11	14	11	15	14	10	14	5	13	9	10	16	13	14	14	8	7	9	14	9	13	9	12	14	9	12	11	13	16	12	7	8	14	10	3	6	16	1	11	7	9	13	0.840	0.267	–	–	T
V	17	8	8	10	10	7	16	12	14	12	12	8	11	16	11	12	17	13	13	15	12	12	14	13	6	10	12	15	17	10	20	11	4	15	12	6	9	11	16	15	19	15	14	14	15	9	9	18	15	7	13	0.949	0.276	–	–	V
W	2	3	0	1	12	3	0	4	0	0	1	1	1	2	2	3	1	7	2	1	0	2	5	2	4	3	1	1	2	1	2	3	1	0	0	2	3	0	2	5	3	4	2	4	2	1	2	1	5	6	2	1.150	1.084	–	–	W
Y	7	3	10	4	5	8	4	7	8	3	5	9	7	5	7	3	3	11	7	10	8	10	2	4	5	5	8	7	9	4	11	4	9	6	4	2	8	6	7	3	5	4	9	7	7	7	8	7	7	5	4	1.570	0.596	–	–	Y
total	214	176	233	204	241	253	233	234	247	242	236	217	223	259	238	211	213	222	238	230	216	212	206	213	200	188	229	223	205	225	268	211	222	248	228	211	225	208	244	244	198	235	274	202	215	215	228	247	233	237	306	0.737	0.064			total

position -1

	C50	C49	C48	C47	C46	C45	C44	C43	C42	C41	C40	C39	C38	C37	C36	C35	C34	C33	C32	C31	C30	C29	C28	C27	C26	C25	C24	C23	C22	C21	C20	C19	C18	C17	C16	C15	C14	C13	C12	C11	C10	C9	C8	C7	C6	C5	C4	C3	C2	C1	C0	C-index	SD	*P-value*	mark	
A	15	6	11	14	18	15	16	15	13	18	15	19	12	16	15	8	12	16	22	12	12	18	15	16	18	11	11	12	12	18	22	12	21	17	16	16	12	21	14	16	10	18	20	16	20	10	12	7	18	12	18	0.823	0.210	–	–	A
C	6	5	10	10	6	3	7	4	8	12	5	7	7	8	5	4	6	2	12	7	6	3	1	2	11	9	11	3	12	6	12	5	5	6	5	5	4	9	10	2	2	6	12	7	7	9	4	7	11	21	19	0.365	0.194	5.55E-08	*	C
D	11	6	18	8	12	13	14	10	8	8	6	9	3	11	13	8	16	11	9	11	11	7	3	11	11	6	4	11	12	8	14	8	9	8	11	5	12	6	15	16	15	11	7	6	4	3	9	14	8	7	15	0.636	0.243	1.14E-01	n.s.	D
E	9	7	12	15	10	8	15	21	11	12	23	12	9	15	6	13	4	9	15	11	9	10	9	12	10	10	7	8	9	11	15	9	6	10	8	10	13	11	10	8	18	13	7	10	9	13	4	15	7	10	15	0.717	0.251	4.27E-01	n.s.	E
F	15	15	17	8	5	9	10	10	13	10	9	8	17	9	4	8	9	6	7	16	11	4	4	11	11	5	13	16	8	10	22	8	8	11	9	8	7	13	3	16	7	11	7	8	11	11	13	9	12	10	17	0.591	0.229	5.75E-05	*	F
G	14	8	18	8	12	31	13	16	30	9	13	9	16	17	10	17	13	11	9	13	11	15	15	16	13	5	9	11	11	15	15	12	12	13	9	7	10	11	12	6	14	16	13	16	10	10	12	10	7	13	11	1.156	0.433	–	–	G
H	5	10	12	7	7	11	6	10	8	5	7	5	7	11	7	4	5	5	8	11	8	4	11	3	8	9	5	9	5	12	5	2	6	11	15	13	14	8	16	8	10	11	10	5	5	7	10	12	8	10	20	0.411	0.159	5.55E-08	*	H
I	15	8	8	7	6	20	12	10	9	10	9	19	9	18	22	7	8	14	14	6	11	21	10	8	7	10	15	8	8	8	6	12	14	17	11	8	11	15	9	22	9	12	25	7	9	6	12	8	16	8	11	1.044	0.439	–	–	I
K	14	6	8	10	7	12	21	9	6	16	7	6	6	7	11	8	13	6	8	10	12	6	9	15	6	10	18	16	9	7	18	7	9	5	7	9	10	10	10	11	10	6	16	7	12	16	10	9	20	9	26	0.392	0.154	5.55E-08	*	K
L	23	23	25	25	33	24	21	20	20	18	20	20	14	32	29	22	29	28	23	28	23	22	24	23	23	18	28	22	13	32	24	30	18	30	18	16	13	18	27	24	22	30	26	25	24	27	21	33	34	27	29	0.822	0.179	–	–	L
M	2	1	8	2	6	6	2	1	7	5	6	2	4	7	2	5	6	5	1	5	9	7	4	0	9	11	6	3	5	1	6	4	5	4	1	2	3	1	5	1	5	2	5	5	3	2	17	3	1	12	1	4.500	3.259	–	–	M
N	8	6	8	9	8	5	5	7	6	14	9	10	12	7	7	6	7	14	5	7	5	7	6	4	3	5	9	9	8	2	5	13	18	12	10	9	8	10	13	3	10	7	13	5	3	11	11	7	7	2	9	0.878	0.378	–	–	N
P	5	8	13	5	12	17	14	9	19	21	17	11	12	18	15	11	14	11	15	20	22	9	13	10	10	11	9	16	14	15	12	10	19	27	15	16	9	10	8	13	7	13	14	15	10	8	9	15	10	22	14	0.940	0.329	–	–	P
Q	8	10	8	11	10	11	12	9	10	8	9	11	16	7	8	6	6	13	14	6	10	9	9	3	6	5	10	7	9	15	9	9	11	15	11	16	15	11	10	10	5	5	10	12	9	5	6	6	6	11	15	0.624	0.206	7.12E-04	n.s.	Q
R	13	9	9	19	20	29	10	12	11	17	12	13	12	10	28	11	10	13	10	12	8	10	14	23	12	7	11	11	9	9	11	9	10	9	13	13	13	6	13	17	7	9	13	10	15	18	14	18	12	8	16	0.790	0.299	–	–	R
S	24	19	21	22	34	13	16	24	23	37	30	26	29	34	33	30	31	21	34	24	22	21	26	20	16	28	22	26	24	21	31	40	21	12	38	29	25	22	21	31	20	28	36	22	27	18	26	21	25	24	28	0.906	0.225	–	–	S
T	7	9	10	9	16	6	11	11	12	6	11	11	14	10	5	18	9	10	13	4	8	15	17	9	12	8	15	11	12	17	18	4	7	12	11	11	17	14	19	8	7	10	5	7	14	13	6	15	10	13	17	0.644	0.229	5.32E-02	n.s.	T
V	12	11	13	4	8	11	18	20	19	8	22	11	12	17	11	21	10	19	13	12	13	14	10	20	11	13	16	19	15	11	9	12	17	20	14	12	15	4	16	21	14	16	14	12	8	18	22	28	9	11	17	0.831	0.285	–	–	V
W	1	2	0	2	2	3	1	3	4	4	1	1	5	1	1	0	0	4	3	5	2	6	2	3	1	2	3	2	3	3	1	2	0	4	0	2	2	3	8	2	3	5	9	1	3	3	7	5	6	4	3	0.933	0.683	–	–	W
Y	7	7	4	9	9	6	9	13	10	4	5	7	7	4	6	4	5	4	3	10	3	4	4	4	2	5	7	3	7	4	13	3	6	5	6	4	12	5	5	9	3	6	12	6	12	7	3	5	6	3	5	1.228	0.576	–	–	Y
total	214	176	233	204	241	253	233	234	247	242	236	217	223	259	238	211	213	222	238	230	216	212	206	213	200	188	229	223	205	225	268	211	222	248	228	211	225	208	244	244	198	235	274	202	215	215	228	247	233	237	306	0.737	0.064			total

Additional file 5. Continued.

MOUSE x-x-S-x-V

position -4

	C50	C49	C48	C47	C46	C45	C44	C43	C42	C41	C40	C39	C38	C37	C36	C35	C34	C33	C32	C31	C30	C29	C28	C27	C26	C25	C24	C23	C22	C21	C20	C19	C18	C17	C16	C15	C14	C13	C12	C11	C10	C9	C8	C7	C6	C5	C4	C3	C2	C1	C0	C-index	SD	*P-value*	mark	
A	6	7	4	12	8	4	4	11	6	12	10	8	12	5	12	2	3	10	7	11	6	8	15	10	7	12	9	8	3	19	11	9	15	7	10	7	3	13	4	1	8	8	8	10	8	4	5	5	10	9	12	0.677	0.306	–	–	A
C	0	3	3	3	2	4	0	3	1	7	3	3	4	3	6	6	2	2	3	2	1	3	0	2	1	3	1	4	3	2	2	1	1	3	1	5	0	1	3	4	1	2	5	3	5	2	2	1	0	3	3	0.833	0.548	–	–	C
D	1	3	4	7	5	5	2	8	8	3	4	6	2	11	5	7	4	7	5	7	5	6	3	3	9	6	4	8	5	3	7	7	5	4	9	12	5	12	4	1	11	4	3	6	6	13	4	4	2	4	10	0.558	0.285	1.45E-01	n.s.	D
E	3	6	5	10	11	7	12	9	8	9	2	3	6	11	9	6	8	10	8	6	5	10	10	10	7	6	5	4	6	5	6	4	4	14	7	9	3	5	11	5	8	5	13	9	7	5	6	9	9	11	13	0.565	0.216	7.93E-01	n.s.	E
F	3	7	4	8	6	4	10	8	2	5	4	8	4	3	6	8	10	8	1	3	4	9	9	6	14	4	3	7	5	4	4	4	5	4	2	3	1	3	6	5	6	3	9	4	2	6	8	2	6	1	7	0.746	0.392	–	–	F
G	9	8	3	10	7	8	15	6	7	7	8	10	10	7	7	7	7	3	5	6	6	7	9	5	5	5	5	16	6	9	6	12	5	10	11	8	4	7	7	5	8	7	5	8	7	13	6	6	4	9	10	0.742	0.271	–	–	G
H	7	3	3	1	6	2	4	4	5	4	2	2	3	3	0	4	5	1	5	6	2	5	6	1	3	3	2	0	4	3	5	1	6	3	4	4	1	3	4	4	2	8	1	1	5	5	2	2	4	3	3	1.113	0.608	–	–	H
I	6	9	9	7	2	44	3	5	5	7	5	5	6	10	6	5	6	7	9	7	4	6	7	10	4	4	4	5	4	5	5	8	7	3	2	4	5	6	8	8	4	4	4	3	5	3	5	8	4	4	8	0.790	0.724	–	–	I
K	5	7	5	4	5	6	3	9	3	7	9	6	11	5	13	23	17	6	5	10	4	7	8	5	7	9	3	7	4	4	2	2	7	7	6	7	7	8	7	5	8	7	4	3	10	6	10	7	8	8	13	0.532	0.279	1.44E-03	n.s.	K
L	14	26	15	8	17	18	9	16	17	9	11	9	10	13	11	15	12	22	18	15	11	24	39	13	12	11	21	14	24	9	13	10	5	16	10	14	13	8	10	19	8	11	22	16	8	12	6	12	14	12	21	0.669	0.285	–	–	L
M	0	2	6	2	0	2	1	2	3	4	3	5	1	6	2	4	4	5	3	7	2	3	8	3	5	3	2	2	2	1	3	2	2	2	1	6	2	2	2	2	4	1	1	0	1	3	3	3	3	3	4	0.695	0.441	–	–	M
N	5	2	3	5	1	5	2	10	3	4	2	11	8	2	4	7	3	5	5	4	3	5	2	5	3	2	4	1	1	0	6	3	3	6	3	5	3	2	5	4	2	2	5	3	5	3	2	5	6	0	8	0.475	0.278	2.20E-03	n.s.	N
P	12	7	14	8	12	7	8	10	7	15	9	9	6	8	7	3	8	5	5	11	10	5	15	11	9	8	8	9	10	8	12	6	7	14	7	13	8	9	4	11	4	10	5	6	8	17	7	12	5	17	18	0.496	0.186	4.74E-04	n.s.	P
Q	10	8	9	5	22	5	6	3	18	1	7	4	4	6	9	4	6	8	7	3	5	6	4	4	3	3	5	9	11	5	2	7	1	7	1	5	5	7	2	1	6	5	6	4	4	4	7	6	7	3	7	0.829	0.539	–	–	Q
R	6	8	12	6	10	6	2	4	4	5	9	7	8	6	8	5	4	6	3	5	11	2	8	4	9	7	6	4	5	5	7	3	1	5	1	8	6	13	6	7	10	11	4	5	10	6	3	6	6	15	15	0.424	0.200	5.67E-07	*	R
S	13	13	13	10	12	10	14	8	9	9	10	12	5	18	12	4	9	6	11	11	20	6	10	10	8	10	20	9	6	12	12	15	10	13	13	10	12	11	10	8	7	9	14	17	18	12	8	18	9	11	26	0.428	0.140	5.73E-08	*	S
T	13	13	8	9	9	13	7	7	10	7	3	6	11	4	6	4	3	4	10	5	6	4	6	8	5	4	7	8	2	2	2	7	8	4	15	7	9	3	9	6	13	9	6	10	5	6	6	12	12	7	11	0.655	0.296	–	–	T
V	10	6	8	12	6	13	8	9	11	5	7	11	6	5	11	3	5	6	10	7	6	10	11	7	3	14	7	10	4	6	6	5	6	7	5	5	7	3	9	8	10	10	10	9	9	5	5	8	8	7	8	0.948	0.328	–	–	V
W	1	1	1	0	2	2	0	0	0	0	1	2	2	1	1	1	1	3	2	1	2	1	0	0	3	5	2	2	1	0	2	1	0	2	2	2	1	2	1	3	4	0	1	2	0	0	0	1	1	0	1	1.260	1.121	–	–	W
Y	6	5	4	2	2	4	4	5	1	3	1	6	1	3	3	2	4	1	2	2	2	4	1	1	5	2	8	4	2	0	1	0	0	2	4	0	2	1	3	5	8	6	1	6	1	3	4	2	6	2	5	0.588	0.411	7.56E-01	n.s.	Y
total	130	144	133	129	145	169	114	137	128	123	110	133	120	130	138	120	121	125	124	129	115	131	171	118	122	121	126	131	108	102	114	107	98	133	114	134	97	119	115	112	132	122	127	125	124	128	99	129	124	129	203	0.614	0.071			total

position -3

	C50	C49	C48	C47	C46	C45	C44	C43	C42	C41	C40	C39	C38	C37	C36	C35	C34	C33	C32	C31	C30	C29	C28	C27	C26	C25	C24	C23	C22	C21	C20	C19	C18	C17	C16	C15	C14	C13	C12	C11	C10	C9	C8	C7	C6	C5	C4	C3	C2	C1	C0	C-index	SD	*P-value*	mark	
A	12	5	11	5	9	8	11	13	10	13	4	7	7	9	9	11	8	6	13	6	10	7	14	7	12	4	12	9	6	6	9	7	10	6	8	9	9	11	6	9	7	6	6	6	7	8	6	3	2	12	17	0.484	0.165	2.34E-04	n.s.	A
C	3	4	3	3	4	6	7	5	2	1	2	1	4	1	7	2	1	4	3	0	1	4	4	2	1	3	6	9	2	0	2	5	3	2	4	1	1	4	1	3	3	4	2	5	0	5	1	3	2	3	3	0.993	0.651	–	–	C
D	3	6	5	5	8	3	8	6	10	4	9	8	8	6	7	5	11	9	5	3	10	3	6	7	9	5	5	4	24	7	7	3	4	4	8	4	5	4	5	4	5	8	4	6	9	5	2	4	11	5	11	0.575	0.311	1.86E-01	n.s.	D
E	1	5	5	8	4	3	2	6	5	5	5	5	5	5	9	2	3	6	9	3	2	12	8	10	7	5	5	4	4	4	4	8	11	8	5	3	1	2	9	4	13	4	8	5	6	5	2	5	5	6	32	0.169	0.085	5.55E-08	*	E
F	9	3	3	8	4	4	5	7	6	11	4	5	6	3	2	3	4	16	6	3	4	3	7	1	5	4	5	2	1	3	2	4	5	6	1	7	4	8	8	3	3	8	8	6	6	2	4	11	9	2	7	0.726	0.421	–	–	F
G	2	14	6	6	9	50	5	9	7	8	7	15	7	10	10	8	9	8	8	9	9	11	2	10	10	10	9	8	12	7	7	8	4	9	3	11	8	8	6	5	9	7	4	7	4	10	10	5	5	4	13	0.660	0.506	–	–	G
H	3	0	5	5	3	2	3	2	4	0	4	8	4	2	3	2	1	3	6	8	12	3	8	4	5	2	3	6	5	5	1	2	1	7	6	7	4	6	5	3	3	2	4	12	1	5	3	0	3	4	6	0.667	0.439	–	–	H
I	3	13	5	5	6	17	7	10	4	6	9	13	4	9	6	11	9	3	10	3	5	8	3	2	3	9	7	7	2	3	6	4	6	4	8	4	5	5	5	7	5	7	9	7	6	4	2	8	3	9	10	0.632	0.315	–	–	I
K	11	3	5	7	4	6	2	3	5	5	2	8	7	2	3	2	5	5	9	7	5	5	15	5	3	6	5	6	2	3	6	7	2	4	14	4	5	2	5	5	6	6	2	3	5	1	3	5	4	7	6	0.840	0.469	–	–	K
L	20	15	11	16	16	6	9	19	17	12	7	9	14	11	12	6	10	8	6	20	14	10	13	15	11	12	14	5	8	12	13	12	9	17	5	15	7	14	19	9	19	19	10	11	24	17	9	11	13	11	14	0.889	0.317	–	–	L
M	1	2	5	1	4	2	2	1	0	1	1	2	5	4	4	3	2	5	3	1	4	1	2	1	3	1	1	2	1	2	1	0	0	5	1	6	1	3	3	2	2	3	3	1	6	2	1	3	4	2	2	1.160	0.779	–	–	M
N	8	4	5	5	5	5	3	10	6	2	2	2	5	9	8	5	2	3	5	4	5	3	8	6	2	2	2	2	7	14	4	6	1	4	9	7	7	4	7	2	7	5	9	9	3	4	8	4	3	4	3	1.707	0.895	–	–	N
P	11	12	11	12	18	9	6	6	10	12	12	8	5	7	3	3	10	7	7	9	7	8	11	7	8	12	13	7	3	8	9	6	5	10	4	12	3	8	6	10	6	9	8	9	5	10	7	10	8	4	14	0.587	0.219	9.98E-01	n.s.	P
Q	6	10	7	7	3	3	12	7	5	10	5	2	3	5	4	7	5	5	1	3	2	3	4	6	1	6	6	9	3	2	3	6	2	7	2	4	3	10	5	3	3	5	6	4	2	1	7	10	6	5	13	0.378	0.203	5.83E-08	*	Q
R	6	4	6	3	3	6	7	5	4	2	3	9	7	13	10	5	7	7	3	8	2	8	8	2	11	3	9	8	11	3	6	6	3	7	4	9	1	5	4	3	6	8	7	1	4	12	7	9	4	11	4	1.500	0.746	–	–	R
S	15	14	13	11	21	20	10	11	7	9	13	9	12	12	9	13	11	9	8	12	9	11	16	12	12	9	9	16	8	6	12	8	15	13	13	13	14	12	11	12	14	7	15	19	17	13	12	10	21	19	16	0.771	0.224	–	–	S
T	8	8	8	9	10	9	7	6	11	6	4	7	3	5	10	3	7	9	5	8	2	5	7	7	2	8	4	10	0	7	12	5	10	6	8	11	7	5	4	10	11	5	8	7	3	8	4	10	7	9	20	0.345	0.136	5.55E-08	*	T
V	5	14	13	6	9	6	4	7	9	7	13	8	9	9	16	24	12	4	7	9	4	12	24	8	3	11	9	7	6	6	3	6	5	9	8	5	7	4	2	10	7	7	11	4	7	7	6	8	12	8	9	0.927	0.492	–	–	V
W	1	2	4	2	3	4	1	3	1	3	1	3	1	1	4	1	2	2	3	1	4	2	2	2	2	1	2	3	3	1	2	0	1	2	1	1	0	1	0	1	1	0	1	2	1	2	1	1	0	1	1	1.680	1.096	–	–	W
Y	2	6	2	5	2	0	3	1	5	6	3	4	4	7	2	4	2	6	7	12	4	12	9	4	12	8	0	7	0	3	5	4	1	3	2	1	5	3	4	7	2	2	2	1	8	7	4	9	2	3	2	2.170	1.531	–	–	Y
total	130	144	133	129	145	169	114	137	128	123	110	133	120	130	138	120	121	125	124	129	115	131	171	118	122	121	126	131	108	102	114	107	98	133	114	134	97	119	115	112	132	122	127	125	124	128	99	129	124	129	203	0.614	0.071			total

position -1

	C50	C49	C48	C47	C46	C45	C44	C43	C42	C41	C40	C39	C38	C37	C36	C35	C34	C33	C32	C31	C30	C29	C28	C27	C26	C25	C24	C23	C22	C21	C20	C19	C18	C17	C16	C15	C14	C13	C12	C11	C10	C9	C8	C7	C6	C5	C4	C3	C2	C1	C0	C-index	SD	*P-value*	mark	
A	10	10	12	6	19	11	11	9	9	9	7	11	10	13	18	6	7	8	7	13	10	7	9	5	9	9	10	8	8	2	4	11	7	10	8	14	10	9	10	5	15	6	6	2	10	13	5	5	8	8	14	0.641	0.244	–	–	A
C	2	4	3	3	2	4	3	3	1	2	3	3	3	3	3	2	8	3	6	5	0	1	4	4	4	1	2	10	0	4	4	0	3	2	3	4	2	2	0	1	2	6	5	2	6	2	1	1	3	3	5	0.592	0.392	5.96E-01	n.s.	C
D	8	5	6	7	5	7	3	8	9	4	9	7	4	3	4	7	0	5	2	5	3	5	6	4	1	7	5	3	23	3	2	4	4	5	3	3	3	5	8	3	6	2	4	5	3	1	5	6	4	4	11	0.451	0.302	3.41E-06	*	D
E	6	2	10	10	3	10	6	12	7	6	6	6	5	14	14	6	6	5	6	7	9	8	21	9	2	9	9	7	6	9	7	5	4	11	5	7	5	8	3	4	4	6	8	9	8	9	1	11	8	5	15	0.485	0.231	9.68E-05	*	E
F	6	5	11	5	3	7	2	6	3	2	5	4	6	4	5	4	5	1	10	9	5	5	7	4	6	4	0	3	3	0	4	3	3	2	6	6	1	1	3	9	3	1	5	2	8	4	5	5	12	7	6	0.767	0.445	–	–	F
G	3	9	7	9	5	9	4	7	3	4	8	8	5	5	5	3	12	7	4	4	4	7	12	10	4	7	3	7	4	9	9	3	6	9	9	8	2	11	10	5	12	13	4	5	7	7	4	9	4	4	10	0.658	0.286	–	–	G
H	0	6	6	5	3	0	6	6	5	1	7	1	3	2	3	3	2	6	3	3	1	4	5	1	0	4	3	3	2	4	1	0	3	1	2	5	2	2	2	6	3	4	2	5	2	1	0	0	6	1	8	0.365	0.251	1.72E-05	*	H
I	3	5	4	6	1	6	2	7	6	5	2	7	4	4	9	10	2	4	4	5	3	8	4	6	5	8	5	4	7	4	4	8	1	4	3	3	1	11	2	6	2	6	8	8	1	11	2	3	3	2	9	0.531	0.290	1.67E-01	n.s.	I
K	7	2	3	4	10	13	5	6	5	9	6	5	2	14	8	6	6	10	11	5	6	3	12	8	4	2	5	7	4	2	3	5	6	3	7	6	4	5	10	7	10	2	14	9	5	8	3	11	6	5	13	0.491	0.247	1.62E-03	n.s.	K
L	13	17	14	7	14	12	11	19	14	13	7	14	15	14	10	14	10	12	9	15	9	10	17	12	16	9	21	17	12	10	10	10	8	15	9	16	10	12	5	8	18	12	11	11	13	17	9	10	7	14	17	0.720	0.204	–	–	L
M	5	5	2	1	3	0	3	0	2	1	4	1	4	1	4	2	2	6	3	0	3	1	0	1	5	9	2	0	0	1	1	0	0	1	3	3	2	2	4	1	2	5	10	2	5	1	4	1	2	4	4	0.620	0.549	–	–	M
N	2	4	1	4	3	0	4	3	3	2	2	3	4	2	3	0	3	3	3	2	2	7	4	2	4	4	3	3	0	3	9	4	1	4	7	4	0	6	4	5	1	4	2	2	2	0	3	2	3	4	17	0.176	0.107	5.55E-08	*	N
P	10	9	13	8	10	7	8	10	12	11	8	5	11	6	9	11	9	8	12	6	9	13	18	6	14	7	8	9	7	4	3	9	10	9	7	7	8	6	9	4	11	9	8	11	14	6	5	16	10	14	6	1.513	0.516	–	–	P
Q	12	9	4	3	10	6	4	3	6	9	8	4	9	10	4	3	4	6	6	5	1	7	6	5	5	8	11	1	8	6	5	12	6	7	5	7	7	11	5	3	3	4	6	9	3	7	4	7	5	4	9	0.673	0.297	–	–	Q
R	8	8	4	5	9	5	5	4	9	3	6	5	5	6	6	5	3	6	5	8	12	12	12	4	8	4	7	3	6	2	8	6	3	8	3	8	4	5	6	10	6	14	5	6	2	8	11	7	7	5	6	1.057	0.456	–	–	R
S	18	17	15	23	19	17	16	12	16	22	6	20	13	13	16	25	21	13	12	17	8	9	12	13	13	11	13	19	5	7	22	11	15	15	19	13	13	11	17	15	19	13	15	16	16	13	14	18	11	15	18	0.824	0.236	–	–	S
T	3	13	4	10	6	8	6	9	4	9	7	20	7	5	7	6	5	7	6	5	6	4	5	6	8	8	10	7	5	17	9	4	12	9	3	9	13	6	12	7	4	6	6	9	10	7	6	7	12	11	11	0.700	0.310	–	–	T
V	10	9	7	8	13	5	8	10	9	7	7	5	3	8	4	5	10	7	8	11	13	9	10	8	12	8	5	8	2	8	3	7	4	5	3	3	5	3	4	3	4	5	5	8	4	4	10	6	6	9	15	0.451	0.188	2.30E-05	*	V
W	0	0	3	3	6	41	4	2	3	1	0	3	4	0	3	0	1	1	1	2	5	6	0	3	1	1	0	3	4	2	2	3	1	7	5	3	2	2	0	3	1	2	1	1	2	3	2	2	3	6	6	0.513	0.958	7.74E-06	*	W
Y	4	5	4	2	1	1	3	1	2	3	2	1	3	3	3	2	5	7	6	2	6	5	7	7	1	1	4	9	2	5	4	2	1	6	4	5	3	1	1	7	6	2	2	3	3	6	5	2	4	4	3	1.187	0.684	–	–	Y
total	130	144	133	129	145	169	114	137	128	123	110	133	120	130	138	120	121	125	124	129	115	131	171	118	122	121	126	131	108	102	114	107	98	133	114	134	97	119	115	112	132	122	127	125	124	128	99	129	124	129	203	0.614	0.071			total

Additional file 5. Continued.

MOUSE x-x-T-x-I

position -4

	C50	C49	C48	C47	C46	C45	C44	C43	C42	C41	C40	C39	C38	C37	C36	C35	C34	C33	C32	C31	C30	C29	C28	C27	C26	C25	C24	C23	C22	C21	C20	C19	C18	C17	C16	C15	C14	C13	C12	C11	C10	C9	C8	C7	C6	C5	C4	C3	C2	C1	C0	C-index	SD	*P-value*	mark	
A	2	4	0	1	2	3	6	4	2	6	1	3	4	5	2	4	3	2	3	1	4	1	1	4	2	2	4	4	4	2	1	1	4	0	0	1	3	1	11	3	0	3	7	7	0	6	3	2	2	2	5	0.572	0.431	1.13E-01	n.s.	A
C	3	0	1	2	2	0	2	1	2	2	1	2	2	2	1	3	2	2	0	2	1	1	1	0	3	1	2	1	2	0	0	1	2	0	1	1	1	0	1	3	0	2	1	0	3	0	1	3	0	1	3	0.433	0.325	3.32E-03	n.s.	C
D	6	1	2	6	0	1	1	1	2	1	1	2	4	0	3	3	5	0	1	2	2	3	4	4	3	2	2	3	0	2	3	2	0	6	5	2	1	3	0	1	3	1	0	0	2	0	1	0	2	1	6	0.333	0.280	5.99E-08	*	D
E	2	2	5	9	2	2	5	0	3	3	4	2	1	2	2	2	7	1	0	4	6	2	1	1	2	1	0	0	1	4	4	0	0	1	5	5	0	1	4	3	7	3	2	5	0	3	3	5	1	3	4	0.655	0.527	7.57E-01	n.s.	E
F	0	2	5	3	3	1	1	4	6	1	4	9	21	6	5	10	9	20	13	25	9	4	12	11	1	4	2	1	2	24	4	4	1	3	0	3	6	1	0	1	3	2	5	7	1	1	2	2	3	0	3	1.780	2.025	–	–	F
G	3	1	4	7	3	10	2	3	2	3	5	2	3	5	6	2	1	4	3	5	4	1	7	3	2	3	3	0	7	1	4	2	3	2	4	6	2	5	2	4	5	2	1	4	2	3	2	8	3	4	0	–	–	–	–	G
H	0	3	0	0	2	0	1	1	0	2	0	0	3	2	0	0	2	5	1	0	1	1	0	3	4	4	0	0	0	2	2	0	0	2	0	1	0	0	0	1	0	1	0	2	1	0	0	0	2	2	3	0.340	0.429	5.66E-05	*	H
I	3	2	7	1	1	2	3	4	6	2	5	7	7	1	5	4	3	2	2	2	6	1	3	8	3	1	6	2	4	3	4	3	4	4	5	1	3	4	0	5	3	4	1	2	3	4	6	4	1	0	7	0.477	0.279	2.69E-04	n.s.	I
K	5	8	3	1	5	0	1	3	7	2	3	6	4	2	7	4	3	2	2	3	6	2	6	9	3	2	3	3	1	3	2	1	0	1	4	0	2	1	4	2	1	3	3	6	10	6	8	2	5	5	5	0.700	0.482	–	–	K
L	7	9	6	5	6	5	4	5	9	5	6	9	6	6	7	9	5	10	6	5	9	2	4	11	2	5	10	8	4	7	4	1	2	3	6	3	8	6	3	7	2	13	7	3	5	7	5	3	8	3	5	1.164	0.523	–	–	L
M	2	1	1	2	1	1	0	3	1	2	0	0	0	1	0	3	0	0	1	6	1	2	2	0	3	3	3	4	1	1	0	3	1	1	1	0	1	1	2	0	0	1	1	2	0	1	1	0	3	2	2	0.660	0.634	1.15E-01	n.s.	M
N	2	1	1	0	2	5	4	3	3	0	3	5	4	0	7	1	4	11	3	2	9	3	4	1	8	8	1	2	2	32	0	4	0	3	2	8	2	2	5	0	1	2	0	1	3	1	1	0	4	2	2	1.720	2.431	–	–	N
P	4	2	1	1	4	6	3	6	2	5	7	6	6	3	0	0	4	4	5	3	4	8	4	4	1	4	1	6	5	0	1	3	2	3	4	4	2	3	5	5	0	1	2	2	0	2	3	6	3	2	4	0.810	0.504	–	–	P
Q	1	2	2	4	5	1	2	2	2	1	3	3	2	1	1	2	1	0	1	0	5	2	6	2	3	3	1	1	2	5	2	1	2	0	2	3	3	1	2	0	4	2	3	0	1	1	1	2	2	3	6	0.337	0.230	5.56E-08	*	Q
R	1	2	2	4	1	1	2	1	1	2	2	3	2	2	6	7	3	1	1	3	2	5	0	8	2	1	1	2	3	1	3	5	3	4	1	3	2	3	2	1	2	3	5	2	5	2	1	3	6	1	6	0.430	0.290	6.87E-07	*	R
S	3	5	5	4	6	4	4	3	1	4	1	6	4	4	4	6	5	2	6	5	4	6	2	3	2	3	0	3	2	0	14	3	2	3	1	7	5	4	4	8	3	2	2	2	5	4	4	3	6	4	9	0.429	0.253	7.94E-08	*	S
T	4	1	4	3	5	3	3	3	4	4	2	2	5	4	2	3	4	0	7	5	3	7	2	5	3	5	4	4	2	5	5	2	3	7	0	5	4	4	7	8	6	3	2	4	2	2	3	0	4	5	4	0.920	0.456	–	–	T
V	1	4	2	3	4	1	2	3	3	5	9	5	4	1	7	2	4	2	12	9	10	4	19	4	4	2	10	5	0	3	3	7	7	5	4	6	8	1	4	5	0	4	5	3	3	1	11	5	3	3	5	0.928	0.700	–	–	V
W	0	1	1	1	0	1	1	0	0	1	1	0	1	0	0	0	0	1	0	0	0	0	0	10	0	2	0	1	0	0	0	1	0	1	0	0	1	0	0	1	1	1	0	1	0	0	0	4	1	0	4	0.170	0.383	5.55E-08	*	W
Y	2	2	2	1	2	3	1	3	2	3	1	0	3	3	3	2	2	2	2	3	3	6	3	2	7	0	1	3	1	1	0	2	2	1	0	2	0	1	1	6	2	1	1	1	1	2	2	1	0	2	3	0.647	0.488	9.90E-01	n.s.	Y
total	51	53	54	58	56	50	48	53	58	54	59	72	86	50	68	67	67	71	69	85	89	61	81	93	58	56	54	53	43	96	56	46	38	50	45	61	54	42	57	64	43	54	48	54	47	46	58	53	59	45	86	0.682	0.157			total

position -3

	C50	C49	C48	C47	C46	C45	C44	C43	C42	C41	C40	C39	C38	C37	C36	C35	C34	C33	C32	C31	C30	C29	C28	C27	C26	C25	C24	C23	C22	C21	C20	C19	C18	C17	C16	C15	C14	C13	C12	C11	C10	C9	C8	C7	C6	C5	C4	C3	C2	C1	C0	C-index	SD	*P-value*	mark	
A	1	3	4	0	2	4	4	0	4	5	3	5	3	3	4	5	6	2	3	7	2	4	3	2	6	5	4	1	3	20	4	3	0	2	5	2	1	5	1	3	3	3	4	4	3	0	3	2	4	0	6	0.567	0.487	3.30E-02	n.s.	A
C	2	0	1	0	4	1	0	2	2	1	1	2	2	2	1	1	0	5	2	2	3	2	2	3	2	1	0	0	0	2	3	2	0	0	1	2	1	1	2	4	1	2	2	1	1	0	3	1	0	2	1	1.500	1.165	–	–	C
D	0	4	2	3	0	2	0	2	3	2	2	4	4	5	1	2	4	3	1	3	4	2	2	4	1	5	2	0	4	4	4	2	6	2	2	6	2	3	4	3	1	1	2	0	2	4	1	3	3	2	2	1.280	0.757	–	–	D
E	3	5	4	7	1	2	2	2	5	8	3	2	2	1	2	5	3	0	3	1	16	1	4	2	1	0	0	6	1	4	3	5	3	5	2	1	1	2	3	4	3	4	3	3	3	5	2	6	6	4	15	0.219	0.174	5.55E-08	*	E
F	4	4	1	0	3	2	0	4	4	1	3	4	10	2	2	6	2	3	11	2	4	0	3	2	2	0	1	4	2	5	2	0	2	1	0	2	10	1	2	3	5	2	1	3	5	6	2	2	1	2	1	2.860	2.458	–	–	F
G	4	5	1	3	0	3	2	3	3	1	7	9	2	2	3	3	3	2	4	3	1	6	2	6	5	4	5	6	4	0	1	3	1	4	3	3	4	6	3	2	1	2	1	3	4	2	6	3	2	3	7	0.454	0.266	5.04E-06	*	G
H	1	0	1	2	1	3	1	2	2	1	1	1	3	2	2	0	1	2	0	1	3	2	3	1	0	1	1	2	0	0	0	1	0	0	1	2	1	1	1	5	2	1	0	0	0	0	3	1	4	3	2	0.660	0.584	2.32E-01	n.s.	H
I	0	6	2	4	5	3	4	1	0	1	2	3	4	2	3	2	5	3	5	2	3	3	5	3	3	2	3	2	2	1	6	3	3	7	0	4	1	2	2	0	0	1	1	5	5	2	4	2	2	1	2	1.350	0.859	–	–	I
K	7	2	2	7	5	5	5	0	3	3	1	4	4	7	8	8	3	1	2	4	12	4	1	5	0	2	8	1	5	1	2	0	1	0	3	5	4	4	6	6	3	5	3	1	0	3	2	5	4	1	8	0.445	0.324	5.22E-05	*	K
L	3	4	7	2	7	3	6	6	4	3	5	9	17	2	8	2	10	11	6	7	6	9	12	9	10	5	3	4	0	37	5	5	4	2	3	0	5	4	3	6	2	6	4	6	5	6	5	5	6	3	9	0.671	0.606	2.83E-01	n.s.	L
M	0	0	4	2	0	2	2	1	3	2	5	2	0	0	2	4	2	0	2	1	2	0	0	0	0	1	1	0	0	0	2	1	0	1	0	1	0	1	1	0	0	1	0	1	1	1	2	2	2	3	3	0.387	0.406	2.35E-04	n.s.	M
N	2	6	0	7	3	1	5	3	5	1	1	2	5	3	3	7	5	3	1	6	3	1	11	5	1	1	3	5	2	0	2	3	0	1	2	4	4	4	6	5	2	1	3	0	3	2	2	4	1	1	4	0.755	0.557	–	–	N
P	0	3	2	3	2	2	1	2	3	4	2	4	3	2	3	1	2	3	5	2	1	2	2	7	3	0	4	4	0	2	1	0	3	2	3	2	2	3	2	4	6	3	2	2	4	0	1	0	3	4	2	1.210	0.750	–	–	P
Q	3	3	2	2	1	0	3	5	1	3	3	1	0	3	3	0	2	4	2	1	3	0	2	5	1	4	1	2	7	7	6	1	1	1	3	1	0	0	1	1	1	3	0	1	1	2	4	4	1	5	6	0.370	0.302	2.45E-07	*	Q
R	8	2	0	1	3	5	1	5	3	7	2	3	8	1	4	1	3	2	2	6	0	0	2	4	4	1	6	1	0	2	2	5	1	5	4	3	4	0	6	2	3	5	6	4	2	0	5	5	6	3	4	0.790	0.547	–	–	R
S	1	2	5	2	7	2	5	4	4	4	5	4	0	2	4	1	3	5	6	6	6	4	4	4	4	7	4	8	6	4	2	2	3	4	4	3	4	1	7	6	3	2	5	7	2	2	8	2	4	0	4	0.970	0.499	–	–	S
T	4	1	2	2	5	2	2	0	1	2	4	1	5	3	5	1	5	6	1	1	3	10	4	15	10	5	2	2	2	1	3	5	2	3	1	9	4	1	3	1	2	4	7	4	1	3	1	2	0	1	2	1.640	1.446	–	–	T
V	4	3	8	8	3	4	2	7	4	4	7	3	6	4	1	3	3	1	2	3	7	4	5	5	2	7	6	5	4	4	5	4	2	5	4	7	4	2	4	2	2	5	3	5	4	7	2	0	3	5	5	0.816	0.379	–	–	V
W	2	0	1	0	0	1	1	0	0	1	0	2	1	0	1	0	0	0	0	0	1	1	0	1	2	2	0	0	0	1	1	1	1	2	1	2	1	0	0	5	0	2	0	1	0	0	2	0	3	2	1	0.840	1.017	–	–	W
Y	2	0	5	3	4	3	2	4	4	0	2	7	7	4	8	15	5	15	11	27	9	6	14	10	1	3	0	0	1	1	2	0	5	3	3	2	1	1	0	2	3	1	1	3	1	1	0	4	4	0	2	2.100	2.535	–	–	Y
total	51	53	54	58	56	50	48	53	58	54	59	72	86	50	68	67	67	71	69	85	89	61	81	93	58	56	54	53	43	96	56	46	38	50	45	61	54	42	57	64	43	54	48	54	47	46	58	53	59	45	86	0.682	0.157			total

position -1

	C50	C49	C48	C47	C46	C45	C44	C43	C42	C41	C40	C39	C38	C37	C36	C35	C34	C33	C32	C31	C30	C29	C28	C27	C26	C25	C24	C23	C22	C21	C20	C19	C18	C17	C16	C15	C14	C13	C12	C11	C10	C9	C8	C7	C6	C5	C4	C3	C2	C1	C0	C-index	SD	*P-value*	mark	
A	0	1	3	5	2	4	7	5	8	5	0	3	5	4	4	2	8	16	4	5	5	5	4	14	8	6	4	4	0	25	3	4	4	2	4	3	3	3	6	1	0	6	4	5	3	3	4	5	4	1	4	1.170	1.042	–	–	A
C	5	0	0	1	0	0	1	1	0	1	1	2	2	0	2	0	0	0	1	1	3	1	0	1	0	2	0	2	2	3	3	0	1	2	1	0	1	1	2	3	1	0	3	1	3	1	1	1	5	1	3	0.420	0.414	1.69E-04	n.s.	C
D	1	5	2	1	4	3	1	2	5	2	2	3	4	2	1	1	2	2	1	2	1	3	5	3	1	2	2	6	0	10	2	1	4	1	3	6	1	2	7	4	4	4	6	2	3	3	0	2	2	1	4	0.685	0.489	–	–	D
E	2	3	4	5	1	3	5	1	0	7	6	4	8	1	4	2	3	0	3	4	4	3	4	2	3	1	4	2	4	2	2	1	2	4	2	6	4	1	2	4	7	0	0	4	1	2	4	5	3	2	11	0.275	0.172	5.55E-08	*	E
F	1	2	1	3	7	3	1	0	0	3	2	0	3	6	4	0	2	6	6	2	2	1	5	4	4	2	3	3	1	2	2	1	4	6	5	1	2	4	1	2	2	6	4	4	1	1	1	2	0	2	4	0.650	0.466	5.42E-01	n.s.	F
G	1	7	7	6	6	3	2	4	3	1	2	6	1	2	6	8	5	4	3	3	3	6	5	1	4	1	8	2	4	0	5	2	3	3	2	1	0	2	1	3	0	3	1	3	1	1	0	0	3	0	5	0.592	0.446	2.19E-01	n.s.	G
H	1	2	1	3	1	0	3	4	2	2	6	1	5	2	0	5	1	1	1	2	2	2	1	3	2	5	3	4	2	2	3	2	1	3	0	1	1	1	3	1	4	3	0	2	0	2	3	0	3	1	4	0.515	0.362	1.02E-02	n.s.	H
I	1	1	5	3	3	3	2	3	4	3	2	3	3	2	4	6	5	2	2	15	4	7	6	2	3	5	3	0	5	4	2	2	1	1	0	3	3	1	2	2	3	3	2	1	4	2	1	2	1	0	6	0.490	0.392	2.29E-05	*	I
K	1	2	0	3	2	2	3	2	2	5	4	7	3	2	4	1	0	0	1	3	3	1	0	5	1	2	6	3	4	2	2	4	1	3	3	2	2	2	6	12	2	1	1	5	4	7	7	5	4	5	4	0.760	0.569	–	–	K
L	9	1	6	10	5	4	6	4	9	1	3	10	5	5	7	12	9	4	15	7	14	3	17	6	6	7	4	7	2	6	8	4	2	1	3	7	7	2	5	7	1	7	3	3	7	3	5	1	12	2	5	1.176	0.744	–	–	L
M	3	1	0	0	0	3	0	3	1	2	1	1	0	0	0	5	2	5	0	4	2	0	5	4	0	0	0	1	0	1	1	1	1	1	0	2	0	0	0	0	0	0	2	2	0	3	1	2	0	1	1	1.220	1.489	–	–	M
N	0	4	4	0	2	0	1	1	1	0	4	3	6	2	7	1	2	2	0	4	1	4	3	4	4	5	1	2	0	1	0	2	0	0	4	2	2	2	2	1	1	0	1	2	2	3	2	0	1	1	3	0.647	0.565	9.11E-01	n.s.	N
P	1	0	2	4	4	2	3	2	6	2	7	2	3	0	3	3	1	3	2	5	3	4	3	6	4	2	6	1	2	4	4	1	2	5	4	5	3	2	2	1	4	2	2	0	2	3	4	4	1	1	4	0.710	0.411	–	–	P
Q	2	4	2	1	1	4	1	2	2	1	0	5	2	3	3	2	4	0	1	3	10	0	2	5	2	2	2	1	0	0	3	3	1	3	3	2	1	2	6	8	2	4	1	5	3	0	3	1	1	1	8	0.300	0.250	5.55E-08	*	Q
R	7	5	2	0	0	2	0	4	4	5	2	1	4	0	4	2	5	0	2	3	2	3	2	2	4	3	3	2	2	17	1	3	1	4	1	0	8	1	1	4	4	3	4	2	2	3	6	4	3	6	2	1.530	1.368	–	–	R
S	4	4	1	4	2	5	4	8	4	6	0	4	9	6	2	4	7	8	5	3	7	6	1	4	4	6	3	5	3	7	5	6	2	6	1	11	7	5	2	6	2	1	2	2	3	2	3	3	5	6	11	0.393	0.211	5.95E-08	*	S
T	4	3	5	1	5	4	2	2	2	5	4	5	13	7	2	2	3	7	2	3	11	2	2	12	0	2	1	3	6	1	2	3	2	2	5	3	1	1	2	2	2	3	0	5	1	0	1	4	4	6	0	–	–	–	–	T
V	5	1	2	7	8	3	4	4	5	2	7	8	9	5	8	8	4	11	10	15	11	8	13	8	5	2	1	2	3	6	4	3	0	2	2	2	6	5	4	2	1	6	4	3	6	3	10	7	3	7	3	1.767	1.113	–	–	V
W	1	1	1	0	1	0	1	0	0	1	3	1	0	0	3	2	3	0	1	1	0	0	1	0	1	0	0	1	2	2	1	0	2	1	1	2	2	2	1	0	2	2	3	1	0	1	0	3	1	0	1	1.040	0.968	–	–	W
Y	2	6	6	1	2	2	1	1	0	0	3	3	1	1	0	1	1	0	9	0	1	2	2	7	2	1	0	2	1	1	3	3	4	0	1	2	0	3	2	1	1	0	5	2	1	3	2	2	3	1	3	0.653	0.635	4.42E-01	n.s.	Y
total	51	53	54	58	56	50	48	53	58	54	59	72	86	50	68	67	67	71	69	85	89	61	81	93	58	56	54	53	43	96	56	46	38	50	45	61	54	42	57	64	43	54	48	54	47	46	58	53	59	45	86	0.682	0.157			total

Additional file 5. Continued.

MOUSE x-x-T-x-L

position -4

	C50	C49	C48	C47	C46	C45	C44	C43	C42	C41	C40	C39	C38	C37	C36	C35	C34	C33	C32	C31	C30	C29	C28	C27	C26	C25	C24	C23	C22	C21	C20	C19	C18	C17	C16	C15	C14	C13	C12	C11	C10	C9	C8	C7	C6	C5	C4	C3	C2	C1	C0	C-index	SD	*P-value*	mark	
A	12	8	7	9	10	14	13	14	13	15	17	12	4	10	5	8	15	12	9	14	12	13	14	5	8	9	3	9	8	4	8	8	10	10	5	15	4	7	7	4	8	9	6	8	6	13	4	5	9	9	17	0.542	0.212	–	–	A
C	3	3	3	2	1	2	5	2	5	5	1	3	6	1	3	1	3	5	2	1	1	3	3	2	3	3	4	2	3	3	3	4	1	5	4	3	4	3	1	4	3	1	1	1	1	3	2	6	1	1	3	0.907	0.481	–	–	C
D	3	10	5	6	4	4	5	6	4	7	4	3	5	7	3	9	5	3	7	5	3	5	5	8	4	3	11	6	6	6	6	4	5	3	5	7	4	3	2	0	3	2	9	2	5	11	6	4	8	5	17	0.301	0.137	2.38E-07	✻	D
E	6	11	4	5	6	9	4	13	8	3	8	5	9	5	4	10	11	8	6	6	5	5	10	7	19	5	5	5	6	9	6	8	5	7	3	11	9	6	8	4	4	2	5	4	10	6	4	5	5	4	19	0.351	0.160	2.05E-03	n.s.	E
F	7	9	9	4	7	2	7	4	5	18	10	3	4	16	10	8	12	12	10	8	17	9	4	4	4	3	4	11	13	3	2	5	8	10	6	2	6	3	7	8	12	5	2	8	7	6	3	1	1	6	13	0.531	0.313	–	–	F
G	9	9	5	8	10	14	8	3	7	10	12	5	14	7	7	9	10	8	4	7	7	8	6	4	1	9	5	8	8	6	6	11	3	8	4	4	10	8	10	5	4	8	9	5	11	5	7	6	5	5	14	0.517	0.198	–	–	G
H	3	0	2	1	2	14	4	4	1	2	4	7	4	4	2	2	2	2	2	7	5	3	1	5	5	3	2	4	2	4	3	2	1	2	6	2	1	1	2	3	1	2	1	4	2	1	15	9	3	2	13	0.255	0.225	5.70E-08	✻	H
I	6	11	12	9	5	11	5	7	3	10	8	3	8	5	5	3	11	3	9	14	16	12	8	6	11	4	3	5	4	5	9	7	8	8	5	12	4	6	7	9	8	6	2	5	9	1	6	3	8	1	4	1.730	0.846	–	–	I
K	3	5	4	4	9	11	5	3	5	8	7	6	12	7	9	11	10	6	10	2	4	3	4	5	6	3	4	8	7	6	9	3	10	5	2	6	7	4	12	8	10	7	5	9	8	5	7	2	5	7	29	0.219	0.094	5.55E-08	✻	K
L	20	12	22	10	14	11	18	12	11	13	18	8	13	23	9	14	22	12	16	19	19	11	14	21	10	9	13	12	8	10	16	11	21	16	15	14	13	19	17	25	18	15	12	17	10	22	9	14	14	21	38	0.391	0.117	1.98E-01	n.s.	L
M	1	2	2	2	2	2	2	2	2	1	4	3	4	5	1	5	2	7	3	3	3	4	4	1	3	3	1	1	1	2	3	0	2	7	4	1	2	3	3	1	3	2	3	7	2	3	2	2	2	5	5	0.540	0.316	–	–	M
N	5	4	5	2	3	7	4	2	4	4	1	5	9	1	5	5	5	6	1	7	3	6	2	2	5	3	3	5	3	3	4	1	3	6	5	3	5	3	6	4	5	6	10	3	2	1	4	2	7	8	8	0.520	0.260	–	–	N
P	5	11	16	4	12	12	6	9	14	5	8	8	9	5	5	12	8	4	4	8	2	4	6	8	6	11	9	9	9	5	12	15	4	10	9	12	10	7	7	8	7	12	9	7	4	2	15	2	4	10	20	0.400	0.177	9.86E-01	n.s.	P
Q	6	6	6	6	7	7	4	3	6	4	4	4	5	9	7	6	3	4	4	6	4	5	6	8	5	11	9	5	7	5	5	7	3	3	4	3	9	6	4	4	7	9	8	7	1	6	7	8	4	10	11	0.522	0.190	–	–	Q
R	8	2	8	4	3	8	12	9	14	3	2	5	8	6	11	8	6	7	4	4	14	4	4	4	15	9	9	8	11	16	11	7	7	8	9	7	8	5	6	8	6	6	8	8	13	2	9	8	6	6	22	0.340	0.152	3.00E-05	✻	R
S	11	9	12	9	16	15	11	6	11	8	5	11	11	10	8	18	9	8	11	13	9	5	5	14	7	15	15	8	15	13	9	13	5	14	16	12	14	19	14	8	8	27	16	2	13	7	11	13	11	11	32	0.351	0.135	1.71E-03	n.s.	S
T	7	4	12	8	3	9	3	5	7	5	5	9	7	7	10	15	11	7	13	4	6	3	5	7	9	10	11	5	8	8	10	7	9	4	8	6	4	8	9	3	5	4	6	5	10	2	7	5	4	9	19	0.366	0.150	8.59E-02	n.s.	T
V	5	4	9	10	9	2	9	3	6	10	9	18	9	13	19	9	11	15	9	22	24	14	15	12	10	6	22	13	13	9	16	10	6	12	8	14	13	16	10	11	11	6	9	8	9	7	8	7	9	9	16	0.673	0.294	–	–	V
W	1	1	2	0	3	1	2	1	1	3	2	2	0	1	1	2	1	0	1	1	1	2	0	0	1	2	2	4	2	3	3	0	1	1	0	3	5	4	0	0	4	0	2	1	0	3	2	0	3	2	2	0.770	0.641	–	–	W
Y	15	4	6	4	8	1	2	6	6	3	3	3	2	9	7	5	4	8	3	2	3	2	7	4	2	1	3	0	4	6	2	1	1	2	5	2	3	1	2	3	4	5	4	2	4	5	3	8	5	2	7	0.563	0.380	–	–	Y
total	136	125	151	107	134	156	129	114	133	137	132	123	143	151	131	160	161	137	128	153	158	121	123	127	134	122	138	128	138	126	143	124	113	141	123	139	135	132	134	120	131	134	127	113	127	111	131	110	114	133	309	0.427	0.042			total

position -3

	C50	C49	C48	C47	C46	C45	C44	C43	C42	C41	C40	C39	C38	C37	C36	C35	C34	C33	C32	C31	C30	C29	C28	C27	C26	C25	C24	C23	C22	C21	C20	C19	C18	C17	C16	C15	C14	C13	C12	C11	C10	C9	C8	C7	C6	C5	C4	C3	C2	C1	C0	C-index	SD	*P-value*	mark	
A	9	8	10	3	11	6	7	8	5	14	9	7	4	9	4	6	6	5	7	7	6	7	8	4	8	10	10	4	4	14	11	9	3	6	5	11	10	16	10	9	13	7	5	5	9	6	7	11	3	15	15	0.521	0.213	–	–	A
C	4	12	5	3	1	2	2	2	3	3	3	3	1	3	7	1	7	3	6	5	4	0	2	3	3	4	3	3	3	2	2	0	3	6	3	0	1	2	3	5	2	1	4	2	3	6	2	1	0	2	15	0.201	0.143	5.55E-08	✻	C
D	6	2	5	5	12	9	1	2	10	6	4	3	9	13	8	5	8	7	3	7	9	2	5	7	20	5	11	3	9	5	7	8	4	10	8	9	9	5	3	6	12	5	3	3	4	4	5	4	3	6	19	0.336	0.185	3.90E-03	n.s.	D
E	3	10	4	3	10	4	8	9	4	3	5	7	9	8	5	15	5	9	10	9	7	7	5	10	7	10	8	7	8	7	8	4	3	6	7	11	11	5	8	8	2	5	12	3	8	11	9	3	8	6	32	0.221	0.089	5.55E-08	✻	E
F	1	13	10	7	7	3	6	8	3	10	8	11	11	14	4	5	6	6	9	6	6	5	7	6	8	6	0	2	8	5	8	5	4	10	6	6	3	8	4	4	3	4	6	6	5	2	2	3	2	3	6	0.983	0.505	–	–	F
G	16	6	5	6	6	10	9	8	1	9	19	9	6	5	6	14	10	8	4	9	4	9	2	12	5	10	10	7	8	8	12	10	7	5	11	7	7	7	11	3	2	5	11	4	7	9	7	10	0	12	9	0.862	0.406	–	–	G
H	7	3	8	4	2	8	0	1	5	4	2	3	4	2	6	1	4	2	3	2	5	0	4	2	0	5	5	4	2	5	7	5	3	6	5	7	4	1	2	5	0	3	6	4	2	3	7	1	6	5	9	0.411	0.241	1.00E+00	n.s.	H
I	7	3	10	7	8	10	6	5	4	7	6	2	11	6	14	11	8	7	5	12	8	7	14	10	8	6	15	9	10	8	4	6	8	7	7	2	1	5	7	1	4	4	7	5	5	1	7	3	5	15	6	1.160	0.575	–	–	I
K	9	8	5	5	2	11	12	13	14	7	4	15	8	7	3	3	6	14	3	6	11	7	6	11	7	1	6	9	26	7	4	7	6	6	3	10	6	7	9	7	7	27	6	14	6	9	5	4	13	14	26	0.320	0.196	3.24E-05	✻	K
L	7	14	18	6	13	23	21	10	16	16	8	9	12	19	11	15	25	10	13	25	27	19	18	21	11	14	16	17	12	17	21	13	24	18	9	7	13	16	13	14	18	17	8	9	12	13	14	13	8	5	28	0.520	0.189	–	–	L
M	0	3	1	3	4	3	2	2	3	3	2	0	4	3	7	5	10	0	7	3	4	3	4	1	2	6	1	6	6	0	2	1	3	4	0	2	4	2	3	2	3	4	3	1	4	4	4	0	1	6	8	0.378	0.263	3.19E-01	n.s.	M
N	4	4	8	2	6	8	4	3	8	7	7	2	5	6	3	9	4	5	1	2	2	5	6	2	3	4	4	4	2	2	4	4	4	4	1	2	4	6	5	3	7	7	2	6	2	5	4	3	4	2	6	0.703	0.336	–	–	N
P	8	4	13	7	6	6	5	6	6	12	15	5	2	5	12	5	6	7	9	6	17	3	4	7	6	4	7	11	2	7	3	4	11	8	18	5	14	7	7	9	5	5	13	5	6	3	5	5	8	2	14	0.509	0.271	–	–	P
Q	9	4	4	6	6	2	9	8	13	8	6	7	5	3	6	13	9	5	4	5	4	2	5	8	5	3	5	2	8	3	10	6	7	3	7	7	5	3	6	7	8	4	5	1	10	6	3	11	2	6	21	0.280	0.132	6.58E-08	✻	Q
R	10	4	2	12	5	9	13	5	10	3	2	7	13	6	7	8	8	5	6	8	6	9	5	5	7	4	5	5	5	9	8	10	3	10	4	10	7	9	9	10	8	8	4	14	12	6	10	6	15	12	10	0.756	0.316	–	–	R
S	15	4	13	11	12	12	6	6	5	6	10	12	14	4	7	14	11	6	8	8	6	9	4	3	7	12	12	7	13	13	14	13	7	8	14	9	14	9	11	10	9	13	12	3	13	11	16	3	18	4	39	0.247	0.099	5.55E-08	✻	S
T	1	7	10	6	9	10	7	6	8	3	8	5	10	15	7	7	9	4	5	6	9	8	2	6	7	8	6	10	5	2	6	3	6	6	4	7	7	5	6	4	9	7	9	8	3	4	5	14	8	8	18	0.372	0.153	7.84E-02	n.s.	T
V	14	7	16	8	9	13	5	7	8	12	4	8	9	16	9	11	10	16	11	12	7	9	16	6	13	7	11	12	3	9	4	7	2	11	7	10	5	11	11	7	9	4	7	11	5	5	16	6	4	4	22	0.404	0.170	8.87E-01	n.s.	V
W	3	0	3	0	2	1	1	2	1	1	1	2	3	4	0	4	2	2	3	0	1	0	0	1	4	0	2	1	2	0	3	4	1	5	1	4	1	4	3	0	5	1	1	7	6	2	1	3	2	3	2	1.030	0.848	–	–	W
Y	3	9	1	3	3	6	5	3	6	3	9	6	3	3	5	8	7	16	11	15	15	10	6	2	3	3	1	5	2	3	5	5	4	2	3	13	9	4	3	6	5	3	3	2	5	1	2	6	4	3	4	1.315	0.921	–	–	Y
total	136	125	151	107	134	156	129	114	133	137	132	123	143	151	131	160	161	137	128	153	158	121	123	127	134	122	138	128	138	126	143	124	113	141	123	139	135	132	134	120	131	134	127	113	127	111	131	110	114	133	309	0.427	0.042			total

position -1

	C50	C49	C48	C47	C46	C45	C44	C43	C42	C41	C40	C39	C38	C37	C36	C35	C34	C33	C32	C31	C30	C29	C28	C27	C26	C25	C24	C23	C22	C21	C20	C19	C18	C17	C16	C15	C14	C13	C12	C11	C10	C9	C8	C7	C6	C5	C4	C3	C2	C1	C0	C-index	SD	*P-value*	mark	
A	9	7	16	8	9	9	11	10	8	6	7	5	7	14	9	8	8	10	3	7	8	6	16	10	15	9	4	12	4	9	12	11	15	13	6	9	11	17	17	10	9	11	11	9	9	8	8	8	3	7	23	0.407	0.148	2.85E-01	n.s.	A
C	4	3	2	2	4	1	3	4	6	9	2	8	1	7	0	3	2	3	2	2	3	3	0	1	1	2	2	3	4	3	4	1	2	4	2	5	1	6	3	4	3	2	3	1	5	2	4	5	2	0	3	0.993	0.644	–	–	C
D	2	5	4	6	7	5	4	2	4	4	6	6	6	7	9	13	6	7	2	6	4	4	1	8	4	7	6	5	3	7	3	5	3	9	5	5	3	7	5	3	6	8	5	5	4	2	5	6	10	7	16	0.333	0.141	1.09E-04	n.s.	D
E	5	1	8	7	6	10	1	5	10	7	5	2	12	4	5	8	12	5	6	8	9	5	6	4	4	2	5	14	6	4	7	10	4	14	7	10	4	8	5	6	10	3	5	2	6	5	6	3	4	5	28	0.221	0.110	5.55E-08	✻	E
F	9	3	3	4	6	7	7	4	4	8	6	4	8	2	4	12	12	3	5	3	6	11	5	5	9	4	4	7	5	6	9	10	6	6	13	7	10	5	8	9	7	2	3	9	9	5	3	4	3	9	15	0.417	0.188	9.78E-01	n.s.	F
G	8	4	12	5	8	12	9	6	8	4	2	6	3	9	8	6	9	14	4	13	4	8	7	9	2	5	16	7	9	9	14	4	5	10	9	9	15	7	7	7	2	9	5	7	5	6	7	6	6	5	15	0.495	0.221	–	–	G
H	4	5	3	2	4	11	2	2	5	3	4	3	2	5	1	5	3	2	3	0	2	2	3	3	2	4	2	2	3	6	5	3	8	1	5	1	5	2	8	3	4	5	4	9	6	3	14	4	5	6	19	0.209	0.136	5.55E-08	✻	H
I	3	4	8	6	7	3	6	8	7	5	3	6	17	20	5	11	9	7	14	10	15	7	7	6	5	7	9	4	10	3	6	2	3	11	2	7	5	1	9	9	7	6	7	4	4	1	3	2	6	7	12	0.557	0.325	–	–	I
K	4	7	2	5	7	14	10	10	5	7	6	6	5	5	7	6	6	6	2	7	9	6	5	2	9	6	2	4	5	9	5	6	7	5	5	15	5	7	6	4	7	5	1	3	9	4	3	6	3	16	20	0.306	0.154	1.81E-07	✻	K
L	13	14	12	13	12	11	17	10	15	20	14	12	19	15	10	16	19	20	17	15	12	14	12	15	18	9	14	7	21	13	20	9	14	14	17	10	11	19	12	13	18	17	14	14	14	17	13	12	21	11	32	0.449	0.106	–	–	L
M	1	3	2	1	3	2	4	2	3	3	4	3	1	1	1	2	4	2	2	2	3	1	2	2	3	2	2	2	5	2	2	3	2	4	3	3	4	2	3	2	2	2	1	0	0	2	1	2	1	1	2	1.100	0.535	–	–	M
N	4	5	5	7	3	5	1	5	4	4	3	7	4	4	8	2	2	1	1	6	2	1	3	5	17	2	1	4	5	9	1	2	3	3	3	6	12	7	2	1	5	3	4	3	5	2	5	11	2	9	5	0.876	0.631	–	–	N
P	7	7	11	6	6	10	7	8	10	8	11	9	7	11	5	10	8	11	20	33	27	11	18	18	11	13	24	17	10	10	9	12	8	6	7	6	5	5	7	5	11	9	10	9	5	4	6	4	12	5	10	1.018	0.587	–	–	P
Q	5	8	7	6	8	5	5	5	3	4	10	9	5	4	7	1	7	4	2	2	4	6	5	9	3	7	6	3	10	3	4	5	1	4	5	5	6	3	2	6	2	4	10	7	5	5	4	3	3	2	10	0.498	0.232	–	–	Q
R	7	3	8	5	6	8	13	3	9	8	12	5	8	7	14	7	7	6	7	5	8	4	5	6	1	12	7	7	10	7	4	7	4	6	5	9	8	7	5	6	10	5	7	6	6	15	8	6	5	6	29	0.241	0.094	5.55E-08	✻	R
S	14	15	9	7	14	11	13	8	14	13	9	11	13	17	14	16	12	14	12	10	19	13	11	8	12	11	14	8	8	11	10	11	14	10	11	14	8	7	16	14	9	25	10	7	18	6	20	4	12	21	29	0.419	0.140	9.96E-01	n.s.	S
T	10	10	8	3	10	9	3	7	3	8	9	9	4	9	6	17	9	5	11	9	5	2	4	8	7	6	3	6	5	3	10	8	5	11	7	4	4	8	5	7	6	6	10	6	3	8	12	7	3	7	16	0.431	0.185	–	–	T
V	15	11	15	8	5	12	4	14	10	10	12	7	13	8	9	11	17	11	10	11	14	11	10	7	8	11	13	7	9	10	10	13	5	7	6	5	9	7	8	9	9	8	11	7	8	8	1	13	12	6	17	0.559	0.185	–	–	V
W	3	1	2	2	1	8	2	1	1	2	3	1	5	1	2	3	3	2	3	1	3	3	0	0	0	0	1	4	2	0	4	1	3	1	2	4	6	3	0	0	3	1	3	2	2	1	1	3	1	1	3	0.680	0.539	–	–	W
Y	9	9	14	4	8	3	7	0	4	4	4	4	3	1	7	3	6	4	2	3	1	3	3	1	3	3	3	5	4	2	4	1	1	2	3	5	3	4	6	2	1	3	3	3	4	7	7	1	0	2	5	0.756	0.526	–	–	Y
total	136	125	151	107	134	156	129	114	133	137	132	123	143	151	131	160	161	137	128	153	158	121	123	127	134	122	138	128	138	126	143	124	113	141	123	139	135	132	134	120	131	134	127	113	127	111	131	110	114	133	309	0.427	0.042			total

Additional file 5. Continued.

MOUSE x-x-T-x-V

position -4

	C50	C49	C48	C47	C46	C45	C44	C43	C42	C41	C40	C39	C38	C37	C36	C35	C34	C33	C32	C31	C30	C29	C28	C27	C26	C25	C24	C23	C22	C21	C20	C19	C18	C17	C16	C15	C14	C13	C12	C11	C10	C9	C8	C7	C6	C5	C4	C3	C2	C1	C0	C-index	SD	*P-value*	mark	
A	4	2	4	5	3	4	18	4	7	9	18	6	8	3	7	3	4	5	3	7	6	7	0	2	8	8	5	3	5	3	8	2	3	4	7	2	7	6	14	4	6	8	4	2	4	7	5	3	4	4	4	1.375	0.884	–	–	A
C	2	3	1	1	1	0	0	0	0	0	1	0	1	1	0	0	2	2	1	1	1	2	2	2	1	2	2	1	0	0	1	4	3	0	2	3	5	1	6	3	1	4	4	1	0	4	1	2	2	2	3	0.527	0.477	–	–	C
D	1	3	9	7	7	1	1	2	8	4	5	1	3	3	2	1	4	6	3	0	1	4	7	6	5	2	4	5	3	4	2	5	5	2	1	0	2	7	4	4	3	2	7	2	1	3	2	1	4	1	3	1.133	0.747	–	–	D
E	5	3	4	1	2	3	3	3	4	4	8	2	4	2	5	3	6	2	6	4	3	4	4	5	4	5	9	0	2	1	0	4	3	4	4	2	1	5	7	3	1	4	3	3	4	2	2	4	3	6	15	0.235	0.123	5.55E-08	*	E
F	8	2	3	4	6	2	5	3	6	4	5	5	9	8	3	24	16	23	17	39	50	41	14	15	9	10	4	3	0	14	5	4	1	1	2	6	0	2	4	1	1	5	4	2	4	1	10	2	1	1	10	0.818	1.062	–	–	F
G	1	4	4	6	8	4	5	1	3	5	6	7	7	4	3	9	10	6	11	7	7	9	5	4	3	6	5	3	3	5	4	2	3	3	4	8	5	5	13	3	7	10	4	5	8	17	8	8	7	2	10	0.574	0.307	–	–	G
H	1	1	5	0	0	2	6	2	6	2	6	0	1	0	1	3	2	3	1	3	1	4	0	2	2	5	2	3	5	0	2	2	4	1	0	1	2	2	0	1	2	2	1	2	0	2	2	1	3	1	5	0.400	0.333	5.84E-02	n.s.	H
I	6	3	5	4	1	14	2	2	10	4	7	8	6	4	4	9	5	5	5	8	4	4	4	27	2	9	5	4	6	2	4	5	2	2	4	10	9	4	9	0	2	1	2	3	4	4	5	6	10	1	9	0.591	0.474	–	–	I
K	3	2	5	5	2	4	3	4	2	6	3	5	10	7	4	8	32	4	7	4	5	3	3	1	2	2	3	4	6	5	6	1	1	7	2	2	0	3	3	4	2	5	5	5	6	6	6	2	3	4	13	0.349	0.342	1.46E-06	*	K
L	8	5	12	2	4	9	3	6	8	5	17	15	6	7	7	11	12	20	9	17	5	10	7	10	8	6	9	10	4	4	4	8	5	10	10	9	4	11	16	9	9	13	8	9	6	4	5	5	8	8	13	0.642	0.300	–	–	L
M	0	2	3	1	2	2	1	2	2	6	5	0	2	5	1	2	5	8	8	27	9	3	14	4	0	4	3	3	0	2	0	0	2	1	1	1	5	0	4	2	4	1	1	1	0	2	0	0	1	3	9	0.344	0.490	2.18E-05	*	M
N	6	2	8	4	2	6	7	5	3	7	2	2	5	3	7	4	5	1	3	2	5	2	4	8	2	5	3	0	0	2	2	3	3	0	5	6	3	1	7	1	3	4	1	2	4	2	2	1	3	0	9	0.373	0.242	1.96E-02	n.s.	N
P	5	4	5	7	2	7	4	4	9	3	4	3	8	7	4	7	5	3	3	5	3	8	6	6	4	7	4	11	10	6	3	3	4	6	3	7	5	7	6	1	7	3	4	7	4	8	6	4	4	5	13	0.402	0.162	1.95E-02	n.s.	P
Q	4	2	6	3	4	2	1	5	6	1	3	12	1	4	3	3	3	3	2	4	2	3	2	2	3	6	4	3	1	3	4	4	0	3	2	3	4	3	3	3	3	3	3	3	3	0	1	2	3	4	12	0.258	0.154	5.55E-08	*	Q
R	1	4	1	6	4	4	5	6	6	1	5	7	2	3	6	8	2	3	3	3	7	3	3	6	0	3	2	4	6	3	2	4	5	4	3	9	6	7	3	5	5	3	4	2	3	1	3	8	6	6	15	0.275	0.138	5.93E-08	*	R
S	13	9	14	7	9	13	8	8	7	8	6	13	14	3	11	9	5	8	6	5	6	13	5	4	5	10	8	10	8	5	7	5	5	4	7	8	5	13	2	10	5	8	6	10	8	15	4	3	6	6	16	0.484	0.202	1.00E+00	n.s.	S
T	6	3	6	4	2	6	5	5	2	5	5	4	6	3	2	6	6	7	6	8	2	5	4	3	3	10	7	7	4	2	7	4	3	9	5	2	5	12	3	6	3	3	6	3	4	9	3	2	7	8	6	0.827	0.385	–	–	T
V	4	7	7	7	8	6	6	6	4	4	4	5	3	5	3	13	4	12	9	10	8	7	6	5	4	3	12	6	3	6	18	5	2	4	12	11	30	15	3	11	4	55	1	11	2	6	51	4	2	4	14	0.626	0.741	–	–	V
W	0	0	2	0	2	2	1	0	1	2	0	2	0	1	0	1	4	1	1	2	0	0	0	0	0	1	0	0	2	1	0	2	2	2	1	1	3	3	0	0	0	0	2	0	1	1	1	1	1	2	1	0.980	1.000	–	–	W
Y	3	1	1	2	5	2	5	5	5	2	3	1	5	2	2	3	1	2	3	0	3	9	0	1	3	3	0	2	2	4	1	3	0	7	3	1	3	2	2	2	2	0	5	1	2	0	2	0	2	0	7	0.337	0.270	4.30E-04	n.s.	Y
total	81	62	105	76	74	93	89	73	99	82	113	98	101	75	75	127	133	124	107	156	128	141	90	113	68	107	91	82	70	72	80	70	56	74	78	92	104	109	109	73	70	134	75	74	68	94	119	59	80	68	187	0.491	0.126			total

position -3

	C50	C49	C48	C47	C46	C45	C44	C43	C42	C41	C40	C39	C38	C37	C36	C35	C34	C33	C32	C31	C30	C29	C28	C27	C26	C25	C24	C23	C22	C21	C20	C19	C18	C17	C16	C15	C14	C13	C12	C11	C10	C9	C8	C7	C6	C5	C4	C3	C2	C1	C0	C-index	SD	*P-value*	mark	
A	4	5	10	6	8	3	6	8	11	9	13	4	5	1	3	7	6	4	6	7	6	3	3	6	3	9	5	8	3	17	8	5	4	3	2	8	4	4	4	2	3	4	4	3	3	8	1	6	7	6	7	0.794	0.437	–	–	A
C	1	0	1	0	2	4	1	1	4	3	0	6	2	0	1	3	1	4	2	4	1	2	1	3	1	3	0	0	2	1	1	1	3	0	5	1	3	1	1	3	4	0	2	2	3	1	6	0	0	1	8	0.230	0.199	6.89E-08	*	C
D	2	4	4	1	2	3	5	1	4	3	4	1	4	6	4	3	2	1	3	4	3	5	4	3	2	6	4	1	6	6	5	0	0	1	4	1	2	2	3	5	3	3	4	1	4	3	1	2	3	3	5	0.604	0.319	–	–	D
E	2	3	4	2	8	5	1	5	5	3	3	6	7	5	5	3	4	6	5	1	3	5	2	4	2	6	8	5	4	1	2	5	1	3	3	5	5	2	1	6	1	5	4	3	1	1	3	2	1	5	32	0.114	0.060	5.55E-08	*	E
F	3	2	6	1	2	0	0	2	4	5	3	2	4	4	6	8	7	1	3	3	4	6	2	3	1	4	4	1	3	1	3	5	0	1	1	5	14	8	4	4	1	2	4	4	1	5	2	2	2	0	2	1.630	1.273	–	–	F
G	8	4	8	7	4	18	4	4	11	5	7	8	4	3	3	3	26	3	7	4	3	10	7	1	12	8	10	5	7	5	2	3	2	1	8	7	6	10	17	2	4	5	3	10	4	18	6	10	6	1	17	0.393	0.288	6.62E-03	n.s.	G
H	5	1	1	1	3	3	2	2	2	1	2	1	0	1	1	3	2	4	1	7	1	2	2	4	1	1	3	2	0	1	3	2	0	2	4	4	1	8	1	0	2	2	2	3	3	1	6	0	0	1	7	0.300	0.249	3.48E-06	*	H
I	4	2	6	4	7	4	7	1	2	3	8	4	13	5	2	5	0	4	5	5	4	2	1	4	3	11	4	1	4	1	3	1	6	3	5	1	4	3	2	4	5	5	3	3	6	3	2	1	2	1	8	0.473	0.312	9.74E-01	n.s.	I
K	5	3	3	8	4	4	13	4	3	9	9	11	7	2	5	14	10	3	3	2	5	6	3	3	2	8	5	2	3	2	3	6	4	6	2	3	4	9	15	3	5	3	3	4	5	3	4	5	9	5	6	0.873	0.537	–	–	K
L	10	4	10	5	8	8	14	8	8	3	5	11	6	5	5	12	5	8	9	8	8	11	8	10	8	5	6	8	6	2	5	6	4	14	10	5	7	9	7	3	9	7	9	12	5	9	9	9	7	7	7	1.077	0.384	–	–	L
M	2	1	3	2	4	2	2	0	2	3	2	1	2	0	3	4	1	5	3	2	1	2	1	0	1	0	2	0	1	1	3	1	1	3	1	2	2	1	3	2	1	4	2	1	0	2	2	2	3	4	6	0.310	0.199	7.33E-06	*	M
N	1	7	6	2	3	5	2	2	9	4	6	5	4	1	5	3	6	12	5	7	0	2	1	5	2	0	2	3	1	3	4	1	5	6	1	4	7	4	1	3	1	4	3	0	2	3	1	0	2	7	6	0.577	0.424	–	–	N
P	3	4	5	7	4	9	5	7	4	4	7	3	4	7	5	2	4	5	4	10	3	6	3	2	3	8	5	7	6	6	5	5	3	1	6	5	6	4	12	8	2	7	7	5	5	7	1	4	2	4	11	0.456	0.204	8.57E-01	n.s.	P
Q	2	2	4	5	2	4	2	1	2	3	5	5	3	4	4	13	8	6	5	3	8	2	4	24	1	3	4	3	1	2	4	2	1	2	0	4	4	1	4	6	2	2	4	5	2	1	4	0	3	2	11	0.342	0.335	5.17E-07	*	Q
R	2	2	6	1	1	3	4	6	4	4	8	3	3	5	6	1	9	4	4	2	3	4	4	3	2	6	5	4	3	3	6	6	2	3	3	5	0	2	1	4	5	4	4	3	2	6	3	4	7	4	3	1.260	0.622	–	–	R
S	8	6	11	11	4	9	5	4	4	7	2	8	11	6	4	8	8	6	7	9	4	7	5	3	8	2	2	7	8	5	5	6	3	7	5	9	7	12	4	6	9	9	7	7	9	7	7	2	10	8	17	0.386	0.149	2.24E-02	n.s.	S
T	8	4	2	5	2	1	4	2	2	1	8	4	4	3	4	3	4	8	5	5	4	13	1	1	3	6	6	9	4	6	3	3	6	5	4	3	2	7	7	2	4	7	5	5	6	1	3	3	7	2	13	0.334	0.188	2.06E-05	*	T
V	5	4	6	5	3	3	6	11	10	7	13	7	8	5	2	4	9	9	7	4	7	8	3	4	2	8	8	9	8	6	8	3	3	7	11	7	4	8	14	3	5	6	3	3	2	10	6	5	3	6	15	0.411	0.193	1.47E-01	n.s.	V
W	0	0	0	0	0	0	3	0	1	3	0	2	3	1	2	0	2	1	1	5	2	1	0	1	1	0	1	0	0	1	2	0	1	1	1	1	1	1	2	6	2	1	1	0	2	5	2	0	2	0	1	1.240	1.379	–	–	W
Y	6	4	9	3	3	5	3	4	7	2	8	6	7	11	5	28	19	30	22	64	58	44	35	29	10	13	7	7	0	2	5	9	7	5	2	12	21	13	6	1	2	54	1	0	3	0	50	2	4	1	5	2.596	3.247	–	–	Y
total	81	62	105	76	74	93	89	73	99	82	113	98	101	75	75	127	133	124	107	156	128	141	90	113	68	107	91	82	70	72	80	70	56	74	78	92	104	109	109	73	70	134	75	74	68	94	119	59	80	68	187	0.491	0.126			total

position -1

	C50	C49	C48	C47	C46	C45	C44	C43	C42	C41	C40	C39	C38	C37	C36	C35	C34	C33	C32	C31	C30	C29	C28	C27	C26	C25	C24	C23	C22	C21	C20	C19	C18	C17	C16	C15	C14	C13	C12	C11	C10	C9	C8	C7	C6	C5	C4	C3	C2	C1	C0	C-index	SD	*P-value*	mark	
A	4	3	7	6	5	7	5	3	3	6	15	10	2	3	11	13	11	8	5	7	10	4	7	21	4	5	9	8	1	3	8	4	2	6	4	12	6	8	3	3	8	8	5	0	4	4	5	1	4	3	12	0.507	0.323	–	–	A
C	2	1	6	1	1	5	0	2	1	2	0	4	6	3	0	2	1	4	2	0	2	2	1	2	0	4	1	0	2	1	3	3	2	1	3	5	0	2	13	0	2	1	4	3	2	3	3	2	5	2	7	0.334	0.316	9.33E-05	*	C
D	4	3	5	3	5	1	2	2	3	4	8	3	6	3	2	4	6	1	10	7	0	3	9	2	2	2	5	6	3	3	1	3	5	0	6	7	3	6	9	6	1	2	3	2	2	4	3	1	3	2	13	0.286	0.182	4.64E-07	*	D
E	2	4	4	2	6	8	5	5	10	5	3	7	5	2	3	4	11	7	4	7	6	5	1	6	1	7	4	8	5	4	12	4	4	10	5	11	4	2	8	9	7	6	5	3	3	2	5	2	3	6	14	0.374	0.191	1.49E-03	n.s.	E
F	0	2	7	5	2	3	2	3	5	4	2	1	3	6	1	2	3	5	1	2	4	3	0	2	2	1	2	0	0	4	3	0	3	6	3	3	3	9	3	1	1	3	3	2	0	2	8	1	3	2	4	0.680	0.505	–	–	F
G	4	3	2	6	5	1	12	4	8	6	12	6	3	4	5	6	10	2	5	6	1	7	4	3	3	5	4	7	1	2	4	6	3	2	5	6	4	8	4	1	5	5	5	6	1	7	4	3	1	3	8	0.575	0.320	–	–	G
H	4	5	4	3	3	2	2	1	2	1	7	1	5	0	3	4	2	4	5	2	5	2	2	0	2	5	3	0	3	1	2	2	1	0	2	3	3	2	2	1	1	2	3	0	2	2	0	2	3	0	6	0.387	0.266	2.29E-02	n.s.	H
I	5	6	5	3	3	3	5	5	4	4	7	5	4	3	4	7	9	6	4	13	25	12	3	6	3	4	1	4	0	2	4	0	4	2	7	0	4	2	3	4	1	1	2	4	3	4	1	0	2	2	2	2.150	1.998	–	–	I
K	2	2	4	1	5	3	4	1	9	5	1	4	2	9	3	4	3	1	3	2	4	8	5	2	2	4	8	6	3	4	5	3	0	5	2	4	26	9	5	6	5	2	2	1	5	4	4	8	7	2	6	0.730	0.644	–	–	K
L	4	5	15	11	6	10	8	9	5	8	3	9	10	10	8	12	10	10	15	19	5	17	6	15	4	9	13	4	6	2	8	16	5	7	2	6	2	3	18	9	9	5	4	3	5	5	49	3	10	6	16	0.554	0.455	–	–	L
M	2	1	3	1	0	2	1	2	2	1	6	3	1	1	3	11	1	8	0	5	5	1	8	3	1	0	2	3	0	0	3	0	2	0	1	0	1	1	3	0	0	51	3	1	0	3	2	3	1	0	2	1.520	3.646	–	–	M
N	2	6	4	4	1	1	2	5	1	0	4	3	2	1	2	3	5	3	3	3	3	7	3	5	2	4	3	2	4	15	0	0	0	8	2	2	8	5	5	2	0	3	3	5	3	3	4	2	0	4	7	0.463	0.369	8.49E-01	n.s.	N
P	4	2	5	3	2	8	8	3	6	7	5	3	9	1	2	6	5	6	9	6	2	7	1	3	5	9	4	5	10	3	5	5	5	7	5	5	4	8	4	3	4	5	5	6	5	7	8	5	7	5	11	0.467	0.197	1.00E+00	n.s.	P
Q	4	3	1	2	3	3	5	5	4	4	5	5	4	5	3	4	2	5	5	3	1	7	1	7	8	8	4	4	5	5	2	4	1	3	6	2	6	6	3	3	1	5	3	3	2	5	3	7	4	2	5	0.784	0.363	–	–	Q
R	2	2	8	5	6	3	2	4	4	6	6	4	7	2	7	4	5	9	2	4	2	2	2	5	4	8	9	9	4	6	4	6	2	3	7	7	9	15	8	8	6	5	3	4	10	6	7	2	6	2	7	0.751	0.392	–	–	R
S	10	2	10	8	2	7	7	6	11	7	10	11	8	3	5	9	13	18	12	16	9	13	5	5	8	4	6	2	9	5	6	3	5	3	4	8	10	8	6	6	8	13	7	14	10	19	4	5	8	10	17	0.468	0.233	9.59E-01	n.s.	S
T	13	4	4	6	8	1	3	3	10	5	4	5	7	5	6	6	5	4	6	9	15	9	4	7	6	9	1	6	4	3	3	5	6	4	1	1	4	3	5	4	3	9	6	8	5	6	3	5	8	4	29	0.187	0.097	5.55E-08	*	T
V	10	4	6	2	8	12	12	7	8	3	12	14	9	12	4	18	8	19	11	43	26	25	25	13	7	13	7	4	5	5	7	4	5	2	7	6	7	4	0	3	6	6	6	7	3	7	2	5	3	8	18	0.500	0.426	–	–	V
W	2	2	1	0	0	10	1	0	1	0	1	0	6	1	1	1	21	1	1	0	1	3	0	4	1	1	1	1	2	2	0	0	0	1	1	2	0	2	1	1	1	1	0	0	0	1	1	0	0	3	1	1.620	3.276	–	–	W
Y	1	2	4	4	3	3	3	3	2	4	2	0	2	1	2	7	2	3	4	2	2	4	3	2	3	5	4	3	3	2	0	2	1	4	5	2	0	6	6	3	1	1	3	2	3	0	3	2	2	2	2	1.330	0.767	–	–	Y
total	81	62	105	76	74	93	89	73	99	82	113	98	101	75	75	127	133	124	107	156	128	141	90	113	68	107	91	82	70	72	80	70	56	74	78	92	104	109	109	73	70	134	75	74	68	94	119	59	80	68	187	0.491	0.126			total

Additional file 5. Continued.

MOUSE x-x-V-x-I

position -4

	C50	C49	C48	C47	C46	C45	C44	C43	C42	C41	C40	C39	C38	C37	C36	C35	C34	C33	C32	C31
A	6	3	7	3	1	7	5	2	5	4	2	5	5	1	2	3	1	5	1	3
C	4	2	0	3	1	1	2	1	0	5	2	2	0	3	0	1	0	2	0	1
D	2	4	4	6	2	3	11	3	3	3	1	3	2	1	1	0	2	4	2	3
E	5	4	3	4	7	2	3	9	5	2	0	3	0	4	3	4	2	6	0	4
F	1	7	0	4	1	4	2	1	7	3	3	2	1	1	1	5	4	3	2	7
G	9	6	5	6	2	2	1	4	7	5	8	4	4	2	1	4	3	4	4	2
H	0	3	1	3	1	2	2	0	3	1	1	3	2	0	3	1	0	1	1	1
I	1	5	1	3	4	7	11	1	3	3	2	0	4	4	2	2	3	4	5	1
K	3	6	5	2	5	4	5	2	4	2	10	8	11	2	3	4	4	5	4	7
L	8	3	13	5	7	6	7	11	5	12	13	7	2	7	4	4	5	3	7	4
M	1	2	0	0	3	2	0	0	2	0	0	0	2	1	2	1	3	3	3	1
N	7	2	2	0	0	2	5	3	3	2	5	2	2	4	0	0	3	2	3	4
P	4	8	2	3	1	6	8	2	6	5	8	7	2	6	4	5	1	2	2	2
Q	3	10	3	2	2	1	6	8	5	4	1	5	3	2	2	3	2	6	8	1
R	2	1	5	5	3	0	3	3	3	3	4	4	8	7	7	4	1	5	1	1
S	1	10	5	3	9	5	3	3	7	4	3	6	5	5	5	3	7	4	7	1
T	0	3	2	4	8	7	2	2	4	3	5	2	9	2	4	4	2	3	4	4
V	9	10	12	7	5	7	7	4	6	4	12	5	10	3	11	6	2	5	3	3
W	0	2	0	0	1	1	0	1	0	6	0	2	2	0	2	0	5	1	1	0
Y	1	1	1	4	1	2	0	0	1	3	0	1	2	4	7	8	12	0	13	4
total	67	92	71	67	64	71	83	60	79	74	80	71	76	59	64	62	62	68	71	54

	C30	C29	C28	C27	C26	C25	C24	C23	C22	C21	C20	C19	C18	C17	C16	C15	C14	C13
A	2	3	1	5	2	1	3	3	5	5	5	1	5	2	5	5	4	4
C	2	0	1	1	0	1	2	4	3	0	0	1	1	1	2	0	0	1
D	1	5	5	4	7	6	5	4	6	1	5	0	3	1	4	2	1	3
E	4	2	3	4	3	1	3	3	3	2	2	0	4	5	2	1	4	3
F	4	3	2	3	2	4	1	4	2	0	3	4	0	1	1	2	3	0
G	5	4	3	18	7	10	13	6	6	8	8	3	4	6	6	3	6	7
H	2	0	1	3	0	2	0	2	1	2	2	0	0	2	8	1	2	5
I	3	4	3	2	4	4	2	1	7	5	5	3	4	6	4	4	3	2
K	2	2	5	3	2	1	6	1	4	2	3	0	2	1	18	11	2	2
L	10	6	9	4	6	4	5	2	6	7	5	10	3	9	6	7	4	6
M	1	0	0	1	0	2	2	1	2	3	2	0	2	1	0	1	3	0
N	2	3	1	8	3	2	0	1	1	1	3	5	5	3	2	2	2	3
P	6	2	2	8	6	4	3	4	8	12	10	4	7	15	5	10	1	16
Q	3	3	4	2	4	1	2	5	3	1	1	1	3	5	0	2	3	8
R	4	2	6	4	7	1	4	2	3	4	2	3	2	5	3	1	2	1
S	0	6	6	9	6	5	3	7	9	3	1	6	1	3	1	4	8	4
T	3	4	5	2	2	1	4	6	5	3	2	0	3	6	3	5	2	5
V	2	6	3	3	8	2	8	2	10	7	3	3	4	4	1	3	3	2
W	0	1	3	2	0	2	0	0	2	0	1	4	0	0	0	0	2	0
Y	8	18	8	6	2	5	6	0	0	1	1	1	2	1	1	0	1	0
total	64	74	71	92	71	59	72	58	86	67	64	49	55	77	72	64	56	72

	C12	C11	C10	C9	C8	C7	C6	C5	C4	C3	C2	C1	C0	C-index	SD	*P-value*	mark	
A	4	1	5	3	5	2	4	5	2	3	1	4	3	1.140	0.568	–	–	A
C	0	3	3	1	5	0	2	1	2	1	2	0	3	0.467	0.447	3.20E-05	✻	C
D	3	4	2	12	0	6	3	3	2	3	5	0	0	–	–	–	–	D
E	1	5	2	4	4	1	5	7	2	6	2	6	7	0.469	0.277	1.98E-07	✻	E
F	2	0	1	3	1	3	3	1	1	4	1	1	4	0.595	0.443	4.64E-02	n.s.	F
G	8	11	2	5	2	3	18	0	2	4	2	3	0	–	–	–	–	G
H	0	1	1	3	2	2	2	2	5	2	0	2	1	1.680	1.518	–	–	H
I	2	6	4	3	3	4	7	4	2	5	1	3	3	1.173	0.657	–	–	I
K	17	7	2	5	2	5	5	1	4	2	2	8	1	4.460	3.699	–	–	K
L	4	4	8	10	2	3	5	9	5	5	5	5	5	1.228	0.553	–	–	L
M	1	2	0	0	0	4	0	2	2	0	2	0	2	0.600	0.571	7.94E-01	n.s.	M
N	4	2	1	2	2	1	7	2	3	0	1	0	11	0.224	0.169	5.55E-08	✻	N
P	1	4	1	2	1	3	2	4	0	7	1	3	10	0.472	0.358	3.34E-06	✻	P
Q	1	0	4	1	2	3	3	2	1	1	1	4	7	0.431	0.315	9.68E-08	✻	Q
R	2	1	4	4	4	7	3	1	4	3	2	3	9	0.364	0.209	5.55E-08	✻	R
S	2	2	7	5	4	9	7	6	5	6	2	8	7	0.689	0.357	9.84E-01	n.s.	S
T	6	3	8	3	2	1	3	3	1	3	0	2	5	0.680	0.404	2.10E-01	n.s.	T
V	5	2	6	5	0	8	1	8	3	9	6	2	6	0.867	0.511	–	–	V
W	0	2	0	0	2	1	1	0	1	4	0	0	2	0.520	0.707	7.50E-03	n.s.	W
Y	0	0	2	1	0	1	0	1	3	2	1	1	3	0.920	1.261	–	–	Y
total	63	60	63	72	43	67	81	62	50	70	37	55	89	0.751	0.124			total

position -3

	C50	C49	C48	C47	C46	C45	C44	C43	C42	C41	C40	C39	C38	C37	C36	C35	C34	C33	C32	C31
A	2	13	1	3	5	6	4	4	5	6	9	5	9	2	5	6	3	2	2	8
C	1	5	3	3	2	2	0	1	3	1	0	1	0	1	3	7	0	2	1	1
D	2	3	3	0	3	2	3	4	7	1	4	3	2	5	2	2	3	7	2	1
E	3	7	1	1	5	2	6	2	5	2	3	2	3	5	4	2	2	1	4	4
F	0	3	0	4	1	1	1	3	2	6	5	1	2	1	1	2	2	2	3	2
G	3	6	9	4	2	7	8	6	3	7	5	2	8	2	4	3	4	8	2	2
H	0	4	5	4	7	2	2	1	0	4	5	5	4	3	0	2	1	3	3	0
I	3	6	3	3	4	5	6	4	5	4	0	1	5	1	1	3	8	5	5	0
K	5	4	9	4	3	1	5	7	9	3	4	7	2	3	3	5	1	4	3	3
L	6	11	4	4	6	6	12	6	5	2	7	10	5	6	10	8	4	11	4	5
M	3	2	2	0	2	2	4	1	2	0	4	3	2	0	2	1	1	0	1	2
N	3	2	6	4	3	2	2	1	8	5	3	5	2	0	1	2	3	1	1	0
P	2	4	3	4	7	3	4	1	4	11	3	5	4	2	4	2	1	4	5	1
Q	3	2	4	4	2	5	3	1	2	2	2	2	5	2	2	2	0	3	6	1
R	4	7	4	2	1	6	3	3	2	3	1	2	1	4	5	2	3	5	2	4
S	9	5	4	10	2	9	7	5	7	8	10	3	4	6	7	3	9	4	4	3
T	5	7	5	8	4	2	4	6	2	5	4	5	3	3	5	2	7	3	13	8
V	9	0	3	3	4	5	6	3	7	3	7	5	8	10	2	5	9	2	7	6
W	2	0	1	1	0	1	0	1	0	0	0	1	0	2	1	2	0	1	2	2
Y	2	1	1	1	1	2	3	0	1	1	4	3	7	1	2	1	1	0	1	1
total	67	92	71	67	64	71	83	60	79	74	80	71	76	59	64	62	62	68	71	54

	C30	C29	C28	C27	C26	C25	C24	C23	C22	C21	C20	C19	C18	C17	C16	C15	C14	C13
A	3	3	6	8	5	5	4	9	6	4	4	1	2	9	6	5	3	8
C	3	6	0	4	2	0	1	2	1	2	0	1	0	0	0	0	0	1
D	3	5	1	3	2	6	3	6	6	4	6	1	1	5	1	3	2	3
E	4	0	7	4	4	2	5	0	7	4	2	3	2	6	1	3	1	2
F	4	4	3	2	2	1	5	4	1	4	9	3	2	11	1	5	1	7
G	2	5	2	5	5	4	3	7	2	2	2	0	3	4	3	2	3	1
H	2	1	2	4	0	1	1	0	2	0	3	0	0	2	1	1	2	2
I	2	1	0	3	6	5	5	6	6	5	1	4	1	1	6	3	2	4
K	3	5	3	3	2	4	1	2	2	2	2	1	2	1	0	9	4	0
L	7	4	7	9	8	5	6	3	17	10	3	7	13	10	9	5	6	11
M	2	9	1	5	2	5	1	0	0	2	1	0	1	1	3	2	1	0
N	1	1	1	2	1	2	3	1	1	1	2	3	3	3	1	5	4	3
P	1	3	2	3	4	1	2	2	5	1	3	5	7	2	8	4	2	6
Q	1	3	6	3	8	0	4	3	4	5	5	4	2	4	2	2	2	1
R	2	1	1	16	4	3	1	3	0	2	5	5	5	1	18	4	5	0
S	4	7	2	3	5	3	6	2	4	5	3	3	3	6	7	3	8	6
T	13	8	17	8	7	6	8	2	8	4	4	3	3	3	2	5	3	3
V	4	4	5	4	1	2	9	3	7	4	7	4	4	6	2	2	5	4
W	0	0	1	1	3	0	3	2	4	2	0	1	0	2	0	0	1	2
Y	3	4	4	2	0	4	1	1	3	4	2	0	1	0	1	1	1	8
total	64	74	71	92	71	59	72	58	86	67	64	49	55	77	72	64	56	72

	C12	C11	C10	C9	C8	C7	C6	C5	C4	C3	C2	C1	C0	C-index	SD	*P-value*	mark	
A	7	2	4	2	3	2	3	1	4	2	1	2	0	–	–	–	–	A
C	0	2	3	3	1	4	2	0	0	0	0	1	1	1.520	1.657	–	–	C
D	15	6	8	15	2	5	6	4	3	8	4	2	4	0.990	0.751	–	–	D
E	2	5	7	2	2	3	3	1	2	5	1	4	10	0.316	0.188	5.55E-08	✻	E
F	7	6	2	3	3	3	1	2	2	2	2	1	5	0.580	0.449	5.44E-04	n.s.	F
G	1	1	4	3	2	3	8	3	3	1	4	3	2	1.860	1.097	–	–	G
H	1	2	2	2	2	3	1	1	2	2	0	1	0	–	–	–	–	H
I	2	6	2	4	0	4	6	2	3	3	1	1	6	0.557	0.343	7.22E-02	n.s.	I
K	4	5	3	3	4	7	4	7	1	3	2	2	6	0.587	0.368	1.20E-03	n.s.	K
L	3	3	4	4	5	5	2	6	9	4	0	7	3	2.160	1.082	–	–	L
M	1	0	1	1	1	2	3	1	1	0	2	2	0	–	–	–	–	M
N	0	0	2	0	0	3	1	5	1	3	1	4	4	0.560	0.433	3.19E-03	n.s.	N
P	2	2	2	5	0	2	7	6	3	8	2	5	6	0.597	0.371	8.77E-03	n.s.	P
Q	3	3	4	2	2	2	5	5	2	8	2	3	1	3.060	1.754	–	–	Q
R	4	3	2	10	6	4	4	4	1	6	4	5	3	1.287	1.113	–	–	R
S	2	5	7	7	2	3	3	3	5	11	2	3	20	0.252	0.123	5.55E-08	✻	S
T	4	4	2	5	1	8	9	3	5	2	4	4	7	0.740	0.451	3.90E-01	n.s.	T
V	4	2	3	1	5	0	3	7	1	1	2	4	4	1.070	0.617	–	–	V
W	0	0	0	0	0	0	0	0	2	0	1	1	1	0.860	1.010	–	–	W
Y	1	3	1	0	2	4	10	1	0	1	2	0	6	0.330	0.343	5.55E-08	✻	Y
total	63	60	63	72	43	67	81	62	50	70	37	55	89	0.751	0.124			total

position -1

	C50	C49	C48	C47	C46	C45	C44	C43	C42	C41	C40	C39	C38	C37	C36	C35	C34	C33	C32	C31
A	5	8	4	5	4	5	9	7	6	3	5	5	6	5	1	2	3	8	3	6
C	3	1	3	3	2	7	0	3	0	0	0	1	0	1	0	1	0	2	1	1
D	2	0	6	5	0	2	5	0	3	6	5	2	4	3	2	2	2	0	7	1
E	7	8	2	2	2	2	5	1	3	2	8	4	5	1	8	6	0	2	4	2
F	2	2	2	2	7	3	1	3	3	4	4	2	1	1	3	2	2	2	4	0
G	3	2	5	4	5	3	6	6	3	5	3	5	2	3	1	1	4	9	9	5
H	2	1	3	2	4	2	2	3	4	7	9	6	10	4	2	4	2	2	2	0
I	4	5	8	6	4	6	3	2	10	5	8	3	1	6	0	2	4	4	8	5
K	2	12	6	5	2	2	5	1	1	1	2	1	4	3	3	4	3	3	1	2
L	6	7	4	5	4	4	5	2	7	3	8	7	1	6	15	10	6	9	6	2
M	4	0	0	1	0	0	2	1	0	0	0	1	1	1	0	1	1	1	1	2
N	5	2	2	0	1	2	4	2	1	1	4	3	0	1	4	4	5	5	3	1
P	2	2	8	7	5	3	3	4	4	7	2	2	0	6	2	3	2	2	1	4
Q	1	3	2	1	3	1	3	5	2	1	1	2	7	4	4	7	2	3	2	4
R	5	3	2	3	6	6	4	6	7	4	2	4	9	2	2	1	1	1	3	4
S	3	12	3	1	3	5	9	3	7	11	8	5	11	7	5	2	8	6	4	1
T	7	7	4	2	2	1	6	4	3	4	4	3	2	1	5	1	0	5	2	6
V	2	7	4	3	6	7	5	6	12	5	2	8	12	4	7	4	9	2	8	5
W	0	1	1	4	1	1	5	1	2	2	2	4	0	0	0	1	7	0	0	2
Y	2	9	2	6	3	9	1	0	1	3	3	3	0	0	0	4	1	2	2	1
total	67	92	71	67	64	71	83	60	79	74	80	71	76	59	64	62	62	68	71	54

	C30	C29	C28	C27	C26	C25	C24	C23	C22	C21	C20	C19	C18	C17	C16	C15	C14	C13
A	7	6	4	4	3	2	2	1	9	3	4	2	3	5	4	2	3	10
C	2	7	1	0	2	3	0	0	6	4	1	1	2	4	1	2	4	1
D	4	0	2	5	4	4	3	4	2	2	3	3	5	4	0	10	4	3
E	3	1	6	3	3	2	6	2	5	3	2	0	4	4	2	1	1	2
F	1	4	3	2	7	0	3	3	5	5	13	3	0	1	5	3	2	3
G	1	7	5	5	2	7	5	5	4	2	4	1	5	5	5	4	3	3
H	1	1	1	1	1	4	1	4	0	1	0	1	0	1	3	0	1	0
I	4	1	6	9	3	4	3	2	2	3	1	2	2	6	1	1	4	4
K	2	4	4	2	13	2	2	2	4	3	2	2	2	5	8	6	3	1
L	5	9	4	10	2	7	6	8	8	9	9	12	6	13	5	10	7	15
M	1	4	1	2	1	1	0	0	3	1	3	0	0	2	1	1	0	3
N	2	2	2	6	0	0	0	3	0	2	4	0	1	4	1	0	2	2
P	3	3	3	3	4	3	2	0	6	4	3	3	5	1	3	3	3	6
Q	3	2	2	6	2	2	7	0	7	10	0	3	6	3	3	3	2	2
R	4	2	2	4	4	1	7	6	5	5	2	1	2	5	1	5	6	2
S	7	1	6	3	4	3	7	9	7	0	1	5	3	3	4	5	2	7
T	3	2	4	19	9	6	7	2	6	4	5	1	4	4	17	3	2	2
V	10	17	14	6	4	7	8	6	3	4	6	4	3	5	3	2	6	4
W	0	0	1	0	1	0	3	0	1	0	0	3	0	0	1	0	0	0
Y	1	1	0	2	2	1	0	1	3	2	1	2	2	2	4	3	1	2
total	64	74	71	92	71	59	72	58	86	67	64	49	55	77	72	64	56	72

	C12	C11	C10	C9	C8	C7	C6	C5	C4	C3	C2	C1	C0	C-index	SD	*P-value*	mark	
A	6	3	5	3	2	3	6	3	1	2	2	0	5	0.840	0.454	–	–	A
C	2	2	3	2	0	0	3	1	0	1	0	2	4	0.430	0.438	1.58E-06	✻	C
D	1	4	5	3	3	1	6	4	2	4	2	4	12	0.263	0.169	5.55E-08	✻	D
E	5	3	3	3	2	4	4	2	4	4	6	1	4	0.825	0.508	–	–	E
F	3	2	1	3	1	3	3	1	1	3	1	6	1	2.820	2.182	–	–	F
G	1	5	8	3	4	6	3	3	0	5	4	2	0	–	–	–	–	G
H	10	0	0	15	3	6	0	2	1	3	2	4	5	0.552	0.610	2.05E-04	n.s.	H
I	6	2	8	2	4	2	4	7	5	1	2	3	1	3.960	2.382	–	–	I
K	7	7	5	3	2	2	3	3	6	6	2	6	11	0.331	0.234	5.55E-08	✻	K
L	7	5	4	13	5	4	6	7	4	7	2	3	5	1.316	0.654	–	–	L
M	1	0	3	2	1	2	1	1	0	2	0	3	1	1.140	1.107	–	–	M
N	2	1	3	2	4	5	2	2	3	2	2	1	6	0.367	0.265	5.62E-08	✻✻	N
P	1	1	2	4	2	4	3	3	2	2	0	3	5	0.616	0.354	2.70E-03	n.s.	P
Q	1	6	0	2	1	1	0	3	0	1	3	1	3	0.933	0.741	–	–	Q
R	0	3	0	4	2	3	4	1	3	5	4	2	5	0.680	0.400	8.96E-01	n.s.	R
S	5	7	3	2	1	6	6	4	8	3	1	6	12	0.405	0.239	5.56E-08	✻	S
T	2	4	5	2	2	11	3	1	1	8	0	3	2	2.110	1.841	–	–	T
V	1	2	2	2	4	1	13	7	4	7	3	2	3	1.853	1.169	–	–	V
W	0	0	1	1	0	0	1	1	2	1	0	0	2	0.510	0.739	4.11E-05	✻	W
Y	2	3	2	1	0	3	10	6	3	3	1	3	2	1.190	1.120	–	–	Y
total	63	60	63	72	43	67	81	62	50	70	37	55	89	0.751	0.124			total

Additional file 5. Continued.

MOUSE x-x-V-x-V

position -4

	C50	C49	C48	C47	C46	C45	C44	C43	C42	C41	C40	C39	C38	C37	C36	C35	C34	C33	C32	C31	C30	C29	C28	C27	C26	C25	C24	C23	C22	C21	C20	C19	C18	C17	C16	C15	C14	C13	C12	C11	C10	C9	C8	C7	C6	C5	C4	C3	C2	C1	C0	C-index	SD	*P-value*	mark	
A	11	3	5	9	8	8	13	3	5	5	8	5	10	7	4	10	8	4	7	5	5	5	10	13	10	9	5	7	8	5	6	3	7	2	4	5	8	3	3	5	2	10	5	10	5	5	9	6	9	8	7	0.943	0.394	3.28E-01	n.s.	A
C	3	2	2	2	1	0	0	4	0	3	2	1	3	1	3	1	2	2	3	2	4	1	1	4	2	2	1	5	5	1	2	0	0	0	2	5	3	3	4	0	1	4	1	2	2	3	4	2	2	1	3	0.693	0.471	5.28E-05	*	C
D	6	8	1	4	4	6	5	4	4	10	3	5	5	7	6	5	3	3	7	6	14	6	7	5	8	5	4	8	0	6	10	0	5	4	6	2	3	3	4	8	3	4	3	1	3	4	4	2	6	2	4	1.210	0.655	–	–	D
E	7	11	5	6	8	4	6	3	4	9	7	6	8	8	3	7	5	7	6	8	11	4	7	3	8	12	5	10	9	5	6	8	18	6	2	3	4	4	8	4	7	2	7	4	7	3	2	3	7	6	8	0.783	0.371	6.40E-06	*	E
F	4	6	1	3	2	3	2	4	6	7	1	1	3	4	9	2	4	10	3	5	3	2	4	6	1	5	5	1	5	3	7	5	4	4	2	4	1	4	6	1	3	3	1	4	3	5	2	4	1	6	4	0.925	0.523	3.07E-01	n.s.	F
G	21	9	7	8	6	3	10	10	9	10	3	6	5	8	5	7	6	8	6	6	6	5	7	2	9	7	11	12	7	8	1	4	8	4	4	6	10	5	13	5	3	4	5	9	9	7	5	3	3	5	6	1.133	0.557	–	–	G
H	5	6	5	2	3	4	6	5	7	1	3	4	3	4	2	5	3	7	0	2	1	3	2	4	4	3	1	0	3	4	3	2	2	1	2	2	2	2	2	3	6	1	2	1	3	1	4	3	3	2	2	1.490	0.848	–	–	H
I	3	4	6	7	7	2	7	4	2	6	3	5	4	4	4	5	1	3	4	3	9	5	15	5	4	8	3	7	2	7	4	3	5	5	7	3	5	1	4	11	2	5	5	5	6	6	1	2	4	2	1	4.700	2.573	–	–	I
K	4	6	5	4	5	17	7	9	13	8	14	12	29	3	21	21	10	9	5	7	19	6	4	6	2	5	4	6	3	3	7	3	6	6	5	8	9	4	17	14	4	15	2	8	2	2	7	1	4	5	5	1.584	1.184	–	–	K
L	10	15	9	10	7	6	12	8	10	13	11	16	8	8	7	16	10	7	11	12	12	9	13	13	6	7	5	7	6	7	7	8	10	14	11	8	7	7	4	10	7	10	5	6	10	8	10	9	10	9	7	1.317	0.404	–	–	L
M	1	0	1	1	6	1	2	0	4	2	1	1	1	2	3	4	2	7	2	2	1	1	0	3	5	2	4	2	0	2	0	3	9	2	3	4	6	2	0	3	0	1	0	2	1	1	1	1	3	1	4	0.530	0.484	5.93E-08	*	M
N	4	3	5	5	5	2	2	3	1	2	6	1	5	4	1	2	2	3	4	2	1	0	5	2	5	1	3	3	1	0	5	3	5	3	3	2	0	4	4	2	1	5	3	3	2	2	3	3	4	0	2	1.400	0.795	–	–	N
P	9	7	10	2	9	8	6	4	2	9	8	4	5	3	9	3	9	6	7	4	6	5	9	5	8	3	5	1	6	1	6	6	2	7	6	5	4	2	5	0	4	4	2	4	10	0	5	2	2	3	6	0.840	0.452	3.56E-02	n.s.	P
Q	6	16	4	7	4	3	2	5	4	2	6	7	4	4	4	7	6	4	3	1	10	2	2	5	6	4	10	1	3	6	1	6	2	2	4	3	5	5	1	5	2	3	1	4	7	3	7	9	4	1	8	0.558	0.354	5.55E-08	*	Q
R	5	5	6	5	11	3	7	3	2	6	6	5	5	7	2	9	4	4	6	3	7	9	4	3	2	1	8	4	5	3	5	3	5	2	4	2	2	3	4	3	3	5	1	5	2	4	8	6	7	4	7	0.651	0.314	5.76E-08	*	R
S	11	6	9	13	7	4	10	3	11	7	9	13	9	4	11	5	8	9	6	7	7	4	4	5	10	7	11	8	6	15	5	5	10	8	7	5	2	11	7	10	6	7	11	7	8	6	7	5	8	7	3	2.540	0.923	–	–	S
T	7	8	9	8	7	3	4	4	4	3	10	4	7	13	4	4	5	6	5	8	4	5	9	8	5	6	2	7	3	2	4	4	4	6	5	8	8	5	7	7	2	5	4	3	3	3	2	5	2	9	5	1.080	0.487	–	–	T
V	7	7	2	15	9	6	8	7	14	6	16	11	14	12	12	10	9	9	9	7	8	5	9	10	6	3	9	7	7	9	8	9	7	6	11	11	11	4	11	1	11	8	7	9	12	9	10	1	7	4	7	1.200	0.468	–	–	V
W	2	0	0	0	0	0	0	1	0	1	1	0	1	0	3	1	5	1	0	2	1	4	0	2	1	3	0	2	0	2	1	1	2	5	1	3	0	0	2	1	0	2	0	0	0	0	0	1	1	3	1	1.120	1.319	–	–	W
Y	4	5	1	5	2	2	3	4	2	1	2	6	2	2	1	3	1	2	4	3	5	3	4	5	5	6	5	1	5	1	6	1	3	2	2	3	1	2	1	2	0	2	0	2	1	1	2	2	2	2	3	0.880	0.546	2.39E-02	n.s.	Y
total	130	127	93	116	111	85	112	88	104	111	120	113	131	105	114	127	103	111	98	95	134	84	116	109	107	99	101	99	84	90	94	77	114	89	91	92	91	74	107	95	67	100	65	89	96	73	93	70	89	80	93	1.067	0.182			total

position -3

	C50	C49	C48	C47	C46	C45	C44	C43	C42	C41	C40	C39	C38	C37	C36	C35	C34	C33	C32	C31	C30	C29	C28	C27	C26	C25	C24	C23	C22	C21	C20	C19	C18	C17	C16	C15	C14	C13	C12	C11	C10	C9	C8	C7	C6	C5	C4	C3	C2	C1	C0	C-index	SD	*P-value*	mark	
A	9	19	10	11	9	5	7	6	4	12	7	13	14	11	9	3	5	10	5	8	10	8	11	10	9	7	6	9	5	5	6	8	5	8	5	4	9	4	13	6	3	7	2	11	5	4	8	4	1	5	7	1.071	0.493	–	–	A
C	5	5	1	3	5	3	4	4	6	4	4	3	2	5	4	4	2	7	4	7	3	3	4	0	4	2	6	4	5	10	3	1	7	3	5	3	3	1	2	2	3	3	2	2	6	1	5	2	3	7	3	1.247	0.645	–	–	C
D	4	4	3	4	3	5	5	5	1	5	3	5	6	4	2	7	4	3	1	1	6	2	5	3	3	5	6	4	4	7	3	3	5	4	5	7	3	5	15	8	4	5	3	3	5	1	3	4	9	0	3	1.433	0.798	–	–	D
E	4	6	3	6	16	5	8	6	8	7	8	2	5	0	10	4	6	6	10	6	9	6	5	7	10	2	3	6	7	8	7	2	8	6	7	6	6	6	5	6	5	6	3	0	6	4	6	3	5	7	8	0.733	0.337	8.67E-08	*	E
F	5	3	4	5	7	1	2	1	2	5	2	1	1	2	5	12	2	7	7	5	4	1	2	2	4	1	0	3	1	4	4	2	8	4	3	7	4	3	3	3	0	5	4	2	5	1	5	2	1	5	2	1.720	1.170	–	–	F
G	8	7	11	9	4	15	7	5	10	9	14	8	21	5	7	7	7	4	7	7	10	5	1	9	5	15	1	5	6	7	6	4	6	5	9	6	6	4	5	4	2	9	4	6	3	6	4	4	10	7	3	2.307	1.221	–	–	G
H	18	2	2	3	3	2	1	1	0	0	3	4	0	4	8	4	1	3	1	5	4	0	0	3	4	2	3	1	3	1	4	0	1	1	1	4	0	1	3	5	1	4	1	2	2	1	5	1	4	2	0	–	–	–	–	H
I	4	6	1	6	3	4	7	2	5	3	6	9	5	6	13	5	1	11	5	3	10	2	11	3	8	8	8	4	4	4	3	3	20	4	9	3	4	2	3	3	2	4	3	7	8	2	4	1	6	0	2	2.580	1.797	–	–	I
K	5	6	8	4	6	3	8	10	5	3	7	14	9	5	4	5	7	3	1	7	5	6	6	6	7	11	2	1	4	1	3	5	3	1	5	10	7	3	2	2	2	5	6	8	9	8	2	2	3	5	10	0.520	0.289	5.55E-08	*	K
L	7	12	9	9	10	8	4	7	9	12	7	11	13	12	10	11	16	13	6	8	9	4	10	14	14	3	4	12	7	10	16	7	13	11	8	7	8	10	11	6	5	6	8	10	5	3	10	4	5	5	6	1.463	0.556	–	–	L
M	4	6	1	6	3	2	1	2	11	5	4	3	8	2	8	2	2	2	2	1	2	2	2	3	0	1	3	1	1	2	1	2	1	2	0	2	1	1	1	2	3	4	0	1	4	2	0	1	1	2	4	0.615	0.547	8.25E-08	*	M
N	1	8	1	3	1	2	5	3	5	2	6	2	5	4	2	8	5	6	1	1	3	2	3	3	2	3	6	3	2	1	5	8	5	0	3	4	5	5	1	3	3	2	3	5	1	1	3	3	1	1	0	–	–	–	–	N
P	9	4	8	12	6	4	11	5	9	4	6	6	1	6	3	6	4	8	8	6	13	12	8	16	2	9	13	8	7	6	4	5	8	6	5	5	2	5	11	7	9	4	2	5	4	3	3	4	7	5	13	0.498	0.248	5.55E-08	*	P
Q	10	7	2	7	6	3	3	3	5	1	3	5	3	7	8	7	3	4	7	3	8	7	13	4	8	2	2	3	3	3	5	6	1	7	6	3	4	1	3	6	2	4	3	6	5	0	4	3	7	1	2	2.270	1.294	–	–	Q
R	5	9	5	4	12	2	3	5	3	10	6	1	4	3	0	1	5	6	8	5	4	4	10	7	4	7	10	7	5	3	3	2	0	3	3	2	4	5	11	8	4	2	3	1	5	7	6	5	3	2	3	1.580	0.947	–	–	R
S	13	7	5	8	5	3	9	6	7	11	5	6	3	6	5	9	6	5	5	3	7	6	8	4	10	5	14	14	4	5	10	7	5	10	7	9	8	6	8	7	8	7	8	8	8	9	7	5	6	8	12	0.592	0.211	5.55E-08	*	S
T	6	7	11	5	3	8	8	7	6	3	4	8	8	4	2	11	10	5	4	3	7	3	7	3	6	6	6	5	6	2	3	8	5	4	5	6	1	3	3	1	3	10	7	5	3	8	9	12	4	7	7	0.803	0.381	4.83E-04	n.s.	T
V	8	6	5	8	7	5	14	9	5	12	19	8	19	12	12	16	13	5	11	11	14	4	1	5	2	4	5	7	6	7	3	4	10	6	4	2	8	5	3	14	7	9	1	4	8	7	6	8	7	9	6	1.283	0.711	–	–	V
W	1	1	1	1	1	1	4	0	2	3	2	0	0	2	2	2	3	0	1	0	1	1	1	2	0	3	0	1	1	2	1	0	1	2	0	1	0	1	1	1	0	1	0	1	1	2	3	2	2	0	0	–	–	–	–	W
Y	4	2	2	2	1	4	1	1	1	0	4	4	4	5	0	3	1	3	4	5	5	6	8	5	5	3	3	1	3	2	4	0	2	2	1	1	8	3	3	1	1	3	2	2	3	3	0	0	4	2	2	1.370	0.952	–	–	Y
total	130	127	93	116	111	85	112	88	104	111	120	113	131	105	114	127	103	111	98	95	134	84	116	109	107	99	101	99	84	90	94	77	114	89	91	92	91	74	107	95	67	100	65	89	96	73	93	70	89	80	93	1.067	0.182			total

position -1

	C50	C49	C48	C47	C46	C45	C44	C43	C42	C41	C40	C39	C38	C37	C36	C35	C34	C33	C32	C31	C30	C29	C28	C27	C26	C25	C24	C23	C22	C21	C20	C19	C18	C17	C16	C15	C14	C13	C12	C11	C10	C9	C8	C7	C6	C5	C4	C3	C2	C1	C0	C-index	SD	*P-value*	mark	
A	6	7	8	10	5	8	15	5	7	4	11	19	12	10	19	10	3	10	8	8	11	10	8	10	11	13	13	13	7	7	5	4	12	8	10	7	13	9	6	7	8	6	3	6	10	4	3	4	6	4	2	4.230	1.863	–	–	A
C	5	8	6	9	8	5	9	3	6	7	2	5	4	3	0	4	4	5	2	8	6	1	7	2	3	6	3	3	5	2	2	3	10	2	8	3	3	1	3	2	5	6	5	2	6	2	4	3	4	2	3	1.447	0.793	–	–	C
D	1	2	4	4	10	3	3	3	3	1	9	4	6	2	5	7	7	5	7	4	8	6	1	4	5	5	3	2	2	0	3	3	4	6	3	6	2	3	4	3	2	4	3	1	6	2	2	1	3	4	7	0.546	0.310	5.55E-08	*	D
E	4	7	7	1	9	4	3	4	4	7	5	6	7	3	3	9	3	6	5	6	10	3	8	5	4	2	2	6	8	7	14	3	5	5	2	4	1	4	6	1	0	4	3	7	4	13	7	4	5	2	2	2.520	1.428	–	–	E
F	6	12	4	3	3	8	6	7	7	2	4	2	2	3	7	3	7	6	5	3	7	3	0	6	8	6	4	4	2	2	2	1	1	3	5	2	3	3	4	6	4	3	1	1	2	2	10	1	2	1	6	0.663	0.428	2.38E-06	*	F
G	9	8	4	6	3	1	5	1	3	2	6	7	5	3	6	6	10	4	5	6	6	1	7	9	3	6	5	9	4	3	4	5	5	3	2	4	6	5	4	4	3	4	3	4	4	2	7	2	2	3	3	1.527	0.726	–	–	G
H	5	3	3	3	2	1	3	6	2	3	2	5	4	4	3	1	1	5	4	0	1	3	3	2	4	4	2	2	3	1	1	3	4	3	5	4	0	0	3	3	3	1	0	2	3	3	1	5	1	3	0	–	–	–	–	H
I	2	3	2	3	3	3	3	2	2	5	7	7	7	2	5	6	5	12	7	5	3	4	5	3	2	2	11	5	4	6	4	5	7	6	3	1	1	1	7	4	3	4	5	3	3	3	8	2	2	1	2	2.090	1.207	–	–	I
K	5	9	4	3	4	4	7	5	6	14	11	7	3	3	5	7	4	5	3	5	6	3	4	6	11	8	6	4	3	3	3	3	5	13	9	7	6	6	12	10	4	11	4	9	5	6	2	5	5	7	9	0.667	0.323	7.99E-08	*	K
L	12	9	9	10	10	11	5	6	6	15	1	7	9	9	9	8	10	9	10	11	12	11	15	13	6	7	13	7	6	10	11	8	7	7	6	11	9	6	5	6	3	14	4	6	8	6	7	4	7	15	7	1.217	0.452	–	–	L
M	3	1	2	3	5	6	7	5	10	4	3	2	2	2	2	8	1	3	2	2	11	0	2	6	0	1	1	1	2	1	7	4	1	2	2	3	2	2	3	0	5	0	3	4	1	2	3	2	1	2	0	–	–	–	–	M
N	3	0	3	2	1	1	8	4	5	3	6	0	3	2	4	6	3	1	3	4	4	1	14	10	3	3	3	3	7	3	4	3	0	3	3	3	7	1	3	3	1	5	0	2	3	4	0	8	3	3	2	1.720	1.329	–	–	N
P	10	4	6	8	4	10	5	5	9	8	12	10	8	12	5	6	3	6	7	5	11	7	3	9	6	5	6	5	5	10	7	5	2	5	4	6	11	4	8	5	6	6	4	10	15	8	13	10	6	2	12	0.578	0.245	5.55E-08	*	P
Q	7	10	7	3	6	0	3	3	6	5	6	5	5	7	3	6	4	1	4	2	4	3	4	2	6	5	4	2	3	2	4	2	4	3	4	3	3	2	4	8	2	4	4	1	2	1	3	3	3	2	9	0.422	0.220	5.55E-08	*	Q
R	4	4	4	6	5	2	2	1	3	3	5	2	2	6	2	2	3	4	1	4	5	5	4	4	4	1	5	5	2	10	2	6	25	4	5	3	7	3	7	7	3	6	5	5	4	5	3	2	7	7	2	2.260	1.756	–	–	R
S	32	17	8	13	14	5	9	9	5	8	15	5	22	6	13	25	18	12	6	4	10	8	9	6	11	8	7	8	7	5	9	4	5	4	7	7	4	7	14	10	4	10	4	6	9	3	6	2	13	6	9	1.020	0.643	9.87E-02	n.s.	S
T	6	11	10	9	5	7	8	7	6	8	6	8	12	13	8	6	6	8	5	8	9	5	5	5	9	7	6	5	6	4	3	6	7	7	7	5	5	6	5	7	4	6	4	11	3	2	3	4	9	10	10	0.664	0.240	5.56E-08	*	T
V	8	5	1	12	8	4	7	4	13	6	7	8	15	12	11	4	8	7	9	5	7	10	8	5	6	7	5	9	6	12	7	7	7	4	3	10	3	8	7	6	4	5	5	9	7	4	8	6	4	2	1	6.900	2.901	–	–	V
W	0	3	0	5	1	0	1	1	1	1	0	1	2	0	1	2	1	2	2	0	1	0	5	0	2	1	1	2	0	0	2	0	1	1	0	2	0	2	1	3	1	0	1	0	0	0	1	2	1	1	3	0.367	0.388	5.55E-08	*	W
Y	2	4	1	3	5	2	3	7	0	5	2	3	1	3	3	1	2	0	3	5	2	0	4	2	3	2	1	4	2	2	0	2	2	0	3	1	5	1	1	0	2	1	4	0	1	1	2	0	5	3	4	0.555	0.414	7.05E-08	*	Y
total	130	127	93	116	111	85	112	88	104	111	120	113	131	105	114	127	103	111	98	95	134	84	116	109	107	99	101	99	84	90	94	77	114	89	91	92	91	74	107	95	67	100	65	89	96	73	93	70	89	80	93	1.067	0.182			total

Additional file 5. Continued.

ZEBRAFISH x-x-S-x-I

position -4

	C50	C49	C48	C47	C46	C45	C44	C43	C42	C41	C40	C39	C38	C37	C36	C35	C34	C33	C32	C31	C30	C29	C28	C27	C26	C25	C24	C23	C22	C21	C20	C19	C18	C17	C16	C15	C14	C13	C12	C11	C10	C9	C8	C7	C6	C5	C4	C3	C2	C1	C0	C-index	SD	*P-value*	mark	
A	3	5	3	2	4	5	3	0	3	6	2	3	1	6	5	2	3	5	3	6	4	2	6	4	5	7	4	2	4	3	1	4	4	4	0	5	1	2	2	7	7	5	5	0	3	0	4	3	4	1	4	0.865	0.472	–	–	A
C	1	7	0	1	0	4	2	5	3	4	1	1	4	2	0	2	0	2	1	2	0	3	4	0	0	2	0	0	2	2	0	1	1	1	0	0	1	1	1	5	1	1	1	3	0	2	1	1	3	2	0	–	–	–	–	C
D	2	8	4	6	4	4	3	8	0	0	8	4	1	0	2	3	0	0	2	2	3	3	6	1	2	8	3	6	3	3	4	3	5	1	0	3	4	7	2	5	1	3	4	9	7	2	3	3	4	7	3	1.173	0.812	–	–	D
E	3	6	5	4	4	2	12	2	3	3	2	1	6	3	7	4	2	5	4	1	1	4	3	7	4	5	3	3	3	2	0	5	7	4	3	3	3	5	4	3	7	6	3	6	5	3	5	9	1	4	11	0.364	0.199	5.55E-08	*	E
F	1	1	3	1	4	2	4	1	4	0	1	1	6	0	4	1	4	0	2	4	4	1	3	3	2	1	0	9	5	3	4	4	4	2	0	1	1	1	1	1	6	7	6	7	3	4	1	3	2	1	0	–	–	–	–	F
G	3	3	3	5	2	5	6	5	4	8	1	1	1	1	6	2	3	5	1	4	5	2	3	6	5	3	2	3	2	3	5	0	4	0	1	8	4	4	1	5	9	6	4	2	2	5	6	3	3	5	4	0.900	0.520	–	–	G
H	3	3	2	2	2	1	2	1	1	1	6	0	0	0	2	0	2	1	0	3	0	1	3	2	7	1	1	1	1	2	1	1	5	1	3	1	2	1	1	0	0	7	1	5	0	3	0	0	3	1	1	1.740	1.747	–	–	H
I	2	2	3	0	3	12	1	6	2	6	6	7	6	5	2	2	2	4	3	4	7	3	1	3	4	3	0	3	8	1	3	5	0	10	11	3	3	4	2	5	3	2	0	2	15	5	5	6	3	3	2	2.010	1.547	–	–	I
K	4	4	1	2	3	7	4	1	0	2	3	2	1	4	35	4	2	4	1	3	4	8	5	11	1	4	11	5	2	1	6	9	5	5	10	5	5	4	7	5	6	3	3	1	1	0	2	1	4	5	5	0.904	1.030	–	–	K
L	5	8	3	4	4	4	12	7	6	8	3	7	3	6	8	11	6	1	1	7	7	8	4	8	10	4	3	5	1	6	7	9	10	6	6	5	8	4	8	10	12	4	4	11	10	8	9	6	11	6	8	0.810	0.357	–	–	L
M	1	4	0	1	2	2	1	1	0	1	1	5	2	1	4	0	3	0	0	0	1	1	3	4	3	1	1	3	1	0	0	1	2	0	0	1	1	1	1	1	5	1	3	1	1	1	3	0	1	0	4	0.355	0.339	4.12E-07	*	M
N	4	3	1	4	4	5	3	4	1	4	3	1	1	1	4	4	2	1	5	0	1	2	2	6	1	1	1	1	2	1	7	2	1	2	0	1	1	4	0	2	4	2	2	1	3	4	1	2	3	1	8	0.290	0.203	5.55E-08	*	N
P	5	4	4	8	7	3	3	7	2	2	1	3	2	3	6	3	1	2	4	4	3	2	1	7	3	1	3	8	3	8	5	4	2	0	7	7	0	10	1	8	9	3	2	2	2	3	0	2	3	6	9	0.420	0.285	3.01E-06	*	P
Q	5	4	2	6	6	2	7	4	1	2	4	2	0	1	7	3	2	2	2	2	3	2	0	1	1	4	5	1	0	2	1	0	1	1	0	3	1	8	1	2	2	2	3	5	1	2	2	4	7	2	3	0.873	0.683	–	–	Q
R	2	4	3	1	0	1	5	1	3	8	2	7	4	1	2	3	4	5	3	3	5	2	3	2	4	4	1	2	6	5	5	4	6	4	4	2	2	4	1	6	3	1	5	5	4	5	2	5	2	6	11	0.313	0.164	5.55E-08	*	R
S	5	6	11	3	9	2	5	7	4	2	11	11	8	16	6	8	6	3	10	6	8	6	7	6	6	4	5	7	8	11	8	6	4	8	5	8	3	4	10	8	3	5	12	1	8	5	8	7	5	6	9	0.736	0.324	–	–	S
T	4	1	8	0	8	2	3	2	2	4	5	4	3	3	5	3	1	3	3	3	4	3	8	8	11	5	5	6	5	5	4	2	4	4	5	1	6	1	4	3	7	3	3	3	2	3	2	6	3	2	4	0.975	0.547	–	–	T
V	3	9	2	2	3	4	4	6	3	5	5	3	3	4	6	1	5	0	7	4	6	6	3	3	6	5	9	1	1	3	5	2	2	1	6	4	7	1	4	2	4	4	7	2	7	3	6	4	8	1	1	4.040	2.204	–	–	V
W	1	0	0	0	0	0	2	1	0	5	1	0	1	2	0	0	0	0	2	1	4	2	2	1	0	1	0	0	1	0	1	2	0	1	1	0	2	1	1	1	0	2	1	1	1	0	1	0	1	0	0	–	–	–	–	W
Y	2	1	2	2	0	0	2	1	1	0	1	3	4	1	1	1	0	1	0	0	2	2	1	1	0	3	0	1	0	0	2	0	2	1	2	1	4	3	2	2	5	0	2	6	7	7	7	3	0	1	4	0.450	0.474	3.32E-06	*	Y
total	59	83	60	54	69	67	84	70	43	71	67	66	57	60	112	57	48	44	54	59	72	63	68	84	75	67	57	67	58	61	69	64	69	56	64	62	59	70	54	81	94	67	71	73	82	65	68	68	71	60	91	0.730	0.132			total

position -3

	C50	C49	C48	C47	C46	C45	C44	C43	C42	C41	C40	C39	C38	C37	C36	C35	C34	C33	C32	C31	C30	C29	C28	C27	C26	C25	C24	C23	C22	C21	C20	C19	C18	C17	C16	C15	C14	C13	C12	C11	C10	C9	C8	C7	C6	C5	C4	C3	C2	C1	C0	C-index	SD	*P-value*	mark	
A	8	5	7	4	3	3	5	5	1	5	6	3	6	3	7	2	3	2	2	6	3	5	4	3	2	4	5	0	2	4	3	3	3	3	4	3	3	2	1	8	4	4	2	3	12	2	2	4	3	2	5	0.756	0.422	–	–	A
C	3	5	0	2	0	0	3	2	1	1	7	0	0	1	3	1	1	3	0	2	0	0	0	2	0	0	3	2	2	2	4	2	2	1	5	0	1	4	2	0	2	2	5	3	0	0	0	3	2	0	2	0.840	0.836	–	–	C
D	7	3	4	3	2	2	3	1	3	2	4	5	4	2	6	0	2	0	2	7	4	0	1	7	6	2	3	3	3	2	3	2	5	4	4	3	2	3	1	5	3	5	3	4	4	5	5	5	2	6	3	1.113	0.593	–	–	D
E	2	5	1	1	5	8	5	4	1	2	2	2	4	2	6	2	1	3	3	0	3	4	3	1	1	2	3	4	4	5	1	4	6	0	2	1	3	3	1	6	6	2	3	4	10	2	5	5	4	3	19	0.168	0.107	5.55E-08	*	E
F	3	2	2	4	1	7	11	2	2	2	0	5	2	1	3	3	4	2	8	1	3	1	2	7	3	5	2	5	3	4	0	7	2	3	3	2	0	2	3	4	6	4	5	4	1	5	3	1	5	6	6	0.553	0.371	4.40E-03	n.s.	F
G	2	5	6	2	6	2	1	5	4	2	1	7	3	5	3	2	0	2	4	3	3	5	6	1	8	4	3	9	2	5	10	5	5	2	3	7	2	3	3	6	6	2	7	7	6	3	1	2	1	0	3	1.280	0.786	–	–	G
H	1	3	4	1	2	3	5	0	2	1	1	2	1	0	1	2	0	1	1	0	3	4	1	0	1	2	2	4	2	1	2	0	4	0	1	1	2	1	3	1	1	2	1	2	0	1	4	1	6	3	3	0.580	0.470	1.56E-02	n.s.	H
I	6	5	2	4	5	5	4	2	2	6	2	3	2	6	4	2	2	1	2	4	11	1	8	5	2	2	2	7	0	2	3	3	7	2	3	5	2	4	5	3	10	4	2	2	6	6	4	3	7	5	5	0.780	0.459	–	–	I
K	1	8	2	2	1	2	6	1	2	6	3	2	0	2	2	4	1	4	6	2	2	3	3	4	7	5	3	3	7	1	3	4	3	2	4	4	5	6	1	2	6	8	2	5	4	3	2	5	3	4	6	0.570	0.327	1.19E-02	n.s.	K
L	3	7	6	2	8	5	3	6	6	7	7	3	9	6	6	5	9	4	5	6	13	6	3	13	2	9	3	2	1	6	11	11	12	10	7	6	8	8	8	7	12	7	7	12	7	2	8	9	2	5	6	1.100	0.519	–	–	L
M	0	0	0	0	2	2	0	3	3	5	1	0	4	1	1	2	2	1	1	4	4	4	6	9	10	0	2	0	4	1	0	1	1	5	1	1	0	2	0	0	0	0	0	1	0	1	0	1	0	2	3	0.587	0.755	3.46E-03	n.s.	M
N	1	3	5	2	2	2	5	3	2	1	6	4	2	8	6	6	1	2	0	3	5	3	6	5	7	3	4	3	0	2	3	1	1	2	7	4	1	3	1	4	2	3	4	4	2	8	2	2	4	6	2	1.660	1.017	–	–	N
P	2	2	2	6	7	3	9	6	1	5	2	1	0	1	6	2	3	3	6	8	3	2	6	3	5	8	5	5	2	5	3	3	3	1	5	1	3	4	3	4	8	1	3	4	2	3	3	7	2	2	8	0.460	0.274	2.57E-06	*	P
Q	2	5	1	3	2	6	3	2	0	4	1	4	3	1	0	4	2	1	0	2	4	3	4	4	1	3	1	2	1	1	1	4	0	0	1	5	4	3	3	1	1	6	2	3	6	2	2	2	1	1	1	2.360	1.651	–	–	Q
R	3	7	3	4	1	5	4	4	5	7	7	7	2	3	35	2	0	5	3	0	1	0	1	4	4	1	3	2	0	1	1	3	3	7	2	2	3	0	0	2	4	1	7	4	5	5	8	7	6	2	2	1.960	2.519	–	–	R
S	4	5	2	2	9	8	8	5	2	5	9	13	4	3	10	11	7	2	4	4	2	6	7	9	5	4	2	2	12	6	4	2	4	5	1	7	9	8	13	8	9	3	10	5	2	6	7	5	6	7	9	0.651	0.347	4.94E-01	n.s.	S
T	3	2	5	2	3	4	3	9	4	3	4	3	3	6	1	4	1	1	1	0	3	7	4	3	1	3	4	7	3	6	6	3	1	2	2	6	4	3	2	9	6	9	2	2	5	5	4	2	8	3	3	1.247	0.743	–	–	T
V	3	7	4	6	4	0	2	5	2	2	4	2	6	6	5	1	4	4	1	4	1	3	1	2	9	7	2	1	5	3	3	3	3	4	6	4	3	5	0	3	6	4	5	3	6	3	5	1	2	1	2	1.760	0.996	–	–	V
W	1	0	1	2	1	0	2	1	0	1	0	0	0	0	1	1	0	2	3	2	1	0	1	1	0	2	1	1	2	1	2	3	0	2	0	0	2	0	1	1	0	0	0	1	0	0	0	0	0	2	0	–	–	–	–	W
Y	4	4	3	2	5	0	2	4	0	4	0	0	2	3	6	1	5	1	2	1	3	6	1	1	1	1	4	5	3	3	6	0	4	1	3	0	2	6	3	7	2	0	1	0	4	3	3	3	7	0	3	0.880	0.679	–	–	Y
total	59	83	60	54	69	67	84	70	43	71	67	66	57	60	112	57	48	44	54	59	72	63	68	84	75	67	57	67	58	61	69	64	69	56	64	62	59	70	54	81	94	67	71	73	82	65	68	68	71	60	91	0.730	0.132			total

position -1

	C50	C49	C48	C47	C46	C45	C44	C43	C42	C41	C40	C39	C38	C37	C36	C35	C34	C33	C32	C31	C30	C29	C28	C27	C26	C25	C24	C23	C22	C21	C20	C19	C18	C17	C16	C15	C14	C13	C12	C11	C10	C9	C8	C7	C6	C5	C4	C3	C2	C1	C0	C-index	SD	*P-value*	mark	
A	0	5	4	3	2	2	8	4	3	5	1	1	2	4	3	6	3	2	9	3	6	3	2	12	7	2	2	5	3	7	5	4	8	3	1	3	0	4	6	10	5	3	8	5	2	1	3	2	3	1	6	0.653	0.435	1.84E-01	n.s.	A
C	0	1	0	1	1	1	1	1	1	1	0	5	1	1	1	0	0	0	1	2	0	2	1	1	2	0	2	0	4	3	1	0	2	2	4	1	1	1	1	0	1	0	1	0	5	3	0	1	2	1	0	–	–	–	–	C
D	4	5	2	4	4	1	15	4	4	3	3	4	2	2	4	4	5	3	2	3	3	4	2	1	4	6	1	3	4	2	5	4	2	2	7	1	7	1	3	7	7	3	4	6	2	5	2	3	10	3	7	0.549	0.353	5.02E-06	*	D
E	3	7	2	2	5	8	3	5	2	6	6	2	2	6	3	2	0	4	1	5	4	3	6	1	1	2	8	5	2	2	2	2	3	6	8	7	7	7	1	2	5	6	6	2	8	4	4	5	4	2	9	0.442	0.249	8.06E-07	*	E
F	2	7	3	2	4	5	3	6	1	3	5	3	1	1	1	3	2	1	5	3	4	4	1	4	1	4	3	4	4	3	4	3	3	4	5	4	5	4	2	4	7	1	4	0	4	3	6	4	2	2	3	1.093	0.539	–	–	F
G	2	5	2	1	4	5	3	6	2	3	1	1	1	1	4	1	3	2	1	3	4	4	6	4	5	3	0	4	4	2	1	4	2	3	1	1	2	2	2	7	4	3	3	4	2	8	4	4	2	6	4	0.760	0.440	–	–	G
H	3	6	4	2	3	0	0	2	0	0	5	8	2	0	7	3	3	3	0	0	1	2	3	3	1	1	3	2	1	2	5	4	2	1	0	1	3	5	2	3	9	3	2	3	4	1	6	3	1	3	1	2.620	2.098	–	–	H
I	5	6	3	2	3	1	6	4	0	4	3	2	7	0	8	3	4	3	3	2	6	2	2	0	3	2	1	1	4	4	3	2	0	3	6	2	1	4	6	3	2	6	7	3	3	4	7	5	2	5	1	3.360	2.028	–	–	I
K	6	6	3	4	6	7	4	2	1	3	1	1	2	3	2	2	3	2	2	4	2	4	2	5	5	3	2	1	2	3	1	5	4	3	0	4	3	4	2	4	7	4	3	7	8	5	7	5	3	2	5	0.696	0.377	9.00E-01	n.s.	K
L	6	2	5	4	3	7	9	4	7	10	6	6	7	3	10	4	5	3	6	10	9	7	10	15	15	10	3	11	7	3	6	11	11	7	1	5	4	8	2	6	8	12	6	3	6	7	5	9	6	5	11	0.609	0.291	2.02E-02	n.s.	L
M	1	1	0	0	0	1	1	1	0	2	6	0	1	1	0	0	1	1	2	2	6	2	2	1	0	3	0	4	0	6	2	0	0	0	3	1	0	4	4	2	4	1	3	3	0	0	3	3	0	2	1	1.600	1.702	–	–	M
N	1	3	2	2	3	4	0	2	1	2	4	2	2	0	4	2	1	0	0	1	5	2	2	3	2	4	2	2	1	4	4	3	3	2	1	1	2	3	1	1	3	0	0	1	2	1	4	5	5	2	5	0.428	0.280	1.40E-06	*	N
P	3	4	2	1	2	3	4	2	3	1	0	2	0	2	1	3	4	1	2	2	2	0	3	17	5	2	3	2	3	3	6	2	1	2	6	5	3	1	1	2	3	5	2	0	2	4	2	1	2	3	4	0.675	0.629	1.11E-01	n.s.	P
Q	3	9	3	2	4	6	5	2	4	0	3	3	3	1	4	4	2	1	2	1	1	4	7	2	4	0	5	5	1	1	3	0	3	0	0	2	2	6	2	3	6	5	2	2	4	4	0	1	6	0	3	0.953	0.693	–	–	Q
R	5	4	3	4	2	0	3	1	1	2	2	4	2	1	4	1	4	3	1	2	3	3	4	1	3	3	1	2	2	1	7	7	3	1	1	2	1	3	2	7	7	3	1	4	2	3	2	4	4	4	4	0.700	0.429	9.84E-01	n.s.	R
S	4	5	3	7	3	6	9	8	4	11	6	11	7	11	9	6	2	6	7	5	7	3	3	5	11	7	5	5	6	6	7	2	6	9	10	6	7	8	4	7	4	1	5	2	11	5	4	3	8	4	7	0.860	0.375	–	–	S
T	5	3	3	1	7	3	4	4	3	4	9	4	4	4	35	7	1	0	3	6	3	5	3	3	1	4	6	6	2	2	3	4	6	3	3	6	2	3	6	2	5	2	4	6	4	0	1	3	2	4	12	0.357	0.401	5.55E-08	*	T
V	4	1	10	9	9	6	6	9	3	3	4	5	5	11	11	6	4	8	2	4	4	8	5	3	2	8	5	3	3	3	1	2	7	4	1	2	5	0	3	6	4	7	5	8	2	4	2	3	5	8	7	0.694	0.392	5.42E-01	n.s.	V
W	0	0	1	0	2	1	0	1	2	5	0	1	1	1	0	0	1	1	0	0	1	1	1	0	2	1	0	1	1	1	1	2	0	0	1	4	0	0	1	1	0	0	5	10	8	2	2	1	2	1	1	1.340	1.965	–	–	W
Y	2	3	5	3	2	0	0	2	1	3	2	1	5	7	1	0	0	0	5	1	1	0	3	3	1	2	5	1	4	3	2	3	3	1	5	4	4	2	3	4	3	2	0	4	3	1	4	3	2	2	0	–	–	–	–	Y
total	59	83	60	54	69	67	84	70	43	71	67	66	57	60	112	57	48	44	54	59	72	63	68	84	75	67	57	67	58	61	69	64	69	56	64	62	59	70	54	81	94	67	71	73	82	65	68	68	71	60	91	0.730	0.132			total

Additional file 5. Continued.

ZEBRAFISH x-x-S-x-L

position -4

	C50	C49	C48	C47	C46	C45	C44	C43	C42	C41	C40	C39	C38	C37	C36	C35	C34	C33	C32	C31	C30	C29	C28	C27	C26	C25	C24	C23	C22	C21	C20	C19	C18	C17	C16	C15	C14	C13	C12	C11	C10	C9	C8	C7	C6	C5	C4	C3	C2	C1	C0	C-index	SD	*P-value*	mark	
A	7	7	6	9	14	6	5	10	14	16	11	5	15	9	8	8	7	7	7	10	7	7	6	3	7	13	16	4	14	9	11	7	6	10	4	10	8	8	7	12	10	5	3	5	10	3	9	10	4	1	18	0.456	0.196	5.79E-08	*	A
C	2	5	2	6	2	8	4	2	4	2	12	5	2	3	8	2	5	4	10	9	3	2	6	2	3	3	1	6	28	7	1	5	4	2	2	4	1	2	2	1	1	3	4	5	2	3	2	4	4	4	8	0.535	0.526	1.56E-06	*	C
D	9	8	5	11	10	11	8	6	7	6	7	7	8	15	10	9	4	11	3	7	11	6	10	3	4	7	5	8	10	5	8	4	6	7	4	10	4	5	5	7	3	7	4	13	6	9	8	16	34	6	4	1.985	1.196	–	–	D
E	6	13	15	7	7	35	5	10	8	6	4	9	9	8	15	8	16	8	13	13	10	7	2	5	8	7	3	6	5	9	7	8	10	8	9	5	7	4	10	2	4	3	8	6	5	9	4	9	6	9	10	0.820	0.504	–	–	E
F	2	1	5	9	7	3	5	6	8	1	5	11	12	4	8	18	4	5	6	9	9	5	10	7	9	6	4	2	6	4	6	1	12	6	3	7	12	7	5	7	10	6	3	12	11	5	9	4	4	6	13	0.503	0.264	4.68E-06	*	F
G	9	6	7	9	11	12	8	7	9	11	7	7	13	10	11	18	8	8	4	12	9	21	8	11	12	7	8	8	5	6	4	7	5	5	10	7	7	7	7	6	4	8	6	4	6	2	5	5	5	7	9	0.887	0.381	–	–	G
H	3	2	2	2	2	4	5	3	5	6	3	7	0	3	1	5	6	4	8	2	3	7	6	3	7	1	6	3	6	1	2	3	3	3	4	3	5	2	5	5	4	3	3	3	3	7	5	3	4	2	2	1.880	0.923	–	–	H
I	2	8	6	4	4	8	8	8	10	7	12	22	11	15	7	12	7	9	12	6	3	6	9	5	6	8	11	3	8	6	7	6	6	13	16	12	7	6	6	5	11	10	8	5	10	14	12	14	9	11	4	2.155	0.947	–	–	I
K	5	5	3	11	6	4	5	3	7	3	5	4	3	5	14	12	9	6	6	6	6	9	2	9	16	6	6	2	15	8	6	6	6	4	3	3	5	8	5	9	7	12	6	4	8	12	9	6	8	8	14	0.480	0.234	8.50E-08	*	K
L	16	23	13	10	11	18	17	10	21	23	20	19	21	20	15	16	19	23	11	18	11	11	14	16	12	15	11	13	10	19	23	10	13	18	20	16	21	8	17	15	12	20	19	20	17	24	16	19	12	13	11	1.471	0.389	–	–	L
M	2	4	9	9	4	3	10	2	2	2	1	2	2	4	6	3	2	6	3	3	3	1	3	5	1	2	0	2	2	4	1	1	2	5	3	3	8	3	3	1	6	5	3	2	3	3	4	5	4	1	8	0.420	0.275	5.77E-08	*	M
N	7	4	1	5	4	2	6	3	6	1	6	7	7	3	1	6	4	7	4	6	6	3	2	5	4	9	3	5	8	3	5	2	5	6	3	4	3	4	7	11	5	8	2	6	5	3	11	10	5	6	8	0.623	0.300	1.41E-01	n.s.	N
P	8	9	11	7	4	1	4	3	4	10	8	5	8	9	10	5	6	2	8	8	6	3	10	8	13	11	17	7	9	6	12	10	1	3	6	17	13	5	2	10	10	11	6	19	7	10	7	8	10	10	14	0.567	0.281	1.13E-03	n.s.	P
Q	8	5	6	4	7	11	5	8	4	3	27	9	2	6	5	3	5	5	9	1	6	7	6	2	8	12	4	4	4	6	10	4	4	2	5	8	10	7	16	4	8	4	5	3	5	4	7	8	4	2	12	0.520	0.346	3.22E-07	*	Q
R	9	5	5	7	1	5	8	8	12	10	5	5	13	6	6	9	6	3	7	4	9	4	4	9	9	9	6	6	7	6	11	10	7	6	9	6	7	7	16	9	4	5	9	5	9	9	6	9	7	8	14	0.517	0.191	5.73E-08	*	R
S	18	20	15	15	20	11	22	18	10	19	17	20	31	11	18	16	18	20	24	15	16	17	23	21	12	16	17	12	4	18	23	16	15	18	20	9	17	9	10	17	10	17	15	6	14	13	9	14	13	14	23	0.690	0.214	9.96E-01	n.s.	S
T	9	10	5	5	4	10	5	16	11	8	8	13	10	9	4	5	11	4	7	8	7	11	7	13	8	7	8	3	7	15	3	6	5	12	4	8	5	9	4	6	8	13	13	5	11	3	12	14	4	10	13	0.620	0.265	1.01E-01	n.s.	T
V	6	10	20	6	12	14	7	7	10	8	9	12	5	7	11	12	7	9	2	16	19	10	13	8	7	11	7	11	13	7	11	12	7	10	5	6	4	7	4	10	13	4	5	5	12	11	9	8	10	7	10	0.912	0.370	–	–	V
W	0	1	0	2	0	2	3	0	1	1	2	0	9	3	1	1	1	0	1	0	1	1	0	1	2	2	5	0	1	8	0	3	1	1	2	0	1	0	2	1	1	4	2	2	1	1	5	0	1	0	2	0.770	0.943	–	–	W
Y	8	2	6	4	8	1	3	4	8	1	2	4	18	3	1	9	1	5	10	6	4	6	4	3	3	4	5	3	1	1	3	1	3	3	7	6	6	4	3	5	4	6	4	5	15	2	2	2	2	5	7	0.646	0.477	4.71E-02	n.s.	Y
total	136	148	142	142	138	169	143	134	161	144	171	173	199	153	160	177	146	146	155	159	149	144	145	139	151	156	143	108	163	148	154	122	121	142	139	144	151	112	136	143	135	154	128	135	160	147	151	168	150	130	204	0.722	0.079			total

position -3

	C50	C49	C48	C47	C46	C45	C44	C43	C42	C41	C40	C39	C38	C37	C36	C35	C34	C33	C32	C31	C30	C29	C28	C27	C26	C25	C24	C23	C22	C21	C20	C19	C18	C17	C16	C15	C14	C13	C12	C11	C10	C9	C8	C7	C6	C5	C4	C3	C2	C1	C0	C-index	SD	*P-value*	mark	
A	10	12	13	9	8	32	12	11	12	4	10	12	12	3	9	8	3	9	3	7	12	10	9	7	9	13	8	5	2	7	15	5	9	9	9	9	13	9	3	7	7	13	7	9	12	4	7	10	12	2	7	1.291	0.666	–	–	A
C	2	4	2	4	0	6	1	1	4	2	3	5	5	10	3	3	3	1	15	10	2	5	4	2	3	2	2	1	4	4	3	1	7	5	3	3	3	6	2	3	1	8	1	2	2	5	0	0	2	0	10	0.340	0.284	5.55E-08	*	C
D	9	5	4	5	5	5	5	6	8	6	3	7	11	8	7	8	9	6	5	8	4	14	6	5	9	14	5	6	0	7	5	4	3	6	9	5	9	4	10	7	5	10	4	7	11	4	17	11	8	7	18	0.384	0.173	5.55E-08	*	D
E	6	1	11	5	4	6	3	5	8	6	6	6	13	9	8	6	8	8	8	1	6	3	6	7	8	4	4	6	7	6	9	3	6	10	8	6	3	9	7	4	5	8	6	4	6	10	3	5	10	5	26	0.240	0.095	5.55E-08	*	E
F	4	5	9	3	4	6	4	9	7	7	2	6	10	12	10	8	6	7	4	7	7	8	4	4	6	9	4	3	3	10	10	10	9	9	4	11	6	3	7	5	1	10	7	10	11	11	9	9	8	4	8	0.855	0.350	–	–	F
G	4	12	12	8	7	6	6	10	11	11	8	6	8	9	9	15	10	9	11	11	9	15	11	3	13	12	4	2	9	9	6	4	5	6	5	10	5	6	4	5	7	4	11	10	9	9	4	10	3	5	6	1.327	0.536	–	–	G
H	3	1	2	2	2	7	2	5	5	5	3	8	3	4	3	4	0	3	2	0	1	2	9	5	4	0	6	4	3	1	8	3	6	8	1	2	4	1	3	7	5	6	3	9	6	2	10	1	5	3	13	0.295	0.196	5.55E-08	*	H
I	7	7	12	11	5	14	12	5	11	9	6	11	3	5	5	15	5	13	4	10	4	5	12	8	2	4	19	5	10	11	7	10	5	5	6	7	6	7	17	12	7	14	4	6	9	3	11	14	7	6	5	1.652	0.788	–	–	I
K	6	7	4	13	7	6	8	6	5	7	7	7	15	5	6	8	1	7	7	6	7	8	5	10	8	6	5	2	7	4	6	1	1	7	6	8	11	6	8	5	9	4	6	10	6	10	15	7	6	7	15	0.452	0.192	5.55E-08	*	K
L	11	19	14	16	18	15	17	11	12	15	12	27	22	17	16	18	16	18	11	25	20	15	12	11	15	17	12	19	23	17	14	10	11	16	17	19	20	12	16	14	15	18	15	7	11	19	13	17	8	26	15	1.052	0.288	–	–	L
M	0	3	1	5	3	6	6	2	2	3	2	2	0	7	6	2	9	1	2	8	0	3	5	8	6	0	8	4	4	1	3	6	3	2	10	3	2	2	6	4	6	2	5	4	8	1	3	3	5	2	1	3.780	2.542	–	–	M
N	8	14	4	1	9	6	8	8	7	5	12	10	8	3	12	4	8	5	4	6	11	4	9	10	3	8	7	8	30	5	8	4	7	10	1	3	8	5	6	4	7	7	1	6	6	10	4	12	6	6	13	0.551	0.341	8.92E-06	*	N
P	6	3	5	4	9	8	5	10	7	7	9	10	8	10	18	9	11	6	19	14	11	5	5	12	9	8	3	9	12	5	8	10	3	12	7	7	7	3	14	11	6	9	9	5	3	4	13	10	7	9	6	1.380	0.605	–	–	P
Q	12	4	4	11	11	5	5	3	6	10	2	5	20	2	7	10	7	7	2	3	9	6	10	6	6	10	4	6	3	5	11	5	3	7	4	8	3	5	1	13	4	8	8	9	19	9	4	5	5	10	9	0.760	0.438	–	–	Q
R	8	10	4	9	7	7	5	3	10	7	12	3	11	6	4	5	7	12	10	8	11	4	6	4	8	9	5	0	8	7	2	6	12	8	20	4	7	7	5	7	14	5	5	9	6	6	12	12	5	1	11	0.660	0.327	2.29E-01	n.s.	R
S	15	17	15	16	24	9	17	16	24	10	23	20	30	26	10	21	16	18	13	11	12	18	16	17	16	18	16	14	13	14	16	18	15	9	16	11	20	8	13	14	14	13	14	11	10	12	3	17	12	12	13	1.174	0.374	–	–	S
T	6	9	9	10	0	5	12	3	9	8	11	16	12	3	11	12	7	4	7	7	2	7	1	9	6	7	7	6	4	16	7	4	5	5	4	7	6	4	2	8	11	3	5	5	6	11	4	6	6	2	11	0.613	0.322	1.81E-02	n.s.	T
V	11	11	12	4	10	14	10	9	6	16	11	8	6	8	12	12	15	7	14	7	11	11	6	7	8	5	16	6	14	14	8	14	4	8	5	12	5	11	10	9	9	8	9	8	11	12	9	16	5	9	11	0.878	0.299	–	–	V
W	1	1	1	3	0	1	1	1	0	1	25	1	1	4	1	1	1	3	3	1	1	0	2	1	4	5	2	1	2	3	1	0	5	0	1	0	5	1	2	1	0	3	1	1	2	0	2	0	26	5	2	1.280	2.471	–	–	W
Y	7	3	4	3	5	5	4	10	7	5	4	3	1	2	3	8	4	2	11	9	9	1	7	3	8	5	6	1	5	2	7	4	2	0	3	9	8	3	0	3	2	1	7	3	6	5	8	3	4	9	4	1.170	0.699	–	–	Y
total	136	148	142	142	138	169	143	134	161	144	171	173	199	153	160	177	146	146	155	159	149	144	145	139	151	156	143	108	163	148	154	122	121	142	139	144	151	112	136	143	135	154	128	135	160	147	151	168	150	130	204	0.722	0.079			total

position -1

	C50	C49	C48	C47	C46	C45	C44	C43	C42	C41	C40	C39	C38	C37	C36	C35	C34	C33	C32	C31	C30	C29	C28	C27	C26	C25	C24	C23	C22	C21	C20	C19	C18	C17	C16	C15	C14	C13	C12	C11	C10	C9	C8	C7	C6	C5	C4	C3	C2	C1	C0	C-index	SD	*P-value*	mark	
A	8	12	8	10	6	4	9	10	12	17	30	16	8	13	13	9	11	14	14	20	9	11	12	10	15	8	12	10	13	13	10	10	7	10	3	9	8	6	8	9	9	14	14	10	12	12	8	11	9	7	14	0.776	0.301	–	–	A
C	4	6	10	8	3	4	6	3	5	5	0	8	2	3	4	9	5	2	7	1	5	5	2	2	1	3	0	1	6	3	3	2	6	2	0	3	5	0	5	0	1	2	2	4	0	2	0	2	4	8	6	0.580	0.428	1.06E-01	n.s.	C
D	7	8	5	4	14	7	13	8	3	8	5	12	3	9	6	9	11	3	6	9	4	9	4	7	5	8	6	9	4	9	8	7	4	3	5	6	6	4	8	6	6	10	5	8	11	7	9	3	10	8	16	0.436	0.169	5.55E-08	*	D
E	10	7	5	5	4	9	8	9	8	17	7	10	6	4	7	7	11	5	8	9	13	4	6	10	6	8	1	5	9	10	10	11	8	7	6	8	14	10	7	8	9	3	3	9	8	10	2	7	7	5	10	0.760	0.299	–	–	E
F	4	7	4	4	7	5	5	4	4	9	6	3	6	7	6	7	2	5	7	5	4	2	4	6	6	6	6	6	6	2	4	9	8	6	12	8	7	7	10	12	10	7	2	4	6	10	7	12	8	3	9	0.682	0.282	6.73E-01	n.s.	F
G	1	6	12	8	8	10	11	9	10	4	10	7	9	4	12	7	5	5	4	10	9	10	15	4	14	7	2	4	9	4	5	5	6	13	6	3	10	4	7	2	5	11	6	8	11	8	15	9	6	6	4	1.880	0.850	–	–	G
H	1	4	13	6	8	3	3	2	9	2	6	4	3	3	4	4	6	6	6	2	4	3	5	5	6	2	1	2	7	3	3	3	5	7	10	5	3	4	6	5	3	7	5	4	2	4	3	8	2	0	2	2.220	1.234	–	–	H
I	5	6	6	7	6	6	3	7	4	3	4	3	9	7	4	7	9	4	10	7	4	17	3	8	4	5	11	1	4	15	6	4	5	10	9	6	2	9	5	7	12	8	3	7	8	5	10	7	4	6	8	0.805	0.393	–	–	I
K	9	8	10	5	5	12	7	4	6	5	3	9	15	5	11	13	9	9	2	5	7	5	6	5	4	7	4	5	8	3	5	3	6	5	13	7	5	3	3	7	3	7	8	9	7	4	16	5	16	10	29	0.240	0.119	5.55E-08	*	K
L	21	12	8	15	16	12	13	17	14	12	15	24	21	29	18	30	13	16	7	23	14	9	13	10	19	16	19	13	9	19	29	13	7	15	9	6	21	5	12	20	8	14	14	15	15	16	14	20	13	16	15	1.012	0.380	–	–	L
M	3	1	3	3	3	4	2	2	6	1	4	1	9	3	1	4	4	6	2	5	2	3	3	4	4	7	8	5	2	4	1	2	2	6	6	1	4	3	6	2	2	1	3	3	2	3	4	4	29	6	4	0.995	1.020	–	–	M
N	3	5	7	4	2	9	6	13	8	7	12	9	2	7	4	7	2	2	7	6	9	2	2	4	6	10	8	3	3	2	7	3	3	9	6	4	3	6	0	9	7	6	3	1	4	6	4	2	7	3	11	0.480	0.267	1.63E-06	*	N
P	3	8	9	8	8	6	5	5	10	8	5	5	15	12	8	7	14	14	13	9	9	9	10	5	5	7	5	6	5	9	9	8	12	9	7	4	5	4	11	5	7	8	8	9	2	4	4	14	4	9	0	–	–	–	–	P
Q	9	5	4	8	3	3	5	4	5	6	8	5	12	3	6	13	7	8	6	8	5	5	4	0	7	4	7	3	8	3	5	8	4	3	6	4	13	7	3	4	4	8	5	5	6	1	7	8	2	6	10	0.566	0.269	8.05E-04	n.s.	Q
R	11	5	13	9	9	45	10	8	11	6	6	10	20	10	7	7	5	9	6	6	8	4	8	22	7	13	6	13	28	5	12	4	3	6	8	6	7	8	7	11	8	5	8	8	8	13	8	7	5	4	11	0.860	0.625	–	–	R
S	7	14	14	16	11	16	17	8	14	12	16	24	40	9	24	15	9	19	27	10	15	23	17	15	15	20	19	10	16	22	17	9	12	11	15	34	14	11	23	16	15	22	24	18	27	20	22	15	11	13	26	0.648	0.251	2.37E-02	n.s.	S
T	12	16	3	6	8	7	3	7	13	5	10	9	4	11	7	9	6	6	9	9	3	10	14	6	9	8	5	3	10	6	5	5	5	5	7	14	13	9	5	6	7	6	5	9	11	5	8	12	5	3	9	0.842	0.359	–	–	T
V	14	14	5	10	11	4	11	8	11	10	9	12	12	8	15	11	13	8	10	6	22	8	11	13	13	14	18	4	9	9	7	12	11	9	8	12	11	8	6	10	13	10	4	2	14	12	8	10	4	6	14	0.714	0.266	1.00E+00	n.s.	V
W	2	0	0	2	0	1	1	1	4	5	3	1	0	2	1	0	0	3	0	4	1	1	1	1	1	3	1	0	4	3	1	0	2	3	0	2	0	3	0	0	2	0	3	0	1	2	1	7	2	5	3	0.533	0.539	5.12E-03	n.s.	W
Y	2	4	3	4	6	2	5	5	4	2	12	1	3	4	2	2	4	2	4	5	2	4	5	2	4	0	4	5	3	4	7	4	5	3	3	2	0	1	4	4	4	5	3	2	5	3	1	5	2	6	3	1.187	0.657	–	–	Y
total	136	148	142	142	138	169	143	134	161	144	171	173	199	153	160	177	146	146	155	159	149	144	145	139	151	156	143	108	163	148	154	122	121	142	139	144	151	112	136	143	135	154	128	135	160	147	151	168	150	130	204	0.722	0.079			total

Additional file 5. Continued.

ZEBRAFISH x-x-S-x-V

position -4

	C50	C49	C48	C47	C46	C45	C44	C43	C42	C41	C40	C39	C38	C37	C36	C35	C34	C33	C32	C31	C30	C29	C28	C27	C26	C25	C24	C23	C22	C21	C20	C19	C18	C17	C16	C15	C14	C13	C12	C11	C10	C9	C8	C7	C6	C5	C4	C3	C2	C1	C0	C-index	SD	*P-value*	mark	
A	5	6	7	10	5	7	11	8	6	1	2	1	5	7	8	7	3	3	3	6	7	7	8	7	1	20	5	6	5	7	9	17	10	4	11	3	7	5	4	2	3	5	4	2	15	6	5	1	6	3	9	0.680	0.429	–	–	A
C	2	1	6	5	1	3	0	1	1	7	4	0	5	2	2	1	1	4	2	1	3	1	2	4	1	3	5	1	1	2	1	1	1	0	1	2	1	6	4	0	0	0	1	1	4	1	1	2	2	4	2	1.050	0.888	–	–	C
D	2	4	2	3	6	5	3	4	1	5	6	4	5	5	8	1	4	5	7	4	0	2	3	3	3	4	4	2	6	6	1	7	8	8	6	0	8	9	5	5	8	10	5	4	7	4	3	1	5	6	18	0.252	0.132	5.55E-08	✻	D
E	1	11	3	5	6	5	8	7	5	6	4	3	5	5	3	4	3	4	10	3	1	2	11	8	3	6	2	3	4	3	2	2	10	4	6	1	4	7	5	3	5	4	9	4	4	2	4	14	6	2	5	0.968	0.569	–	–	E
F	3	2	6	3	3	1	2	3	2	5	1	5	6	2	5	0	4	4	1	0	2	4	5	3	1	4	18	6	2	2	0	2	2	3	5	3	2	5	1	7	3	2	4	7	1	1	6	2	9	2	4	0.860	0.729	–	–	F
G	4	6	8	9	6	6	4	4	8	5	8	4	8	10	3	6	6	8	2	7	7	6	6	4	4	3	5	17	4	5	7	6	5	5	9	5	4	7	10	7	5	2	3	2	7	14	5	1	2	2	4	1.455	0.742	–	–	G
H	6	3	1	5	2	2	3	0	2	2	0	5	2	2	1	2	3	0	2	1	2	1	2	0	1	1	3	1	4	1	5	3	2	3	3	3	1	3	6	1	2	2	3	1	4	4	1	2	4	2	7	0.329	0.213	1.42E-06	✻	H
I	4	7	4	5	7	13	3	9	3	6	3	8	5	5	2	1	5	6	6	4	3	4	7	0	5	4	2	7	6	1	2	7	2	5	5	4	3	4	4	2	2	2	2	4	1	18	8	6	0	0	8	0.565	0.401	–	–	I
K	3	5	4	11	2	6	6	6	0	7	3	4	1	4	6	3	4	7	4	9	2	7	4	4	4	7	3	2	8	9	6	7	5	4	2	3	3	3	3	5	3	3	2	4	11	2	3	4	9	4	12	0.385	0.205	3.44E-04	n.s.	K
L	9	11	5	15	6	14	10	9	7	5	7	6	10	4	13	9	9	3	5	9	8	8	6	6	6	7	9	5	6	3	10	8	6	12	10	9	13	7	8	6	6	14	9	11	8	6	8	4	14	8	15	0.543	0.197	–	–	L
M	1	3	2	2	0	1	2	2	1	1	0	5	1	5	1	3	1	4	1	1	0	2	1	4	1	4	1	0	1	1	3	1	0	5	4	2	6	1	1	5	3	1	0	0	0	0	2	4	3	1	6	0.313	0.273	1.77E-04	n.s.	M
N	1	3	2	2	2	1	3	4	7	2	4	3	3	4	7	2	2	6	3	4	6	4	2	4	3	4	5	4	7	4	4	4	3	3	2	4	5	3	3	4	11	3	5	8	2	5	10	1	2	2	6	0.640	0.354	–	–	N
P	5	3	6	4	5	4	3	3	4	6	8	3	2	1	5	5	9	3	2	6	2	5	4	2	4	6	5	6	2	6	8	3	7	4	4	10	3	2	6	4	4	2	7	1	6	10	4	13	4	11	14	0.346	0.186	2.87E-07	✻	P
Q	2	3	4	4	25	2	4	2	4	3	4	2	3	3	3	1	5	5	3	6	3	3	5	5	0	3	6	2	5	0	6	4	4	2	3	2	2	1	4	1	0	3	1	4	3	4	5	4	10	4	6	0.623	0.593	–	–	Q
R	0	7	11	3	5	2	6	2	4	2	4	4	5	2	7	4	1	3	3	4	2	3	6	2	6	4	7	2	1	6	4	3	7	1	1	4	4	4	3	7	3	6	22	4	1	3	2	1	2	9	11	0.380	0.312	9.26E-05	✻	R
S	9	3	10	11	12	13	8	5	5	9	10	11	12	11	9	11	4	7	10	12	9	9	6	12	7	14	15	2	13	3	11	4	10	18	7	12	12	11	7	11	9	11	6	6	4	10	5	26	10	15	21	0.454	0.200	4.10E-01	n.s.	S
T	8	12	8	2	16	12	12	5	7	12	2	6	0	2	9	6	5	2	5	6	8	6	4	7	8	3	1	5	2	5	2	3	6	5	7	3	7	7	5	4	6	8	2	7	5	2	10	3	8	11	14	0.424	0.243	3.42E-02	n.s.	T
V	7	8	7	5	7	8	7	7	6	10	3	7	5	9	6	6	5	5	3	7	6	9	22	4	2	8	5	6	4	3	3	3	7	5	10	5	4	3	5	3	2	8	4	4	7	9	6	4	3	1	9	0.651	0.355	–	–	V
W	0	1	0	1	2	2	0	1	0	0	0	3	0	0	1	0	0	0	2	0	2	0	1	0	4	2	3	0	0	1	0	1	3	2	0	1	0	0	0	0	1	1	1	0	1	0	0	1	0	0	2	0.380	0.511	2.47E-02	n.s.	W
Y	1	3	2	1	2	3	3	1	2	3	4	0	3	1	2	2	1	3	4	1	2	2	3	0	5	2	3	2	2	1	1	3	3	1	3	1	1	3	4	6	2	1	2	0	1	2	11	5	4	1	2	1.190	0.909	–	–	Y
total	73	102	98	106	120	110	98	83	75	97	77	84	86	84	101	74	75	82	78	91	75	85	108	79	69	109	107	79	83	69	85	89	101	94	99	77	90	91	88	83	78	88	92	74	92	103	99	99	103	88	175	0.511	0.070			total

position -3

	C50	C49	C48	C47	C46	C45	C44	C43	C42	C41	C40	C39	C38	C37	C36	C35	C34	C33	C32	C31	C30	C29	C28	C27	C26	C25	C24	C23	C22	C21	C20	C19	C18	C17	C16	C15	C14	C13	C12	C11	C10	C9	C8	C7	C6	C5	C4	C3	C2	C1	C0	C-index	SD	*P-value*	mark	
A	7	5	3	9	6	3	7	3	7	2	4	2	4	7	9	8	0	4	8	5	4	2	6	0	4	10	3	8	4	4	7	5	5	7	2	8	1	7	5	9	5	6	2	2	8	5	4	12	7	7	8	0.655	0.333	–	–	A
C	5	5	4	3	1	6	3	2	3	2	2	1	1	0	1	3	0	0	0	5	2	0	0	0	2	0	8	1	5	3	0	4	0	3	1	2	2	1	1	0	1	0	2	4	1	2	0	1	2	4	6	0.330	0.313	3.66E-04	n.s.	C
D	2	9	6	6	8	4	1	2	5	5	2	3	6	5	3	3	5	5	7	7	4	3	6	6	5	4	5	3	5	4	1	2	6	5	10	4	3	4	3	5	4	4	1	3	3	3	3	10	3	3	16	0.274	0.131	5.55E-08	✻	D
E	6	6	5	2	4	1	6	7	5	8	8	4	2	3	3	3	5	7	5	4	4	9	5	10	5	6	8	5	6	4	3	9	12	5	6	4	6	2	6	5	8	5	5	3	2	5	4	3	2	5	23	0.223	0.098	5.55E-08	✻	E
F	2	3	6	1	2	8	3	6	7	3	1	9	4	3	2	2	5	3	2	4	1	4	2	9	1	3	4	6	1	1	3	4	7	1	2	7	3	6	6	10	5	3	4	6	1	3	2	4	5	3	6	0.643	0.391	–	–	F
G	7	9	7	8	6	12	8	0	3	6	6	6	4	8	6	6	3	8	5	5	3	10	13	5	6	10	4	9	4	9	1	8	7	6	4	8	3	4	1	6	5	6	3	5	3	6	9	18	3	3	15	0.407	0.214	1.42E-03	n.s.	G
H	4	1	2	1	2	1	3	2	1	1	2	6	5	0	2	3	5	1	4	1	4	7	3	4	0	3	1	2	0	4	4	5	2	3	8	2	4	3	2	2	1	1	4	0	0	1	3	2	4	1	4	0.635	0.458	–	–	H
I	3	6	3	4	5	11	6	10	4	5	6	4	4	1	6	3	6	4	1	7	3	3	5	1	2	9	4	3	6	0	3	3	5	4	4	4	7	6	4	3	2	4	2	6	6	1	10	3	4	11	7	0.649	0.361	–	–	I
K	4	2	3	5	3	5	4	7	2	5	2	5	3	4	3	5	6	1	4	4	3	4	22	5	1	4	4	1	13	8	1	4	1	1	6	4	2	0	3	3	7	2	6	4	8	3	7	4	7	4	4	1.095	0.861	–	–	K
L	3	13	17	9	12	11	7	6	10	15	4	11	13	5	10	5	6	10	7	10	7	5	5	5	4	11	14	4	3	2	13	4	7	11	5	7	11	11	10	6	11	5	24	11	11	9	7	8	5	9	12	0.715	0.345	–	–	L
M	0	2	8	0	2	2	2	2	3	0	2	3	3	3	1	2	2	3	0	1	2	1	3	0	0	3	3	1	1	1	2	0	1	2	3	2	2	4	8	3	2	3	0	1	1	4	1	2	0	0	0	–	–	–	–	M
N	4	2	3	5	1	2	1	2	3	1	3	3	5	3	5	4	0	0	5	1	7	8	2	4	4	2	4	1	6	1	3	2	2	3	6	6	8	8	1	1	0	5	5	2	9	3	8	4	4	6	4	0.890	0.587	–	–	N
P	4	3	3	8	8	7	6	10	5	5	3	5	2	5	11	6	7	7	5	6	4	4	2	4	3	5	7	8	3	2	3	6	8	8	10	2	2	6	3	4	8	7	5	5	2	13	2	3	16	2	7	0.780	0.432	–	–	P
Q	1	7	5	5	4	6	8	2	1	5	5	3	1	1	10	2	2	3	6	3	2	3	1	3	5	6	2	2	4	0	5	4	6	9	2	3	5	1	2	1	1	1	4	3	2	6	5	0	7	5	5	0.720	0.471	–	–	Q
R	5	4	5	10	3	2	7	3	3	4	1	6	9	3	8	2	2	5	6	4	2	5	3	2	3	6	9	6	4	5	3	5	4	2	5	2	4	3	6	2	3	7	3	4	5	3	2	3	5	5	10	0.426	0.204	1.19E-02	n.s.	R
S	7	9	7	10	29	12	12	7	2	10	8	4	9	11	6	7	11	10	5	10	7	5	13	11	9	6	8	7	5	7	9	9	13	10	8	5	9	16	11	9	4	13	13	7	10	22	17	8	7	5	32	0.293	0.142	5.55E-08	✻	S
T	2	3	4	4	7	5	7	4	1	9	6	5	4	3	5	3	2	6	3	11	8	5	5	3	1	6	7	3	3	5	9	6	7	5	4	4	5	4	5	6	1	7	2	5	4	5	5	2	8	7	10	0.482	0.218	8.96E-01	n.s.	T
V	7	7	4	12	8	9	5	4	5	8	8	1	3	9	6	4	7	4	0	1	5	5	8	4	9	11	6	6	7	6	6	4	5	7	4	0	7	3	8	5	4	5	4	2	3	6	4	9	10	4	4	1.395	0.664	–	–	V
W	0	2	0	0	3	2	0	2	3	0	2	1	1	2	3	2	0	0	2	1	2	0	1	0	2	0	0	2	0	1	4	1	0	0	0	0	0	0	0	0	3	0	2	0	0	1	2	1	0	1	1	0.980	1.116	–	–	W
Y	0	4	3	4	6	1	2	2	2	3	2	2	3	8	1	1	1	1	3	1	1	2	3	3	3	4	6	1	3	2	5	4	3	2	9	3	6	2	3	3	3	4	1	1	13	2	4	2	4	3	1	3.100	2.297	–	–	Y
total	73	102	98	106	120	110	98	83	75	97	77	84	86	84	101	74	75	82	78	91	75	85	108	79	69	109	107	79	83	69	85	89	101	94	99	77	90	91	88	83	78	88	92	74	92	103	99	99	103	88	175	0.511	0.070			total

position -1

	C50	C49	C48	C47	C46	C45	C44	C43	C42	C41	C40	C39	C38	C37	C36	C35	C34	C33	C32	C31	C30	C29	C28	C27	C26	C25	C24	C23	C22	C21	C20	C19	C18	C17	C16	C15	C14	C13	C12	C11	C10	C9	C8	C7	C6	C5	C4	C3	C2	C1	C0	C-index	SD	*P-value*	mark	
A	10	15	7	13	31	6	6	6	4	11	5	10	7	6	12	4	2	6	6	10	6	7	28	7	4	9	6	8	11	7	6	7	9	7	5	12	4	5	7	9	8	13	25	4	13	7	6	7	16	5	10	0.890	0.577	–	–	A
C	3	3	2	1	2	5	3	5	2	6	2	4	4	1	2	1	3	3	3	0	3	1	3	1	1	1	1	6	2	2	0	0	1	3	2	3	2	0	2	0	2	2	4	0	12	0	4	2	3	1	0	–	–	–	–	C
D	8	14	3	6	5	5	4	1	1	2	5	0	4	3	7	6	5	3	6	3	4	6	9	1	4	7	7	5	2	6	5	3	5	0	9	2	7	6	2	4	5	3	5	2	0	5	6	3	7	2	11	0.405	0.241	3.72E-02	n.s.	D
E	2	3	4	5	5	8	3	5	4	7	4	3	6	6	6	1	8	4	2	4	6	2	2	8	2	5	5	4	5	5	1	5	6	6	9	6	8	4	4	7	5	6	7	5	6	9	5	2	7	3	13	0.377	0.156	1.88E-05	✻	E
F	1	3	10	1	11	1	2	2	1	1	3	2	3	3	2	2	3	3	4	7	4	4	2	2	4	1	3	2	1	2	1	2	1	5	6	3	1	6	4	2	2	2	3	4	5	6	6	3	4	5	6	0.537	0.365	–	–	F
G	2	7	6	5	1	8	8	6	8	5	3	5	6	1	6	3	6	10	4	8	10	2	7	2	6	13	10	7	5	5	5	2	10	10	4	4	7	1	2	4	5	2	7	6	1	4	1	1	2	3	3	1.707	0.984	–	–	G
H	1	2	0	7	0	3	6	5	4	1	0	1	2	1	0	0	2	2	2	1	2	4	3	0	2	3	0	3	1	1	1	0	1	1	3	3	7	1	1	4	3	1	2	0	2	1	2	0	4	5	8	0.253	0.227	6.03E-08	✻	H
I	2	5	2	2	4	1	0	4	5	10	7	2	2	6	4	2	3	1	0	4	1	7	0	5	1	1	3	1	6	1	0	6	2	7	6	2	2	5	3	3	3	8	1	4	5	12	2	5	3	2	12	0.288	0.218	8.75E-08	✻	I
K	3	4	5	5	2	12	12	4	3	6	3	3	2	4	1	5	3	3	5	3	0	2	6	1	3	2	6	1	2	7	5	3	3	4	4	3	5	6	6	7	5	5	6	8	11	3	2	7	3	3	6	0.723	0.431	–	–	K
L	7	8	8	9	4	11	7	9	11	5	7	6	8	8	8	6	5	8	4	11	6	3	2	12	10	13	6	7	7	3	9	6	6	11	10	4	8	7	6	5	5	8	5	6	10	7	10	6	4	4	14	0.509	0.180	1.00E+00	n.s.	L
M	0	1	3	3	3	2	2	1	1	3	3	4	4	0	2	3	0	1	6	3	1	2	3	2	2	2	1	0	0	1	3	0	2	1	1	1	4	2	3	2	3	3	1	3	6	0	4	4	0	1	2	1.030	0.745	–	–	M
N	0	2	5	4	3	2	4	2	3	1	0	2	1	3	1	6	0	3	5	2	3	1	1	1	4	7	0	0	1	2	9	4	3	0	6	1	2	2	1	4	2	0	2	1	2	0	4	2	2	1	7	0.334	0.282	5.27E-05	✻	N
P	6	4	7	3	1	9	10	5	3	11	3	3	1	5	3	4	6	7	8	5	5	4	5	9	2	1	6	3	8	2	6	10	6	7	8	3	5	3	6	2	5	5	0	1	3	3	6	4	9	7	5	0.992	0.530	–	–	P
Q	5	5	3	3	4	4	3	4	2	3	1	5	3	7	4	1	1	4	3	4	1	3	2	4	2	4	8	2	5	4	1	8	9	6	4	2	4	6	2	5	5	2	1	4	3	4	6	8	0	0	8	0.460	0.263	5.14E-01	n.s.	Q
R	7	5	10	2	8	7	4	3	3	1	2	2	4	7	5	1	4	2	5	4	4	5	4	2	3	2	10	8	5	4	4	5	7	2	5	7	4	3	6	2	4	2	3	3	2	19	2	6	3	3	5	0.900	0.604	–	–	R
S	7	6	11	19	15	9	11	8	7	9	14	18	12	13	22	9	11	8	5	8	3	17	13	9	11	17	18	7	5	8	15	8	8	10	7	6	7	18	18	14	8	10	9	11	4	9	16	26	16	14	33	0.342	0.150	1.74E-07	✻	S
T	8	5	4	7	9	8	8	6	4	7	6	7	8	4	6	11	5	7	3	9	9	8	8	6	4	6	7	7	11	4	3	10	12	5	7	4	7	9	9	4	2	8	6	6	4	10	8	4	6	13	13	0.522	0.189	–	–	T
V	0	6	3	6	11	7	3	3	6	7	4	5	7	4	7	6	4	3	3	3	4	3	6	2	2	14	5	3	5	3	7	7	7	4	1	5	4	4	2	2	4	6	5	3	3	3	5	8	1	6	12	0.387	0.207	2.14E-04	n.s.	V
W	0	3	2	3	1	1	0	2	1	0	2	1	0	0	3	2	2	0	3	1	2	2	1	1	0	0	0	1	1	1	1	2	2	2	1	2	0	0	3	0	0	1	0	0	0	0	1	1	10	6	4	0.340	0.434	1.33E-04	n.s.	W
Y	1	1	3	2	0	1	2	2	2	1	3	1	2	2	0	1	2	4	1	1	1	2	3	4	2	1	5	4	0	1	3	1	1	3	1	4	2	3	1	3	2	1	0	3	0	1	3	0	3	4	3	0.627	0.424	–	–	Y
total	73	102	98	106	120	110	98	83	75	97	77	84	86	84	101	74	75	82	78	91	75	85	108	79	69	109	107	79	83	69	85	89	101	94	99	77	90	91	88	83	78	88	92	74	92	103	99	99	103	88	175	0.511	0.070			total

Additional file 5. Continued.

ZEBRAFISH x-x-T-x-I

position -4

	C50	C49	C48	C47	C46	C45	C44	C43	C42	C41	C40	C39	C38	C37	C36	C35	C34	C33	C32	C31	C30	C29	C28	C27	C26	C25	C24	C23	C22	C21	C20	C19	C18	C17	C16	C15	C14	C13	C12	C11	C10	C9	C8	C7	C6	C5	C4	C3	C2	C1	C0	C-index	SD	*P-value*	mark	
A	5	1	3	0	2	3	2	7	2	4	5	8	11	1	2	2	1	3	0	1	5	4	1	9	1	1	1	1	8	1	0	5	7	2	2	1	3	1	5	3	3	4	2	1	1	1	1	3	1	4	6	0.483	0.418	8.60E-04	n.s.	A
C	0	0	1	3	2	0	1	1	2	0	0	1	3	0	1	1	0	0	0	0	1	0	2	0	2	1	1	1	2	0	1	1	1	1	1	0	1	0	0	3	0	2	1	0	1	0	2	2	2	0	0	–	–	–	–	C
D	3	3	2	5	1	1	2	1	1	0	3	2	3	4	4	4	6	2	3	2	2	2	6	9	3	7	0	3	1	5	8	2	1	1	5	13	4	1	2	3	0	1	3	0	5	5	1	4	4	2	5	0.620	0.500	3.08E-01	n.s.	D
E	5	1	3	5	4	2	4	2	1	2	2	1	4	1	1	2	2	0	2	2	2	4	1	3	3	7	3	5	2	1	0	1	3	2	3	0	4	3	3	5	6	2	5	1	2	2	1	2	2	2	3	0.840	0.527	–	–	E
F	2	3	0	2	1	3	3	1	1	3	0	5	2	1	1	2	1	3	2	3	3	2	1	3	1	0	3	3	1	1	2	4	2	1	2	5	1	4	1	1	3	4	4	5	5	1	0	0	3	0	6	0.350	0.241	6.11E-08	✻	F
G	4	1	6	1	0	2	1	4	4	1	2	4	3	7	7	1	2	3	2	9	1	4	6	3	2	2	1	0	10	4	4	0	2	0	3	9	4	1	5	2	3	1	3	3	4	1	3	6	2	0	1	3.060	2.419	–	–	G
H	0	0	0	2	0	2	0	0	2	1	0	4	1	0	1	1	3	5	0	1	2	1	1	1	2	1	1	1	1	1	3	1	0	0	1	0	2	0	0	0	0	0	0	1	0	0	1	0	0	1	5	0.180	0.222	5.55E-08	✻	H
I	2	2	7	1	4	2	3	6	5	0	2	3	2	2	3	4	1	2	1	3	4	2	4	5	5	1	1	1	3	3	6	4	6	1	5	7	2	1	3	6	2	0	2	1	4	1	6	1	2	1	2	1.450	0.949	–	–	I
K	3	22	10	1	6	2	3	3	2	2	1	2	2	3	2	4	0	5	0	6	3	5	4	7	2	7	2	1	10	6	3	4	2	4	1	2	4	3	3	1	5	2	7	4	5	1	0	2	3	0	3	1.213	1.174	–	–	K
L	3	4	5	4	8	4	4	1	4	7	8	6	5	2	8	6	9	4	3	2	1	6	4	6	10	2	4	6	3	4	6	4	3	6	6	2	11	6	5	4	6	4	3	4	7	5	2	4	6	4	4	1.205	0.546	–	–	L
M	0	0	3	0	1	0	1	4	0	4	0	0	2	0	1	2	1	0	1	2	1	1	0	7	3	0	0	1	0	1	0	0	1	2	0	0	3	1	2	0	1	0	0	0	1	2	2	0	2	0	0	–	–	–	–	M
N	5	5	1	2	4	2	2	3	0	1	1	6	5	1	3	3	4	5	2	2	8	2	1	6	1	2	1	2	0	1	5	3	2	3	5	11	8	2	1	4	1	6	2	1	1	2	2	1	4	1	1	2.920	2.284	–	–	N
P	1	0	3	2	2	1	1	2	2	2	3	4	9	5	1	3	1	3	7	3	5	4	5	2	1	0	0	0	4	1	2	2	2	4	5	2	5	3	8	3	0	3	4	5	2	1	3	3	3	2	8	0.348	0.248	5.58E-08	✻	P
Q	4	2	1	7	1	0	0	0	1	2	3	5	0	0	0	0	3	2	3	0	3	1	7	1	0	2	1	0	0	3	1	1	3	7	3	6	7	2	2	2	1	2	3	1	0	1	0	2	2	2	8	0.250	0.253	5.56E-08	✻	Q
R	0	0	2	2	0	3	3	1	0	4	0	1	0	3	5	0	4	0	1	1	4	0	3	0	2	2	1	2	2	3	2	2	5	2	3	0	2	3	0	1	1	4	1	1	1	8	1	2	3	3	6	0.313	0.279	5.60E-08	✻	R
S	1	4	3	0	2	2	2	2	1	3	3	4	8	8	1	4	3	3	4	0	1	5	1	3	4	4	1	7	5	2	4	2	2	4	0	9	8	4	4	5	3	8	1	3	2	2	3	3	6	3	7	0.477	0.317	2.44E-05	✻	S
T	7	4	0	1	3	3	2	0	4	3	0	3	3	6	1	6	3	0	5	2	1	3	7	5	8	2	4	3	5	2	6	5	2	9	4	4	2	1	4	1	2	9	4	3	5	4	5	2	4	3	6	0.583	0.369	3.72E-01	n.s.	T
V	3	0	6	3	4	2	3	3	1	3	5	3	0	3	4	2	3	0	2	4	3	2	6	1	4	1	4	13	2	5	2	1	5	3	4	8	15	5	7	1	3	1	1	3	2	2	9	7	1	1	2	1.760	1.489	–	–	V
W	0	0	0	1	0	0	1	0	1	0	1	2	0	0	0	0	0	1	1	1	0	1	0	0	1	3	2	0	0	1	1	1	0	1	0	0	1	0	0	0	0	0	0	0	1	0	1	2	1	0	0	–	–	–	–	W
Y	0	2	0	1	2	0	0	4	0	2	0	0	0	0	1	1	1	2	1	3	3	2	0	0	2	0	0	2	0	0	1	1	1	2	0	4	0	0	2	2	2	1	0	1	3	4	1	1	0	3	1	1.160	1.218	–	–	Y
total	48	54	56	43	47	34	38	45	34	44	39	64	63	47	47	48	48	43	40	47	53	51	60	71	57	45	31	52	59	45	57	44	50	55	53	83	87	41	57	47	42	54	46	38	52	43	44	47	51	32	74	0.669	0.149			total

position -3

	C50	C49	C48	C47	C46	C45	C44	C43	C42	C41	C40	C39	C38	C37	C36	C35	C34	C33	C32	C31	C30	C29	C28	C27	C26	C25	C24	C23	C22	C21	C20	C19	C18	C17	C16	C15	C14	C13	C12	C11	C10	C9	C8	C7	C6	C5	C4	C3	C2	C1	C0	C-index	SD	*P-value*	mark	
A	5	1	1	0	4	2	0	2	2	1	1	1	0	2	3	2	2	4	3	4	3	1	0	3	2	1	1	10	2	4	4	1	2	2	3	4	5	3	0	1	2	7	5	3	2	3	0	8	7	3	5	0.528	0.427	7.75E-03	n.s.	A
C	3	1	2	1	1	1	0	5	3	3	0	0	2	1	0	1	2	2	1	1	0	0	0	1	1	1	0	3	0	1	1	3	3	1	0	0	1	0	1	0	1	4	1	0	0	0	6	0	2	1	2	0.620	0.689	1.03E-02	n.s.	C
D	0	4	4	2	0	2	2	4	1	3	4	6	3	1	1	1	0	1	1	1	2	2	1	4	2	2	0	3	5	0	3	2	9	7	3	8	3	6	4	2	1	0	1	2	5	1	0	1	4	0	3	0.827	0.717	–	–	D
E	3	2	2	6	3	2	1	1	0	1	1	2	2	3	0	7	0	2	3	2	3	2	1	5	3	5	4	3	1	1	0	2	8	3	1	1	1	3	3	3	1	3	3	0	5	5	2	5	4	2	9	0.280	0.201	5.55E-08	✻	E
F	2	1	1	3	1	0	1	4	2	1	2	4	0	4	2	1	5	4	5	2	1	0	4	6	4	1	1	6	0	2	6	0	1	3	1	1	1	3	1	3	5	1	2	1	2	0	2	2	0	4	0	–	–	–	–	F
G	1	1	3	0	0	2	1	3	4	4	4	10	12	0	4	3	3	1	3	4	6	7	3	0	7	1	1	6	4	0	1	1	2	2	3	7	3	3	1	2	1	3	1	4	1	5	1	2	3	3	2	1.470	1.255	–	–	G
H	0	2	1	1	0	1	1	1	3	1	1	4	4	2	3	1	4	2	0	4	2	2	5	2	1	1	0	0	2	2	0	0	1	3	1	0	7	0	0	2	1	1	3	4	0	4	2	0	2	0	1	1.680	1.584	–	–	H
I	2	3	4	1	2	3	2	0	1	2	3	0	3	2	1	3	4	3	3	3	2	4	7	6	3	2	0	3	1	3	8	1	2	6	1	6	3	0	3	2	1	6	6	1	3	3	1	2	4	0	3	0.900	0.633	–	–	I
K	4	18	7	2	2	1	3	1	1	0	1	5	4	8	6	2	3	0	4	3	6	3	2	2	0	4	1	1	8	1	1	5	2	1	3	6	9	0	2	3	8	2	2	3	2	3	0	1	3	6	1	3.300	3.164	–	–	K
L	6	2	2	1	9	3	15	6	3	1	5	9	14	4	5	5	3	6	3	6	7	4	14	9	6	1	4	2	1	6	7	6	4	4	7	1	8	9	12	11	2	5	4	2	7	5	2	2	4	1	16	0.331	0.222	5.56E-08	✻	L
M	2	1	0	2	3	1	4	1	1	5	1	2	0	2	2	2	3	0	2	1	1	0	1	3	0	1	1	0	1	3	0	2	0	2	1	0	1	0	2	0	0	2	2	0	3	1	0	1	1	0	4	0.320	0.295	5.98E-08	✻	M
N	0	1	7	3	2	1	3	2	1	1	1	2	1	2	6	3	2	2	1	2	2	4	2	2	3	0	1	0	4	3	3	3	3	2	4	4	2	1	3	1	2	1	1	1	1	3	8	3	1	3	2	1.140	0.808	–	–	N
P	1	0	1	2	0	1	0	0	1	1	5	1	4	9	3	2	0	2	0	1	6	2	2	1	2	1	6	3	1	4	1	1	1	4	2	4	3	0	5	2	1	3	0	0	1	2	3	1	4	2	0	–	–	–	–	P
Q	4	4	2	0	1	0	1	2	0	4	1	3	1	2	0	4	0	2	2	1	2	3	4	2	2	4	3	3	7	1	9	3	0	1	6	18	6	1	0	5	3	2	1	1	2	3	3	4	0	1	7	0.383	0.422	6.34E-08	✻	Q
R	7	4	1	5	5	1	0	2	3	6	0	2	3	1	3	0	1	1	2	3	0	3	3	5	1	1	0	2	3	2	1	1	0	3	4	3	1	3	1	2	4	2	2	4	1	1	2	4	4	0	6	0.377	0.281	4.81E-07	✻	R
S	3	3	7	4	3	3	2	4	1	2	4	3	5	4	3	6	7	1	2	4	4	3	9	4	2	8	3	1	7	3	3	3	4	3	4	2	5	3	9	2	5	5	7	4	7	1	3	3	2	2	6	0.640	0.337	4.04E-01	n.s.	S
T	1	2	4	2	6	2	0	3	4	3	1	4	1	0	2	2	6	3	1	1	3	3	1	4	9	4	1	2	2	2	2	3	3	3	3	5	5	3	3	1	2	1	1	4	5	2	7	5	3	1	0	–	–	–	–	T
V	3	3	4	8	3	5	2	1	0	1	2	0	4	0	0	2	2	2	3	1	3	6	1	3	3	3	4	2	2	4	2	3	3	2	3	3	3	1	5	2	0	0	3	3	4	1	2	0	1	2	4	0.600	0.410	9.03E-01	n.s.	V
W	0	0	1	0	1	0	0	1	1	0	0	1	0	0	2	0	0	1	0	0	0	1	0	0	1	2	0	0	8	1	0	3	0	2	0	1	1	1	0	0	0	0	0	1	1	0	0	1	1	0	0	–	–	–	–	W
Y	1	1	2	0	1	3	0	2	2	4	2	5	0	0	1	1	1	4	1	3	0	1	0	9	5	2	0	2	0	2	5	1	2	1	3	9	19	1	2	3	2	6	1	0	0	0	0	2	1	1	3	0.760	1.065	–	–	Y
total	48	54	56	43	47	34	38	45	34	44	39	64	63	47	47	48	48	43	40	47	53	51	60	71	57	45	31	52	59	45	57	44	50	55	53	83	87	41	57	47	42	54	46	38	52	43	44	47	51	32	74	0.669	0.149			total

position -1

	C50	C49	C48	C47	C46	C45	C44	C43	C42	C41	C40	C39	C38	C37	C36	C35	C34	C33	C32	C31	C30	C29	C28	C27	C26	C25	C24	C23	C22	C21	C20	C19	C18	C17	C16	C15	C14	C13	C12	C11	C10	C9	C8	C7	C6	C5	C4	C3	C2	C1	C0	C-index	SD	*P-value*	mark	
A	3	1	3	2	2	0	2	3	4	4	4	7	4	1	4	4	1	7	3	2	4	5	6	6	6	1	1	2	1	3	4	0	6	3	1	2	4	4	3	1	1	1	2	2	2	7	2	3	3	1	2	1.480	0.931	–	–	A
C	2	1	0	3	2	2	1	0	1	1	1	1	0	0	0	1	1	0	2	2	1	0	1	1	2	1	0	3	2	0	0	1	2	2	0	0	0	0	2	0	2	0	2	0	2	1	1	2	2	1	1	1.040	0.903	–	–	C
D	3	2	3	3	2	2	0	2	4	2	1	2	5	1	2	1	4	0	0	1	2	0	5	3	2	2	3	5	2	3	3	0	2	2	7	4	5	4	9	1	1	10	3	2	2	5	1	1	4	2	1	2.700	2.092	–	–	D
E	0	2	5	2	7	3	3	4	3	2	2	5	5	1	3	2	1	2	3	3	4	3	2	3	5	4	3	10	10	1	3	2	2	5	4	3	5	4	1	2	2	1	1	0	3	1	3	9	4	4	8	0.405	0.274	6.19E-08	✻	E
F	4	1	4	2	3	1	3	4	0	3	3	5	1	1	4	2	2	3	2	4	6	3	1	9	4	2	2	3	0	3	5	2	4	5	8	2	5	1	0	3	3	3	3	0	3	1	4	0	2	4	1	2.860	1.895	–	–	F
G	3	3	1	4	4	2	1	5	4	3	4	6	3	2	6	6	0	4	1	4	6	4	5	0	4	2	2	2	3	1	1	2	4	2	3	2	2	3	3	3	1	1	1	1	0	0	1	0	3	1	3	0.860	0.568	–	–	G
H	2	5	1	2	1	1	2	1	0	4	0	0	4	0	2	4	6	2	0	1	1	1	6	3	2	1	2	0	0	3	1	0	1	2	3	1	3	1	3	4	2	3	2	0	3	1	0	1	0	0	7	0.251	0.226	5.55E-08	✻	H
I	0	1	1	1	2	0	2	0	3	1	1	1	3	2	1	0	0	2	2	1	3	5	2	3	1	2	0	1	3	2	1	1	1	0	1	2	7	2	1	2	2	3	2	2	3	2	5	3	5	0	1	1.820	1.466	–	–	I
K	0	1	4	2	1	1	0	7	3	2	2	1	3	4	2	3	3	1	1	4	0	4	1	3	4	2	0	0	2	4	2	2	1	8	0	5	13	2	4	7	3	5	1	4	5	1	2	3	5	0	8	0.345	0.305	5.65E-08	✻	K
L	4	3	9	9	5	4	5	3	2	4	1	7	14	4	3	4	4	3	5	7	9	6	11	10	3	6	3	2	7	6	2	2	8	4	6	11	7	3	11	6	1	9	6	2	9	2	5	9	5	0	3	1.807	1.041	–	–	L
M	3	0	3	0	0	0	0	0	1	0	2	0	1	4	0	4	2	1	0	1	1	1	0	0	2	0	0	0	0	0	2	4	0	1	1	2	1	1	0	1	0	2	1	0	1	0	1	0	0	0	1	0.880	1.154	–	–	M
N	1	1	2	4	1	3	2	1	1	1	2	7	3	1	3	1	2	0	3	1	0	1	1	2	4	3	0	2	3	0	0	3	2	2	0	7	4	0	1	1	0	2	2	6	1	6	2	2	2	1	2	1.000	0.869	–	–	N
P	2	0	3	0	2	0	2	0	1	0	3	4	3	2	3	4	3	1	2	3	0	0	2	7	3	5	4	2	6	4	7	1	2	3	4	6	4	2	5	2	1	4	5	2	1	3	6	4	2	0	4	0.675	0.477	–	–	P
Q	1	3	1	0	2	1	6	0	0	2	0	1	1	2	3	5	2	3	2	2	6	4	2	2	1	4	2	2	2	0	2	4	1	3	0	2	1	2	3	3	2	3	3	3	2	0	0	0	0	4	9	0.222	0.169	5.55E-08	✻	Q
R	2	22	9	1	2	0	0	2	1	2	0	3	0	0	3	0	1	5	4	4	0	1	4	2	5	1	1	1	1	6	2	3	4	1	2	0	15	3	1	3	6	2	1	4	3	0	3	5	1	4	2	1.460	1.908	–	–	R
S	6	2	5	4	1	2	0	4	2	4	2	5	6	4	2	0	3	3	5	3	5	1	6	1	6	4	4	4	12	4	5	5	4	6	2	11	4	3	4	4	7	0	3	5	6	4	1	2	3	4	8	0.483	0.293	6.07E-05	✻	S
T	4	3	1	2	3	3	1	2	1	1	4	0	4	9	3	0	4	2	2	0	0	5	0	5	1	0	3	1	0	1	3	3	2	3	3	3	1	1	0	1	2	2	0	2	2	4	0	2	4	4	6	0.357	0.295	9.22E-08	✻	T
V	4	2	0	2	5	8	2	4	2	5	3	3	3	8	0	4	5	2	2	4	2	5	2	3	0	4	1	11	2	2	5	5	4	2	3	2	3	2	2	3	4	2	5	2	1	3	5	0	4	1	4	0.790	0.526	–	–	V
W	0	0	0	0	0	0	2	2	0	0	1	0	0	1	0	2	2	0	0	0	0	0	0	0	1	0	0	0	1	2	7	3	0	0	5	17	2	0	0	0	1	0	0	1	3	0	0	0	1	0	0	–	–	–	–	W
Y	4	1	1	0	2	1	4	1	1	3	3	6	0	0	3	1	2	2	1	0	3	2	3	8	1	1	0	1	2	0	2	1	0	1	0	1	1	3	4	0	1	1	3	0	0	2	2	1	1	1	3	0.547	0.538	3.75E-02	n.s.	Y
total	48	54	56	43	47	34	38	45	34	44	39	64	63	47	47	48	48	43	40	47	53	51	60	71	57	45	31	52	59	45	57	44	50	55	53	83	87	41	57	47	42	54	46	38	52	43	44	47	51	32	74	0.669	0.149			total

Additional file 5. Continued.

ZEBRAFISH x-x-T-x-L

position -4

	C50	C49	C48	C47	C46	C45	C44	C43	C42	C41	C40	C39	C38	C37	C36	C35	C34	C33	C32	C31	C30	C29	C28	C27	C26	C25	C24	C23	C22	C21	C20	C19	C18	C17	C16	C15	C14	C13	C12	C11	C10	C9	C8	C7	C6	C5	C4	C3	C2	C1	C0	C-index	SD	*P-value*	mark	
A	4	9	24	7	7	9	6	2	7	7	6	5	6	9	5	4	11	2	3	3	12	9	4	6	2	6	6	5	5	3	6	8	2	5	4	7	2	5	5	3	6	5	3	4	7	4	7	4	3	5	13	0.445	0.269	–	–	A
C	0	1	0	3	1	1	5	3	2	4	1	0	4	1	1	1	5	4	2	1	4	4	2	1	1	1	1	2	2	3	1	1	2	1	3	4	1	4	3	4	3	3	0	1	1	0	1	2	1	1	4	0.490	0.353	–	–	C
D	3	4	5	7	3	4	2	1	6	3	3	4	9	4	1	3	2	3	3	7	4	2	3	5	1	2	6	8	10	2	6	2	4	1	5	5	5	3	6	7	3	7	8	5	4	2	2	3	7	6	16	0.264	0.138	6.19E-08	*	D
E	1	6	5	5	2	6	3	3	3	4	6	1	11	6	0	5	4	8	6	5	4	4	9	7	30	6	4	8	6	6	4	5	9	4	3	7	5	4	6	6	6	2	4	6	3	3	9	2	7	3	18	0.302	0.233	5.75E-08	*	E
F	3	4	7	5	5	5	2	1	7	14	13	2	4	3	5	3	3	4	3	3	4	6	2	0	3	5	5	3	4	3	5	2	5	4	5	4	1	4	5	2	7	4	2	4	7	7	1	1	3	1	12	0.342	0.218	7.95E-05	*	F
G	7	5	7	5	7	2	5	9	8	5	4	4	3	2	6	5	14	9	0	5	7	7	8	14	6	6	5	4	6	4	3	8	3	5	1	2	7	8	7	4	1	11	4	2	7	4	2	8	1	4	8	0.678	0.375	–	–	G
H	3	1	1	2	2	4	2	4	4	0	3	1	0	3	3	1	4	1	6	5	0	4	4	1	2	2	2	3	13	3	5	0	3	1	1	0	6	0	3	1	1	6	2	3	0	1	4	1	3	0	5	0.500	0.458	–	–	H
I	1	9	9	9	6	3	6	2	3	5	7	13	1	10	3	1	10	4	3	8	10	4	9	7	8	6	13	9	3	5	5	4	4	4	2	6	0	7	8	11	8	5	3	3	9	5	9	5	3	7	6	0.983	0.529	–	–	I
K	10	1	3	4	6	3	7	5	5	7	6	5	7	10	6	8	3	11	8	5	3	2	5	3	2	1	6	6	3	4	9	4	8	4	8	3	1	6	6	13	12	7	10	1	7	2	3	4	15	10	27	0.213	0.121	5.55E-08	*	K
L	8	8	16	10	14	9	16	12	7	5	16	12	6	9	12	3	4	7	7	11	11	15	10	9	3	13	11	10	16	10	6	15	13	12	15	10	4	11	12	17	10	8	4	14	5	10	9	10	12	8	20	0.505	0.186	–	–	L
M	2	1	1	1	3	2	0	4	0	2	1	3	2	4	1	4	4	3	4	0	4	0	2	3	5	1	2	0	2	3	0	0	2	2	6	3	7	2	2	7	0	1	4	5	1	1	0	0	2	4	2	1.130	0.925	–	–	M
N	6	6	2	1	1	7	3	4	6	5	6	6	8	3	4	3	4	5	4	5	5	1	1	2	5	1	4	5	6	4	2	2	3	4	2	5	7	3	1	5	6	8	1	3	4	2	2	1	2	7	5	0.772	0.408	–	–	N
P	3	11	4	3	6	3	6	6	6	4	4	1	6	4	4	8	4	5	4	2	3	1	3	7	1	4	9	8	2	4	2	4	2	4	3	3	2	2	2	3	2	3	2	4	4	2	6	2	4	5	8	0.493	0.265	–	–	P
Q	2	0	1	2	8	4	1	7	5	1	9	2	6	2	8	3	4	3	3	4	4	5	1	2	0	6	5	5	3	4	3	3	4	5	4	7	8	1	4	3	5	3	7	2	2	5	4	3	3	5	10	0.382	0.216	1.64E-01	n.s.	Q
R	7	2	1	3	5	4	3	8	3	1	3	3	7	9	5	6	4	5	5	8	6	5	5	4	2	3	2	3	7	4	11	15	5	12	5	1	4	4	4	7	4	6	5	3	4	5	2	1	5	6	14	0.346	0.199	1.05E-04	n.s.	R
S	3	6	5	8	4	15	9	6	8	9	8	5	4	4	6	10	13	10	3	11	5	10	5	10	3	9	7	3	10	6	14	4	5	12	5	8	7	9	9	6	15	11	9	9	5	5	4	3	9	4	12	0.613	0.270	–	–	S
T	9	2	8	3	6	4	3	6	7	9	7	4	1	6	10	7	9	6	6	3	4	9	7	5	10	4	2	4	6	8	4	3	7	9	6	4	6	7	6	4	12	4	8	3	3	2	4	5	4	12	17	0.339	0.154	8.91E-04	n.s.	T
V	7	11	11	6	8	3	1	3	1	11	10	9	5	7	11	4	5	5	4	5	11	8	5	4	3	7	12	8	7	6	4	9	2	2	4	6	6	5	2	7	5	6	7	8	3	5	4	11	3	3	8	0.750	0.369	–	–	V
W	1	0	1	0	0	1	1	4	1	0	1	0	0	1	0	1	1	0	0	1	0	0	0	0	1	2	1	0	0	2	1	2	2	2	1	0	1	2	0	0	3	0	2	1	0	1	0	1	1	0	3	0.267	0.301	5.59E-07	*	W
Y	3	2	3	4	5	2	4	3	0	4	1	0	1	2	13	4	10	4	6	3	4	2	4	4	1	2	3	0	3	1	1	0	0	1	1	0	3	1	2	4	1	3	1	1	2	6	7	4	2	5	4	0.715	0.625	–	–	Y
total	83	89	114	88	99	91	85	93	89	100	115	80	91	99	104	84	118	99	80	95	105	98	89	94	89	87	106	94	114	85	92	91	85	94	84	85	83	88	93	114	110	103	86	82	78	72	80	71	90	96	212	0.437	0.052			total

position -3

	C50	C49	C48	C47	C46	C45	C44	C43	C42	C41	C40	C39	C38	C37	C36	C35	C34	C33	C32	C31	C30	C29	C28	C27	C26	C25	C24	C23	C22	C21	C20	C19	C18	C17	C16	C15	C14	C13	C12	C11	C10	C9	C8	C7	C6	C5	C4	C3	C2	C1	C0	C-index	SD	*P-value*	mark	
A	5	2	9	7	3	2	3	5	4	4	10	5	2	2	4	7	4	4	4	4	3	8	2	3	1	5	9	6	9	3	5	6	9	3	3	5	5	3	10	6	3	6	5	2	4	2	3	3	1	2	7	0.643	0.341	–	–	A
C	1	3	1	2	1	1	2	0	3	3	2	1	2	3	2	1	1	1	4	1	1	0	2	1	0	3	0	0	2	0	1	0	0	8	0	2	1	1	2	2	2	2	1	2	0	1	1	1	3	0	16	0.093	0.087	5.55E-08	*	C
D	3	4	3	3	4	5	2	2	5	6	7	4	5	7	3	6	8	7	4	3	7	5	5	5	31	2	4	9	3	5	4	3	5	3	3	3	2	2	3	8	10	8	4	6	6	6	3	3	13	6	17	0.315	0.256	1.14E-07	*	D
E	0	6	1	2	1	5	7	3	3	2	4	2	8	6	4	4	8	8	10	2	6	4	4	11	1	9	3	6	7	1	2	11	6	4	5	4	8	7	5	3	1	2	4	1	5	1	4	1	4	3	29	0.151	0.097	5.55E-08	*	E
F	4	6	2	6	10	8	5	6	5	1	10	4	8	4	11	2	5	6	1	5	2	10	4	9	1	8	7	2	7	5	2	6	3	8	4	4	1	3	1	5	10	6	1	2	3	3	0	3	4	5	1	4.760	2.861	–	–	F
G	7	2	7	8	8	6	9	3	7	4	8	7	3	2	1	8	9	3	5	2	3	1	1	6	6	5	8	4	8	4	9	2	8	4	1	6	7	2	3	4	5	4	4	5	2	0	6	6	0	2	5	0.940	0.524	–	–	G
H	2	3	6	2	4	7	4	5	4	2	1	5	2	4	2	2	2	3	1	9	1	2	2	2	1	5	4	2	3	3	2	3	3	2	6	5	6	1	4	4	4	3	4	3	3	2	2	3	2	2	8	0.398	0.211	1.07E-01	n.s.	H
I	8	2	8	7	5	4	7	4	3	10	7	1	6	5	10	2	8	6	6	7	6	5	5	4	7	4	4	9	16	6	9	8	4	3	10	2	3	3	3	5	8	10	4	5	4	2	10	0	10	15	15	0.400	0.219	3.91E-01	n.s.	I
K	6	4	10	6	1	8	6	2	13	2	3	4	9	5	3	5	3	6	3	4	8	2	8	3	3	4	11	6	6	5	6	4	2	4	5	3	5	1	9	11	6	7	5	11	1	7	4	5	4	12	7	0.774	0.424	–	–	K
L	10	12	16	11	13	8	5	14	7	10	9	13	4	9	5	15	12	6	16	15	12	13	12	13	3	6	20	12	13	8	8	7	6	19	10	4	7	14	13	15	17	12	14	9	13	9	10	8	6	9	18	0.591	0.221	–	–	L
M	0	4	0	3	3	2	3	1	2	4	4	2	1	7	10	3	3	3	1	2	2	3	3	3	2	4	1	3	1	4	6	2	2	2	3	5	2	1	2	2	2	5	0	2	0	3	2	1	1	1	8	0.320	0.229	4.80E-06	*	M
N	4	4	10	5	5	3	3	6	5	5	6	1	5	7	11	2	8	0	2	4	8	7	4	9	1	2	2	4	2	1	2	7	2	6	4	1	1	2	1	1	13	1	3	3	6	3	3	2	5	0	7	0.577	0.417	–	–	N
P	3	6	2	0	1	2	2	2	6	5	4	3	1	1	6	1	6	4	2	6	16	4	2	3	2	2	3	5	4	3	2	0	6	5	3	4	7	9	3	7	2	2	3	4	3	5	3	4	4	3	7	0.531	0.373	–	–	P
Q	4	7	15	5	3	5	4	7	1	8	6	1	3	4	7	5	4	11	3	5	6	10	1	4	2	4	3	3	6	7	5	7	3	4	3	4	5	9	5	5	6	8	10	4	3	3	5	4	4	5	14	0.366	0.190	1.62E-03	n.s.	Q
R	1	5	5	4	4	4	6	8	3	4	8	1	11	5	2	4	4	7	6	6	2	4	6	3	7	8	3	3	5	7	2	5	4	3	7	4	1	9	5	5	7	10	2	2	5	6	6	3	8	8	10	0.496	0.236	–	–	R
S	10	7	4	8	13	3	3	4	6	8	4	2	7	6	7	3	19	7	3	5	4	3	10	4	9	8	7	5	7	10	13	3	11	3	5	12	10	7	6	6	8	7	8	6	8	8	5	7	8	8	13	0.531	0.248	–	–	S
T	5	7	6	3	7	5	9	6	1	9	7	3	4	10	5	2	2	3	3	5	5	6	13	2	5	1	3	10	9	4	2	3	4	5	3	10	4	3	5	5	2	4	6	4	4	3	4	1	8	6	13	0.378	0.205	1.54E-02	n.s.	T
V	4	1	5	1	10	5	2	9	8	10	8	18	6	6	9	6	10	12	5	9	10	5	3	7	5	2	8	3	3	7	6	3	6	6	5	5	6	6	8	11	2	3	2	3	5	5	4	8	4	5	11	0.545	0.293	–	–	V
W	3	0	2	1	1	1	0	3	1	0	4	0	4	4	2	5	0	0	1	0	0	2	1	0	1	1	1	2	0	0	3	3	0	0	0	1	0	4	1	4	2	0	3	3	2	2	2	1	1	1	1	1.460	1.417	–	–	W
Y	3	4	2	4	2	7	3	3	2	3	3	3	0	2	0	1	2	2	0	1	3	4	1	2	1	4	5	0	3	2	3	8	1	2	4	1	2	1	4	5	0	3	3	5	1	1	3	7	0	3	5	0.516	0.368	–	–	Y
total	83	89	114	88	99	91	85	93	89	100	115	80	91	99	104	84	118	99	80	95	105	98	89	94	89	87	106	94	114	85	92	91	85	94	84	85	83	88	93	114	110	103	86	82	78	72	80	71	90	96	212	0.437	0.052			total

position -1

	C50	C49	C48	C47	C46	C45	C44	C43	C42	C41	C40	C39	C38	C37	C36	C35	C34	C33	C32	C31	C30	C29	C28	C27	C26	C25	C24	C23	C22	C21	C20	C19	C18	C17	C16	C15	C14	C13	C12	C11	C10	C9	C8	C7	C6	C5	C4	C3	C2	C1	C0	C-index	SD	*P-value*	mark	
A	4	9	6	3	15	8	9	5	3	6	9	6	2	4	6	2	8	6	5	9	8	6	5	6	3	7	9	6	4	7	12	12	9	16	5	7	7	8	7	8	10	7	2	3	8	4	7	7	8	6	17	0.399	0.175	3.33E-01	n.s.	A
C	2	2	1	3	3	3	3	1	0	3	2	3	1	1	0	0	3	1	1	5	1	3	0	2	0	2	1	2	0	1	0	0	2	0	0	1	2	1	4	6	0	1	0	1	1	1	1	0	3	3	4	0.385	0.351	5.83E-01	n.s.	C
D	0	3	16	8	2	1	5	8	4	7	4	6	3	8	10	5	10	11	4	3	5	5	4	8	0	0	3	4	6	6	4	6	4	5	2	5	1	6	6	4	1	7	3	3	6	2	4	0	8	2	20	0.238	0.157	5.56E-08	*	D
E	2	5	8	5	7	5	5	3	6	4	3	1	10	11	9	2	7	5	6	8	11	8	4	6	8	10	8	6	9	7	7	5	8	4	4	4	4	6	2	10	8	5	4	4	4	2	5	2	7	7	20	0.291	0.127	1.00E-07	*	E
F	4	4	5	3	3	10	2	3	3	2	3	2	6	3	2	6	7	3	4	3	6	10	6	7	2	3	4	4	7	3	4	1	9	7	9	1	4	6	1	10	1	1	4	3	6	3	3	3	2	5	7	0.609	0.353	–	–	F
G	3	12	5	6	3	4	7	14	6	2	9	14	1	4	4	0	6	10	6	10	4	5	7	9	4	7	7	3	10	4	2	5	6	6	2	1	10	3	5	7	10	12	7	3	2	2	3	2	2	2	8	0.695	0.434	–	–	G
H	8	3	2	4	3	4	2	2	3	1	9	1	2	2	3	1	4	5	4	2	2	6	6	3	1	4	1	5	4	3	5	4	5	3	6	5	2	2	2	6	5	3	2	6	1	3	3	3	2	5	10	0.346	0.184	5.67E-03	n.s.	H
I	2	3	5	4	3	5	5	5	4	4	2	5	14	7	5	3	1	5	3	2	5	5	6	4	11	3	6	4	7	0	5	3	1	4	3	4	2	5	8	3	9	6	8	4	3	2	1	4	5	4	9	0.493	0.280	–	–	I
K	5	2	2	3	3	3	2	4	4	3	6	2	2	9	6	4	4	2	3	3	7	3	5	0	2	6	1	7	3	5	3	10	2	3	6	5	4	6	3	7	4	5	4	2	3	3	4	3	6	9	12	0.338	0.175	7.38E-04	n.s.	K
L	4	5	10	15	5	8	12	6	9	12	9	5	6	16	14	5	11	18	13	11	15	7	3	11	9	9	15	6	11	11	8	4	5	8	12	11	7	10	11	19	16	14	19	13	10	11	14	9	7	7	15	0.675	0.270	–	–	L
M	3	4	2	1	2	1	1	2	1	1	2	1	5	4	1	1	7	2	4	0	2	2	2	1	0	1	2	5	2	1	1	1	0	2	2	3	2	3	1	2	1	3	0	0	0	0	2	3	0	4	3	0.620	0.499	–	–	M
N	4	0	5	1	9	3	0	5	4	5	4	7	4	2	8	1	4	1	1	4	0	2	3	3	26	3	4	1	11	1	0	1	0	5	1	1	7	3	3	1	3	4	1	1	2	3	1	4	13	6	3	1.240	1.424	–	–	N
P	7	3	9	5	6	1	6	7	7	5	7	4	4	3	6	7	4	2	2	9	2	2	3	8	2	2	6	2	6	4	2	5	8	7	8	7	5	3	6	3	8	3	4	9	6	7	3	4	7	3	6	0.830	0.379	–	–	P
Q	7	5	6	4	5	5	3	4	7	8	5	5	3	5	1	4	7	4	5	4	8	6	4	3	2	3	2	3	5	1	6	3	2	4	3	4	3	4	4	2	2	8	3	3	4	6	4	4	4	2	4	1.045	0.437	–	–	Q
R	5	2	9	10	2	9	4	2	7	7	4	6	11	1	8	8	3	1	4	6	6	8	2	5	2	2	12	5	6	4	4	4	1	4	3	5	1	1	10	4	6	2	3	6	3	1	4	4	4	6	16	0.296	0.177	5.83E-06	*	R
S	8	7	9	2	6	10	12	6	6	8	20	4	6	11	14	13	11	7	6	5	10	4	10	6	6	8	15	7	8	9	8	11	13	5	6	8	4	6	11	6	11	5	2	4	7	6	5	6	4	15	26	0.305	0.139	9.07E-07	*	S
T	3	7	6	4	8	1	2	2	3	4	7	3	2	3	1	15	1	11	1	4	0	2	8	3	4	5	3	6	6	5	9	1	2	2	4	6	6	8	2	6	3	7	6	4	2	4	5	6	3	6	11	0.404	0.260	6.53E-01	n.s.	T
V	7	9	8	4	11	5	4	10	11	13	4	4	6	4	3	3	14	2	7	4	9	9	11	7	1	10	3	11	6	7	8	7	5	5	5	3	7	5	4	9	7	9	9	7	6	6	6	3	3	3	14	0.463	0.214	–	–	V
W	1	0	0	1	1	1	1	2	1	1	0	1	1	0	1	1	2	1	1	2	1	4	0	1	0	1	1	0	2	1	0	3	0	0	1	1	4	1	0	0	3	0	0	2	1	2	2	3	0	1	2	0.540	0.513	–	–	W
Y	4	4	0	2	2	4	0	2	0	4	6	0	2	1	2	3	4	2	0	1	3	1	0	1	6	1	3	7	1	5	4	5	3	4	2	3	1	1	3	1	2	1	5	4	3	4	3	1	2	0	5	0.492	0.357	–	–	Y
total	83	89	114	88	99	91	85	93	89	100	115	80	91	99	104	84	118	99	80	95	105	98	89	94	89	87	106	94	114	85	92	91	85	94	84	85	83	88	93	114	110	103	86	82	78	72	80	71	90	96	212	0.437	0.052			total

Additional file 5. Continued.

ZEBRAFISH x-x-T-x-V

position -4

	C50	C49	C48	C47	C46	C45	C44	C43	C42	C41	C40	C39	C38	C37	C36	C35	C34	C33	C32	C31	C30	C29	C28	C27	C26	C25	C24	C23	C22	C21	C20	C19	C18	C17	C16	C15	C14	C13	C12	C11	C10	C9	C8	C7	C6	C5	C4	C3	C2	C1	C0	C-index	SD	*P-value*	mark	
A	0	3	5	2	6	6	8	3	4	11	6	4	3	3	2	2	5	1	4	1	5	0	2	2	1	2	1	1	2	3	5	2	7	1	4	3	0	4	6	3	3	4	5	3	2	4	4	2	4	2	3	1.107	0.717	–	–	A
C	2	1	4	1	4	0	2	1	2	3	2	2	1	0	1	1	2	3	0	0	0	2	1	1	0	1	4	0	2	0	0	2	0	0	0	0	2	0	0	3	1	2	3	1	0	0	1	0	0	1	4	0.295	0.302	–	–	C
D	2	3	7	7	2	1	2	3	5	3	5	1	3	4	3	0	3	2	2	3	3	2	4	1	2	3	1	4	6	3	2	3	5	2	2	3	1	11	5	0	8	2	2	2	1	2	1	6	7	8	5	0.652	0.457	–	–	D
E	5	4	2	5	1	1	4	3	4	4	4	2	6	3	4	1	5	2	4	4	1	5	2	0	4	2	8	3	2	1	3	5	3	3	2	1	5	7	9	5	4	4	7	7	2	1	7	3	2	3	13	0.275	0.156	9.96E-01	n.s.	E
F	1	3	5	0	0	2	1	0	1	5	1	1	0	6	5	6	3	3	3	0	4	4	3	5	6	1	2	5	2	2	2	3	2	3	3	4	4	0	3	0	6	2	0	3	1	0	1	2	3	1	10	0.246	0.186	6.62E-01	n.s.	F
G	3	4	4	7	4	2	3	3	6	2	7	0	7	16	1	6	2	10	3	2	8	4	7	2	1	4	2	5	7	8	2	7	5	4	10	3	4	1	3	3	5	3	6	2	2	10	2	2	9	2	3	1.500	1.026	–	–	G
H	0	8	0	2	3	0	0	0	2	0	8	3	0	1	0	7	2	1	1	2	2	2	2	1	1	1	1	2	1	0	4	0	1	2	0	2	1	0	2	3	0	0	1	1	2	0	1	0	3	0	7	0.217	0.270	3.19E-03	n.s.	H
I	4	5	3	3	2	2	5	4	3	3	3	4	8	3	6	4	7	2	2	3	5	4	4	31	3	4	4	13	1	2	2	2	1	6	5	2	5	5	3	2	0	3	4	4	3	1	10	5	1	3	21	0.204	0.213	6.73E-08	*	I
K	4	2	8	5	6	1	5	7	2	1	2	2	2	1	6	3	4	2	2	8	7	2	5	2	4	3	6	3	2	3	2	1	0	6	3	2	3	1	0	8	3	7	1	2	8	5	3	6	8	0	14	0.256	0.172	2.80E-01	n.s.	K
L	4	4	8	3	2	6	1	7	7	8	5	2	3	1	4	4	4	5	2	6	4	4	3	6	3	8	3	11	3	2	8	3	5	3	7	12	10	8	7	5	8	6	3	4	7	5	5	1	5	6	20	0.251	0.127	2.00E-01	n.s.	L
M	1	2	0	3	1	2	2	1	0	2	0	0	1	1	1	1	1	2	0	1	2	3	0	4	1	1	1	1	0	1	4	0	2	2	0	0	1	1	6	2	0	5	2	0	2	1	1	1	1	1	12	0.113	0.109	5.55E-08	*	M
N	3	4	4	6	1	0	2	7	2	4	0	0	3	13	1	3	3	6	1	5	6	3	5	3	0	3	4	4	3	2	1	1	1	3	1	5	8	4	12	3	2	0	1	9	3	3	1	2	2	1	12	0.273	0.235	2.21E-01	n.s.	N
P	2	4	5	2	2	5	5	5	3	1	2	0	3	3	1	7	3	1	0	4	5	2	1	2	2	1	3	3	2	2	0	2	1	6	1	2	2	5	3	3	2	0	7	2	3	3	2	4	4	6	10	0.278	0.178	8.28E-01	n.s.	P
Q	2	3	1	2	2	0	0	3	1	2	5	2	4	3	3	3	1	0	3	2	2	2	7	1	7	2	3	3	4	1	1	0	3	2	3	3	0	9	1	5	1	2	3	2	3	2	1	3	6	4	11	0.233	0.170	2.64E-03	n.s.	Q
R	0	0	4	3	5	0	3	4	1	1	1	0	0	4	2	3	3	3	0	4	3	1	3	6	5	5	3	2	3	1	4	2	3	1	3	4	1	5	3	0	2	10	3	1	2	6	2	5	5	2	26	0.105	0.077	5.55E-08	*	R
S	5	3	8	5	4	7	4	17	3	3	7	6	1	5	5	8	10	4	8	3	6	1	8	6	4	1	6	0	6	4	5	3	11	3	7	0	1	8	1	6	5	2	7	3	20	7	6	4	11	7	26	0.212	0.146	6.67E-05	*	S
T	3	1	3	4	7	3	6	4	2	2	1	6	10	5	5	7	7	10	4	2	3	3	2	10	4	8	2	2	3	2	5	3	2	3	2	4	5	4	2	1	7	3	4	5	3	7	4	1	3	8	8	0.518	0.301	–	–	T
V	6	3	7	6	3	3	3	0	1	0	2	4	3	5	4	9	5	6	4	2	4	6	3	6	5	5	10	6	7	8	6	4	9	1	2	8	12	6	8	5	4	5	6	3	5	0	9	3	2	3	9	0.527	0.296	–	–	V
W	1	1	1	1	0	1	0	2	1	2	1	0	0	1	0	0	1	1	1	0	1	2	1	1	0	0	0	0	0	0	1	2	0	0	0	1	1	0	1	2	0	0	0	0	1	1	0	3	0	0	0	–	–	–	–	W
Y	2	1	3	3	5	0	6	2	1	1	0	1	1	1	1	0	1	1	3	1	2	1	2	1	5	0	2	3	2	4	2	1	0	0	1	1	1	1	1	5	2	3	4	1	2	0	2	0	1	1	2	0.850	0.730	–	–	Y
total	50	59	82	70	60	42	62	76	51	58	62	40	59	79	55	75	72	65	47	53	73	53	65	91	58	55	66	71	58	49	59	46	61	51	56	60	67	80	76	64	63	63	69	55	72	58	63	53	77	59	216	0.288	0.050			total

position -3

	C50	C49	C48	C47	C46	C45	C44	C43	C42	C41	C40	C39	C38	C37	C36	C35	C34	C33	C32	C31	C30	C29	C28	C27	C26	C25	C24	C23	C22	C21	C20	C19	C18	C17	C16	C15	C14	C13	C12	C11	C10	C9	C8	C7	C6	C5	C4	C3	C2	C1	C0	C-index	SD	*P-value*	mark	
A	2	0	7	5	4	4	5	7	4	8	5	3	8	5	7	2	2	3	5	3	3	1	2	2	5	3	4	4	4	2	5	5	5	8	1	8	4	4	6	2	7	0	4	1	3	3	2	1	6	4	12	0.330	0.178	–	–	A
C	1	1	0	0	0	0	0	3	2	1	0	1	0	0	0	4	2	1	2	1	1	1	2	4	3	2	1	1	0	1	1	1	0	0	0	5	1	1	0	1	0	2	2	3	0	1	3	1	7	2	7	0.189	0.209	1.55E-04	n.s.	C
D	0	3	2	2	4	2	5	0	2	3	5	0	9	10	2	0	3	1	1	1	1	3	3	1	3	1	3	5	2	3	2	0	3	3	2	1	0	3	1	2	6	2	6	0	5	3	1	3	6	3	4	0.660	0.543	–	–	D
E	4	12	5	7	3	1	1	4	6	6	1	2	3	1	1	9	6	5	4	0	7	2	6	5	2	2	7	2	2	5	7	1	4	5	3	4	3	2	3	2	7	4	3	2	2	4	1	2	3	7	35	0.109	0.070	5.55E-08	*	E
F	2	1	2	2	3	2	2	3	2	2	2	1	2	2	3	6	2	4	2	1	3	4	2	3	2	0	4	6	0	5	2	2	2	4	3	1	9	6	4	3	2	2	2	3	5	3	0	4	1	1	2	1.340	0.854	–	–	F
G	2	3	2	6	4	1	1	7	5	1	2	1	4	6	1	4	3	0	1	8	3	2	2	2	7	6	4	2	4	2	3	4	3	1	3	2	1	4	7	1	6	6	4	2	3	4	3	6	6	1	17	0.195	0.120	1.17E-04	n.s.	G
H	0	1	1	1	0	1	1	0	0	0	2	1	1	2	1	1	1	4	4	3	1	5	7	3	3	1	3	2	3	1	0	3	0	1	1	6	5	0	0	1	2	1	3	1	2	4	6	1	6	1	3	0.653	0.613	–	–	H
I	4	9	5	5	4	3	6	0	5	4	3	2	3	7	0	2	5	2	1	3	2	4	1	4	2	3	7	1	3	1	2	2	7	1	5	0	3	6	2	3	2	3	3	5	0	1	9	5	2	3	14	0.236	0.157	5.38E-02	n.s.	I
K	5	2	6	3	2	1	5	1	3	1	3	3	3	7	4	4	6	2	3	4	3	1	3	4	4	4	1	6	6	2	2	3	4	4	1	2	10	9	10	2	4	2	8	3	7	4	8	5	10	1	7	0.574	0.358	–	–	K
L	7	1	6	7	7	5	4	6	1	5	9	8	3	0	8	9	7	6	3	5	11	4	5	11	7	5	2	7	3	5	3	3	7	4	4	4	6	11	5	12	4	6	7	5	16	3	5	3	4	6	11	0.518	0.273	–	–	L
M	1	4	2	2	3	2	1	2	1	2	1	0	2	2	2	2	1	5	2	2	3	0	5	0	1	0	2	0	0	3	3	0	0	2	2	3	0	0	2	1	1	7	1	0	0	0	2	2	1	0	5	0.320	0.300	–	–	M
N	1	2	4	0	3	1	4	8	3	4	1	1	6	13	1	4	3	7	3	2	5	3	2	4	2	0	2	0	1	3	4	3	3	2	1	5	3	8	6	7	0	1	1	1	6	2	1	1	3	3	7	0.440	0.361	–	–	N
P	2	1	1	3	5	5	2	3	2	3	4	2	4	4	2	2	1	5	2	3	3	4	4	3	3	2	5	3	1	4	5	1	1	2	4	5	3	3	2	1	1	3	3	3	2	6	3	1	1	6	6	0.480	0.237	–	–	P
Q	3	4	12	7	6	5	2	10	1	2	3	2	1	2	4	7	5	2	3	1	8	3	3	34	1	10	1	14	6	1	1	4	3	6	3	0	2	6	3	4	2	3	1	5	1	1	4	3	4	3	12	0.370	0.433	–	–	Q
R	1	3	5	2	5	2	7	2	3	2	3	2	2	3	2	1	3	2	3	3	5	2	3	1	2	2	5	3	1	0	5	5	2	0	2	4	1	1	1	0	7	2	4	3	2	5	6	1	3	0	5	0.536	0.349	–	–	R
S	2	3	9	6	2	2	3	4	4	2	0	5	5	1	1	4	4	2	3	1	5	2	3	3	3	4	1	3	5	2	8	4	5	0	6	1	4	6	4	6	4	5	7	7	7	3	5	6	6	3	20	0.191	0.104	7.27E-06	*	S
T	5	3	3	2	1	2	3	7	4	4	7	3	1	2	7	2	4	2	0	2	1	2	2	2	1	4	4	2	3	0	2	1	4	4	2	1	3	2	3	3	3	4	2	5	4	3	1	3	3	4	25	0.114	0.064	5.55E-08	*	T
V	5	0	8	5	1	3	7	6	1	7	4	1	2	5	8	5	5	7	4	5	3	2	1	3	2	3	6	8	8	5	4	3	5	2	9	4	3	3	12	7	3	8	7	3	3	5	2	1	0	5	16	0.274	0.163	9.92E-01	n.s.	V
W	1	1	1	4	0	0	0	2	0	1	2	0	0	3	0	4	4	2	0	2	4	0	1	0	1	0	2	0	1	2	0	0	1	1	1	0	0	1	1	4	0	2	0	0	2	1	1	0	3	1	0	–	–	–	–	W
Y	2	5	1	1	3	0	3	1	2	0	5	2	0	4	1	3	5	3	1	3	1	8	8	2	4	3	2	2	5	2	0	1	2	1	3	4	6	4	4	2	2	0	1	3	2	2	0	4	2	5	8	0.325	0.240	–	–	Y
total	50	59	82	70	60	42	62	76	51	58	62	40	59	79	55	75	72	65	47	53	73	53	65	91	58	55	66	71	58	49	59	46	61	51	56	60	67	80	76	64	63	63	69	55	72	58	63	53	77	59	216	0.288	0.050			total

position -1

	C50	C49	C48	C47	C46	C45	C44	C43	C42	C41	C40	C39	C38	C37	C36	C35	C34	C33	C32	C31	C30	C29	C28	C27	C26	C25	C24	C23	C22	C21	C20	C19	C18	C17	C16	C15	C14	C13	C12	C11	C10	C9	C8	C7	C6	C5	C4	C3	C2	C1	C0	C-index	SD	*P-value*	mark	
A	6	4	9	9	4	6	2	8	4	6	7	5	12	22	5	5	8	9	1	4	7	4	1	39	6	2	7	7	6	6	8	3	6	6	3	5	5	6	5	7	3	10	5	5	5	8	4	5	5	1	16	0.408	0.357	–	–	A
C	0	1	2	2	3	0	0	2	2	0	2	4	1	0	1	4	5	2	2	0	7	0	0	3	4	4	5	2	2	0	0	0	1	1	1	7	0	2	0	0	2	0	3	2	0	3	0	1	5	0	4	0.440	0.470	–	–	C
D	2	4	7	7	4	1	1	2	2	2	3	5	1	4	1	6	3	6	3	2	2	4	8	4	2	2	3	10	2	3	2	4	3	0	3	5	2	4	11	2	3	3	3	3	5	5	1	2	3	3	18	0.192	0.124	7.26E-07	*	D
E	2	2	5	2	7	4	5	5	3	3	5	1	1	5	3	3	9	9	3	2	4	6	4	3	5	5	3	6	6	6	1	2	3	6	2	3	2	3	7	5	7	1	4	3	9	1	8	2	9	3	21	0.198	0.109	4.62E-05	*	E
F	0	2	1	1	1	3	3	1	2	2	1	0	0	5	3	1	3	4	1	1	1	2	2	2	1	3	0	1	1	2	1	0	0	4	0	1	2	4	4	0	1	1	2	1	1	3	3	2	2	2	6	0.280	0.209	9.54E-01	n.s.	F
G	4	10	5	7	6	3	4	5	2	3	4	2	6	4	5	5	2	2	6	4	1	1	6	1	4	4	4	6	4	1	5	4	5	0	7	2	6	2	5	7	10	5	1	2	1	5	4	1	2	4	7	0.569	0.319	–	–	G
H	2	4	0	0	4	1	1	1	2	2	3	0	3	2	0	1	3	1	3	1	0	0	1	1	1	2	0	1	0	1	2	2	1	0	1	1	1	4	1	2	0	1	2	3	0	3	1	0	2	1	8	0.173	0.145	2.23E-05	*	H
I	1	1	2	3	0	3	0	0	2	3	2	2	3	2	1	1	1	4	0	3	4	0	1	2	0	1	3	2	3	2	2	3	2	0	2	4	5	1	2	3	0	2	6	2	5	3	3	1	2	3	6	0.343	0.237	–	–	I
K	1	1	2	1	4	0	1	2	2	3	3	3	1	1	3	2	3	0	0	10	3	5	3	2	3	1	2	3	5	2	2	2	1	3	4	6	5	2	3	5	7	4	1	3	1	0	0	3	4	4	7	0.377	0.276	–	–	K
L	2	7	13	7	5	1	4	10	3	4	3	1	1	3	2	10	5	0	3	6	3	6	6	4	9	2	2	2	5	2	3	7	4	1	3	4	0	11	2	7	5	5	3	6	15	4	0	3	2	2	11	0.396	0.298	–	–	L
M	0	1	4	0	1	4	2	2	1	1	3	1	3	2	0	1	0	1	2	0	4	3	0	0	0	0	1	0	1	1	3	1	0	0	2	0	1	2	3	1	1	0	3	0	1	1	1	0	1	2	5	0.248	0.241	4.83E-02	n.s.	M
N	2	0	0	0	1	0	4	7	2	5	6	0	1	2	3	1	2	2	1	3	2	1	1	4	2	2	2	3	0	1	0	1	0	3	2	2	5	0	1	0	1	2	5	1	1	0	1	3	2	3	8	0.233	0.210	1.81E-02	n.s.	N
P	2	2	5	4	7	2	4	8	3	3	3	1	5	6	2	3	5	2	3	3	3	1	9	3	5	5	4	5	8	3	8	1	6	3	0	3	2	9	6	4	1	3	12	2	4	5	9	9	5	3	19	0.225	0.137	1.56E-03	n.s.	P
Q	1	1	4	1	4	0	2	4	7	1	3	2	6	3	6	1	4	2	4	1	3	3	4	6	2	6	3	3	3	4	3	4	6	4	2	2	4	3	6	5	1	3	2	2	1	2	3	5	2	3	2	1.570	0.839	–	–	Q
R	3	2	5	3	3	3	6	0	1	3	0	3	3	4	4	6	5	1	2	2	4	1	6	6	2	1	7	1	0	3	2	0	2	3	8	0	11	5	3	3	3	2	0	4	2	0	6	5	1	1	12	0.252	0.195	1.42E-01	n.s.	R
S	5	7	7	6	3	7	3	3	7	3	3	4	6	7	5	11	2	7	4	6	12	6	4	7	4	6	9	1	5	4	3	6	5	6	5	4	9	6	10	2	4	8	4	8	6	2	4	3	14	8	17	0.331	0.157	–	–	S
T	5	3	5	6	0	1	8	8	2	1	1	2	2	0	4	2	5	6	6	0	5	5	1	2	4	7	1	12	4	1	8	2	7	3	4	4	1	8	1	7	3	7	9	4	9	5	3	4	6	7	29	0.146	0.097	5.56E-08	*	T
V	9	4	5	7	3	2	9	5	4	10	7	4	4	6	6	9	2	5	1	4	8	3	5	2	1	1	8	3	2	2	5	4	5	1	3	6	4	5	2	1	7	3	3	4	5	7	11	1	7	5	17	0.271	0.151	8.87E-01	n.s.	V
W	1	0	1	0	0	1	0	0	0	2	1	0	0	0	0	2	2	1	0	0	0	1	2	0	0	0	0	2	0	0	1	0	1	6	0	1	1	1	1	0	0	0	0	0	0	0	0	1	0	2	2	0.310	0.524	–	–	W
Y	2	3	0	4	0	0	3	3	0	1	2	0	0	1	1	1	3	1	2	1	0	1	1	0	3	1	2	1	1	5	0	0	3	1	4	0	1	2	3	3	4	3	1	0	1	1	1	2	3	2	1	1.560	1.327	–	–	Y
total	50	59	82	70	60	42	62	76	51	58	62	40	59	79	55	75	72	65	47	53	73	53	65	91	58	55	66	71	58	49	59	46	61	51	56	60	67	80	76	64	63	63	69	55	72	58	63	53	77	59	216	0.288	0.050			total

Additional file 5. Continued.

ZEBRAFISH x-x-V-x-I

position -4

	C50	C49	C48	C47	C46	C45	C44	C43	C42	C41	C40	C39	C38	C37	C36	C35	C34	C33	C32	C31	C30	C29	C28	C27	C26	C25	C24	C23	C22	C21	C20	C19	C18	C17	C16	C15	C14	C13	C12	C11	C10	C9	C8	C7	C6	C5	C4	C3	C2	C1	C0	C-index	SD	*P-value*	mark	
A	5	4	4	4	2	0	3	6	3	4	2	5	8	2	3	1	2	6	4	11	1	0	9	3	2	1	7	0	11	4	4	1	3	3	2	2	6	2	4	3	6	1	2	2	2	3	0	7	2	2	1	3.480	2.597	–	–	A
C	2	1	1	2	4	1	2	2	3	3	1	2	0	0	1	0	1	2	4	2	1	1	0	2	0	1	0	1	2	1	4	1	0	2	0	0	0	3	2	1	1	1	5	7	1	1	1	1	0	1	2	0.750	0.716	1.49E-05	*	C
D	5	2	0	3	2	4	2	0	4	3	0	0	3	2	3	2	5	3	7	1	3	5	4	3	5	4	2	2	1	1	5	0	0	4	1	0	3	5	3	3	0	3	4	1	5	1	2	2	3	0	1	2.520	1.764	–	–	D
E	2	1	5	5	3	3	0	3	2	2	1	2	3	2	4	3	3	4	5	4	3	1	4	0	4	3	3	4	3	0	8	4	1	0	1	3	4	3	5	0	5	2	6	2	5	3	3	3	5	2	6	0.490	0.285	5.55E-08	*	E
F	2	3	1	11	1	4	2	2	5	4	2	3	1	0	2	3	1	2	2	0	1	1	1	5	2	1	1	1	0	2	3	4	2	2	2	0	0	1	1	1	2	5	3	2	2	0	0	3	3	4	0	–	–	–	–	F
G	2	7	9	7	0	5	12	5	3	6	8	4	4	4	6	3	2	10	3	6	2	2	1	8	4	22	13	2	4	6	5	2	4	1	2	3	7	3	5	2	1	3	1	5	10	3	2	2	0	0	2	2.310	1.966	–	–	G
H	3	0	2	2	0	3	1	3	1	0	0	0	2	2	0	1	1	3	0	1	0	1	0	1	0	2	1	0	1	1	2	1	1	0	9	2	3	2	1	1	1	1	2	0	1	2	3	0	1	1	0	–	–	–	–	H
I	3	2	2	3	3	2	6	5	2	0	5	3	4	0	4	1	0	3	0	3	1	0	0	6	3	3	2	2	5	4	4	4	4	4	3	2	2	3	2	5	6	0	0	2	0	3	1	4	2	1	0	–	–	–	–	I
K	3	1	2	4	3	2	6	0	2	0	3	6	3	4	2	0	2	5	1	2	2	3	3	1	5	3	0	4	6	2	9	0	4	3	25	3	2	2	1	4	2	3	1	6	1	1	4	0	3	3	0	–	–	–	–	K
L	6	3	10	5	8	3	7	3	4	7	4	3	3	8	9	5	6	4	7	5	3	3	8	4	6	1	2	5	6	1	1	6	4	5	6	4	6	5	10	1	2	4	5	4	2	7	1	6	2	3	3	1.553	0.778	–	–	L
M	2	0	0	0	2	1	0	0	2	0	0	6	0	0	0	0	1	0	1	2	0	1	0	0	0	0	1	1	1	4	0	1	0	1	1	3	2	0	1	0	1	0	0	3	0	0	2	0	1	0	1	0.820	1.224	1.02E-03	n.s.	M
N	4	1	2	1	0	2	1	0	11	3	0	1	2	5	1	0	6	2	1	5	3	1	0	1	4	1	1	0	0	1	2	2	3	4	2	0	1	0	3	2	1	2	1	7	4	3	0	1	1	1	5	0.400	0.422	5.55E-08	*	N
P	4	0	2	5	3	2	8	2	2	1	1	3	3	1	3	0	2	4	2	2	2	0	2	2	0	3	3	3	7	5	1	0	3	3	5	6	1	4	5	5	2	2	0	2	3	2	0	2	3	3	6	0.430	0.303	5.55E-08	*	P
Q	5	3	2	3	3	4	2	2	1	3	2	5	2	1	1	5	1	1	0	2	2	1	2	3	2	0	2	3	3	0	0	2	2	6	4	3	1	3	0	2	1	1	1	2	4	1	1	2	1	3	7	0.303	0.201	5.55E-08	*	Q
R	4	6	2	3	2	1	1	2	0	5	1	1	6	2	1	1	0	2	1	3	2	2	4	4	5	4	5	4	4	5	7	4	3	4	2	1	3	2	2	2	3	5	2	3	1	0	4	8	1	1	2	1.410	0.930	–	–	R
S	5	5	4	5	6	4	1	1	5	4	2	3	8	0	1	0	5	4	2	2	1	3	0	9	1	4	2	3	5	4	4	5	3	1	1	4	3	4	2	7	2	7	6	2	4	4	8	6	3	4	4	0.895	0.542	4.58E-02	n.s.	S
T	4	2	4	6	5	2	3	0	2	6	2	3	9	2	8	12	0	5	2	0	2	6	2	2	3	1	2	2	5	2	2	1	5	2	3	1	2	2	4	0	10	4	0	2	3	3	4	1	1	2	4	0.780	0.640	4.69E-05	*	T
V	11	9	8	5	6	8	3	12	16	5	8	7	7	6	3	2	4	3	6	2	4	4	12	1	4	6	3	8	3	3	4	6	3	6	7	5	3	24	4	2	5	4	1	4	0	1	1	5	1	2	1	5.340	4.192	–	–	V
W	0	2	0	0	0	0	0	0	0	0	0	0	0	0	0	0	2	0	2	3	0	0	0	1	0	1	1	0	0	0	0	3	1	0	1	0	0	0	0	1	1	1	0	0	0	0	0	1	0	3	0	–	–	–	–	W
Y	1	1	1	2	0	0	2	0	2	0	1	1	0	2	2	2	10	2	0	1	1	0	2	3	1	1	0	1	1	1	2	0	3	0	0	0	0	0	1	2	0	1	0	0	1	0	0	1	2	1	2	0.550	0.778	5.60E-08	*	Y
total	73	53	61	76	53	51	62	48	70	56	43	58	68	43	54	41	54	65	50	57	34	35	54	59	51	62	51	46	68	47	67	47	49	51	77	42	49	68	56	44	52	50	40	56	49	38	37	55	35	37	47	1.124	0.232			total

position -3

	C50	C49	C48	C47	C46	C45	C44	C43	C42	C41	C40	C39	C38	C37	C36	C35	C34	C33	C32	C31	C30	C29	C28	C27	C26	C25	C24	C23	C22	C21	C20	C19	C18	C17	C16	C15	C14	C13	C12	C11	C10	C9	C8	C7	C6	C5	C4	C3	C2	C1	C0	C-index	SD	*P-value*	mark	
A	7	2	7	3	7	5	3	10	25	3	9	8	8	9	7	12	2	4	4	0	3	1	3	3	2	4	0	6	7	1	2	1	4	6	11	7	3	3	5	3	2	2	1	0	0	4	0	7	4	2	2	2.320	2.116	–	–	A
C	0	5	1	5	5	2	3	0	0	1	0	1	2	0	2	0	0	0	6	4	0	4	2	1	1	1	1	0	1	2	1	2	0	0	0	0	0	0	3	0	1	2	2	11	1	0	2	0	2	0	1	1.540	2.092	–	–	C
D	14	5	5	2	2	4	9	2	2	2	2	1	2	0	2	1	1	2	1	3	2	3	1	1	3	2	1	4	2	5	5	1	0	2	3	1	1	4	7	2	9	1	3	2	3	0	4	3	2	3	2	1.420	1.267	–	–	D
E	2	1	2	4	1	3	2	0	1	3	1	2	8	2	2	0	1	2	2	2	4	1	2	2	4	2	1	4	5	3	4	4	1	4	2	1	2	2	4	6	1	1	0	3	4	0	1	4	1	0	5	0.456	0.331	5.55E-08	*	E
F	3	1	1	9	4	0	0	3	1	3	2	1	2	0	2	0	1	2	0	2	1	1	3	1	0	3	1	4	2	1	3	2	3	2	1	1	1	2	1	1	1	3	3	3	4	2	0	5	3	3	1	1.960	1.603	–	–	F
G	4	3	6	3	0	3	3	5	3	3	4	3	5	1	5	3	4	10	3	1	3	5	7	4	4	20	7	2	2	2	3	3	6	1	4	2	1	23	6	0	1	3	1	4	6	2	2	0	1	2	2	1.990	2.074	–	–	G
H	1	3	1	1	1	1	0	0	0	0	2	3	2	0	1	2	0	2	5	1	2	0	0	0	3	0	1	0	1	2	3	1	2	0	1	0	2	1	4	1	1	0	0	0	3	1	0	1	0	2	1	1.160	1.201	–	–	H
I	2	1	2	3	4	1	4	1	4	1	3	3	4	1	6	2	7	1	3	4	3	2	0	2	0	2	1	3	3	2	1	3	2	2	7	3	1	2	2	2	1	0	2	3	1	2	1	1	0	2	0	–	–	–	–	I
K	2	3	4	1	1	2	7	3	7	3	1	2	0	1	2	3	7	1	2	5	1	3	2	2	3	4	2	1	5	2	4	0	3	1	3	1	1	3	0	3	5	5	3	9	5	4	3	1	0	2	5	0.552	0.399	5.56E-08	*	K
L	6	8	6	9	2	3	9	6	6	12	1	13	9	2	5	4	2	4	6	11	3	6	12	6	6	5	4	4	2	8	5	7	2	3	3	2	8	6	6	4	4	5	2	3	2	3	7	15	0	3	1	5.400	3.289	–	–	L
M	2	2	1	4	1	1	1	2	1	0	0	0	1	0	2	1	2	1	2	1	0	1	0	1	1	1	2	1	1	0	0	0	1	1	1	0	3	1	1	0	1	0	1	0	1	0	1	0	4	0	0	–	–	–	–	M
N	2	5	4	4	1	3	0	3	2	2	2	2	3	2	0	0	2	3	0	2	0	2	0	5	2	2	2	2	0	2	5	2	1	2	1	3	4	4	2	2	1	0	0	1	2	0	1	3	1	2	2	0.960	0.699	1.68E-01	n.s.	N
P	1	2	0	3	1	3	1	1	1	3	0	3	2	1	5	2	2	5	2	1	1	0	2	2	2	4	2	0	2	2	3	5	4	3	8	5	2	2	5	1	6	0	1	0	1	1	4	3	4	1	5	0.460	0.353	5.55E-08	*	P
Q	3	1	2	1	1	8	5	2	1	5	0	1	4	2	0	1	0	2	0	2	1	0	5	3	7	2	4	0	7	1	6	4	3	2	0	3	1	0	1	1	1	5	0	2	4	3	0	2	4	3	1	2.320	2.075	–	–	Q
R	2	1	3	4	2	0	3	2	1	2	4	4	1	2	3	3	1	2	3	4	1	0	2	6	3	2	1	5	1	2	4	0	1	1	25	1	5	1	2	3	1	1	7	2	3	0	1	1	1	4	0	–	–	–	–	R
S	8	5	5	5	10	3	2	1	7	4	5	1	3	3	1	1	11	4	4	2	4	0	4	11	3	1	2	1	4	2	3	1	4	7	1	3	2	3	1	7	4	7	4	5	5	1	6	2	5	4	14	0.274	0.186	5.55E-08	*	S
T	3	2	5	10	5	5	1	2	2	4	3	3	3	3	0	2	3	12	5	2	3	4	5	7	3	3	5	4	14	0	7	4	7	5	2	7	3	3	1	1	2	7	4	5	0	5	2	6	0	1	3	1.300	0.953	–	–	T
V	10	1	0	2	4	2	7	3	3	5	4	5	3	12	7	4	4	8	1	6	2	2	1	1	1	2	12	4	7	10	7	6	2	5	2	1	6	3	5	6	6	7	4	1	1	6	0	1	1	0	2	2.030	1.530	–	–	V
W	0	0	1	2	0	0	0	0	1	0	0	0	0	1	1	0	1	0	0	0	0	0	0	0	3	1	0	0	0	0	0	0	1	1	1	0	1	3	0	0	0	1	0	0	1	1	2	0	1	2	0	–	–	–	–	W
Y	1	2	5	1	1	2	2	2	2	0	0	2	6	1	1	0	3	0	1	4	0	0	3	1	0	1	2	1	2	0	1	1	2	3	1	1	2	2	0	1	4	0	2	2	2	3	0	0	1	1	0	–	–	–	–	Y
total	73	53	61	76	53	51	62	48	70	56	43	58	68	43	54	41	54	65	50	57	34	35	54	59	51	62	51	46	68	47	67	47	49	51	77	42	49	68	56	44	52	50	40	56	49	38	37	55	35	37	47	1.124	0.232			total

position -1

	C50	C49	C48	C47	C46	C45	C44	C43	C42	C41	C40	C39	C38	C37	C36	C35	C34	C33	C32	C31	C30	C29	C28	C27	C26	C25	C24	C23	C22	C21	C20	C19	C18	C17	C16	C15	C14	C13	C12	C11	C10	C9	C8	C7	C6	C5	C4	C3	C2	C1	C0	C-index	SD	*P-value*	mark	
A	1	6	6	7	6	2	4	3	3	7	8	0	0	1	4	4	1	7	2	2	1	3	9	2	4	3	1	2	1	0	1	1	3	2	2	0	1	5	2	1	1	1	2	5	5	1	3	0	3	2	4	0.705	0.573	6.50E-05	*	A
C	2	1	2	0	1	3	0	0	1	0	1	1	2	0	0	1	1	1	5	3	0	2	2	4	2	1	2	2	0	1	0	0	0	2	3	0	1	1	1	0	1	0	1	1	1	2	0	9	3	0	1	1.340	1.599	–	–	C
D	9	2	0	3	2	3	8	3	0	1	4	4	6	2	3	1	2	1	1	1	2	3	2	2	2	2	1	2	2	4	4	2	1	3	2	7	4	1	4	1	3	2	8	1	6	4	1	1	1	3	1	2.740	2.058	–	–	D
E	6	5	1	8	0	3	3	7	14	3	5	8	8	3	1	1	1	1	6	2	3	0	1	3	5	0	2	3	3	3	8	2	7	2	6	2	1	4	1	10	3	2	7	4	4	0	5	1	4	4	2	1.860	1.461	–	–	E
F	2	7	9	2	3	2	1	3	14	3	1	3	2	4	4	0	7	4	5	1	1	2	2	2	1	3	2	1	1	4	5	0	0	6	1	2	2	7	4	1	1	6	2	1	1	1	1	2	3	3	2	1.450	1.299	–	–	F
G	2	1	4	4	2	5	6	1	1	5	0	3	3	5	6	14	3	9	4	11	0	4	3	10	2	3	2	1	5	1	4	5	7	3	1	4	2	17	6	3	4	3	1	3	0	6	0	1	3	0	2	1.930	1.735	–	–	G
H	7	0	1	1	4	1	2	1	2	3	1	0	3	2	2	0	0	1	1	3	3	0	0	0	2	0	1	0	0	1	0	0	1	0	4	1	0	0	2	0	4	0	1	0	2	0	1	5	1	1	3	0.433	0.514	5.58E-08	*	H
I	1	1	4	3	5	6	12	6	4	5	2	3	2	2	4	1	4	3	1	1	4	2	3	5	4	4	1	2	12	4	1	1	3	1	0	1	5	0	2	3	7	2	3	4	5	2	5	0	3	1	1	3.200	2.499	–	–	I
K	2	2	5	1	2	4	2	0	1	2	5	3	3	1	0	1	4	9	1	3	0	2	3	3	10	2	4	2	5	1	4	1	3	6	6	2	2	3	3	1	3	2	2	2	2	0	2	5	1	5	8	0.345	0.259	5.55E-08	*	K
L	11	3	5	12	6	4	3	5	3	6	4	9	10	6	8	3	3	2	5	4	8	4	4	4	4	2	4	4	10	7	4	14	6	7	3	4	6	10	3	5	5	9	5	5	4	7	4	3	1	3	1	5.420	2.793	–	–	L
M	1	2	1	0	2	0	0	1	2	0	1	0	0	0	0	2	1	2	0	2	0	2	1	1	0	0	0	0	3	1	0	0	1	1	0	1	0	0	0	0	2	2	0	0	2	1	0	2	0	0	0	–	–	–	–	M
N	6	3	4	8	1	2	2	3	3	2	1	3	0	3	3	1	2	2	1	4	2	1	3	7	1	1	1	2	4	3	2	0	1	1	3	4	3	1	3	5	3	3	0	5	0	0	4	3	2	4	1	2.520	1.752	–	–	N
P	2	2	1	2	1	2	0	1	1	3	1	0	3	0	1	1	1	1	5	4	2	1	6	1	1	7	4	4	1	4	1	1	4	3	2	2	1	4	4	3	2	1	0	5	2	2	5	1	1	2	1	2.180	1.650	–	–	P
Q	4	5	5	3	2	0	6	2	0	1	0	1	7	0	5	3	2	1	0	1	1	2	1	5	2	1	2	1	2	3	5	5	4	1	3	0	3	1	5	6	1	0	1	8	3	4	0	4	4	0	3	0.840	0.701	5.79E-02	n.s.	Q
R	2	2	2	3	5	0	4	2	4	1	2	2	5	0	0	3	6	3	1	1	2	1	1	0	1	2	5	5	6	1	2	1	1	3	0	1	3	3	2	0	4	4	1	1	4	2	1	3	0	1	1	2.180	1.662	–	–	R
S	4	2	3	5	4	2	2	3	5	2	0	8	6	3	3	2	1	4	4	3	2	1	6	1	4	3	3	6	3	1	3	4	1	7	4	4	7	3	1	4	5	4	3	1	2	3	1	4	1	2	4	0.800	0.449	9.80E-05	*	S
T	8	2	2	1	3	4	3	4	3	4	1	3	2	0	4	1	2	6	0	4	1	1	2	5	0	23	11	4	8	2	11	4	1	2	26	3	2	1	4	0	0	3	2	8	1	0	2	6	1	4	3	1.300	1.665	–	–	T
V	2	3	3	3	2	5	3	1	6	4	2	6	4	9	1	2	3	4	5	5	1	3	2	3	3	3	4	4	1	6	8	3	4	0	4	3	5	4	4	1	0	3	1	1	2	0	1	5	3	0	4	0.775	0.493	9.56E-05	*	V
W	1	0	0	3	0	0	1	0	0	2	0	1	0	1	2	0	10	1	2	0	1	1	2	1	2	0	0	1	0	0	0	1	0	0	0	1	0	1	1	0	2	0	0	0	2	0	0	0	0	0	3	0.267	0.517	5.55E-08	*	W
Y	0	4	3	7	2	3	0	2	3	2	4	0	2	1	3	0	0	3	1	2	0	0	1	0	1	2	1	0	1	0	4	2	1	1	7	0	1	2	4	0	1	3	0	1	1	3	1	0	0	2	2	0.820	0.844	9.45E-03	n.s.	Y
total	73	53	61	76	53	51	62	48	70	56	43	58	68	43	54	41	54	65	50	57	34	35	54	59	51	62	51	46	68	47	67	47	49	51	77	42	49	68	56	44	52	50	40	56	49	38	37	55	35	37	47	1.124	0.232			total

Additional file 5. Continued.

ZEBRAFISH x-x-V-x-V

position -4

	C50	C49	C48	C47	C46	C45	C44	C43	C42	C41	C40	C39	C38	C37	C36	C35	C34	C33	C32	C31	C30	C29	C28	C27	C26	C25	C24	C23	C22	C21	C20	C19	C18	C17	C16	C15	C14	C13	C12	C11	C10	C9	C8	C7	C6	C5	C4	C3	C2	C1	C0	C-index	SD	*P-value*	mark	
A	1	2	3	3	9	8	6	6	3	5	8	6	5	24	5	13	5	6	2	5	3	0	4	5	6	7	4	2	7	8	7	3	5	10	2	15	10	2	7	4	3	5	3	4	4	6	4	2	6	2	9	0.611	0.439	1.35E-07	✻	A
C	1	4	0	6	1	3	0	2	1	2	0	2	0	2	4	1	2	3	1	1	0	1	2	6	0	2	1	1	2	2	5	1	2	2	1	1	0	1	0	0	0	1	0	3	2	4	2	1	2	1	1	1.640	1.495	–	–	C
D	5	10	7	5	3	6	5	8	4	2	1	6	4	4	6	4	4	14	3	8	9	3	4	6	10	5	8	4	3	3	8	2	3	2	2	2	2	3	2	3	5	1	5	3	1	2	4	3	5	6	3	1.520	0.891	–	–	D
E	5	13	9	4	6	6	5	5	3	10	7	1	3	3	3	3	7	8	3	8	4	3	5	5	4	14	4	3	2	4	2	4	3	5	3	8	6	5	5	4	3	1	7	7	2	7	4	5	2	5	5	0.992	0.539	–	–	E
F	1	4	3	2	3	8	7	2	5	2	3	5	3	4	2	2	3	3	6	3	7	3	2	2	3	1	4	2	4	0	1	1	3	5	1	1	4	2	1	3	1	2	1	7	3	4	1	11	1	4	2	1.560	1.077	–	–	F
G	4	3	5	6	4	5	6	6	5	3	1	5	7	11	3	4	6	3	5	7	4	9	1	6	3	4	3	5	1	5	4	3	3	3	4	6	5	9	3	2	1	3	4	5	10	4	7	1	3	6	5	0.904	0.449	9.83E-01	n.s.	G
H	3	8	3	1	0	1	1	1	2	2	1	0	2	6	4	2	4	2	3	2	2	0	3	1	0	0	1	0	1	2	1	1	2	2	0	1	3	2	1	5	1	1	2	1	2	1	2	1	2	0	1	1.780	1.569	–	–	H
I	6	9	6	14	12	3	4	2	3	9	4	4	2	7	7	1	5	8	6	4	4	3	3	4	2	7	4	3	5	3	6	2	3	5	2	4	2	2	4	5	3	6	5	4	0	2	5	1	1	3	4	1.095	0.677	–	–	I
K	3	3	2	5	4	6	10	3	3	10	6	8	12	3	2	2	1	3	3	3	1	6	2	4	4	7	5	5	2	2	2	4	2	1	2	1	3	4	4	4	3	7	1	7	4	7	2	4	4	4	3	1.333	0.822	–	–	K
L	11	7	4	9	13	7	12	6	9	7	16	4	7	5	11	4	10	5	5	13	11	12	13	7	3	9	6	5	5	6	4	6	4	23	7	4	5	5	4	3	2	6	5	8	5	3	6	8	3	3	7	1.017	0.568	–	–	L
M	3	2	1	4	1	4	1	1	2	2	1	1	1	0	3	0	2	1	2	1	2	4	0	0	1	4	1	1	1	0	1	1	5	2	1	1	1	1	4	6	1	1	1	2	1	1	0	0	3	0	3	0.533	0.467	9.60E-07	✻	M
N	1	1	4	4	1	3	1	3	1	5	17	2	5	10	3	3	4	2	5	0	2	3	2	1	2	4	5	2	0	1	0	4	6	5	2	5	5	1	2	0	2	2	3	2	7	3	1	6	2	3	7	0.451	0.405	5.55E-08	✻	N
P	7	4	3	5	4	8	4	3	4	4	7	6	3	5	5	4	8	3	10	5	3	3	10	3	3	2	4	5	1	4	2	6	3	0	4	4	4	2	3	4	9	3	2	2	4	4	4	2	1	6	4	1.045	0.543	–	–	P
Q	3	3	3	7	6	7	4	3	4	4	2	2	1	2	0	3	6	3	1	1	4	6	3	7	3	4	4	1	3	4	5	0	2	7	4	1	3	3	3	1	1	3	3	3	5	3	8	1	2	5	3	1.113	0.648	–	–	Q
R	5	5	3	3	6	2	9	7	4	4	2	1	7	4	5	3	1	1	5	3	5	2	2	3	4	1	1	8	8	4	4	3	2	4	3	2	4	5	3	3	2	3	5	2	1	1	1	4	6	5	5	0.724	0.402	5.49E-03	n.s.	R
S	5	4	6	9	1	6	7	4	5	4	2	4	4	3	8	5	5	8	3	3	6	3	4	3	4	3	5	6	5	1	6	7	3	3	7	7	4	3	5	5	5	6	8	4	4	4	5	3	4	6	3	1.567	0.592	–	–	S
T	2	4	3	0	12	2	5	5	6	7	5	3	5	4	5	8	1	9	8	2	2	3	5	4	4	5	7	4	5	0	1	1	3	6	5	9	2	0	8	3	1	4	2	2	3	3	2	6	4	3	10	0.406	0.257	5.55E-08	✻	T
V	8	8	6	7	3	10	5	5	8	2	7	5	16	7	11	4	7	8	8	14	14	7	9	12	4	6	7	5	10	13	5	4	10	4	7	8	8	2	5	3	11	1	3	3	3	7	8	6	1	2	5	1.348	0.698	–	–	V
W	0	0	0	2	1	0	1	0	0	2	2	0	0	0	11	2	0	0	3	0	0	1	0	1	1	0	1	2	3	0	2	1	0	1	0	2	1	0	1	0	1	3	0	0	0	1	0	0	1	1	1	0.960	1.714	–	–	W
Y	3	3	1	4	2	2	9	6	6	4	3	7	2	0	1	2	1	1	2	3	2	1	2	2	3	0	8	1	2	3	2	1	1	3	1	3	0	4	4	1	1	0	0	0	0	2	2	2	0	1	2	1.140	1.025	–	–	Y
total	77	97	72	100	92	97	102	78	78	90	95	72	89	104	99	70	82	91	84	86	85	73	76	82	64	85	83	65	70	65	68	55	65	93	58	85	72	56	69	59	56	59	60	69	61	69	68	67	53	66	83	0.918	0.170			total

position -3

	C50	C49	C48	C47	C46	C45	C44	C43	C42	C41	C40	C39	C38	C37	C36	C35	C34	C33	C32	C31	C30	C29	C28	C27	C26	C25	C24	C23	C22	C21	C20	C19	C18	C17	C16	C15	C14	C13	C12	C11	C10	C9	C8	C7	C6	C5	C4	C3	C2	C1	C0	C-index	SD	*P-value*	mark	
A	7	11	6	13	6	5	15	2	7	7	4	4	9	5	9	10	2	6	7	4	11	10	8	3	2	12	9	8	1	6	6	4	1	4	0	5	2	5	7	4	3	3	9	8	3	3	8	7	4	2	7	0.849	0.478	8.79E-01	n.s.	A
C	2	7	0	0	5	1	1	1	2	5	4	2	4	3	4	5	6	5	3	10	2	5	2	0	1	8	4	3	6	1	4	1	3	0	4	0	4	0	4	1	9	1	4	1	1	1	1	2	2	3	3	0.987	0.800	–	–	C
D	3	2	4	4	6	3	7	1	8	4	4	1	1	5	10	5	11	1	10	5	7	4	4	3	1	1	5	4	2	6	3	2	3	1	0	1	7	2	3	7	2	1	2	0	5	1	4	5	2	2	2	1.850	1.322	–	–	D
E	3	5	3	4	3	5	3	4	5	8	5	2	4	0	8	2	6	10	4	2	5	3	5	5	3	3	4	3	5	3	6	2	4	5	1	3	7	5	6	1	6	2	5	2	5	3	3	7	4	1	8	0.508	0.248	5.55E-08	✻	E
F	5	7	2	2	0	1	3	2	1	3	7	0	0	0	0	1	3	10	3	1	1	0	1	0	1	1	2	3	3	6	4	4	5	6	0	5	4	1	1	1	1	4	0	1	0	4	0	2	0	4	3	0.773	0.766	1.85E-01	n.s.	F
G	7	8	9	15	18	6	13	10	4	11	9	6	8	13	7	3	9	4	5	3	11	2	2	5	2	3	1	1	6	6	10	7	5	6	10	1	4	4	10	6	3	11	6	4	3	7	5	6	4	8	6	1.090	0.624	–	–	G
H	2	1	1	1	0	1	0	2	3	1	1	1	1	0	6	4	2	1	5	1	0	4	1	2	3	1	1	0	2	0	1	2	0	1	2	6	0	4	2	2	0	1	0	1	0	3	0	2	2	1	5	0.312	0.303	5.55E-08	✻	H
I	0	3	3	6	2	3	5	3	2	2	8	2	2	6	5	4	4	3	1	4	8	3	4	1	4	2	3	3	3	3	1	2	8	4	8	3	1	3	0	2	1	4	1	6	4	9	2	2	1	1	2	1.650	1.094	–	–	I
K	5	4	10	7	5	6	5	2	6	5	3	12	7	7	5	3	1	6	5	3	5	7	2	6	6	8	3	4	0	4	1	2	0	4	1	1	4	4	2	0	6	3	3	4	2	6	3	3	4	7	5	0.848	0.499	9.26E-01	n.s.	K
L	6	9	7	12	4	16	8	6	3	8	5	3	12	31	6	6	9	9	3	10	6	4	6	10	6	3	9	3	7	4	7	4	4	12	4	6	4	0	4	3	2	4	6	8	1	3	10	4	5	3	5	1.300	0.952	–	–	L
M	4	2	2	4	3	1	2	4	0	1	3	4	3	2	0	0	2	1	0	5	1	2	4	4	2	0	5	4	1	2	2	1	0	1	0	2	3	0	0	1	0	1	0	1	1	0	0	0	1	1	3	0.553	0.507	1.32E-04	n.s.	M
N	0	1	0	0	1	3	6	2	3	3	4	1	5	2	1	2	4	5	4	5	1	0	2	1	2	0	5	1	1	0	2	3	1	13	1	6	0	3	3	2	1	2	2	5	3	4	8	3	0	3	1	2.600	2.407	–	–	N
P	6	4	6	6	6	5	4	4	4	6	2	8	8	7	1	2	5	6	4	1	4	5	6	16	8	5	3	5	7	2	3	4	5	2	3	3	5	4	6	7	3	4	5	4	3	3	3	5	2	0	5	0.920	0.498	–	–	P
Q	2	3	4	6	5	7	5	3	3	4	16	6	2	3	3	5	1	3	1	4	1	4	4	6	5	2	6	1	7	0	1	2	2	0	2	1	3	1	3	1	1	3	1	9	2	2	1	4	2	1	4	0.820	0.683	1.09E-01	n.s.	Q
R	4	11	7	6	5	3	7	5	2	4	2	2	4	0	1	1	1	5	7	4	2	0	8	1	3	8	7	8	3	5	4	2	4	2	4	4	1	5	4	5	2	1	3	0	0	4	1	2	5	1	2	1.800	1.254	–	–	R
S	8	4	4	4	8	4	4	10	10	1	4	6	3	6	11	4	11	5	5	5	4	12	4	6	6	9	7	4	6	7	3	4	6	8	4	8	8	3	4	4	7	3	7	3	8	6	5	0	5	10	12	0.480	0.218	5.55E-08	✻	S
T	4	9	2	4	8	12	9	3	6	5	6	7	5	3	8	4	3	5	7	6	5	5	6	5	3	7	3	7	4	1	2	5	5	7	3	9	4	5	3	7	6	5	2	3	8	7	7	5	1	9	4	1.325	0.585	–	–	T
V	4	4	1	5	4	6	4	14	6	9	7	4	7	9	13	5	1	5	7	10	9	1	4	6	2	7	3	2	3	6	4	3	7	11	10	3	6	3	6	3	2	5	4	5	7	1	1	7	3	6	3	1.767	1.013	–	–	V
W	2	1	1	0	1	2	0	0	1	0	1	0	1	0	1	3	0	1	1	1	0	0	0	1	1	4	1	0	0	1	1	0	0	3	1	18	0	1	1	2	0	0	0	1	1	1	5	0	2	0	1	1.240	2.646	–	–	W
Y	3	1	0	1	2	7	1	0	2	3	0	1	3	2	0	1	1	0	2	2	2	2	3	1	3	1	2	1	3	2	3	1	2	3	0	0	5	3	0	0	1	1	0	3	4	1	1	1	4	3	2	0.880	0.732	9.43E-01	n.s.	Y
total	77	97	72	100	92	97	102	78	78	90	95	72	89	104	99	70	82	91	84	86	85	73	76	82	64	85	83	65	70	65	68	55	65	93	58	85	72	56	69	59	56	59	60	69	61	69	68	67	53	66	83	0.918	0.170			total

position -1

	C50	C49	C48	C47	C46	C45	C44	C43	C42	C41	C40	C39	C38	C37	C36	C35	C34	C33	C32	C31	C30	C29	C28	C27	C26	C25	C24	C23	C22	C21	C20	C19	C18	C17	C16	C15	C14	C13	C12	C11	C10	C9	C8	C7	C6	C5	C4	C3	C2	C1	C0	C-index	SD	*P-value*	mark	
A	5	8	4	7	6	15	20	13	8	8	8	6	9	3	5	4	5	4	10	5	6	10	2	16	4	7	12	4	10	3	2	4	6	2	6	3	7	2	4	1	5	1	3	8	1	5	1	1	4	6	4	1.495	1.013	–	–	A
C	5	3	7	5	1	4	3	3	6	5	2	3	7	11	5	5	6	8	2	14	11	9	6	1	3	2	6	6	3	3	2	1	3	2	7	2	5	2	8	0	6	8	6	2	2	4	5	8	1	2	1	4.620	2.962	–	–	C
D	5	3	2	9	3	3	4	2	1	5	4	1	2	4	11	5	10	3	3	3	4	6	3	1	0	4	0	5	4	5	4	1	2	4	3	6	2	2	2	0	2	2	5	2	9	4	1	3	3	1	5	0.692	0.484	1.87E-04	n.s.	D
E	4	5	8	1	4	5	3	4	0	3	4	6	3	1	9	4	4	3	7	5	1	5	2	3	1	9	3	4	6	7	4	1	1	4	1	2	4	2	4	2	1	2	4	7	7	5	1	6	4	2	6	0.627	0.371	2.17E-05	✻	E
F	2	5	3	7	11	10	8	3	5	3	17	5	3	23	4	5	2	3	4	4	4	1	0	4	2	2	4	5	4	6	1	2	0	8	3	1	4	3	0	5	3	1	1	3	0	1	4	0	6	4	7	0.597	0.588	1.41E-07	✻	F
G	5	8	5	4	6	6	5	3	5	4	7	1	6	3	4	13	3	5	3	4	3	3	3	11	2	6	4	8	3	4	3	1	1	4	3	21	9	1	1	3	3	2	2	5	2	1	6	3	0	1	0	–	–	–	–	G
H	2	2	1	0	2	0	7	2	1	2	4	5	1	4	3	1	3	2	4	3	0	2	3	1	2	4	0	1	1	0	1	2	3	2	1	2	0	0	6	1	1	0	1	0	1	2	1	6	1	2	3	0.640	0.559	7.75E-05	✻	H
I	2	1	2	5	3	3	2	3	6	3	5	3	6	1	10	1	9	6	8	5	3	2	4	7	2	2	2	0	0	7	6	4	6	4	0	1	0	8	5	7	6	1	5	8	0	2	5	2	2	3	3	1.253	0.872	–	–	I
K	4	6	5	9	4	2	5	5	2	6	3	3	3	13	1	2	2	1	2	3	4	2	6	0	3	8	6	2	1	2	0	5	3	5	1	1	3	5	6	4	1	6	3	3	8	10	3	4	3	3	2	1.920	1.303	–	–	K
L	7	7	4	6	6	7	2	7	4	7	5	5	5	5	7	5	10	8	14	9	11	11	8	12	7	8	10	4	4	4	7	3	7	10	4	1	8	3	4	5	6	7	6	2	2	5	12	7	5	5	4	1.590	0.707	–	–	L
M	1	0	0	1	4	3	4	2	1	4	1	1	3	2	0	0	1	1	0	1	2	0	1	1	2	1	3	0	1	3	5	1	0	5	4	0	2	1	1	2	2	1	1	2	0	2	2	2	0	1	0	–	–	–	–	M
N	4	3	0	3	2	4	1	1	1	4	2	5	1	2	2	5	2	12	6	2	1	1	2	2	4	3	3	1	2	3	2	1	0	4	3	3	4	1	1	2	3	4	2	1	7	5	2	2	3	8	0	–	–	–	–	N
P	5	9	7	6	5	8	11	9	10	13	12	10	8	4	4	3	2	3	1	5	8	2	7	4	3	3	4	6	5	1	7	3	3	7	4	5	5	4	2	2	4	3	0	2	5	4	8	1	1	2	7	0.714	0.440	6.99E-03	n.s.	P
Q	6	1	5	2	4	3	1	1	2	5	2	0	3	6	0	3	1	3	3	1	4	0	4	3	8	1	6	0	1	2	3	2	4	1	0	5	2	5	0	1	2	0	1	2	2	0	1	3	0	1	6	0.387	0.325	5.55E-08	✻	Q
R	1	11	9	5	2	4	3	2	7	2	4	0	2	3	5	4	5	6	2	2	2	3	5	2	1	2	3	0	6	5	5	3	4	5	0	0	5	0	4	2	6	6	2	6	4	0	2	5	1	1	7	0.483	0.340	5.65E-08	✻	R
S	4	5	0	9	9	4	11	4	4	4	7	8	5	9	8	4	5	4	2	6	5	8	5	4	14	12	8	8	8	4	5	10	8	6	7	5	4	6	10	6	2	4	5	7	5	8	6	5	5	5	9	0.682	0.294	5.57E-05	✻	S
T	5	6	3	13	7	4	6	5	6	6	1	3	2	1	13	5	5	7	2	6	8	4	4	2	2	8	5	5	8	1	2	8	8	4	5	11	2	4	9	5	2	5	2	3	2	6	3	5	10	12	6	0.870	0.511	7.90E-01	n.s.	T
V	4	7	2	4	5	11	5	5	6	5	5	6	14	7	6	1	5	9	6	5	5	4	5	4	3	3	4	4	2	2	6	2	6	14	2	12	4	2	2	7	0	6	8	5	4	1	3	2	2	5	4	1.235	0.757	–	–	V
W	1	0	2	2	2	0	0	0	0	0	0	0	3	1	2	0	0	3	4	1	1	0	5	1	0	0	0	0	0	0	1	1	0	0	0	1	1	1	0	2	0	0	0	0	0	2	0	2	0	1	3	0.267	0.387	5.55E-08	✻	W
Y	5	7	3	2	6	1	1	4	3	1	2	1	3	1	0	0	2	0	1	2	2	0	1	3	1	0	0	2	1	3	2	0	0	2	4	3	1	4	0	2	1	0	3	1	0	2	2	0	2	1	6	0.293	0.268	5.55E-08	✻	Y
total	77	97	72	100	92	97	102	78	78	90	95	72	89	104	99	70	82	91	84	86	85	73	76	82	64	85	83	65	70	65	68	55	65	93	58	85	72	56	69	59	56	59	60	69	61	69	68	67	53	66	83	0.918	0.170			total

Additional file 5. Continued.

FRUIT FLY x-x-S-x-I

position -4

	C50	C49	C48	C47	C46	C45	C44	C43	C42	C41	C40	C39	C38	C37	C36	C35	C34	C33	C32	C31	C30	C29	C28	C27	C26	C25	C24	C23	C22	C21	C20	C19	C18	C17	C16	C15	C14	C13	C12	C11	C10	C9	C8	C7	C6	C5	C4	C3	C2	C1	C0	C-index	SD	*P-value*	mark	
A	8	0	7	4	5	3	3	5	3	5	3	5	5	7	1	5	1	2	1	3	2	0	2	1	3	5	3	2	4	3	2	3	6	4	3	5	2	4	3	5	3	1	5	4	5	5	6	1	1	2	2	1.710	0.937	–	–	A
C	0	1	0	1	2	1	1	0	0	2	1	1	3	1	1	0	2	1	0	2	2	0	0	2	2	1	1	0	0	1	1	0	0	1	1	2	1	0	2	1	3	0	0	2	2	3	1	0	0	0	0	–	–	–	–	C
D	2	4	4	6	3	2	4	3	5	2	3	4	2	3	4	2	5	4	1	4	5	2	4	3	1	0	0	4	4	2	2	2	1	3	3	5	1	4	4	3	1	2	4	5	0	4	3	5	2	2	3	0.987	0.490	–	–	D
E	4	1	5	3	1	1	3	3	2	0	3	1	2	3	1	1	1	3	3	4	2	2	1	5	1	2	3	0	3	3	5	3	2	2	4	8	2	0	2	2	3	1	2	1	4	4	0	5	1	3	5	0.484	0.318	6.59E-05	✻	E
F	1	3	2	0	0	4	0	2	4	3	2	3	1	3	0	0	1	1	3	4	2	0	1	3	2	0	3	2	3	1	3	2	2	0	4	3	2	6	1	2	3	4	2	1	4	4	5	1	1	2	3	0.707	0.489	–	–	F
G	4	1	1	2	2	2	2	1	2	2	6	5	1	2	8	4	1	6	9	4	3	4	2	3	2	0	3	4	5	1	2	3	1	7	4	3	2	4	1	6	1	2	5	4	4	2	2	2	4	2	3	1.020	0.651	–	–	G
H	0	0	1	1	3	1	1	2	2	0	0	4	4	1	1	2	0	1	0	2	0	1	1	1	1	2	0	1	2	1	0	0	2	0	1	1	1	1	2	2	2	1	6	1	0	1	0	1	1	0	1	1.180	1.190	–	–	H
I	2	3	3	2	1	0	2	4	3	4	2	1	3	1	4	3	0	2	4	1	1	2	6	3	5	2	0	4	5	5	0	2	2	3	3	2	2	0	3	1	2	5	3	5	8	6	5	1	2	2	3	0.900	0.592	–	–	I
K	4	2	2	6	2	2	1	0	3	4	1	1	1	2	29	3	5	2	1	2	1	4	4	6	3	2	3	7	2	3	4	8	1	1	1	2	4	1	2	3	2	3	4	2	3	2	3	7	4	2	6	0.557	0.682	8.46E-06	✻	K
L	9	9	6	7	3	5	4	4	2	5	10	7	9	3	6	8	3	4	4	6	1	3	5	5	4	8	4	2	6	3	5	3	8	6	15	0	4	3	6	3	2	6	4	4	3	2	4	7	10	3	12	0.422	0.229	6.12E-08	✻	L
M	0	0	1	1	0	1	0	0	1	1	1	1	1	1	1	0	0	1	0	2	1	2	1	0	0	5	0	1	2	1	1	0	0	0	1	2	1	0	0	1	0	0	2	0	3	2	0	0	1	0	3	0.267	0.323	5.55E-08	✻	M
N	1	2	1	1	1	3	4	2	2	4	3	2	1	3	0	4	6	1	4	2	2	1	2	5	2	5	2	1	3	4	4	1	2	2	3	5	0	4	2	3	4	4	5	1	2	3	4	4	4	3	2	1.340	0.724	–	–	N
P	3	5	0	2	0	4	2	3	5	3	3	5	2	0	2	4	0	4	5	4	2	2	4	1	2	5	3	2	0	3	3	4	4	5	1	3	4	4	2	5	3	0	1	1	4	2	1	4	2	2	1	2.700	1.568	–	–	P
Q	0	2	1	1	0	2	1	1	3	1	5	2	2	2	1	0	0	0	1	0	2	3	2	0	1	2	3	2	2	4	1	2	1	0	2	1	3	1	3	1	5	2	0	1	1	3	5	2	1	0	5	0.324	0.265	5.56E-08	✻	Q
R	6	1	1	1	3	1	2	4	2	5	0	1	4	1	2	5	3	3	4	2	3	2	2	1	3	5	4	2	3	4	6	2	1	4	2	3	1	3	6	3	5	4	7	2	1	4	1	6	7	5	5	0.612	0.360	3.79E-01	n.s.	R
S	6	5	4	4	4	3	5	4	8	4	10	11	4	5	6	5	4	5	1	7	4	4	3	8	3	10	3	8	6	8	6	5	4	10	7	7	6	3	5	5	5	3	2	11	7	6	5	10	4	11	11	0.516	0.226	2.84E-05	✻	S
T	1	6	1	4	3	2	5	3	5	0	8	2	7	2	1	6	2	2	3	3	1	4	3	2	2	3	5	5	2	0	3	0	3	3	4	4	3	4	5	4	8	3	3	3	2	2	4	5	2	6	3	1.093	0.628	–	–	T
V	4	3	3	4	6	1	2	2	2	6	1	5	3	3	1	1	1	2	0	5	2	6	6	2	5	4	5	3	4	2	4	7	3	3	5	2	2	3	2	3	7	6	5	2	7	2	3	3	4	2	2	1.690	0.897	–	–	V
W	1	0	0	0	1	0	1	0	0	2	1	0	1	0	0	1	0	0	2	0	1	0	1	2	0	1	0	0	2	0	0	0	1	1	0	0	0	1	0	3	0	2	1	1	0	0	0	0	0	1	1	0.560	0.760	8.36E-01	n.s.	W
Y	2	1	2	3	1	1	6	1	2	3	0	2	2	0	4	0	1	2	3	0	1	3	2	1	5	1	2	3	1	2	2	1	0	2	0	4	1	1	3	3	3	1	2	1	2	2	1	2	1	1	5	0.360	0.256	5.56E-08	✻	Y
total	58	49	45	53	41	39	49	44	56	56	63	63	58	43	73	54	36	46	49	57	38	45	52	54	47	63	47	53	59	51	54	48	44	57	64	62	42	47	54	59	62	50	63	52	62	59	53	66	52	49	76	0.695	0.106			total

position -3

	C50	C49	C48	C47	C46	C45	C44	C43	C42	C41	C40	C39	C38	C37	C36	C35	C34	C33	C32	C31	C30	C29	C28	C27	C26	C25	C24	C23	C22	C21	C20	C19	C18	C17	C16	C15	C14	C13	C12	C11	C10	C9	C8	C7	C6	C5	C4	C3	C2	C1	C0	C-index	SD	*P-value*	mark	
A	7	5	1	4	3	2	8	1	6	6	4	7	4	2	3	0	2	4	1	4	2	2	6	4	3	3	0	3	1	3	8	5	1	9	4	6	2	5	4	7	3	2	4	5	5	6	2	3	4	3	5	0.756	0.428	–	–	A
C	2	2	2	3	1	0	1	2	4	0	0	4	5	1	0	2	1	1	0	5	1	3	1	2	0	3	0	2	0	0	1	0	1	1	0	3	0	1	1	1	0	1	0	1	1	1	1	2	2	4	1	1.400	1.355	–	–	C
D	3	1	2	2	1	3	1	3	2	6	4	7	1	1	1	2	2	2	3	2	2	4	0	1	4	3	4	1	2	6	0	1	3	0	2	3	2	2	4	2	3	4	3	3	1	3	3	2	1	0	3	0.787	0.507	–	–	D
E	0	2	1	4	6	5	4	2	3	2	4	1	0	2	3	4	2	3	3	1	3	4	3	2	2	5	5	1	3	4	2	8	3	2	1	2	3	0	3	2	6	2	1	5	2	4	4	4	3	2	6	0.477	0.274	1.33E-05	✻	E
F	1	3	2	0	2	1	1	0	1	3	2	1	4	4	7	4	2	1	2	2	1	1	1	4	0	3	2	1	2	2	3	2	2	1	4	6	3	4	0	1	5	4	1	2	4	1	3	0	1	3	2	1.100	0.789	–	–	F
G	4	3	5	4	1	3	2	2	5	5	3	2	3	1	6	5	3	4	6	3	3	3	5	0	4	7	3	4	4	5	1	3	3	3	6	2	4	1	1	2	2	2	8	4	5	5	2	3	2	4	1	3.420	1.679	–	–	G
H	1	1	1	2	1	0	0	1	0	2	2	0	3	1	0	4	1	2	2	3	0	0	1	1	1	2	2	4	0	1	0	0	0	1	0	2	0	0	1	0	2	1	1	3	3	1	0	2	3	1	2	0.600	0.562	1.05E-01	n.s.	H
I	3	3	2	1	2	3	2	1	3	4	2	6	1	1	3	3	4	4	2	5	3	5	2	5	5	4	4	2	5	2	2	4	1	4	3	1	1	3	4	2	2	3	2	5	2	4	2	4	7	3	7	0.431	0.205	6.14E-08	✻	I
K	0	4	2	3	3	3	2	2	3	3	1	2	4	5	2	5	1	1	2	0	0	4	0	1	1	3	0	6	7	3	2	2	0	3	7	4	2	6	5	5	5	5	7	1	2	6	3	6	0	5	11	0.271	0.190	5.55E-08	✻	K
L	6	3	2	6	5	4	6	2	8	2	4	5	6	5	6	3	4	6	6	4	4	2	2	14	4	3	6	4	5	4	8	6	4	4	4	6	6	3	4	7	4	3	8	8	4	6	6	7	7	1	8	0.618	0.275	2.03E-01	n.s.	L
M	2	2	0	0	1	0	2	3	0	0	4	1	5	1	2	1	2	0	3	0	2	2	3	0	3	2	2	2	1	3	2	1	1	2	0	1	0	0	1	2	2	0	2	1	1	0	0	3	0	2	1	1.400	1.212	–	–	M
N	1	2	4	1	3	1	1	2	7	1	2	5	1	1	2	4	1	2	3	2	2	2	3	2	6	3	2	4	2	4	2	1	3	2	3	3	2	3	4	1	4	3	4	2	3	4	1	4	4	4	4	0.665	0.345	8.86E-01	n.s.	N
P	5	3	1	1	1	2	4	4	0	2	5	2	3	4	4	2	1	0	0	4	2	3	3	2	3	3	1	1	3	2	1	2	3	1	0	2	2	2	3	4	2	0	3	1	3	2	1	6	2	2	3	0.753	0.466	–	–	P
Q	5	0	3	1	0	0	0	1	2	2	1	0	0	2	0	3	0	3	2	0	2	0	6	3	1	2	2	1	5	3	2	1	1	1	4	2	4	4	0	5	1	3	1	1	5	0	2	3	2	1	1	1.860	1.629	–	–	Q
R	6	2	6	8	1	1	5	4	2	4	6	6	4	3	25	0	1	4	3	5	1	2	0	3	3	3	2	4	2	3	3	3	4	4	3	4	2	3	3	8	3	2	4	2	5	7	7	1	4	4	4	0.975	0.897	–	–	R
S	3	3	3	3	2	4	1	6	5	6	10	5	5	3	7	4	6	5	4	6	3	5	4	5	4	5	6	8	7	4	4	1	8	11	8	5	2	3	5	2	8	7	6	2	4	2	4	7	3	3	6	0.790	0.367	–	–	S
T	0	4	4	4	3	3	4	3	0	4	5	2	2	2	0	3	0	1	1	5	5	2	5	0	0	3	3	1	3	0	2	1	3	5	7	3	4	5	5	3	3	1	0	2	5	4	2	3	2	4	5	0.544	0.352	1.37E-01	n.s.	T
V	6	4	3	3	4	2	3	5	2	3	3	6	6	4	1	4	1	2	0	3	0	0	4	4	2	4	2	2	3	1	7	4	1	2	4	7	2	2	3	2	2	4	4	3	4	2	6	5	2	3	6	0.520	0.285	5.40E-04	n.s.	V
W	1	0	1	1	0	0	1	0	2	0	0	1	0	0	1	0	0	0	2	1	1	1	0	0	1	1	0	1	1	1	0	1	0	1	0	0	1	0	2	0	1	1	3	0	0	0	2	0	0	0	0	–	–	–	–	W
Y	2	2	0	2	1	2	1	0	1	1	1	0	1	0	0	1	2	1	4	2	1	0	3	1	0	1	1	1	3	0	4	2	2	0	4	0	0	0	1	3	4	2	1	1	3	1	2	1	3	0	0	–	–	–	–	Y
total	58	49	45	53	41	39	49	44	56	56	63	63	58	43	73	54	36	46	49	57	38	45	52	54	47	63	47	53	59	51	54	48	44	57	64	62	42	47	54	59	62	50	63	52	62	59	53	66	52	49	76	0.695	0.106			total

position -1

	C50	C49	C48	C47	C46	C45	C44	C43	C42	C41	C40	C39	C38	C37	C36	C35	C34	C33	C32	C31	C30	C29	C28	C27	C26	C25	C24	C23	C22	C21	C20	C19	C18	C17	C16	C15	C14	C13	C12	C11	C10	C9	C8	C7	C6	C5	C4	C3	C2	C1	C0	C-index	SD	*P-value*	mark	
A	7	1	3	3	5	2	2	2	7	3	5	5	7	1	6	3	3	2	5	4	3	4	2	3	0	2	1	2	7	5	5	2	5	4	3	6	3	3	2	1	4	1	3	4	4	3	9	8	1	5	6	0.603	0.340	1.98E-01	n.s.	A
C	3	1	0	0	0	3	2	2	3	1	2	2	1	2	1	3	0	3	1	2	0	0	0	0	0	1	1	0	2	0	1	2	0	0	0	1	0	0	0	2	1	1	2	0	2	0	1	0	2	1	1	1.040	1.029	–	–	C
D	2	2	1	4	1	2	3	2	7	1	4	10	2	5	2	2	2	0	2	4	3	1	4	1	2	5	2	3	3	0	4	4	1	4	5	6	4	2	4	4	1	4	4	5	5	2	3	4	4	3	3	1.033	0.614	–	–	D
E	3	5	2	1	2	1	2	3	1	4	6	6	4	2	0	3	3	1	3	5	3	3	4	5	5	2	3	1	2	3	3	2	2	1	4	4	3	4	2	2	2	0	8	1	3	7	2	4	3	2	0	–	–	–	–	E
F	1	0	3	1	1	1	2	3	4	1	4	0	3	1	1	0	1	1	1	1	0	5	2	6	2	2	0	3	1	3	1	0	2	4	1	0	0	3	2	2	5	3	2	2	6	0	4	3	1	3	2	0.980	0.802	–	–	F
G	5	1	3	2	1	5	3	3	2	5	5	6	2	4	7	6	3	8	4	5	4	2	5	2	7	3	6	3	2	2	3	6	0	5	2	2	4	3	3	2	5	1	4	2	2	5	2	4	2	2	6	0.583	0.300	1.76E-01	n.s.	G
H	1	3	1	3	1	0	0	1	1	1	2	2	1	1	2	1	0	1	3	3	0	2	1	2	0	1	2	2	1	5	4	1	1	2	0	2	0	2	1	3	2	2	2	2	3	0	3	0	3	0	2	0.770	0.582	–	–	H
I	1	3	3	3	4	3	3	1	2	3	1	3	4	4	2	1	3	2	1	4	3	3	3	2	3	1	2	4	3	2	1	1	1	2	2	4	2	3	4	6	4	4	1	2	1	3	4	2	3	2	8	0.323	0.145	5.55E-08	✻	I
K	3	7	1	2	4	0	1	5	5	4	3	4	3	3	5	3	2	2	2	3	1	2	6	8	4	5	3	3	5	2	4	2	3	1	4	3	3	4	6	5	3	6	4	0	1	5	2	4	1	4	3	1.107	0.585	–	–	K
L	2	2	5	3	3	3	5	5	3	2	6	5	6	1	2	8	2	3	5	6	3	4	5	5	5	3	6	5	8	4	3	4	7	7	9	2	3	1	2	5	3	3	3	3	4	0	4	4	5	4	7	0.574	0.271	4.23E-02	n.s.	L
M	2	0	1	1	1	1	1	0	0	0	0	0	2	1	0	0	1	1	2	2	1	1	0	2	0	2	2	1	2	3	1	2	2	2	3	1	2	0	1	2	0	1	2	0	0	1	2	2	0	0	1	1.080	0.900	–	–	M
N	1	3	3	6	3	3	5	2	1	4	4	0	1	3	0	1	3	2	1	2	0	3	1	1	3	5	0	3	4	4	6	0	1	5	3	4	3	3	1	4	3	5	1	6	1	5	2	5	4	3	7	0.391	0.248	7.68E-08	✻	N
P	2	3	2	3	0	2	4	1	3	6	3	4	5	2	6	6	1	3	8	4	1	2	1	2	2	2	2	2	4	0	3	3	3	3	3	4	0	1	0	0	3	0	2	4	6	4	2	2	1	2	4	0.660	0.448	8.11E-01	n.s.	P
Q	1	1	2	2	3	4	7	1	1	2	0	3	2	0	3	2	2	0	0	2	1	0	1	1	2	1	4	1	1	2	2	4	1	5	1	3	2	4	2	5	1	1	1	3	6	2	5	4	3	0	2	1.070	0.821	–	–	Q
R	8	1	3	2	4	2	2	0	5	2	1	3	2	2	3	3	2	3	1	1	5	1	5	7	0	7	3	5	2	2	2	2	1	1	2	4	2	0	2	0	4	3	1	1	3	6	0	3	5	2	8	0.328	0.238	5.56E-08	✻	R
S	5	1	4	6	4	3	1	1	4	7	5	3	2	2	7	5	4	6	2	1	5	8	2	3	4	10	3	6	5	7	3	4	4	4	8	5	4	6	5	3	5	3	9	5	2	3	3	7	3	7	8	0.548	0.266	3.94E-03	n.s.	S
T	7	5	3	4	2	2	2	2	3	6	6	2	4	3	23	3	1	4	2	1	2	2	3	1	3	4	2	3	3	5	3	7	3	2	6	7	2	2	8	3	10	3	3	4	4	4	4	5	6	4	0	–	–	–	–	T
V	1	5	2	4	1	0	2	5	3	2	4	3	2	4	1	2	1	2	3	6	3	1	2	2	5	5	4	3	3	1	3	1	4	1	3	3	4	2	3	3	3	5	7	2	5	5	1	3	2	2	4	0.720	0.383	–	–	V
W	1	1	1	0	0	0	1	0	1	1	0	1	0	0	0	1	2	1	1	0	0	0	0	0	0	0	1	0	0	1	0	1	1	2	0	0	1	1	2	1	1	1	2	3	2	1	0	1	0	2	1	0.720	0.757	–	–	W
Y	2	4	2	3	1	2	1	5	0	1	2	1	5	2	2	1	0	1	2	1	0	1	5	1	0	1	1	3	0	1	1	1	2	3	3	1	1	3	5	5	2	3	2	3	2	3	0	1	3	1	3	0.640	0.475	4.12E-01	n.s.	Y
total	58	49	45	53	41	39	49	44	56	56	63	63	58	43	73	54	36	46	49	57	38	45	52	54	47	63	47	53	59	51	54	48	44	57	64	62	42	47	54	59	62	50	63	52	62	59	53	66	52	49	76	0.695	0.106			total

Additional file 5. Continued.

FRUIT FLY x-x-S-x-L

position -4

	C50	C49	C48	C47	C46	C45	C44	C43	C42	C41	C40	C39	C38	C37	C36	C35	C34	C33	C32	C31	C30	C29	C28	C27	C26	C25	C24	C23	C22	C21	C20	C19	C18	C17	C16	C15	C14	C13	C12	C11	C10	C9	C8	C7	C6	C5	C4	C3	C2	C1	C0	C-index	SD	*P-value*	mark	
A	5	7	4	7	6	8	14	5	3	6	6	3	9	9	8	4	7	7	7	7	8	5	5	11	5	7	5	4	4	8	4	3	11	8	9	6	8	6	9	5	7	6	11	5	3	4	9	8	4	9	10	0.658	0.241	5.70E-08	✻	A
C	1	0	3	1	2	1	6	4	3	5	1	2	1	1	3	0	0	0	1	2	0	2	1	5	1	1	1	1	4	2	2	1	1	4	1	1	4	3	2	3	1	2	1	3	4	2	2	1	2	2	4	0.485	0.355	5.74E-08	✻	C
D	3	5	1	6	3	7	5	10	4	5	10	7	7	4	8	5	3	5	2	5	4	4	5	6	4	4	2	1	1	9	5	3	3	7	5	10	2	6	2	1	5	3	6	2	0	2	3	5	4	2	5	0.884	0.487	7.54E-01	n.s.	D
E	5	7	9	4	3	29	5	4	4	8	4	5	6	5	2	5	7	4	5	4	5	5	6	6	1	4	7	8	5	5	10	5	6	5	9	2	4	6	4	4	4	10	1	3	0	7	3	6	8	6	4	1.400	1.003	–	–	E
F	3	7	7	8	1	1	7	2	4	1	0	3	4	5	3	2	5	4	1	1	0	6	3	5	2	6	2	2	5	2	2	4	6	8	8	2	3	3	6	12	5	3	2	3	4	4	1	5	4	7	3	1.293	0.824	–	–	F
G	5	4	6	2	6	7	4	6	5	5	9	3	3	5	6	6	9	5	4	7	6	12	5	5	6	11	6	3	10	2	8	9	11	7	6	3	2	6	3	6	4	9	3	4	4	5	7	2	11	4	7	0.820	0.366	9.93E-03	n.s.	G
H	1	4	2	2	1	4	1	3	2	1	0	2	2	2	1	1	0	3	2	3	3	6	3	7	2	2	2	3	1	0	3	5	2	2	2	2	6	3	1	3	1	4	3	2	1	2	1	0	3	2	3	0.760	0.504	4.36E-03	n.s.	H
I	8	2	2	5	6	1	5	5	9	7	9	6	3	7	5	5	5	4	7	10	4	7	8	9	2	2	3	2	8	1	4	1	8	3	7	9	4	12	3	9	8	6	7	11	11	7	4	6	7	2	3	1.907	0.957	–	–	I
K	9	7	5	5	4	3	4	5	3	9	2	3	5	6	8	5	5	6	4	3	4	8	1	7	6	5	5	7	9	5	7	4	6	10	4	3	4	6	8	10	1	7	3	8	5	6	5	6	6	9	7	0.789	0.314	9.47E-03	n.s.	K
L	9	13	16	12	9	11	10	15	8	17	15	16	12	9	7	12	7	8	7	4	5	11	9	7	13	11	8	16	2	13	9	4	13	10	12	11	12	12	3	11	14	12	9	7	17	10	22	7	7	17	12	0.885	0.338	8.21E-01	n.s.	L
M	1	2	5	0	2	6	6	4	3	0	1	4	3	0	1	4	3	4	1	2	0	2	1	1	1	1	4	0	3	1	4	0	1	2	3	0	2	2	1	1	1	1	4	2	1	5	6	5	3	1	2	1.110	0.877	–	–	M
N	3	7	5	6	4	4	6	5	5	5	10	4	8	6	6	3	8	5	4	3	3	5	4	6	1	3	2	6	2	6	6	5	5	2	1	5	2	6	10	2	7	5	4	1	4	1	2	6	6	8	6	0.777	0.365	1.50E-02	n.s.	N
P	6	6	6	8	3	3	1	4	3	2	3	3	3	4	4	6	5	4	6	4	8	9	5	5	4	5	4	2	2	6	4	8	9	3	4	4	1	9	3	8	5	4	2	8	5	5	6	11	6	7	4	1.230	0.567	–	–	P
Q	2	3	2	2	2	3	1	2	6	5	3	4	4	2	6	3	3	7	5	2	5	2	1	7	4	7	4	4	3	5	5	4	2	3	6	5	3	0	1	2	6	6	3	3	4	1	4	4	7	4	3	1.213	0.598	–	–	Q
R	4	4	5	4	3	3	8	12	6	0	4	8	4	4	4	2	4	5	11	6	6	7	6	5	9	6	6	8	6	3	5	6	5	4	5	5	3	7	11	5	6	5	4	4	3	6	4	6	9	7	9	0.607	0.255	5.58E-08	✻	R
S	3	9	8	10	5	8	11	8	14	9	8	5	8	9	4	12	5	9	16	13	12	7	4	5	10	13	13	5	12	5	8	13	10	5	11	7	7	10	6	11	9	5	10	6	10	9	15	11	13	4	9	0.978	0.359	–	–	S
T	5	5	8	6	12	6	13	2	4	6	2	10	9	3	9	4	4	5	3	10	7	6	7	5	5	6	3	6	4	8	5	4	5	6	10	7	3	3	6	4	3	5	1	5	6	2	1	5	6	5	4	1.375	0.655	–	–	T
V	8	7	6	9	9	8	2	3	2	8	9	7	7	6	4	7	6	8	5	4	6	5	5	3	6	4	9	9	4	7	3	9	8	8	6	7	7	10	7	12	12	4	8	6	6	4	4	8	6	5	6	1.077	0.386	–	–	V
W	0	1	1	0	1	1	2	0	0	2	2	0	0	1	0	1	2	1	0	1	1	0	0	1	0	1	0	1	0	2	2	1	0	0	1	0	0	1	0	1	0	1	0	3	1	3	5	1	0	1	0	–	–	–	–	W
Y	6	4	3	3	2	2	3	3	2	2	3	1	4	1	4	3	1	5	1	6	1	2	6	3	2	4	2	3	3	0	3	5	7	8	5	4	3	2	1	1	1	1	3	3	2	2	1	2	3	5	2	1.470	0.871	–	–	Y
total	87	104	104	100	84	116	114	102	90	103	101	96	102	89	93	90	89	99	92	97	88	111	85	109	84	103	88	91	88	90	99	94	119	105	115	93	80	113	87	111	100	99	85	89	91	87	105	105	115	107	103	0.949	0.098			total

position -3

	C50	C49	C48	C47	C46	C45	C44	C43	C42	C41	C40	C39	C38	C37	C36	C35	C34	C33	C32	C31	C30	C29	C28	C27	C26	C25	C24	C23	C22	C21	C20	C19	C18	C17	C16	C15	C14	C13	C12	C11	C10	C9	C8	C7	C6	C5	C4	C3	C2	C1	C0	C-index	SD	*P-value*	mark	
A	7	5	6	4	4	28	9	5	11	2	3	6	6	7	4	13	7	3	2	9	7	9	5	7	5	9	8	5	6	8	5	9	4	5	11	6	4	10	8	4	8	4	6	3	5	8	9	7	8	5	4	1.695	0.977	–	–	A
C	3	7	4	1	1	1	1	0	1	1	2	3	3	5	3	3	2	0	6	1	1	0	3	4	2	2	3	5	2	2	5	0	2	4	2	1	2	2	2	2	0	3	2	2	2	1	1	1	5	1	4	0.560	0.403	1.27E-07	✻	C
D	6	4	9	7	3	9	7	5	5	7	6	4	5	3	6	4	3	4	6	0	1	7	2	3	2	3	3	1	2	5	1	7	8	6	4	7	5	4	3	5	5	2	4	3	5	6	5	7	4	1	7	0.640	0.307	6.71E-07	✻	D
E	4	4	3	4	7	7	6	3	2	2	5	4	4	6	2	6	7	5	6	4	2	4	6	5	5	7	7	6	4	4	3	4	6	7	6	4	6	5	7	2	2	2	3	6	6	7	1	3	14	5	5	0.960	0.435	–	–	E
F	3	4	4	8	4	6	3	3	1	4	6	2	4	5	5	1	2	3	2	8	1	3	3	6	4	6	3	3	4	3	3	2	4	7	2	5	3	7	5	13	5	5	1	4	7	2	2	5	4	6	1	4.120	2.219	–	–	F
G	6	5	11	8	4	6	5	10	6	7	5	5	7	4	6	8	8	9	4	6	5	15	2	7	9	5	3	5	7	4	5	6	9	4	11	2	4	3	3	5	5	7	5	8	4	6	6	3	2	6	3	1.973	0.849	–	–	G
H	1	2	2	4	2	1	0	2	4	0	5	1	2	3	0	1	2	2	1	3	3	3	2	6	2	4	1	4	3	6	2	1	3	1	0	3	3	4	1	2	2	4	1	5	3	3	6	1	7	4	5	0.512	0.338	5.57E-08	✻	H
I	8	8	4	6	2	3	14	1	7	6	10	7	3	9	5	4	1	8	7	5	6	7	3	4	7	6	3	5	6	8	10	6	12	2	5	3	5	10	4	7	10	3	3	1	5	3	4	7	5	5	6	0.943	0.475	9.99E-01	n.s.	I
K	6	2	1	6	3	5	6	5	5	7	2	4	8	0	4	1	7	7	2	6	7	3	7	10	4	8	6	5	2	2	3	4	9	6	13	5	6	7	7	0	5	4	5	7	4	5	4	6	5	9	10	0.510	0.257	5.55E-08	✻	K
L	8	8	9	6	5	16	11	16	13	9	7	8	10	7	12	12	10	9	8	7	9	11	3	11	9	14	10	10	11	9	11	8	9	8	13	13	3	15	5	17	11	13	6	7	10	10	4	13	12	8	4	2.420	0.804	–	–	L
M	2	3	1	2	3	0	0	3	1	2	3	1	2	3	1	2	2	1	2	1	2	3	4	2	3	0	2	2	1	1	0	2	3	3	3	0	0	1	3	3	2	1	3	0	2	1	1	3	2	0	1	1.760	1.098	–	–	M
N	6	4	8	5	1	5	3	2	6	9	3	5	5	3	5	3	10	10	10	5	10	4	4	6	4	3	4	3	7	4	3	5	1	4	4	3	2	1	3	5	7	7	5	5	3	3	3	3	3	7	8	0.585	0.293	6.31E-08	✻	N
P	0	4	2	6	7	0	6	9	3	6	6	5	3	8	5	1	4	6	6	4	4	5	4	5	4	4	6	3	5	8	4	1	4	8	4	3	9	2	6	5	3	5	5	6	3	1	9	6	6	11	0	–	–	–	–	P
Q	4	9	5	6	5	1	6	5	2	5	4	7	3	3	7	4	6	1	1	6	4	5	3	3	2	6	6	1	3	3	5	4	5	7	9	2	2	6	3	4	3	5	5	2	8	7	4	6	4	6	6	0.743	0.337	2.25E-03	n.s.	Q
R	4	9	9	8	4	5	11	11	3	8	6	10	9	3	0	2	5	3	6	5	5	10	10	3	4	5	3	7	8	3	4	4	14	8	4	12	5	12	6	7	7	2	4	6	4	9	6	4	11	5	16	0.391	0.195	5.55E-08	✻	R
S	8	6	9	11	11	5	10	2	10	9	12	9	5	5	9	8	2	7	9	10	6	4	7	10	7	13	8	4	6	9	17	10	11	13	8	9	8	14	4	13	12	15	9	8	10	6	10	12	9	11	9	0.978	0.354	–	–	S
T	6	8	5	2	10	5	9	11	6	11	6	4	8	4	8	5	3	10	4	4	4	7	3	5	6	4	3	8	2	2	5	6	11	6	10	5	5	5	5	10	5	6	4	3	3	5	21	4	3	7	7	0.863	0.472	2.01E-02	n.s.	T
V	4	8	6	5	2	4	3	4	3	4	4	6	6	6	7	8	5	7	6	9	7	2	5	7	4	1	6	8	4	7	10	6	2	3	5	6	5	2	8	1	7	5	4	5	3	1	7	9	6	4	7	0.734	0.315	1.39E-03	n.s.	V
W	1	0	2	0	3	3	0	3	0	2	1	2	2	3	1	0	1	2	0	2	1	2	5	2	0	0	1	2	1	0	1	3	0	0	0	0	0	2	1	3	0	1	2	3	0	2	1	1	1	2	0	–	–	–	–	W
Y	0	4	4	1	3	6	4	2	1	2	5	3	7	2	3	4	2	2	4	2	3	7	4	3	1	3	2	4	4	2	2	6	2	3	1	4	3	1	3	3	1	5	8	5	4	1	1	4	4	4	0	–	–	–	–	Y
total	87	104	104	100	84	116	114	102	90	103	101	96	102	89	93	90	89	99	92	97	88	111	85	109	84	103	88	91	88	90	99	94	119	105	115	93	80	113	87	111	100	99	85	89	91	87	105	105	115	107	103	0.949	0.098			total

position -1

	C50	C49	C48	C47	C46	C45	C44	C43	C42	C41	C40	C39	C38	C37	C36	C35	C34	C33	C32	C31	C30	C29	C28	C27	C26	C25	C24	C23	C22	C21	C20	C19	C18	C17	C16	C15	C14	C13	C12	C11	C10	C9	C8	C7	C6	C5	C4	C3	C2	C1	C0	C-index	SD	*P-value*	mark	
A	8	10	10	9	8	5	12	3	12	7	6	6	5	8	8	8	3	8	6	8	8	6	5	8	6	3	7	7	7	4	11	4	11	9	11	3	5	6	5	6	5	3	7	8	6	6	5	5	8	5	8	0.850	0.297	1.73E-01	n.s.	A
C	2	3	9	3	2	4	3	5	2	1	4	2	1	2	3	1	1	5	0	2	1	2	2	1	1	5	2	0	3	3	2	2	3	4	5	1	3	2	2	2	3	1	2	2	1	0	0	2	3	1	0	–	–	–	–	C
D	3	6	2	5	3	7	6	3	5	6	4	6	2	1	4	5	0	2	4	3	5	7	10	6	2	7	5	6	1	2	7	7	10	5	8	3	3	5	6	2	6	5	6	7	4	5	0	5	3	12	3	1.580	0.832	–	–	D
E	5	2	4	7	3	6	10	9	5	6	0	4	4	3	6	4	4	8	8	5	6	6	2	5	2	4	3	4	5	6	5	10	11	4	7	6	6	7	1	4	5	4	8	5	6	7	5	9	6	4	4	1.330	0.572	–	–	E
F	2	3	4	6	1	4	4	5	11	7	4	4	4	6	5	2	1	4	7	2	4	3	3	2	1	4	3	3	3	3	6	4	2	3	4	4	1	13	4	5	3	3	4	4	4	0	6	5	7	4	5	0.804	0.460	1.31E-04	n.s.	F
G	2	4	8	5	7	5	9	3	5	7	8	5	5	3	6	5	4	6	2	8	6	6	7	11	9	8	1	2	4	3	3	6	5	6	3	3	0	3	2	7	8	4	2	4	5	5	8	4	8	4	2	2.540	1.182	–	–	G
H	2	0	2	2	2	2	3	4	1	4	3	2	2	5	2	1	3	1	3	4	2	6	3	5	3	0	3	0	2	4	1	4	1	2	1	5	3	6	0	3	2	2	2	2	1	0	3	2	4	3	2	1.230	0.751	–	–	H
I	5	10	1	5	7	2	4	5	5	1	3	3	8	2	2	3	10	8	3	4	1	12	5	5	5	5	3	6	4	5	10	5	7	5	10	4	10	7	8	2	6	4	7	4	5	4	7	4	5	2	5	1.032	0.529	–	–	I
K	5	6	8	3	7	2	7	5	6	4	6	8	8	4	4	6	4	5	5	8	6	4	5	3	4	12	10	7	9	4	3	5	5	4	2	9	3	9	5	3	10	6	3	4	8	8	7	13	13	10	24	0.254	0.114	5.55E-08	✻	K
L	11	10	9	7	9	11	7	8	6	12	9	10	11	13	6	6	11	7	7	5	8	10	5	8	11	11	6	9	8	9	5	6	12	9	12	6	5	6	13	15	12	11	12	5	8	6	9	7	19	15	12	0.755	0.254	7.43E-05	✻	L
M	2	3	4	2	1	1	3	1	0	1	5	3	3	4	2	5	8	0	2	1	1	3	0	5	0	2	2	4	3	1	0	1	1	3	3	1	1	3	3	11	1	1	3	0	2	2	0	3	2	4	0	–	–	–	–	M
N	3	4	8	4	5	3	3	2	2	2	5	5	2	3	3	3	3	4	5	3	1	6	5	4	3	5	6	5	4	6	5	5	5	3	5	6	4	1	5	5	2	6	7	6	8	2	6	12	3	5	3	1.453	0.662	–	–	N
P	2	11	5	7	4	9	3	4	3	2	3	2	7	5	9	3	3	4	3	6	4	3	3	8	1	4	0	7	1	6	8	4	10	10	6	6	8	8	6	1	7	5	4	3	3	7	14	1	5	2	2	2.500	1.485	–	–	P
Q	4	3	2	5	4	2	6	8	2	7	11	7	4	3	6	8	5	3	7	3	5	4	2	6	3	3	7	3	0	0	3	2	3	5	9	9	4	6	1	5	0	2	2	5	2	8	3	2	4	0	5	0.832	0.513	1.87E-01	n.s.	Q
R	8	2	4	4	1	27	5	8	3	2	5	3	8	8	4	1	5	7	5	5	11	3	8	5	6	5	4	10	7	7	4	3	4	6	5	8	3	4	5	7	4	8	2	2	2	9	6	5	2	4	5	1.096	0.781	–	–	R
S	6	8	10	7	7	11	10	8	11	11	8	9	10	6	7	12	8	9	12	13	8	7	5	9	11	11	5	8	7	6	10	7	11	5	13	3	8	11	10	11	11	12	6	10	8	1	8	11	4	12	8	1.080	0.334	–	–	S
T	6	10	8	5	7	6	9	10	4	9	5	8	9	7	4	3	6	8	9	4	2	14	5	6	6	6	9	4	5	10	6	6	7	6	4	6	4	0	3	6	5	6	1	6	5	5	7	8	7	8	5	1.240	0.500	–	–	T
V	5	6	5	5	6	7	4	5	5	6	8	3	6	2	8	7	9	6	3	9	6	7	6	6	4	4	5	3	7	6	7	9	6	11	5	6	6	8	4	5	5	9	3	8	2	7	7	3	5	3	5	1.152	0.397	–	–	V
W	2	1	0	2	0	0	1	0	0	4	2	2	1	2	1	1	0	0	0	1	1	0	1	1	2	0	3	0	3	1	1	0	0	1	1	2	1	1	0	1	1	2	1	4	4	1	2	2	2	1	2	0.600	0.544	2.37E-04	n.s.	W
Y	4	2	1	7	0	2	5	6	2	4	2	4	2	2	3	6	1	4	1	3	2	2	3	5	4	4	4	3	5	4	2	4	5	4	1	2	2	7	4	10	4	5	3	0	7	4	2	2	5	8	3	1.187	0.687	–	–	Y
total	87	104	104	100	84	116	114	102	90	103	101	96	102	89	93	90	89	99	92	97	88	111	85	109	84	103	88	91	88	90	99	94	119	105	115	93	80	113	87	111	100	99	85	89	91	87	105	105	115	107	103	0.949	0.098			total

Additional file 5. Continued.

FRUIT FLY x-x-S-x-V

position -4

	C50	C49	C48	C47	C46	C45	C44	C43	C42	C41	C40	C39	C38	C37	C36	C35	C34	C33	C32	C31	C30	C29	C28	C27	C26	C25	C24	C23	C22	C21	C20	C19	C18	C17	C16	C15	C14	C13	C12	C11	C10	C9	C8	C7	C6	C5	C4	C3	C2	C1	C0	C-index	SD	*P-value*	mark	
A	6	2	1	1	3	9	2	6	3	7	3	5	3	4	4	8	5	11	9	8	4	2	3	4	5	5	7	5	7	4	5	2	4	7	5	13	5	9	6	3	3	4	4	1	8	3	5	3	3	4	4	1.215	0.645	–	–	A
C	2	1	1	2	0	2	0	1	2	4	1	0	2	1	2	1	2	3	1	1	0	2	0	1	3	1	2	2	0	3	3	2	2	1	1	2	1	1	0	1	1	0	3	1	1	2	3	0	0	2	0	–	–	–	–	C
D	6	3	1	5	3	6	4	2	2	4	3	1	4	0	2	6	3	2	3	3	5	3	4	4	3	6	4	1	1	3	4	1	6	4	4	3	5	6	3	4	2	4	3	4	9	6	2	4	1	2	5	0.696	0.353	1.00E+00	n.s.	D
E	3	4	3	4	0	6	4	2	3	2	3	1	3	4	5	3	2	6	2	5	5	6	5	2	4	1	7	4	3	2	2	3	1	5	2	4	4	7	2	8	3	3	4	3	4	5	1	3	4	1	5	0.692	0.346	1.00E+00	n.s.	E
F	3	2	3	4	3	5	0	2	1	3	2	4	1	1	3	4	2	1	6	2	0	1	3	0	1	4	4	4	6	3	1	0	0	2	4	11	3	3	1	1	4	3	1	2	6	1	2	1	1	3	1	2.560	2.012	–	–	F
G	3	5	1	14	4	3	4	6	3	2	5	3	2	4	3	5	7	6	3	2	2	4	4	7	1	7	5	5	2	8	8	1	9	4	5	2	4	6	4	5	5	6	4	1	4	7	1	1	1	2	6	0.700	0.421	–	–	G
H	1	1	4	2	0	4	1	1	1	1	1	1	2	1	1	5	1	0	1	0	2	2	2	1	1	2	4	2	4	0	2	3	2	1	1	0	2	2	1	4	1	2	2	4	0	1	1	1	0	0	3	0.527	0.421	1.04E-03	n.s.	H
I	3	1	5	9	8	6	2	2	5	2	6	5	1	0	4	5	4	5	4	6	4	3	2	1	6	1	7	5	3	2	5	2	5	8	5	4	6	9	1	6	5	4	3	2	6	6	0	8	3	2	1	4.140	2.312	–	–	I
K	1	1	2	2	5	3	2	5	0	3	3	4	4	5	5	4	3	4	5	5	7	2	10	4	1	3	2	3	5	1	2	2	4	0	2	4	6	3	3	2	1	2	4	4	3	2	4	3	7	1	8	0.408	0.238	5.62E-08	*	K
L	8	5	2	4	4	8	8	7	8	5	7	4	7	3	4	3	5	6	2	7	2	6	3	5	8	8	25	7	3	7	5	12	5	5	6	5	5	11	7	4	5	9	6	9	8	5	12	8	3	3	5	1.256	0.725	–	–	L
M	1	0	2	2	1	4	1	3	1	1	0	2	1	2	2	3	2	0	2	3	1	2	0	2	2	3	0	2	2	2	1	2	0	2	3	2	1	5	1	1	1	6	0	1	2	0	0	2	1	0	5	0.320	0.256	5.55E-08	*	M
N	5	4	0	8	4	5	0	2	1	2	5	2	2	4	1	4	3	2	5	4	1	3	0	0	3	2	4	1	4	5	3	2	0	3	2	5	2	4	3	4	1	4	3	3	3	1	4	0	5	4	5	0.568	0.351	4.82E-01	n.s.	N
P	5	3	2	3	7	2	3	3	4	6	2	1	4	2	2	1	6	4	4	4	4	4	0	3	1	5	5	4	7	1	6	3	3	2	1	5	3	5	5	3	4	2	6	4	2	6	3	1	4	3	9	0.384	0.189	5.55E-08	*	P
Q	4	4	6	3	25	3	9	0	3	2	3	0	4	2	4	3	1	3	5	3	2	3	3	2	3	2	2	2	2	2	4	1	2	2	3	2	2	4	6	4	3	1	5	3	4	2	3	1	1	0	7	0.466	0.505	5.70E-08	*	Q
R	6	0	5	4	4	3	5	3	4	2	3	2	2	2	7	3	3	4	3	2	3	3	4	5	3	3	4	5	3	3	4	5	1	1	3	2	7	2	4	5	5	4	7	6	0	5	1	3	3	2	7	0.494	0.237	2.76E-05	*	R
S	4	8	6	3	9	5	10	6	5	10	4	7	5	5	11	4	3	3	12	9	8	8	6	4	5	5	4	4	9	9	6	8	7	10	7	4	9	2	9	4	8	11	8	6	4	12	9	6	2	2	12	0.542	0.227	3.53E-03	n.s.	S
T	5	2	3	4	5	8	2	5	4	4	5	2	3	5	2	0	2	9	4	6	2	8	3	6	4	4	6	2	6	7	1	3	2	2	3	5	2	2	2	3	6	5	8	2	5	3	4	5	3	4	3	1.320	0.667	–	–	T
V	6	3	6	2	4	2	6	9	5	1	4	4	5	5	9	6	3	0	4	10	9	6	5	4	6	4	6	7	4	4	2	3	5	4	0	8	6	11	4	5	5	10	6	3	3	4	2	3	4	2	4	1.195	0.619	–	–	V
W	0	1	1	1	1	0	0	0	1	0	0	2	0	1	1	1	1	1	2	1	1	1	1	1	2	1	0	0	1	0	0	0	1	0	2	1	0	0	1	0	0	2	2	1	0	0	0	1	0	1	1	0.700	0.678	–	–	W
Y	1	4	1	1	4	1	0	1	1	3	1	1	2	0	3	2	0	4	2	1	4	3	2	1	0	1	1	1	2	1	1	1	2	3	0	1	3	3	3	1	0	3	1	6	1	2	3	2	2	3	6	0.300	0.218	5.55E-08	*	Y
total	73	54	55	78	94	85	63	66	57	64	61	51	57	51	75	71	58	74	79	82	66	72	60	57	62	68	99	66	74	67	65	56	61	66	59	83	76	95	66	68	63	85	80	66	73	73	60	56	48	41	97	0.697	0.126			total

position -3

	C50	C49	C48	C47	C46	C45	C44	C43	C42	C41	C40	C39	C38	C37	C36	C35	C34	C33	C32	C31	C30	C29	C28	C27	C26	C25	C24	C23	C22	C21	C20	C19	C18	C17	C16	C15	C14	C13	C12	C11	C10	C9	C8	C7	C6	C5	C4	C3	C2	C1	C0	C-index	SD	*P-value*	mark	
A	6	5	4	7	5	5	6	7	6	3	6	1	2	3	5	6	2	7	5	6	8	4	2	4	2	0	4	3	10	4	6	4	3	5	6	5	4	10	7	2	8	2	7	4	3	4	4	2	3	0	5	0.908	0.450	–	–	A
C	1	0	0	1	2	3	2	1	2	3	0	0	0	0	1	3	1	2	2	1	2	0	0	1	2	1	2	1	2	2	1	1	3	0	2	1	0	2	1	0	1	3	2	1	0	2	0	1	1	1	2	0.610	0.477	4.32E-01	n.s.	C
D	4	5	7	1	2	8	1	1	3	0	4	0	2	2	1	2	3	3	6	4	1	3	4	4	6	0	3	5	2	5	6	1	6	0	2	4	3	4	1	2	4	4	4	3	2	4	4	5	1	2	7	0.440	0.275	2.14E-06	*	D
E	7	2	4	3	4	7	1	4	0	4	2	4	3	3	4	4	4	2	5	7	3	3	4	6	5	3	6	2	4	2	2	3	4	1	4	0	1	5	5	4	1	7	3	3	4	6	5	4	5	0	11	0.325	0.165	5.55E-08	*	E
F	2	1	1	0	1	0	1	0	2	1	1	3	1	0	2	2	4	3	3	2	2	1	3	3	3	7	3	2	1	2	2	1	1	2	2	9	6	1	5	7	2	1	1	0	2	0	0	2	2	2	5	0.420	0.380	7.78E-08	*	F
G	5	9	4	6	5	10	7	2	7	6	4	5	4	5	8	6	8	5	4	4	3	8	2	4	5	8	7	7	2	7	3	0	0	11	3	8	3	6	3	4	7	5	5	5	4	3	3	5	1	0	5	0.984	0.498	–	–	G
H	0	0	2	0	3	3	3	0	0	3	0	3	1	2	4	0	0	4	0	2	3	0	1	1	0	1	3	2	2	3	0	5	1	1	0	4	0	0	3	1	2	4	2	3	3	0	1	1	0	0	7	0.220	0.211	5.55E-08	*	H
I	4	3	2	3	3	4	3	3	2	3	7	1	7	1	4	2	3	7	4	2	2	1	1	5	4	7	2	4	7	6	4	3	5	3	4	6	3	5	6	5	6	5	3	5	6	2	1	4	1	4	4	0.940	0.453	–	–	I
K	1	7	2	15	4	2	1	3	2	4	1	6	5	3	1	4	3	2	1	8	0	3	3	2	3	5	6	4	3	3	4	3	1	2	3	6	4	1	4	5	2	6	5	6	4	10	2	2	5	2	3	1.227	0.866	–	–	K
L	5	3	2	1	7	5	5	7	2	9	5	5	3	6	5	6	6	5	7	8	5	6	4	5	8	4	29	7	9	3	6	5	8	8	5	4	6	8	3	6	6	7	6	4	6	9	6	6	0	3	8	0.735	0.487	–	–	L
M	1	0	4	4	0	0	1	3	0	2	3	3	0	2	4	3	1	3	1	1	2	2	2	3	2	1	2	2	1	0	0	1	0	1	2	2	2	1	0	2	3	2	4	2	2	2	1	1	1	0	1	1.640	1.191	–	–	M
N	1	3	4	0	0	3	9	3	3	3	1	1	4	3	4	3	2	3	5	1	3	3	6	2	2	4	7	3	3	6	2	7	0	3	6	4	7	2	0	2	2	1	4	0	3	4	4	4	2	2	6	0.513	0.335	1.59E-04	n.s.	N
P	6	1	3	6	9	9	3	5	5	2	4	3	6	3	1	6	1	2	4	2	8	4	5	1	4	2	4	7	4	1	5	3	5	4	4	6	5	11	5	4	2	7	8	4	3	6	3	1	0	2	3	1.393	0.796	–	–	P
Q	4	1	2	2	0	2	2	3	2	5	4	2	4	2	6	4	2	6	2	3	2	3	4	1	2	1	4	1	3	0	2	2	2	2	3	0	2	6	2	1	3	3	4	3	4	1	3	1	5	3	5	0.524	0.297	1.70E-03	n.s.	Q
R	5	3	2	4	5	3	9	1	4	2	4	7	3	4	3	2	1	4	7	3	2	9	5	2	5	4	2	5	3	4	3	4	4	4	3	6	6	5	3	6	3	6	8	6	6	2	6	2	3	4	4	1.035	0.477	–	–	R
S	4	3	5	12	31	8	3	6	7	7	6	2	4	6	11	5	9	5	9	6	6	7	2	4	2	5	8	1	10	11	7	10	5	10	3	8	8	11	6	7	3	10	6	3	8	4	6	6	7	4	10	0.674	0.443	7.61E-01		S
T	9	3	3	5	2	2	3	7	2	1	2	2	4	3	3	7	5	2	3	13	6	3	5	2	3	4	1	4	3	5	5	1	6	1	3	2	5	6	4	0	3	4	3	6	5	4	3	1	3	7	4	0.945	0.577	–	–	T
V	2	3	2	5	5	6	2	4	5	6	3	1	3	1	5	4	2	8	5	4	6	1	5	6	4	4	3	5	2	2	5	1	4	3	2	3	4	7	5	8	4	5	5	3	4	9	5	4	5	5	6	0.683	0.307	1.00E+00	n.s.	V
W	1	0	1	0	1	1	0	2	0	0	1	0	0	0	0	1	1	0	2	2	1	2	1	1	0	1	1	1	0	0	0	1	0	1	0	1	1	1	1	1	0	2	0	1	0	1	3	0	0	0	0	–	–	–	–	W
Y	5	2	1	3	5	4	1	4	3	0	3	2	1	2	3	1	0	1	4	3	1	9	1	0	0	6	2	0	3	1	2	0	3	4	2	4	6	3	2	1	1	1	0	4	4	0	0	4	3	0	1	2.300	1.951	–	–	Y
total	73	54	55	78	94	85	63	66	57	64	61	51	57	51	75	71	58	74	79	82	66	72	60	57	62	68	99	66	74	67	65	56	61	66	59	83	76	95	66	68	63	85	80	66	73	73	60	56	48	41	97	0.697	0.126			total

position -1

	C50	C49	C48	C47	C46	C45	C44	C43	C42	C41	C40	C39	C38	C37	C36	C35	C34	C33	C32	C31	C30	C29	C28	C27	C26	C25	C24	C23	C22	C21	C20	C19	C18	C17	C16	C15	C14	C13	C12	C11	C10	C9	C8	C7	C6	C5	C4	C3	C2	C1	C0	C-index	SD	*P-value*	mark	
A	10	3	5	7	29	4	4	3	6	5	8	5	1	5	3	8	5	3	3	7	5	3	5	7	5	3	7	2	4	6	8	10	6	7	3	8	2	7	4	6	8	10	7	1	6	6	5	2	6	4	9	0.638	0.449	1.46E-01	n.s.	A
C	1	0	0	3	2	3	2	1	1	0	1	0	0	1	3	2	2	5	0	1	1	1	1	1	1	1	4	1	2	0	2	1	1	2	1	1	2	2	0	2	3	1	1	2	2	1	1	0	1	0	6	0.223	0.180	5.55E-08	*	C
D	5	5	5	2	2	1	2	4	4	2	3	3	2	0	4	4	5	6	10	5	2	2	4	5	3	0	4	1	3	3	1	1	2	3	4	4	4	2	5	3	8	3	1	3	3	6	3	1	2	3	8	0.408	0.238	5.59E-08	*	D
E	3	3	5	3	1	1	3	1	2	2	3	2	1	5	2	7	1	0	5	3	2	3	3	4	5	5	3	3	2	4	4	3	5	6	1	2	5	6	1	3	3	6	6	2	4	4	3	1	3	3	7	0.451	0.235	2.70E-06	*	E
F	5	0	6	0	5	5	3	4	0	2	3	4	4	3	2	2	1	6	5	12	1	2	2	0	5	3	4	6	2	1	3	2	2	2	1	3	5	7	2	2	2	2	5	3	3	3	2	1	0	2	9	0.333	0.244	5.55E-08	*	F
G	5	4	3	5	2	8	5	7	4	5	2	4	3	7	4	4	6	5	5	4	4	10	5	4	6	5	26	7	1	7	5	5	5	8	6	3	5	5	3	4	4	9	5	10	4	3	3	5	2	2	3	1.753	1.193	–	–	G
H	1	2	0	2	1	2	2	1	0	1	3	2	4	0	1	0	1	2	0	1	0	3	0	0	1	3	2	1	2	1	1	1	1	2	3	1	1	0	1	3	2	1	3	1	1	0	0	0	2	0	1	1.260	1.046	–	–	H
I	4	1	1	3	6	2	7	5	4	4	3	2	2	2	1	2	3	3	3	1	3	4	3	1	1	5	3	3	2	2	0	4	2	3	4	5	5	2	6	4	2	5	5	5	4	4	3	5	6	1	8	0.403	0.202	5.58E-08	*	I
K	4	1	6	2	2	5	1	7	1	2	2	3	5	4	3	2	5	2	5	4	2	3	0	4	4	2	1	1	6	3	4	2	5	0	6	5	4	8	1	4	3	3	5	0	3	2	4	4	1	1	6	0.523	0.314	1.31E-02	n.s.	K
L	5	5	2	9	7	8	6	6	5	8	5	4	9	2	8	6	8	8	4	7	6	6	4	3	5	4	9	10	7	8	6	4	7	2	5	6	8	7	7	4	9	8	5	3	8	4	5	6	5	6	7	0.854	0.286	–	–	L
M	1	3	1	2	3	2	1	2	1	0	0	1	0	1	0	2	2	2	1	2	1	0	4	1	2	0	0	1	0	0	2	2	1	3	3	1	3	3	3	2	0	1	2	1	2	1	1	1	1	0	0	–	–	–	–	M
N	3	2	1	1	2	3	2	4	4	1	1	2	3	1	4	7	1	2	4	4	3	4	2	2	3	4	5	3	5	2	7	2	2	4	4	5	4	5	4	4	3	2	5	0	5	1	1	3	4	1	5	0.604	0.319	6.11E-01	n.s.	N
P	7	4	1	2	7	2	2	3	2	6	3	1	1	3	4	4	3	5	5	3	8	2	5	5	4	1	4	4	6	2	3	1	3	3	0	5	5	7	5	3	1	5	6	4	5	5	4	4	2	3	4	0.915	0.462	–	–	P
Q	0	2	2	1	4	3	5	3	4	4	3	2	3	1	10	4	3	5	4	2	4	2	1	2	1	5	7	1	5	1	1	1	0	0	2	2	2	3	2	5	0	2	4	5	3	4	3	5	1	0	0	–	–	–	–	Q
R	2	2	2	3	1	1	1	1	2	2	3	2	1	2	5	1	1	3	4	1	2	1	1	3	2	4	1	4	3	4	2	2	2	0	1	8	2	8	4	1	2	2	3	3	0	4	2	4	1	1	5	0.468	0.330	5.62E-06	*	R
S	8	3	3	3	4	11	9	6	9	8	3	2	7	8	9	3	4	6	10	8	14	14	4	3	6	6	6	7	7	9	7	8	8	11	2	6	8	7	10	6	4	13	5	10	6	10	4	4	3	6	8	0.845	0.379	–	–	S
T	1	2	3	18	6	8	3	1	2	6	3	4	7	1	3	3	2	3	1	6	2	4	4	4	5	6	4	3	6	4	2	3	3	2	5	4	4	4	4	2	1	4	4	4	2	9	4	4	5	3	4	0.990	0.674	–	–	T
V	6	7	8	3	8	11	2	6	5	2	9	5	2	3	5	7	3	6	7	7	4	7	9	6	2	9	8	5	7	9	5	3	4	5	3	10	3	7	2	7	4	4	7	5	4	4	5	5	3	3	4	1.355	0.583	–	–	V
W	1	1	0	2	0	2	1	0	0	1	2	1	1	0	2	1	0	1	2	0	0	1	2	0	0	1	0	0	1	0	1	0	1	1	1	0	0	0	0	1	0	1	0	1	1	0	5	1	0	1	0	–	–	–	–	W
Y	1	4	1	7	2	3	2	1	1	3	1	2	1	2	2	2	2	1	1	4	2	0	1	2	1	1	1	3	3	1	1	1	1	2	4	4	4	5	2	2	4	3	1	3	7	2	2	0	0	1	3	0.713	0.517	–	–	Y
total	73	54	55	78	94	85	63	66	57	64	61	51	57	51	75	71	58	74	79	82	66	72	60	57	62	68	99	66	74	67	65	56	61	66	59	83	76	95	66	68	63	85	80	66	73	73	60	56	48	41	97	0.697	0.126			total

Additional file 5. Continued.

FRUIT FLY x-x-T-x-I

position -4

	C50	C49	C48	C47	C46	C45	C44	C43	C42	C41	C40	C39	C38	C37	C36	C35	C34	C33	C32	C31	C30	C29	C28	C27	C26	C25	C24	C23	C22	C21	C20	C19	C18	C17	C16	C15	C14	C13	C12	C11	C10	C9	C8	C7	C6	C5	C4	C3	C2	C1	C0	C-index	SD	*P-value*	mark	
A	2	7	1	2	2	3	1	5	2	4	2	1	0	4	2	1	2	1	4	1	7	2	7	2	2	5	2	2	1	2	4	0	5	2	5	3	2	1	2	0	2	2	3	3	1	3	0	0	1	2	0	–	–	–	–	A
C	0	3	1	1	4	2	0	1	1	1	0	2	0	1	3	0	0	1	0	1	0	1	0	1	5	0	2	5	0	1	0	2	4	1	1	0	2	0	1	0	0	0	0	0	1	2	2	0	2	2	1	1.140	1.325	–	–	C
D	4	7	3	6	1	2	2	0	5	7	3	0	2	2	3	0	1	2	3	1	2	1	3	2	2	1	3	1	3	1	1	0	1	3	5	2	3	2	1	0	2	2	1	1	1	1	2	4	15	1	1	2.420	2.459	–	–	D
E	1	3	1	2	0	0	0	1	1	1	1	1	2	0	1	3	1	3	3	2	0	2	1	1	2	1	5	2	0	1	0	3	2	3	2	5	3	3	3	1	2	1	0	1	2	1	4	2	6	1	9	0.193	0.154	5.55E-08	*	E
F	2	1	2	1	0	1	3	2	1	2	0	3	1	1	2	1	1	1	1	1	0	0	0	5	1	1	2	1	5	5	2	2	1	1	3	3	4	0	0	2	0	0	2	3	6	1	2	0	1	2	2	0.820	0.727	–	–	F
G	2	4	1	4	6	2	1	2	2	4	7	1	1	2	2	1	6	2	2	4	3	4	3	5	1	2	1	4	2	4	1	0	0	1	3	0	2	3	2	3	3	1	4	1	2	3	2	4	3	1	2	1.240	0.791	–	–	G
H	0	0	1	2	1	0	0	0	0	0	0	0	2	2	0	2	0	0	1	4	0	1	0	1	1	0	0	0	2	1	0	1	2	0	1	1	2	0	1	0	1	1	0	1	0	2	2	1	0	1	0	–	–	–	–	H
I	1	0	2	2	5	2	2	1	4	4	0	4	0	2	3	1	0	2	1	2	7	2	4	2	2	1	3	2	3	4	0	4	3	1	0	3	1	4	3	5	2	0	4	0	4	2	8	2	2	1	5	0.468	0.356	3.18E-04	n.s.	I
K	0	1	4	3	23	1	0	2	1	1	3	4	4	2	1	1	4	2	1	4	3	4	6	2	0	2	1	0	3	1	3	1	2	0	2	2	4	3	3	2	0	1	1	2	1	0	2	1	7	3	4	0.620	0.836	1.85E-02	n.s.	K
L	2	8	3	2	11	1	2	6	3	3	4	5	2	3	2	3	8	2	2	4	5	5	2	4	2	4	10	3	3	4	2	6	4	6	7	5	2	5	3	1	5	4	4	1	6	4	4	2	4	4	1	3.940	2.170	–	–	L
M	1	1	1	0	1	1	0	0	2	0	2	0	0	1	0	0	0	1	2	0	1	1	0	2	1	0	3	1	1	0	2	2	0	1	2	0	4	2	1	1	1	0	2	0	1	0	1	0	1	1	1	0.900	0.909	–	–	M
N	0	1	2	0	0	1	6	0	1	0	2	2	1	1	2	4	0	1	1	3	2	1	1	2	0	0	1	1	0	2	0	1	0	1	2	4	5	1	0	2	3	3	2	2	1	0	2	6	2	2	6	0.257	0.248	5.55E-08	*	N
P	3	2	3	1	2	3	2	1	2	2	4	3	2	2	1	1	1	2	1	2	1	1	0	4	2	3	0	1	3	4	2	6	4	2	2	3	3	4	4	2	1	0	4	2	0	3	1	0	2	5	4	0.545	0.341	2.05E-02	n.s.	P
Q	0	0	0	2	0	1	2	2	0	3	0	1	1	1	3	2	1	1	0	5	3	3	2	1	2	0	1	1	1	5	3	2	3	1	1	2	4	2	4	1	2	2	0	2	0	5	3	0	4	0	3	0.567	0.482	3.09E-01	n.s.	Q
R	0	2	1	2	1	1	1	5	1	2	2	1	4	4	1	2	2	2	4	5	1	2	0	1	4	2	4	4	2	2	1	1	2	4	3	0	0	2	3	2	0	2	1	1	3	1	0	2	0	1	4	0.470	0.341	1.67E-04	n.s.	R
S	1	3	1	4	5	1	3	7	2	2	2	1	1	3	4	1	2	3	3	2	1	6	1	2	2	9	3	3	3	2	2	1	5	4	2	3	3	3	3	2	1	1	1	2	0	4	3	2	2	4	7	0.374	0.240	5.56E-08	*	S
T	6	3	1	4	5	3	2	3	1	3	7	5	0	0	3	2	4	0	5	0	3	5	2	2	2	2	6	3	1	2	2	3	4	6	0	3	3	5	0	5	2	2	2	0	2	2	2	2	0	3	2	1.330	0.913	–	–	T
V	0	3	6	8	2	0	3	3	2	3	4	3	1	4	3	3	3	0	5	5	3	4	3	2	6	2	5	1	4	3	5	2	4	5	2	5	4	4	2	3	3	6	2	1	2	1	2	2	1	1	3	1.007	0.573	–	–	V
W	0	0	0	1	1	0	1	1	0	1	2	1	0	1	0	0	0	1	0	0	2	1	1	0	0	0	0	0	0	0	1	0	0	1	0	1	0	1	0	0	0	0	1	1	0	1	1	0	0	0	0	–	–	–	–	W
Y	0	0	0	4	0	3	0	1	2	1	0	0	2	4	0	0	0	2	1	2	1	2	0	2	2	1	2	1	1	0	2	0	2	0	2	2	2	1	2	2	3	3	1	2	2	1	2	2	1	1	2	0.670	0.540	1.00E+00	n.s.	Y
total	25	49	34	51	70	28	31	43	33	44	45	38	26	40	36	28	36	29	40	48	45	48	36	43	39	36	54	36	38	44	33	37	48	43	45	47	53	46	38	34	33	31	35	26	35	37	45	32	54	36	57	0.695	0.153			total

position -3

	C50	C49	C48	C47	C46	C45	C44	C43	C42	C41	C40	C39	C38	C37	C36	C35	C34	C33	C32	C31	C30	C29	C28	C27	C26	C25	C24	C23	C22	C21	C20	C19	C18	C17	C16	C15	C14	C13	C12	C11	C10	C9	C8	C7	C6	C5	C4	C3	C2	C1	C0	C-index	SD	*P-value*	mark	
A	3	9	6	6	2	1	4	7	3	5	1	2	0	2	5	2	3	1	4	4	3	3	3	1	5	5	1	2	2	2	2	3	3	2	3	3	4	3	2	1	0	2	5	0	3	1	4	2	2	4	3	0.973	0.609	–	–	A
C	0	1	2	1	0	1	0	3	0	1	0	2	2	0	1	3	0	1	0	0	0	2	0	1	0	1	1	1	0	0	0	1	0	1	1	3	0	1	1	0	1	2	0	0	1	2	0	1	0	0	0	–	–	–	–	C
D	1	2	2	3	2	2	1	1	1	1	1	1	1	2	0	2	1	2	4	2	2	1	1	2	1	0	4	1	5	2	1	2	4	2	1	0	1	0	1	1	2	2	1	1	3	0	2	1	2	0	4	0.390	0.273	5.97E-08	*	D
E	1	2	1	6	4	2	1	1	1	3	1	2	1	1	4	2	0	1	0	2	2	2	2	2	4	2	0	5	0	2	1	3	3	1	2	4	2	4	3	2	5	3	3	1	1	3	2	7	3	2	11	0.204	0.138	5.55E-08	*	E
F	2	1	1	2	2	2	1	2	0	2	1	1	4	3	1	3	2	2	4	2	2	1	2	2	0	0	3	3	2	1	2	2	4	3	1	4	3	2	0	2	2	2	2	0	2	4	3	2	5	1	2	1.000	0.580	–	–	F
G	2	1	1	1	2	2	3	2	2	4	2	4	0	3	2	1	4	3	2	3	1	3	3	2	2	1	5	0	1	4	0	0	2	2	2	3	4	0	5	1	1	0	2	1	1	2	2	1	1	0	3	0.640	0.441	9.77E-01	n.s.	G
H	0	1	0	1	1	1	1	1	2	2	0	0	0	1	1	0	1	0	0	1	2	2	0	0	1	1	2	5	1	1	1	1	4	4	7	1	0	1	1	2	1	0	1	2	1	0	2	2	4	0	1	1.280	1.415	–	–	H
I	1	7	2	4	3	3	4	0	1	3	2	3	0	4	0	1	1	0	2	3	5	4	1	2	1	1	2	4	5	6	2	3	3	5	1	6	4	4	0	2	1	4	2	0	2	2	3	2	2	4	4	0.635	0.432	7.79E-01	n.s.	I
K	1	2	3	2	21	0	0	1	2	3	2	1	1	1	2	0	2	0	1	1	1	2	3	3	3	2	6	1	1	1	2	2	4	1	1	3	6	4	5	5	0	2	2	6	3	1	4	2	4	2	2	1.280	1.546	–	–	K
L	1	3	4	7	8	2	1	3	9	3	5	3	1	5	2	3	7	3	5	7	8	5	5	2	3	3	5	2	6	3	2	7	3	2	0	3	9	8	3	5	4	3	1	4	3	7	3	2	1	7	3	1.340	0.778	–	–	L
M	1	1	0	0	0	1	1	0	0	2	5	1	2	0	0	1	0	1	1	1	1	2	0	1	0	0	1	0	0	1	1	1	1	1	0	1	0	1	3	0	0	1	2	0	0	0	0	0	0	0	0	–	–	–	–	M
N	1	0	1	3	1	2	1	5	0	0	6	2	2	2	1	1	0	2	0	3	3	5	2	3	4	3	3	2	1	4	2	3	0	1	2	0	1	2	2	2	1	3	3	1	1	1	2	0	9	1	5	0.400	0.345	1.24E-07	*	N
P	1	3	1	1	7	0	4	0	3	3	3	2	2	3	6	1	1	1	2	3	1	0	2	1	2	1	2	1	2	2	3	0	0	3	1	2	3	2	3	1	2	0	0	0	0	3	2	1	4	2	2	0.930	0.742	–	–	P
Q	1	4	3	1	2	1	0	2	1	1	1	1	2	0	0	2	2	1	1	3	2	1	1	0	2	3	2	1	1	7	2	2	4	0	2	2	4	0	2	0	2	0	2	0	1	0	3	2	3	3	4	0.415	0.341	7.17E-07	*	Q
R	3	4	1	5	4	3	2	1	1	2	2	3	1	3	3	2	2	5	0	1	2	6	3	3	2	4	4	0	2	0	2	2	2	4	4	1	1	3	2	1	2	0	2	3	3	1	6	3	6	3	3	0.833	0.510	–	–	R
S	4	3	1	4	2	0	3	4	3	4	3	6	2	2	2	2	4	3	3	1	1	2	3	5	2	2	1	3	2	3	4	1	3	3	4	4	6	2	1	3	3	2	4	2	2	2	2	2	0	3	4	0.665	0.326	9.60E-01	n.s.	S
T	2	1	4	1	5	3	1	2	1	1	3	1	0	5	4	1	2	0	4	4	4	2	1	7	3	6	6	0	1	2	2	2	3	2	4	3	3	2	4	3	2	3	2	2	4	5	3	1	3	2	3	0.880	0.538	–	–	T
V	0	4	0	1	1	2	2	4	2	1	4	1	4	1	2	0	2	1	3	5	1	5	2	4	2	1	3	4	6	1	2	1	2	5	4	2	0	2	0	2	3	1	0	3	1	2	2	1	1	2	2	1.050	0.758	–	–	V
W	0	0	0	0	1	0	0	1	0	1	1	0	1	0	0	0	1	0	2	1	0	0	1	2	0	0	3	0	0	2	2	1	0	0	1	0	1	2	0	0	0	1	0	0	2	0	0	0	1	0	0	–	–	–	–	W
Y	0	0	1	2	2	0	1	3	1	2	2	2	0	2	0	1	1	2	2	1	4	0	1	0	2	0	0	1	0	0	0	0	3	1	4	2	1	3	0	1	1	0	1	0	1	1	0	0	3	0	1	1.100	1.129	–	–	Y
total	25	49	34	51	70	28	31	43	33	44	45	38	26	40	36	28	36	29	40	48	45	48	36	43	39	36	54	36	38	44	33	37	48	43	45	47	53	46	38	34	33	31	35	26	35	37	45	32	54	36	57	0.695	0.153			total

position -1

	C50	C49	C48	C47	C46	C45	C44	C43	C42	C41	C40	C39	C38	C37	C36	C35	C34	C33	C32	C31	C30	C29	C28	C27	C26	C25	C24	C23	C22	C21	C20	C19	C18	C17	C16	C15	C14	C13	C12	C11	C10	C9	C8	C7	C6	C5	C4	C3	C2	C1	C0	C-index	SD	*P-value*	mark	
A	4	5	3	5	6	2	2	4	1	2	4	4	1	2	5	3	5	1	3	3	3	2	5	4	3	2	4	6	8	5	2	2	3	1	2	7	2	1	1	2	5	3	4	2	2	2	0	2	3	1	2	1.540	0.862	–	–	A
C	1	2	0	0	1	1	0	1	0	0	1	1	0	1	0	0	2	0	0	1	2	1	0	1	0	1	1	1	2	0	0	2	0	1	2	0	0	2	1	2	0	3	0	0	0	0	1	1	0	0	1	0.720	0.809	–	–	C
D	0	3	1	1	2	3	0	1	1	2	2	2	2	2	0	4	0	0	1	1	1	5	4	2	1	1	0	0	1	3	0	1	5	3	2	2	7	3	3	0	1	2	4	2	2	0	1	0	3	3	5	0.360	0.310	8.15E-08	*	D
E	0	3	5	2	2	1	1	6	3	4	6	6	1	3	0	3	2	3	4	3	1	1	1	1	2	3	6	2	5	0	2	3	2	3	1	3	6	4	1	1	2	0	0	1	2	2	1	4	3	2	5	0.492	0.346	6.11E-04	n.s.	E
F	0	1	2	2	2	2	0	1	1	1	0	1	1	2	1	1	1	1	0	0	1	1	4	1	3	1	5	0	4	3	1	1	4	1	3	2	3	2	1	0	0	0	2	3	6	2	5	3	0	0	0	–	–	–	–	F
G	4	2	2	1	2	0	2	1	1	1	4	3	0	2	2	1	2	2	1	3	3	3	1	2	3	4	3	2	2	5	1	1	3	3	4	4	4	0	1	4	0	3	1	2	0	2	8	4	17	3	2	1.290	1.290	–	–	G
H	0	1	0	0	1	1	0	0	2	1	0	0	1	2	1	0	0	1	1	2	1	6	1	0	1	2	3	1	0	2	1	0	0	4	1	1	1	2	3	2	2	0	1	2	1	2	1	0	1	2	3	0.387	0.389	1.53E-06	*	H
I	3	2	2	4	0	0	3	3	1	3	1	4	2	5	3	0	2	0	6	4	2	3	0	1	2	2	5	2	3	5	6	4	3	3	2	4	0	1	3	1	0	0	0	2	2	4	4	2	1	2	4	0.585	0.412	3.59E-01	n.s.	I
K	0	5	3	5	4	1	1	2	0	3	2	2	1	1	1	3	1	2	3	5	0	3	1	2	2	2	1	2	1	2	1	0	1	3	2	2	2	1	4	4	3	3	0	3	3	6	4	1	1	5	4	0.550	0.375	2.85E-02	n.s.	K
L	2	7	4	2	5	5	2	4	3	5	4	3	4	3	6	1	4	5	1	4	4	2	3	5	0	1	5	0	0	1	1	2	5	5	4	3	2	5	3	3	4	5	4	0	3	2	4	1	1	0	3	1.013	0.595	–	–	L
M	1	0	0	1	1	1	1	0	2	1	1	0	1	3	1	0	1	0	1	2	1	0	0	0	1	0	0	1	1	1	1	0	1	0	2	0	0	1	2	0	0	0	0	0	0	0	0	2	2	1	0	–	–	–	–	M
N	1	2	3	3	3	3	2	5	0	3	1	0	1	2	2	1	2	1	2	5	3	6	1	5	3	3	1	5	1	1	0	4	4	3	1	1	4	0	2	1	1	2	2	0	1	3	2	2	5	1	3	0.733	0.513	–	–	N
P	0	0	1	3	3	3	2	3	5	3	1	2	1	0	2	3	2	0	1	3	6	4	1	1	6	3	3	1	1	2	4	2	1	1	3	3	2	2	2	0	0	3	4	0	5	2	2	2	4	4	2	1.120	0.773	–	–	P
Q	0	1	2	3	2	0	1	2	2	3	3	1	1	0	3	0	1	0	0	1	1	2	2	2	2	2	1	1	0	2	4	1	0	0	0	3	3	1	0	1	0	2	1	2	1	1	3	2	2	2	4	0.350	0.267	5.90E-08	*	Q
R	3	2	0	1	21	0	1	2	1	3	4	1	1	4	1	4	1	3	2	3	2	3	4	1	1	1	2	6	2	2	2	2	1	3	2	1	3	4	5	3	3	1	2	0	1	1	2	0	3	3	6	0.413	0.497	5.76E-08	*	R
S	2	0	1	7	3	1	3	3	4	1	3	2	1	2	0	0	4	2	3	3	5	2	1	9	2	3	3	3	1	4	2	6	4	5	3	4	3	6	2	2	3	3	4	3	1	3	2	1	2	3	6	0.467	0.294	5.84E-06	*	S
T	1	5	3	5	6	2	4	1	3	5	2	2	2	0	3	0	3	2	4	2	3	2	3	2	4	0	6	3	2	0	2	4	5	2	2	3	5	6	0	4	2	1	4	1	1	3	2	2	3	1	3	0.887	0.545	–	–	T
V	2	3	0	2	5	1	4	3	3	1	3	3	4	3	5	3	1	3	4	2	4	2	2	2	2	3	4	0	3	4	3	2	2	0	6	4	3	2	3	0	5	0	0	1	1	0	1	2	2	2	1	2.400	1.498	–	–	V
W	0	4	0	0	1	0	1	0	0	1	1	1	0	0	0	0	2	1	2	0	0	0	0	1	1	0	0	0	0	0	0	0	1	2	1	0	0	2	0	2	2	0	0	0	2	1	0	0	0	1	1	0.600	0.881	4.88E-01	n.s.	W
Y	1	1	2	4	0	1	1	1	0	1	2	0	1	3	0	1	0	2	1	1	2	0	2	1	0	2	1	0	1	2	0	0	3	0	2	0	3	1	1	2	0	0	2	2	1	1	2	1	1	0	2	0.560	0.491	1.19E-01	n.s.	Y
total	25	49	34	51	70	28	31	43	33	44	45	38	26	40	36	28	36	29	40	48	45	48	36	43	39	36	54	36	38	44	33	37	48	43	45	47	53	46	38	34	33	31	35	26	35	37	45	32	54	36	57	0.695	0.153			total

Additional file 5. Continued.

FRUIT FLY x-x-T-x-L

position -4

	C50	C49	C48	C47	C46	C45	C44	C43	C42	C41	C40	C39	C38	C37	C36	C35	C34	C33	C32	C31	C30	C29	C28	C27	C26	C25	C24	C23	C22	C21	C20	C19	C18	C17	C16	C15	C14	C13	C12	C11	C10	C9	C8	C7	C6	C5	C4	C3	C2	C1	C0	C-index	SD	*P-value*	mark	
A	5	4	3	9	4	4	5	12	6	2	6	4	6	8	7	3	5	2	8	8	3	2	4	6	5	5	8	6	3	2	7	2	1	4	7	4	7	3	9	2	4	5	2	4	1	4	7	4	3	2	10	0.474	0.238	1.25E-01	n.s.	A
C	1	1	1	2	1	1	4	1	2	0	2	0	1	0	2	1	1	2	1	0	0	2	2	2	2	0	2	1	0	1	2	1	2	2	4	0	2	1	1	0	2	2	0	2	0	0	0	1	2	1	3	0.407	0.325	4.92E-01	n.s.	C
D	2	3	2	6	4	3	4	3	1	3	3	2	1	1	5	4	5	2	1	4	2	3	6	2	2	4	2	1	4	2	1	1	3	2	1	1	4	3	1	3	1	2	5	0	3	4	3	3	3	1	3	0.880	0.475	–	–	D
E	2	5	2	3	0	6	6	5	7	3	5	5	7	4	4	4	6	1	4	4	4	0	3	4	29	2	7	2	3	4	0	7	4	5	4	3	0	8	8	3	3	2	6	4	2	4	0	3	5	2	11	0.389	0.376	1.11E-06	✻	E
F	6	4	3	3	1	1	1	8	7	2	4	6	0	2	2	3	1	5	3	1	7	5	5	5	2	4	3	4	1	2	2	4	2	5	4	1	3	2	3	5	2	1	4	9	5	4	2	0	5	4	3	1.120	0.683	–	–	F
G	6	2	5	5	3	2	3	5	3	4	3	3	5	1	6	4	5	6	5	5	2	3	3	8	2	8	5	7	2	4	5	4	4	3	1	2	3	4	2	4	1	2	6	5	2	1	7	2	4	2	5	0.756	0.364	–	–	G
H	1	1	2	1	0	0	4	3	0	2	2	1	6	0	1	2	4	1	2	0	2	0	1	1	1	1	3	4	0	2	3	3	2	3	3	2	2	2	0	2	2	1	2	2	1	1	2	2	4	1	2	0.880	0.643	–	–	H
I	6	0	2	4	5	3	2	3	5	4	1	4	3	3	4	5	3	1	5	4	2	3	4	2	2	0	4	1	2	1	5	3	3	5	7	5	4	3	4	6	6	0	2	2	2	5	3	4	5	3	7	0.471	0.238	5.47E-01	n.s.	I
K	3	3	2	5	6	3	3	2	7	6	3	6	1	4	5	2	3	2	4	5	4	3	3	2	4	0	2	3	6	4	5	4	4	3	3	3	4	5	6	4	10	4	1	5	4	5	3	4	4	3	15	0.253	0.113	5.55E-08	✻	K
L	9	8	10	6	5	12	8	4	9	12	13	5	8	10	6	11	10	10	7	7	7	4	5	10	1	2	10	8	8	9	4	11	5	7	6	10	6	6	6	9	4	9	12	6	8	5	5	5	11	6	10	0.750	0.274	–	–	L
M	1	7	2	1	2	0	0	4	2	3	4	2	1	0	3	1	2	3	5	2	4	2	0	0	2	4	2	2	1	0	2	1	2	1	1	4	3	5	2	1	0	3	2	2	2	1	2	2	0	1	2	0.990	0.752	–	–	M
N	2	1	2	6	5	1	2	4	2	0	1	4	3	4	5	2	4	3	4	4	5	4	1	4	4	1	4	2	5	1	3	3	4	3	4	3	1	2	3	4	3	2	5	3	2	4	3	3	4	0	7	0.426	0.203	3.09E-02	n.s.	N
P	1	3	1	1	3	2	4	4	1	1	1	1	5	4	2	3	6	8	2	3	4	2	4	3	2	3	3	1	4	1	2	3	6	2	3	6	4	5	2	2	2	3	3	4	2	2	3	2	4	1	6	0.480	0.262	1.95E-01	n.s.	P
Q	2	3	3	0	1	4	1	1	0	1	6	2	3	3	3	3	2	1	1	1	5	7	2	2	2	7	3	2	0	0	2	5	3	0	1	3	0	2	1	4	3	1	4	5	2	0	3	2	3	1	7	0.331	0.251	1.82E-07	✻	Q
R	2	6	1	4	2	9	5	1	2	4	5	3	2	5	5	0	1	3	5	6	5	2	0	2	2	4	3	4	2	7	2	5	6	2	4	7	6	2	3	1	2	4	3	5	4	4	2	1	5	6	2	1.760	0.996	–	–	R
S	5	5	2	6	3	3	6	9	5	4	8	7	2	1	4	2	7	6	1	6	6	4	17	5	4	3	2	6	3	8	5	6	3	6	4	2	5	8	5	4	7	2	2	6	5	3	1	4	2	6	12	0.393	0.225	4.42E-06	✻	S
T	7	2	3	4	6	4	5	4	3	4	1	2	4	4	3	3	6	6	3	4	3	4	4	6	3	3	4	5	3	2	5	8	7	4	4	2	4	1	2	2	9	5	3	3	2	4	6	5	4	1	6	0.653	0.291	–	–	T
V	2	5	3	3	4	3	9	2	3	2	2	4	4	5	5	6	6	6	3	7	3	5	3	6	4	3	3	5	6	3	7	5	4	6	4	6	5	8	5	4	4	7	4	3	2	2	3	5	5	3	6	0.723	0.279	–	–	V
W	0	1	0	3	1	0	0	2	0	0	0	0	0	1	0	0	0	0	2	1	2	1	0	2	2	1	1	1	1	0	0	0	0	0	0	0	0	1	1	0	2	1	1	1	1	0	0	0	2	1	1	0.660	0.798	–	–	W
Y	0	3	4	1	2	0	1	2	4	2	3	1	1	6	2	1	5	0	4	2	3	0	3	1	1	0	1	1	0	3	1	2	1	0	3	2	6	3	3	1	2	1	1	1	2	3	4	0	0	2	3	0.633	0.514	–	–	Y
total	63	67	53	73	58	61	73	79	69	59	73	62	63	66	74	60	82	68	70	74	73	56	70	73	76	55	72	66	54	56	63	78	66	63	68	66	69	74	67	61	69	57	68	72	52	56	59	52	75	47	121	0.542	0.067			total

position -3

	C50	C49	C48	C47	C46	C45	C44	C43	C42	C41	C40	C39	C38	C37	C36	C35	C34	C33	C32	C31	C30	C29	C28	C27	C26	C25	C24	C23	C22	C21	C20	C19	C18	C17	C16	C15	C14	C13	C12	C11	C10	C9	C8	C7	C6	C5	C4	C3	C2	C1	C0	C-index	SD	*P-value*	mark	
A	4	7	3	4	3	4	5	11	5	2	9	7	6	3	6	8	6	6	9	7	6	3	4	8	8	6	6	2	5	3	8	6	4	7	8	7	3	9	3	5	4	3	6	4	4	4	5	3	5	3	3	1.780	0.705	–	–	A
C	1	0	0	5	2	2	0	1	5	1	2	2	0	0	1	1	0	0	3	1	1	2	2	0	2	1	1	0	1	1	2	1	2	1	3	1	0	1	0	0	2	1	1	0	0	2	1	0	1	1	4	0.290	0.287	6.40E-08	✻	C
D	2	4	4	5	5	1	3	5	7	4	4	1	4	1	6	3	6	2	1	2	5	1	13	2	27	1	5	1	2	1	3	6	4	4	5	3	4	6	1	4	7	1	3	1	2	4	3	0	1	2	2	1.920	2.029	–	–	D
E	4	5	4	3	2	4	5	2	3	2	2	5	7	2	6	2	7	3	2	4	7	5	4	1	3	3	4	1	1	6	3	4	7	2	1	4	4	3	3	3	3	4	4	2	4	4	2	2	3	6	8	0.443	0.205	3.64E-03	n.s.	E
F	1	3	3	3	1	1	3	4	4	1	2	2	3	1	2	2	1	1	3	4	0	3	1	3	0	2	3	2	4	3	6	2	1	5	4	4	4	0	2	2	3	2	3	3	5	3	4	2	4	2	3	0.847	0.453	–	–	F
G	2	8	3	3	1	5	5	6	1	4	5	6	3	6	9	2	6	3	5	6	7	2	5	9	2	2	8	2	1	0	2	5	2	1	1	8	3	4	4	5	4	5	7	7	4	2	3	3	4	1	4	1.010	0.582	–	–	G
H	0	1	3	2	2	1	1	1	3	1	3	4	0	1	2	1	0	1	5	2	0	0	2	2	3	1	1	3	3	1	0	3	0	0	1	3	2	2	1	1	0	1	2	1	0	3	0	0	2	2	6	0.247	0.203	5.55E-08	✻	H
I	5	6	3	0	4	3	3	7	7	6	5	3	3	4	2	3	8	5	5	3	3	6	6	2	1	4	2	6	1	7	1	6	1	1	4	1	3	3	3	2	2	4	5	2	3	5	6	2	2	2	6	0.603	0.326	–	–	I
K	1	3	3	6	0	7	8	6	5	2	5	2	3	4	9	2	6	4	2	3	2	1	0	6	1	5	4	4	2	3	3	5	0	4	8	4	7	4	5	3	5	4	5	5	9	2	2	4	5	8	9	0.447	0.253	4.44E-02	n.s.	K
L	9	7	2	3	3	5	11	6	4	7	3	9	6	5	7	12	8	6	3	7	3	1	3	8	6	2	11	9	8	6	7	5	4	3	4	7	8	8	8	8	9	4	5	10	5	7	12	13	8	3	7	0.909	0.411	–	–	L
M	1	1	0	2	0	3	2	0	1	2	1	1	2	1	2	3	1	1	2	1	0	0	2	1	0	4	2	1	0	3	0	2	1	3	0	0	2	3	1	0	2	0	1	0	1	0	0	2	2	0	1	1.200	1.069	–	–	M
N	5	3	1	2	3	4	2	0	2	1	6	2	3	6	3	3	4	4	2	2	3	5	0	2	0	3	3	5	1	1	1	1	3	5	4	3	3	2	3	6	3	2	0	4	1	2	0	2	7	0	7	0.380	0.251	6.25E-05	✻	N
P	2	2	5	4	5	0	2	0	4	4	5	3	3	4	4	1	4	7	3	2	7	3	4	7	4	3	2	6	2	3	5	7	5	3	6	4	6	2	6	3	3	2	3	4	1	3	4	3	3	3	5	0.724	0.345	–	–	P
Q	3	7	7	0	4	2	1	4	1	2	5	0	3	3	3	1	4	2	3	2	4	7	0	4	3	1	3	4	4	4	0	4	3	1	3	0	1	5	5	4	5	2	1	3	3	0	1	2	4	1	6	0.463	0.309	8.15E-01	n.s.	Q
R	4	0	1	3	5	3	6	6	5	1	4	1	4	4	1	1	4	2	4	7	4	6	8	1	7	5	2	2	4	1	3	3	5	6	1	6	2	2	3	2	4	9	4	4	3	1	4	5	2	3	4	0.890	0.513	–	–	R
S	3	4	3	10	6	6	2	4	2	3	4	4	5	4	5	7	6	3	4	6	3	4	3	4	2	3	3	4	5	5	7	9	8	4	3	2	5	6	2	6	3	2	9	8	4	6	4	1	8	5	13	0.352	0.160	7.57E-08	✻	S
T	7	1	3	2	4	6	5	6	5	6	4	5	0	6	3	3	3	9	5	3	8	1	6	5	7	2	2	7	4	5	3	0	3	5	4	2	8	6	7	4	3	2	4	2	3	2	2	4	3	2	18	0.224	0.118	5.55E-08	✻	T
V	6	4	5	11	7	2	4	3	3	5	2	3	5	7	1	1	7	4	5	6	4	4	4	5	0	5	6	1	5	1	4	4	10	5	4	0	3	5	6	2	4	5	2	6	0	3	4	2	8	1	11	0.371	0.216	9.81E-07	✻	V
W	0	0	0	2	0	0	0	4	2	0	0	0	2	0	2	2	1	4	0	1	1	0	2	1	0	1	0	0	1	2	2	2	0	2	1	2	0	1	1	0	0	0	2	4	0	0	0	1	0	0	0	–	–	–	–	W
Y	3	1	0	3	1	2	5	3	0	5	2	2	1	4	0	2	0	1	4	5	5	2	1	2	0	1	4	6	0	0	3	3	3	1	3	5	1	2	3	1	3	4	1	2	0	3	2	1	3	2	4	0.555	0.405	–	–	Y
total	63	67	53	73	58	61	73	79	69	59	73	62	63	66	74	60	82	68	70	74	73	56	70	73	76	55	72	66	54	56	63	78	66	63	68	66	69	74	67	61	69	57	68	72	52	56	59	52	75	47	121	0.542	0.067			total

position -1

	C50	C49	C48	C47	C46	C45	C44	C43	C42	C41	C40	C39	C38	C37	C36	C35	C34	C33	C32	C31	C30	C29	C28	C27	C26	C25	C24	C23	C22	C21	C20	C19	C18	C17	C16	C15	C14	C13	C12	C11	C10	C9	C8	C7	C6	C5	C4	C3	C2	C1	C0	C-index	SD	*P-value*	mark	
A	5	5	2	6	5	3	8	3	1	5	1	8	6	3	2	3	7	3	1	2	6	2	3	6	4	1	4	7	3	5	7	7	8	6	7	3	3	6	1	5	4	3	3	5	5	5	7	3	3	3	6	0.713	0.342	–	–	A
C	3	0	0	0	1	2	0	1	1	2	0	2	3	3	0	1	3	1	1	0	2	1	1	0	0	1	0	1	1	3	2	2	0	1	2	1	2	1	3	0	1	1	0	1	0	0	0	1	3	1	3	0.373	0.341	7.00E-03	n.s.	C
D	1	2	4	4	4	5	6	4	5	4	2	3	5	1	2	5	7	2	7	4	5	6	3	3	3	3	2	4	1	2	4	5	3	4	1	2	2	4	2	3	3	0	3	2	1	1	6	1	5	1	12	0.270	0.143	5.55E-08	✻	D
E	1	3	3	1	0	4	6	11	6	2	4	4	2	6	6	7	4	4	5	5	6	7	5	4	2	1	11	7	5	2	5	4	4	6	6	3	9	4	6	3	4	8	5	3	6	2	2	2	7	2	17	0.265	0.142	5.55E-08	✻	E
F	3	4	2	4	2	2	3	5	5	3	2	2	2	2	1	1	7	5	4	2	0	2	4	4	3	4	3	0	1	2	2	2	7	4	3	2	2	0	0	3	3	1	4	6	0	3	4	4	2	1	6	0.457	0.281	2.17E-01	n.s.	F
G	2	5	5	3	0	1	5	4	2	2	3	5	4	6	5	1	6	3	6	3	1	2	4	3	3	2	6	2	3	2	3	6	1	5	3	1	3	8	5	4	6	2	5	4	4	1	0	0	1	3	6	0.547	0.316	–	–	G
H	3	1	3	3	6	3	0	1	4	2	4	1	2	3	3	2	2	0	2	3	3	2	2	1	2	4	2	2	1	1	2	0	2	2	1	1	1	3	3	5	2	5	1	2	1	6	1	3	2	2	5	0.452	0.276	1.53E-02	n.s.	H
I	4	4	4	2	2	6	5	6	4	3	6	2	2	1	5	5	5	6	2	4	4	3	1	2	5	4	2	2	2	2	1	3	4	2	1	1	3	7	4	2	6	3	5	4	3	4	3	1	4	0	5	0.664	0.334	–	–	I
K	3	6	4	7	1	3	4	5	2	2	1	3	4	1	6	3	7	2	10	7	7	4	2	4	4	5	5	4	3	6	5	8	1	3	4	7	4	4	7	3	4	4	1	5	4	4	2	12	3	4	10	0.428	0.229	5.50E-04	n.s.	K
L	7	5	4	9	6	6	8	9	4	7	5	5	6	6	10	2	10	6	8	10	4	2	4	8	7	5	3	5	4	7	6	7	8	5	6	4	5	5	3	9	9	5	8	8	5	3	8	5	8	3	5	1.208	0.428	–	–	L
M	2	1	0	2	0	0	0	1	2	0	2	4	3	1	1	2	1	3	0	1	0	2	1	0	0	2	2	0	1	3	1	1	4	2	3	2	1	3	3	1	2	0	5	1	2	3	1	1	2	3	1	1.560	1.232	–	–	M
N	2	2	0	2	8	2	3	4	3	6	2	2	1	3	4	6	2	4	1	1	4	3	5	2	27	3	5	9	2	5	3	3	0	1	1	4	3	3	3	0	2	1	2	0	4	2	5	1	2	5	10	0.336	0.392	5.64E-08	✻	N
P	1	5	4	6	2	6	4	3	4	3	10	4	6	5	2	6	0	5	2	1	2	1	3	8	0	3	0	3	2	4	3	2	6	2	8	3	2	0	6	5	2	4	5	9	2	4	6	5	5	2	1	3.720	2.330	–	–	P
Q	1	2	3	3	4	2	3	4	7	5	5	2	3	2	2	4	3	2	3	4	4	2	13	6	5	2	5	3	2	1	5	5	5	2	1	4	5	2	4	2	4	4	5	1	2	2	5	1	2	1	3	1.127	0.683	–	–	Q
R	1	2	1	4	1	3	6	2	0	0	4	3	2	1	2	1	3	3	4	6	8	3	6	3	1	4	3	3	5	4	1	2	3	6	2	7	0	7	3	1	1	1	2	6	0	5	2	2	6	4	6	0.500	0.345	5.10E-01	n.s.	R
S	9	4	5	5	2	5	4	6	4	4	6	5	2	8	5	4	3	5	5	9	4	6	4	4	4	3	4	3	5	2	1	7	2	6	3	6	8	4	6	3	6	5	4	3	4	3	5	2	6	5	7	0.651	0.254	–	–	S
T	5	6	3	6	3	2	3	2	6	4	2	1	5	6	6	4	5	4	5	4	4	2	6	8	5	2	6	4	5	2	3	6	3	0	5	5	8	4	3	6	5	4	3	3	2	2	1	2	6	2	6	0.663	0.304	–	–	T
V	9	5	5	2	9	0	3	4	3	2	7	4	1	4	7	3	5	2	3	5	5	3	1	7	0	6	4	4	2	3	6	5	3	4	5	3	4	3	3	3	3	3	5	3	1	3	1	3	5	2	5	0.744	0.398	–	–	V
W	0	3	0	1	1	1	0	1	4	1	0	1	3	2	1	0	0	3	0	1	2	1	0	0	1	0	0	0	2	0	0	0	0	1	1	0	0	1	1	1	1	0	0	2	0	0	0	1	2	0	2	0.400	0.495	1.62E-03	n.s.	W
Y	1	2	1	3	1	5	2	3	2	2	7	1	1	2	4	0	2	5	1	2	2	2	2	0	0	0	5	3	4	0	3	3	2	1	5	7	4	5	1	2	1	3	2	4	6	3	0	2	1	3	5	0.492	0.360	1.99E-01	n.s.	Y
total	63	67	53	73	58	61	73	79	69	59	73	62	63	66	74	60	82	68	70	74	73	56	70	73	76	55	72	66	54	56	63	78	66	63	68	66	69	74	67	61	69	57	68	72	52	56	59	52	75	47	121	0.542	0.067			total

Additional file 5. Continued.

FRUIT FLY x-x-T-x-V

position -4

	C50	C49	C48	C47	C46	C45	C44	C43	C42	C41	C40	C39	C38	C37	C36	C35	C34	C33	C32	C31	C30	C29	C28	C27	C26	C25	C24	C23	C22	C21	C20	C19	C18	C17	C16	C15	C14	C13	C12	C11	C10	C9	C8	C7	C6	C5	C4	C3	C2	C1	C0	C-index	SD	*P-value*	mark	
A	2	3	6	3	6	5	5	6	1	8	6	3	2	2	4	4	2	1	2	3	2	9	3	4	4	3	2	2	4	3	3	3	3	2	3	5	5	7	5	2	2	0	6	3	1	4	3	3	2	2	4	0.870	0.466	–	–	A
C	0	1	0	0	0	1	4	0	0	1	1	1	0	0	0	2	0	1	2	0	0	0	0	0	3	1	0	1	0	1	1	1	0	0	1	0	0	0	0	2	0	1	0	0	1	0	0	0	0	0	1	0.540	0.862	3.08E-01	n.s.	C
D	2	1	6	5	3	0	4	2	0	4	6	1	2	2	4	0	3	1	2	3	3	0	2	3	4	3	1	1	2	4	3	2	1	2	2	0	2	2	1	1	3	2	3	3	2	2	4	1	1	1	5	0.448	0.290	1.11E-03	n.s.	D
E	2	0	1	3	1	3	1	2	0	4	0	2	1	0	3	0	4	3	4	2	2	3	2	2	1	1	2	1	1	3	0	3	0	2	1	3	7	0	2	3	2	2	1	1	3	3	2	1	3	1	0	–	–	–	–	E
F	2	2	1	3	3	2	1	0	2	0	2	1	1	2	3	0	2	2	1	1	0	4	5	3	1	1	3	0	3	6	5	0	2	0	1	3	3	2	0	1	1	1	3	3	2	3	2	4	3	2	1	1.960	1.414	–	–	F
G	1	0	1	2	4	6	7	2	5	4	2	3	3	2	3	4	2	6	4	6	1	2	1	4	3	6	2	2	6	8	0	1	3	1	3	10	3	4	5	3	5	4	3	0	0	1	1	3	0	3	1	3.100	2.206	–	–	G
H	1	3	1	0	1	0	3	0	1	0	1	1	0	0	3	2	4	0	1	1	1	0	0	2	0	0	1	2	0	1	0	2	0	1	0	0	3	2	0	0	0	0	0	5	0	1	0	1	2	1	2	0.480	0.597	5.19E-03	n.s.	H
I	3	3	2	2	3	6	4	3	4	4	1	4	5	5	3	0	4	2	4	4	1	1	3	24	3	1	0	1	6	4	5	4	1	1	6	7	4	6	1	4	2	2	2	2	1	3	1	2	1	2	2	1.670	1.722	–	–	I
K	1	7	2	1	4	1	2	1	2	0	3	1	0	1	2	1	5	4	3	3	2	1	0	3	1	4	0	1	2	4	2	1	2	3	1	2	2	2	5	1	0	5	0	0	4	2	3	3	4	2	7	0.303	0.227	5.63E-08	✻	K
L	4	2	5	3	3	0	5	8	7	6	7	4	4	3	6	3	3	4	4	6	7	3	1	7	6	3	7	4	4	9	6	4	6	3	7	4	3	2	3	5	5	3	4	1	3	4	1	4	3	3	6	0.707	0.324	–	–	L
M	0	1	1	0	3	2	3	2	0	1	2	3	3	1	1	1	3	0	1	0	1	2	0	1	2	2	1	1	0	0	2	0	3	1	0	0	4	2	1	1	0	3	1	1	1	0	2	0	1	0	1	1.220	1.093	–	–	M
N	2	1	4	2	4	2	2	2	3	2	2	2	2	0	2	0	2	0	3	2	0	1	1	2	0	0	5	1	1	0	2	2	2	1	2	1	2	1	2	4	0	3	2	2	2	2	1	3	1	6	7	0.260	0.184	5.55E-08	✻	N
P	3	4	1	8	2	2	6	3	4	2	3	3	5	6	2	4	1	4	4	2	5	2	4	1	4	4	6	0	0	3	2	2	1	2	5	1	3	2	7	4	0	1	2	1	3	3	3	3	2	1	10	0.292	0.181	5.55E-08	✻	P
Q	2	0	0	3	1	4	4	1	0	0	0	5	1	2	2	3	2	6	0	5	1	2	1	3	2	2	3	3	2	0	2	5	1	0	2	2	4	0	0	1	1	2	1	1	3	2	1	1	3	1	6	0.310	0.254	7.07E-08	✻	Q
R	2	6	0	2	1	2	1	2	3	3	1	3	3	2	5	3	1	4	1	6	2	2	2	0	3	4	5	2	2	6	2	2	2	1	0	1	4	2	1	3	3	4	4	2	0	3	1	2	1	1	4	0.590	0.384	7.62E-01	n.s.	R
S	7	1	3	3	2	2	7	4	1	2	0	4	3	5	1	1	3	1	6	4	2	4	6	6	3	3	1	5	5	4	2	3	4	4	2	5	1	3	3	3	3	5	1	2	3	5	2	3	4	2	4	0.795	0.422	–	–	S
T	5	2	5	4	0	2	3	2	4	1	4	4	2	5	1	2	4	4	3	4	1	3	2	2	4	7	2	5	0	5	8	6	5	2	3	6	1	3	3	3	1	2	3	1	5	2	1	1	4	5	8	0.393	0.226	8.66E-06	✻	T
V	2	3	5	1	3	2	1	2	1	4	2	6	3	2	4	4	5	3	5	9	6	3	1	3	2	4	2	1	7	7	2	9	8	6	6	6	14	19	8	4	4	4	0	3	3	1	2	4	1	2	5	0.836	0.684	–	–	V
W	0	0	0	2	0	0	0	0	0	1	0	0	0	2	0	1	0	0	1	1	0	1	1	0	0	0	0	0	0	0	0	1	0	1	0	1	0	2	1	0	0	0	0	1	0	0	0	0	0	0	0	–	–	–	–	W
Y	1	1	0	1	1	0	3	2	3	1	1	0	1	1	3	2	3	4	2	0	2	2	2	0	0	0	1	2	1	1	0	0	2	1	3	0	1	1	1	2	2	2	2	1	3	4	2	2	0	3	2	0.730	0.555	–	–	Y
total	42	41	44	48	45	42	66	44	41	48	44	51	41	43	52	37	53	50	53	62	39	45	37	70	46	49	44	35	46	69	47	51	46	34	48	57	66	62	49	47	34	46	38	33	40	45	32	41	36	38	76	0.610	0.121			total

position -3

	C50	C49	C48	C47	C46	C45	C44	C43	C42	C41	C40	C39	C38	C37	C36	C35	C34	C33	C32	C31	C30	C29	C28	C27	C26	C25	C24	C23	C22	C21	C20	C19	C18	C17	C16	C15	C14	C13	C12	C11	C10	C9	C8	C7	C6	C5	C4	C3	C2	C1	C0	C-index	SD	*P-value*	mark	
A	3	3	4	7	1	3	7	2	1	9	3	3	2	5	3	3	4	6	5	8	4	4	4	10	7	5	4	1	3	5	3	5	6	2	4	3	10	1	3	5	5	5	4	6	1	5	2	3	7	3	4	1.060	0.555	–	–	A
C	1	1	0	1	0	3	1	0	1	0	2	3	0	1	0	0	0	0	0	3	0	0	2	0	0	0	1	0	1	2	0	0	1	0	1	0	3	1	1	0	1	2	0	1	0	0	0	1	1	0	2	0.360	0.463	1.47E-05	✻	C
D	2	1	1	2	1	4	1	2	4	3	1	1	1	1	4	3	2	1	3	0	2	4	1	5	4	2	1	1	0	3	4	2	1	0	2	1	3	2	3	2	0	0	3	1	3	4	6	1	2	1	3	0.680	0.471	–	–	D
E	2	1	0	2	3	2	1	2	2	5	4	2	0	0	2	3	3	4	2	3	2	3	3	1	3	3	0	2	1	4	3	0	0	2	2	2	4	5	3	2	3	2	0	1	2	1	1	1	5	1	10	0.210	0.136	5.55E-08	✻	E
F	0	3	0	2	2	1	3	1	2	1	3	3	0	1	1	0	3	4	3	1	3	0	2	3	2	1	3	0	0	3	3	1	1	4	6	9	1	5	0	1	1	1	2	0	4	1	1	1	2	1	3	0.640	0.583	–	–	F
G	6	0	2	4	1	1	5	6	4	2	2	2	2	6	3	0	3	1	3	3	1	3	3	1	2	0	5	1	4	5	4	1	3	2	2	0	2	1	3	4	1	3	3	0	2	0	0	1	0	0	9	0.251	0.193	5.55E-08	✻	G
H	0	1	1	1	2	0	0	0	2	2	1	0	2	3	2	1	2	1	0	1	0	0	0	1	0	3	0	0	0	3	1	0	1	0	2	1	2	0	3	1	3	1	0	0	2	3	1	0	0	1	0	–	–	–	–	H
I	2	1	3	4	3	2	4	3	1	1	4	3	2	2	5	0	5	0	5	3	2	2	2	1	2	1	2	3	6	1	1	5	0	3	3	1	2	6	2	8	3	2	4	1	4	2	2	3	0	2	3	0.860	0.568	–	–	I
K	2	0	2	1	4	1	6	2	2	0	2	6	2	2	4	3	3	3	3	2	1	5	1	2	2	2	2	3	1	4	1	0	1	2	0	3	2	2	2	3	0	0	2	5	1	2	3	0	1	2	3	0.700	0.491	–	–	K
L	3	4	3	6	2	5	4	4	5	4	2	0	5	5	5	2	1	5	2	5	3	4	1	1	0	2	3	0	7	5	5	6	4	2	3	4	5	3	3	3	3	6	3	3	2	6	1	1	3	5	1	3.380	1.748	–	–	L
M	1	0	3	2	2	1	0	2	1	0	0	0	0	1	1	1	2	0	1	1	0	0	0	1	1	0	1	3	0	1	0	1	1	0	0	1	0	1	0	0	1	3	0	0	0	1	0	0	0	1	2	0.360	0.429	6.49E-06	✻	M
N	2	1	2	1	4	1	4	3	1	4	2	4	1	1	1	1	2	1	2	7	2	2	0	0	2	3	2	2	0	3	2	3	3	1	2	2	1	1	1	2	0	2	1	1	1	0	1	2	3	2	3	0.613	0.433	–	–	N
P	3	4	2	3	4	0	5	5	4	1	4	4	3	0	5	1	4	3	4	5	3	3	2	4	3	3	4	4	1	4	3	5	1	2	1	2	3	0	2	2	1	2	0	3	4	2	0	3	2	1	5	0.536	0.298	9.64E-01	n.s.	P
Q	2	1	2	2	3	3	4	1	3	3	1	2	4	1	3	0	2	3	2	3	2	0	1	26	0	2	2	2	4	2	3	3	1	0	0	3	6	4	1	1	2	6	3	2	1	5	1	3	1	0	2	1.320	1.834	–	–	Q
R	2	1	2	1	1	2	3	0	1	2	0	1	0	2	4	3	5	3	2	1	4	2	3	2	4	3	3	2	4	6	1	4	1	4	2	2	3	2	6	3	4	0	1	2	3	2	1	3	1	3	1	2.340	1.437	–	–	R
S	4	6	3	2	3	6	8	2	2	6	5	4	5	3	3	5	2	3	5	2	6	5	5	6	3	5	2	0	2	6	3	4	4	3	4	8	4	1	3	4	1	7	7	2	2	4	1	4	3	4	5	0.768	0.371	–	–	S
T	3	5	4	3	6	3	2	3	3	2	1	5	3	6	3	1	5	5	5	6	1	2	3	2	4	3	3	5	4	1	2	3	6	2	4	5	1	7	3	4	2	0	3	1	0	1	5	6	1	6	7	0.469	0.258	1.57E-02	n.s.	T
V	1	4	5	1	2	3	6	4	2	3	3	2	7	1	2	6	2	2	3	4	2	4	1	1	5	2	4	5	4	4	8	3	4	1	3	1	4	2	7	2	3	2	0	2	3	2	4	1	3	3	10	0.306	0.175	5.55E-08	✻	V
W	0	0	0	2	0	1	1	1	0	0	2	1	0	0	1	1	0	0	1	1	0	0	2	0	0	4	1	0	1	1	0	0	0	0	1	1	0	2	0	0	0	0	0	0	1	1	2	5	0	1	0	–	–	–	–	W
Y	3	4	5	1	1	0	1	1	0	0	2	5	2	2	0	3	3	5	2	3	1	2	1	3	2	5	1	1	3	6	0	5	7	4	6	8	10	16	3	0	0	2	2	2	4	3	0	2	1	1	3	0.960	0.977	–	–	Y
total	42	41	44	48	45	42	66	44	41	48	44	51	41	43	52	37	53	50	53	62	39	45	37	70	46	49	44	35	46	69	47	51	46	34	48	57	66	62	49	47	34	46	38	33	40	45	32	41	36	38	76	0.610	0.121			total

position -1

	C50	C49	C48	C47	C46	C45	C44	C43	C42	C41	C40	C39	C38	C37	C36	C35	C34	C33	C32	C31	C30	C29	C28	C27	C26	C25	C24	C23	C22	C21	C20	C19	C18	C17	C16	C15	C14	C13	C12	C11	C10	C9	C8	C7	C6	C5	C4	C3	C2	C1	C0	C-index	SD	*P-value*	mark	
A	3	5	7	9	1	5	9	4	6	5	3	2	3	6	3	2	1	1	3	4	3	2	3	26	2	1	2	5	3	3	2	4	4	5	5	4	6	1	0	4	1	2	5	2	3	2	1	2	1	3	8	0.473	0.472	1.42E-04	n.s.	A
C	2	0	0	0	1	1	1	0	2	2	1	1	1	1	1	1	2	1	2	0	1	0	0	0	0	1	0	0	2	1	1	0	3	0	0	1	0	0	0	0	0	1	1	0	0	2	2	0	0	0	5	0.144	0.162	5.55E-08	✻	C
D	2	3	2	2	3	0	4	2	4	1	2	1	3	2	3	2	6	2	2	3	2	1	3	5	3	5	8	1	2	3	0	1	3	0	3	1	5	5	5	2	3	3	0	1	3	3	0	3	3	2	4	0.640	0.411	–	–	D
E	2	5	2	2	1	1	3	2	3	1	2	5	3	3	4	5	1	3	2	2	2	4	1	2	3	5	4	3	1	2	3	3	2	2	1	5	4	1	5	3	0	5	2	3	3	1	3	3	1	2	10	0.262	0.134	5.55E-08	✻	E
F	1	0	1	3	2	1	5	2	0	1	0	2	2	2	3	1	4	0	2	2	1	3	2	1	1	1	3	1	1	3	4	1	0	1	1	2	2	1	6	2	2	4	4	2	3	1	2	5	0	1	1	1.900	1.403	–	–	F
G	3	1	2	2	4	3	2	8	1	4	2	3	2	1	4	0	2	7	4	4	4	3	2	2	2	4	5	4	1	2	2	1	5	0	0	3	4	2	1	1	2	6	2	3	4	1	3	0	1	4	0	–	–	–	–	G
H	1	1	2	2	2	1	0	0	0	1	1	1	1	2	2	2	1	2	1	3	0	0	0	0	1	1	0	0	0	0	0	1	3	2	1	1	2	1	1	1	0	1	2	0	1	0	2	0	0	1	2	0.480	0.428	4.24E-02	n.s.	H
I	1	0	1	1	5	3	2	2	3	3	4	4	3	1	2	1	4	5	5	6	3	3	4	4	8	0	2	4	1	7	7	0	0	2	3	3	8	2	6	4	8	1	2	6	0	0	0	2	4	4	4	0.770	0.569	–	–	I
K	3	2	3	1	6	0	5	1	2	0	1	1	1	5	1	2	1	1	4	3	1	2	3	3	2	1	2	1	4	5	2	2	5	3	4	6	4	3	2	5	2	1	1	3	2	4	3	4	1	2	3	0.840	0.518	–	–	K
L	3	6	4	3	3	4	7	2	3	3	2	4	3	2	3	4	2	2	8	4	3	4	4	2	2	1	3	1	4	7	5	2	1	5	2	1	2	6	4	4	1	1	2	2	3	2	3	2	4	1	2	1.560	0.831	–	–	L
M	2	1	2	0	0	1	0	0	1	0	1	2	0	0	2	1	0	2	0	0	0	0	0	4	3	1	1	0	2	4	2	0	2	0	1	0	1	0	2	2	2	1	1	0	2	0	0	2	0	2	0	–	–	–	–	M
N	1	5	1	3	2	2	3	2	2	3	6	1	1	2	3	3	1	3	4	1	2	1	2	4	4	4	0	3	4	2	1	2	6	3	4	1	5	6	1	8	0	3	1	1	2	5	3	2	2	1	7	0.377	0.246	4.22E-07	✻	N
P	4	1	2	3	3	5	1	4	2	3	7	3	2	4	4	2	5	0	4	5	2	2	2	3	2	3	2	1	1	2	3	2	0	5	5	2	2	3	3	1	2	1	5	0	1	3	1	1	3	2	3	0.860	0.509	–	–	P
Q	2	0	2	4	0	2	3	0	2	4	1	9	0	0	0	6	4	4	1	3	2	2	0	3	3	2	0	0	4	1	2	5	0	0	3	2	2	2	1	0	0	4	2	1	1	3	0	3	0	2	2	0.970	0.934	–	–	Q
R	2	0	1	2	2	2	3	3	2	3	1	4	1	0	2	0	6	3	2	2	3	2	0	1	0	3	1	1	1	4	1	7	2	3	5	11	5	10	4	1	2	2	2	2	1	4	0	3	0	1	5	0.492	0.458	1.40E-03	n.s.	R
S	5	4	3	3	2	3	2	6	2	4	3	4	6	5	5	1	1	1	3	6	1	2	3	1	4	3	1	3	4	7	4	2	3	0	1	5	6	3	4	2	5	5	3	2	2	4	2	4	3	4	8	0.405	0.203	4.24E-06	✻	S
T	2	4	3	4	1	4	4	2	2	4	3	0	5	0	5	0	3	5	2	7	4	5	3	1	2	7	2	2	5	4	3	2	3	0	4	2	2	7	2	4	1	1	0	2	2	5	2	4	6	3	9	0.333	0.205	5.62E-08	✻	T
V	3	1	3	2	5	3	8	3	2	3	2	0	4	6	5	3	6	3	2	6	4	6	4	3	3	3	5	4	3	6	4	3	2	1	2	5	5	4	1	3	1	3	1	1	6	1	3	1	4	3	1	3.300	1.741	–	–	V
W	0	0	0	0	1	0	0	0	0	0	0	2	0	0	0	0	0	2	2	0	0	1	0	2	0	0	0	0	1	2	1	2	0	1	1	0	0	1	0	0	0	0	0	1	1	0	0	0	0	0	0	–	–	–	–	W
Y	0	2	3	2	1	1	4	1	2	3	2	2	0	1	0	1	3	3	0	1	1	2	1	3	1	3	3	1	2	4	0	11	2	1	2	2	1	4	1	0	2	1	2	1	0	4	2	0	3	0	2	0.920	0.888	–	–	Y
total	42	41	44	48	45	42	66	44	41	48	44	51	41	43	52	37	53	50	53	62	39	45	37	70	46	49	44	35	46	69	47	51	46	34	48	57	66	62	49	47	34	46	38	33	40	45	32	41	36	38	76	0.610	0.121			total

Additional file 5. Continued.

FRUIT FLY x-x-V-x-I

position -4

	C50	C49	C48	C47	C46	C45	C44	C43	C42	C41	C40	C39	C38	C37	C36	C35	C34	C33	C32	C31	C30	C29	C28	C27	C26	C25	C24	C23	C22	C21	C20	C19	C18	C17	C16	C15	C14	C13	C12	C11	C10	C9	C8	C7	C6	C5	C4	C3	C2	C1	C0	C-index	SD	*P-value*	mark	
A	3	4	4	0	7	4	2	4	4	3	3	7	7	1	3	4	1	2	1	9	2	3	2	3	3	4	2	4	1	2	6	0	0	4	8	3	2	1	3	1	4	5	3	2	5	5	2	3	3	2	6	0.537	0.336	5.55E-08	*	A
C	0	0	0	1	2	0	1	1	1	0	0	0	0	0	0	0	2	0	1	0	3	3	3	1	1	1	0	2	1	3	2	1	1	1	0	0	1	0	1	1	1	1	0	1	1	1	1	1	0	0	2	0.420	0.444	5.56E-08	*	C
D	2	2	3	1	0	3	5	4	3	1	3	5	4	1	0	4	2	2	2	3	2	1	2	2	1	4	2	2	3	1	2	2	3	3	1	2	1	5	1	2	3	2	1	3	2	2	1	6	2	1	1	2.300	1.313	–	–	D
E	2	3	5	1	4	2	5	3	6	4	4	2	2	2	4	0	4	3	2	4	4	2	2	4	0	5	0	6	1	3	3	5	1	3	7	6	1	3	6	1	5	5	1	0	1	2	3	0	4	3	2	1.490	0.923	–	–	E
F	3	2	1	2	3	2	4	2	0	1	3	0	2	3	2	3	3	0	2	2	3	1	2	0	0	1	0	1	1	1	1	3	2	4	2	4	1	3	2	2	1	0	2	0	2	4	3	0	2	3	0	–	–	–	–	F
G	4	5	4	6	5	2	7	5	4	2	4	6	3	2	2	2	2	4	3	1	2	3	3	5	2	1	4	2	9	3	12	2	2	5	4	1	2	4	2	1	0	3	2	4	5	3	4	2	0	5	2	1.700	1.083	–	–	G
H	1	1	1	1	0	0	0	1	0	2	2	0	2	0	3	1	1	3	2	1	1	1	0	2	0	3	2	1	0	2	1	1	2	0	0	0	1	1	0	0	1	0	2	0	1	0	2	1	0	1	2	0.480	0.451	5.58E-08	*	H
I	6	0	4	3	1	3	5	3	2	1	4	1	1	1	3	3	2	1	6	2	3	2	1	3	4	1	4	2	1	2	2	2	3	4	2	3	2	6	0	2	1	0	1	5	1	3	1	5	3	1	2	1.220	0.790	–	–	I
K	3	3	3	5	3	1	3	1	1	1	5	2	1	3	6	1	0	3	2	0	3	4	1	3	3	2	4	2	3	2	4	3	1	2	25	4	1	2	0	3	0	4	0	8	1	4	5	0	0	3	3	0.960	1.210	2.40E-02	n.s.	K
L	2	2	3	4	7	5	5	7	6	3	4	3	2	7	2	4	2	4	6	7	4	2	4	2	5	5	5	6	2	2	4	5	2	5	5	5	2	2	8	3	3	3	4	5	7	9	3	1	4	2	2	2.040	0.947	–	–	L
M	0	0	2	0	1	1	0	0	2	2	0	3	0	0	1	2	3	1	1	0	0	0	0	1	3	1	1	0	0	0	0	3	1	0	1	0	2	1	2	1	0	0	0	0	2	1	1	2	2	1	1	0.900	0.974	7.84E-02	n.s.	M
N	6	5	0	2	1	2	0	1	0	0	2	1	0	3	2	2	2	1	3	3	0	1	5	2	0	1	3	1	1	4	1	3	1	3	1	3	1	0	1	5	2	1	1	1	1	4	0	3	1	1	2	0.880	0.753	2.69E-02	n.s.	N
P	2	1	1	3	0	2	3	0	1	0	2	3	3	2	0	1	1	1	1	0	3	4	1	2	1	4	4	1	1	1	3	0	3	3	1	0	3	2	6	1	0	2	0	3	0	0	3	1	1	3	1	1.680	1.392	–	–	P
Q	2	0	1	3	2	2	0	3	0	2	3	2	1	1	1	3	4	5	2	1	2	2	2	1	0	1	3	2	0	1	1	5	2	2	2	1	0	2	2	1	2	1	0	0	4	1	5	1	0	2	3	0.573	0.442	6.28E-08	*	Q
R	2	6	2	5	0	3	4	4	2	4	1	2	2	3	3	5	3	1	2	4	2	2	6	1	6	4	6	0	3	4	2	3	4	0	3	3	0	2	3	3	3	2	1	1	4	0	2	2	3	2	1	2.700	1.607	–	–	R
S	2	4	2	3	1	17	4	10	4	2	1	1	1	1	5	3	2	6	2	2	2	6	1	3	0	4	0	3	23	4	1	2	1	2	0	1	4	1	2	2	3	3	3	2	1	3	4	2	4	4	3	1.093	1.309	–	–	S
T	3	1	3	4	2	2	2	2	3	2	2	3	1	2	1	9	4	2	3	6	2	5	2	1	4	1	1	1	1	1	2	5	4	2	0	1	1	3	5	5	2	0	1	4	1	2	1	1	3	3	2	1.220	0.852	–	–	T
V	3	3	4	2	1	1	0	4	2	2	1	3	2	1	3	4	2	3	1	2	3	3	8	3	2	1	4	3	1	3	1	3	3	0	1	2	1	3	4	6	2	1	1	4	4	4	2	1	4	1	1	2.460	1.515	–	–	V
W	1	1	0	0	0	0	0	0	0	0	0	1	1	0	0	0	0	0	0	0	2	1	1	0	0	2	0	0	0	0	0	0	1	0	0	0	0	0	1	1	0	0	0	0	0	0	0	1	0	1	0	–	–	–	–	W
Y	1	1	2	0	1	0	2	3	0	1	3	1	1	1	2	1	7	0	1	1	2	2	2	0	0	1	2	0	1	0	3	2	3	0	0	1	0	1	0	3	0	3	3	1	0	1	0	0	1	2	4	0.315	0.330	5.55E-08	*	Y
total	48	44	45	46	41	52	52	58	41	33	47	46	36	34	43	52	47	42	43	48	45	48	48	39	35	47	47	39	53	39	51	50	40	43	63	40	26	42	49	44	33	36	26	44	43	49	43	33	37	41	40	1.086	0.181			total

position -3

	C50	C49	C48	C47	C46	C45	C44	C43	C42	C41	C40	C39	C38	C37	C36	C35	C34	C33	C32	C31	C30	C29	C28	C27	C26	C25	C24	C23	C22	C21	C20	C19	C18	C17	C16	C15	C14	C13	C12	C11	C10	C9	C8	C7	C6	C5	C4	C3	C2	C1	C0	C-index	SD	*P-value*	mark	
A	3	2	4	2	4	4	4	6	2	2	4	4	1	3	5	5	2	4	3	1	2	3	3	1	3	4	4	4	2	3	12	4	1	7	4	2	3	1	1	2	3	5	0	3	1	4	5	2	2	3	2	1.590	0.962	–	–	A
C	0	2	1	1	0	1	0	1	1	3	0	0	1	0	2	1	2	1	1	2	1	1	3	1	1	1	3	0	2	1	1	1	0	0	3	3	1	0	4	2	1	0	1	1	0	1	2	3	0	0	1	1.160	1.037	–	–	C
D	5	1	1	5	3	2	4	2	3	1	1	4	3	0	6	0	0	1	2	5	3	2	1	4	3	5	1	4	3	4	6	3	0	1	2	0	3	1	3	4	6	3	1	1	1	4	4	5	2	1	0	–	–	–	–	D
E	2	1	0	2	1	3	1	4	2	2	2	0	2	3	2	2	3	0	1	3	4	1	3	4	1	1	3	3	4	2	0	3	3	2	1	3	1	3	2	2	1	5	3	2	3	5	0	2	2	5	2	1.100	0.655	–	–	E
F	1	2	0	1	0	4	3	2	1	2	2	1	1	3	1	2	2	0	3	3	3	5	2	1	1	3	7	3	3	0	2	1	1	2	0	3	0	3	1	1	1	1	0	1	4	2	2	1	3	0	1	1.820	1.424	–	–	F
G	3	3	3	2	3	3	3	4	3	1	4	4	4	2	3	3	2	3	0	5	0	2	5	2	4	4	0	1	20	5	3	3	1	3	3	3	0	0	4	5	0	1	2	4	3	2	1	0	2	2	2	1.430	1.436	–	–	G
H	1	0	0	3	2	1	0	1	4	2	0	1	0	1	0	1	2	0	1	1	1	0	1	1	1	1	2	1	0	0	2	1	0	2	2	1	3	2	3	1	1	1	1	0	1	2	0	0	1	0	0	–	–	–	–	H
I	3	7	4	1	1	0	3	2	2	3	1	3	3	3	3	1	3	1	3	2	3	3	6	2	4	1	4	3	1	1	2	7	1	2	1	2	1	3	5	0	1	4	2	2	1	6	1	3	3	4	5	0.512	0.329	5.55E-08	*	I
K	1	1	3	2	1	1	4	3	3	2	2	2	1	1	2	7	3	0	2	1	2	1	1	0	2	4	3	2	2	1	3	0	0	2	5	1	2	4	1	0	2	2	2	4	3	3	2	2	1	2	2	1.010	0.681	2.78E-01	n.s.	K
L	5	4	6	3	0	4	7	4	2	5	8	4	4	5	4	8	5	6	7	4	4	2	3	4	4	9	7	2	3	6	5	8	5	6	5	5	2	2	3	4	7	1	3	2	6	2	5	3	5	2	4	1.100	0.497	–	–	L
M	0	3	0	2	1	0	0	3	0	0	1	2	3	1	0	0	1	2	0	0	2	1	1	0	0	0	0	1	1	0	2	0	1	3	0	0	0	0	0	0	0	1	1	2	0	1	0	0	0	1	0	–	–	–	–	M
N	1	2	3	1	4	1	1	4	2	1	3	3	2	0	3	4	3	1	4	2	3	8	4	3	1	1	2	3	2	4	2	3	3	1	2	0	1	1	3	1	0	1	2	2	3	2	2	3	4	5	2	1.170	0.733	–	–	N
P	5	4	4	3	3	2	3	1	2	1	4	3	3	4	1	2	1	3	3	1	2	3	0	1	1	1	3	2	2	2	1	0	3	1	2	1	2	0	2	3	1	1	0	3	2	4	5	3	2	3	1	2.180	1.273	–	–	P
Q	2	0	2	3	3	2	3	4	4	0	4	1	1	2	1	4	4	4	2	4	2	0	3	0	2	0	1	0	1	1	2	0	1	2	0	1	1	4	4	1	1	2	0	0	2	2	1	1	0	1	4	0.430	0.346	5.55E-08	*	Q
R	4	2	3	2	3	2	3	1	3	2	3	5	2	0	4	5	1	2	2	4	5	1	4	1	2	4	0	1	0	1	1	2	2	1	27	3	0	4	1	4	3	1	1	2	1	2	2	0	1	2	3	0.880	1.259	7.03E-04	n.s.	R
S	4	4	5	2	5	2	2	2	3	0	2	4	1	2	3	2	6	5	4	2	3	1	2	1	2	2	3	5	2	1	4	2	7	0	2	3	2	4	6	2	2	1	1	6	2	1	3	0	2	2	2	1.340	0.830	–	–	S
T	1	1	2	5	2	14	4	5	1	3	1	2	2	0	2	0	4	2	1	4	2	6	5	6	1	0	1	2	0	4	2	4	4	0	1	3	2	2	2	4	1	2	3	0	4	4	3	2	2	4	4	0.660	0.575	2.22E-07	*	T
V	6	3	1	3	3	3	3	5	1	2	3	3	1	2	0	3	1	6	1	3	1	4	0	3	0	5	1	1	2	3	0	6	5	5	2	3	1	5	2	4	0	1	2	4	5	2	2	1	3	3	4	0.645	0.426	1.23E-06	*	V
W	0	2	1	1	0	0	1	1	1	1	1	0	0	1	0	0	1	1	2	0	1	1	0	1	1	0	0	0	1	0	0	0	0	0	0	1	1	2	0	1	1	1	0	1	1	0	0	0	1	0	0	–	–	–	–	W
Y	1	0	2	2	2	3	3	3	1	0	1	0	1	1	1	2	1	0	1	1	1	3	1	3	1	1	2	1	2	0	1	2	2	3	1	2	0	1	2	3	1	2	1	4	0	0	3	2	1	1	1	1.460	1.014	–	–	Y
total	48	44	45	46	41	52	52	58	41	33	47	46	36	34	43	52	47	42	43	48	45	48	48	39	35	47	47	39	53	39	51	50	40	43	63	40	26	42	49	44	33	36	26	44	43	49	43	33	37	41	40	1.086	0.181			total

position -1

	C50	C49	C48	C47	C46	C45	C44	C43	C42	C41	C40	C39	C38	C37	C36	C35	C34	C33	C32	C31	C30	C29	C28	C27	C26	C25	C24	C23	C22	C21	C20	C19	C18	C17	C16	C15	C14	C13	C12	C11	C10	C9	C8	C7	C6	C5	C4	C3	C2	C1	C0	C-index	SD	*P-value*	mark	
A	4	3	0	2	3	2	4	5	5	2	3	0	1	1	8	3	3	3	4	4	6	3	2	4	3	4	5	2	4	2	3	7	2	7	3	3	1	6	3	3	4	1	1	4	4	5	7	0	2	1	4	0.810	0.470	2.92E-04	n.s.	A
C	1	1	3	1	0	3	2	1	3	3	1	1	0	0	0	0	3	2	2	0	2	0	1	1	1	1	1	0	0	0	1	1	0	1	0	0	0	0	3	1	0	1	0	1	2	0	0	3	1	1	0	–	–	–	–	C
D	0	3	1	3	3	2	3	2	2	0	4	3	1	2	1	2	3	1	0	1	1	2	6	3	1	2	2	1	2	1	6	2	5	4	0	2	0	1	3	6	3	1	0	2	7	2	3	2	5	1	7	0.323	0.243	5.55E-08	*	D
E	2	1	2	1	4	2	2	2	4	2	1	1	3	1	2	1	4	0	0	3	3	7	4	1	3	2	4	5	1	1	4	4	3	2	2	3	1	1	1	3	1	3	1	2	3	4	1	3	1	3	4	0.575	0.351	5.55E-08	*	E
F	1	1	3	1	5	0	3	5	0	3	3	1	5	1	3	1	2	3	0	2	1	3	3	3	2	2	3	2	5	2	3	1	4	5	3	2	2	2	1	2	3	3	1	4	2	0	3	1	1	1	1	2.260	1.382	–	–	F
G	5	7	6	2	1	1	4	6	4	2	7	5	3	12	6	11	5	10	3	4	7	3	8	1	1	2	5	3	0	2	3	3	3	2	3	5	2	4	4	2	3	1	2	3	0	3	2	0	3	0	2	1.840	1.357	–	–	G
H	5	2	3	3	2	2	3	1	1	0	3	1	1	1	1	1	0	1	0	0	2	1	0	0	1	3	4	1	0	3	1	1	2	1	1	0	1	1	0	1	0	0	2	0	0	1	2	1	1	0	2	0.620	0.585	2.16E-06	*	H
I	6	0	4	1	3	1	1	3	0	2	0	6	1	3	4	3	1	1	2	2	1	1	1	0	5	3	0	1	4	1	1	2	1	1	2	1	1	2	2	1	2	3	2	2	1	5	0	3	1	2	3	0.640	0.503	8.12E-07	*	I
K	4	1	4	4	2	18	4	4	2	2	4	3	2	1	1	2	1	0	1	4	3	3	3	1	1	2	2	4	1	1	2	4	2	3	4	3	2	2	3	3	5	2	0	3	1	4	2	2	4	5	1	2.820	2.537	–	–	K
L	1	3	2	5	4	4	8	5	4	4	3	4	1	3	2	5	3	3	4	4	6	6	4	2	4	4	3	3	5	5	3	5	8	3	5	3	1	3	2	3	3	1	4	5	4	0	1	0	4	3	5	0.700	0.341	5.67E-08	*	L
M	0	3	2	1	0	0	0	0	3	2	0	1	1	0	0	3	0	1	0	0	0	0	1	0	0	0	0	0	0	0	0	3	1	0	1	2	2	3	2	0	1	0	0	0	1	0	0	0	0	0	0	–	–	–	–	M
N	2	2	1	1	1	0	2	2	1	3	2	4	3	1	2	2	2	1	4	2	2	2	0	1	1	3	0	0	2	1	1	0	2	0	2	0	0	1	3	2	0	1	3	2	0	2	2	4	1	3	1	1.580	1.126	–	–	N
P	4	6	2	2	4	1	4	5	2	1	2	6	3	2	0	1	2	0	5	4	1	1	2	3	0	3	1	3	2	2	1	3	0	2	1	1	1	3	2	3	2	2	1	3	5	2	1	1	1	1	2	1.100	0.756	–	–	P
Q	1	1	2	0	0	4	1	3	3	2	2	0	2	2	0	3	3	4	1	4	2	1	0	5	3	1	3	1	0	0	6	0	1	1	0	1	2	2	7	2	0	2	0	0	0	3	5	1	0	2	3	0.593	0.569	8.48E-07	*	Q
R	0	2	2	4	1	1	2	2	2	0	2	2	2	2	4	0	1	3	3	2	2	4	2	2	1	3	1	2	1	0	1	2	0	2	2	2	2	3	3	1	1	2	1	1	2	4	0	4	3	5	2	0.960	0.596	1.14E-01	n.s.	R
S	4	2	2	7	0	2	4	4	2	4	2	2	3	0	2	6	3	4	2	3	2	2	4	2	5	5	5	4	3	4	5	3	0	5	1	6	1	1	4	3	2	1	1	3	1	7	9	1	3	6	2	1.570	0.985	–	–	S
T	1	0	2	2	4	5	3	3	2	0	2	2	0	1	3	5	3	0	7	4	3	3	2	4	1	3	2	4	21	5	3	2	5	1	28	3	1	3	2	7	1	5	4	5	3	2	1	4	2	1	0	–	–	–	–	T
V	3	1	3	3	3	2	0	4	1	0	2	3	2	0	3	2	1	1	4	3	1	3	5	1	1	1	2	2	1	3	7	4	0	3	1	3	4	1	3	1	2	3	1	3	5	4	2	1	3	6	0	–	–	–	–	V
W	0	2	0	0	1	0	0	1	0	0	0	1	0	1	0	0	6	0	0	0	0	0	0	3	1	0	0	0	0	2	0	2	0	0	1	0	0	1	0	0	0	0	1	0	1	0	0	0	0	0	0	–	–	–	–	W
Y	4	3	1	3	0	2	2	0	0	1	4	0	2	0	1	1	1	4	1	2	0	3	0	2	0	3	4	1	1	4	0	1	1	0	3	0	2	2	1	0	0	4	1	1	1	1	2	2	1	0	1	1.460	1.328	–	–	Y
total	48	44	45	46	41	52	52	58	41	33	47	46	36	34	43	52	47	42	43	48	45	48	48	39	35	47	47	39	53	39	51	50	40	43	63	40	26	42	49	44	33	36	26	44	43	49	43	33	37	41	40	1.086	0.181			total

Additional file 5. Continued.

FRUIT FLY x-x-V-x-V

position -4

	C50	C49	C48	C47	C46	C45	C44	C43	C42	C41	C40	C39	C38	C37	C36	C35	C34	C33	C32	C31	C30	C29	C28	C27	C26	C25	C24	C23	C22	C21	C20	C19	C18	C17	C16	C15	C14	C13	C12	C11	C10	C9	C8	C7	C6	C5	C4	C3	C2	C1	C0	C-index	SD	*P-value*	mark	
A	1	1	2	5	3	6	3	2	2	3	2	9	5	1	3	6	11	6	7	5	1	3	3	7	5	3	0	2	3	5	4	3	6	4	4	8	5	7	8	4	6	10	4	1	4	7	2	2	3	3	6	0.700	0.414	1.01E-03	n.s.	A
C	2	1	2	0	2	0	1	0	2	2	0	2	5	2	0	2	1	2	1	2	1	0	0	1	1	0	3	0	3	1	0	0	2	0	1	0	2	0	1	0	2	2	0	1	0	2	1	0	0	1	0	–	–	–	–	C
D	4	6	2	3	1	4	1	5	5	3	3	2	5	6	3	4	2	4	1	3	3	2	2	2	6	5	1	2	5	1	5	5	0	2	3	0	3	2	3	2	1	2	2	2	5	2	2	5	5	5	3	1.013	0.547	–	–	D
E	4	3	4	2	7	4	5	3	3	4	6	2	5	6	2	2	2	3	5	2	5	4	5	0	1	5	2	4	2	3	1	4	1	0	2	4	3	2	2	4	6	1	6	2	1	1	1	3	0	1	5	0.600	0.357	1.54E-06	*	E
F	2	2	2	0	1	3	3	1	0	0	2	0	2	2	3	4	3	3	3	1	3	2	1	0	1	0	5	1	1	2	1	3	6	4	1	3	4	1	2	2	4	4	3	2	1	2	3	1	1	0	0	–	–	–	–	F
G	3	4	1	9	3	4	2	4	2	2	8	4	3	4	7	1	0	2	11	5	4	7	7	3	5	4	1	4	10	6	1	2	1	6	6	11	3	4	2	0	6	0	4	2	3	1	1	4	2	4	2	1.930	1.370	–	–	G
H	1	2	2	4	0	2	1	1	0	3	0	3	3	1	2	2	5	1	1	0	1	2	1	1	0	1	0	1	1	1	0	3	1	2	1	1	4	1	0	2	1	4	1	2	1	1	2	1	0	1	3	0.480	0.394	6.29E-08	*	H
I	3	1	2	2	2	3	2	5	5	3	3	3	3	2	4	1	3	3	4	3	1	1	4	3	5	4	2	1	1	2	4	2	5	2	0	2	0	4	0	1	3	3	2	3	2	2	2	1	5	4	3	0.853	0.453	8.79E-01	n.s.	I
K	5	4	0	4	1	4	6	2	1	3	2	3	1	1	4	3	5	5	3	4	1	7	4	5	4	4	1	4	3	4	1	4	4	5	6	1	0	3	6	2	1	3	2	2	3	5	7	1	2	7	6	0.543	0.312	5.77E-08	*	K
L	4	1	10	7	4	4	2	4	0	7	5	3	6	7	2	2	3	3	5	4	2	4	4	5	1	2	3	5	1	1	2	4	5	3	4	6	10	1	2	3	3	1	5	7	3	7	4	3	1	2	1	3.740	2.248	–	–	L
M	1	0	0	0	2	1	2	1	1	1	2	3	2	3	1	7	0	0	1	1	1	2	0	1	1	1	2	1	0	1	0	0	0	2	1	1	0	1	1	0	1	0	1	1	0	1	1	0	3	1	5	0.216	0.238	5.55E-08	*	M
N	1	3	1	2	4	2	1	3	1	1	9	3	3	4	3	2	2	0	2	3	4	5	1	4	4	1	2	2	2	4	7	3	3	1	2	1	2	0	1	3	1	4	0	4	1	1	1	0	3	2	5	0.476	0.347	5.55E-08	*	N
P	4	2	4	7	2	6	2	5	5	4	5	5	1	3	4	5	3	4	4	3	0	3	5	0	1	3	2	8	3	1	3	4	3	3	5	3	3	2	7	1	1	3	1	4	4	4	1	3	2	4	3	1.100	0.588	–	–	P
Q	1	1	3	7	2	1	3	1	5	3	6	3	2	4	4	4	5	6	6	1	5	1	4	5	0	4	1	3	1	0	5	3	4	2	3	5	3	2	1	0	3	3	1	3	4	2	7	2	1	0	2	1.460	0.947	–	–	Q
R	1	5	4	8	4	1	1	2	2	1	6	4	1	4	5	9	5	4	3	1	5	2	4	2	4	2	6	3	1	4	0	2	3	1	6	4	3	2	7	5	5	3	0	6	1	1	4	1	6	5	4	0.845	0.532	9.50E-01	n.s.	R
S	8	3	2	3	3	6	6	3	4	7	4	1	4	3	5	4	7	2	3	4	4	4	1	3	4	1	2	4	3	2	7	5	5	3	6	3	7	5	3	10	5	2	2	7	6	3	5	5	6	4	2	2.090	0.973	–	–	S
T	3	2	5	2	3	1	2	3	7	2	6	3	3	5	4	2	3	3	1	3	6	4	1	4	2	4	0	0	4	2	3	3	4	2	1	2	3	2	3	2	5	1	0	3	3	7	1	4	4	1	1	2.880	1.662	–	–	T
V	0	5	0	6	3	8	3	7	4	8	6	8	13	12	7	8	4	3	8	2	7	2	5	2	8	6	2	7	4	5	2	3	6	7	5	6	5	10	6	3	3	5	7	4	4	7	2	1	3	1	4	1.265	0.710	–	–	V
W	1	0	0	0	1	0	1	0	1	0	0	0	0	0	0	0	0	0	1	0	0	0	0	1	0	2	0	0	1	1	0	0	0	1	0	0	1	0	1	0	1	1	1	0	0	0	0	1	1	0	0	–	–	–	–	W
Y	1	2	0	3	1	0	0	0	1	3	1	2	4	2	2	1	1	1	2	1	3	2	3	2	3	3	2	2	4	2	3	3	1	6	3	1	0	2	0	1	3	1	1	0	1	2	2	0	2	1	3	0.580	0.420	5.63E-06	*	Y
total	50	48	46	74	49	60	47	52	51	60	76	63	71	72	65	69	65	55	72	48	57	57	55	51	56	55	37	54	53	48	49	56	60	56	60	62	61	51	56	45	61	53	43	56	47	58	49	38	50	47	58	0.957	0.152			total

position -3

	C50	C49	C48	C47	C46	C45	C44	C43	C42	C41	C40	C39	C38	C37	C36	C35	C34	C33	C32	C31	C30	C29	C28	C27	C26	C25	C24	C23	C22	C21	C20	C19	C18	C17	C16	C15	C14	C13	C12	C11	C10	C9	C8	C7	C6	C5	C4	C3	C2	C1	C0	C-index	SD	*P-value*	mark	
A	3	2	3	10	5	8	3	3	3	2	7	2	8	2	5	6	3	2	4	1	8	4	6	6	3	8	1	6	4	3	3	2	3	7	3	7	3	6	2	4	6	5	1	2	3	5	1	2	5	3	5	0.816	0.444	7.83E-02	n.s.	A
C	0	3	2	2	0	1	2	0	0	0	3	2	1	2	3	3	2	1	1	0	0	1	0	0	0	1	2	2	0	1	1	0	1	0	0	1	1	2	1	0	2	1	1	1	1	0	4	2	1	0	1	1.100	1.035	–	–	C
D	2	2	2	2	4	2	2	2	6	5	4	7	5	5	4	3	1	4	2	2	2	3	2	2	3	1	1	2	5	1	1	3	1	2	0	0	5	1	2	2	3	2	4	2	1	1	3	3	3	3	4	0.650	0.381	1.29E-05	*	D
E	1	2	2	3	2	4	4	2	3	4	4	3	4	5	3	4	4	1	3	5	2	2	0	1	4	4	3	3	1	6	3	2	6	2	7	3	1	0	5	5	6	2	5	6	4	3	4	1	6	1	8	0.403	0.215	5.55E-08	*	E
F	1	1	1	1	3	2	1	2	3	3	7	3	1	3	1	1	2	1	4	1	2	2	1	3	1	1	2	1	1	3	1	5	3	3	5	6	4	2	4	4	4	1	0	0	1	1	1	0	1	2	4	0.535	0.391	9.82E-08	*	F
G	4	6	3	8	2	4	2	5	3	7	3	8	5	7	5	6	4	9	9	5	9	6	4	5	6	1	6	3	5	7	5	6	9	4	9	7	11	9	2	4	2	2	3	7	3	3	3	3	1	2	5	1.008	0.498	–	–	G
H	0	0	0	4	2	0	3	1	2	2	2	1	5	0	0	1	3	2	2	3	1	0	2	2	2	0	1	0	0	1	0	1	2	1	3	1	0	0	4	0	2	0	0	5	0	1	0	0	1	2	1	1.300	1.374	–	–	H
I	5	3	2	5	3	5	3	3	2	1	3	3	6	4	0	2	2	0	2	1	0	3	4	0	1	2	1	3	2	2	2	0	1	6	2	2	3	3	2	2	3	4	1	2	5	13	4	0	3	1	3	0.880	0.718	4.63E-01	n.s.	I
K	3	3	5	1	3	4	2	1	1	4	4	3	1	4	2	6	2	1	3	2	3	2	3	0	3	5	2	4	1	0	1	1	3	5	1	0	2	2	1	2	2	4	2	2	2	2	5	3	6	2	4	0.630	0.375	8.78E-06	*	K
L	7	2	6	7	0	7	4	7	8	6	8	1	7	5	13	13	10	11	9	6	10	6	4	3	2	7	3	5	4	2	7	6	2	8	8	2	4	6	7	3	1	3	3	3	1	4	2	3	2	2	4	1.300	0.782	–	–	L
M	0	1	0	0	5	0	0	2	0	1	2	2	0	0	0	0	1	1	1	3	2	1	1	1	0	0	0	1	0	1	1	0	2	0	1	1	1	1	0	0	1	1	0	1	2	1	0	1	1	1	0	–	–	–	–	M
N	2	1	0	1	1	3	4	2	1	3	6	6	3	3	1	2	2	5	2	3	0	3	0	1	3	8	2	1	5	3	3	1	2	3	2	3	0	0	5	4	4	1	3	3	4	2	1	4	3	4	1	2.580	1.727	–	–	N
P	7	5	4	4	2	1	2	1	2	8	4	3	4	7	4	1	6	0	5	3	2	8	1	5	4	5	1	6	2	2	3	4	6	3	4	3	6	3	5	1	2	5	3	5	4	2	3	1	1	3	4	0.880	0.493	8.95E-01	n.s.	P
Q	1	2	3	1	3	4	2	3	2	0	2	4	2	5	2	0	5	2	3	4	1	0	1	1	2	1	0	1	2	0	3	3	2	3	0	4	1	1	1	1	1	2	3	2	4	3	2	2	2	2	1	2.020	1.301	–	–	Q
R	1	3	2	6	0	2	0	2	2	3	6	2	5	5	0	3	3	1	6	1	2	1	4	2	4	3	1	1	2	2	2	4	3	1	2	3	2	2	2	2	3	1	3	2	1	2	4	0	2	3	1	2.380	1.497	–	–	R
S	3	4	4	4	3	2	4	4	1	4	4	1	2	5	6	3	5	5	4	3	4	7	6	2	4	0	2	3	5	1	7	7	2	1	4	9	7	3	5	1	4	2	2	6	2	3	1	4	2	5	4	0.910	0.486	1.00E+00	n.s.	S
T	2	1	2	2	5	4	3	2	4	2	3	5	3	3	8	4	4	1	5	2	3	2	8	9	1	2	1	5	4	7	2	5	5	3	2	0	3	2	0	5	4	4	2	0	4	5	5	2	4	5	3	1.127	0.673	–	–	T
V	2	6	3	8	4	5	5	9	5	4	2	2	6	5	4	6	3	6	6	2	3	5	5	7	4	3	6	6	6	2	2	3	4	2	3	7	3	5	5	4	5	6	5	6	3	5	2	2	4	5	4	1.105	0.432	–	–	V
W	1	0	1	1	0	0	0	0	0	0	0	0	1	1	1	0	0	1	0	0	0	0	0	1	4	1	1	0	0	0	0	0	0	0	2	0	2	0	0	1	0	0	0	1	0	0	2	1	0	1	1	0.480	0.789	7.41E-04	n.s.	W
Y	5	1	1	4	2	2	1	1	3	1	2	5	2	1	3	5	3	1	1	1	3	1	3	0	5	2	1	1	4	4	2	3	3	2	2	3	2	3	3	0	6	7	2	0	2	2	2	4	2	0	0	–	–	–	–	Y
total	50	48	46	74	49	60	47	52	51	60	76	63	71	72	65	69	65	55	72	48	57	57	55	51	56	55	37	54	53	48	49	56	60	56	60	62	61	51	56	45	61	53	43	56	47	58	49	38	50	47	58	0.957	0.152			total

position -1

	C50	C49	C48	C47	C46	C45	C44	C43	C42	C41	C40	C39	C38	C37	C36	C35	C34	C33	C32	C31	C30	C29	C28	C27	C26	C25	C24	C23	C22	C21	C20	C19	C18	C17	C16	C15	C14	C13	C12	C11	C10	C9	C8	C7	C6	C5	C4	C3	C2	C1	C0	C-index	SD	*P-value*	mark	
A	2	0	4	5	6	5	2	3	5	3	2	8	5	7	2	5	1	4	4	1	2	5	4	4	1	9	2	5	4	3	2	5	7	4	2	4	2	2	2	5	4	5	3	4	2	3	3	0	3	3	4	0.890	0.477	9.68E-01	n.s.	A
C	0	2	0	1	0	2	0	0	2	0	2	1	1	1	0	1	0	1	1	0	0	2	0	0	0	0	0	1	1	1	2	0	1	1	0	0	1	1	1	1	0	1	1	0	1	1	2	2	1	2	2	0.390	0.368	5.61E-08	*	C
D	4	2	3	6	1	6	4	6	1	4	5	3	9	3	3	4	3	1	1	4	4	4	4	1	2	2	1	3	3	2	3	2	4	1	3	4	5	3	5	1	4	2	3	0	2	4	3	2	0	2	6	0.507	0.288	5.55E-08	*	D
E	2	5	4	4	3	1	3	1	3	5	0	1	8	4	4	1	2	2	7	8	5	5	2	2	2	3	4	2	1	3	3	7	4	3	4	2	2	4	3	5	3	2	2	4	2	2	6	2	1	2	4	0.800	0.460	3.85E-02	n.s.	E
F	1	3	2	5	0	2	2	4	7	4	5	0	3	3	4	1	7	1	1	2	1	0	1	0	2	1	2	3	2	1	2	4	1	2	5	2	2	4	4	1	3	3	3	4	2	3	5	3	2	1	3	0.840	0.556	4.63E-01	n.s.	F
G	3	7	0	3	6	4	4	2	5	6	9	7	4	10	10	15	9	13	12	7	6	3	4	3	6	2	3	1	4	6	2	2	5	4	6	9	5	2	3	7	4	4	2	4	5	2	4	1	4	0	3	1.660	1.079	–	–	G
H	0	1	2	3	0	0	2	1	1	1	1	0	1	1	0	1	2	0	1	0	2	1	4	0	0	2	1	0	3	1	1	1	1	2	2	2	1	1	2	0	2	0	0	1	2	0	0	0	1	2	1	1.060	0.956	–	–	H
I	3	1	3	5	2	4	2	4	2	3	5	4	2	1	2	1	3	2	2	2	4	3	3	2	4	4	4	0	0	2	1	3	2	3	2	3	2	2	0	1	2	3	3	5	2	7	2	0	2	1	0	–	–	–	–	I
K	2	6	6	3	3	3	4	2	1	2	4	3	2	4	2	7	2	3	6	0	4	2	3	2	6	5	0	1	0	1	3	3	5	4	4	5	3	1	2	2	4	3	5	1	1	4	2	5	3	3	3	1.013	0.567	–	–	K
L	8	4	3	10	5	4	5	8	2	1	4	5	7	4	5	9	6	6	9	3	3	5	3	8	9	3	4	5	6	7	5	7	4	5	2	4	2	5	5	3	4	4	2	3	6	3	1	2	7	5	3	1.600	0.732	–	–	L
M	2	1	2	1	0	0	1	1	2	0	2	1	0	1	1	1	2	0	2	0	0	2	0	1	0	0	0	0	1	0	1	1	0	0	1	0	3	0	0	0	1	3	1	0	1	2	0	0	2	1	2	0.410	0.437	1.20E-07	*	M
N	3	2	2	3	2	5	3	3	1	3	1	2	2	3	3	2	3	2	5	5	3	1	5	0	4	0	2	4	1	2	2	3	2	2	1	2	7	3	4	3	2	3	2	4	1	1	2	1	4	4	2	1.300	0.700	–	–	N
P	5	3	3	2	6	5	4	3	1	2	8	8	5	6	4	2	7	2	4	4	3	3	7	5	5	1	2	5	3	3	3	1	5	3	9	6	3	4	5	3	9	1	2	3	8	3	6	2	3	3	3	1.353	0.704	–	–	P
Q	1	0	1	5	1	3	0	1	2	1	4	2	3	5	2	3	3	2	2	3	0	1	1	4	1	3	1	3	4	1	3	3	1	6	3	2	2	2	7	0	5	1	5	2	1	6	0	1	2	3	3	0.787	0.570	1.37E-01	n.s.	Q
R	3	3	0	4	3	5	2	2	5	5	5	1	5	5	3	2	4	1	4	2	3	3	1	1	3	2	3	2	2	2	1	3	4	4	3	3	7	3	3	1	4	2	1	3	2	4	3	5	3	3	2	1.480	0.707	–	–	R
S	6	2	5	5	4	4	1	3	3	5	5	5	4	6	3	2	4	6	3	1	4	2	3	5	3	11	4	3	5	4	4	2	3	3	6	1	2	4	2	0	3	6	1	3	4	4	3	3	3	3	2	1.810	0.903	–	–	S
T	1	2	2	2	2	4	5	2	4	3	5	3	4	0	5	1	4	3	2	1	7	3	1	3	3	3	2	9	2	5	3	1	3	2	2	2	4	7	2	4	2	3	3	4	1	2	2	3	3	3	2	1.490	0.842	–	–	T
V	2	3	3	4	4	2	3	4	1	8	5	4	5	8	9	7	1	4	2	2	5	8	6	8	2	3	2	3	8	2	5	7	5	4	2	3	7	3	5	6	2	3	1	9	1	7	2	4	5	4	5	0.852	0.462	2.07E-01	n.s.	V
W	1	1	0	2	0	0	0	0	0	1	2	2	0	0	2	0	0	0	1	1	1	0	1	0	0	1	0	0	0	0	0	0	0	0	0	2	0	0	0	0	1	2	0	0	0	0	0	0	1	0	3	0.147	0.235	5.55E-08	*	W
Y	1	0	1	1	1	1	0	2	3	3	2	3	1	0	1	4	2	2	3	2	0	4	2	2	3	0	0	4	3	2	3	1	3	3	3	6	1	0	1	2	2	2	3	2	3	0	3	2	0	2	5	0.380	0.266	5.55E-08	*	Y
total	50	48	46	74	49	60	47	52	51	60	76	63	71	72	65	69	65	55	72	48	57	57	55	51	56	55	37	54	53	48	49	56	60	56	60	62	61	51	56	45	61	53	43	56	47	58	49	38	50	47	58	0.957	0.152			total

Additional file 5. Continued.

NEMATODE x-x-S-x-I

position -4

	C50	C49	C48	C47	C46	C45	C44	C43	C42	C41	C40	C39	C38	C37	C36	C35	C34	C33	C32	C31	C30	C29	C28	C27	C26	C25	C24	C23	C22	C21	C20	C19	C18	C17	C16	C15	C14	C13	C12	C11	C10	C9	C8	C7	C6	C5	C4	C3	C2	C1	C0	C-index	SD	*P-value*	mark	
A	11	7	8	3	5	4	5	6	5	6	4	7	5	6	3	3	12	6	7	7	6	6	2	5	3	6	8	4	8	3	11	4	3	4	6	3	5	5	5	8	5	5	5	5	3	8	6	5	4	3	15	0.365	0.145	5.55E-08	✻	A
C	3	1	3	2	1	2	1	2	2	2	0	5	0	14	5	5	6	3	1	2	4	1	1	0	3	2	2	2	1	2	4	0	2	4	0	1	2	1	1	1	1	1	3	2	4	1	0	3	2	2	2	1.130	1.115	−	−	C
D	5	5	3	7	6	4	6	5	7	4	2	5	1	5	5	5	2	5	10	6	8	1	4	4	5	3	3	2	7	3	3	4	4	5	10	2	7	3	2	9	3	7	3	7	4	7	7	8	4	3	3	1.600	0.735	−	−	D
E	4	7	7	6	12	3	3	4	4	4	4	7	3	10	2	3	4	3	4	5	6	3	7	2	4	3	4	10	6	10	7	5	5	6	2	8	5	4	4	6	3	6	4	2	9	4	7	8	4	7	11	0.473	0.214	5.55E-08	✻	E
F	12	8	8	5	6	6	6	7	5	7	4	6	4	3	7	6	9	2	3	0	6	6	7	4	2	4	6	7	5	6	5	4	3	2	3	5	3	3	5	4	8	3	5	0	4	5	5	5	6	6	5	1.004	0.438	−	−	F
G	4	4	6	4	4	2	3	5	2	7	4	4	5	6	6	2	6	3	7	4	6	5	4	3	3	8	5	5	7	4	2	9	6	8	6	7	8	4	4	3	2	11	3	8	5	5	6	5	8	3	3	1.673	0.682	−	−	G
H	4	3	2	2	1	1	1	1	1	1	0	3	2	3	0	1	1	2	0	2	4	3	2	3	0	2	4	1	0	1	1	4	4	3	4	1	2	2	3	4	1	2	3	1	1	2	3	2	2	5	3	0.673	0.429	3.35E-02	n.s.	H
I	4	4	13	13	11	3	6	5	9	12	7	11	7	10	8	5	9	5	10	9	4	3	10	7	11	10	7	9	5	6	8	6	5	5	13	4	10	5	8	6	13	6	9	8	6	8	12	4	3	5	5	1.508	0.591	−	−	I
K	4	8	7	6	7	5	5	5	5	4	6	2	5	6	22	2	3	6	5	2	5	2	3	9	5	4	1	7	4	9	4	9	12	10	11	9	7	9	7	13	11	4	4	10	4	9	12	10	6	9	6	1.113	0.617	−	−	K
L	16	10	13	6	9	9	9	11	14	13	5	11	17	11	5	8	9	4	8	11	5	12	14	5	5	12	9	11	5	6	6	14	13	11	13	5	12	3	8	14	6	7	14	6	6	9	6	12	12	10	11	0.855	0.320	1.00E+00	n.s.	L
M	6	4	0	3	4	4	4	3	2	1	6	2	5	3	3	2	2	2	2	3	4	5	3	1	1	3	5	0	8	1	2	2	2	2	1	2	2	2	1	2	7	1	0	3	4	1	4	0	7	3	0	−	−	−	−	M
N	5	5	2	4	5	6	5	4	3	6	7	5	12	2	6	3	4	2	3	5	1	2	7	4	5	3	6	3	3	8	4	4	4	8	10	2	7	1	3	10	3	6	3	10	3	5	8	1	12	11	6	0.837	0.472	3.61E-01	n.s.	N
P	4	13	6	3	4	3	8	1	7	4	4	3	7	6	2	7	6	3	5	4	6	5	3	2	6	7	8	3	4	4	6	5	3	6	6	7	3	6	8	4	7	6	4	5	4	3	1	15	3	4	6	0.847	0.430	9.26E-01	n.s.	P
Q	8	3	2	6	4	1	4	1	2	5	4	6	1	0	5	5	4	4	5	0	3	6	12	3	7	2	2	2	3	3	2	2	3	1	3	1	5	4	4	6	5	3	5	4	3	6	7	3	3	9	4	0.960	0.581	−	−	Q
R	3	4	5	4	0	6	6	9	7	6	7	10	7	3	6	3	4	7	4	5	3	7	8	6	11	9	10	5	6	8	7	4	6	5	4	8	6	8	4	2	8	6	10	5	3	11	14	11	6	2	4	1.545	0.692	−	−	R
S	10	3	8	13	6	22	8	16	5	6	12	2	8	8	4	14	10	9	10	12	12	4	7	11	8	7	8	7	14	4	6	3	7	15	12	10	11	9	7	11	15	13	6	8	11	12	9	11	9	11	15	0.619	0.255	8.17E-08	✻	S
T	7	4	5	11	14	4	10	4	9	8	8	7	8	8	3	4	5	6	5	8	8	4	12	8	11	9	6	6	3	6	12	11	4	6	9	13	3	16	6	5	6	6	5	7	6	8	10	2	11	9	9	0.813	0.345	4.87E-01	n.s.	T
V	7	3	3	7	7	1	4	6	10	8	11	11	5	10	5	5	4	5	6	7	8	10	8	8	9	5	8	13	12	5	5	2	3	11	12	6	6	13	8	3	7	8	6	10	3	5	3	5	2	8	8	0.843	0.381	9.96E-01	n.s.	V
W	3	1	2	0	1	1	0	0	0	1	0	1	2	0	1	0	1	0	1	2	1	0	1	1	1	1	1	0	0	1	1	0	2	2	0	0	0	1	2	0	1	2	0	0	3	2	1	0	0	3	0	−	−	−	−	W
Y	1	3	2	1	2	4	5	5	4	4	5	4	2	1	3	5	1	5	1	2	4	0	5	1	1	2	1	0	4	4	2	5	2	2	0	4	2	0	3	2	4	5	4	3	1	0	2	2	3	2	2	1.300	0.808	8.53E-03	n.s.	Y
total	121	100	105	106	109	91	99	100	103	109	100	112	106	115	101	88	102	82	97	96	104	85	120	87	101	102	104	97	105	94	98	97	93	116	125	98	106	99	93	113	116	108	96	104	87	111	123	112	107	115	118	0.874	0.084			total

position -3

	C50	C49	C48	C47	C46	C45	C44	C43	C42	C41	C40	C39	C38	C37	C36	C35	C34	C33	C32	C31	C30	C29	C28	C27	C26	C25	C24	C23	C22	C21	C20	C19	C18	C17	C16	C15	C14	C13	C12	C11	C10	C9	C8	C7	C6	C5	C4	C3	C2	C1	C0	C-index	SD	*P-value*	mark	
A	5	6	3	3	3	3	2	3	4	1	3	7	8	5	6	8	13	5	9	6	7	6	7	2	6	5	7	10	3	6	8	2	2	10	7	4	6	3	5	2	6	5	4	13	6	3	7	4	3	5	8	0.668	0.336	9.62E-05	✻	A
C	5	1	1	2	2	5	3	2	2	1	1	2	1	1	3	0	1	1	3	4	2	2	2	1	1	3	2	2	2	1	2	0	0	2	1	3	3	0	4	2	1	3	4	1	2	1	2	4	2	4	1	2.000	1.245	−	−	C
D	5	2	5	0	8	7	6	8	6	8	6	5	6	5	6	4	7	10	6	4	9	3	8	12	7	6	6	1	5	5	5	3	2	5	10	3	7	6	3	10	5	8	4	8	4	2	5	2	9	5	4	1.410	0.630	−	−	D
E	5	8	5	8	6	4	4	3	4	5	1	1	4	5	2	5	4	1	3	3	5	3	14	2	10	3	5	9	8	3	3	6	2	3	2	1	5	3	5	9	7	5	3	4	4	4	2	6	4	3	7	0.640	0.365	1.75E-06	✻	E
F	4	10	7	1	5	11	9	6	4	6	6	6	9	10	7	4	6	4	1	9	6	4	10	6	8	3	3	6	5	2	3	4	7	8	10	7	8	3	7	6	4	6	6	9	10	7	9	2	7	9	8	0.775	0.321	4.64E-01	n.s.	F
G	2	2	1	4	4	3	8	12	7	5	7	2	3	4	7	1	5	3	10	6	4	4	2	6	4	7	6	5	6	8	10	3	3	5	6	6	8	4	0	5	3	7	4	5	6	4	5	0	9	3	2	2.440	1.284	−	−	G
H	3	5	2	2	4	2	1	4	3	4	3	5	2	4	5	4	2	1	3	4	1	0	1	4	1	4	0	1	1	5	2	0	3	3	2	2	1	3	1	0	3	1	6	3	2	1	1	3	1	3	2	1.220	0.764	−	−	H
I	10	4	12	15	4	11	9	11	10	14	16	6	10	6	4	9	9	8	12	5	10	4	10	4	8	0	10	11	8	5	5	13	3	10	9	3	8	6	4	11	16	7	5	5	6	16	20	10	4	12	8	1.070	0.517	−	−	I
K	10	5	6	14	5	8	6	6	7	6	3	11	5	4	5	4	7	3	4	5	4	6	8	3	8	5	8	4	3	6	2	7	14	9	8	3	8	4	10	7	8	6	6	7	6	9	5	11	3	6	7	0.909	0.385	−	−	K
L	11	15	16	10	17	6	12	7	10	15	9	19	16	17	7	8	10	10	7	9	11	8	8	11	8	5	4	8	11	9	9	11	8	6	11	12	4	8	7	9	10	11	12	7	7	7	9	11	12	9	2	4.940	1.677	−	−	L
M	2	4	2	0	2	2	0	4	0	5	1	4	0	1	0	3	2	1	4	3	3	2	4	2	2	7	4	4	0	4	5	6	3	6	2	2	2	3	1	3	1	2	2	1	2	1	1	1	5	4	4	0.625	0.432	7.70E-03	n.s.	M
N	4	6	6	7	7	1	3	7	4	6	6	10	3	6	4	6	5	5	2	5	5	1	6	5	3	2	4	5	5	2	5	6	2	7	7	3	6	6	7	10	6	7	9	6	2	9	6	7	8	4	7	0.754	0.310	2.48E-01	n.s.	N
P	8	5	3	4	6	3	4	6	6	4	5	2	8	9	2	2	4	2	7	5	6	7	8	7	1	10	6	3	5	8	7	4	4	4	8	4	5	6	1	8	5	5	6	4	3	5	3	10	2	11	8	0.653	0.300	2.20E-04	n.s.	P
Q	5	2	5	2	5	5	2	0	6	5	3	1	4	5	3	1	1	3	4	2	1	2	6	5	2	4	1	5	7	1	3	3	4	5	5	3	3	4	6	3	5	2	2	1	3	7	3	2	2	6	6	0.567	0.297	9.93E-07	✻	Q
R	10	5	3	6	5	4	6	4	8	4	4	5	2	2	20	3	3	5	6	4	6	9	0	4	6	5	11	5	6	2	4	3	6	3	5	3	4	3	3	7	4	3	7	6	4	6	4	7	3	3	13	0.386	0.230	5.55E-08	✻	R
S	9	6	9	10	12	9	6	5	10	5	9	11	8	11	6	9	12	7	6	7	10	12	13	5	9	18	5	7	10	9	11	10	13	12	10	16	14	11	11	9	12	13	4	12	3	10	21	13	14	6	10	0.980	0.356	−	−	S
T	9	6	8	3	7	7	10	5	6	8	6	8	6	9	0	7	2	4	3	5	3	7	6	2	7	5	8	2	7	9	5	9	3	6	5	9	7	7	8	4	9	6	4	4	4	6	8	10	5	4	11	0.542	0.213	5.55E-08	✻	T
V	9	8	7	12	3	0	6	5	3	4	7	4	7	6	8	6	6	4	4	6	5	3	3	3	8	4	7	5	6	2	4	4	9	10	12	10	2	12	7	7	5	5	5	5	8	6	8	6	11	14	7	0.889	0.418	−	−	V
W	3	0	0	1	0	0	2	1	0	1	1	1	0	1	3	1	0	2	1	1	0	1	2	0	0	2	1	2	2	1	2	0	0	0	0	0	0	0	0	0	0	0	1	1	0	3	2	2	0	0	1	0.820	0.941	1.00E+00	n.s.	W
Y	2	0	4	2	4	0	0	1	3	2	3	2	4	4	3	3	3	3	2	3	6	1	2	3	2	4	6	2	5	6	3	3	5	2	5	4	5	7	3	1	6	6	2	2	5	4	2	1	3	4	2	1.580	0.853	−	−	Y
total	121	100	105	106	109	91	99	100	103	109	100	112	106	115	101	88	102	82	97	96	104	85	120	87	101	102	104	97	105	94	98	97	93	116	125	98	106	99	93	113	116	108	96	104	87	111	123	112	107	115	118	0.874	0.084			total

position -1

	C50	C49	C48	C47	C46	C45	C44	C43	C42	C41	C40	C39	C38	C37	C36	C35	C34	C33	C32	C31	C30	C29	C28	C27	C26	C25	C24	C23	C22	C21	C20	C19	C18	C17	C16	C15	C14	C13	C12	C11	C10	C9	C8	C7	C6	C5	C4	C3	C2	C1	C0	C-index	SD	*P-value*	mark	
A	7	5	5	8	7	3	7	5	9	9	5	6	6	3	3	8	5	7	3	8	9	6	10	10	7	3	5	6	3	4	7	5	8	10	5	5	5	9	3	8	9	8	3	3	3	8	5	4	2	2	3	1.960	0.779	−	−	A
C	3	2	2	0	1	4	0	1	2	2	1	2	1	1	3	7	1	3	6	3	2	0	2	0	0	4	1	0	1	0	3	1	1	1	2	2	0	0	1	1	2	0	4	1	0	2	0	4	0	0	1	1.600	1.591	−	−	C
D	5	4	6	10	1	3	3	10	3	3	4	7	5	4	1	1	3	1	5	4	3	4	3	4	5	4	7	7	7	2	5	1	4	4	12	8	5	9	5	5	5	7	3	3	6	3	1	5	3	3	4	1.130	0.615	−	−	D
E	4	8	5	9	2	6	12	5	9	5	9	7	4	7	4	3	3	11	3	4	4	3	6	4	5	4	3	9	8	9	4	4	5	3	5	5	6	5	4	5	6	4	7	7	1	8	10	7	6	6	4	1.415	0.599	−	−	E
F	5	1	3	4	4	11	2	3	8	6	4	3	8	10	2	4	1	1	5	5	8	2	3	6	4	4	6	4	6	5	3	3	5	6	4	3	4	2	5	6	3	4	5	2	7	1	8	5	2	8	5	0.896	0.463	−	−	F
G	5	6	2	8	5	3	7	4	4	1	5	10	3	4	2	5	3	3	6	5	3	4	3	6	1	2	3	8	7	10	7	2	3	8	9	2	3	5	4	0	6	7	0	5	6	4	5	9	3	6	3	1.547	0.821	−	−	G
H	2	1	1	4	4	3	0	2	4	0	2	3	2	2	1	1	2	3	2	1	5	2	2	1	2	1	1	0	1	3	2	5	3	2	2	0	3	1	0	0	2	1	3	4	0	2	0	0	6	1	5	0.380	0.292	5.55E-08	✻	H
I	6	6	9	5	9	2	2	4	5	7	4	6	6	3	8	3	6	1	8	6	3	5	9	8	10	4	3	9	7	6	6	6	9	5	11	6	7	8	6	10	8	6	3	5	8	6	4	6	6	4	11	0.545	0.208	5.55E-08	✻	I
K	7	8	9	3	4	13	6	8	2	7	7	5	6	7	4	4	10	2	5	7	8	5	7	4	1	4	11	11	5	7	10	13	6	5	7	6	12	12	2	12	7	8	6	12	7	7	8	7	7	9	12	0.583	0.247	1.32E-07	✻	K
L	13	10	6	6	8	6	10	10	6	8	12	7	6	11	9	11	9	7	13	6	9	3	11	12	8	14	7	7	11	9	10	10	5	8	14	9	5	4	12	9	10	8	8	5	5	18	9	5	2	15	6	1.453	0.538	−	−	L
M	9	0	4	2	1	0	4	4	1	3	3	2	2	0	3	1	7	1	4	2	4	1	3	2	3	6	7	1	4	1	4	1	0	4	1	2	2	2	3	3	3	1	2	2	2	1	2	1	1	2	2	1.240	0.938	−	−	M
N	4	1	6	2	7	7	3	3	2	8	6	2	2	7	2	1	8	2	5	5	6	5	5	3	3	5	4	3	6	3	3	0	5	6	5	11	4	3	3	7	9	10	2	10	4	2	4	6	7	10	7	0.677	0.375	4.89E-03	n.s.	N
P	6	5	4	8	16	0	11	6	13	4	4	6	6	4	9	4	4	5	1	3	6	7	9	5	7	7	5	4	5	3	3	5	2	6	5	3	4	5	7	2	7	1	3	6	6	3	4	3	7	7	3	1.773	0.958	−	−	P
Q	7	5	2	4	6	3	3	4	2	6	2	2	6	3	5	1	3	4	1	2	4	3	6	3	5	4	4	1	4	5	3	6	2	4	4	6	3	3	4	2	3	3	3	2	1	5	8	1	3	4	7	0.514	0.238	5.55E-08	✻	Q
R	3	5	4	8	5	4	5	4	3	5	6	4	9	8	4	6	8	2	4	2	5	6	7	1	6	6	4	3	2	1	6	5	7	10	11	5	5	11	7	6	5	6	5	6	7	6	13	6	10	9	11	0.520	0.234	5.55E-08	✻	R
S	10	13	10	8	9	7	10	9	7	15	9	7	9	8	4	5	12	6	12	9	6	9	11	6	14	15	10	7	6	9	8	12	10	16	12	10	12	13	9	14	10	14	12	5	11	16	20	13	13	10	10	1.024	0.330	−	−	S
T	12	13	17	7	12	9	4	7	12	15	7	20	13	16	23	9	5	13	8	13	8	4	5	5	7	5	11	5	11	8	6	11	9	10	5	9	11	3	8	11	8	10	11	18	6	13	9	13	15	7	7	1.420	0.617	−	−	T
V	9	6	8	5	6	2	5	9	4	4	5	11	11	4	8	8	11	8	5	5	6	6	5	2	8	8	7	5	7	4	5	3	5	4	5	2	11	3	7	8	9	8	8	6	4	5	7	14	8	8	9	0.716	0.288	5.64E-03	n.s.	V
W	0	0	0	1	0	0	0	1	1	0	1	1	1	3	2	2	0	0	1	1	1	3	3	3	1	1	1	0	1	2	2	0	0	0	0	0	2	0	0	0	2	1	1	0	0	0	2	1	3	0	6	0.150	0.166	5.55E-08	✻	W
Y	4	1	2	4	2	5	5	1	6	1	4	1	0	10	4	4	1	2	0	5	4	7	10	2	4	1	4	7	3	3	1	4	4	4	6	4	2	1	3	4	2	1	7	2	3	1	4	2	3	4	2	1.690	1.129	−	−	Y
total	121	100	105	106	109	91	99	100	103	109	100	112	106	115	101	88	102	82	97	96	104	85	120	87	101	102	104	97	105	94	98	97	93	116	125	98	106	99	93	113	116	108	96	104	87	111	123	112	107	115	118	0.874	0.084			total

Additional file 5. Continued.

NEMATODE x-x-S-x-L

position -4

	C50	C49	C48	C47	C46	C45	C44	C43	C42	C41	C40	C39	C38	C37	C36	C35	C34	C33	C32	C31	C30	C29	C28	C27	C26	C25	C24	C23	C22	C21	C20	C19	C18	C17	C16	C15	C14	C13	C12	C11	C10	C9	C8	C7	C6	C5	C4	C3	C2	C1	C0	C-index	SD	*P-value*	mark	
A	9	7	11	11	6	11	6	12	4	7	6	5	14	8	9	8	9	6	5	8	4	8	8	12	10	7	5	10	4	9	8	9	5	8	5	6	9	13	4	11	9	4	4	4	7	2	10	7	13	4	10	0.762	0.286	–	–	A
C	0	5	3	2	1	1	3	2	1	4	0	4	4	0	3	2	4	1	2	5	4	1	4	3	2	2	2	3	1	3	2	2	2	4	2	2	4	1	2	0	3	2	2	1	3	3	1	3	1	2	6	0.380	0.216	5.62E-06	*	C
D	5	7	7	7	5	3	4	4	8	8	4	11	8	11	6	8	10	12	5	9	7	4	8	12	8	7	4	9	10	10	12	8	14	5	11	8	4	4	9	4	8	9	6	4	7	7	9	4	5	17	15	0.501	0.200	1.06E-02	n.s.	D
E	6	5	14	13	5	20	1	8	5	10	5	10	6	7	10	6	9	9	4	10	7	4	4	7	13	9	10	6	6	6	7	8	9	4	9	1	7	8	7	12	4	8	5	4	4	7	10	6	9	5	12	0.615	0.281	–	–	E
F	6	7	10	10	5	7	12	7	13	13	7	11	6	13	11	9	3	7	12	7	3	8	6	6	4	2	6	8	8	11	2	8	7	8	4	1	6	6	12	9	9	2	4	4	6	4	4	8	10	3	9	0.789	0.355	–	–	F
G	9	3	10	10	5	6	3	11	4	5	9	6	5	7	3	3	10	4	4	3	2	10	4	4	8	12	8	6	4	2	4	4	4	2	14	18	7	2	7	3	5	6	5	3	6	8	2	5	6	2	14	0.419	0.243	2.42E-05	*	G
H	3	4	2	3	4	2	5	3	1	1	5	4	0	4	0	4	1	3	1	5	4	2	3	2	4	3	2	4	2	4	9	2	1	0	1	2	3	1	5	1	5	4	1	3	3	3	3	3	10	6	6	0.503	0.332	1.93E-01	n.s.	H
I	10	3	9	12	9	9	13	18	16	11	20	13	12	15	8	9	6	11	5	12	10	10	7	6	10	12	7	10	10	7	7	10	11	8	11	5	11	9	9	11	11	7	8	9	16	10	8	11	9	10	11	0.911	0.291	–	–	I
K	8	3	5	7	10	5	3	11	7	5	13	6	9	7	17	2	7	8	11	8	10	4	11	8	8	8	12	12	10	10	7	10	13	10	10	5	6	13	10	20	5	5	5	2	12	8	7	8	4	15	21	0.400	0.177	6.47E-08	*	K
L	22	9	13	16	10	18	9	12	20	8	16	20	11	8	17	12	13	15	13	6	8	10	6	10	12	12	10	15	13	12	11	14	13	10	4	9	12	12	11	12	17	15	14	13	9	11	8	12	15	13	8	1.528	0.457	–	–	L
M	5	1	1	3	4	2	4	1	5	1	2	1	5	2	5	6	3	1	4	5	10	3	1	1	3	3	5	2	5	3	1	2	5	4	5	4	1	5	3	2	1	2	4	1	6	0	0	0	4	2	15	0.199	0.134	5.55E-08	*	M
N	5	2	5	11	3	5	4	3	6	4	3	6	3	6	9	8	6	9	4	5	6	4	8	7	8	2	2	8	6	13	6	4	8	7	5	5	2	7	4	6	2	7	3	8	9	5	6	4	4	7	20	0.280	0.121	5.55E-08	*	N
P	8	6	4	5	11	9	1	9	5	5	8	3	6	8	4	3	4	6	3	6	6	4	4	5	2	11	2	2	3	2	8	7	4	9	8	3	9	5	3	7	7	7	7	8	5	3	8	10	5	6	6	0.947	0.421	–	–	P
Q	3	16	4	11	8	11	1	14	7	6	7	3	8	7	7	5	6	4	7	8	8	6	7	2	5	3	8	5	0	5	3	3	5	1	2	5	6	4	3	2	2	6	7	7	3	1	5	2	7	7	15	0.364	0.213	5.56E-08	*	Q
R	0	9	7	7	6	10	5	6	5	7	8	8	6	8	9	9	4	8	15	8	7	6	6	9	7	7	6	6	5	5	8	5	5	4	8	6	3	5	12	8	6	8	5	8	7	8	8	11	18	4	15	0.475	0.189	9.64E-07	*	R
S	8	11	11	10	8	7	8	17	9	6	6	5	9	7	9	14	14	18	10	10	11	15	6	17	9	5	14	8	13	12	10	10	13	8	15	13	14	16	18	17	18	9	9	12	19	12	10	19	14	17	16	0.725	0.248	–	–	S
T	8	12	4	11	9	11	11	8	3	7	7	5	6	5	9	3	5	10	8	7	4	8	5	12	6	10	10	8	10	3	6	8	7	5	9	11	7	12	13	9	6	8	12	10	7	11	8	7	10	8	9	0.887	0.291	–	–	T
V	7	8	14	16	10	7	7	15	3	6	9	10	8	17	6	14	12	8	6	7	8	12	12	6	6	8	10	9	7	7	2	11	14	7	10	0	6	12	8	5	11	7	5	11	13	16	9	9	9	11	10	0.902	0.360	–	–	V
W	1	0	3	5	0	0	2	3	1	1	0	1	0	2	0	1	3	0	3	1	7	1	2	4	1	2	0	2	0	0	0	1	3	0	2	1	0	2	1	2	0	0	1	4	0	1	1	3	2	2	3	0.480	0.500	3.87E-01	n.s.	W
Y	4	5	6	6	6	6	1	6	5	3	8	5	1	1	4	3	2	5	4	4	4	2	3	3	4	6	3	0	3	2	3	2	5	1	6	2	3	2	0	6	4	4	3	6	6	5	7	3	5	7	3	1.300	0.643	–	–	Y
total	127	123	143	176	125	150	103	170	128	118	143	137	127	143	146	129	131	145	126	134	130	122	115	136	130	131	126	133	120	126	116	128	148	105	141	107	120	139	141	147	133	120	110	122	148	125	124	135	160	148	224	0.590	0.067			total

position -3

	C50	C49	C48	C47	C46	C45	C44	C43	C42	C41	C40	C39	C38	C37	C36	C35	C34	C33	C32	C31	C30	C29	C28	C27	C26	C25	C24	C23	C22	C21	C20	C19	C18	C17	C16	C15	C14	C13	C12	C11	C10	C9	C8	C7	C6	C5	C4	C3	C2	C1	C0	C-index	SD	*P-value*	mark	
A	13	5	8	13	6	22	5	6	11	10	8	16	4	8	13	4	10	9	5	7	10	7	11	6	10	12	7	11	4	5	12	8	3	7	9	6	8	11	7	3	8	3	7	7	7	2	3	9	10	6	9	0.893	0.412	–	–	A
C	2	4	1	8	5	2	2	4	2	5	3	3	2	3	2	6	2	2	4	5	2	2	4	2	3	0	2	1	4	4	2	2	4	5	3	0	1	4	4	5	2	2	0	2	2	2	3	1	8	4	8	0.368	0.219	6.96E-08	*	C
D	4	4	6	9	6	6	2	4	5	4	4	8	8	4	5	6	7	6	4	7	6	9	8	9	1	2	3	5	6	8	7	7	9	3	10	3	6	4	8	6	3	11	3	4	7	7	5	7	5	7	10	0.576	0.224	1.00E+00	n.s.	D
E	4	10	4	8	2	5	4	9	6	5	7	8	7	6	5	5	10	4	4	3	2	5	5	8	5	8	3	6	4	6	7	8	3	7	6	5	4	8	8	7	8	6	3	2	5	4	5	3	4	7	9	0.618	0.229	–	–	E
F	7	14	15	12	10	10	7	12	6	2	9	8	7	6	11	4	6	4	9	7	5	10	4	7	8	10	7	5	5	5	10	4	8	8	6	5	6	5	8	8	6	3	9	7	8	9	10	9	6	18	17	0.453	0.181	4.44E-06	*	F
G	7	4	8	5	2	11	6	8	5	5	6	4	8	4	3	8	8	4	5	6	4	8	6	5	6	3	6	5	4	5	4	6	3	4	13	10	9	7	8	6	11	7	4	8	5	7	3	7	4	3	8	0.745	0.293	–	–	G
H	2	2	3	2	1	3	3	6	3	2	2	0	2	5	5	3	2	5	2	3	5	4	0	4	2	1	4	2	0	3	2	4	3	0	2	1	1	1	8	5	3	5	2	5	3	3	2	5	1	4	8	0.353	0.214	1.25E-07	*	H
I	8	6	9	13	10	10	10	16	4	13	10	8	10	8	11	5	8	7	7	8	8	9	8	4	12	8	11	12	6	6	12	12	13	9	9	12	5	9	8	11	6	6	4	10	16	13	9	10	9	10	16	0.573	0.177	9.96E-01	n.s.	I
K	6	8	9	12	10	9	7	6	8	9	10	5	5	8	10	5	4	6	6	5	6	7	6	8	9	5	8	6	9	6	8	3	10	8	6	4	10	9	11	8	6	3	10	10	14	13	8	8	12	5	21	0.366	0.120	5.55E-08	*	K
L	10	11	14	23	8	14	11	27	13	13	18	12	12	22	18	18	7	22	16	20	13	12	7	16	9	10	17	14	11	9	7	12	7	7	10	5	8	12	11	20	16	14	21	8	9	10	11	13	16	15	23	0.573	0.213	7.40E-01	n.s.	L
M	2	1	3	4	3	4	2	3	2	1	3	6	5	3	4	6	5	3	1	2	1	1	3	2	5	6	4	3	2	0	4	2	4	2	3	6	3	4	2	1	0	4	4	2	3	4	2	0	5	1	2	1.460	0.807	–	–	M
N	6	5	5	5	7	5	7	9	9	3	8	6	3	10	6	5	7	7	7	9	8	4	7	9	4	8	4	6	3	8	5	5	10	3	3	5	8	5	6	6	4	9	3	4	13	5	8	8	9	7	15	0.421	0.150	9.54E-08	*	N
P	4	5	5	3	4	4	3	5	9	4	5	7	8	5	7	6	9	11	9	9	10	4	6	13	3	6	6	5	6	11	3	8	6	7	4	5	3	6	11	9	3	8	6	4	3	9	5	8	11	15	10	0.652	0.287	–	–	P
Q	5	7	11	2	5	3	2	5	7	4	7	7	3	3	10	5	5	7	3	4	5	6	3	11	4	5	5	3	6	3	2	6	3	8	5	6	5	5	3	2	8	6	2	3	8	4	6	5	4	7	4	1.270	0.558	–	–	Q
R	11	13	9	7	6	7	8	5	7	4	7	11	6	6	8	9	11	12	5	5	10	9	5	7	7	9	8	4	14	5	3	1	9	4	9	6	6	9	5	13	5	2	3	2	11	4	9	5	16	7	15	0.485	0.218	1.21E-02	n.s.	R
S	13	6	13	20	7	15	8	17	13	14	18	15	10	15	10	10	12	16	16	11	11	12	13	12	18	10	8	15	9	14	11	20	19	8	13	10	11	14	14	13	20	13	13	15	18	7	20	14	10	14	26	0.506	0.140	3.20E-03	n.s.	S
T	7	5	3	11	9	4	2	11	4	2	7	5	8	8	7	8	7	5	11	7	8	8	7	8	7	10	7	7	6	11	5	3	16	4	8	8	10	12	7	6	12	9	4	5	5	5	6	10	8	3	15	0.475	0.191	2.10E-04	n.s.	T
V	9	9	12	11	15	7	7	10	12	12	6	7	10	12	9	9	3	10	5	8	6	4	6	4	8	6	12	13	14	10	10	9	13	7	11	8	7	6	9	13	9	4	8	18	6	13	4	6	13	5	3	2.967	1.101	–	–	V
W	3	1	1	1	1	0	0	2	1	3	2	1	5	0	1	3	3	1	2	4	6	1	2	0	2	3	2	1	1	1	0	1	2	1	1	0	1	1	1	1	1	1	2	0	1	0	1	3	2	1	0	–	–	–	–	W
Y	4	3	4	7	8	9	7	5	1	3	3	0	4	7	1	4	5	4	5	4	4	0	4	1	7	9	2	9	6	6	2	7	3	3	10	2	8	7	2	4	2	4	2	6	4	4	4	4	7	9	5	0.920	0.508	–	–	Y
total	127	123	143	176	125	150	103	170	128	118	143	137	127	143	146	129	131	145	126	134	130	122	115	136	130	131	126	133	120	126	116	128	148	105	141	107	120	139	141	147	133	120	110	122	148	125	124	135	160	148	224	0.590	0.067			total

position -1

	C50	C49	C48	C47	C46	C45	C44	C43	C42	C41	C40	C39	C38	C37	C36	C35	C34	C33	C32	C31	C30	C29	C28	C27	C26	C25	C24	C23	C22	C21	C20	C19	C18	C17	C16	C15	C14	C13	C12	C11	C10	C9	C8	C7	C6	C5	C4	C3	C2	C1	C0	C-index	SD	*P-value*	mark	
A	9	13	5	19	9	12	5	8	10	5	10	7	4	8	14	10	10	7	7	13	6	11	6	7	9	4	7	8	8	6	4	6	5	8	6	6	6	10	8	9	11	4	10	7	13	8	7	5	9	5	7	1.154	0.432	–	–	A
C	2	3	4	5	4	2	3	6	2	2	3	1	5	2	7	2	2	5	0	0	2	2	0	2	3	5	2	2	5	1	1	3	0	0	2	2	2	3	1	2	4	5	2	2	1	3	2	1	5	1	3	0.840	0.556	–	–	C
D	8	6	5	11	2	7	3	11	5	13	5	8	7	9	8	7	5	17	5	6	3	5	4	1	6	9	7	7	7	8	6	4	7	9	8	7	5	8	6	5	4	5	7	3	9	5	9	6	8	4	5	1.320	0.555	–	–	D
E	12	7	6	10	5	10	9	7	7	3	10	9	5	9	5	3	7	11	8	7	7	7	7	7	4	6	8	6	7	10	8	4	6	6	7	3	7	9	8	7	6	8	6	8	9	6	7	5	5	2	13	0.532	0.164	1.31E-01	n.s.	E
F	8	6	14	7	5	2	7	10	9	7	11	5	7	3	11	9	3	7	13	10	9	4	7	8	10	3	5	6	8	5	3	8	19	1	10	7	6	7	7	7	9	9	8	5	9	10	8	7	14	7	14	0.543	0.234	3.74E-01	n.s.	F
G	2	8	4	10	2	8	2	3	1	5	5	6	7	5	7	3	7	7	4	7	6	12	8	7	5	3	3	8	1	9	7	6	6	4	7	5	5	8	6	2	3	11	4	5	8	5	4	6	8	2	7	0.791	0.362	–	–	G
H	6	3	3	4	5	2	1	1	2	2	1	5	3	3	3	1	2	2	4	2	4	3	0	3	2	3	2	6	6	4	2	9	8	2	2	3	4	4	3	2	4	3	1	2	2	2	2	7	6	6	4	0.810	0.478	–	–	H
I	9	6	10	11	10	7	7	14	6	9	9	11	11	12	12	6	3	9	5	12	8	12	8	14	8	9	9	10	9	3	7	5	12	7	10	5	7	10	12	12	12	11	5	6	15	7	13	10	10	11	8	1.140	0.357	–	–	I
K	5	1	6	15	7	7	7	9	6	5	5	8	7	9	6	8	4	8	4	7	10	5	8	7	13	5	13	6	10	5	6	7	11	6	7	5	14	12	11	8	4	9	5	11	11	10	5	11	12	13	46	0.171	0.067	5.55E-08	*	K
L	13	10	10	15	13	14	8	11	8	7	11	9	6	10	16	10	16	6	16	14	21	15	17	12	9	15	11	10	11	12	11	13	10	9	10	10	10	9	17	9	15	9	9	11	12	13	7	7	9	23	17	0.681	0.211	–	–	L
M	5	2	4	2	3	7	1	7	5	4	2	1	2	1	3	2	8	5	2	1	1	1	3	2	4	3	6	3	1	3	8	2	1	4	4	0	1	3	2	2	6	1	2	1	1	4	3	3	3	2	4	0.735	0.488	–	–	M
N	4	7	6	7	4	2	3	5	10	4	6	5	6	8	8	2	7	4	3	4	5	1	3	7	6	6	6	5	6	12	3	7	3	9	3	7	7	2	5	11	9	6	6	10	3	2	4	10	10	10	15	0.385	0.179	1.89E-07	*	N
P	9	7	14	6	4	3	5	7	6	4	12	7	8	2	8	6	11	7	3	6	3	7	1	10	3	9	5	8	6	4	5	5	10	6	5	4	4	5	8	7	3	5	6	6	4	1	7	3	5	3	4	1.465	0.676	–	–	P
Q	4	6	2	11	3	5	3	8	7	6	9	8	8	6	5	7	6	6	13	3	9	5	4	5	5	5	4	6	6	3	9	9	6	4	6	4	5	5	6	13	2	3	6	5	4	7	5	3	4	4	5	1.152	0.494	–	–	Q
R	3	8	7	9	9	20	4	11	10	9	5	11	9	7	4	11	7	2	7	5	4	6	8	4	10	7	7	6	4	8	7	4	8	4	10	5	7	8	8	7	10	3	3	3	8	10	8	10	13	17	15	0.500	0.230	1.12E-02	n.s.	R
S	13	9	22	13	20	12	10	16	8	12	12	14	11	7	8	21	5	16	9	10	15	10	9	14	11	8	12	15	9	13	11	17	13	10	16	13	13	13	8	18	14	10	12	10	15	12	12	10	12	7	20	0.610	0.179	–	–	S
T	8	8	8	2	2	12	9	12	8	7	7	12	6	15	11	8	14	13	8	9	6	7	11	7	11	10	8	10	6	5	11	8	9	7	12	11	6	8	14	10	7	6	8	8	9	5	8	9	10	8	19	0.457	0.144	3.68E-06	*	T
V	3	6	6	9	10	7	10	8	10	10	9	6	9	17	7	8	7	8	12	10	6	5	7	12	6	11	6	7	6	9	1	8	10	4	7	4	6	8	5	14	6	8	7	9	11	6	8	10	9	13	7	1.146	0.407	–	–	V
W	2	0	1	2	0	1	2	0	4	0	3	2	0	4	0	2	1	2	2	3	2	1	2	0	2	1	3	1	0	1	1	1	1	0	2	1	1	2	4	0	0	1	0	3	1	4	2	4	1	7	2	0.800	0.728	–	–	W
Y	2	7	6	8	8	10	4	16	4	4	8	2	6	6	3	3	6	3	1	5	3	3	2	7	3	9	2	3	4	5	5	2	3	5	7	5	4	5	2	2	4	3	3	7	3	5	3	8	7	3	9	0.531	0.300	1.19E-01	n.s.	Y
total	127	123	143	176	125	150	103	170	128	118	143	137	127	143	146	129	131	145	126	134	130	122	115	136	130	131	126	133	120	126	116	128	148	105	141	107	120	139	141	147	133	120	110	122	148	125	124	135	160	148	224	0.590	0.067			total

Additional file 5. Continued.

NEMATODE x-x-S-x-V

position -4

	C50	C49	C48	C47	C46	C45	C44	C43	C42	C41	C40	C39	C38	C37	C36	C35	C34	C33	C32	C31	C30	C29	C28	C27	C26	C25	C24	C23	C22	C21	C20	C19	C18	C17	C16	C15	C14	C13	C12	C11	C10	C9	C8	C7	C6	C5	C4	C3	C2	C1	C0	C-index	SD	*P-value*	mark	
A	5	10	5	8	6	5	8	13	4	11	6	3	10	7	6	7	3	8	7	9	7	6	5	6	4	11	18	4	8	2	10	7	3	7	5	9	9	8	6	9	7	11	10	8	7	7	6	8	9	7	2	3.650	1.412	–	–	A
C	2	0	1	4	2	1	3	0	0	1	3	3	3	2	4	1	3	2	0	3	2	0	1	1	2	2	2	4	1	3	1	1	1	1	2	4	2	1	1	1	5	1	0	1	0	0	3	1	6	3	5	0.364	0.282	6.30E-07	*	C
D	7	6	5	4	9	5	4	1	4	4	3	4	4	0	4	3	5	6	5	11	5	6	8	10	3	4	4	6	7	4	4	2	5	6	4	5	2	7	11	12	5	2	6	6	3	2	7	2	1	6	6	0.830	0.429	–	–	D
E	7	8	5	5	1	5	5	4	4	3	10	7	7	5	5	5	4	2	3	7	7	9	9	3	1	5	4	6	3	8	9	3	4	6	4	7	13	2	10	3	7	5	5	8	3	7	6	6	4	4	8	0.683	0.310	–	–	E
F	6	6	4	3	5	1	3	3	2	7	4	3	8	2	3	4	3	3	5	2	2	2	4	3	3	6	4	9	7	5	3	0	2	4	3	6	4	6	2	7	5	5	2	2	5	3	6	8	2	3	7	0.571	0.283	1.46E-01	n.s.	F
G	6	5	9	8	5	5	4	5	2	4	8	8	6	5	7	3	10	5	5	1	3	6	5	4	5	8	3	5	14	8	8	4	4	3	7	7	9	3	4	5	3	5	7	7	8	11	3	3	4	4	4	1.405	0.625	–	–	G
H	3	0	4	1	4	2	2	0	2	2	0	2	1	0	1	4	1	5	2	2	2	4	5	2	5	2	3	0	1	3	4	3	5	1	1	1	1	2	0	0	3	1	3	2	1	5	2	3	4	2	5	0.436	0.304	8.98E-04	n.s.	H
I	5	5	10	11	5	4	5	9	12	4	3	6	8	8	3	6	9	8	5	3	6	4	12	9	9	10	10	4	9	8	7	6	3	10	2	12	9	4	6	9	4	4	7	7	8	8	3	7	8	6	9	0.756	0.299	–	–	I
K	7	2	1	1	2	7	0	3	6	4	8	2	9	5	5	5	6	5	5	5	7	8	7	4	4	3	9	4	6	2	7	6	12	7	15	10	9	12	8	7	7	7	11	4	9	8	8	11	5	6	20	0.311	0.156	5.55E-08	*	K
L	9	8	13	7	15	9	8	7	5	6	9	11	7	7	8	12	8	7	6	4	7	12	11	3	8	12	7	6	10	8	5	11	9	7	12	5	8	5	7	6	10	11	10	11	8	10	8	8	4	6	10	0.822	0.257	–	–	L
M	3	2	0	1	2	4	3	0	0	3	5	4	3	5	3	2	4	5	1	1	3	4	4	1	5	2	0	0	3	5	3	1	1	2	4	1	4	4	0	3	1	3	2	1	0	5	3	6	4	1	3	0.847	0.564	–	–	M
N	3	9	3	5	4	7	2	4	3	3	5	5	8	5	3	2	0	7	4	2	8	4	5	5	1	8	5	4	7	5	0	6	5	7	7	10	5	6	5	4	10	7	3	8	5	2	7	10	4	7	8	0.635	0.306	1.00E+00	n.s.	N
P	7	2	1	4	2	3	4	3	2	3	4	9	3	8	4	5	1	4	4	4	5	6	4	3	4	5	4	5	7	6	4	5	3	5	0	5	5	7	6	3	7	3	8	8	6	14	3	7	4	1	9	0.511	0.269	5.31E-04	n.s.	P
Q	4	3	2	3	18	5	7	3	4	2	5	4	4	3	1	2	2	2	1	1	3	3	4	2	2	3	2	3	6	1	2	3	2	5	5	3	3	4	11	4	4	5	3	4	4	1	2	2	3	3	7	0.509	0.387	1.68E-06	*	Q
R	7	3	4	2	7	6	1	6	2	1	5	3	0	3	7	3	6	5	5	4	4	3	6	2	6	5	7	5	7	8	2	2	5	5	6	4	2	3	10	8	5	7	16	8	3	10	12	13	6	7	14	0.381	0.224	5.56E-08	*	R
S	12	6	4	10	3	6	8	8	12	8	3	8	10	9	10	15	10	13	8	8	8	6	8	9	12	9	7	9	7	8	11	8	7	11	4	10	9	6	5	12	10	10	4	8	12	11	10	12	10	11	7	1.243	0.381	–	–	S
T	5	8	7	3	4	8	7	4	13	5	11	9	10	6	9	5	6	5	11	8	6	6	8	3	8	4	8	9	5	4	2	8	12	3	8	8	8	6	4	9	1	6	10	3	3	5	6	11	5	10	9	0.740	0.308	–	–	T
V	5	4	4	8	11	6	4	6	6	5	8	2	4	11	9	8	8	9	7	10	1	9	6	10	7	5	5	3	4	1	6	8	5	5	12	7	8	9	18	10	8	6	5	9	4	3	9	6	5	8	16	0.421	0.191	5.57E-08	*	V
W	2	1	1	1	1	0	0	1	0	0	2	1	2	1	0	1	0	3	2	2	0	0	1	2	2	1	1	1	2	0	0	0	0	0	2	2	0	1	0	1	1	0	1	0	2	2	1	4	3	2	1	1.060	0.978	–	–	W
Y	3	1	2	2	2	2	2	2	3	5	1	4	4	3	0	3	1	2	1	4	3	1	1	2	3	1	1	4	5	3	3	2	3	6	2	2	0	0	4	4	1	5	4	1	2	1	6	2	4	1	3	0.827	0.505	–	–	Y
total	108	89	85	91	108	91	80	82	86	81	103	98	111	95	92	96	90	106	87	91	89	99	114	84	94	106	104	91	119	92	91	86	91	101	105	118	110	96	118	117	104	104	117	106	93	115	111	130	95	98	153	0.649	0.077			total

position -3

	C50	C49	C48	C47	C46	C45	C44	C43	C42	C41	C40	C39	C38	C37	C36	C35	C34	C33	C32	C31	C30	C29	C28	C27	C26	C25	C24	C23	C22	C21	C20	C19	C18	C17	C16	C15	C14	C13	C12	C11	C10	C9	C8	C7	C6	C5	C4	C3	C2	C1	C0	C-index	SD	*P-value*	mark	
A	7	9	5	8	7	4	4	5	9	8	7	6	4	3	6	7	10	7	1	7	9	3	6	5	5	4	7	10	5	3	3	3	4	2	4	12	12	4	2	11	6	3	4	5	8	6	9	10	9	4	5	1.208	0.544	–	–	A
C	5	0	1	0	3	1	3	3	2	0	1	3	0	1	1	0	2	3	1	2	3	0	2	1	0	3	2	1	2	2	1	2	2	2	1	1	1	0	3	3	4	2	1	2	1	3	1	1	4	0	2	0.830	0.611	–	–	C
D	4	8	4	3	5	6	3	6	4	3	1	5	6	5	10	3	2	5	4	6	2	3	5	5	4	7	9	7	7	7	5	6	5	5	3	6	4	7	5	3	3	4	7	5	5	5	4	3	1	1	16	0.295	0.122	5.55E-08	*	D
E	3	4	1	5	4	8	8	5	1	6	2	3	4	4	3	5	7	4	5	7	7	4	13	5	5	4	5	5	4	3	6	8	5	2	4	3	2	2	9	4	8	2	10	3	3	4	3	4	10	4	12	0.400	0.206	6.05E-08	*	E
F	5	2	4	1	3	3	7	5	4	2	2	4	3	5	7	10	2	4	2	9	3	7	1	2	7	7	6	4	5	5	5	4	3	5	5	7	5	7	4	8	7	5	6	4	4	5	7	10	6	8	6	0.820	0.367	–	–	F
G	7	10	2	9	3	5	4	5	3	6	7	4	7	4	3	6	4	4	2	5	7	5	6	8	8	5	8	8	5	2	3	3	8	6	4	14	5	4	5	4	9	6	9	6	1	2	3	5	3	3	6	0.883	0.418	–	–	G
H	2	2	0	2	4	4	1	2	2	1	5	4	5	3	4	2	4	1	1	2	3	0	2	1	0	2	2	4	1	2	0	1	1	1	0	4	2	4	1	0	5	1	1	1	4	4	4	4	4	1	5	0.444	0.308	4.18E-02	n.s.	H
I	8	7	3	6	4	7	6	7	5	3	10	6	3	7	10	7	8	4	7	5	8	7	3	10	10	10	6	3	7	0	4	7	10	6	13	10	4	8	2	2	8	12	6	10	11	6	11	13	2	9	10	0.682	0.308	–	–	I
K	11	6	5	5	6	5	4	1	4	4	6	7	6	4	3	2	2	9	3	7	5	3	7	6	6	6	5	2	5	7	6	3	5	11	14	5	10	3	4	6	4	5	5	10	5	9	7	6	3	5	10	0.556	0.256	2.89E-03	n.s.	K
L	3	6	11	8	9	7	4	5	10	11	13	6	12	6	11	12	9	12	11	3	5	15	9	4	5	8	9	5	13	16	10	7	7	10	3	7	7	3	11	6	2	14	9	6	10	10	9	11	11	10	6	1.403	0.564	–	–	L
M	1	2	2	2	4	5	3	1	2	1	5	2	1	2	1	4	0	3	1	1	0	3	4	5	4	5	2	6	5	0	2	2	3	0	2	3	4	2	5	0	0	2	5	1	1	1	2	3	2	2	2	1.190	0.820	–	–	M
N	11	7	5	5	3	3	1	3	3	5	5	5	4	8	1	2	8	9	6	3	4	4	5	5	6	4	6	4	5	7	5	5	5	5	2	3	5	5	3	5	10	6	4	6	9	10	5	3	3	8	11	0.462	0.205	4.53E-07	*	N
P	4	5	11	4	2	5	4	4	4	3	5	9	10	5	5	6	7	9	11	6	6	4	4	2	3	3	5	12	5	3	7	6	6	6	4	9	6	8	10	8	4	2	5	5	3	9	5	8	4	5	8	0.715	0.313	–	–	P
Q	3	2	1	2	7	1	2	2	6	3	5	4	6	4	1	5	2	4	5	3	2	5	1	5	0	3	3	6	7	7	2	3	4	4	8	2	3	1	3	2	1	4	1	7	1	5	5	2	6	2	8	0.433	0.253	7.85E-05	*	Q
R	9	3	4	5	3	6	3	5	7	6	5	3	6	5	10	7	3	4	2	4	6	7	5	3	5	4	6	2	3	2	3	6	4	6	9	3	6	7	4	8	5	12	5	7	4	9	8	9	6	7	7	0.774	0.323	–	–	R
S	13	2	8	12	21	7	12	11	6	7	5	10	12	5	5	11	8	10	9	7	9	8	23	5	15	15	5	4	16	8	15	6	8	14	17	12	17	13	18	14	10	6	16	9	12	7	9	13	9	8	11	0.949	0.412	–	–	S
T	3	10	8	7	5	6	4	2	5	3	7	5	13	6	3	2	8	8	9	6	3	9	5	4	4	6	6	4	5	7	7	6	3	7	3	6	8	4	10	10	6	4	8	10	5	8	8	14	3	9	11	0.567	0.246	1.47E-01	n.s.	T
V	4	1	6	5	6	6	4	3	6	4	5	6	5	11	4	4	3	4	4	5	5	9	9	6	5	6	7	2	7	8	5	5	6	8	6	9	5	13	13	21	11	11	12	6	6	11	9	8	3	7	13	0.515	0.267	1.55E-04	n.s.	V
W	1	1	2	0	2	0	2	2	0	1	3	2	3	2	2	1	0	0	1	1	1	1	1	1	2	2	1	0	1	0	1	1	1	1	0	0	0	1	4	0	0	2	0	0	0	1	0	0	1	1	2	0.500	0.474	5.69E-04	n.s.	W
Y	4	2	2	2	7	2	1	5	3	4	4	4	1	5	2	0	1	2	2	2	1	2	3	1	0	2	4	2	11	3	1	2	1	0	3	2	4	0	2	2	1	1	3	3	0	0	2	3	5	4	2	1.230	0.986	–	–	Y
total	108	89	85	91	108	91	80	82	86	81	103	98	111	95	92	96	90	106	87	91	89	99	114	84	94	106	104	91	119	92	91	86	91	101	105	118	110	96	118	117	104	104	117	106	93	115	111	130	95	98	153	0.649	0.077			total

position -1

	C50	C49	C48	C47	C46	C45	C44	C43	C42	C41	C40	C39	C38	C37	C36	C35	C34	C33	C32	C31	C30	C29	C28	C27	C26	C25	C24	C23	C22	C21	C20	C19	C18	C17	C16	C15	C14	C13	C12	C11	C10	C9	C8	C7	C6	C5	C4	C3	C2	C1	C0	C-index	SD	*P-value*	mark	
A	11	8	4	8	22	3	6	9	6	13	6	5	9	11	5	6	7	5	6	4	7	4	11	11	9	9	8	7	10	10	2	8	7	7	7	10	11	3	6	6	7	7	1	14	8	15	8	4	7	4	9	0.849	0.401	–	–	A
C	1	3	1	0	3	4	2	1	2	0	0	2	2	2	0	2	0	1	2	1	2	2	1	0	3	2	0	1	2	1	1	0	0	1	1	3	1	0	1	1	0	1	1	2	2	0	2	0	1	1	0	–	–	–	–	C
D	4	5	6	7	8	6	6	4	4	5	5	4	10	6	3	8	6	3	3	5	12	7	6	6	6	8	2	4	9	7	4	4	11	7	8	3	6	3	2	3	3	6	12	4	2	2	6	5	7	4	11	0.504	0.225	5.76E-05	*	D
E	4	4	1	4	8	7	2	1	5	4	7	9	2	8	5	6	5	8	6	11	2	6	9	2	5	6	3	6	3	10	4	4	4	7	3	8	7	4	7	5	8	1	6	8	9	10	0	4	2	3	7	0.751	0.384	–	–	E
F	3	7	6	5	2	10	5	3	6	4	8	5	7	4	5	4	7	6	5	7	2	6	6	2	2	5	2	4	6	8	5	4	9	4	6	5	7	6	9	7	6	6	3	4	4	7	9	8	1	4	13	0.409	0.161	5.55E-08	*	F
G	7	4	8	2	4	7	6	4	3	7	4	7	7	4	2	3	6	4	3	4	7	8	4	7	1	9	3	6	7	4	6	5	5	4	5	4	4	7	4	4	5	10	3	3	2	2	2	6	5	3	6	0.803	0.338	–	–	G
H	4	2	3	2	1	1	1	1	1	0	0	2	3	2	1	3	4	3	4	1	3	3	4	2	3	0	3	0	6	4	1	1	2	1	5	5	2	2	5	2	5	0	6	1	4	2	2	4	3	7	3	0.847	0.576	–	–	H
I	4	3	7	8	11	5	3	6	5	2	6	7	5	9	6	1	5	11	2	5	5	5	4	7	5	11	11	4	16	7	6	5	2	7	4	5	3	1	8	8	4	7	7	7	3	17	12	8	6	5	13	0.478	0.258	2.72E-06	*	I
K	4	4	3	4	2	7	5	4	6	6	2	6	2	4	4	6	5	5	5	5	5	4	4	5	5	4	8	5	7	6	6	3	9	6	7	4	4	6	6	9	5	7	11	2	1	6	4	9	5	6	3	1.720	0.658	–	–	K
L	5	5	5	6	6	3	6	8	9	4	6	10	11	7	10	8	6	10	8	4	4	7	8	1	7	4	6	3	10	6	9	10	6	8	4	8	11	8	9	6	5	2	7	6	5	6	6	14	8	14	15	0.460	0.182	1.22E-07	*	L
M	2	2	1	2	1	1	2	5	0	0	8	3	3	3	1	1	1	0	0	3	1	1	1	2	2	1	5	1	3	0	0	2	0	0	3	2	3	1	3	4	6	0	0	3	1	1	3	1	3	0	2	0.920	0.853	–	–	M
N	3	1	1	6	3	2	1	5	2	4	8	1	5	2	3	2	5	4	4	7	2	3	5	5	4	1	5	7	2	5	7	3	6	7	3	5	6	3	3	3	4	5	4	5	3	4	4	4	4	5	12	0.327	0.148	5.55E-08	*	N
P	7	5	7	6	6	8	6	8	3	7	3	5	7	3	8	6	2	8	7	4	4	4	4	4	2	6	6	3	4	2	4	7	1	5	5	4	3	5	8	6	4	5	8	7	5	3	6	9	7	11	11	0.487	0.191	5.78E-05	*	P
Q	9	3	2	4	2	4	1	2	3	2	4	4	5	1	1	2	5	6	2	3	1	1	1	2	5	3	5	4	6	6	3	3	4	1	5	10	5	2	2	7	6	4	2	0	5	3	4	4	2	4	7	0.500	0.296	2.62E-03	n.s.	Q
R	12	2	3	2	5	2	3	1	5	3	6	5	5	3	8	3	6	4	6	5	7	6	5	3	10	7	13	4	3	1	8	10	6	3	7	7	5	7	6	6	6	9	7	7	11	6	9	10	8	3	9	0.642	0.312	1.00E+00	n.s.	R
S	10	9	6	8	3	4	8	12	10	6	9	6	13	9	8	11	9	12	8	6	9	12	15	11	10	12	6	10	6	3	9	11	6	11	10	13	9	11	12	12	13	16	11	13	10	11	8	16	11	7	11	0.875	0.272	–	–	S
T	7	13	14	9	9	9	8	1	7	10	13	10	7	13	13	6	6	8	6	7	7	7	10	3	7	8	11	7	4	6	8	2	5	8	14	7	9	15	15	14	8	8	13	8	5	13	16	12	6	7	9	0.976	0.390	–	–	T
V	7	7	5	2	6	4	7	4	5	3	7	3	5	2	6	14	3	5	4	6	6	7	12	9	2	5	4	6	4	4	5	2	6	10	7	12	9	7	10	11	6	8	12	9	7	5	8	9	4	9	7	0.914	0.412	–	–	V
W	3	1	2	2	1	2	0	0	2	0	1	0	1	0	0	3	0	1	0	1	0	0	2	0	3	1	1	2	3	0	2	0	0	2	1	0	1	0	2	0	0	0	1	0	2	1	0	1	1	0	2	0.460	0.493	2.23E-03	n.s.	W
Y	1	1	0	4	5	2	2	3	2	1	0	4	2	2	3	1	2	2	6	2	3	6	2	2	3	4	2	7	8	2	1	2	2	2	0	3	4	5	0	3	3	2	2	3	4	1	2	2	4	1	3	0.867	0.579	–	–	Y
total	108	89	85	91	108	91	80	82	86	81	103	98	111	95	92	96	90	106	87	91	89	99	114	84	94	106	104	91	119	92	91	86	91	101	105	118	110	96	118	117	104	104	117	106	93	115	111	130	95	98	153	0.649	0.077			total

Additional file 5. Continued.

NEMATODE x-x-T-x-I

position -4

	C50	C49	C48	C47	C46	C45	C44	C43	C42	C41	C40	C39	C38	C37	C36	C35	C34	C33	C32	C31	C30	C29	C28	C27	C26	C25	C24	C23	C22	C21	C20	C19	C18	C17	C16	C15	C14	C13	C12	C11	C10	C9	C8	C7	C6	C5	C4	C3	C2	C1	C0	C-index	SD	*P-value*	mark	
A	4	5	7	5	1	5	2	6	5	6	6	10	9	3	5	6	8	1	1	3	6	1	2	4	8	3	8	3	9	1	5	6	3	1	6	4	5	5	4	6	3	6	4	7	4	4	2	1	2	2	2	2.230	1.183	–	–	A
C	0	1	1	2	1	1	4	2	4	1	2	2	0	0	1	2	2	1	1	1	4	0	4	3	1	2	0	6	0	0	1	0	0	1	3	1	1	1	1	1	0	3	1	3	0	1	0	3	0	3	4	0.365	0.347	5.85E-08	✻	C
D	3	2	2	2	1	5	5	4	4	2	3	4	4	4	2	2	2	3	4	3	2	3	5	5	1	1	5	4	6	4	9	1	0	3	5	2	2	2	3	0	1	4	5	2	1	4	6	6	5	2	4	0.800	0.449	1.00E+00	n.s.	D
E	0	8	2	3	4	3	3	1	3	3	2	5	4	6	2	2	6	4	2	5	4	7	5	2	7	8	3	4	4	2	1	3	7	1	1	2	4	4	3	2	8	5	2	6	2	7	6	1	1	4	8	0.460	0.265	6.60E-08	✻	E
F	5	4	1	5	0	2	3	3	4	10	6	1	5	5	3	6	6	3	8	2	2	5	3	3	1	7	3	3	3	5	1	5	2	1	3	2	2	6	3	4	3	2	2	3	5	3	4	1	1	3	1	3.460	2.002	–	–	F
G	7	6	7	3	3	1	5	1	3	4	2	2	3	3	2	3	7	1	2	3	3	3	4	3	3	1	2	3	1	5	1	4	1	0	3	5	1	2	4	2	3	4	2	3	2	5	5	1	1	3	4	0.740	0.422	2.78E-01	n.s.	G
H	2	1	1	0	0	0	1	1	2	2	0	3	3	2	0	0	1	0	0	0	1	1	0	0	2	1	2	1	1	1	0	2	0	0	0	2	2	0	2	3	0	2	0	4	0	1	0	0	1	4	2	0.520	0.562	9.36E-03	n.s.	H
I	3	9	7	11	3	6	4	5	4	11	4	8	5	6	4	4	5	7	9	12	6	2	5	4	8	2	5	5	5	4	2	3	6	6	5	4	5	1	4	5	7	3	4	4	12	13	7	3	8	4	6	0.930	0.459	–	–	I
K	4	6	8	18	1	4	2	1	5	3	5	0	4	4	2	1	3	4	9	2	2	1	3	4	1	4	11	6	5	11	5	3	7	3	2	9	6	5	4	6	4	3	4	1	3	9	8	11	3	3	8	0.583	0.422	2.67E-05	✻	K
L	10	10	8	4	5	13	10	9	7	8	7	10	6	8	4	8	8	6	9	6	6	5	8	7	11	8	11	3	11	4	6	7	6	5	6	3	6	1	4	7	7	2	10	9	7	10	8	1	1	5	3	2.273	0.930	–	–	L
M	0	2	1	2	1	2	1	3	2	2	1	2	5	1	1	1	2	2	0	1	1	3	2	3	1	4	1	3	1	1	2	5	1	3	2	1	2	4	3	2	1	2	2	1	2	0	1	1	2	2	0	–	–	–	–	M
N	3	6	2	6	9	3	2	9	6	4	5	4	7	4	5	5	4	11	1	3	0	3	3	4	6	5	3	1	5	4	1	4	3	8	3	4	5	2	1	1	3	5	3	3	2	0	5	6	5	4	7	0.574	0.327	7.21E-06	✻	N
P	3	3	3	1	6	0	2	1	3	4	3	7	1	3	2	2	5	2	3	3	1	2	3	0	0	2	3	2	1	4	0	1	5	1	3	7	3	11	0	2	4	1	2	1	1	3	2	1	3	7	5	0.532	0.429	3.45E-07	✻	P
Q	4	2	4	3	4	0	1	4	0	3	3	3	3	3	2	1	0	2	0	2	3	0	2	4	4	6	1	1	2	0	1	3	2	1	4	1	4	3	2	3	2	2	4	1	5	3	5	5	4	8	3	0.867	0.571	–	–	Q
R	2	5	4	0	2	4	5	7	1	1	3	3	3	1	5	5	5	4	1	5	6	10	3	0	1	3	4	4	9	5	1	6	2	2	7	4	1	6	1	7	5	2	4	5	3	1	4	4	1	4	7	0.517	0.323	4.63E-07	✻	R
S	3	4	5	4	5	12	6	5	2	6	3	2	5	4	5	6	11	4	9	5	4	5	5	10	5	8	6	8	0	1	3	4	11	5	5	8	5	2	4	6	8	1	4	8	8	5	8	3	3	7	2	2.660	1.319	–	–	S
T	6	11	2	5	2	7	1	2	5	8	3	5	2	6	5	5	2	1	1	3	2	3	5	3	3	8	6	4	3	5	4	6	4	5	3	11	5	3	2	2	5	3	7	2	2	3	3	1	12	3	8	0.525	0.321	1.26E-07	✻	T
V	2	5	6	4	11	6	4	2	4	7	4	4	6	2	2	1	7	1	3	5	3	5	10	7	3	6	6	6	4	2	3	4	3	8	0	5	3	3	5	3	6	5	2	6	5	6	6	5	5	4	7	0.643	0.310	4.76E-03	n.s.	V
W	1	1	1	0	2	3	0	1	1	0	2	0	2	1	0	0	2	1	2	0	0	1	0	0	0	0	1	1	0	1	1	0	1	0	0	2	1	0	1	1	0	0	0	0	0	0	3	1	1	0	1	0.720	0.834	1.00E+00	n.s.	W
Y	1	2	4	1	2	1	2	3	4	1	2	5	3	1	1	3	4	1	4	2	1	0	3	3	1	3	2	4	3	1	1	5	2	3	4	3	1	2	4	4	2	1	3	2	4	1	5	3	1	2	3	0.807	0.437	1.00E+00	n.s.	Y
total	63	93	76	79	63	78	63	70	69	86	66	80	80	67	53	63	90	59	69	66	57	60	75	69	67	82	83	72	73	61	48	72	66	57	65	80	64	63	55	67	72	56	65	71	68	79	88	58	60	74	85	0.814	0.118			total

position -3

	C50	C49	C48	C47	C46	C45	C44	C43	C42	C41	C40	C39	C38	C37	C36	C35	C34	C33	C32	C31	C30	C29	C28	C27	C26	C25	C24	C23	C22	C21	C20	C19	C18	C17	C16	C15	C14	C13	C12	C11	C10	C9	C8	C7	C6	C5	C4	C3	C2	C1	C0	C-index	SD	*P-value*	mark	
A	5	7	4	3	4	4	3	4	5	5	4	8	1	3	4	3	8	6	8	4	5	3	10	4	5	4	7	6	4	4	4	4	2	6	4	9	4	3	5	4	6	3	5	3	4	5	7	4	5	4	4	1.180	0.446	–	–	A
C	0	7	2	2	2	2	3	1	0	0	0	0	2	0	1	0	1	2	2	2	0	0	1	2	1	0	3	1	3	1	0	2	0	0	4	1	1	1	2	1	6	0	1	0	0	1	0	0	0	1	2	0.620	0.746	2.90E-02	n.s.	C
D	1	7	6	1	7	1	0	5	2	1	3	1	3	1	2	7	4	1	4	2	5	4	6	1	1	0	5	2	1	3	2	1	4	0	3	1	0	4	0	4	2	2	3	1	5	4	5	3	3	2	2	1.360	0.995	–	–	D
E	3	2	4	3	3	7	4	6	4	1	4	5	7	2	6	4	4	1	4	2	1	6	5	5	4	6	3	3	1	3	3	4	4	0	4	1	7	2	1	3	7	1	2	9	7	5	4	1	5	3	3	1.240	0.680	–	–	E
F	1	6	5	4	3	3	4	6	1	4	2	2	5	3	3	3	2	3	4	2	3	3	3	2	5	0	3	5	3	2	2	2	5	3	0	13	1	6	2	5	4	5	6	3	6	4	4	1	2	6	2	1.750	1.061	–	–	F
G	3	3	3	2	1	0	5	5	5	4	1	1	2	6	1	5	5	0	4	6	4	4	8	5	5	3	9	3	2	2	3	8	2	2	6	6	2	2	4	3	1	2	0	1	0	1	4	4	6	9	3	1.153	0.774	–	–	G
H	0	1	0	1	1	4	1	1	1	3	1	1	0	5	1	0	4	0	1	1	1	2	2	0	0	1	0	1	1	1	0	1	1	4	3	1	1	1	0	3	0	0	1	2	1	2	3	0	0	0	2	0.600	0.623	6.72E-05	✻	H
I	4	9	7	4	1	5	7	7	2	9	4	7	8	2	3	1	12	3	4	8	3	7	8	3	5	3	5	3	4	4	2	3	8	6	3	4	6	8	2	2	5	6	4	6	4	2	4	7	2	5	6	0.803	0.405	9.34E-01	n.s.	I
K	6	5	2	20	5	7	3	0	3	11	5	6	4	3	1	3	8	5	6	4	7	3	2	3	3	5	7	3	7	5	3	2	8	3	5	3	3	3	5	2	7	2	5	4	5	4	6	4	2	3	4	1.155	0.759	–	–	K
L	8	9	5	9	9	3	8	7	6	10	3	7	3	5	8	8	7	4	7	7	7	7	5	7	4	6	4	5	4	8	2	8	3	10	2	3	5	2	5	4	5	5	10	6	5	9	9	8	2	4	2	2.970	1.175	–	–	L
M	2	1	1	3	3	3	0	1	1	1	5	0	5	0	0	2	4	9	0	1	0	2	2	2	1	1	2	1	0	0	3	0	1	0	1	6	1	3	1	1	1	1	0	0	2	2	1	0	3	3	1	1.660	1.791	–	–	M
N	5	4	3	3	0	6	2	2	4	4	1	6	4	2	4	4	5	1	2	2	3	3	6	3	4	7	2	2	7	3	4	5	5	4	4	7	7	4	3	4	3	4	4	1	2	4	2	1	3	3	6	0.593	0.282	7.80E-06	✻	N
P	1	3	5	3	1	4	2	2	6	6	9	5	3	1	1	5	3	3	1	2	4	1	2	4	7	4	4	4	3	2	0	3	2	2	3	7	6	2	0	3	4	4	2	2	1	0	8	2	7	5	5	0.656	0.426	2.49E-02	n.s.	P
Q	1	3	2	0	5	4	3	2	0	6	2	3	2	5	4	2	2	2	2	2	1	1	2	1	2	2	4	1	8	2	3	0	3	2	2	2	2	3	4	2	3	4	2	3	4	5	0	1	6	1	10	0.256	0.166	5.55E-08	✻	Q
R	7	5	3	2	4	4	1	4	5	5	1	3	7	6	1	2	0	2	4	3	3	2	2	5	4	6	4	6	8	3	1	7	4	2	0	2	2	4	3	3	0	5	2	6	0	1	3	4	1	3	2	1.650	1.016	–	–	R
S	5	13	9	10	7	5	2	7	8	3	8	9	10	12	4	7	11	5	5	7	5	7	4	9	4	9	6	8	3	7	7	5	5	5	7	2	7	3	5	6	8	5	4	11	8	9	6	10	7	6	6	1.117	0.425	–	–	S
T	4	1	9	3	2	5	5	1	6	5	8	11	8	5	3	6	5	5	4	5	2	1	2	4	7	8	6	6	5	3	6	6	2	5	3	2	5	5	7	12	4	2	4	6	3	4	4	2	3	5	9	0.522	0.266	5.88E-08	✻	T
V	6	4	2	4	5	6	6	7	6	4	2	1	4	3	4	0	2	5	3	3	1	2	3	4	3	10	7	6	5	5	1	8	4	2	10	9	2	3	4	4	4	5	9	7	9	5	10	4	1	10	13	0.360	0.205	5.55E-08	✻	V
W	0	0	1	0	0	1	0	0	0	2	1	2	0	1	2	0	2	1	0	0	1	1	0	0	1	3	0	5	0	1	1	2	1	0	0	0	1	1	1	1	1	0	1	0	1	2	6	1	0	0	0	–	–	–	–	W
Y	1	3	3	2	0	4	4	2	4	2	2	2	2	2	0	1	1	1	4	3	1	1	2	5	1	4	2	1	4	2	1	1	2	1	1	1	1	3	1	0	1	0	0	0	1	10	2	1	2	1	3	0.640	0.571	3.72E-04	n.s.	Y
total	63	93	76	79	63	78	63	70	69	86	66	80	80	67	53	63	90	59	69	66	57	60	75	69	67	82	83	72	73	61	48	72	66	57	65	80	64	63	55	67	72	56	65	71	68	79	88	58	60	74	85	0.814	0.118			total

position -1

	C50	C49	C48	C47	C46	C45	C44	C43	C42	C41	C40	C39	C38	C37	C36	C35	C34	C33	C32	C31	C30	C29	C28	C27	C26	C25	C24	C23	C22	C21	C20	C19	C18	C17	C16	C15	C14	C13	C12	C11	C10	C9	C8	C7	C6	C5	C4	C3	C2	C1	C0	C-index	SD	*P-value*	mark	
A	5	9	4	3	3	8	5	7	5	4	9	4	3	3	8	3	11	1	6	6	2	4	0	6	4	4	6	3	6	2	3	2	6	3	5	5	0	2	5	3	6	3	3	5	3	4	0	1	5	5	8	0.533	0.292	1.65E-07	✻	A
C	5	2	0	2	2	2	2	1	1	1	2	1	5	0	0	1	1	1	4	1	2	0	2	1	3	4	0	1	0	2	0	3	0	1	0	1	1	0	3	1	2	0	0	1	0	0	1	0	4	0	0	–	–	–	–	C
D	3	4	5	2	5	4	1	2	3	3	5	5	3	2	2	3	7	2	1	2	4	4	8	3	1	4	0	3	9	1	3	3	1	3	1	2	4	2	3	5	11	2	1	3	0	4	7	2	1	1	2	1.600	1.120	–	–	D
E	2	8	5	3	2	0	1	5	5	5	6	4	3	1	6	5	2	2	8	3	4	3	4	4	6	5	5	7	4	6	3	7	4	4	4	3	4	6	4	4	5	4	5	2	3	6	7	2	3	3	5	0.828	0.359	–	–	E
F	1	5	1	3	3	0	3	0	0	1	1	6	4	2	2	1	3	0	1	3	4	7	3	1	2	1	2	3	5	2	3	4	3	3	1	7	3	7	1	1	3	1	3	3	4	3	1	4	1	1	5	0.508	0.362	3.60E-07	✻	F
G	4	2	5	4	2	3	6	0	5	3	7	6	4	6	3	4	2	11	5	0	2	2	6	4	4	1	4	1	4	1	0	2	4	6	2	5	6	4	1	3	4	1	1	5	2	3	9	4	4	1	6	0.593	0.377	3.09E-04	n.s.	G
H	0	1	3	4	0	5	1	1	0	4	2	4	0	0	0	0	0	1	0	2	0	1	2	1	0	2	1	1	1	0	0	2	0	0	0	2	1	0	3	0	2	0	0	0	1	1	1	1	0	1	2	0.520	0.639	2.31E-04	n.s.	H
I	4	9	10	5	6	7	6	6	6	7	1	3	8	5	3	2	4	6	5	4	4	2	2	8	6	7	9	7	8	7	2	6	4	2	4	7	3	5	6	2	1	6	3	4	5	5	8	2	3	7	2	2.520	1.134	–	–	I
K	5	6	5	3	0	2	2	6	5	3	4	4	6	4	3	4	7	6	1	6	2	5	3	0	1	5	8	2	3	6	3	3	4	6	4	1	5	3	2	4	5	3	4	6	7	14	7	7	1	5	7	0.603	0.345	5.04E-04	n.s.	K
L	5	6	5	6	5	10	3	6	11	10	5	4	8	5	4	6	11	6	9	6	5	4	5	2	9	8	7	7	5	4	7	6	6	2	6	3	3	5	2	8	5	6	9	3	4	7	6	5	1	8	8	0.723	0.292	1.13E-01	n.s.	L
M	2	2	1	5	1	4	2	4	0	4	3	2	2	3	2	2	2	1	2	0	3	1	1	3	0	5	0	1	3	3	1	3	0	0	1	2	1	1	0	3	2	6	1	2	1	0	0	1	1	3	1	1.860	1.457	–	–	M
N	5	1	3	0	2	1	5	4	2	5	4	1	1	5	2	3	3	2	3	0	2	4	4	2	3	1	4	5	2	3	2	3	5	3	1	3	2	5	2	3	2	4	2	0	8	7	3	2	3	4	2	1.460	0.844	–	–	N
P	5	4	1	4	8	8	3	4	4	7	4	4	5	7	1	2	4	0	2	6	4	1	5	4	2	4	3	4	2	1	5	1	6	2	7	4	8	5	1	5	5	2	6	8	6	2	5	3	9	5	4	1.040	0.554	–	–	P
Q	1	7	2	2	3	0	3	0	1	0	2	4	5	1	1	0	2	3	2	5	2	2	1	3	7	6	6	4	1	4	1	1	2	3	0	4	1	1	1	4	2	3	7	3	2	1	4	2	2	1	6	0.417	0.316	5.83E-08	✻	Q
R	3	0	0	20	2	5	0	7	8	6	0	2	6	2	4	1	3	4	0	6	1	1	7	6	4	4	7	5	4	3	2	2	3	2	8	5	3	2	2	5	2	2	4	0	4	3	6	4	5	3	8	0.470	0.402	6.11E-08	✻	R
S	5	7	5	4	5	2	6	4	4	4	3	7	7	7	4	6	9	3	6	4	4	5	11	5	3	4	7	2	3	3	5	7	4	1	7	7	6	6	7	7	6	4	5	9	3	5	7	5	3	15	5	1.072	0.481	–	–	S
T	4	7	11	4	1	7	2	4	3	7	3	12	2	7	3	9	6	4	4	3	5	5	3	6	5	4	6	5	5	10	2	8	6	6	4	6	3	4	7	5	3	3	6	7	5	4	9	2	5	3	4	1.275	0.596	–	–	T
V	1	7	3	3	6	6	7	6	5	6	3	4	8	4	3	10	4	6	7	7	6	6	5	6	6	10	8	8	4	1	5	4	6	5	4	13	7	3	5	2	4	4	4	8	4	7	3	7	6	7	4	1.370	0.572	–	–	V
W	2	3	3	0	3	0	2	2	1	3	1	0	0	0	0	0	5	0	2	0	1	0	2	2	1	3	0	0	1	1	1	2	1	0	0	0	1	0	0	1	1	2	0	0	0	0	2	2	2	1	1	1.080	1.175	–	–	W
Y	1	3	4	2	4	4	3	1	0	3	1	3	0	3	2	1	4	0	1	2	0	3	1	2	0	0	0	3	3	1	0	3	1	5	6	0	2	2	0	1	1	0	1	2	6	3	2	2	1	0	5	0.372	0.321	5.56E-08	✻	Y
total	63	93	76	79	63	78	63	70	69	86	66	80	80	67	53	63	90	59	69	66	57	60	75	69	67	82	83	72	73	61	48	72	66	57	65	80	64	63	55	67	72	56	65	71	68	79	88	58	60	74	85	0.814	0.118			total

Additional file 5. Continued.

NEMATODE x-x-T-x-L

position -4

	C50	C49	C48	C47	C46	C45	C44	C43	C42	C41	C40	C39	C38	C37	C36	C35	C34	C33	C32	C31	C30	C29	C28	C27	C26	C25	C24	C23	C22	C21	C20	C19	C18	C17	C16	C15	C14	C13	C12	C11	C10	C9	C8	C7	C6	C5	C4	C3	C2	C1	C0	C-index	SD	*P-value*	mark	
A	7	3	3	4	7	5	7	6	3	6	6	6	4	7	4	7	5	3	4	4	8	10	6	4	5	3	10	4	7	6	7	5	6	3	12	2	6	5	6	3	7	4	3	3	5	2	1	3	5	6	5	1.032	0.434	−	−	A
C	0	0	0	0	0	2	1	4	2	0	2	5	3	2	4	1	2	1	1	1	3	1	5	1	0	1	1	1	3	2	5	1	3	1	2	3	1	5	3	4	0	1	2	4	0	3	1	0	1	3	4	0.460	0.383	7.86E-02	n.s.	C
D	4	1	4	6	4	3	9	3	11	7	3	5	4	6	5	3	3	5	4	4	5	8	2	3	5	7	7	3	12	1	3	4	3	6	3	3	2	2	4	7	5	2	6	3	5	5	6	3	2	3	8	0.560	0.287	6.39E-01	n.s.	D
E	8	4	5	6	5	2	3	6	3	5	8	5	2	5	4	2	4	5	5	8	10	7	4	9	21	3	8	4	6	2	5	7	3	3	4	4	5	4	3	8	4	5	4	6	4	4	7	14	7	3	13	0.420	0.248	4.60E-07	✻	E
F	1	3	4	3	8	3	11	6	7	2	9	9	1	5	6	5	6	5	3	9	1	5	6	1	2	5	5	7	5	7	5	3	6	1	3	5	3	1	3	6	9	10	5	3	6	4	4	5	8	1	4	1.205	0.648	−	−	F
G	4	4	2	3	1	4	1	2	5	3	4	3	3	3	3	5	7	5	4	3	6	3	8	4	4	8	2	4	3	3	2	6	5	5	4	2	4	6	3	6	3	4	5	7	5	6	2	3	5	3	6	0.667	0.276	−	−	G
H	5	5	1	3	0	4	4	0	0	0	2	0	1	0	2	1	2	0	3	0	3	3	5	0	2	1	1	4	2	1	0	1	0	3	2	3	6	1	1	3	0	2	1	0	2	1	2	1	4	2	3	0.600	0.539	−	−	H
I	6	6	4	10	7	18	7	3	10	4	2	5	5	1	4	8	8	9	5	12	9	2	5	4	4	9	5	4	3	2	9	9	7	6	10	3	8	4	4	7	9	5	4	5	9	8	5	10	5	6	14	0.449	0.221	2.51E-03	n.s.	I
K	4	4	3	6	4	4	10	4	4	10	5	5	12	2	2	6	7	7	4	5	3	4	2	6	5	2	3	8	5	1	5	5	4	5	7	3	5	4	3	6	5	4	7	5	4	9	6	2	12	6	9	0.564	0.269	3.93E-01	n.s.	K
L	6	5	14	10	8	9	8	8	8	12	8	11	10	8	15	5	7	10	11	12	4	9	5	13	3	13	11	13	5	6	8	10	7	7	8	7	6	8	8	8	8	9	14	2	9	5	8	6	7	6	13	0.643	0.224	−	−	L
M	1	2	2	3	2	3	4	3	1	0	0	2	2	2	3	2	3	4	2	1	0	2	5	6	5	1	3	4	1	2	0	1	3	1	1	1	2	3	3	2	1	0	0	2	6	2	2	1	1	1	6	0.347	0.247	1.32E-07	✻	M
N	5	2	3	7	8	6	5	4	7	6	2	14	8	4	7	4	6	3	6	3	6	7	6	4	4	9	3	5	8	4	6	3	3	6	4	3	3	4	6	2	3	7	3	4	4	2	7	2	6	2	9	0.547	0.257	8.92E-01	n.s.	N
P	7	2	7	6	4	1	3	4	5	6	2	5	4	6	5	7	4	6	10	5	3	6	3	1	4	6	4	6	5	0	7	1	6	5	5	1	4	4	5	4	7	1	3	8	5	5	5	5	3	5	7	0.646	0.290	−	−	P
Q	2	2	2	2	2	3	0	6	2	2	4	3	4	6	5	1	2	3	1	4	3	1	1	2	1	4	5	1	6	2	5	1	6	0	7	3	3	4	1	0	2	7	1	2	2	1	4	0	6	4	7	0.403	0.276	2.89E-04	n.s.	Q
R	4	7	1	13	5	4	4	10	5	12	6	3	5	6	9	9	2	5	3	4	8	3	6	2	4	7	3	3	7	3	4	3	1	8	7	7	7	3	3	4	5	5	1	6	3	8	0	4	2	2	9	0.547	0.310	5.09E-01	n.s.	R
S	8	7	9	4	4	7	8	5	10	11	13	11	8	4	7	6	8	8	6	12	6	9	7	3	9	5	6	6	7	12	4	15	11	5	6	6	12	6	13	12	12	5	8	12	6	6	8	7	5	12	16	0.496	0.184	2.06E-02	n.s.	S
T	7	8	5	10	7	5	8	3	6	8	7	8	7	12	9	5	9	8	4	5	6	2	8	12	0	5	5	6	3	11	7	5	10	2	7	6	5	8	4	7	5	10	4	4	3	3	4	3	7	3	12	0.510	0.222	4.48E-01	n.s.	T
V	7	4	10	3	7	6	8	9	7	8	7	5	9	3	5	8	6	3	10	4	1	8	4	9	5	3	6	7	6	6	12	4	6	5	8	7	5	5	4	2	6	8	4	9	9	3	12	5	15	4	9	0.704	0.307	−	−	V
W	0	1	0	0	1	1	2	0	1	0	0	0	1	2	0	0	7	1	0	3	1	0	1	1	0	2	0	2	0	2	3	0	3	1	0	1	0	1	0	1	2	0	1	1	0	0	1	1	1	1	0	−	−	−	−	W
Y	6	1	3	1	2	1	4	4	4	3	3	1	1	1	5	1	4	3	5	0	4	1	2	2	1	5	3	4	2	3	3	6	5	2	0	1	5	4	3	1	2	1	2	2	4	4	1	3	7	4	3	0.933	0.563	−	−	Y
total	92	71	82	100	86	91	107	90	101	105	93	106	94	85	104	86	102	94	91	99	90	91	91	87	84	99	91	96	96	76	100	90	98	75	100	71	92	82	80	93	95	90	78	88	91	81	86	78	109	77	157	0.578	0.060			total

position -3

	C50	C49	C48	C47	C46	C45	C44	C43	C42	C41	C40	C39	C38	C37	C36	C35	C34	C33	C32	C31	C30	C29	C28	C27	C26	C25	C24	C23	C22	C21	C20	C19	C18	C17	C16	C15	C14	C13	C12	C11	C10	C9	C8	C7	C6	C5	C4	C3	C2	C1	C0	C-index	SD	*P-value*	mark	
A	6	8	6	4	6	11	7	8	9	8	5	5	10	6	7	5	6	5	3	3	8	1	10	7	4	5	8	5	4	5	8	6	1	3	5	6	4	1	2	5	9	6	5	8	4	2	4	4	14	5	5	1.148	0.527	−	−	A
C	2	3	1	2	2	3	4	0	1	2	2	2	1	4	5	1	3	1	6	2	1	0	1	4	2	0	2	1	1	0	2	1	3	1	2	3	1	2	1	3	3	2	1	0	2	3	5	11	1	0	11	0.193	0.171	5.55E-08	✻	C
D	5	0	5	4	3	4	2	2	3	3	0	5	3	3	7	4	6	7	6	3	7	2	3	7	21	5	7	5	1	5	7	1	7	2	5	3	3	0	5	3	9	4	2	2	4	2	2	8	4	2	5	0.852	0.650	−	−	D
E	6	4	6	7	3	8	4	5	2	8	5	7	6	6	5	6	4	7	8	6	3	8	7	3	4	5	5	3	6	4	3	6	4	3	2	3	4	8	3	9	11	4	9	4	2	1	2	2	4	2	13	0.380	0.173	1.28E-07	✻	E
F	2	1	2	11	8	5	9	8	8	9	3	8	6	7	5	3	5	3	3	4	1	2	0	3	3	7	5	7	6	4	7	1	7	7	4	3	4	2	3	4	2	1	5	3	6	5	5	3	8	4	7	0.663	0.361	−	−	F
G	2	5	5	5	4	8	4	4	9	2	9	3	6	3	7	5	6	6	0	2	7	4	5	6	1	6	3	3	4	5	3	6	4	4	8	2	8	6	3	4	6	1	10	5	4	6	3	2	6	3	9	0.518	0.246	4.84E-01	n.s.	G
H	1	2	1	0	1	4	4	2	2	4	2	2	1	3	0	1	2	1	4	2	1	1	2	1	0	3	2	2	9	1	2	2	4	3	1	3	2	4	0	1	2	0	0	2	3	2	3	2	0	2	6	0.330	0.260	7.40E-08	✻	H
I	7	3	8	7	1	6	9	5	3	6	6	10	3	3	3	2	10	4	5	11	2	6	2	6	3	11	5	2	10	6	4	1	3	2	7	3	7	6	1	5	2	6	4	4	13	6	10	1	2	6	12	0.430	0.252	3.28E-04	n.s.	I
K	2	5	3	4	7	3	8	6	7	6	9	7	5	4	8	3	9	10	13	14	1	11	6	3	4	10	7	7	8	7	4	9	10	8	7	4	3	6	5	3	6	7	3	8	4	8	6	4	10	6	11	0.578	0.256	−	−	K
L	10	5	7	11	8	5	7	11	9	9	13	12	11	7	10	9	11	12	8	7	8	8	9	6	14	8	7	10	10	9	5	12	10	6	12	7	8	3	7	8	10	10	5	8	9	6	5	7	6	11	3	2.840	0.803	−	−	L
M	2	1	3	3	0	2	0	0	3	5	5	5	4	0	2	3	1	2	2	3	2	1	0	1	0	2	1	1	1	1	0	2	4	1	3	0	2	2	2	5	2	1	1	3	4	1	1	1	1	3	3	0.633	0.482	−	−	M
N	4	2	7	3	5	2	9	4	7	10	1	3	4	5	7	7	2	2	7	6	9	5	6	4	5	4	6	6	2	4	10	1	3	4	5	0	3	5	2	4	3	5	3	5	5	4	5	4	6	3	5	0.912	0.448	−	−	N
P	8	2	2	7	4	7	3	3	5	1	4	10	4	6	4	6	1	9	3	5	5	7	6	5	2	6	7	4	6	4	8	6	11	1	6	2	2	3	8	6	4	7	12	0	4	2	6	2	5	4	6	0.817	0.441	−	−	P
Q	2	3	4	2	0	5	5	1	4	3	3	5	6	0	4	3	3	4	4	5	7	0	4	3	2	0	4	9	4	2	5	5	2	5	2	5	3	6	4	6	1	4	4	6	3	6	4	2	7	5	8	0.465	0.244	4.68E-02	n.s.	Q
R	2	2	2	7	6	2	3	7	4	5	0	3	4	5	6	3	7	6	2	3	3	4	9	6	2	4	3	2	7	2	3	7	7	8	6	2	10	6	8	2	5	7	4	7	2	3	2	6	3	3	8	0.555	0.288	9.85E-01	n.s.	R
S	9	9	4	5	9	7	12	9	9	8	4	6	8	9	5	6	11	8	6	7	6	16	8	8	6	12	5	8	7	2	12	10	6	2	8	13	13	7	6	10	6	7	6	7	9	9	7	8	12	8	20	0.395	0.139	5.67E-08	✻	S
T	12	6	9	10	10	5	5	5	4	9	12	3	5	6	6	10	4	2	4	5	6	7	6	7	5	4	5	9	3	9	7	6	2	7	4	5	7	7	7	9	8	8	2	5	1	4	5	6	5	8	11	0.556	0.228	8.27E-01	n.s.	T
V	4	7	2	6	6	3	9	7	6	4	6	6	2	2	11	5	1	3	3	7	6	5	5	4	2	3	4	11	6	2	5	5	3	3	8	4	3	7	12	4	3	8	2	5	8	9	9	3	9	0	12	0.430	0.229	6.15E-04	n.s.	V
W	1	0	3	1	1	0	1	2	0	2	1	1	3	4	0	1	8	1	2	1	1	1	1	0	1	2	1	1	0	2	2	0	1	2	2	3	3	1	0	0	0	1	0	1	2	0	1	0	2	1	1	1.300	1.374	−	−	W
Y	5	3	2	1	2	1	2	1	6	1	3	3	2	2	2	3	2	1	2	3	6	2	1	3	3	2	4	0	1	2	3	3	6	3	3	0	2	0	1	2	3	1	0	5	2	2	1	2	4	1	1	2.300	1.488	−	−	Y
total	92	71	82	100	86	91	107	90	101	105	93	106	94	85	104	86	102	94	91	99	90	91	91	87	84	99	91	96	96	76	100	90	98	75	100	71	92	82	80	93	95	90	78	88	91	81	86	78	109	77	157	0.578	0.060			total

position -1

	C50	C49	C48	C47	C46	C45	C44	C43	C42	C41	C40	C39	C38	C37	C36	C35	C34	C33	C32	C31	C30	C29	C28	C27	C26	C25	C24	C23	C22	C21	C20	C19	C18	C17	C16	C15	C14	C13	C12	C11	C10	C9	C8	C7	C6	C5	C4	C3	C2	C1	C0	C-index	SD	*P-value*	mark	
A	4	4	6	6	1	5	5	2	9	8	12	6	4	4	7	8	12	9	10	1	4	5	1	8	3	3	4	6	5	1	7	7	7	3	1	9	8	3	4	5	3	6	4	2	3	3	2	1	7	4	2	2.520	1.414	−	−	A
C	2	2	2	0	2	2	1	2	3	5	0	0	1	1	3	3	5	0	1	3	2	1	2	0	1	0	1	3	1	1	2	0	2	4	2	4	2	1	2	2	4	2	1	0	1	6	1	1	3	1	5	0.364	0.282	4.55E-06	✻	C
D	5	1	7	3	2	4	4	7	3	3	4	2	3	2	6	4	4	7	4	5	0	4	6	6	6	4	4	6	3	5	7	7	5	4	7	1	3	6	5	7	8	1	6	7	3	0	3	10	3	4	9	0.491	0.240	1.85E-01	n.s.	D
E	6	2	4	5	4	1	3	5	5	6	6	4	5	6	8	2	5	3	4	3	12	9	7	7	2	8	5	5	6	3	5	8	10	6	4	4	6	3	4	5	8	2	7	1	6	7	3	5	5	2	17	0.296	0.135	5.55E-08	✻	E
F	5	6	2	9	9	8	6	7	6	3	6	5	4	6	2	3	4	6	5	6	2	4	3	4	3	8	2	6	4	3	8	6	0	4	3	5	4	4	2	6	6	6	8	1	7	5	5	3	5	5	10	0.480	0.206	9.16E-02	n.s.	F
G	10	4	1	3	2	6	5	4	7	6	2	3	3	4	4	6	5	1	9	6	2	2	4	4	5	5	0	5	2	6	4	2	10	2	5	3	6	4	3	10	5	5	3	3	3	2	8	1	14	2	4	1.105	0.685	−	−	G
H	2	1	5	1	1	4	4	2	2	1	3	6	6	0	4	3	1	0	1	2	2	5	1	1	4	4	2	2	3	2	3	6	1	1	1	1	3	1	0	2	2	0	3	0	2	2	2	3	2	2	5	0.448	0.317	6.29E-03	n.s.	H
I	4	8	4	11	5	9	9	7	7	10	6	3	13	11	8	7	8	11	7	9	5	7	5	1	6	5	4	5	8	7	6	6	4	2	7	6	3	6	4	6	9	7	4	7	11	6	9	7	8	3	10	0.662	0.255	−	−	I
K	4	1	7	2	7	3	5	4	6	8	4	6	3	9	6	6	4	10	8	13	3	11	5	2	4	2	13	6	3	9	3	1	2	5	4	5	4	6	6	6	6	5	4	6	8	9	8	4	6	10	15	0.376	0.189	1.12E-07	✻	K
L	8	2	8	11	10	7	11	8	10	9	4	10	6	11	8	6	10	7	6	10	10	11	12	8	4	9	13	12	18	5	8	6	12	7	5	7	6	11	10	8	5	13	9	12	8	3	8	7	5	7	10	0.842	0.298	−	−	L
M	0	1	4	1	4	0	2	2	3	2	1	6	4	4	6	2	2	5	1	3	1	0	2	4	1	4	1	1	3	0	3	0	3	1	3	1	1	1	2	1	4	2	3	3	0	2	3	0	2	4	3	0.727	0.525	−	−	M
N	2	9	2	4	4	7	4	4	3	5	1	2	6	2	4	7	4	4	2	4	8	2	4	3	20	4	6	5	4	2	9	1	4	5	7	1	5	3	4	7	5	3	4	5	8	2	5	6	5	2	9	0.509	0.335	4.49E-03	n.s.	N
P	7	2	2	11	4	6	12	5	2	3	14	6	6	3	4	2	3	4	7	4	4	4	7	6	5	6	3	5	4	4	1	0	2	11	10	5	8	4	6	3	4	8	2	4	4	7	4	4	6	2	7	0.714	0.411	−	−	P
Q	5	1	5	4	4	2	4	4	3	3	3	5	3	2	1	5	6	2	1	1	2	2	6	4	2	6	2	3	4	6	3	4	4	1	9	1	3	4	3	4	1	6	5	3	4	2	1	4	4	1	7	0.480	0.251	1.87E-02	n.s.	Q
R	4	0	4	5	3	6	9	4	1	7	4	4	6	3	4	1	5	5	5	6	6	4	3	3	3	5	2	3	2	2	4	8	4	4	6	2	5	3	1	4	1	2	3	7	4	2	1	5	2	5	15	0.256	0.129	5.55E-08	✻	R
S	12	7	7	6	6	9	4	8	12	10	11	9	8	7	4	6	11	8	7	9	15	5	6	8	6	9	6	7	5	6	8	11	6	6	13	5	6	9	4	6	9	7	3	10	5	8	8	9	14	9	8	0.975	0.330	−	−	S
T	1	6	6	5	4	4	5	7	8	10	4	12	5	5	8	9	1	6	1	5	6	3	6	6	6	5	7	3	7	6	8	6	11	6	3	7	10	6	6	5	6	8	3	6	5	7	4	0	9	7	7	0.829	0.355	−	−	T
V	7	6	4	10	10	6	10	6	6	4	4	13	5	4	12	4	9	4	7	6	5	8	9	10	1	9	10	7	9	3	6	9	6	3	3	2	4	5	9	6	7	6	2	8	5	5	10	6	6	4	11	0.582	0.246	−	−	V
W	1	2	0	0	0	1	2	2	2	0	1	2	1	0	1	2	2	1	4	1	0	1	0	1	0	2	5	2	2	1	1	0	1	0	3	1	2	1	1	0	0	0	1	1	1	1	0	0	0	1	0	−	−	−	−	W
Y	3	6	2	3	4	1	2	0	3	2	3	2	2	1	4	0	1	1	1	2	1	3	2	1	2	1	1	4	3	4	4	2	4	0	4	1	3	1	4	0	2	1	3	2	3	2	1	2	3	2	3	0.727	0.440	−	−	Y
total	92	71	82	100	86	91	107	90	101	105	93	106	94	85	104	86	102	94	91	99	90	91	91	87	84	99	91	96	96	76	100	90	98	75	100	71	92	82	80	93	95	90	78	88	91	81	86	78	109	77	157	0.578	0.060			total

Additional file 5. Continued.

NEMATODE x-x-T-x-V

position -4

	C50	C49	C48	C47	C46	C45	C44	C43	C42	C41	C40	C39	C38	C37	C36	C35	C34	C33	C32	C31	C30	C29	C28	C27	C26	C25	C24	C23	C22	C21
A	1	9	4	2	5	9	3	2	6	5	7	4	5	3	4	1	3	7	5	2	6	4	5	1	7	1	3	2	3	2
C	2	1	1	3	2	2	1	2	0	1	3	2	0	0	3	2	1	0	1	0	0	1	0	1	0	1	4	1	0	0
D	2	3	1	3	2	2	5	2	5	4	3	3	3	5	6	4	2	3	5	7	3	1	6	6	5	3	5	4	4	2
E	1	2	3	2	2	5	5	5	5	4	0	8	4	4	4	1	2	4	1	4	3	5	6	4	1	4	3	1	6	1
F	3	5	3	3	2	3	5	5	3	2	5	1	2	5	4	1	1	3	1	3	8	2	5	3	4	3	6	2	5	1
G	2	6	4	5	3	5	5	3	5	4	2	9	2	8	4	6	3	6	4	0	2	4	1	5	3	1	3	4	2	2
H	1	0	2	1	0	2	1	4	2	0	1	1	3	2	1	1	2	0	1	3	2	2	4	4	1	1	1	1	1	3
I	7	4	3	7	8	4	8	5	0	9	4	6	5	7	8	2	7	9	1	4	3	3	7	21	6	4	3	3	6	5
K	4	1	1	3	2	2	2	3	2	5	4	2	6	5	4	3	4	5	7	4	3	4	3	7	3	1	4	9	2	6
L	5	5	6	4	7	6	6	5	11	7	3	5	7	7	4	7	2	4	6	8	9	9	5	7	8	9	6	9	7	4
M	2	3	2	1	0	2	2	1	1	1	1	4	2	3	3	3	3	1	1	1	0	1	2	1	1	1	0	2	1	2
N	3	7	2	8	4	5	3	6	2	5	3	4	0	3	4	4	2	6	5	4	6	4	3	3	4	3	6	2	1	3
P	4	4	4	3	7	5	2	3	5	5	9	4	5	1	2	4	1	3	2	2	10	2	1	0	11	3	5	4	1	8
Q	1	1	3	2	3	0	2	1	3	2	1	5	3	3	2	2	3	1	2	1	0	5	2	3	3	4	4	0	2	2
R	3	2	2	3	2	3	3	5	4	6	7	3	3	4	0	1	1	1	3	2	4	4	3	3	2	2	2	2	5	3
S	9	3	7	2	6	3	5	7	16	9	5	8	6	4	5	12	4	5	3	10	5	7	4	5	8	4	7	2	4	2
T	7	5	3	3	2	6	5	5	8	8	7	7	5	4	5	3	1	8	9	2	1	4	4	7	6	4	7	3	2	6
V	9	3	7	6	3	2	7	11	4	10	3	4	3	1	3	8	3	3	5	3	6	3	5	3	6	3	2	5	3	3
W	1	0	3	1	0	2	0	0	1	0	1	0	0	0	1	0	3	0	0	0	1	1	1	2	1	0	0	0	0	0
Y	1	2	4	5	2	5	2	2	1	1	0	2	1	0	1	0	2	1	7	3	2	3	2	5	0	1	7	5	2	1
total	68	66	65	67	62	73	72	77	84	88	69	82	65	69	68	65	50	70	69	63	74	69	69	91	80	53	78	61	57	56

	C20	C19	C18	C17	C16	C15	C14	C13	C12	C11	C10	C9	C8	C7	C6	C5	C4	C3	C2	C1	C0	C-index	SD	*P-value*	mark	
A	5	3	2	3	3	2	3	10	11	5	6	5	1	3	3	3	1	5	1	5	5	0.804	0.484	–	–	A
C	1	1	0	3	3	1	3	1	1	2	2	0	0	2	2	0	1	1	0	3	0	–	–	–	–	C
D	2	4	1	2	9	5	5	6	1	6	3	5	2	2	3	3	7	4	2	6	3	1.247	0.609	–	–	D
E	4	4	8	4	7	9	5	5	7	3	3	3	5	7	4	1	4	3	3	2	3	1.273	0.682	–	–	E
F	3	2	1	3	2	1	4	6	4	1	4	5	3	4	2	2	1	4	3	1	2	1.550	0.816	–	–	F
G	3	3	4	2	3	5	8	4	1	2	6	4	3	2	3	3	2	3	2	5	4	0.905	0.468	–	–	G
H	3	2	3	0	1	2	3	0	0	2	0	1	1	3	3	1	1	3	0	0	3	0.513	0.394	6.51E-03	n.s.	H
I	2	5	6	3	4	2	6	4	4	6	11	4	10	10	2	7	4	4	2	3	3	1.787	1.116	–	–	I
K	3	4	2	9	5	6	11	9	4	8	3	3	2	5	2	8	4	5	3	7	6	0.713	0.391	5.09E-01	n.s.	K
L	10	5	3	4	5	2	4	6	2	3	10	4	7	6	9	4	6	3	5	6	9	0.649	0.246	2.18E-01	n.s.	L
M	1	2	1	0	0	1	4	0	3	1	0	0	1	0	2	3	3	0	1	4	3	0.500	0.388	1.76E-03	n.s.	M
N	5	9	2	3	3	8	3	3	1	0	3	5	2	5	3	5	8	3	5	5	6	0.653	0.333	2.14E-01	n.s.	N
P	3	4	1	1	4	7	2	1	2	1	4	2	5	5	2	5	6	7	4	6	4	0.960	0.615	–	–	P
Q	3	3	3	7	3	3	2	7	2	1	5	1	1	5	4	4	10	3	5	4	5	0.568	0.382	1.02E-03	n.s.	Q
R	8	11	1	4	4	2	1	3	9	7	5	6	4	3	1	4	8	2	6	5	6	0.607	0.381	4.74E-03	n.s.	R
S	7	9	6	9	8	5	3	7	3	6	6	4	3	6	4	9	10	5	7	5	12	0.498	0.229	1.98E-07	*	S
T	6	5	5	9	5	5	7	3	6	3	8	5	4	3	3	9	4	8	4	4	14	0.361	0.152	5.55E-08	*	T
V	7	6	2	4	4	5	8	9	8	8	5	6	5	5	11	6	3	5	3	4	3	1.673	0.812	–	–	V
W	0	1	1	0	0	1	0	0	0	1	2	1	0	2	0	0	1	1	0	0	2	0.300	0.404	5.60E-08	*	W
Y	4	4	1	3	1	4	0	1	1	4	1	5	2	2	2	1	1	3	2	4	5	0.464	0.353	1.10E-04	n.s.	Y
total	80	87	53	73	74	76	82	85	70	70	87	69	61	80	65	78	85	72	58	79	98	0.727	0.100			total

position -3

	C50	C49	C48	C47	C46	C45	C44	C43	C42	C41	C40	C39	C38	C37	C36	C35	C34	C33	C32	C31	C30	C29	C28	C27	C26	C25	C24	C23	C22	C21
A	5	10	4	6	1	2	5	7	3	7	8	6	5	1	4	3	0	7	4	14	3	2	4	5	4	4	2	6	4	2
C	2	0	0	0	1	0	1	2	1	1	3	4	2	0	1	0	0	2	2	0	1	1	3	0	0	1	0	1	1	2
D	5	7	1	5	5	6	5	1	3	1	1	3	1	1	5	4	4	5	0	3	6	2	4	5	5	6	1	6	1	2
E	3	1	4	1	2	9	8	5	1	1	2	5	5	7	5	3	4	4	3	6	7	9	4	6	9	3	6	4	7	3
F	2	0	3	4	2	1	3	3	3	0	6	6	1	5	1	5	3	3	3	2	2	3	1	1	2	4	5	4	2	1
G	8	5	2	2	6	5	4	3	5	6	3	2	3	5	4	5	2	2	2	1	1	5	5	7	1	3	4	2	2	3
H	0	0	1	0	1	3	2	3	0	1	2	0	0	0	1	1	1	0	3	1	5	1	1	1	1	1	1	0	1	0
I	1	3	5	0	6	2	6	3	2	5	3	7	4	5	3	6	2	2	1	1	5	5	2	1	1	1	5	2	3	3
K	3	2	7	2	4	5	5	4	9	7	4	6	3	2	5	2	4	6	7	3	4	2	4	4	6	4	9	2	3	3
L	6	8	4	11	9	7	7	4	10	9	4	9	4	5	5	4	3	5	6	4	5	6	5	2	7	3	8	2	8	5
M	4	3	0	2	1	2	3	4	3	6	0	3	2	6	3	2	1	1	1	1	3	2	1	1	2	0	2	1	1	1
N	6	3	4	3	2	1	3	3	3	6	0	3	7	1	4	1	2	3	8	2	5	3	2	5	3	2	1	3	3	5
P	2	2	2	5	1	4	2	5	7	2	3	3	3	10	4	8	7	0	5	3	4	6	3	2	0	5	1	7	3	7
Q	1	2	2	2	4	1	2	3	3	9	4	1	3	1	2	0	1	1	2	3	3	2	5	22	9	3	2	1	2	2
R	2	1	4	6	0	3	1	5	4	3	3	4	3	3	1	3	1	3	3	3	6	7	4	2	3	3	5	1	1	2
S	7	8	10	8	4	9	9	12	9	3	9	5	5	6	10	6	7	13	7	8	5	4	6	8	7	3	8	10	7	8
T	5	3	5	6	5	7	3	4	8	10	9	6	4	3	6	7	3	6	7	4	3	4	4	10	9	3	6	4	3	4
V	3	3	5	3	6	3	0	4	7	10	0	7	6	1	3	4	4	6	2	1	4	5	7	6	7	3	6	4	3	3
W	0	0	0	0	0	2	1	2	1	1	3	0	0	2	0	1	0	0	1	2	1	0	0	1	0	0	2	0	0	0
Y	3	5	2	1	2	1	2	0	2	0	2	2	4	5	1	0	1	1	2	1	1	0	4	2	4	1	4	1	2	0
total	68	66	65	67	62	73	72	77	84	88	69	82	65	69	68	65	50	70	69	63	74	69	69	91	80	53	78	61	57	56

	C20	C19	C18	C17	C16	C15	C14	C13	C12	C11	C10	C9	C8	C7	C6	C5	C4	C3	C2	C1	C0	C-index	SD	*P-value*	mark	
A	4	5	5	8	5	5	7	5	10	6	7	6	5	8	1	3	7	3	6	4	5	0.992	0.516	–	–	A
C	1	0	0	2	1	0	2	1	2	1	2	1	1	1	1	4	0	1	1	1	0	–	–	–	–	C
D	6	3	2	1	2	3	3	3	5	1	1	3	5	3	4	2	2	3	1	3	4	0.800	0.460	–	–	D
E	5	5	2	6	10	1	6	4	4	2	11	3	1	5	5	3	4	3	2	6	17	0.265	0.147	5.55E-08	*	E
F	2	1	4	5	2	4	2	3	2	6	4	2	3	7	3	1	4	1	5	1	2	1.430	0.839	–	–	F
G	3	6	5	3	2	11	4	5	2	3	7	1	4	4	4	7	3	4	4	0	5	0.760	0.416	–	–	G
H	0	3	0	0	0	1	3	1	1	0	1	1	3	1	1	2	3	1	0	3	2	0.570	0.580	1.08E-04	n.s.	H
I	3	2	4	3	6	3	3	1	5	4	6	5	3	7	3	4	7	6	4	11	5	0.740	0.426	–	–	I
K	8	6	2	5	9	4	5	3	3	6	1	6	4	4	8	15	8	3	1	4	4	1.180	0.647	–	–	K
L	5	6	3	6	2	2	4	6	4	4	5	12	4	7	7	6	1	4	1	5	5	1.076	0.491	–	–	L
M	4	2	4	2	4	1	3	2	2	1	1	0	0	2	2	1	1	1	1	2	4	0.490	0.350	3.93E-05	*	M
N	4	5	1	5	4	6	3	6	3	2	5	3	4	2	1	5	6	3	2	1	3	1.120	0.598	–	–	N
P	2	4	4	5	5	4	4	9	3	5	6	4	3	5	5	3	3	5	6	4	3	1.367	0.710	–	–	P
Q	8	11	0	0	3	7	1	6	1	4	6	1	3	2	0	3	4	7	2	2	2	1.690	1.838	–	–	Q
R	3	3	4	6	2	1	7	2	3	5	1	4	1	2	2	1	6	1	3	5	3	1.013	0.583	–	–	R
S	9	9	5	5	5	10	11	5	4	7	9	7	5	5	6	6	7	14	8	6	10	0.728	0.245	–	–	S
T	3	6	4	6	8	10	7	13	8	6	7	0	4	4	6	5	6	6	5	7	9	0.627	0.265	3.62E-02	n.s.	T
V	8	5	2	3	1	1	5	8	4	7	3	7	5	7	4	5	6	4	4	11	10	0.452	0.240	5.99E-08	*	V
W	0	2	1	1	2	1	1	1	1	0	3	2	0	2	1	0	2	0	1	3	0	–	–	–	–	W
Y	2	3	1	1	1	1	1	1	3	0	1	1	3	2	1	2	5	2	1	0	5	0.352	0.273	5.75E-08	*	Y
total	80	87	53	73	74	76	82	85	70	70	87	69	61	80	65	78	85	72	58	79	98	0.727	0.100			total

position -1

	C50	C49	C48	C47	C46	C45	C44	C43	C42	C41	C40	C39	C38	C37	C36	C35	C34	C33	C32	C31	C30	C29	C28	C27	C26	C25	C24	C23	C22	C21
A	3	6	5	3	6	6	2	5	4	3	4	6	3	8	2	5	2	3	5	5	5	11	5	24	7	5	3	8	0	3
C	0	4	2	8	0	1	2	1	8	2	2	1	0	4	2	0	3	2	2	2	6	5	0	3	2	1	1	1	1	1
D	4	4	2	2	5	3	5	5	6	6	4	6	0	7	7	3	5	3	1	1	1	1	4	6	5	3	1	1	3	8
E	5	1	3	2	4	0	5	5	9	8	3	3	2	2	3	3	4	3	3	4	9	4	5	3	6	3	14	7	3	3
F	4	6	0	2	2	5	2	9	3	3	1	7	3	3	5	4	2	0	2	2	5	2	4	2	1	2	2	6	2	1
G	3	1	3	3	3	5	0	4	3	6	1	4	4	1	4	3	3	6	4	4	9	1	3	2	2	4	1	3	1	0
H	0	0	0	2	2	4	1	2	0	2	0	1	3	1	2	0	1	0	0	3	1	2	2	6	0	0	0	3	0	1
I	2	8	9	11	3	4	4	4	6	8	11	5	9	9	5	7	4	9	5	5	4	3	5	0	10	5	5	3	10	6
K	2	2	2	2	0	5	4	4	2	2	0	2	3	5	2	2	1	4	6	7	4	6	3	3	6	3	4	1	2	0
L	5	5	3	3	7	4	9	4	7	6	5	6	6	7	3	13	7	3	9	5	4	3	2	6	5	6	7	7	6	0
M	0	4	6	4	2	2	1	1	0	3	2	2	3	0	1	3	1	0	0	1	0	0	3	2	1	0	2	1	2	2
N	4	2	2	1	1	1	3	6	4	4	0	4	1	4	0	2	1	2	5	3	4	5	3	1	4	4	4	4	2	2
P	4	5	2	4	4	6	3	2	5	5	6	6	4	2	3	6	2	3	0	3	3	6	1	2	4	0	3	1	4	9
Q	4	0	2	1	2	3	3	0	1	2	3	3	1	1	3	2	1	5	1	1	0	1	3	1	2	0	5	2	2	3
R	5	4	2	1	4	2	4	6	1	2	4	5	0	2	2	1	1	1	2	3	1	1	0	2	1	1	4	1	4	2
S	9	1	4	6	6	7	6	8	7	5	5	9	5	4	6	1	2	10	6	6	7	5	5	7	7	4	3	3	1	4
T	5	5	8	8	5	5	5	4	9	13	10	3	7	5	6	6	6	9	3	4	4	6	6	6	12	6	5	3	6	5
V	7	6	6	2	5	10	10	7	7	6	4	8	9	2	9	2	3	5	12	4	6	6	7	12	3	6	11	4	4	6
W	1	2	0	1	0	0	1	0	2	2	1	0	0	0	0	0	0	0	0	0	1	0	1	2	0	0	0	0	2	0
Y	1	0	4	1	1	0	2	0	0	0	3	1	2	2	3	2	1	2	3	0	0	1	7	1	2	0	3	2	2	0
total	68	66	65	67	62	73	72	77	84	88	69	82	65	69	68	65	50	70	69	63	74	69	69	91	80	53	78	61	57	56

	C20	C19	C18	C17	C16	C15	C14	C13	C12	C11	C10	C9	C8	C7	C6	C5	C4	C3	C2	C1	C0	C-index	SD	*P-value*	mark	
A	7	11	1	7	7	6	2	9	4	4	9	4	3	8	1	8	6	5	4	5	7	0.766	0.519	–	–	A
C	3	2	1	0	0	0	1	1	2	1	6	0	0	1	0	3	2	0	3	3	2	0.960	0.979	–	–	C
D	3	3	1	4	3	3	6	2	4	3	4	3	3	2	2	3	2	4	1	4	7	0.491	0.265	1.06E-05	*	D
E	6	2	2	3	4	5	7	4	11	1	1	5	4	4	2	2	2	5	2	2	3	1.353	0.882	–	–	E
F	5	5	4	3	1	4	2	5	6	4	3	2	2	5	3	6	6	7	2	5	7	0.491	0.282	9.89E-05	*	F
G	2	7	6	4	4	7	1	5	3	2	2	2	2	3	2	0	3	4	2	2	0	–	–	–	–	G
H	2	1	2	1	3	2	0	4	1	1	0	0	0	1	4	5	2	2	2	2	4	0.370	0.362	7.84E-08	*	H
I	5	9	10	8	8	10	8	3	1	1	7	5	7	7	6	2	9	7	1	4	4	1.485	0.719	–	–	I
K	6	4	6	5	3	4	4	3	4	7	5	0	4	2	5	5	2	5	0	3	7	0.474	0.270	1.25E-05	*	K
L	3	6	3	4	7	5	12	4	4	7	7	6	5	6	4	5	12	3	5	5	7	0.789	0.353	–	–	L
M	1	2	2	0	2	5	0	1	0	1	3	0	1	3	2	1	3	1	0	2	2	0.790	0.701	–	–	M
N	2	4	1	3	2	1	2	4	0	2	3	4	5	2	6	3	4	3	3	6	5	0.572	0.316	9.42E-02	n.s.	N
P	1	5	4	3	4	2	3	7	1	4	7	2	4	1	6	7	3	7	10	5	4	0.970	0.552	–	–	P
Q	5	3	2	0	1	1	0	3	1	2	2	3	2	3	1	1	5	1	2	7	4	0.515	0.383	4.43E-04	n.s.	Q
R	8	6	0	5	5	2	6	1	2	6	4	4	1	4	3	3	2	1	0	0	3	0.880	0.652	–	–	R
S	3	7	2	6	3	8	7	12	7	6	6	8	8	13	7	8	10	6	3	6	11	0.536	0.238	1.19E-05	*	S
T	8	2	4	14	6	4	10	9	9	5	6	9	6	5	2	6	2	4	7	9	10	0.624	0.269	1.17E-02	n.s.	T
V	8	6	1	1	7	6	6	7	6	11	8	8	2	8	5	8	7	5	10	7	8	0.790	0.342	–	–	V
W	0	1	1	0	0	0	1	0	1	0	4	2	1	1	1	0	2	1	0	1	1	0.660	0.872	1.00E+00	n.s.	W
Y	2	1	0	2	4	1	4	1	3	2	0	2	1	1	3	2	1	1	1	1	2	0.790	0.693	–	–	Y
total	80	87	53	73	74	76	82	85	70	70	87	69	61	80	65	78	85	72	58	79	98	0.727	0.100			total

Additional file 5. Continued.

NEMATODE x-x-V-x-I

position -4

	C50	C49	C48	C47	C46	C45	C44	C43	C42	C41	C40	C39	C38	C37	C36	C35	C34	C33	C32	C31	C30	C29	C28	C27	C26	C25	C24	C23	C22	C21	C20	C19	C18	C17	C16	C15	C14	C13	C12	C11	C10	C9	C8	C7	C6	C5	C4	C3	C2	C1	C0	C-index	SD	*P-value*	mark	
A	4	5	9	7	5	2	2	7	4	2	5	14	13	6	9	2	9	2	4	3	8	4	8	5	6	7	5	20	4	6	3	4	0	3	2	2	2	6	8	2	2	6	2	6	5	3	1	3	1	3	4	1.255	0.910	−	−	A
C	2	2	1	0	2	0	0	2	3	1	3	1	0	6	2	0	3	2	4	1	1	1	2	2	1	1	0	3	2	1	1	1	1	1	3	0	1	1	1	2	2	1	3	5	1	1	2	2	1	1	0	−	−	−	−	C
D	8	2	4	2	6	5	5	6	2	4	7	5	8	3	4	5	7	3	4	8	8	5	3	4	9	4	4	4	1	6	6	5	4	1	6	8	3	4	2	5	2	0	10	2	5	5	0	3	4	1	8	0.555	0.291	8.21E-08	✻	D
E	2	4	7	6	4	3	3	0	5	4	8	4	7	2	4	1	5	8	5	4	1	7	6	5	2	4	7	2	3	7	9	5	2	6	1	1	3	1	4	0	3	5	6	6	4	6	3	4	8	3	2	2.100	1.129	−	−	E
F	5	4	5	4	5	1	6	2	5	3	3	9	6	2	6	3	6	3	5	2	4	5	2	5	3	2	3	1	4	4	3	4	4	5	7	1	3	6	4	8	2	4	3	9	4	3	5	1	3	2	4	0.995	0.478	−	−	F
G	4	3	3	3	3	1	13	3	5	3	3	4	6	4	4	5	7	5	11	4	6	4	7	3	7	5	5	6	1	2	6	3	0	1	4	3	5	2	3	3	5	2	5	5	12	5	0	2	5	1	1	4.240	2.638	−	−	G
H	0	3	2	0	1	2	3	1	1	3	1	2	2	1	1	3	0	0	0	1	0	1	2	0	1	3	2	0	1	2	3	1	2	0	1	0	2	1	1	2	1	1	2	0	2	3	5	1	4	0	2	0.710	0.590	3.54E-01	n.s.	H
I	6	11	12	1	6	7	7	7	4	6	1	5	3	4	7	5	4	6	7	3	1	8	3	6	8	4	4	6	3	7	4	3	4	7	6	6	2	4	4	11	7	4	6	5	2	3	1	3	3	5	5	1.008	0.498	−	−	I
K	6	2	4	8	5	8	3	2	9	7	8	4	6	9	2	4	3	7	3	2	4	3	8	2	3	6	3	7	4	6	3	5	5	3	23	4	8	3	3	2	7	6	8	2	11	5	2	6	6	4	18	0.293	0.192	5.55E-08	✻	K
L	7	8	8	7	8	5	6	8	7	5	8	7	6	9	7	2	6	5	8	7	8	4	4	5	8	7	7	8	5	10	7	5	6	7	7	3	9	5	7	5	4	6	3	5	7	3	8	4	5	4	6	1.033	0.299	−	−	L
M	1	1	0	2	1	2	0	4	2	0	3	1	1	1	0	1	0	1	2	3	0	0	1	0	4	2	1	1	1	3	0	0	0	0	0	1	0	2	0	0	0	1	1	3	1	1	1	1	0	3	5	0.216	0.224	5.55E-08	✻	M
N	7	4	7	6	5	3	1	10	3	7	1	2	3	1	3	1	3	4	3	3	3	1	5	3	2	5	2	2	1	6	0	4	4	4	5	4	5	5	4	7	9	1	4	2	3	6	1	6	6	6	4	0.965	0.558	−	−	N
P	10	4	3	5	4	4	2	6	3	2	5	4	5	2	6	11	9	2	5	4	3	5	1	4	4	1	5	2	5	5	5	1	1	2	3	3	3	5	5	9	1	3	2	2	5	2	3	3	4	7	2	2.000	1.138	−	−	P
Q	3	2	2	4	3	6	3	1	2	4	3	2	3	3	5	2	3	1	2	2	1	2	2	1	4	0	1	2	2	1	1	5	9	2	1	5	4	2	2	7	3	2	4	8	7	1	3	4	1	6	2	1.490	0.992	−	−	Q
R	2	6	2	6	3	1	3	3	5	2	4	1	9	2	4	8	3	4	4	1	6	3	3	5	8	5	3	3	4	1	1	3	3	7	3	4	5	7	7	5	5	5	2	0	5	5	3	5	1	0	2	1.900	1.064	−	−	R
S	8	4	8	2	4	2	6	7	4	5	3	6	2	5	4	5	2	6	2	4	8	7	3	6	3	3	3	4	5	2	5	5	8	3	2	5	2	7	8	3	10	7	4	5	12	7	4	8	9	13	7	0.743	0.375	2.57E-02	n.s.	S
T	1	4	4	3	3	3	4	7	4	7	4	5	11	4	1	4	4	2	5	10	3	2	8	5	0	8	4	5	4	2	4	5	4	4	4	1	4	4	2	7	2	3	5	4	1	0	2	8	6	7	0	−	−	−	−	T
V	11	7	5	4	1	7	4	6	4	4	5	3	6	4	8	4	4	6	3	4	4	6	7	7	3	5	5	7	5	2	3	3	4	2	3	3	4	5	3	3	5	1	2	1	3	5	6	2	2	9	5	0.880	0.416	8.75E-01	n.s.	V
W	0	1	2	2	2	1	0	0	0	0	1	1	0	0	0	1	0	3	0	2	0	0	0	0	0	0	2	1	0	0	2	1	1	2	0	1	1	0	1	0	1	0	0	0	1	0	0	3	0	0	0	−	−	−	−	W
Y	3	1	2	0	0	2	4	3	2	3	1	0	3	1	1	1	6	1	1	0	2	1	2	3	1	4	1	3	2	1	0	4	0	2	1	2	4	1	1	0	1	2	2	6	0	2	4	0	3	3	5	0.372	0.300	5.55E-08	✻	Y
total	90	78	90	72	71	65	75	85	74	72	77	80	100	69	78	68	84	71	78	68	71	69	77	71	77	76	67	87	57	74	66	67	62	62	82	57	70	71	70	81	72	60	74	76	91	66	54	69	72	78	82	0.895	0.112			total

position -3

	C50	C49	C48	C47	C46	C45	C44	C43	C42	C41	C40	C39	C38	C37	C36	C35	C34	C33	C32	C31	C30	C29	C28	C27	C26	C25	C24	C23	C22	C21	C20	C19	C18	C17	C16	C15	C14	C13	C12	C11	C10	C9	C8	C7	C6	C5	C4	C3	C2	C1	C0	C-index	SD	*P-value*	mark	
A	6	10	4	1	5	2	6	6	5	5	5	5	5	6	5	4	11	3	8	4	1	5	5	9	5	4	1	3	4	8	6	7	2	6	2	4	4	4	7	6	1	5	3	6	5	6	1	7	4	4	4	1.205	0.557	−	−	A
C	2	1	0	2	3	0	1	1	3	0	1	2	0	1	1	0	0	1	0	1	1	1	1	0	2	2	7	1	0	1	0	1	1	0	2	0	1	1	1	0	0	0	1	3	0	0	1	0	2	1	2	0.510	0.610	3.04E-06	✻	C
D	3	2	4	10	3	2	10	4	1	2	5	2	5	2	7	2	1	1	4	4	1	4	2	1	4	4	4	5	1	5	7	5	2	5	1	3	1	5	2	4	6	5	5	4	1	4	1	3	1	2	9	0.382	0.241	5.55E-08	✻	D
E	5	1	2	9	5	3	4	4	1	4	1	4	5	2	2	7	2	3	7	3	0	0	7	3	4	4	3	4	2	3	3	3	4	5	2	4	3	2	3	2	2	2	2	5	1	4	4	3	6	4	3	1.120	0.609	−	−	E
F	3	6	4	2	2	7	4	1	4	4	8	1	1	3	3	3	4	7	7	5	6	5	6	4	5	6	2	2	2	3	4	4	1	4	4	6	4	4	4	7	4	1	2	10	6	3	4	1	5	3	11	0.365	0.184	5.55E-08	✻	F
G	2	4	8	3	4	1	2	9	4	5	3	6	6	6	3	5	5	10	7	3	3	3	7	5	6	2	5	19	5	1	2	7	2	2	7	1	2	4	3	5	0	1	4	2	5	3	5	6	5	3	3	1.473	1.013	−	−	G
H	3	1	2	2	3	3	2	1	3	1	0	9	0	3	1	2	0	0	0	3	2	1	1	1	2	1	1	4	1	2	1	1	1	0	1	0	3	2	4	3	4	2	0	2	0	3	0	2	0	2	1	1.720	1.591	−	−	H
I	13	5	5	4	3	5	11	7	4	6	9	3	9	6	9	5	9	6	8	9	4	4	5	2	4	5	8	5	2	4	5	8	2	1	4	3	7	6	4	8	6	1	6	9	6	6	3	6	4	6	3	1.867	0.846	−	−	I
K	3	6	7	2	6	2	4	7	4	3	5	3	6	6	3	2	1	3	1	0	3	6	6	4	6	8	4	3	3	5	5	3	2	2	2	4	2	5	1	5	5	5	7	1	4	2	1	7	9	8	3	1.347	0.722	−	−	K
L	17	15	10	4	6	10	6	3	12	5	10	7	10	4	11	4	8	7	5	9	7	7	6	5	6	3	2	9	6	7	5	4	8	5	6	8	11	5	3	7	4	7	7	8	8	3	1	3	6	6	4	1.680	0.786	−	−	L
M	4	3	0	3	1	0	3	2	2	1	5	0	3	0	3	1	2	0	2	2	3	1	3	0	2	2	0	1	3	2	2	2	2	0	1	1	0	0	1	0	0	3	4	1	0	1	2	2	0	2	0	−	−	−	−	M
N	3	3	7	4	1	1	3	7	1	7	2	5	2	2	7	6	6	2	3	2	3	7	3	5	4	2	6	6	3	7	5	3	4	1	5	2	5	5	6	8	2	6	5	4	3	5	4	3	3	5	5	0.816	0.385	8.84E-01	n.s.	N
P	4	4	4	6	5	8	3	4	4	5	3	7	3	4	7	3	7	2	7	3	5	5	4	2	0	4	4	1	2	4	5	2	1	4	1	1	1	4	5	5	4	3	5	1	6	5	3	2	5	2	6	0.630	0.309	1.50E-06	✻	P
Q	2	2	2	1	0	1	2	2	3	1	0	3	1	4	2	0	0	4	1	3	3	0	5	3	6	4	1	2	1	1	2	1	4	1	3	1	3	2	3	1	3	4	1	0	2	2	1	2	3	1	1	2.000	1.385	−	−	Q
R	2	2	1	0	3	2	1	7	1	4	3	5	5	10	4	2	6	1	0	3	5	5	4	5	7	3	3	8	4	3	3	0	3	3	22	3	4	2	7	2	5	3	4	2	2	6	3	3	0	4	1	3.800	3.381	−	−	R
S	4	3	11	9	1	12	5	3	8	7	5	3	11	5	3	5	9	4	2	3	7	5	5	9	4	8	3	4	7	5	2	6	7	10	4	10	6	7	6	2	9	3	4	8	11	2	8	10	9	12	13	0.471	0.231	5.55E-08	✻	S
T	9	2	8	6	8	0	5	9	4	2	5	2	6	0	3	8	3	6	4	4	5	2	3	7	4	6	3	6	5	4	2	2	8	2	4	2	6	10	5	8	8	6	6	5	13	4	3	3	2	6	6	0.813	0.445	6.63E-01	n.s.	T
V	4	6	6	4	7	5	1	4	5	8	3	9	16	2	3	4	7	3	6	4	8	7	1	4	3	5	8	4	4	5	3	7	4	5	9	2	6	1	3	6	6	2	7	2	10	6	6	4	6	7	4	1.290	0.673	−	−	V
W	0	1	1	0	1	1	0	0	1	0	1	1	5	0	0	0	1	4	1	0	1	0	1	0	2	2	0	0	2	0	1	1	0	3	0	1	0	1	1	0	0	0	0	0	4	0	1	0	1	0	0	−	−	−	−	W
Y	1	1	4	0	4	0	2	4	4	2	3	3	1	3	1	5	2	4	5	3	3	1	2	2	1	1	2	0	0	4	3	0	4	3	2	1	1	1	1	2	3	1	1	3	4	1	2	2	1	0	3	0.693	0.471	1.41E-01	n.s.	Y
total	90	78	90	72	71	65	75	85	74	72	77	80	100	69	78	68	84	71	78	68	71	69	77	71	77	76	67	87	57	74	66	67	62	62	82	57	70	71	70	81	72	60	74	76	91	66	54	69	72	78	82	0.895	0.112			total

position -1

	C50	C49	C48	C47	C46	C45	C44	C43	C42	C41	C40	C39	C38	C37	C36	C35	C34	C33	C32	C31	C30	C29	C28	C27	C26	C25	C24	C23	C22	C21	C20	C19	C18	C17	C16	C15	C14	C13	C12	C11	C10	C9	C8	C7	C6	C5	C4	C3	C2	C1	C0	C-index	SD	*P-value*	mark	
A	6	2	3	4	2	4	4	4	7	6	1	6	3	8	6	5	9	8	2	0	10	10	4	9	3	3	2	5	1	6	4	3	2	3	2	2	3	4	5	5	2	3	3	5	6	3	1	6	4	7	2	2.160	1.206	−	−	A
C	2	3	4	0	4	2	3	0	3	2	1	1	2	1	0	1	4	2	4	1	2	1	2	0	3	3	3	3	2	2	3	2	1	2	1	0	1	2	0	1	2	2	1	1	0	2	1	2	2	0	1	1.740	1.157	−	−	C
D	8	2	7	4	5	2	1	5	1	3	3	2	5	3	2	4	3	5	6	2	1	2	2	3	3	7	3	3	4	5	2	4	0	4	2	4	1	0	3	2	2	5	2	5	8	3	2	0	3	3	4	0.805	0.477	1.79E-01	n.s.	D
E	4	2	6	4	6	5	1	3	4	4	6	4	3	5	5	3	4	2	4	1	3	3	3	5	5	6	4	4	7	7	4	1	3	2	8	2	6	6	7	11	8	4	7	3	8	5	4	7	4	5	5	0.912	0.412	−	−	E
F	2	4	1	3	2	8	3	8	3	2	11	3	3	5	4	3	4	5	4	6	4	5	5	5	4	8	3	7	3	6	3	5	2	4	9	1	2	2	7	4	3	0	3	3	2	2	0	3	7	2	3	1.320	0.771	−	−	F
G	7	3	1	6	4	7	2	2	4	5	4	4	4	3	1	5	3	4	5	3	3	4	3	3	1	3	3	3	4	4	2	2	4	4	3	5	7	5	4	2	1	2	4	3	3	2	1	4	3	2	2	1.710	0.750	−	−	G
H	5	3	1	1	2	0	2	3	1	1	2	1	1	1	4	0	1	1	1	2	0	4	3	4	1	0	1	0	1	2	3	0	2	2	3	0	2	3	2	2	2	1	0	2	1	2	2	1	3	4	4	0.430	0.312	5.56E-08	✻	H
I	6	7	8	7	2	5	7	10	5	4	3	4	10	5	8	3	5	2	11	4	7	6	6	5	1	2	5	5	5	4	5	3	2	5	3	4	7	6	2	8	2	5	4	10	5	5	5	2	7	5	7	0.734	0.333	4.13E-03	n.s.	I
K	8	3	9	4	3	4	5	5	6	3	4	11	4	4	5	3	6	0	4	8	4	10	5	4	8	5	2	7	3	4	3	5	12	6	4	7	4	4	5	6	1	7	6	7	3	4	3	4	3	8	16	0.316	0.150	5.55E-08	✻	K
L	4	12	10	4	6	5	7	9	10	9	1	10	11	3	7	9	6	7	5	11	3	6	3	9	7	7	6	2	3	9	6	10	4	3	4	3	5	7	7	2	3	3	5	6	7	5	8	4	5	5	5	1.212	0.544	−	−	L
M	4	4	5	3	0	1	0	2	2	4	5	0	5	2	0	4	3	2	0	4	2	1	3	3	2	3	0	1	2	1	0	2	3	1	1	3	0	0	0	6	1	1	1	3	3	1	1	1	0	3	2	0.990	0.811	−	−	M
N	2	3	1	2	2	3	5	3	2	2	6	3	4	2	0	2	5	1	6	2	5	0	5	1	4	4	7	2	3	5	5	1	1	5	1	4	1	1	2	2	8	6	3	4	4	3	3	3	4	1	2	1.540	0.925	−	−	N
P	3	3	2	5	3	4	7	3	4	1	3	4	4	1	2	3	2	1	6	5	2	3	7	2	5	3	1	4	5	2	6	1	3	3	5	1	4	2	4	4	4	3	7	3	4	10	5	6	6	2	1	3.660	1.902	−	−	P
Q	2	2	2	2	3	1	0	3	2	7	4	4	5	5	5	3	4	1	0	3	5	0	4	1	10	3	5	5	0	3	2	9	0	0	1	4	2	1	1	4	3	3	4	3	4	3	2	4	4	3	3	1.007	0.708	−	−	Q
R	3	4	5	3	3	3	7	1	1	4	4	5	3	6	4	1	2	10	0	2	1	0	6	4	2	1	2	4	4	3	2	3	4	2	1	3	4	5	1	4	6	1	4	1	6	2	1	3	3	5	5	0.636	0.395	2.02E-05	✻	R
S	10	8	7	3	5	2	5	4	5	3	5	7	15	5	2	5	4	5	4	3	3	5	3	2	3	8	6	8	3	2	7	7	6	4	6	6	12	9	7	7	5	4	9	5	7	2	9	4	3	10	9	0.620	0.306	7.68E-07	✻	S
T	7	5	3	3	6	5	5	5	4	5	5	3	7	6	12	3	1	3	2	4	8	2	5	6	5	2	5	19	3	5	0	3	4	5	23	3	1	6	6	2	8	4	4	6	9	6	1	6	5	4	2	2.600	1.974	−	−	T
V	5	3	11	9	9	2	9	11	5	5	4	4	7	2	7	2	8	7	7	5	7	3	7	3	7	5	3	3	2	1	5	2	9	4	2	4	7	5	3	7	9	3	4	6	8	2	2	1	2	4	6	0.840	0.448	7.65E-01	n.s.	V
W	0	0	1	1	0	0	0	3	1	1	1	1	1	2	2	0	9	3	4	1	0	1	1	0	0	2	0	0	0	1	1	2	0	0	1	0	0	1	1	0	0	0	2	0	2	1	1	1	1	1	2	0.510	0.739	6.85E-07	✻	W
Y	2	5	3	4	4	2	2	1	4	1	4	3	3	0	2	9	1	2	3	1	1	3	0	2	3	1	6	2	2	2	3	2	0	3	2	1	1	2	3	2	2	3	1	0	1	3	2	7	3	4	1	2.460	1.729	−	−	Y
total	90	78	90	72	71	65	75	85	74	72	77	80	100	69	78	68	84	71	78	68	71	69	77	71	77	76	67	87	57	74	66	67	62	62	82	57	70	71	70	81	72	60	74	76	91	66	54	69	72	78	82	0.895	0.112			total

Additional file 5. Continued.

NEMATODE x-x-V-x-V

position -4

	C50	C49	C48	C47	C46	C45	C44	C43	C42	C41	C40	C39	C38	C37	C36	C35	C34	C33	C32	C31	C30	C29	C28	C27	C26	C25	C24	C23	C22	C21	C20	C19	C18	C17	C16	C15	C14	C13	C12	C11	C10	C9	C8	C7	C6	C5	C4	C3	C2	C1	C0	C-index	SD	*P-value*	mark	
A	5	6	4	7	8	7	6	5	7	4	14	9	9	4	9	6	6	8	7	5	3	8	5	9	3	8	6	5	4	9	5	11	2	2	1	5	9	4	4	1	7	4	11	5	1	12	5	4	1	8	6	0.993	0.488	–	–	A
C	1	3	2	2	5	3	4	1	1	1	2	1	0	1	0	5	1	2	1	0	2	1	1	2	2	2	2	1	5	2	4	2	0	0	1	2	1	1	1	2	1	2	1	3	5	2	2	0	2	1	2	0.890	0.665	9.96E-01	n.s.	C
D	4	5	5	5	7	14	4	2	4	6	2	8	3	4	1	6	3	2	3	5	3	6	7	2	5	3	7	3	5	8	10	5	3	5	1	7	7	6	7	5	3	3	4	5	2	4	7	7	6	4	7	0.694	0.342	4.11E-04	n.s.	D
E	2	7	4	4	3	6	4	5	5	4	12	4	4	6	4	2	2	6	3	9	2	3	7	3	3	5	3	6	1	2	6	7	4	4	4	5	5	8	3	3	6	1	5	9	9	1	5	7	5	3	4	1.155	0.576	–	–	E
F	1	4	5	4	2	4	5	4	0	1	5	6	4	3	3	2	5	3	5	2	3	3	3	0	3	2	5	3	3	1	1	5	1	1	3	6	5	7	4	11	4	8	6	2	3	0	2	11	4	2	5	0.720	0.475	5.19E-03	n.s.	F
G	5	9	9	7	9	4	7	5	4	2	6	4	6	7	3	6	1	9	7	4	6	3	3	7	9	3	4	7	0	2	5	2	4	4	2	2	5	6	5	6	5	5	3	8	2	4	4	4	1	12	4	1.235	0.628	–	–	G
H	0	2	1	1	3	0	0	1	3	2	1	1	1	0	3	2	4	2	2	0	3	2	1	0	0	3	2	0	0	3	3	1	6	3	4	2	0	2	0	2	0	3	3	3	1	0	2	4	3	2	0	–	–	–	–	H
I	8	4	6	7	6	3	5	4	5	7	7	8	6	5	9	5	6	4	4	3	4	1	7	6	3	1	6	1	6	4	5	6	6	8	4	2	4	1	4	4	5	5	5	3	4	5	6	5	6	1	3	1.600	0.646	–	–	I
K	10	5	3	3	8	9	0	2	5	10	7	4	5	7	4	3	4	1	9	7	2	3	3	3	6	8	4	7	1	5	7	3	8	6	8	7	6	4	3	5	7	6	4	5	5	6	6	4	3	7	9	0.573	0.262	5.88E-08	✻	K
L	4	5	5	4	3	12	11	6	5	9	5	4	5	3	6	5	1	7	7	6	5	5	5	12	6	9	2	9	2	9	5	8	7	9	15	8	2	3	4	4	3	7	6	7	12	6	8	3	4	4	10	0.604	0.297	1.82E-07	✻	L
M	2	3	2	0	2	0	0	5	3	2	3	0	0	6	2	1	3	1	2	1	5	1	4	3	2	0	8	1	0	1	1	0	1	1	2	3	0	0	3	0	0	2	2	1	1	0	0	2	2	2	2	0.860	0.857	9.92E-01	n.s.	M
N	3	4	5	5	4	5	2	2	2	3	3	9	5	3	5	4	2	1	2	6	4	8	5	7	9	4	5	7	1	2	3	1	4	5	1	1	5	3	2	3	5	3	8	7	4	8	2	2	4	4	2	2.020	1.078	–	–	N
P	8	9	2	7	4	7	3	4	3	1	3	2	5	3	1	6	3	3	5	6	3	2	3	0	3	4	1	3	6	2	5	2	7	4	4	4	1	9	4	3	1	8	7	3	1	7	1	6	4	5	6	0.660	0.382	8.04E-04	n.s.	P
Q	3	3	2	2	2	3	5	3	1	7	2	3	6	5	0	1	0	5	8	6	2	6	6	2	6	3	2	7	2	5	2	2	6	4	5	1	5	1	1	3	2	3	4	1	1	3	2	4	1	2	4	0.805	0.503	1.49E-01	n.s.	Q
R	7	4	2	4	3	4	4	6	3	5	4	6	3	5	6	5	4	2	6	8	3	4	2	2	3	3	3	6	5	4	3	4	3	5	1	8	4	8	5	4	6	4	4	1	6	4	3	10	4	11	2	2.240	1.031	–	–	R
S	8	9	6	10	5	7	5	6	4	6	8	3	10	4	7	5	6	5	2	6	4	7	3	4	9	4	5	10	1	4	7	3	9	4	7	5	8	13	7	10	10	8	8	11	9	9	9	7	10	13	10	0.680	0.275	1.02E-04	n.s.	S
T	5	6	4	8	5	3	7	6	3	7	10	7	5	7	3	5	6	4	5	5	9	2	7	9	3	5	6	3	5	6	4	5	3	6	4	9	6	8	7	11	6	6	8	2	3	8	5	9	4	6	4	1.430	0.530	–	–	T
V	5	8	7	9	4	5	7	9	3	8	4	7	9	3	8	4	5	2	4	4	5	8	3	4	8	7	7	4	6	6	3	4	9	3	3	4	1	4	5	10	5	7	11	4	4	6	1	2	4	3	5	1.064	0.481	–	–	V
W	0	0	1	1	2	2	1	2	0	0	0	0	1	1	0	2	1	1	2	2	0	0	0	1	0	1	1	0	0	0	1	1	0	0	1	0	0	1	0	2	1	0	1	2	1	0	0	0	0	0	0	–	–	–	–	W
Y	3	0	0	4	2	4	2	2	1	6	2	4	1	0	1	1	2	0	1	4	0	1	2	1	3	3	0	4	1	1	1	4	1	1	2	0	3	5	1	4	1	2	3	1	3	2	2	1	1	2	5	0.384	0.291	5.55E-08	✻	Y
total	84	96	75	94	87	102	82	80	62	91	100	90	88	77	75	76	65	68	85	89	68	74	77	77	86	78	79	87	54	76	81	76	84	75	73	81	77	94	70	93	78	87	104	83	77	87	72	92	69	92	90	0.904	0.115			total

position -3

	C50	C49	C48	C47	C46	C45	C44	C43	C42	C41	C40	C39	C38	C37	C36	C35	C34	C33	C32	C31	C30	C29	C28	C27	C26	C25	C24	C23	C22	C21	C20	C19	C18	C17	C16	C15	C14	C13	C12	C11	C10	C9	C8	C7	C6	C5	C4	C3	C2	C1	C0	C-index	SD	*P-value*	mark	
A	7	9	4	5	8	4	4	4	3	1	1	5	11	3	4	5	6	4	4	5	6	6	5	2	11	3	5	4	1	5	3	5	11	5	5	4	8	8	3	9	7	6	3	9	8	5	5	9	2	3	8	0.658	0.319	4.37E-05	✻	A
C	8	3	1	1	0	1	0	3	1	1	4	1	2	1	2	0	0	0	2	0	1	1	0	2	2	1	1	2	0	3	1	0	0	0	1	1	2	1	0	3	1	2	2	1	0	1	0	1	1	1	0	–	–	–	–	C
D	6	3	6	3	4	7	5	3	3	6	0	2	7	2	3	6	4	2	2	3	3	4	3	2	5	6	5	7	2	7	2	7	3	5	3	3	4	4	6	3	4	2	7	5	2	2	4	7	5	5	3	1.360	0.605	–	–	D
E	4	8	4	5	2	3	4	4	2	7	4	4	4	4	7	1	3	5	4	5	3	10	2	5	5	8	1	4	2	6	7	2	5	0	6	5	2	5	4	1	6	2	2	3	5	12	4	5	2	4	4	1.060	0.577	–	–	E
F	4	6	5	2	4	5	4	2	4	3	5	0	3	3	2	5	5	3	4	8	3	4	5	4	5	4	1	2	2	9	7	2	6	5	2	6	2	6	4	5	1	4	6	5	1	7	3	6	4	6	6	0.680	0.317	1.24E-04	n.s.	F
G	4	6	7	6	6	9	7	6	3	5	9	4	8	5	2	5	2	2	7	4	3	5	3	2	9	2	9	3	3	6	3	4	3	5	4	6	4	6	4	7	7	5	8	8	6	1	2	1	5	8	0	–	–	–	–	G
H	2	3	0	1	3	2	0	1	3	2	4	2	2	3	3	1	2	1	2	3	2	1	1	1	1	3	2	1	0	1	0	3	0	2	1	1	2	2	1	0	1	2	1	3	3	1	1	3	2	1	2	0.830	0.511	1.00E+00	n.s.	H
I	7	10	4	8	9	15	8	2	3	5	5	8	5	6	6	1	1	6	8	6	3	8	9	4	5	3	2	9	8	9	5	6	9	7	4	5	5	14	5	8	7	4	7	4	5	6	6	5	5	6	3	2.040	0.925	–	–	I
K	2	6	2	3	4	4	4	3	3	1	11	14	1	5	1	0	6	6	3	6	3	2	4	1	3	5	5	3	2	2	7	2	6	3	4	4	4	3	0	4	0	9	8	2	4	4	3	3	6	5	6	0.653	0.445	1.73E-05	✻	K
L	9	5	4	9	7	12	4	6	6	10	6	7	4	4	5	5	4	7	13	9	8	4	4	6	3	7	6	6	7	6	6	7	10	8	4	8	7	1	3	2	5	6	8	0	6	6	4	7	6	4	3	2.007	0.834	–	–	L
M	1	1	5	3	3	3	2	3	2	2	4	2	3	2	5	3	2	3	3	2	0	4	3	2	2	2	0	1	1	2	0	5	0	3	2	1	2	3	0	1	1	2	3	0	2	1	1	2	1	2	1	2.060	1.284	–	–	M
N	3	4	1	4	4	7	4	6	1	7	9	11	4	3	3	8	4	5	6	3	3	4	3	7	3	5	2	8	2	4	2	7	4	3	4	4	5	6	4	4	3	3	5	7	6	7	4	5	7	6	8	0.585	0.259	6.64E-08	✻	N
P	7	5	3	5	1	6	7	3	5	4	5	4	6	5	6	4	4	6	4	2	6	3	3	4	8	2	3	8	2	1	9	1	1	2	3	5	2	6	3	5	3	3	9	7	4	1	5	5	2	2	12	0.350	0.177	5.55E-08	✻	P
Q	2	0	2	5	4	3	3	1	4	7	1	1	4	8	3	7	3	3	0	1	4	2	1	5	1	2	4	4	2	1	4	0	2	2	1	1	7	1	3	3	2	4	3	2	2	1	2	5	1	2	1	2.720	1.896	–	–	Q
R	3	4	6	7	5	2	5	4	6	4	5	2	4	4	4	3	5	1	4	7	0	4	4	6	3	3	6	5	5	3	5	6	7	7	5	4	5	3	1	5	4	6	4	1	2	4	5	4	2	4	9	0.462	0.184	5.55E-08	✻	R
S	5	4	5	9	11	6	6	10	3	7	6	5	2	8	5	5	3	5	11	7	3	3	8	8	6	8	11	2	4	4	7	5	4	5	7	6	6	8	14	12	3	11	10	9	5	13	7	9	4	9	8	0.835	0.368	4.61E-01	n.s.	S
T	4	9	4	12	7	5	6	10	7	11	4	4	5	4	1	9	5	4	4	6	8	4	12	3	3	5	4	6	5	3	4	6	6	4	9	7	4	11	4	12	7	8	8	6	7	3	5	8	5	12	4	1.550	0.693	–	–	T
V	3	7	9	3	2	7	7	6	2	5	11	11	7	6	10	5	4	2	2	10	7	3	3	7	8	6	8	4	4	1	5	7	2	6	6	8	4	5	7	5	12	8	7	9	8	10	8	6	8	7	10	0.616	0.268	1.02E-07	✻	V
W	1	1	1	1	1	0	1	2	0	1	4	2	1	0	2	1	0	3	1	0	0	0	1	1	1	1	3	1	0	0	0	0	3	1	0	0	1	0	1	2	2	0	1	0	1	1	0	1	0	0	0	–	–	–	–	W
Y	2	2	2	2	2	1	1	1	1	2	2	1	5	1	1	2	2	0	1	2	2	2	3	5	2	2	1	7	2	3	4	1	2	2	2	2	1	1	3	2	2	0	2	2	0	1	3	0	1	5	2	0.980	0.677	–	–	Y
total	84	96	75	94	87	102	82	80	62	91	100	90	88	77	75	76	65	68	85	89	68	74	77	77	86	78	79	87	54	76	81	76	84	75	73	81	77	94	70	93	78	87	104	83	77	87	72	92	69	92	90	0.904	0.115			total

position -1

	C50	C49	C48	C47	C46	C45	C44	C43	C42	C41	C40	C39	C38	C37	C36	C35	C34	C33	C32	C31	C30	C29	C28	C27	C26	C25	C24	C23	C22	C21	C20	C19	C18	C17	C16	C15	C14	C13	C12	C11	C10	C9	C8	C7	C6	C5	C4	C3	C2	C1	C0	C-index	SD	*P-value*	mark	
A	9	7	6	3	6	5	13	4	7	5	8	15	11	5	2	6	4	3	8	3	2	8	8	0	2	2	3	6	0	4	2	6	8	3	5	4	5	7	4	8	4	7	10	4	2	2	4	5	3	6	3	1.760	1.019	–	–	A
C	2	4	3	5	0	3	3	1	0	2	0	3	2	0	1	1	0	0	2	3	2	1	1	0	8	1	4	0	2	2	2	3	1	5	4	1	1	2	2	2	1	4	1	2	2	1	0	2	0	1	1	1.860	1.616	–	–	C
D	2	4	4	4	6	5	2	2	3	8	7	7	3	4	3	2	3	4	2	6	3	7	5	3	3	5	4	5	1	2	5	2	4	7	4	2	1	6	6	9	4	5	5	1	2	2	3	2	4	3	5	0.784	0.383	7.92E-02	n.s.	D
E	7	5	3	3	2	5	4	4	3	3	3	4	5	3	3	7	1	4	7	4	3	3	5	8	4	6	3	10	3	4	8	2	3	4	6	12	4	4	6	2	2	2	7	4	6	2	6	5	5	4	6	0.743	0.360	1.33E-03	n.s.	E
F	4	3	3	8	6	7	7	4	2	5	8	2	3	5	4	6	3	7	8	7	6	6	6	10	2	5	3	6	3	5	7	6	4	7	7	3	2	1	7	4	5	4	4	6	4	9	5	9	2	2	7	0.720	0.309	2.29E-02	n.s.	F
G	0	8	5	5	2	6	1	5	5	1	5	4	4	1	2	3	6	4	0	4	1	4	5	7	4	3	5	3	0	4	3	5	9	2	3	7	14	5	3	2	5	5	3	1	2	5	2	5	2	1	4	0.955	0.634	–	–	G
H	1	2	2	1	3	2	3	2	1	1	1	1	3	4	5	2	2	3	2	4	1	1	1	0	2	2	0	4	3	2	0	7	3	3	0	2	1	1	1	2	2	4	3	4	3	4	2	3	2	2	2	1.100	0.693	–	–	H
I	5	6	7	9	2	8	2	5	6	7	6	3	5	14	4	3	4	6	13	7	4	4	6	4	8	6	5	5	3	4	4	4	2	3	7	4	2	6	1	3	3	6	4	2	7	6	2	5	5	6	4	1.265	0.632	–	–	I
K	3	4	4	2	5	6	5	2	4	7	4	4	2	7	4	2	3	1	2	2	3	3	5	4	5	3	4	3	4	11	5	3	2	4	3	4	7	6	1	6	3	6	4	7	2	4	6	5	5	4	5	0.820	0.373	1.32E-01	n.s.	K
L	8	8	8	7	8	9	6	10	2	8	7	3	8	7	9	3	11	4	6	8	9	6	4	8	9	11	6	11	7	4	6	5	5	4	4	6	1	5	2	4	8	7	6	5	6	1	5	8	2	4	9	0.687	0.284	4.60E-04	n.s.	L
M	1	3	1	1	1	2	4	3	2	0	1	1	3	2	3	2	2	4	3	0	5	2	1	3	1	0	2	0	1	0	4	1	3	1	2	3	3	2	0	3	1	1	3	0	0	4	1	4	1	0	4	0.455	0.338	5.58E-08	✻	M
N	1	5	2	6	4	1	3	4	2	5	4	3	2	2	6	2	1	4	3	2	1	6	1	2	4	8	3	3	7	4	6	5	4	3	3	5	11	3	4	12	5	3	3	8	5	1	3	3	2	6	3	1.307	0.794	–	–	N
P	4	5	6	4	2	5	7	4	3	5	8	3	3	3	4	2	5	6	3	4	1	4	3	4	5	5	5	4	5	5	1	3	2	7	5	3	2	4	5	4	5	2	5	4	2	5	2	5	5	2	5	0.800	0.308	4.17E-01	n.s.	P
Q	3	2	2	2	3	5	5	4	3	5	2	4	2	3	0	2	1	2	4	6	3	1	3	1	3	1	3	5	1	1	2	4	4	2	1	2	2	4	1	5	4	4	3	4	1	4	2	1	1	3	4	0.680	0.357	4.57E-03	n.s.	Q
R	7	1	3	5	3	6	2	4	8	5	7	2	4	3	3	2	2	4	7	1	2	3	1	4	3	2	4	3	2	4	5	2	5	3	6	2	3	6	4	2	2	1	3	3	3	2	4	3	2	7	3	1.167	0.596	–	–	R
S	5	1	3	4	10	6	3	9	2	5	9	9	7	3	8	3	7	2	7	5	2	3	3	3	5	6	8	7	5	5	4	4	5	7	6	7	8	11	10	9	5	8	12	12	13	7	7	8	9	11	8	0.795	0.368	5.23E-01	n.s.	S
T	6	8	4	8	11	6	2	7	2	3	11	13	5	3	3	10	2	3	3	6	8	5	8	4	6	1	9	3	3	5	5	5	8	2	4	6	3	14	5	8	7	6	14	5	6	16	3	10	6	10	7	0.889	0.498	8.69E-01	n.s.	T
V	11	13	8	10	5	13	9	4	5	14	7	7	11	8	5	13	6	3	2	16	9	4	7	9	8	5	5	9	3	5	7	7	8	5	2	6	6	4	7	7	7	11	11	7	7	10	9	8	11	17	8	0.978	0.425	–	–	V
W	0	1	0	4	3	1	0	0	0	0	1	1	0	0	0	0	0	1	0	0	0	1	0	3	0	1	1	0	0	4	1	2	0	0	0	0	0	0	0	0	1	0	0	1	1	1	2	0	1	1	0	–	–	–	–	W
Y	5	6	1	3	5	1	1	2	2	2	1	1	5	0	6	5	2	3	3	1	3	2	4	0	4	5	2	0	1	1	4	0	4	3	1	2	1	3	1	1	4	1	3	3	3	1	4	1	1	2	2	1.200	0.827	–	–	Y
total	84	96	75	94	87	102	82	80	62	91	100	90	88	77	75	76	65	68	85	89	68	74	77	77	86	78	79	87	54	76	81	76	84	75	73	81	77	94	70	93	78	87	104	83	77	87	72	92	69	92	90	0.904	0.115			total
