# Supplementary material for: Evolutionarily conserved bias of amino-acid usage refines the definition of PDZ-binding motif
Source: BMC Genomics. 2011 Jun 8;12:300. doi: 10.1186/1471-2164-12-300 (PMC3138430; doi:10.1186/1471-2164-12-300)
Supplement: Additional file 7 — 68 C-terminal peptides of mouse genome-encoded proteins possessing refined PB motifs and their bindings to PDZ domains. [file 1471-2164-12-300-S7.PDF]

Additional file 7

68 C-terminal peptides of mouse genome-encoded proteins possessing refined PB motifs and their bindings to PDZ domains

| Name | Sequence | Affinity |
|---|---|---|
| AcvR2 | VDFPPKESSL | y |
| AcvR2b | VDLLPKESSI | y |
| Cav1.2 | ADSRSYVSNL | y |
| Cftr | TEEEVQETRL | y |
| Claudin 23 | QNSLPCDSDL | y |
| Cnksr2 | HTHSYIETHV | y |
| CRIPT | DTKNYKQTSV | y |
| Dlgap1/2/3 | IYIPEAQTRL | y |
| EphA3 | TQSKNGPVPV | y |
| EphA4 | QQMHGRMVPV | y* |
| EphA5 | VQMVNGMVPV | n |
| EphB2 | QMNQIQSVEV | y |
| EphB3 | QMNQTLPVQV | y |
| EphB6_1 | HLRQPGSVEV | y |
| ErbB2 | PEYLGLDVPV | y* |
| Frizzled | TNSKQGETTV | y |
| GluR2_1 | NVYGIESVKI | y |
| GluR3 | NVYGTESVKI | y* |
| GluRdelta1 | ALDTSHGTSI | y |
| GluRdelta2 | GNDPDRGTSI | y |
| GRK6 | DSEEELPTRL | y |
| Htr2c | NVVSERISSV | y |
| KCNK3 | RGLMKRRSSV | n |
| KCNK4_2 | GRLRDKAVPV | y |
| KIF1B | NLKAGRETTV | y |
| Kir2.1 | PRPLRRESEI | y |
| Kir2.2 | VRPYRRESEI | y |
| Kir3.2_2 | VANLENESKV | y |
| Kir3.3 | LPPPESESKV | y* |
| Kir4.1 | SALSVRISNV | y |
| Kir4.2 | RSLLLQQSNV | y |
| Kv1.1 | VNKSKLLTDV | y |
| Kv1.2 | VNITKMLTDV | y |
| Kv1.3 | VNIKKIFTDV | y |
| Kv1.4 | SNAKAVETDV | y |
| Kv1.5 | CLDTSRETDL | y |
| Kv1.6 | YAEKRMLTEV | y |
| Kv1.7 | PAGKHMVTEV | y |
| Kv3.3_2 | FGERDSETQV | y |
| Kv4.1 | LPETVKISSL | y |

| Name | Sequence | Affinity |
|---|---|---|
| Kv4.2 | GGNIVRVSAL | y |
| L-glutaminase | LSKENLESMV | y |
| Megalin | ANLVKEDSDV | y |
| mGluR1 | RDYKQSSSTL | y |
| Na/Pi cotransporter | LPAHHNATRL | y |
| Nav1.4 | VRPGVKESLV | y |
| Nav1.5 | SPDRDRESIV | y |
| Nav2 | EEKASIQTQI | y |
| Neuroligin 2 | LPHPHSTTRV | y |
| NMDAR2A | KKMPSIESDV | y |
| NMDAR2B | EKLSSIESDV | y |
| NMDAR2C | RRISSLESEV | y |
| NMDAR2D | AHFSSLESEV | y |
| PDGFR | PLAEAEDSFL | y |
| PDGFRa_1 | SSDLVEDSFL | y |
| PDGFRa_2 | HSGKYDLSVV | n |
| PIX | NDPAWDETNL | y |
| PKC | FVHPILQSAV | y |
| PMCA1 | SPLHSLETSL | y |
| Sapk3 | GARVPKETAL | y |
| Sema4b | LGSEIRDSVV | y |
| Sema4c | PDSNPEESSV | y |
| Sema4f | PLATCDETSI | y |
| Stargazin | NTANRRTTPV | y |
| TRPC4 | AHEDYVTTRL | y |
| TRPC5 | GQEEQVTTRL | y |
| TRPM6 | RSSLEDHTRL | y |
| TRPV3 | ELDEFPETSV | y |

2-AA PB motif are underlined. Additional AAs contributing to the refined PB motifs are shown in red (see 'm' (mouse) column in Figure 4).

y :confirmed by Stiffler *et al.* and Chen *et al.*

y*:confirmed by others.

n :binding to PDZ is not confirmed

For details, see text.
